# Abstracts of the ICARE 2024 78th SIAARTI National Congress

**DOI:** 10.1186/s44158-024-00192-0

**Published:** 2024-10-08

## Anaesthesia in the frail patient

### A1 When a heart transplanted patient needs major abdominal surgery: is “awake neuraxial anesthesia” an option?

#### L. Mori, B. Basta, D. Vailati, E. Bonvecchio, G. Marino

##### Anesthesia and Intensive Care, Melegnano Hospital-ASST Melegnano e Martesana, Vizzolo Predabissi, Vizzolo Predabissi (Milano), Italy

###### **Correspondence:** L. Mori

*Journal of Anesthesia, Analgesia and Critical Care 2024*, **4(1):**A1

Background

In the surgical population, comorbidities, aging, and frailty are increasingly prevalent [1]. The decision to operate was sometimes challenging due to the difficult balancing of risks versus benefits. Many efforts are undertaken in order to minimize anesthesiological and surgical impact on physiological status and so to reduce perioperative complications. In this context a possible anesthesiological strategy for abdominal surgery is the so called “Awake Neuraxial Anesthesia”, that combines neuraxial anesthesia and mild sedation, keeping patient in spontaneous breathing.

**Case report**

A 72-year-old man, with Marfan syndrome and severe dilatative cardiomyopathy, received a heart transplant in 2020; he presented also recurrent episodes of pneumothorax, ischemic strokes without severe sequelae and chronic renal failure (CRF). In 2024, he was diagnosed with colon cancer necessitating right hemicolectomy. Among the issues was altered autonomic physiology due to donor heart denervation, resulting in preload-dependent heart [2]. Risks included also infectious complications due to immunomodulatory therapy, pneumothorax during mechanical ventilation, metabolic complications due to CRF. Following a multidisciplinary evaluation to explore strategies to minimize perioperative risk, we decided to perform neuraxial anesthesia in spontaneous breathing. Preoperatively, the patient was prepared according to the local ERAS protocol. Intraoperatively, standard monitoring (ECG, SpO2), invasive arterial pressure, semi-invasive cardiac monitoring (proAqt), BIS, capnography, and temperature sensor were applied. Segmental spinal anesthesia was performed at T9-T10 with levobupivacaine 0.5% 10 mg, dexmedetomidine 5 mcg, and 1 ml saline (total volume 4 ml, final levobupivacaine concentration 0.25%). Written informed consent was obtained with special regard to off-label intrathecal dexmedetomidine use. As expected, because of orthosympatic block, secondary to thoracic spinal anesthesia, hypotension occurred immediately post-intrathecal injection, promptly treated with noradrenaline infusion maintained throughout the procedure (maximum dose 0.18 mcg/kg/min); proAqt showed decreased systemic vascular resistance and preserved cardiac index. Furthermore an epidural catheter was placed at T7-T8 for postoperative analgesia and intraoperative use, administering 5 ml 1% lidocaine boluses (total 20 ml) during critical surgical steps. Intravenous sedation was achieved by dexmedetomidine infusion (0,1-1 mcg/kg/h) and 10 mg ketamine boluses (totaling 100 mg), resulting in a Richmond Agitation-Sedation Scale of -2/-1 and bispectral index (BIS™) > 80. Spontaneous ventilation was maintained with a 2 L/min oxygen mask throughout, arterial blood gases were checked at baseline and hourly, with P/F always > 300, CO2 and pH within range. The laparoscopic procedure lasted two hours without issues, with good surgical maneuverability according to the surgeon's opinion. Postoperative analgesia was ensured by 0.2% ropivacaine at 4-6 ml/hour rate via epidural catheter. Cautionary hospitalization in intensive care setting was mantained for 24 h. Postoperative period was uneventful without medical or surgical complications.

**Conclusion**

The approach of “Awake Neuraxial Anesthesia”, within advanced monitored anesthesia care framework, could represent an effective and safe option for high risk patient, even in major abdominal surgery.

Consent to data collection and publication was obtained from the patient.

References

1. Partridge JSL, Harari D, Dhesi JK. Frailty in the older surgical patient: a review, Age and Ageing, 2012;41(2):142–147.
2. Choudhury M. Post-cardiac transplant recipient: Implications for anaesthesia. Indian J Anaesth 2017;61:768–74.

### A2 Lumbar erector spinae plane block: a valid and safety anesthesiologic option for femure neck fracture surgery in elderly patients

#### G. Ranieri^1^, F. Rucci^1^, F. Pontecorvi^1^, M.G. Frigo^1^, A. Coviello^1^, A.U. De Siena^1^, A. Tognù^1^, F. Fattorini^2^, P. Ciocchetti^1^

##### ^1^Ospedale Isola Tiberina, Gemelli Isola, Roma, Italy; ^2^Department of Neurosciences, Reproductive and Odontostomatological Sciences, University Federico II, Napoli, Italy

###### **Correspondence:** G. Ranieri

*Journal of Anesthesia, Analgesia and Critical Care 2024*, **4(1):**A2

Background: Erector Spinae Plane Block (ESPB) was first described for use in thoracic chronic pain: it is based on the injection between transverse process and erector spinae muscle (1). In the last year, its use was extended to pain therapy and various types of surgery (2–6). Few cases reported the use of lumbar ESPB (L-ESPB) (7–9). However, the exact mechanism of action is still unclear. The main hypothesis is the lumbar plexus ventral branches travel in a tunnel formed by the fatty-muscular fibers structure between erector spinae muscle, transverse process, psoas compartment, and epidural space: this low-resistance tunnel provides a route for local anesthetic spread following a ESPB (10). Our study aims to show our experience with L-ESPB as anesthetic technique in elderly patients with femur fracture undergoing osteosynthesis with intramedullary nailing or screws.

Methods: This is a prospective observational single-center study, conducted between October 2023 and March 2024 at Gemelli Isola Tiberina Hospital. Patients with ASA-physical status III-IV undergoing femoral fracture osteosyntheses within 48 h of emergency access, in which neuraxial anesthesia and general anesthesia (GA) have too risks or contraindications due to anticoagulants treatment or severe comorbidities, were enrolled. After written informed consent, L-ESPB (Fig. 1-2) was performed with ropivacaine 0,5% 20 ml, lidocaine 2% 8 ml and 12 ml of normal saline in lateral position with the fractured limb up. Exclusion criteria were: inability to express written consent and allergy to local anesthetics. Under bispectral index (BIS) monitoring, propofol continuous endovenous (EV) infusion was used to maintain a BIS between 60 and 80; a bolus of 10 mg EV ketamine was administered, if an increasing of 15% of invasive blood pressure or facial grimaces occur (11). Primary outcomes were the needing of local anesthetic infiltration (iLA) and conversion to GA. Secondary outcomes were: static and dynamic numeric rating pain score (NRS) pre and 30 min after block, after 6, 12, 18, and 24 h; propofol intraoperative dosage (mg/Kg/h), number of ketamine 10 mg bolus, incidence of postoperative confusion, hours to first analgesic rescue dose request. Descriptive statistic was performed to analyze the variables. Continuous data were presented as median and interquartile range (IQR); dichotomous data were presented as absolute and relative frequencies.

Results: 24 patients met the inclusion and exclusion criteria (Table 1). No patients need iLA or GA to complete the surgical procedure. Before the L-ESPB all patients reported severe hip pain. 30 min after L-ESPB the static pain reduced from a median of 3 to 5. The L-ESPB showed a good control of post-operative pain (Fig. 3-4). Intraoperative median value of propofol and ketamine (was respectively 0,8 mg/Kg/h (IQR 0,5- 1,3) and 10 mg (IQR 0-18,8) (Table 2). Request for first rescue dose was at16 hours after the surgery (IQR 12—20). Symptoms of postoperative confusion was observed in 4 patients (17%) (Table 3).

Conclusion: In elderly patients with severity comorbidities undergoing femoral fracture surgery, L-ESPB combined with mild sedation and EV analgesia provided with ketamine and propofol could be a valid anesthetic option.

**Table 1:** **(abstract A2).** See text for description

N.	Age	Gender	Comorbidities	TAO	Fracture	Surgery	ASA
1	89	M	SY, CRF, HF (FE 35%), FA	Y	Subtrochanteric	I N	3
2	91	F	COPD, FA, HF(FE 30%), ICD, DM, SY	Y	Subtrochanteric	I N	4
3	93	M	CRF, IHD (PTCA stent), FA, AD	Y	Subtrochanteric	I N	3
4	86	M	DM, SY, AD, HF (FE 35%), RVA, INS-T	Y	Intracapsular	Screws	4
5	88	F	INS-A, AD, CRF, FA	Y	Subtrochanteric	I N	3
6	88	F	SY, DM, COPD IN CPAP Domic, FA, INS-T	Y	Subtrochanteric	I N	3
7	85	F	INS-A, IHD(PTCA stent), HF(FE 35%), FA	Y	Intracapsular	Screws	4
8	92	F	CRF, DM, SY, AD	N	Subtrochanteric	I N	3
9	95	M	COPD, SY, FA	Y	Intracapsular	Screws	3
10	90	M	AD, PEN, HF, FA	Y	Intracapsular	Screws	3
11	87	F	SY, DM, CRF, HF (FE 40%)	N	Subtrochanteric	I N	3
12	84	M	CRF, FA, INS-T, ST-A, HF(FE 25%), ICD	Y	Intracapsular	Screws	4
13	88	F	DM, AD, COPD, FA	Y	Subtrochanteric	I N	3
14	86	F	CRF, IHD(2 X BPAC), HF (FE 35%), INS-T	Y	Subtrochanteric	I N	4
15	90	M	ST-A, PMK, IRC, DM2	Y	Subtrochanteric	I N	3
16	88	F	FA, SY, HF (FE 35%) + PE	Y	Subtrochanteric	I N	3
17	85	F	SY, CRF, FA, HF (FE 35%), ST-M	Y	Subtrochanteric	I N	4
18	89	M	AD, CRF, COPD, IHD (2 X BPAC)	Y	Intracapsular	Screws	3
19	92	M	SY, DM2, HF, FA	Y	Subtrochanteric	I N	3
20	88	F	FA, SY, HF (FE 40%) + PE, PEN	Y	Subtrochanteric	I N	3
21	94	M	IHD, (PTCA stent), HF (FE 35%), PMK, DM2	N	Subtrochanteric	I N	3
22	89	F	FA, PMK, HF(FE 40%),DM	Y	Intracapsular	Screws	4
23	88	F	SY, COPD, AD, PEN, FA	Y	Intracapsular	Screws	3
24	87	M	IHD (2 X BPAC), SY, FA	Y	Subtrochanteric	I N	3

*IN* intramedullary nailing, *SY* systemic ypertension, *IRC* chronic renal failure, *HF* Heart Failure, *FE* eiection fraction, *FA* atrial fibrillation, *COPD* chronic obstructuive pulmonary disease, *ICD* implantable cardiac defibrillator, *DM* diabete mellitus, *IHD* ischemic heart disease, *PTCA* percutaneous transluminal coronary angioplasty, *AD* Alzheimer disease, *RVA* replacement valvol aortic, *INS-T* tricuspid insufficiency, *INS-A* aortic insufficiency, *PEN* pregress neurological event, *ST-A* aortic stenosis, *BPAC* coronary artery bypass, *PMK* pacemaker, *DM2* diabete mellitus 2 types, *PE* pleuric effusion, *ST-M* mitralic stenosis

**Table 2:** **(abstract A2).** Intraoperative variables

	Median	25th Percentile	75th Percentile
timing surgery (min)	45	45	50
Bromage Score 60 min	2	1	3
Target Propofol	0,8	0,5	1,3
Ketamine n. Bolus (10 mg)	10	0	18,8
Bispectral Index	70	68	75

**Table 3:** **(abstract A2).** Post-operative variables

Timing first rescue dose in h (Median, 25th-75th Percentile)	16	dic-20
Cognitive Impairment (n, %)	4	16,70%
Periprocedural complications (n, %)	0	0,00%
Intensive Care access (n, %)	10	41,70%

**Fig. 1:**
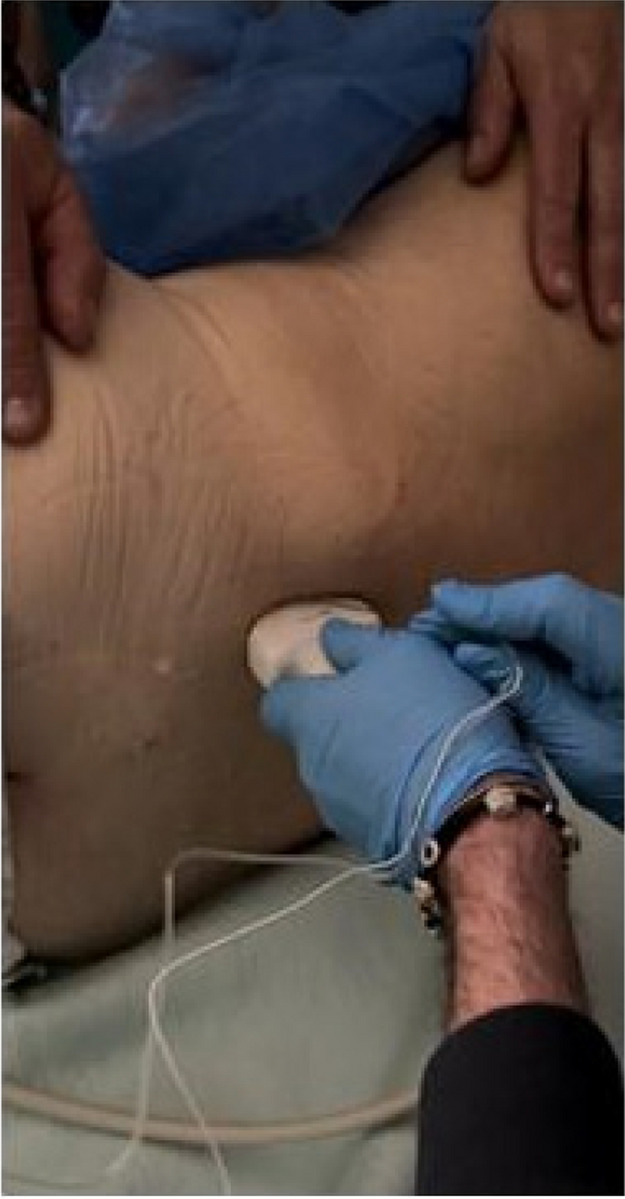
** (abstract A2).** L-ESPB lateral position, out-of-plane technique

**Fig. 2:**
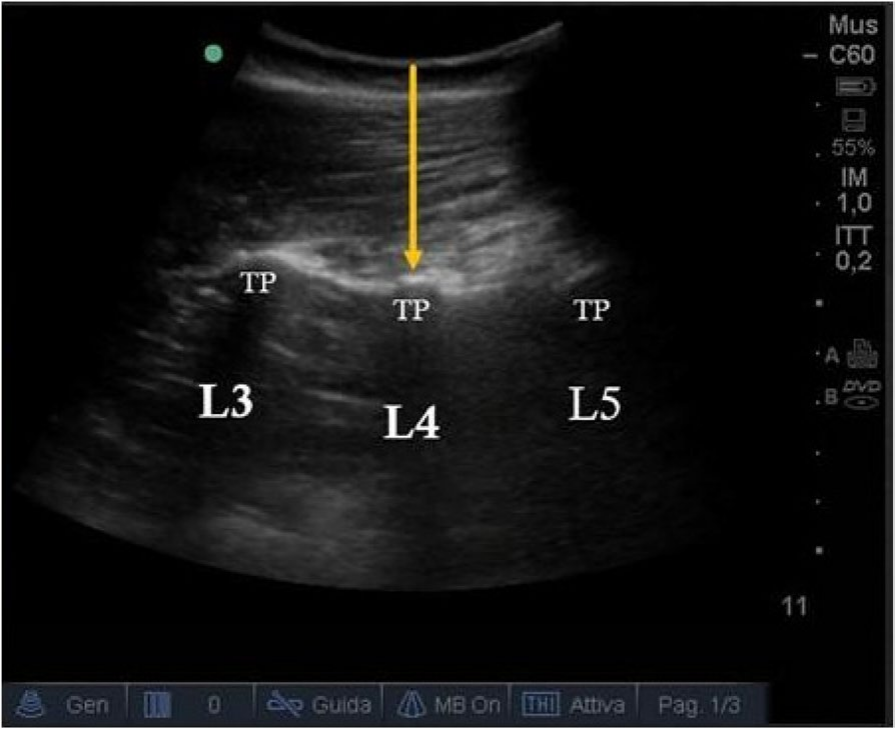
** (abstract A2).** Ultrasound image of the target point of the L-ESPB, also called “trident sign” (orange arrow=needle path, TP=transverse process, L3,L4,L5=lumbar level

**Fig. 3:**
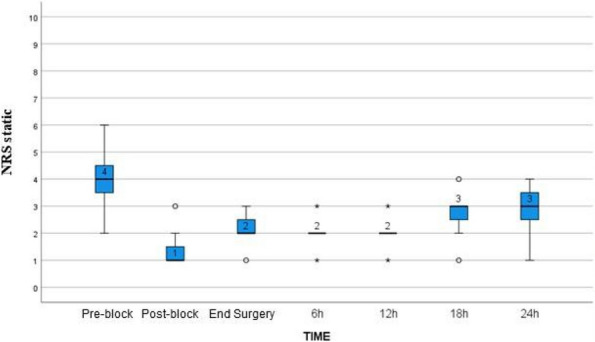
** (abstract A2).** NRS static pain at different time-point

**Fig. 4:**
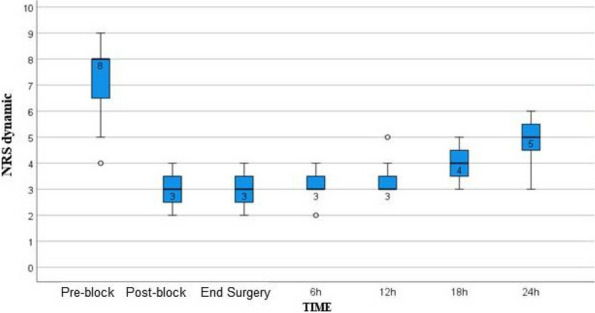
** (abstract A2).** NRS dynamic pain at different time-point

### A3 Post-operative complications after femur surgery: older does not mean worse

#### M. Fabris^1,2^, M. Comuzzi^1,2^, A. Poles^1,2^, M. Camolese^1,2^, F. Mazzon^1,2^, M. Grandesso^1,2^, G. Melchioretto^1,2^, G. Tripi^2^, T. Bove^1,2^

##### ^1^Department of Medicine (DMED), University of Udine, Udine, Italy; ^2^Department of Anesthesia and Intensive Care Medicine, ASUFC, University Hospital of Udine, Udine, Italy

###### **Correspondence:** M. Fabris

*Journal of Anesthesia, Analgesia and Critical Care 2024*, **4(1):**A3

**Background**

Proximal femur fractures (PFF) are one the leading causes of elderly patient’s hospitalization [1]. They represent a major health issue not only due to their increasing incidence but also in consideration of their potential to precipitate preexisting morbidities [2], particularly cardiovascular, pulmonary and neurological [3].

This study is aimed to evaluate the most common complications after hip-surgery in the elderly patient and to identify whether age could be a predictor for prolonged post-operative recovery.

**Materials and methods**

We retrospective enrolled all the patients whom underwent urgent orthopedic surgery for hip-joint fracture between January 1st and May 31st 2023 at Santa Maria della Misericordia hospital, Udine, Italy. Only patients aged more than 65 years, with anesthesia accomplished by loco-regional techniques (spinal anesthesia, peripheral nerve block or epidural catheter) were included. Polytraumatic injuries, defined as fractures involving more than two body districts, and patients who suffered high energy trauma (e.g. explosions, precipitations, road crashes) were not taken into account.

**Results**

About the 160 patients who underwent hip-surgery, 156 met the eligibility criteria. The population accounted for 114 females (73.1%) and 42 males (26.9%), with a median age of 85.6 ± 6.5 years (Fig. 1). The mean postoperative recovery was about 14.9 ± 7.1 days.

We further evaluated any perioperative complication within 60 days after surgery (Fig. 2):

- Infections (primarily pneumonia and urinary tract infections) affected 37 patients (23.72%);
- 30 patients (19.23%) experienced any neurological issue (minor or major stroke, cognitive impairment or delirium);
- Cardiac complications (acute on chronic of heart failure, arrhythmia, acute myocardial infraction) were seen in 26 patients (18.59%);
- Acute kidney injury (AKI) was diagnosed, according to KDIGO definition [4], in 20 patients (12.82%). Interestingly, 3 out of 20 patients (15%) who experienced AKI, did not show serum creatinine levels over the upper reference limit (according to our laboratory 0.95 mg/dL for women and 1.17 mg/dL for men).
- Overall mortality was about 5.77% (9 patients); 3 patients (1.92%) died within one month from surgery whereas 6 patients (3.85%) died between 31 and 60 days from surgery.

Statistical analysis (Spearman’s rank correlation) showed no correlation between age and postoperative stay (p = 0.094). Consistently with Nottingham Hip-Fracture Score classification [5], we further divided the population depending on the age: 65-85 years and older than 85 years; no difference was found between the two subgroups about the length of postoperative stay nor with perioperative complications.

**Conclusions**

According to the results of our study, in the specific setting of elderly population undergoing hip-fracture surgery, age cannot be considered as a risk factor for prolonged hospitalization (p > 0.05). Neither it could be assumed as an independent predictor for postoperative complications.

Second, as reported in our study, AKI criteria might be accomplished even without serum creatinine level elevation over the upper reference limit. In the specific setting of elderly, causes may be addressed to prolonged bed rest, sarcopenia and malabsorption, whose lead to lower creatinine dismiss from muscles. Such a situation may cause renal function impairment underdiagnosis and undertreatment.

**Fig. 1:**
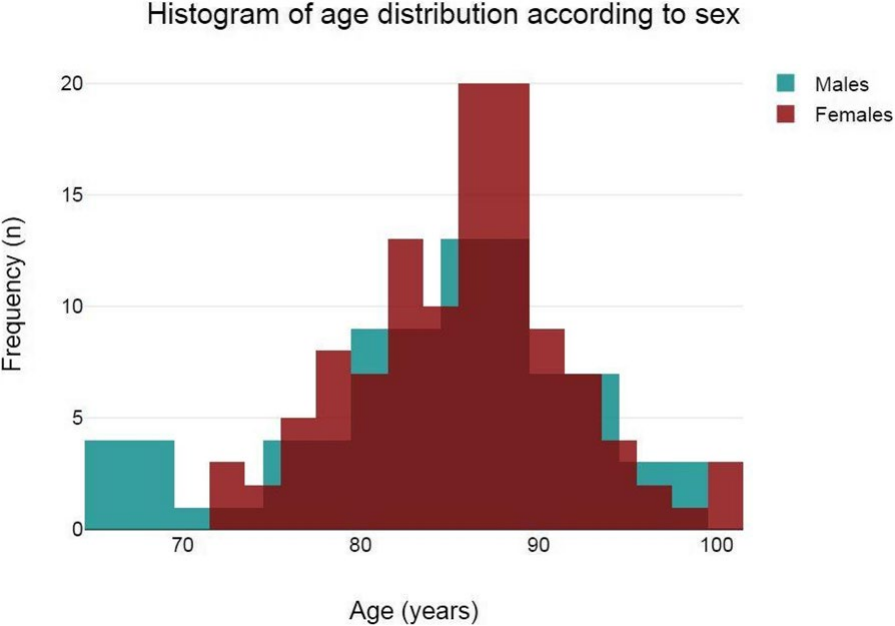
** (abstract A3).** See text for description

**Fig. 2:**
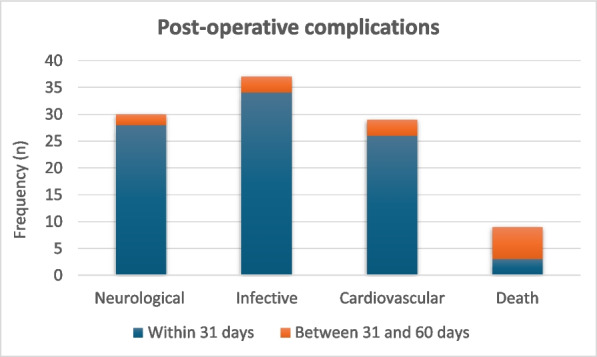
** (abstract A3).** See text for description

## Multi-organ donors and Anaesthesia and Intensive Care in organ transplantation

### A4 Gender issues in solid organ and tissue donation: a 20-year experience of the Maggiore Della Carità hospital in Novara

#### M. Zuliani^1^, G. Stefania^2^, V. Tesser^2^, M. Ronzani^2^, E. Riccardi^1^, F. Santangelo^1^, F. Moretto^1,2^, E. Shahi^1,3^, S. Gino ^3^, C. Izzo^4^, M. Candriella^2^, S. Piazza^5^, A. Puma^6^, E. Ceriani^6^, F. Grossi^2^, A. Germani^7^, R. Potenza^7^, G. Cammarota^1,8^, R. Vaschetto^1,2^

##### ^1^Università del Piemonte Orientale, Dipartimento di Medicina Traslazionale, Novara, Italy; ^2^Azienda Ospedaliero Universitaria Maggiore della Carità, Anestesia e Terapia Intensiva, Noavra, Italy; ^3^Azienda Ospedaliero Universitaria Maggiore della Carità, Direzione Medica,, Novara, Italy; ^4^Azienda Ospedaliero Universitaria Maggiore della Carità, Nefrologia,, Nobara, Italy; ^5^Azienda Ospedaliero Universitaria Maggiore della Carità, Cure Palliative, Novara, Italy; ^6^Azienda Ospedaliero Universitaria Maggiore della Carità, Medicina Interna, Novara, Italy; ^7^Coordinamento regionale donazioni e prelievi di organi e tessuti- Piemonte e Valle DAosta, Torino, Italy; ^8^ Azienda Ospedaliero Universitaria SS. Antonio e Biagio e Cesare Arrigo, Anestesia e Terapia IntensivA, Alessandria, Italy

###### **Correspondence:** M. Zuliani

*Journal of Anesthesia, Analgesia and Critical Care 2024*, **4(1):**A4

**Background**

Organ donation is affected by legal, social, religious, and ethnic factors as well as health concerns. Although organs themselves are gender-neutral, women account for up to two-thirds of all organ donations all over the world. There are no obvious reasons why women are more willing to undergo the risks of surgery than men, nor is this gender inequity mirrored in the demand for donated organs. In Italy, all citizens are required by law to declare their willingness to organ donation after death. Despite this, among the brain death and cardiac death patients, the percentage of consent expressed when one is alive remains low. Understanding the demographics of deceased patients and their willingness to donate organs is essential for effective transplantation programs. This study aims to provide a descriptive analysis of deceased donors in our hospital from 2003 to 2023 to find whether a potential gender difference in organ donation exists in our experiences.

**Methods**

We conducted a retrospective observational analysis of all the corneal and multiorgan donations from the “Maggiore della Carità” university hospital in Novara from 2003 to 2023. The Novara hospital is a 700-bed-hub organized hospital, with a 14-bed mixed intensive care unit (ICU) and an 8-bed cardiothoracic ICU, referencing the eastern Piedmont area in Italy. Data has been extracted from both electronic medical records in use at the hospital and the Valle d’Aosta and Piedmont Regional Coordination of Organ and Tissue Donation and Retrievals database. Results: In 20 years, 375 subjects resulted from brain-death donors. As far as gender differences, deceased multiorgan donors, 171 (46%) were females and 204 (54%) were males with the average age being 60 (15) years for females and 58 (18) for males. The donor percentage was higher in males for 15 years while for 5 years it was higher in females. From 2019 to 2023, data on the total number of ICU deceased subjects were available. Considering about 850 subjects entering the ICU every year, 75 (9%) female and 124 (19%) male subjects died in the ICU. The average percentage of multiorgan donors among those who died in ICU was 10% for females and 9% for males. As far as tissue donors are concerned, in 20 years, 665 subjects resulted cornea donors, 237 (36%) females and 428 (64%) males. Overall, the number of donors remained quite stable during these years, except for the year 2023, when a peak increase in corneal donation occurred due to the intense educational program that had been applied in 2022 to favour corneal donation in the hospital.

**Conclusion**

This study provides insights into the demographics of deceased donor patients in a university hospital in eastern Piedmont. More data is required to successfully investigate gender differences among donors, more specifically patient’s registration status on the Italian organ donor register (SIT) and in case of non-expression in life, which family member is entitled to decide on organ donation on their behalf.

### A5 Strategies for hemodynamic maintenance of potential brain-dead donor: our experience

#### G. Spinelli^1^, A. Carbone^1^, R. Russo^1^, M. Bova^1^, L. Tombolini^2^, N. Zarrillo^1^

##### ^1^ASL Caserta—Po San Rocco, Sessa Aurunca—Caserta, Italy; ^2^universita' Politecnica delle Marche, Ancona, Italy

###### **Correspondence:** A. Carbone

*Journal of Anesthesia, Analgesia and Critical Care 2024*, **4(1):**A5

**Background**

Organ transplantation is the most effective therapeutic alternative for many patients with end-stage diseases. Optimal donor management is essential to maximize the function of transplanted organs and the quality of life and survival benefits conveyed to the recipients. Hemodynamic support is an essential component of optimizing future allograft function: brain-death often results in an initial hypertensive crisis followed by hypotension.

Three main causes contribute to hemodynamic instability following brain-death, including:

- An initial sympathetic surge preceding medullary damage in brain death leads hypertension, left ventricular dysfunction, cardiac stunning, neurogenic pulmonary edema, and arrhythmias.
- The spinal cord infarction that follows herniation causes loss of sympathetic tone and hypotension.
- Pituitary loss of function.

Optimization of blood pressure is crucial to improve organ donation outcomes: guidelines recommend targeting a mean arterial pressure (MAP) of at least 60 to 65 mmHg in potential organ donor. Any intervention should be easily reversible, and the initial management of hypotension may require administration of vasoconstrictors to compensate the vasoplegia. The optimal vasopressor agent for organ donors has not been determined yet, so norepinephrine remains the first choice, but its potent alpha-agonist effects may exacerbate pulmonary capillary permeability and mesenteric and coronary vasoconstriction, thus reducing the acceptability of organs for transplantation.

**Discussion**

In January 2024, in our ICU, we procured two organ donors, that were managed with supportive measures to mitigate the above-described hemodynamic derangements that occur after brain-death. To ensure organ perfusion, we used the very short acting B1-blocker landiolol for the management of tachycardia and vasopressin plus low-dose norepinephrine for the management of vasoplegia.

All medications were used in continuous infusion: landiolol (started with a rate of 5 y/kg/min and increased as needed up to 10-15 y/kg/min) was effective to mitigate the hyperactivation of the sympathetic nervous system without adverse effect on the MAP or myocardial contractility. Vasopressin was initiated early at a dosage of 0.02 IU/min and increased up to 0,03 IU/min: However, it was necessary to add low-dose norepinephrine (starting from 0.2 y/kg/min up to 0.6 y/kg/min) to achieve a MAP of at least 60-65 mmHg.

The hemodynamic management of donors is complicated as elevated doses of vasoactive drugs can reduce the perfusion of organs to be harvested, making the fine tuning of their administration essential to optimize their post-transplant function. Actually, Landiolol, a very short-acting B1-blocker, decreases the heart race with less negative effect on blood pressure or myocardial contractility and Vasopressin acts as a vasoconstrictor by stimulating the non-adrenergic V1 receptor if administered in supraphysiologic concentrations, without direct effects on heart rate or vasoconstriction on the pulmonary arteries. Vasopressin analogues may also be helpful in the management of vasodilatory shock: and are increasingly used to reduce the dose of the first-line noradrenergic agent. Our strategy of early use of vasopressin to manage vasoplegia of donors, although off label, may reduce adrenergic vasopressors doses and the related complications and is supported by pathophysiological bases and evidence of increased rate of organ recovery.

### A6 Orthotopic liver transplantation in acute-on-chronic liver failure patients: survival and post-operative course

#### M. Monfroni, M. Checchi, M.L. Bindi, G. Biancofiore

##### Department of Surgical, Medical, Molecular Pathology, and Critical Care Medicine, University of Pisa, Pisa, Italy

###### **Correspondence:** M. Monfroni

*Journal of Anesthesia, Analgesia and Critical Care 2024*, **4(1):**A6

**Background**

In the past decade, numerous studies have assessed the survival and post-operative outcomes of patients with acute-on-chronic liver failure (ACLF) who undergo liver transplantation. Despite encouraging results, eligibility of ACLF patients for Orthotopic Liver Transplantation (OLT) is still on debate.

**Aim**

To evaluate post-transplant survival, intra and post-operative course of Decompensated Cirrhosis (DC) patients with or without acute-on-chronic liver failure.

**Methods**

We conducted a monocentric retrospective analysis of liver transplant recipients with decompensated cirrhosis over a three-year period from January 1st, 2020, to December 31st, 2022, at the Liver Transplant Center of the University Hospital of Pisa. ACLF diagnostic criteria and severity grading classification followed those outlined by the European Association for the Study of Liver-Chronic Liver Failure (EASL-CLIF) in the CANONIC study. Data collected included demographic information, MELD score, MELD-Na score, CLIF-C OF score, and CLIF-C ACLF score, calculated immediately pre-transplant. Intra-operative considerations comprised blood product usage, vasopressor or inotropic support, and reperfusion syndrome incidence. Post-operative evaluation included ICU length of stay (LOS), duration of mechanical ventilation, and post-operative complications assessed by the Comprehensive Complication Index (CCI) score. Data were compared between DC transplant patients with and without ACLF. Post-transplant survival of both groups was reported with a maximum follow-up of three years and six months.

**Results**

During the study period, 447 orthotopic liver transplants were performed, with 165 (36.9%) for decompensated cirrhosis. Among these, 21 patients (12.7%) met ACLF diagnostic criteria. ACLF patients were significantly younger [51yrs. (48 – 54) vs. 57yrs. (52.8 – 63); p = 0.05] and had higher median leukocyte counts [7.8 (5.6 – 9.4) × 109/L vs. 4.2 (3 – 5.9) × 109/L; p < 0.0001]. Alcohol-related cirrhosis was the predominant etiology. ACLF patients demonstrated significantly higher blood unit consumption [5 (3 – 7) vs. 3 (1 – 5); p = 0.007], incidence of reperfusion syndrome (57.1% vs. 22.2%; p = 0.002), requirement for vasopressor (90.5% vs. 67.4%; p = 0.039) and inotropic support (14.3% vs. 2.8%; p = 0.045) during the intra-operative period. Median ICU LOS [8 (5 – 16.5) vs. 5 (4—7); p = 0.003] and CCI scores [52.2 (42.7 – 99) vs. 29.6 (15 – 41.9); p < 0.0001] were also significantly higher in ACLF patients. The 1-year survival rate in ACLF patients and no ACLF patients was respectively 71,4% (95% CI 54,5 – 93,6) vs. 89,3 (95% CI 84,3 – 94,6) (Fig. 1).

**Conclusions**

Our findings indicate promising survival outcomes following OLT in ACLF patients, albeit with significant differences compared to non-ACLF patients. ACLF patients experienced more complex intra and post-operative courses, necessitating greater economic and healthcare resource utilization.

Informed consent was obtained.

**Fig. 1:**
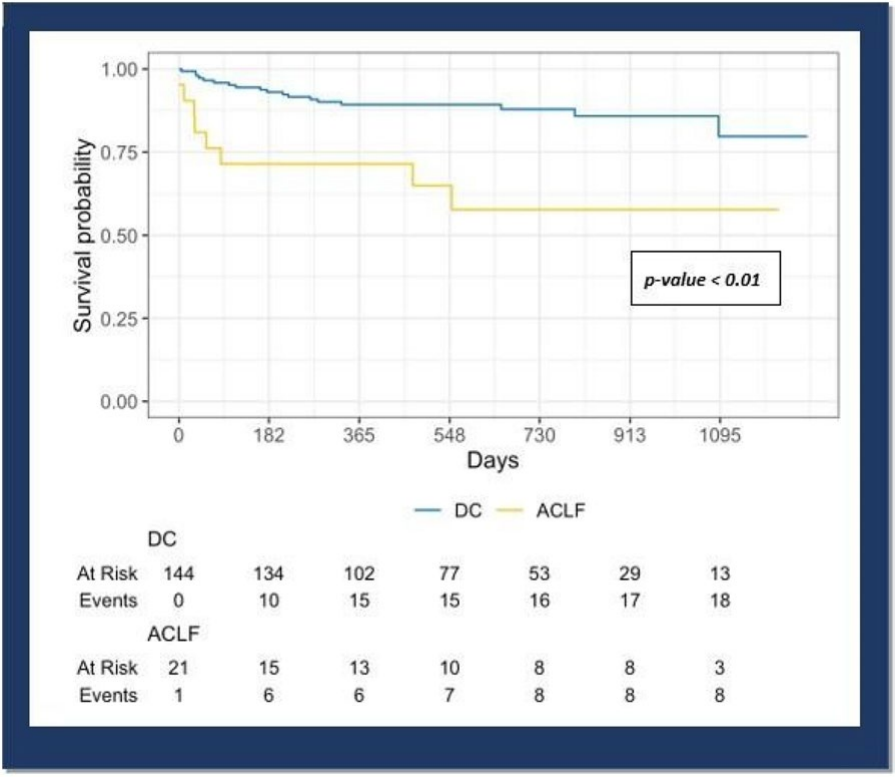
** (abstract A6).** Survival probability curves (Kaplan-Meier) of transplanted patients with decompensated cirrhosis alone (DC) and with ACLF. Log-rank test was used to compare the Kaplan-Meier curves

### A7 Preconditioning and organ protection in donation after circulatory death

#### P. Zanatta^1^, F. Linassi^1,2^, F. Mazzon^3^, G. Fullin^1^, L. Polesello^1^, E. Nascimben^1^, G. Feltrin^4^, G. Gerosa^5^

##### ^1^Department of Anesthesia and Intensive Care, Ca' Foncello Treviso Regional Hospital, Treviso, Italy; ^2^Department of Pharmaceutical and Pharmacological Sciences, University of Padova, Padova, Italy; ^3^Department of Medicine, Anesthesia and Intensive Care Clinic, University of Udine, Udine, Italy; ^4^Regional Transplant Center, Veneto Region, Padova, Italy; ^5^Cardiac Surgery Unit, Cardio-Thoraco-Vascular and Public Health Department, Padova University Hospital, Padova, Italy

###### **Correspondence:** F. Mazzon

*Journal of Anesthesia, Analgesia and Critical Care 2024*, **4(1):**A7

**Background**

Donation after circulatory death (DCD) is an accepted strategy to expand the potential donor pool. The complexity of organ procurement from DCD donors requires the development of new strategies for organ protection, including normothermic regional perfusion (NRT) or extracorporeal membrane oxygenation (ECMO) and also a pharmacological preconditioning protocol. For this purpose, a preconditioning protocol is routinely employed by our team for each DCD donation, before and during the withdrawal life sustaining treatment (WLST) and after NRT/ECMO (Fig. 1). The protocol includes pharmacological treatments combined to reduce oxidative stress (melatonin, N-acetylcysteine, ascorbic acid), improve microcirculation (statins) and mitigate organ’s ischemic injury (steroids) and ischemia/reperfusion injury (remifentanil).

However, there is currently no data available on tissue or functional organ damage after DCD, apart from organ transplantation outcomes.

**Methods**

To evaluate the protective effect of our protocol on organ preservation, we design two groups: the DCD patients performed in our center (case group), and the second group with the same individuals but after the first out-of-hospital cardiac arrest. For both groups we collect AST (Ul/L), ALT (Ul/L), Creatinine (mg/dL) and Hemoglobin (g/L) values after one hour from the NRT/ECMO start and one hour from Return of Spontaneous Circulation (ROSC). We also compare Low Flow Time (LFT) and No Flow Time (NFT) of both groups. To compare data from the groups we used Wilcoxon Signed-Rank test calculator, considering a significant p value as < 0,05.

**Results**

Nine DCD transplants using the NRT/ECMO were performed in our hospital (case group). The control group consisted of six patients who experienced a first cardiac arrest prior to hospital admission. Time of no flow was significantly lower in the control group than in the case group (p < 0,05), being 285% longer in the patients with DCD protocol. Hemoglobin was significantly lower in the case group (p value < 0,05). No other differences were found among the other variables considered Table 1.

**Conclusions**

The similar tissue-damage variables between the groups, despite a significantly longer no-flow time in the case group receiving our preconditioning protocol, suggests its protective effect with potential benefits for organ donors.

Informed consent was obtained.

**Reference**

1. Gerosa G, Zanatta P, Angelini A, Fedrigo M, Bianco R, Pittarello D, Lena T, Pepe A, Toscano G, Zanella F, Feltrin G, Pradegan N, Tarzia V. Overcoming the Boundaries of Heart Warm Ischemia in Donation After Circulatory Death: The Padua Case. ASAIO J. 2024 Feb 9. 10.1097/MAT.0000000000002141. Epub ahead of print. PMID: 38334806.

**Fig. 1:**
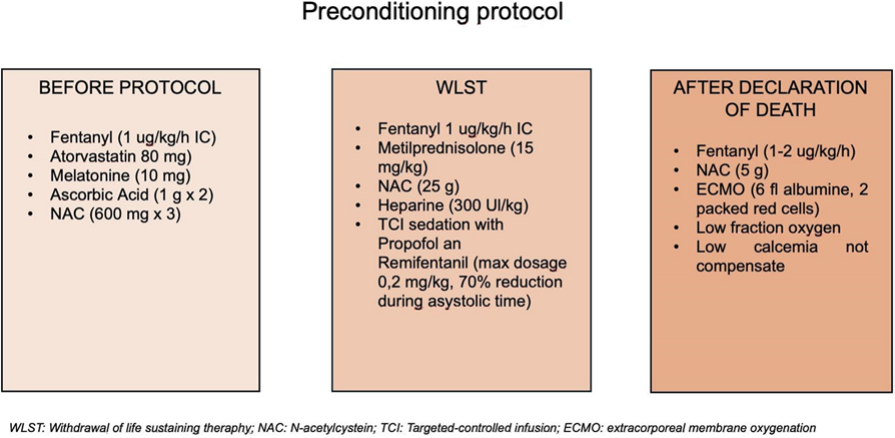
** (abstract A7).** Preconditioning protocol used in our center

**Table 1:** **(abstract A7).** Descriptive analysis of population of the study and statistics results

Variables	DCD patients n. 9	Control group n. 6	W-value	W critical value (*p* > 0,05)
Age, yrs	63 (12)	62.5 (8.5)	3	0
Male/Female	9/0	6/0	-	-
ICU recovery, days	12 (8)	13.5 (6)	-	-
No flow time, min	34 (8)	10 (7.5)	0	0
Low flow time, min	15 (6)	28.5 (22.25)	3	0
Creatinine (mg/dL)	0.9 (0.29)	1.02 (0.0.75)	3	0
AST (Ul/L)	88 (50)	315.5 (261.25)	3	0
ALT (Ul/L)	88 (145)	339.5 (396)	4	0
Hemoglobin (g/L)	9.9 (0.7)	13.9 (0.65)	0	0

Data are reported as median and Interquartile ranges

*ICU* Intensive Care Unit, *AST* aspartate aminotransferase, *ALT* alanine transaminase

### A8 Training in corneal tissue sampling techniques: 4 years of experience of a sicilian team

#### L. Mazzeo, M.F. Crupi, F. Corallo, F. Anastasi, M. Pagano, S. Leonardi

##### IRCCS Centro Neurolesi Bonino-Pulejo, Messina, Italy

###### **Correspondence:** L. Mazzeo

*Journal of Anesthesia, Analgesia and Critical Care 2024*, **4(1):**A8

**Background**

Organ and tissue donation is a topic of increasing interest in the scientific community. The 'Anesthesia and Resuscitation Unit' of the IRCCS Centro Neurolesi Bonino-Pulejo in Messina has been committed to raising community awareness of this issue for years. It is also active in the ongoing training of team to ensure an effective and efficient service based on latest scientific evidence and the needs that have emerged from performance evaluations over time.

**Methods**

Our team recorded an increase in corneal tissue donation between 2020-2024. During this period, specific training on this procedure has been carried out to operate effectively among the facilities on Sicilian territory.

**Results**

Training performed on anaesthesiologists enabled an effective management of the increasing corneal tissue donation. The number of tissue samples increased despite the prolonged absence of ophthalmologists. The trend of corneal donation performed in our unit is summarized in Fig. 1.

**Discussions**

Psychological training on communication with caregivers has proven to be crucial in sensitive situations such as the death of family members and loved ones, but also on communication to raise community awareness on these issues. In this context, training on tissue harvesting techniques to be implemented and the continuous training of doctors involved in tissue transplantation are also crucial to the success of the intervention {1}. Therefore, training of anaesthetists in tissue harvesting techniques can improve the process of organ and tissue donation and transplantation. From this perspective, multidisciplinary team is a key resource in current healthcare. Teamwork allows individual medical specialties to be respected while at the same time establishing collaborations between anesthesiologists and their colleagues from other specialties in sharing retrieval techniques to promote the timeliness of harvest procedures and reduce possible negative effects due to staff gap in hospitals {2,3}. Community awareness of organ and tissue donation is valuable, and the community's confidence could be strengthened by knowing the clinical experience of the team in their region {4,5}.

Informed consent was obtained from all participants.

**References**

1. Ong, H. S.,et al. (2022). 'Endothelium-Out' and 'Endothelium-In' Descemet Membrane Endothelial Keratoplasty (DMEK) Graft Insertion Techniques: A Systematic Review With Meta-Analysis. Frontiers in Medicine, 9, 868533.
2. Wykrota, A. A. et al. (2022). Approval rates for corneal donation and the origin of donor tissue for transplantation at a university-based tertiary referral center with corneal subspecialization hosting a LIONS Eye Bank. BMC ophthalmology, 22, 1–12.
3. Collins, J. W., et al. (2020). Surgical training benefits from the cumulative outcomes of proficiency-based training and mentorship. JAMA surgery, 155(7), 616–616.
4. Cho, W. H. (2020). Causes of donation failure and improvement measures analyzed based on data from domestic deceased donors in 2019. Korean Journal of Transplantation, 34(4), 219.
5. Park, S. Y. et al. (2022). Exploring the experiences and perspectives of emergency physicians on brain death organ tissue donation after the Life-Sustaining Treatment Decision Act. Korean Journal of Transplantation, 36(1), 29.

**Fig. 1:**
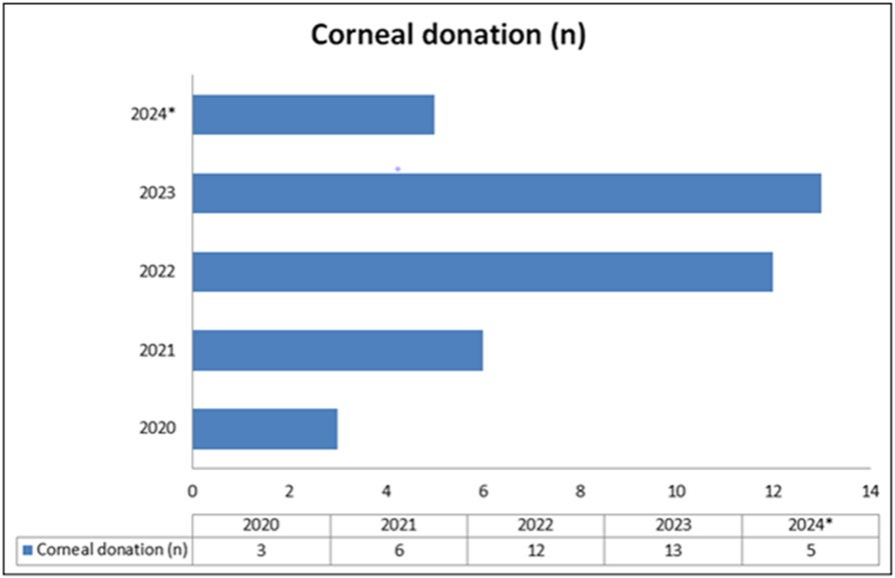
** (abstract A8).** Summary trend of tissue donation at the 'Anesthesia and Resuscitation' Unit of the IRCCS Centro Neurolesi Bonino-Pulejo P.O. Piemonte. Legend: *2024 in progress

### A9 Towards interdisciplinary shared protocols to manage organ procurement in forensic deaths: the experience of Puglia region in promoting the culture of donation

#### T. Ciciriello^1^, C. Musajo Somma^2^, L. Gesualdo^2^, M. Bellino^3^, S. Sablone^3^, F. Introna^3^, V. Malcangi^1^, F. Puntillo^1^

##### ^1^Università degli Studi di Bari Aldo Moro—Anestesia, Rianimazione e Terapia Intensiva e del Dolore, Bari, Italy; ^2^Centro Regionale Trapianti, Bari, Italy; ^3^ Università degli Studi di Bari Aldo Moro—Medicina Legale, Bari, Italy

###### **Correspondence:** T. Ciciriello

*Journal of Anesthesia, Analgesia and Critical Care 2024*, **4(1):**A9

Organ transplantation is one of the most important contributions of modern medicine to society since it provides a decisive therapeutic alternative for terminal organ failure.[1] The majority of organ donors (> 90%) are represented by patients deceased after the irreversible cessation of the whole brain function (i.e. brain death) in Intensive Care Units where the organ donation process begins. Organ procurement is a multi-phasic and multi-disciplinary process in which the intensivist gets fully involved throughout the entire transplantation trial, including identifying the potential donor and its correct management according to law procedures, organ withdrawal and peri-operative management of the receiving patient. [2]

In Italy organ procurement is currently reaching good results which are comparable to or even better than those achieved in other European countries. However, there is an enormous disproportion between organ demand and supply and still remain barriers to donation, including those represented by potential deceased donors under medico-legal jurisdiction. [3]

For such cases, recovery procedures cannot be performed until the Public Prosecutor has issued a specific authorization, therefore, it is necessary to ensure the justice interest in death investigation without compromising the organ donation process. Currently, it seems that justice interests prevail over ethical-humanitarian ones. [4]

National transplant center (CNT) has recently adopted the operational guidelines contained in the document issued by the Lombardia Region, but there is no interdisciplinary (legally and medically) national consensus on how to best perform organ donation when it intersects forensic investigations and, as a result, many possibilities of donation get lost. [5]

A possible way to fix this issue was proposed already in 2015 by the Puglia Region with a shared operational standard procedure between the judicial authority and the organ procurement team. [6]

This work aims to analyze the impact of such an initiative in the Puglia region and compare it to the national trend by a retrospective analysis of Regional Transplant Center and CNT archives from 2009 to 2023. The study showed that, unlike the national trend, the apulian recommendations have contributed to lowering oppositions by the judicial authority to organ and tissue procurement in forensic deaths (Fig. 1).

These findings underline how the use of joint protocols and quick communication between all parties involved would allow overcoming ostensible conflicts between judicial and health needs while the intensivist, following the process from the first steps, could play a key role in coordinating and speeding up the whole procedure, helping to exploit the precious instrument of organ donation at its best.

**References**

1. Maciel CB, et al., Curr Neurol Neurosci Rep. 2016;16(9). 10.1007/s11910-016-0682-1
2. Rohrig SAH et al., Qatar Med J. 2020;2019(2):1–2. 10.5339/qmj.2019.qccc.12
3. Trapianti CN. LISTE D’ATTESA AL 10/05/2024.; 2018. https://trapianti.sanita.it/statistiche/liste_attesa_1.aspx
4. Shafer TJ, et al., Am J Transplant. 2004;4(2):160–168. 10.1046/j.1600-6143.2003.00327.x
5. Aurelio MT, et al.,' PROCUREMENT E PROCURE '.; 2007. https://www.trapianti.salute.gov.it/imgs/C_17_cntPubblicazioni_361_allegato.pdf
6. Sablone S, et al., J Forensic Leg Med. 2024;102(December 2023):102657. 10.1016/j.jflm.2024.102657.

**Fig. 1:**
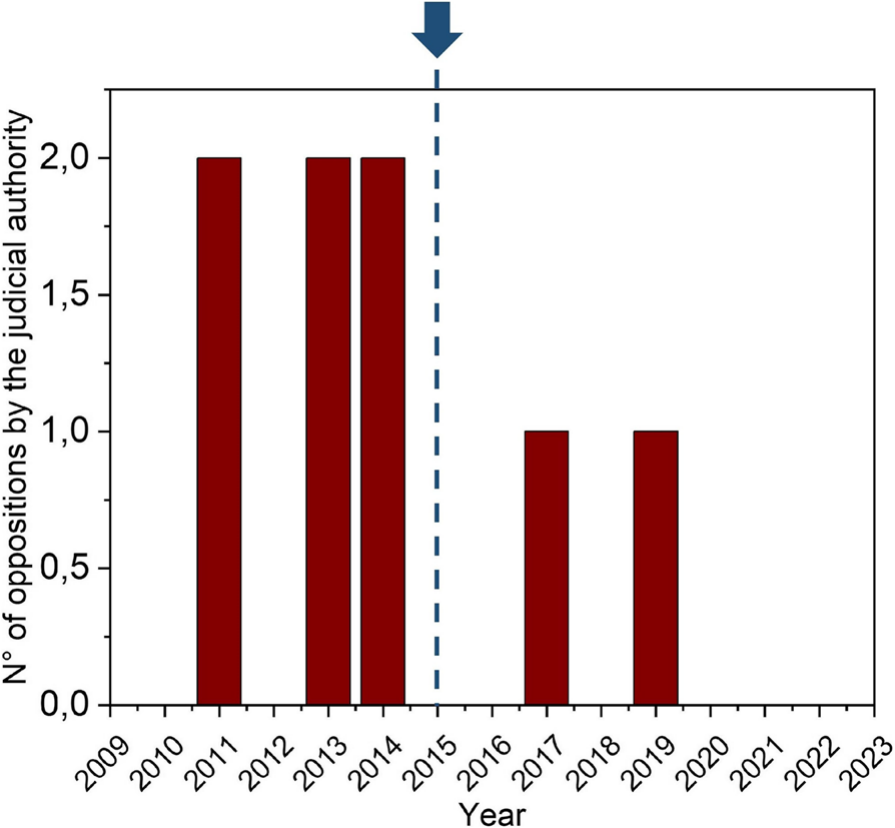
** (abstract A9).** Number of oppositions by the judicial authority during the period 2009-2023 and their decrease after the apulian initiative of 2015

### A10 Early prone positioning as a rescue therapy for moderate-to-severe primary graft dysfunction after bilateral lung transplant

#### M. Bisi^1^, G. Gianino^1^, F. Monteleone^1^, P. Zanon^1^, M.F. Toma^1^, M. Della Paolera^1^, T.A. Giacon^1^, A. Boscolo^1,2^, T. Pettenuzzo^2^, N. Sella^2^, P. Navalesi^1,2^

##### ^1^Department of Medicine, University Hospital of Padua, Padua, Italy; ^2^Anesthesia and Intensive Care, Univeristy Hospital of Padua, Padua, Italy

###### **Correspondence:** M. Bisi

*Journal of Anesthesia, Analgesia and Critical Care 2024*, **4(1):**A10

**Background**

One of the most challenging problems affecting survival after lung transplant (LT) is primary graft dysfunction (PGD), a form of acute lung injury occurring up to 72 h after surgery. PGD is characterized by diffuse radiological bilateral infiltrates and refractory hypoxemia with arterial partial pressure of oxygen (PaO2) to fractional inspired oxygen (FiO2) ratio below 300 mmHg in the moderate form known as PGD2, and below 200 mmHg in severe one, defined as PGD3. PGD has been reported to occur in almost 30% of LT and is associated with longer hospital length of stay and higher 90-day mortality. Even if available therapies are mainly supportive [1], promising results from recent findings have been obtained with prone positioning (PP). PP has already shown great benefits in patients with acute respiratory distress syndrome (ARDS) [2; 3].

**Aim of the study**

The hypothesis of this study is that PP, a rescue maneuver to treat refractory hypoxemia due to PGD, may improve LT outcomes especially when applied early.

**Materials and Methods**

All consecutive bilateral- LT recipients developing moderate-to-severe PGD within 24 h from intensive care unit admission were enrolled (Fig. 1). Specifically, from January 2020 to November 2021, patients developing PGD after LT were monitored in ‘supine position’ for at least 24 h before considering PP: only in case of radiological or oxygenation worsening, patients were turned prone, generally between 24-48 h after diagnosis (‘late PP’). After November 2021, patients were routinely turned prone within 24 h from PGD (‘early PP’). A propensity score weighting analysis with clinically covariates was applied.

**Results**

One hundred and thirty LT patients were screened and sixty seven enrolled. Twenty-five (37%) recipients were treated in ‘supine position’, twenty four (36%) in ‘early PP’ and eighteen (27%) in ‘late PP’. After propensity score weighting, both ‘supine’ treatment (estimate 8.23, standard error 2.97, p = 0.007) and ‘early PP’ treatment (estimate 9.42, standard error 2.59, p < 0.001) were associated with greater 28-day ventilator free time than ‘late PP’ (Table 1). Moreover, ‘early PP’ was associated with better oxygenation, driving pressure, static respiratory system compliance as compared to ‘late PP’ and improved PaO2/FiO2 at 72 h, as compared to ‘supine position’ (Fig. 2).

**Conclusions**

‘Early PP’ in LT recipients with moderate-to-severe PGD is associated with greater 28-day ventilator free time, better oxygenation, compliance and driving pressure than’late PP’ group.

Informed consent was obtained.

**References**

1. Avtaar Singh SS et al., ISHLT Primary Graft Dysfunction Incidence, Risk Factors, and Outcome: A UK National Study. Transplantation. 2019 Feb;103(2):336–343.
2. Guérin C, et al., PROSEVA Study Group. Prone positioning in severe acute respiratory distress syndrome. N Engl J Med. 2013 Jun 6;368(23):2159–68.
3. Schmidt M, et al., PRONECMO Investigators, the REVA Network, and the International ECMO Network (ECMONet). Prone Positioning During Extracorporeal Membrane Oxygenation in Patients With Severe ARDS: The PRONECMO Randomized Clinical Trial. JAMA. 2023 Dec 26;330(24):2343–2353.

**Table 1:** **(abstract A10).** Data are expressed as number and (percentage), median and [interquartile range] or mean and standard deviation. Primary outcome balanced according to propensity score weighting procedure for the variables used in the propensity score estimation. For secondary outcomes we defined as “baseline” arterial blood gas (ABG) the worst sample collected during the first 24 h after ICU admission

	**Early PP ** ***N*** ** = 24 (36)**	**Late PP ** ***N*** ** = 18(27)**	**Supine position ** ***N*** ** = 25 (37)**	**Overall ** ***p*** **-value**	**Early PP vs Late PP**	**Early PP vs Supine**	**Late PP vs Supine**
**Unadjusted primary outcome**							
Ventilator-free days (range 28 days)	26 [19–26]	12 [2–20]	25 [21–26]	**0,002**	**0,001**	0,966	** < 0,001**
**Adjusted primary outcomes**	**Estimate coefficent**	**Standard Error**	**p-value**				
Early PP vs Late PP	9,42	** < 0,001**	** < 0,001**				
Ealrly PP vs Supine	1,19	0,656	0,656				
Supine vs Late PP	8,23	**0,007**	**0,007**				
**Secondary Outcomes**							
**Baseline**							
PaO_2_/FiO_2_	215 ± 57	182 ± 61	224 ± 54	0,057	0,117	0,537	0,057
Driving Pressure cmH2O	12 ± 2	11 ± 1	11 ± 2	0,594	0,703	0,937	0,703
Crs ml/cmH2O	34 ± 10	36 ± 6	36 ± 9	0,672	0,741	0,741	0,741
**At the end of PP**							
PaO_2_/FiO_2_	330 ± 65	250 ± 62	-	** < 0,001**	-	-	-
Driviing Pressure cmH2O	11 ± 1	12 ± 3	-	0,733	-	-	-
Crs ml/cmH2O	39 ± 13	33 ± 6	-	0,435	-	-	-
**12 h after re-supination**							
PaO_2_/FiO_2_	332 ± 70	230 ± 53	-	** < 0,001**	-	-	-
Driviing Pressure cmH2O	8 ± 6	14 ± 1	-	**0,041**	-	-	-
Crs ml/cmH2O	48 ± 9	37 ± 4	-	**0,017**	-	-	-
**72 h after PGD diagnosis**							
PaO_2_/FiO_2_	309 ± 72	242 ± 33	250 ± 39	**0,002**	**0,002**	**0,002**	0,261

*ABG* arterial blood gas analysis, *PaO2/FiO2* ratio between arterial oxygen partial pressure and inspiratory oxygen fraction, *PaCO2* arterial carbon dioxide pressure, *DP* driving pressure, *Crs* compliance respiratory system, *ICU* intensive care unit, *h* hours, *n* number, *PGD* primary graft dysfunction

**Fig. 1:**
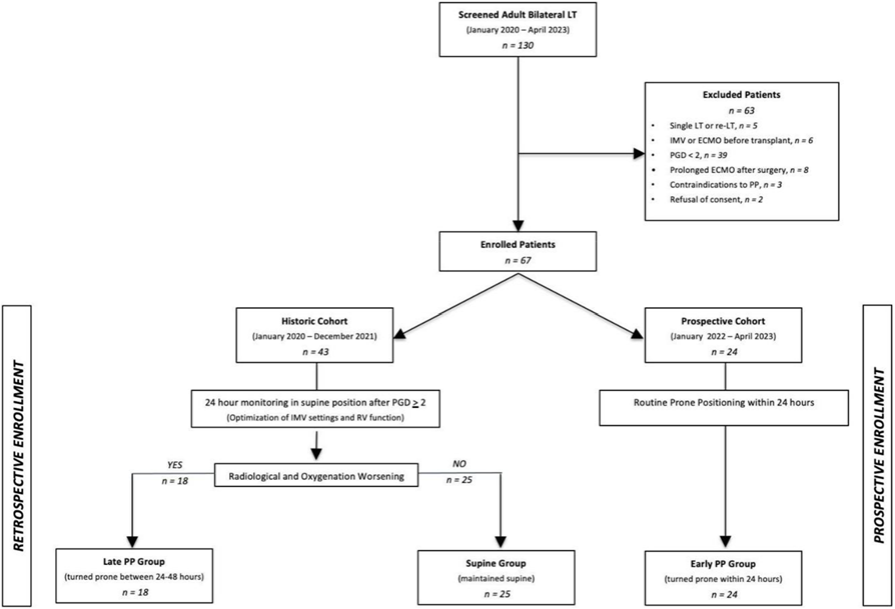
** (abstract A10).** Study flowchart

**Fig. 2:**
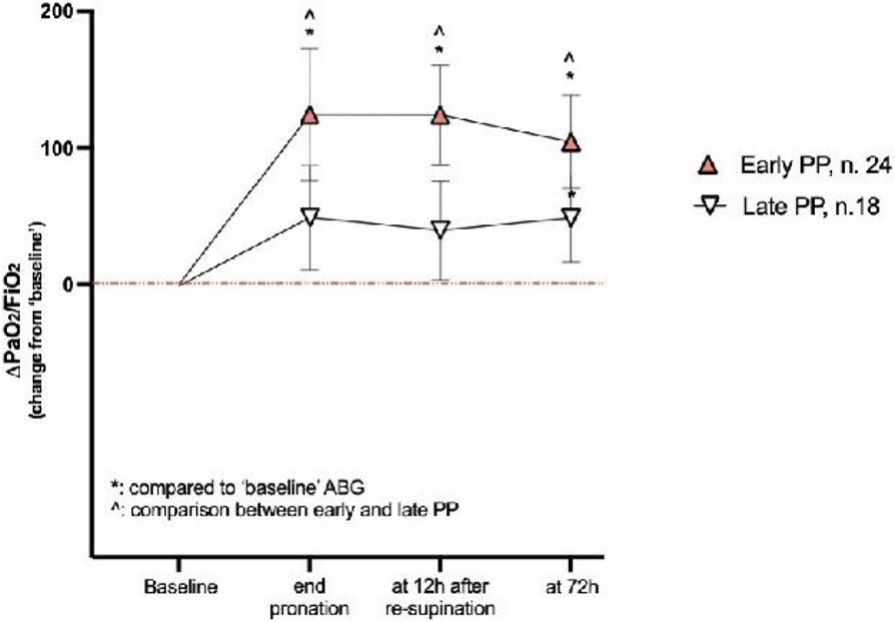
** (abstract A10).** ∆PaO2/FiO2 at the end of prone position

## Safety, quality and clinical risk

### A11 Early warning score an early warning tool: observational study to identify strategies for effective use

#### M. Sena^1^, A. Dacomi^1,2^, S. Brusa^1,2^, E. Azzolini^1,2^, M. Greco^1,2^, B. Mazzoleni^2^

##### ^1^IRCCS Istituto Clinico Humanitas, Rozzano, Italia, Milano, Italy; ^2^Humanitas University, Milano, Italy

###### **Correspondence:** M. Sena

*Journal of Anesthesia, Analgesia and Critical Care 2024*, **4(1):**A11

**Background**

Regular assessment of patients' vital signs and symptoms is crucial to promptly identify and address any clinical deterioration. This practice helps nurses objectively evaluate and address concerns during bedside clinical evaluations [1]. For this reason, validated tools have been developed for timely recognition and communication of clinical changes, such as the multi-parametric National Early Warning Score (NEWS) [2].The modified NEWS score has been in use at IRCCS Istituto Clinico Humanitas (ICH) (Fig. 1) as a clinical monitoring tool for all in-patients across hospital wards. However, the limitations in daily use, such as occurrence of delayed EWS registration or incomplete data for EWS calculation, are not know. This study aims to observe the area of improvements in EWS application in a large academic medical center, focusing on delayed or incomplete EWS registration.

**Methods**

We conducted a quality-improvement retrospective observational study extracting data from ICH electronic health record, looking at patients aged 18 or more across all surgical and medical wards for more than 24 h in the year 2023 (1st Jan-31st Dec), focusing on incomplete (gray) or expired EWSs. In addition, a 12-question survey was administered to nurse coordinators from each ward, to identify possible area of improvements.

**Results**

We included 472,523 admissions. Expired EWS data were largely more frequent than incomplete EWS (67.6% vs 4%). Yearly frequencies of expired EWS were represented as bar plot over daily time for each ward (Fig. 2), and as heatmaps showing their percent incidence for each nursing shift and each ward (Fig. 3). The occurrence of expired EWS were higher at time of nurses' shift changes and nursing handovers. Additionally, there was a higher prevalence of expired EWS in wards were nurse coordinators reported inherent limitations in their use due to the unique characteristics of the patient population (i.e. patients with with pre-existing altered vital parameters caused by a specific disease, resulting in frequent baseline alterations in EWS scores).

**Conclusions**

Expired EWS are frequent and depend on nurse perception of EWS clinical utility to grant patient safety and on the characteristics of admitted patients. To reduce expired EWS incidence, we proposed two improvement strategies. On one side, to delegate collection of vital parameters during handovers to supporting stuff, and on the other side to introduce an automatical countdown alert to notify nurses when the EWS expiration time is approaching, allowing for better care planning and patient monitoring. We plan to test the effect of these interventions in subsequent pre-post quality study.

Informed consent was obtained.

**References**

1. Douw G, Schoonhoven L, Holwerda T, Huisman-de Waal G, van Zanten ARH, van Achterberg T, et al. Nurses’ worry or concern and early recognition of deteriorating patients on general wards in acute care hospitals: A systematic review. Crit Care
2. Williams B. The National Early Warning Score: From concept to NHS implementation. Clinical Medicine, Journal of the Royal College of Physicians of London. 2022 Nov 1;22(6):499–505.

**Fig. 1:**
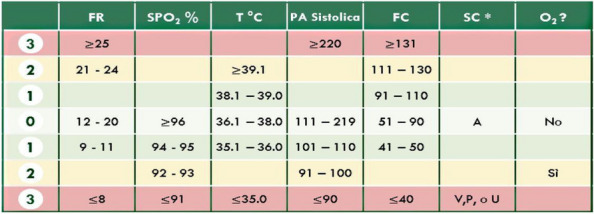
** (abstract A11).** Early warning score scheme used in ICH

**Fig. 2:**
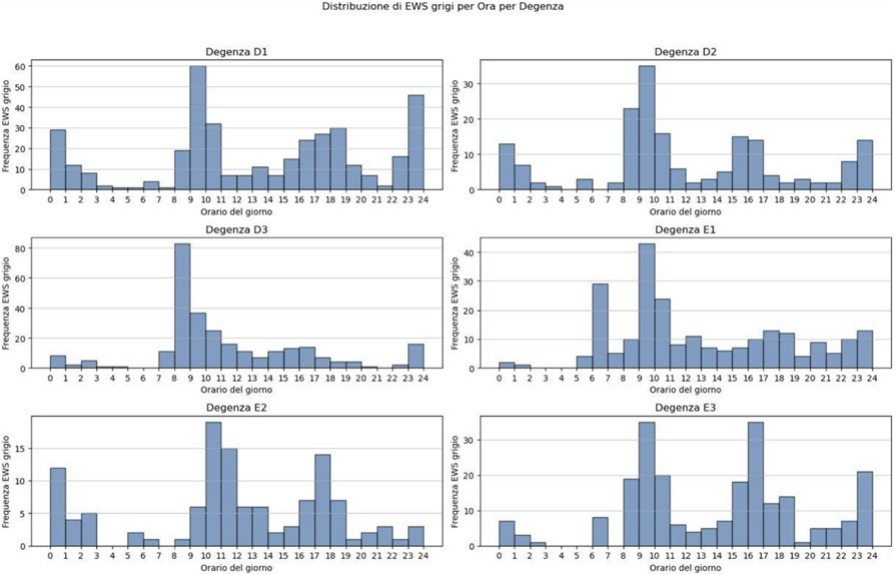
** (abstract A11).** EWS expired distribution by hour and by ward

**Fig. 3:**
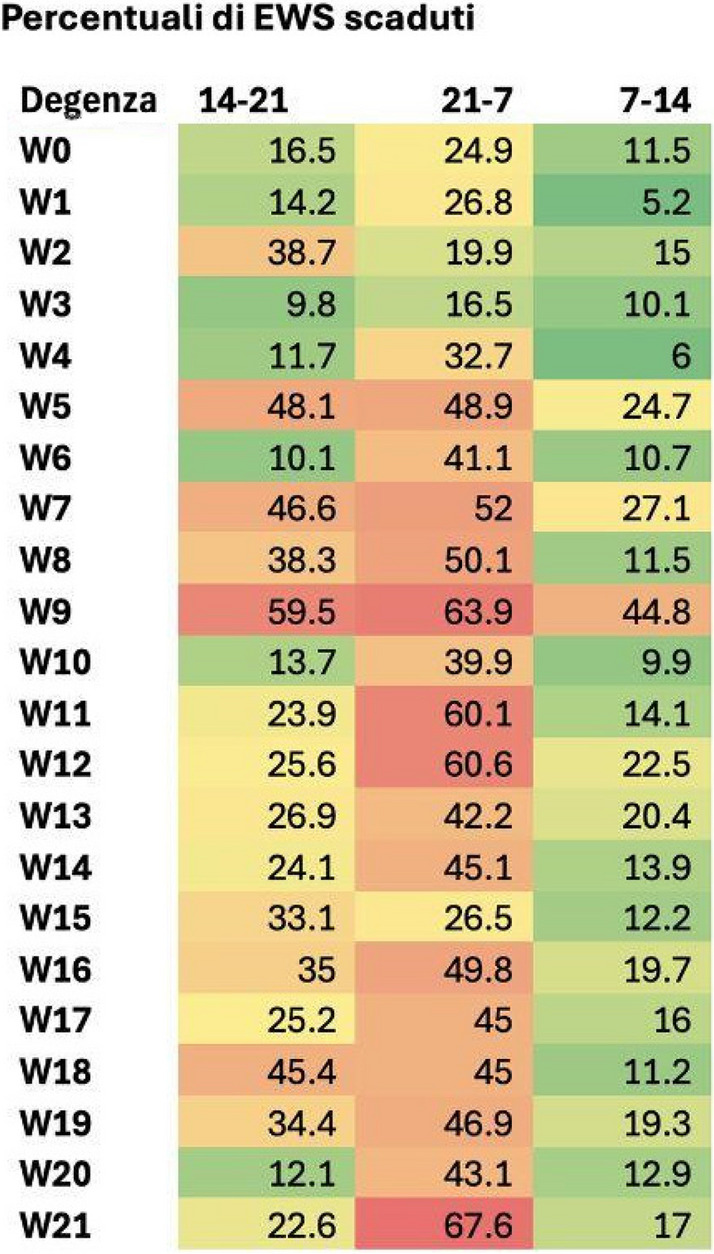
** (abstract A11).** Heatmap: EWC expired distribution by nurses’ shift wards

### A12 Best CICC is the CICC that doesn’t need to be replaced, Securacath® in the ICU

#### F. Tomeo, I. Calamai, M. Luchini, R. Spina

##### SOC Anestesia e Rianimazione ospedale S.Giuseppe, Empoli, Italy

###### **Correspondence:** F. Tomeo

*Journal of Anesthesia, Analgesia and Critical Care 2024*, **4(1):**A12

**Introduction**

Dislocation is one of the most common causes of complications related to centrally inserted venous catheters (CICCs). The critically ill patient in intensive care needs life-saving infusion therapies, therefore the dislocation of a vascular device turns into a lack of therapy for the time necessary for repositioning, with the risk of progression of the disease, destabilization of vital parameters as well as complications related to dislocation and repositioning.

The literature has demonstrated that the use of sutures for fixing vascular devices is absolutely to be avoided. Adhesive-type sutureless devices have been used for years and require accurate management due to the danger of detachment in the event of profuse sweating of the patient or excessive traction.

The SecurAcath® is a new subcutaneous anchoring device that has proven to be very effective in minimizing catheter migration. Originally conceived by MichealRosemberg and approved by the FDA in July 2010 and the CE mark in January 2011. The pourpose of this study is to record the incidence of displacement and complication of CICCs in the ICU fixed with SecurAcath® and compare it with that highlighted by international literature.

**Methods**

The SecurAcath® is a subcutaneous catheter securement system. The device utilizes a small anchor that is placed just beneath the skin at the catheter insertion site and is attached to the catheter shaft. The device is available in different sizes, from 3 to 12F. SecurAcath® was applied to secure the device In all patients in whom a CICC was necessary during hospitalization in the ICU, after obtaining the consent of the patient or of their relatives. The study does not imply any difference in the quality of care and interventions carried out on patients. The type of device, the date of implantation and removal and the reason for its replacement and its complications are recorded in an anonymous database for subsequent data analysis.

**Results**

We report the preliminary data of the study. Currently, 48 patients have been enrolled and 60 CICCs have been implanted. 34 patients had the CICC removed due to clinical need, 14 patients were discharged with the CICC. 12 CICCs were replaced: 7 for systemic fever where a CRBSI could not be excluded, 1 for CICC malposition, 1 for CICC rupture, 3 for accidental removal or self-removal of the patient. No SecurAcath® was replaced due to breakage of the anchoring device itself. No insertion site infections, injury or dermatities were recorded.

**Conclusion**

Out of 60 CICCs only 3 were displaced with a displacement rate of 5% (7.3%, 4.4/1000 days/catheter). Clinical studies on the use of SecurAcath® for PICCs demonstrate a catheter dislodgement rate (1.5%, 0.7/1000 days/catheter). International data reports a dislocation rate of 12% for adhesive fastening systems. There were no complications directly related to the use of securAcath. No malpositioning of the SecurAcath® due to operator error was recorded. The study needs to be expanded in the sample and the cost-effectiveness of the product must be explored.

## Transfusion, haemostasis and thrombosis

### A13 A case of Shiga Toxin-producing *Escherichia* coli Hemolytic-Uremic Syndrome (STEC-HUS) in an adult

#### P. Ferrara, T. Tassinati, G. Dallocchio, A. Pinamonti, F. Tartari, M. Vason

##### Azienda Ospedaliero-Universitaria di Ferrara, Ferrara, Italy

###### **Correspondence:** P. Ferrara

*Journal of Anesthesia, Analgesia and Critical Care 2024*, **4(1):**A13

**Background**

STEC-HUS is a disease that mainly affects children under the age of 5 (70% of cases in children under 3 years old; mortality 3–5%) [1,2]. This is why we report the case of an adult.

**Case report**

On January, a 58-year-old woman, returning from a trip to Zanzibar, went to the emergency department for worsening crampy abdominal pain and the hemorrhagic transformation of diarrhea that had already been present for a few days. Suspecting bacterial colitis, the woman was admitted to the infectious diseases department where she was prescribed empirical antibiotic therapy. In the following days, the neurological state worsened until, three days after hospital admission, the woman appeared unable to follow simple orders; acute kidney injury, thrombocytopenia and increased LDH were also associated. In addition, stool culture was positive for shiga-like toxin-producing enterohemorrhagic E. coli. Suspecting STEC-HUS, the nephrologist indicated to continue the hydrating therapy. At the time of our evaluation, the woman's neurological status and renal function appeared to be improving and it was believed there were no indications for admission in ICU. However, during the night, laboratory tests were suggestive of significant hemolysis, for which it was decided to admit the woman to ICU. In agreement with nephrologists and haematologists it was decided to perform daily Therapeutic Plasma Exchange (TPE) sessions (three TPE sessions). Two days after ICU admission, epileptic seizures and coma complicated the neurological state of the woman who, consequently, was intubated. The renal function indices had also progressively worsened, therefore, the woman underwent a one hemodialysis session. Two days after intubation, the platelet count returned to normal. Furthermore, once she emerged from the coma, she was extubated. However, three days later, a second phase of thrombocytopenia and increased hemolysis indices were observed for which three further sessions of TPE were performed. In the following days, a slow but complete recovery of her neurological status was observed. Two weeks after admission, the woman was discharged from ICU. During the follow-up 3 months after ICU discharge, an informed consent to the publication of the data was collected from the woman.

During the post-ICU discharge follow-up, an informed consent to the publication of the data was collected from the woman.

**Conclusion**

The optimal management of STEC-HUS in adults is a challenge. A multidisciplinary approach and supportive care, especially a correct management of fluid therapy, are fundamental but the appropriateness of additional therapies, such as TPE, remains to be clarified [1,2].

**References**

1. Michael M, Bagga A, Sartain SE, Smith RJH. Haemolytic uraemic syndrome. Lancet. 2022 Nov 12;400(10364):1722–1740.
2. Freedman SB, van de Kar NCAJ, Tarr PI. Shiga Toxin-Producing Escherichia coli and the Hemolytic-Uremic Syndrome. N Engl J Med. 2023 Oct 12;389(15):1402–1414.

### A14 Incidence of asymptomatic catheter-related thrombosis in intensive care unit patients: a prospective cohort study. Secondary analysis of thromboelastographic data

#### G. Turconi^1^, C. Bonetti^1^, I. Silvestri^1^, A. Guzzardella^1^, C. Tomarelli^1^, G. Zimei^1^, F. Cappelli^1^, C. Abbruzzese^2^, M. Brioni^2^, G. Grasselli^1,2^, M. Panigada^2^

##### ^1^Dipartimento di Fisiopatologia Medico-Chirurgica e dei Trapianti, Università degli Studi di Milano, Milano, Italy; ^2^S.C. Anestesia e Terapia Intensiva Adulti, IRCCS Ca' Granda Ospedale Maggiore Policlinico, Milano, Italy

###### **Correspondence:** G. Turconi

*Journal of Anesthesia, Analgesia and Critical Care 2024*, **4(1):**A14

**Background**

The placement of central venous catheters (CVC) in Intensive Care Unit (ICU) is a common procedure as it is essential for the treatment and monitoring of most patients. However, it may be associated with various complications, including catheter-related thrombosis (CRT), that can occur asymptomatically or can result in catheter dysfunction, pulmonary embolism, and catheter related bloodstream infection (CRBSI) [1].

We have recently demonstrated in a prospective cohort study a relatively high prevalence of asymptomatic CRT (25% of the patients) [2].

Viscoelastic point-of-care tests, such as thromboelastography (TEG®; Hemoscope Corporation, Niles, IL, USA), provide global information on the dynamics of clot development stabilization, and dissolution, reflecting in vivo hemostasis. A recent meta-analysis showed that TEG was able to detect hypercoagulability starting on post-op day one [3]. Aim of this study was to verify the hypothesis that TEG parameters were different in patients who developed CRT compared to patients who did not.

**Materials and methods**

This is a pre-planned secondary analysis of a cohort prospective study on the incidence of asymptomatic catheter-related thrombosis in intensive care unit patients [2]. From September 14, 2019 to March 31, 2022 patients admitted to the ICU of IRCCS Ca’ Granda Ospedale Maggiore Policlinico di Milano who needed a CVC were considered for enrollment. Exclusion criteria were: 1)age < 18 years; 2)history of neoplasia or thrombophilia. Informed consent was obtained. Duplex ultrasound screening was performed using Rapid Central Vein Assessment (RaCeVA) protocol before the first CVC positioning and then daily to diagnose CRT. TEG (TEG® 5000 analyzer) on fresh whole blood activated with kaolin and using heparinase cups, was performed at enrollment and on day 3.

TEG parameters are presented as median and 25th-75th quartiles. Mann–Whitney test was used to compare patients who developed CRT vs. those who were CRT free (no-CRT).

**Results**

203 patients were enrolled in the study. 65% were males, and the median age was 60 [49–70] years. 25% of patients developed a CRT. The median time interval between catheter placement and CRT was 5 [3–10] days. Data regarding TEG parameters on day 1 and 3 are presented in Table 1 and 2, respectively. TEGs were performed in 130 patients (34 CRT and 94 no-CRT) on day 1 and in 110 patients (28 CRT and 82 no-CRT) on day 3. No difference was found in any of the TEG parameters analyzed between CRT and no-CRT patients (Fig. 1).

**Conclusions**

None of the TEG parameters collected on the day of catheterization and on day 3 is associated with the subsequent asymptomatic catheter-related thrombosis development.

**References**

1. Timsit JF, Farkas JC, Boyer JM, et al. Central vein catheter-related thrombosis in intensive care patients: incidence, risks factors, and relationship with catheter-related sepsis. Chest. 1998;114(1):207–213.
2. Abbruzzese C, Guzzardella A, Consonni D, et al. Incidence of asymptomatic catheter-related thrombosis in intensive care unit patients: a prospective cohort study. Ann Intensive Care. 2023;13(1):106.
3. Brown W, Lunati M, Maceroli M, et al. Ability of Thromboelastography to Detect Hypercoagulability: A Systematic Review and Meta-Analysis. J Orthop Trauma. 2020;34(6):278–286.

**Table 1:** **(abstract A14).** TEG parameters on day 1 in CRT and no-CRT patients

	Overall, median [p25; p75]	No-CRT, median [p25; p75]	CRT, median [p25; p75]	*p*
R (min)	6.0 [4.2; 8.1]	6.0 [4.2;8.3]	6.1 [4.3;7]	0.68
Angle (°)	66.75 [58.3;72.8]	66.75 [58.2;72.4]	66.85 [58.6;73.0]	0.75
MA (mm)	74.4 [65.8;79.4]	74.35 [64.3; 79.35]	76.5 [69.9;79.9]	0.22
Ly30 (%)	0.2 [0; 1.5]	0.2 [0; 1.5]	0.15 [0; 1.6]	0.93

**Table 2:** **(abstract A14).** TEG parameters on day 3 in CRT and no-CRT patients

	Overall, median [p25; p75]	No-CRT, median [p25; p75]	CRT, median [p25; p75]	*p*
R (min)	5.8 [4.7; 8.3]	5.8 [4.6; 8.4]	5.85 [4.85;7.3]	0.69
Angle (°)	64.7 [55.2; 71.4]	64.7 [54.3; 70.6]	64.4 [56.7;74.0]	0.33
MA (mm)	73.0 [65.6; 79.0]	73.0 [64.1;79.5]	72.6 [66.1; 70.2]	0.92
Ly30 (%)	0.0 [0.0; 1.2]	0.1 [0.0; 1.7]	0.0 [0.0; 0.4]	0.35

**Fig. 1:**
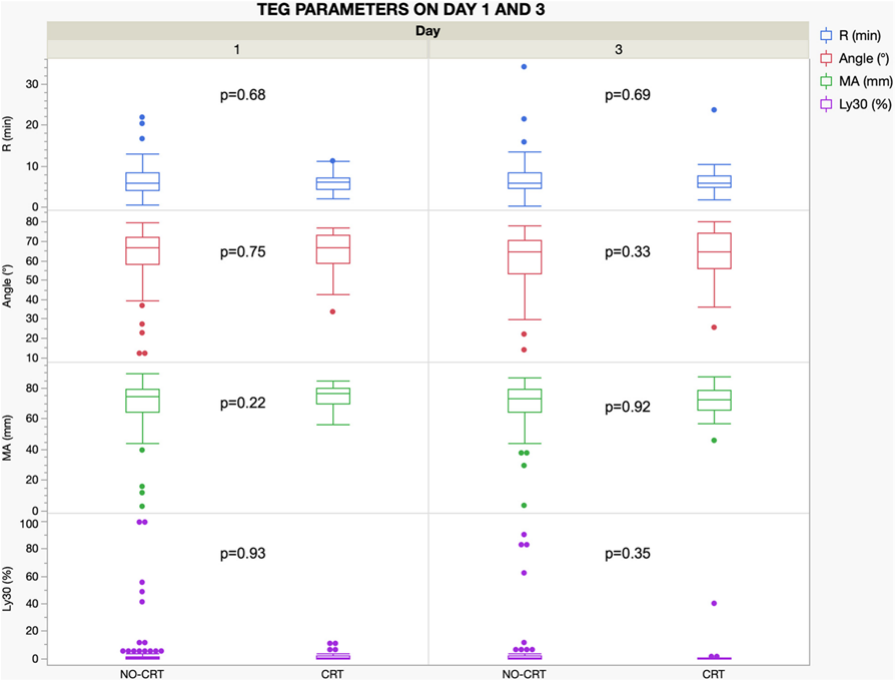
** (abstract A14).** TEG parameters on day 1 and 3

## Underwater and hyperbaric emergencies

### A15 Circulating microvesicles in acute carbon monoxide poisoning

#### A. Puggioni^1^, S. Nembrini^2^, A. Burgner^1^, T. Esposito^1−3^, A. Chiocchetti^2−4^, G. Cappellano^2−4^, M. Manfredi^1−5^, C. De Luca^1^, M.M. Tomasino^1^, L. Mesiano^1^, G.F. Massidda^1^, L. Scotti^1^, S. Bazzano^3^, S. Guido^3^, G.L. Vignazia^3^, A. Bolognini^6^, G. Cammarota^1−7^, A. Giovanniello^8^, F. Della Corte^1−3^, R. Vaschetto^1−3^

##### ^1^Università del Piemonte Orientale, Dipartimento di Medicina Traslazionale, Novara, Italy; ^2^Università del Piemonte Orientale, Dipartimento di Scienze della Salute, Interdisciplinary Research Center of Autoimmun, Novara, Italy; ^3^Azienda Ospedaliero Universitaria Maggiore della Carità, Anestesia e Rianimazione, Novara, Italy; ^4^Università del Piemonte Orientale, Center for Translational Research on Autoimmune and Allergic Disease-CAAD, Novara, Italy; ^5^Università del Piemonte Orientale, Dipartimento di Scienze ed Innovazione Tecnologica, Novara, Italy; ^6^Centro Iperbarico Sassarese, Sassari, Italy; ^7^Azienda Ospedaliera SS. Antonio e Biagio e Cesare Arrigo, Anestesia e Rianimazione, Alessandria, Italy; ^8^Casa di Cura HABILITA I Cedri, Fara Novarese, Italy

###### **Correspondence:** A. Puggioni

*Journal of Anesthesia, Analgesia and Critical Care 2024*, **4(1):**A15.

**Background**

Carbon monoxide (CO) is responsible for several accidental intoxications and deaths every year worldwide. CO poisoning can induce cellular damage due to hypoxia and inflammation, which might be associated with severe delayed neurological sequelae (DNS), but the underlying mechanisms are still poorly understood. Hence, we aimed to investigate the role of microvesicles (MVs) in CO-related damage pathogenesis.

**Materials and methods**

The approval of the Ethics Committee was obtained, and all participants provided written informed consent. Patients admitted to the Emergency Department (ED) with a documented CO-intoxication and scheduled for hyperbaric oxygen therapy (HBOT) were enrolled. Two blood samples were taken: on ED admission (T0), and after HBOT (T1). Twelve age-, sex- and smoke habit-matched subjects were enrolled as control group. Population characteristics and clinical data were collected, and a 45-day follow-up for DNS was performed. Plasma MVs were analyzed with flow cytometry, and subpopulations of leukocyte, endothelial, and platelet origin were identified.

**Results**

Between June 2022 and June 2023, 26 CO-intoxicated patients were enrolled, showing a median age of 57 (43–71) years, with 13 (50%) men and 7 (27%) active smokers. All poisonings were accidental, and median intoxication time was 12 (interquartile range IQR:2.8–21) hours, with 13 cases classified as mild-moderate, the other 13 as severe. Median carboxyhemoglobin (COHb) level measured on arterial blood gases (ABG) was 24% (IQR: 16.6–27.4). Twenty-five out of the 26 patients were treated with HBOT, while one patient could not undergo treatment due to an unfavorable risk–benefit ratio. Our data showed a significant increase in platelet-derived MVs at T0 in CO-intoxicated patients compared to controls (6107 (IQR: 1548–9183) vs 2391 (IQR: 1739–3261) MVs/μL, Mann–Whitney test p = 0.04), but no differences in MV subpopulations were found after HBOT. Endothelial and platelet-derived MV levels at T0 positively correlated with intoxication time (Test for correlation coefficient p = 0.0028), while COHb showed a positive relation with MVs of platelet origin only (Test for correlation coefficient p = 0.0021). These results might reflect CO-induced hypoxic damage and platelet activation. Lastly, the only patient who did not undergo HBOT after CO intoxication developed DNS at follow-up.

**Conclusions**

CO intoxication seems to induce an increase in platelet-derived MVs, while HBOT might not have an influence on MV levels. However, further research and validation studies with an adequate sample size are necessary to confirm our results and potentially translate these findings into clinical practice.

### A16 Early hyperbaric oxygen therapy for the management of gas embolism after removal of central venous catheter

#### A. Molinaro^1^, L.M. Titherington^2^, A. Franci^1^, G. Fulceri^1^, F. Socci^1^, M. Bonizzoli^1^

##### ^1^Intensive Care Unit and Regional, ECMO Referral Centre; Center for Hyperbaric Medicine, Careggi University Hospital, Firenze, Italy; ^2^Section of Anesthesiology and Intensive Care, Department of Anesthesia and CriticalCare, Careggi University Hospital, Firenze, Italy

###### **Correspondence:** A. Molinaro

*Journal of Anesthesia, Analgesia and Critical Care 2024*, **4(1):**A16

**Background**

Cerebral air embolism is a rare, life-threatening complication of insertion and removal of intravenous catheters. Prompt recognition of signs and symptoms is crucial in management. Hyperbaric oxygen therapy (HBO) can be a viable treatment strategy in patients that present with neurological complications[1].

**Case presentation**

After obtaining informed consent we report the following case report. A 16 yo female patient underwent surgery in Careggi Hospital for resection of a hepatocellular carcinoma. A central venous catheter (CVC) was placed in the right internal jugular (RIJ) vein pre-operatively under ultrasound guidance. There were no postoperative complications and she was to be discharged 7 days post-surgery. Prior to discharge, the CVC was removed. After removal of the device, the patient immediately complained of pain at the site of insertion and intense generalised asthenia. The patient subsequently lost consciousness and had a tonic–clonic seizure. She was stabilised and underwent a direct head CT, which was negative for acute cerebral events. The patient was transferred to the Stroke Unit and underwent an angio-CT of the neck and intracranial vessels which showed multiple air bubbles in the right internal jugular vein and the right brachiocephalic vein. A pulmonary CT showed subcutaneous air bubbles adjacent to the distal tract of the RIJ. On regaining consciousness, the patient complained of hyposthenia on the right arm and hypoesthesia of the left arm. She underwent two sessions of HBO Tab. 6 U.S. Navy (Fig. 1) with full regression of neurological symptoms during the first session of HBO, started four hours after the onset of symptoms.

**Discussion and conclusion**

HBO is the primary treatment for decompression sickness and arterial gas embolism from SCUBA diving [2]. HBO can reduce the size of the air bubble by displacing nitrogen with the high concentrations of oxygen. The hyperoxia produced can also reduce the effects of the local hypoxia caused by the air embolism. Timing is fundamental for successful treatment as a drastic reduction in efficacy of treatment has been shown after six hours [3]. Our case demonstrates the effective use of HBO for treatment of a paradoxical gas embolism in a dramatic case associated with the removal of an intravascular device. However, the importance of timing cannot be understated, prompt recognition of this rare condition is crucial in reducing neurological long-term sequelae.

Informed consent was obtained.

**References**

1. Blanc, P., Boussuges, A., Henriette, K., Sainty, J. M., & Deleflie, M. (n.d.). Iatrogenic cerebral air embolism: importance of an early hyperbaric oxygenation. Intensive Care Medicine, 2002 May;28(5):559–63. 10.1007/s00134-002-1255-0. Epub 2002 Mar 21.
2. Leitch, D., & Green, R. (n.d.). Pulmonary barotrauma in divers and the treatment of cerebral arterial gas embolism. Aviat Space Environ Med., 1986;57(10 Pt 1):931.
3. Moon, R. (n.d.). Hyperbaric oxygen treatment for air or gas embolism. Undersea Hyperb Med, 2014;41(2):159.

**Fig. 1:**
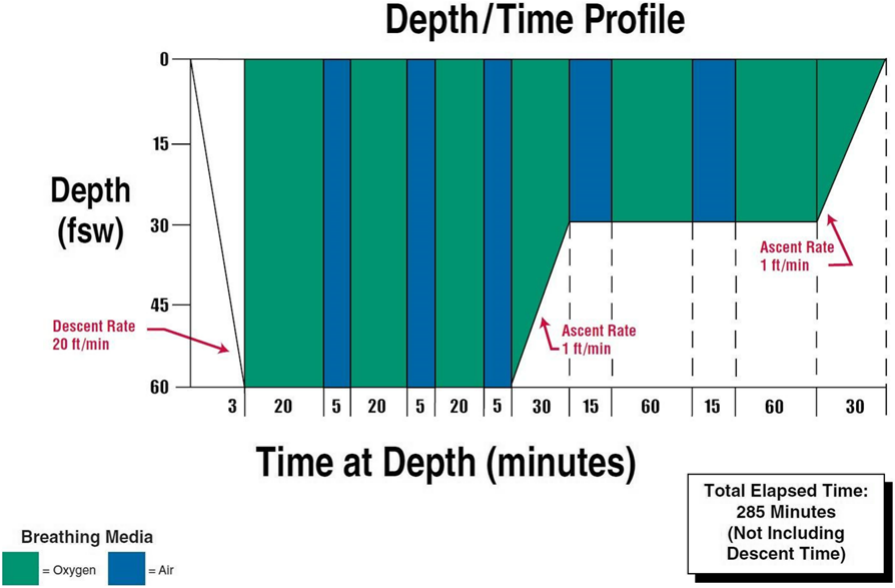
** (abstract A16).** US Navy Treatment Table 6 (from wikimedia commons)

## Critical care and out-of-hospital emergency medicine

### A17 One year description of ischemic stroke patients transported by EMS at the emergency department of Niguarda hospital

#### F. Bernasconi^2^, M. Favarato^1^, M. Generali^2^, A. Coppo^3^, I. Serrai^2^, A. Cervo^2^, A. Chieregato^2^

##### ^1^Università Milano Bicocca, Milano, Italy; ^2^Grande Ospedale Metropolitano Niguarda, Milano, Italy; ^3^Agenzia Regionale Emergenza Urgenza, Milano, Italy

###### **Correspondence:** F. Bernasconi

*Journal of Anesthesia, Analgesia and Critical Care 2024*, **4(1):**A17

**Introduction**

Identification of a stroke patient in the field allows them to be sent directly to the most appropriate hospital, minimizing diagnostic and treatment times. Moreover, if a large vessel occlusion (LVO) is suspected, it could also be transported directly to centers equipped with interventional neuroradiology.

This descriptive analysis on the population with suspected stroke transported by local EMS and to show the frequency and clinical characteristics of the population with intracranial large vessel occlusion.

**Materials and methods**

This is a single center analysis, derived from a multicenter study active in the Milan Metropolitan area (AREU_AIPO registered protocol). We reviewed 1 year (1 March 2023 – 28 February 2024) of patients with a “stroke code” transported by 118 to the Niguarda Hospital in Milan. A stroke code is a patient with positive Cincinnati prehospital stroke scale (CPSS), symptoms onset within 24 h, not in coma and with modified Rankin scale (mRS) less than or equal to 3. We analyzed baseline characteristics: hospital diagnosis, occlusion site, and National Institutes of health Stroke Scale (NIHSS) at triage. We observed the distribution frequency of NIHSS in the population with and without LVO, and finally compared the frequency of LVO in stroke patients (NIHSS > 5) from that with minor stroke (NIHSS 0–5).

Due to the retrospective and observational nature of this study the need for informed consent was waived.

**Results**

Baseline characteristics are resumed in Table 1. 514 were assigned a stroke code, 308 were actual strokes at hospital diagnosis. LVO was found in 88 patients (37% of ischemic strokes), most of them situated in the anterior circulation (respectively 88 vs 4 patients). Distribution frequencies of NIHSS between two different groups (LVO vs non-LVO) are shown in graph 1 and the comparison of frequency of LVO in graph 2. A statistically significant difference (p < 0.0001, RR 3.01) between minor stroke vs stroke patients was found.

**Conclusions**

Triaging patients with suspected ischemic stroke based on clinical findings may be reasonable, cause patients with higher NIHSS have a 3-fold relative risk of having LVO.

However, even patients with few symptoms may have LVO. Therefore, it is important that all patients with neurological deficit are referred to stroke ready hospitals: with level I – II diagnostics, and efficient secondary transport systems should the need for a more experienced center arise.

**References**

1. Powers WJ, Rabinstein A, Ackerson T, et al. 2019 Update to the 2018 Guidelines for the Early Management of Acute Ischemic Stroke. Stroke. 2019;50:e344–e418
2. Agostoni E, Carolei A, Micieli G, et al. The organisation of the acute ischemic stroke management: key notes of the Italian Neurological Society and of the Italian Stroke Organization. Neurol Sci. 2018 Mar;39(3):415–422.

**Fig. 1:**
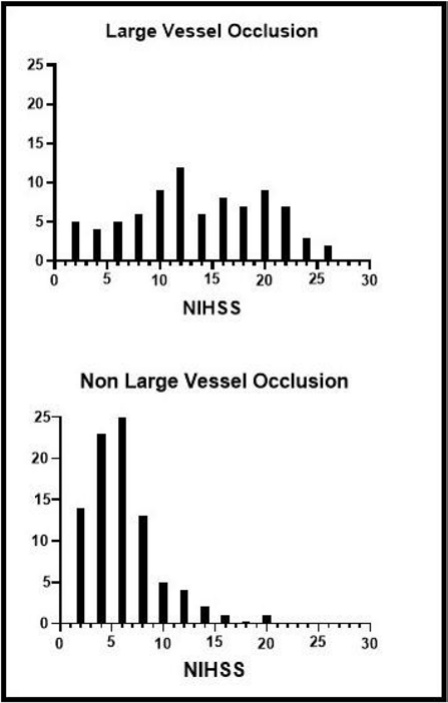
** (abstract A17).** NIHSS distribution in patients with and without large vessel occlusion

**Fig. 2:**
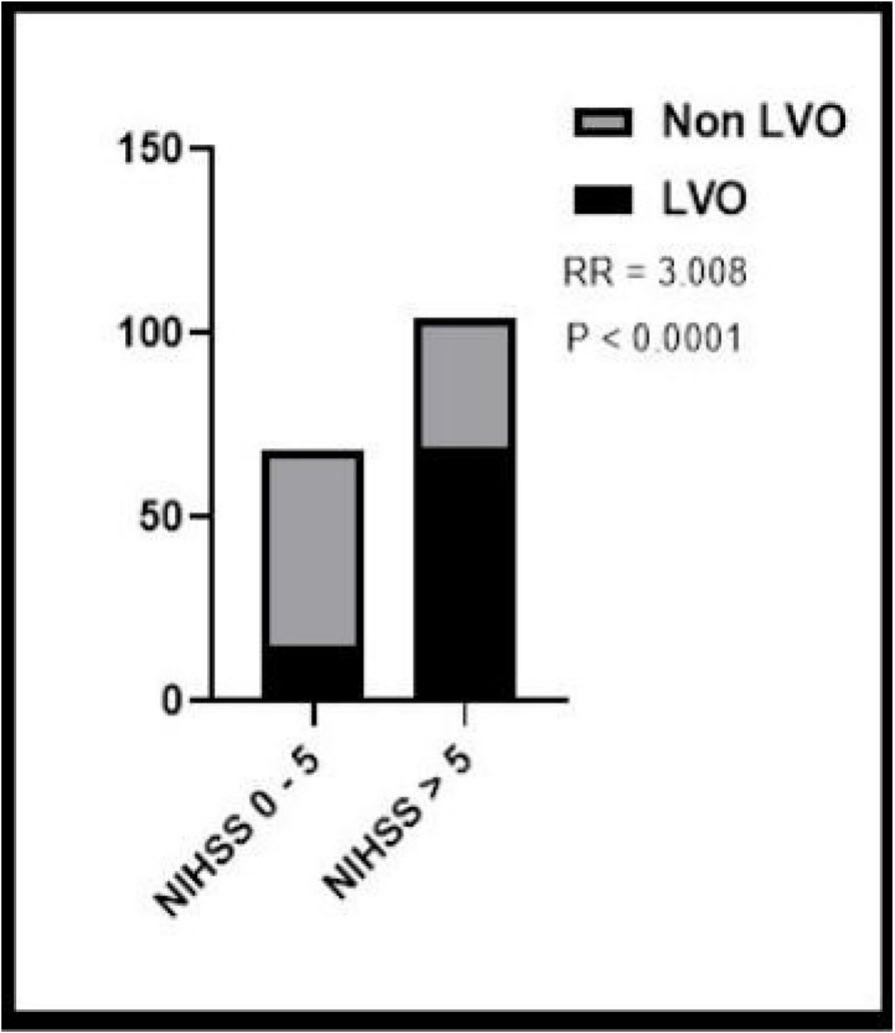
** (abstract A17).** Comparison of large vessel occlusion (LVO) frequency between two different populations, minor stroke (NIHSS 0-5) and stroke (NIHSS >5). RR = Relative Risk

**Table 1:** **(abstract A17).** Baseline population characteristics. *of ischemic stroke

**Suspected stroke, n°**	514
**- Ischemic stroke, n° (%)**	- 239 (47)
**- Hemorragic stroke, n° (%)**	- 69 (13)
**- Non stroke, n° (%)**	- 206 (40)
**Age, yrs, average (± SD)**	72 ± 16
**Female, n° (%)**	221 (43)
**AngioTC, n° (%)**	215 (90*)
**NIHSS, average (± SD)**	9 ± 6
**LVO, n° (%)**	88 (37*)
**- Anterior (ICA, M1, M2, A1)**	84 (95)
**- Posterior**	4 (5)

## Critical care and intra-hospital emergency medicine

### A18 Non-pharmacological therapies for the administration of anxiety and care in pediatric emergency room

#### N. Tiberii^1^, M.L. Simonetti^2^

##### ^1^Filadelfia Cooperativa sociale Onlus, Teramo, Italy; ^2^AST Ascoli Piceno, Ascoli Piceno, Italy

###### **Correspondence:** N. Tiberii

*Journal of Anesthesia, Analgesia and Critical Care 2024*, **4(1):**A18

**Background**

An analysis of the epidemiological data was carried out in which an annual increase in accesses to the Emergency Room (ER) of Ascoli Piceno (AP) was found. ER visits for the years 2020/2021/2022/2023 were taken into consideration; in the year 2020, 23,550 accesses were recorded of which 10.45% were pediatric, in the year 2021 were recorded 24,744 which 10.92% were pediatric, for the year 2022 were recorded 27,389 which 12.46% pediatric and in 2023 they were 30,125 which 11.64% pediatric. The color code assigned to pediatric patients was taken into consideration, the prevalence in all years is given by the green code. The main pathology found in 2020 and 2021 is trauma (> 34%) whereas in 2022 and 2023 the main pathology is fever (> 27%).

**Materials and methods**

The population from 0 to 16 years old was taken into consideration, the age at which one can still be taken care of by the Free Choice Pediatrician in the province of AP from 2020 to 2023. From the analysis of the epidemiological data taken into consideration the pediatric accesses are increasing, for a total of 12,084 pediatric accesses over the 4 years. After analyzing the preliminary data, an anonymous questionnaire consisting of 14 items was created and distributed via social networks to parents in the province of AP; the same aimed at investigating the motivation of the parent who decides to access the ER, and how the team welcomed and acted towards the child and the parent.

**Results**

The collection was carried out from February to December 2023 with a total of 270 correctly completed questionnaires. From the analysis we can observe that 35.95% of parents accessed their child more than once, 71.5% by autonomous decision and only 13.4% through the Pediatrician, this is because 37, 1% of parents still do not have a good relationship of trust with it, 57.2% because the Pediatrician clinic was closed. As regards the behavior of the team, for 36.96% of parents it did not address itself and did not act in the best way with the pediatric patient. 49.5% of parents believe that non-pharmacological therapy techniques should always be used as they lower the fears of the children and the parents themselves and this allows the team to work more peacefully.

**Conclusions**

The results of this investigation highlighted that one of the most significant factors is to improve the environment by inserting distractions for children that can deconcentrate their attention, for example using a room with colored walls, games and other sources of distraction could help relate positively to the team. A relevant result also emerged regarding greater preparation on the part of the team regarding the pediatric patient, as a negative experience within the ER can create trauma in the child for future experiences.

Informed consent was obtained.

### A19 Early Advanced Life Support (ALS), Lucas, and aortic counterpulsation in pseudopea: a tale of effective resuscitative practice

#### C. Marconi^1^, E. Cogi^2^, M. Magri^2^, P. Gnesin^2^

##### ^1^Universita' Degli Studi Di Brescia, Brescia, Italy; ^2^Asst Franciacorta, Chiari, Brescia, Italy; ^3^Asst Franciacorta, CHIARI, Brescia, Italy; ^4^Asst Franciacorta, Chiari, Brescia, Italy

###### **Correspondence:** C. Marconi

*Journal of Anesthesia, Analgesia and Critical Care 2024*, **4(1):**A19

A 77-year-old man with diabetes, hypertension, active smoker (20/day) with a past medical history of STEMI (PTCA + stent in 2009) and residual multiple stenoses (100% on AL, 99% on IVP), was admitted to the Cardiology department for elective coronary angiography (new PTCA + Stent on proximal IVA and CFX). A few hours after the procedure, he complained of chest pain with rapid clinical deterioration: onset of gasping, marked hypotension, and evolving ST- elevation on ECG rapidly progressing to ACC. Pseudo-PEA presentation rhythm was initially diagnosed through arterial monitoring already in place for angiographic procedures. Immediate ALS initiation ensued: orotracheal intubation, IV adrenaline (total 4 mg), and placement of a mechanical chest compressor Lucas. Subsequently, VF occurred, and 5 shocks at 200 J were delivered, along with IV amiodarone and lidocaine. After 20 min, pseudo-PEA persisted. In agreement with the hemodynamic team, a new angiographic procedure during ACC was opted for (Lucas still active and mechanical artificial ventilation). Angiography revealed acute intra- stent thrombosis, leading to re-PTCA with flow restoration, placement of an aortic counterpulsator and continuous adrenaline infusion (0,2 mcg/kg/min), resulting in ROSC approximately 60 min after initiation of maneuvers, with BP 110/40 mmHg and HR 95 bpm. By the second day in the Intensive Care Unit, the patient began improving until removal of the counterpulsator (EF 45%) and cessation of catecholamines. Alert and cooperative, he was extubated. On the third day of admission, with stable vital signs and no motor or sensory deficits in all four limbs (GCS 15), he was transferred to the intermediate care unit.

Literature indicates that ultrasound has enabled the classification of PEA into two groups: true PEA and pseudo-PEA (pPEA), a term describing pulselessness with minimal cardiac activity. Some reports have demonstrated that pPEA has a better prognosis regarding ROSC and higher survival rates [1, 2]. In our clinical case, pPEA was diagnosed via arterial monitoring and influenced resuscitative choices toward attempting to remove a possible primary cause of ACC (acute thrombosis), despite the now extended time frame. The absence of neurological deficits is the result of immediate application of effective ALS combined with a mechanical chest compressor (Lucas). This offers an intriguing insight for future cases of ACC with pPEA, both in intra and extra-hospital settings [3]: immediate CPR with an automatic compressor and the prospect of a removable cause might encourage more aggressive resuscitation with continuation of resuscitative efforts beyond a conventional time window.

Informed consent to publication was obtained.

**References**

1. Wu C, Zheng Z, Jiang L, et al. The predictive value of bedside ultrasound to restore spontaneous circulation in patients with pulseless electrical activity: a systematic review and meta-analysis. 2018;13(1): e0191636
2. Devia Jaramillo et al. Rhythms and prognosis of patients with cardiac arrest, emphasis on pseudo- pulseless electrical activity: another reason to use ultrasound in emergency rooms in Colombia (2020) 13:62
3. Gaspari R, Weekes A, et al. Emergency department point-of-care ultrasound in out-of-hospital and in- ED cardiac arrest. Resuscitation. 2016;109:33–9

### A20 Is lidocaine useful in extubation in intensive care?

#### S. Geppert^1^, S. Tantillo^2^, I. Ottaviani^2^, M. Guarnera^2^, F. Benvenuti^2^, M. Losito^1^, F. Franzoi^1^, F. Talarico^2^, N. Cilloni^2^

##### ^1^Dipartimento di Medicina e Chirurgia, Università di Bologna, Bologna, Italy; ^2^AUSL di Bologna, Ospedale Maggiore, UOC Terapia Intensiva e HUB maxiemergenze, Bologna, Italy

###### **Correspondence:** S. Tantillo

*Journal of Anesthesia, Analgesia and Critical Care 2024*, **4(1):**A20

**Background**

The administration of intravenous lidocaine to prevent post-extubation complications in postoperative patients is well described in literature1,2. However, this role of lidocaine in ICU patients has not been investigated. We report a case of successful extubation after lidocaine administration in a patient admitted to the ICU for respiratory failure.

**Clinical case**

A 50 year woman, heavy smoker, with a history of COPD, presented to the emergency department for severe cough and dyspnea. A CT scan was performed, revealing interstitial pneumonia.

A NIV trial was attempted, but was interrupted early for worsening of the symptoms.

The patient was then admitted to the ICU, intubated and placed on mechanical ventilation. On day one the patient developed a severe bronchospasm, requiring deep sedation and a multimodal bronchodilator therapy. On day five post admission, after improvement of respiratory symptoms, sedation was discontinued for a spontaneous breathing trial. Several episodes of coughing were registered and a lidocaine iv bolus was successfully administered. It was then decided to start lidocaine i.v. infusion before extubation attempt. After the i.v. bolus of lidocaine 1,5 mg/kg and the infusion of 1 mg/kg/h for 3 h, no more episodes of coughing were registered and the patient was successfully extubated at the first attempt. Informed consent was obtained for data processing for clinical research purposes.

**Conclusion**

The role of Lidocaine in ICU has been scarcely investigated. The use of i.v. lidocaine perioperatively decreased airway complications, including coughing and sore throat, and there was no associated increased risk of harm.1 The mechanism underlying the inhibitory effect of lidocaine on coughing is not completely understood. Many mechanisms have been proposed, like the suppression of airway sensory C fibres, responsible for cough and dyspnoea.3 This case illustrates the potential use of i.v. lidocaine in ICU, especially in patients with a bronchial hyperreactivity to prevent post-extubation complications. More studies are needed in order to investigate the efficacy of i.v. lidocaine in reducing respiratory complications after extubation in the ICU.

**References**

1. Yang SS, Wang NN, Postonogova T et al. Intravenous lidocaine to prevent postoperative airway complications in adults: a systematic review and meta-analysis. Br J Anaesth. 2020 Mar;124(3):314–323. 10.1016/j.bja.2019.11.033.
2. Lin J, Wang W-D, Yang Q-Y et al. Effect of intravenous and topical laryngeal lidocaine on sore throat after extubation: a prospective randomized controlled study. Eur Rev Med Pharmacol Sci. 2024 Mar;28(6):2493–2500. 10.26355/eurrev_202403_35756.
3. Burki NK, Lee LY. Blockade of airway sensory nerves and dyspnea in humans. Pulm Pharmacol Ther. 2010 Aug;23(4):279–82. 10.1016/j.pupt.2010.02.002. Epub 2010 Feb 25.

### A21 Renal outcomes in the early use of vasopressin in the treatment of septic shock: design of a single-center prospective randomized controlled multidisciplinary clinical trial

#### L. Giuntoli^1^, M. Guarnera^1^, E. Gamberini^1^, L. Gamberini^2^, S. Ciardo^2^, A. Domanico^3^, E. Accogli^3^, V. Dalmastri^4^, G. Rizzoli^5^, A. Innocenti^6^, N. Cilloni^1^

##### ^1^Intensive Care Unit, Maggiore Hospital, AUSL Bologna, Bologna, Italy; ^2^Division of Anesthesia, Intensive Care and Prehospital Emergency, Ospedale Maggiore Carlo Alberto Pizzardi,, Bologna, Italy; ^3^Internal Medicine Unit A, Azienda USL-Maggiore Hospital, Bologna, Italy; ^4^Department of Nephrology and Dialysis, Maggiore Hospital,, Bologna, Italy; ^5^Department of Anaesthesia and Intensive Care, University of Ferrara, Ferrara, Italy; ^6^Alma Mater Studiorum, Dipartimento Di Scienze Mediche E Chirurgiche, Anesthesia and Intensive Care Medicine, Policlin, Bologna, Italy

###### **Correspondence:** M. Guarnera

*Journal of Anesthesia, Analgesia and Critical Care 2024*, **4(1):**A21

I**ntroduction**

As recommended by the guidelines, norepinephrine is the vasoactive drug of first choice in the tratment of septic shock, to which it is recommended to add vasopressin in continuous infusion if adeguate mean pressure values are not achieved.1 There are several studies2,3 in the literature that have evaluated the effects of vasopressin on the complications of disease, in particular regarding renal function and the possible need for dialysis support, with inconclusive results. The aim of the present study is to verify whether the earlier association of vasopressin and norepinephrine determines variations in terms of use of renal function replacement techniques in the first 7 days of hospitalization in the Intensive Care Unit (ICU), compared to patients in whom vasopressin is added only when norepinephrine reaches a higher dosage. Secondary objectives of the study are: duration of shock, differences in terms of renal function such as KDIGO class, changes in renal perfusion assessed by ultrasound evaluation according to CEUS method, duration of dialysis therapy, onset of complications associated with the use of vasopressors, length of ICU stay, 28- and 90-day mortality.

**Study design**

Patients will be divided into two groups, randomized with a 1:1 ratio through a computer-generated scheme; Group A: addition of vasopressin (with standard dosage of 0.03 IU/min), when the dosage of norepinephrine is higher than 0.25 mcg/kg/min, Group B: addition of vasopressin (with standard dosage of 0.03 IU/min) when the norepinephrine dosage is higher than 0.5 mcg/kg/min. The expected sample for the study is 264 patients. The two groups will be compared for general characteristics of the population with basic tests appropriate for the type of comparison to be made. Continuous variables will be expressed as means and standard deviations if characterized by a normal distribution according to the Shapiro–Wilk test, and the t-test for independent groups will be applied for their comparison. Ordinal and continuous variables not normally distributed will be expressed as medians and interquartile ranges and compared with the Mann–Whitney U test. Categorical variables will be expressed as numbers and percentages and compared using the Chi square test. The tests carried out will be two-tailed, a p-value less than 0.05 will be considered significant.

**Conclusions**

The clinical study has received approval from the national ethical committee for drug testing and will take place in the ICU of the Maggiore Hospital in Bologna, lasting 2 years, with collaboration of nephrologists and expert ultrasound specialists.

**References**

1. Ukor IF, Walley KR. Vasopressin in Vasodilatory Shock. Crit Care Clin. 2019 Apr;35(2):247–261.10.1016/j.ccc.2018.11.004.
2. Gordon CA, Mason AJ, Thirunavukkarasu N et al. Effect of Early Vasopressin vs Norepinephrine on Kidney Failure in Patients With Septic Shock: The VANISH Randomized Clinical Trial. JAMA. 2016 Aug 2;316(5):509–18. 10.1001/jama.2016.10485.
3. Russel JA, Walley KR, Singer J et al. Vasopressin versus norepinephrine infusion in patients with septic shock. N Engl J Med. 2008 Feb 28;358(9):877–87. 10.1056/NEJMoa067373

### A22 Critical care outreach at “Spedali Civili Di Brescia” hospital: report of first year

#### M. Filippini ^1^, A. Capone^2^, N. Latronico^1,2^

##### ^1^Department of Emergency and Critical Care, Brescia Spedali Civili University Hospital, Italy, Brescia, Italy; ^2^University of Brescia, Italy, Brescia, Italy

###### **Correspondence:** A. Capone

*Journal of Anesthesia, Analgesia and Critical Care 2024*, **4(1):**A22

**Background**

Critical Care Outreach (CCO) is a more recent development of traditional Rapid Response Systems which, apart from clinical assessment, may offer a more holistic, supportive approach for critical patients outside Intensive Care Units (ICU) [1,2]. In February 2023, a CCO program started at Brescia Spedali Civili University Hospital: this is the report of its activity.

**Materials and methods**

The report describes CCO activity in terms of number of examined patients (single examinations and re-examinations, total and daily), critical patients managed outside the ICU or transferred to ICU (a patient was defined in a critical condition if he/she needed continuous cardiocirculatory and respiratory monitoring, ventilatory support, catecholamines, or continuous renal replacement therapy), mean weekly number of emergency rescue interventions per 100 occupied beds before CCO activation (from August the 13th to September the 30th 2022) and after CCO activation (from January the 1st to March the 19th 2024); before-after difference was evaluated with Student’s t-test with a level of statistical significance set at p < 0.05.

**Results**

From February the 1st 2023 to January the 31st 2024, CCO worked for 250 days (from Monday to Friday, except for holiday days, from 8:00 to 16:00: 2,000 h of work) in a hospital with about 900 beds (min 753, max 958). CCO examined 702 patients (2.8 new patients/day, 62% males, mean age: 66 years) and performed 2,206 total examinations and re-examinations (8.8 examinations/day). Of 406 patients in critical condition (58%), 75 (11%) were transferred to the ICU and 331 (47%) were managed outside the ICU with collaboration between the CCO and the general ward teams (Fig. 1). The mean weekly rate of emergency rescue interventions per 100 occupied beds was 1.28 in the pre-CCO period and 0.45 after (p < 0.01) (Table 1).

**Conclusions**

CCO took charge of a great part of the Intensive Care work and made it possible to treat many critical patients outside the ICU; furthermore, this activity strongly reduced the number of emergency rescue interventions.

Informed consent was obtained.

**References**

1. Trenchard-Turner, N, Desai, N, & Metaxa, V. Critical care outreach teams: a service without walls. Intensive Care Medicine. 2023; 49(5), 572–574.
2. National Outreach Forum Quality and operational standards for critical care outreach services. 2020

**Fig. 1:**
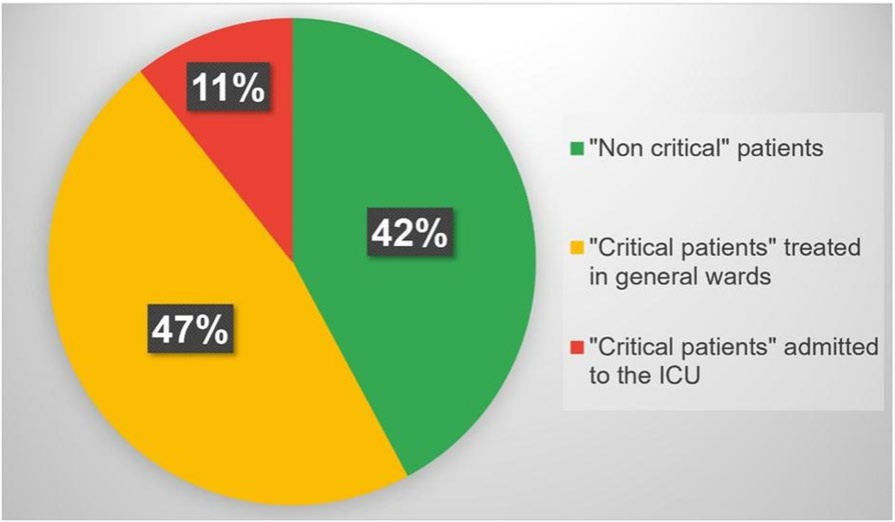
** (abstract A22).** Critical patients examined by CCO managed outside the ICU and admitted to the ICU

**Table 1:** **(abstract A22).** Weekly emergency rescue interventions before and after CCO activation

	Before CCO activation	After CCO activation	*p*
Weekly emergency rescue interventions, mean (SD)	9 (3.87)	4 (2)	< 0.01
Occupied beds (mean number during study periods)	707	840	-
Number of weekly interventions per 100 occupied beds, mean (SD)	1.28 (0.56)	0.45 (0.24)	< 0.01

*SD* Standard Deviation

### A23 Newly developed negative T waves non-ischemic in the ECG during non-cardiac surgery

#### R. Caramia^1^, M. Arces^2^, P. Palazzo^3^, M. Sacco^4^, E. Indolfi^5^, R. Flora^6^, P. Fedele^7^, C. Galizia^8^, A. Angrisano^9^, F. Mangini^10^, S. Pungente^11^, M.G. di Bari^12^, C. Guarino^13^

##### ^1^Anesthesia, Resuscitation and Pain Therapy Unit, D. Camberlingo Hospital,, Francavilla Fontana, Italy; ^2^Anesthesia, Resuscitation and Pain Therapy Unit, D. Camberlingo Hospital,, Francavilla Fontana, Italy; ^3^General Surgery Unit, D. Camberlingo Hospital, Francavilla Fontana, Italy; ^4^General Surgery Unit, D. Camberlingo Hospital, Francavilla Fontana, Italy; ^5^ICU/Cardiology Unit, D. Camberlingo Hospital, Francavilla Fontana, Italy; ^6^ICU/Cardiology Unit, D. Camberlingo Hospital, Francavilla Fontana, Italy; ^7^Anesthesia, Resuscitation and Pain Therapy Unit, D. Camberlingo Hospital, Francavilla Fontana, Italy; ^8^Anesthesia, Resuscitation and Pain Therapy Unit, D. Camberlingo Hospital, Francavilla Fontana, Italy; ^9^General Surgery Unit, D. Camberlingo Hospital, Francavilla Fontana, Italy; ^10^Department of Cardiology, General Regional Hospital F. Miulli, Acquaviva delle Fonti, Italy; ^11^General Surgery Unit, D. Camberlingo Hospital, Francavilla Fontana, Italy; ^12^Anesthesia, Resuscitation and Pain Therapy Unit, D. Camberlingo Hospital, Francavilla Fontana, Italy; ^13^Anesthesia, Resuscitation and Pain Therapy Unit, D. Camberlingo Hospital,, Francavilla Fontana, Italy

###### **Correspondence:** R. Caramia

*Journal of Anesthesia, Analgesia and Critical Care 2024*, **4(1):**A23

**Background**

Every year around the world approximately 300 million adult patients undergo non-cardiac surgery. Approximately 3% of these subjects suffer major cardiac complications.

The presence of T-wave inversion on the ECG can occur in various conditions, some even serious. This paper describes the case of a patient who experienced the appearance of negative T waves during non-cardiac surgery, without other particular signs or symptoms.

**Case report**

A 36-year-old male patient underwent laparoscopic cholecystectomy. He was severely obese (BMI 43.7) with unrecognized high blood pressure and a heavy smoker. He never had a cardiac examination and his preoperative ECG was normal (Fig. 1). In the operating room the patient had severe arterial hypertension (205/100 mmHg) and a saturation of 89%. Hypertension was treated with urapidil and clonidine. Before the induction of anesthesia, the blood pressure had stabilized at 140/80. There was no ECG abnormality on the monitor. Anesthesia was induced with propofol, fentanyl, rocuronium and was maintained with sevoflurane/oxygen, and rocuronium.

During the operation, the patient had blood pressure values around 100/70 mmHg and at a certain point negative T waves appeared on the monitor. A blood sample was taken to measure cardiac enzymes and the consultant cardiologist was called. The patient presented a single hypotension value of 90/50 mmHg, treated with fluid therapy.

Once the surgery was finished, the patient was given a 12-lead ECG which confirmed the T- waves inversion in V3-V6 leads (Fig. 2). The results of the blood tests revealed normal values. We proceeded to wake up the patient who showed no cardiological symptoms with normal hemodynamic parameters. The patient was monitored with ECGs and an echocardiogram which showed dilatation of the left ventricle with wall hypertrophy. No abnormalities of ventricular kinetics. Negative T-waves were present in the ECGs performed after 6 h (Fig. 3), decreasing after 12 h (Fig. 4) and finally disappearing the next day (Fig. 5). The patient presented no further problems and was discharged after 5 days.

We hypothesize that the appearance of the inversion of the T waves was due to a 'relative hypotension' during the operation as the patient went from a condition of severe hypertension to values at the low limits of normal, creating a 'discrepancy' of coronary flow without apparent damage.

**Conclusion**

The appearance of T-wave inversion on the ECG can occur in various conditions even dangerous. We describe a case of a patient who experienced the appearance of T wave inversion probably due to relative intraoperative hypotension.

The patient gave informed consent for publication.

**Fig. 1:**
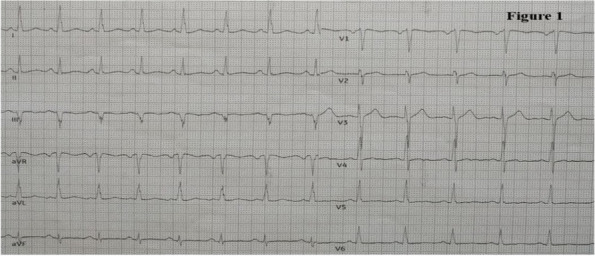
** (abstract A23).** See text for description

**Fig. 2:**
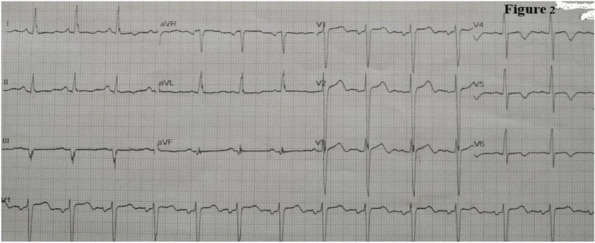
** (abstract A23).** See text for description

**Fig. 3:**
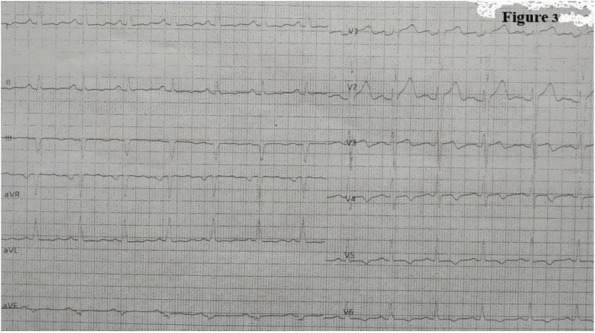
** (abstract A23).** See text for description

**Fig. 4:**
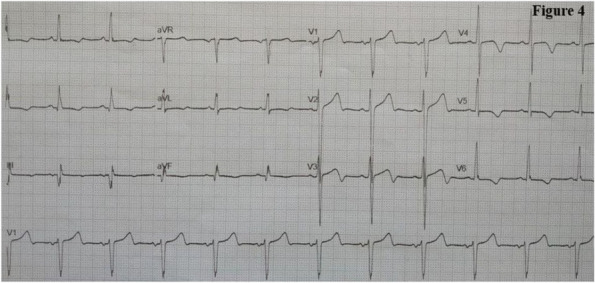
** (abstract A23).** See text for description

**Fig. 5:**
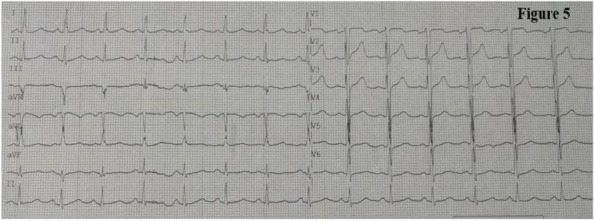
** (abstract A23).** See text for description

### A24 Atypical presentation of giant hiatal Hernia with cardio-pulmonary failure in the emergency department

#### A. Antoniucci^1^, L. Graziosi^2^, E. De Robertis^1^

##### ^1^University of Perugia, Department of Anesthesia and Intensive Care Medicine, Perugia, Italy; ^2^Hospital Santa Maria della Misericordia, General surgery, Perugia, Italy

###### **Correspondence:** A. Antoniucci

*Journal of Anesthesia, Analgesia and Critical Care 2024*, **4(1):**A24

**Background**

Dyspnoea is usually caused by respiratory or cardiovascular diseases. Only sometimes, it is due to displacement of abdominal organs into the thorax [1]. Hiatal hernias (HH) may give rise to diagnostic difficulties because the symptoms can suggest different disorders and gastrointestinal manifestations do not occur in all the patients.

**Case Report**

A 65-year-old women was admitted to the emergency department with dyspnoea, nausea and a positive SARS-CoV 2 swab. Her comorbidities were HH and gastric reflux, previous pulmonary embolism and obesity. In the emergency room she developed haemodynamic instability with fast atrial fibrillation and hypercapnic respiratory acidosis. Lung US showed confluent B-lines at lung level and marked gastric distension. Before starting non-invasive ventilation (NIV) to treat respiratory acidosis, a nasogastric tube (NGT) was placed, which drained 5L of fluid with mixed old gastric material, with partial improvement of the vital parameters. CT of the abdomen (Fig. 1) showed a voluminous transitional gastric hernia, with massive gastrectasia. Despite initial improvement with NIV, she required admission to our intensive care unit for invasive mechanical ventilation. In the absence of gastric volvulus, peritonitis or signs of ischaemia, surgery was not indicated as an emergency. After haemodynamic and respiratory stabilization, she underwent diaphragmatic hiatoplasty with Nissen fundoplication, and was successfully transferred to the ward.

**Discussion**

The initial presentation of a giant HH in this case was characterised by a predominant respiratory compromise associated with cardiovascular impairment and only mild gastrointestinal symptoms. We believe that the haemodynamic instability was secondary to caval compression caused by the hernia and dehydration. Viral respiratory illnesses during the winter season can complicate the clinical picture: in this case, Covid was not the main cause of the respiratory failure but probably only aggravated the respiratory symptoms of a massive gastric hernia, in a patient with history of HH and gastric reflux.

**Learning point**

Lung US and NGT placement before starting NIV might play a pivotal role in finding abdominal cause of respiratory failure and haemodynamic instability. Surgery is the definitive treatment for giant HH.

Informed consent was obtained.

**Reference**

1. Lesinski J. et al.; Giant hiatal hernias, Advances in Respiratory Medicine 2019; 87(1):54-62.

**Fig. 1:**
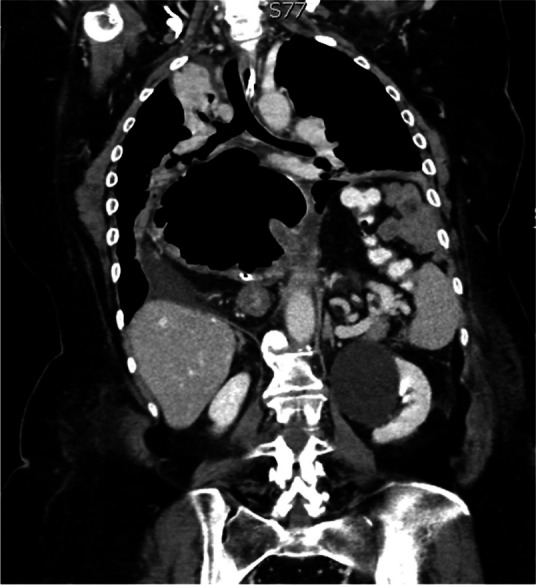
** (abstract A24).** CT scan at emergency department

### A25 An unusual intoxication: a case report of an acute liver failure caused by dimethylformamide

#### M. Albore, E. Ravera, I. Pabon, G. Carlidi, S. Marchisio, A. Della Selva

##### ASL CN2, Alba, Italy

###### **Correspondence:** M. Albore

*Journal of Anesthesia, Analgesia and Critical Care 2024*, **4(1):**A25

**Rationale**

Chronic exposure to dimethylformamide, an industrial solvent, may cause impaired liver function and may present with symptoms mimicking acute abdomen. Up to what point can the organ damage caused by chronic exposure be worsened by an acute intoxication?

We report the case of a 21-year-old male patient who presented to the emergency department (ED) with general malaise, vomiting, diarrhea, abdominal pain and ataxia. The initial assessment blood tests showed an elevation of the cytolysis and cholestasis enzymes with an increase in clotting times. The hepatological intensive care unit was therefore contacted, they suggested admitting the patient in a general intensive care unit (ICU) for monitoring of his clinical condition and serial blood testing.

In the ICU an infusion of n-acetylcysteine, vitamin K and phosphorus was immediately started. The state of consciousness was never altered. The abuse of illegal substances as well as a psychiatric evaluation to rule out a suicide attempt were performed. Urine and serum samples collected and sent to the toxicology referral center (CAV) were tested for the presence of new synthetic drugs with results negative. The ceruloplasmin dosage was found to be at the lower limits but further tests excluded Wilson disease and the sierology for hepatotropic viruses was negative as well as for autoimmune diseases.

Further anamnestic data was collected and the isolated fact of an accidental ingestion, during a work shift, of a small quantity of dimethylformamide from an unmarked bottle prompted new samples to be collected and sent to the CAV for further targeted testing for this substance. The urine sample confirmed the presence of dimethylformamide.

It is unlikely that the administration of a small quantity of dimethylformamide could have caused an acute liver failure (ALF) of this magnitude, however, it is possible, as can be seen from the literature, that the substance was absorbed over time through inhalation or skin contact in the workplace and that the oral ingestion contributed to reaching high blood levels.

In the following days there was a reduction in cytolysis enzymes and a slow normalization in clotting times [Img.1]. The patient never showed signs of encephalopathy or renal failure. Discharge to a medical ward occurred on the fifth day after admission to intensive care with restitutio ad integrum within a few days.

**Conclusions**

Acute liver failure can cause high mortality and morbidity and requires intensive monitoring in order to anticipate the possible evolution that could result in the need for transplant as a last therapeutic resource. Sometimes the cause may remain unknown, other times the etiological agent can be so rare as to warrant the search for any other plausible alternative.

Informed consent was obtained.

**Fig. 1:**
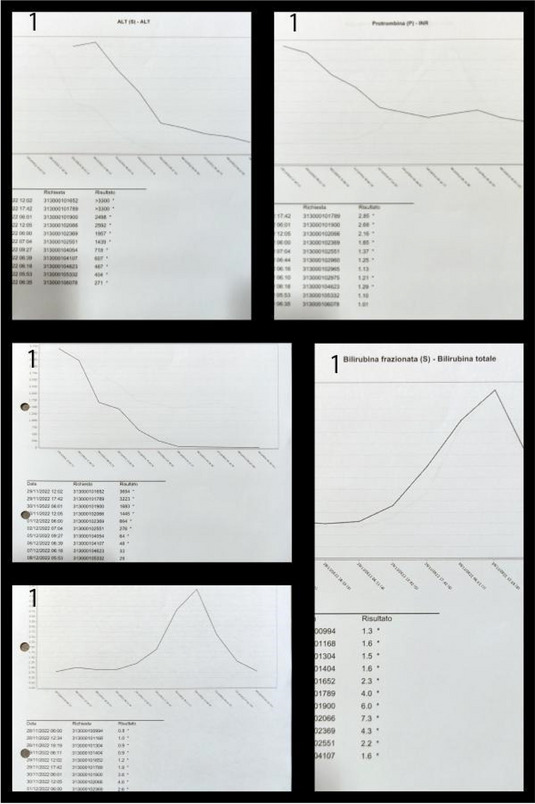
** (abstract A25).** See text for description

### A26 Ischemic spinal cord injury- monitoring and prevention in aortic surgery using NIRS (Near-Infrared Spectroscopy Monitoring): retrospective analysis

#### A. Imparato^1^, S. Notaro^1^, E. Piscitelli^1^, V. Capaldo^1^, L. Maresca^2^, A. Casalino^2^, D. Ceriello^2^, R. Auriemma^2^, A. Luongo^2^, G. Sabino^1^, A. Riccio^1^, A. Notaro^1^, B. Di Benedetto^2^, A. Corcione^1^, C. Esposito^1^

##### ^1^Intensive care and ECMO-Monaldi Hosital-AORN colli Hospital, Napoli, Italy; ^2^Vascular surgery unite-Monaldi Hospital-AORN colli Hospital

###### **Correspondence:** V. Capaldo

*Journal of Anesthesia, Analgesia and Critical Care 2024*, **4(1):**A26

**Background**

Ischemic spinal cord Injury (SCI) can occur in significant cases in acute aortic pathology or aortic surgery [1–2], like endovascular (TEVAR) and thoracoabdominal aneurysm open surgery (TAAA). Occurring during clamping and de-clamping, with hypoperfusion or local vascular injury [3] as Adamkiewicz.[4].SCI prevention strategy is CSF drainage (CSFD)not free from complications [5].

**Objective**

Evaluate NIRS as an indirect method of measurement of blood flow to the medulla during aortic surgery.

Studies suggest that the blood supply of medulla depends on a vast "collateral vascular network" [6] which also interconnects the paraspinous muscles, therefore a reduction in regional oximetry (rSO2) in this muscles corresponds to a reduction in blood flow to the medulla [7].

**Material and Methods**

After ethics committee authorization and informed consent of the participants for publication consent. A retrospective analysis was conducted from January 2019 to January 2023 and selected AAA, TAAA, TEVAR, had received NIRS and CSFD. Interventions: 132, separated in 2 groups: High(H) and Low risk (L). Group H: 63, preventive CSFD and NIRS through the INVOSTM oximeter model 5100c device (Somanetics Corp, Troy, MI, USA). Group L:69, NIRS only. The optodes were applied in the paravertebral, thoraco-lumbar (Fig. 1; Fig. 2) Statistical analysis: for parametric variables, T-student was used and statistical significance was calculated with a P < 0.01, anova and Pearson's linear regression.

**Result**

The rSO2 variations was significant with P < 0.01, rSO2 of the H group > L (26% > 7). The dependence between rSO2 (dependent variable) and ICP (independent variable) was calculated through bivariate analysis. These 2 statistical variables are dependent in that the modality of one influences the modality of the other. Finally, through Pearson's linear regression, show that there is a positive linear correlation between the two variables because r is close to 1. Median rSO2 value 70, Rapid onset of desaturation: < 4', Mean ICP 13 mmHg, Minimum PIC value 15—maximum of 23 mmHg, Mean ICP value increase was 8 mmHg, Mean PAM 83 mmHG, Mean MAP induced during ICP increases: 94 mmHg, Mean duration of postop ventilation in ICU hours: 6 h, Mortality at 28 days: 2%. NIRS highlighted a statistically significant variation in rSo2 (p < 0.01) during increases in ICP.

Group H, variations in perfusion of 10–11% corresponded to increases in intramedullary pressure of 4–5 mmHg highlighted at some critical moments. This allowed us to increase the deliquoration volume, bringing the PPC back to normal levels Group L the reduction in perfusion was 7–9% compared to baseline in this case the MAP was increased up to 95–100 mmHg. No complications were detected in group L unlike group H in which 2 patients showed the presence of blood in the CSF.

**Conclusions**

NIRS represents integrative monitoring in patients with CSFD in high-risk patients.

We suggest the use of intraoperative NIRS in all low-risk patients and postoperatively in ICU to identify early evolution towards spinal cord damage.

**References**

1. Zalewski, N. L., Rabinstein, A. A., Krecke, K. N., Brown, R. D., Jr, Wijdicks, E. F. M., Weinshenker, B. G., Kaufmann, T. J., Morris, J. M., Aksamit, A. J., Bartleson, J. D., Lanzino, G., Blessing, M. M., & Flanagan, E. P. (2019). Characteristics of Spontaneous Spinal Cord Infarction and Proposed Diagnostic Criteria. JAMA neurology, 76(1), 56–63. 10.1001/jamaneurol.2018.2734
2. Robertson, C. E., Brown, R. D., Jr, Wijdicks, E. F., & Rabinstein, A. A. (2012). Recovery after spinal cord infarcts: long-term outcome in 115 patients. Neurology, 78(2), 114–121. 10.1212/WNL.0b013e31823efc93
3. Gaul, C., Dietrich, W., & Erbguth, F. J. (2008). Neurological symptoms in aortic dissection: a challenge for neurologists. Cerebrovascular diseases (Basel, Switzerland), 26(1), 1–8. 10.1159/000135646
4. Romi, F., & Naess, H. (2016). Spinal Cord Infarction in Clinical Neurology: A Review of Characteristics and Long-Term Prognosis in Comparison to Cerebral Infarction. European neurology, 76(3–4), 95–98. 10.1159/000446700
5. Hnath, J. C., Mehta, M., Taggert, J. B., Sternbach, Y., Roddy, S. P., Kreienberg, P. B., Ozsvath, K. J., Chang, B. B., Shah, D. M., & Darling, R. C., 3rd (2008). Strategies to improve spinal cord ischemia in endovascular thoracic aortic repair: Outcomes of a prospective cerebrospinal fluid drainage protocol. Journal of vascular surgery, 48(4), 836–840. 10.1016/j.jvs.2008.05.073
6. Griepp, E. B., Di Luozzo, G., Schray, D., Stefanovic, A., Geisbüsch, S., & Griepp, R. B. (2012). The anatomy of the spinal cord collateral circulation. Annals of cardiothoracic surgery, 1(3), 350–357. 10.3978/j.issn.2225-319X.2012.09.03
7. Etz, C. D., Kari, F. A., Mueller, C. S., Silovitz, D., Brenner, R. M., Lin, H. M., & Griepp, R. B. (2011). The collateral network concept: a reassessment of the anatomy of spinal cord perfusion. The Journal of thoracic and cardiovascular surgery, 141(4), 1020–1028. 10.1016/j.jtcvs.2010.06.023

**Fig. 1:**
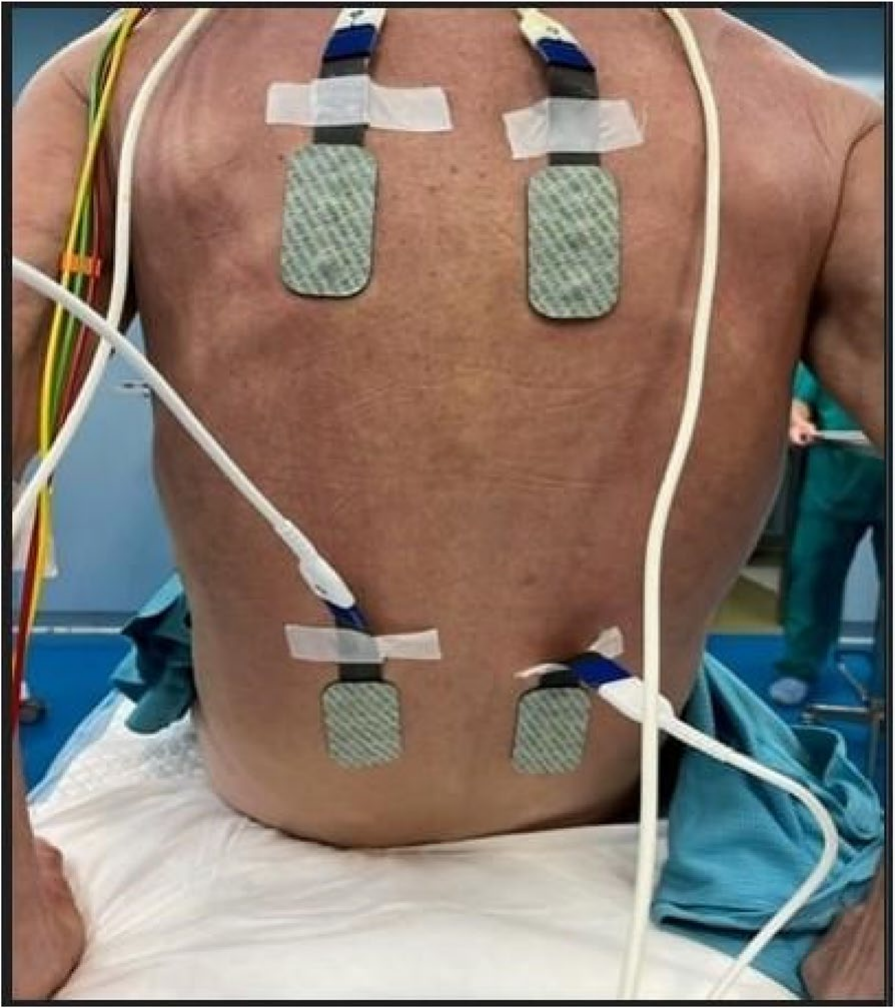
** (abstract A26).** See text for description

**Fig. 2:**
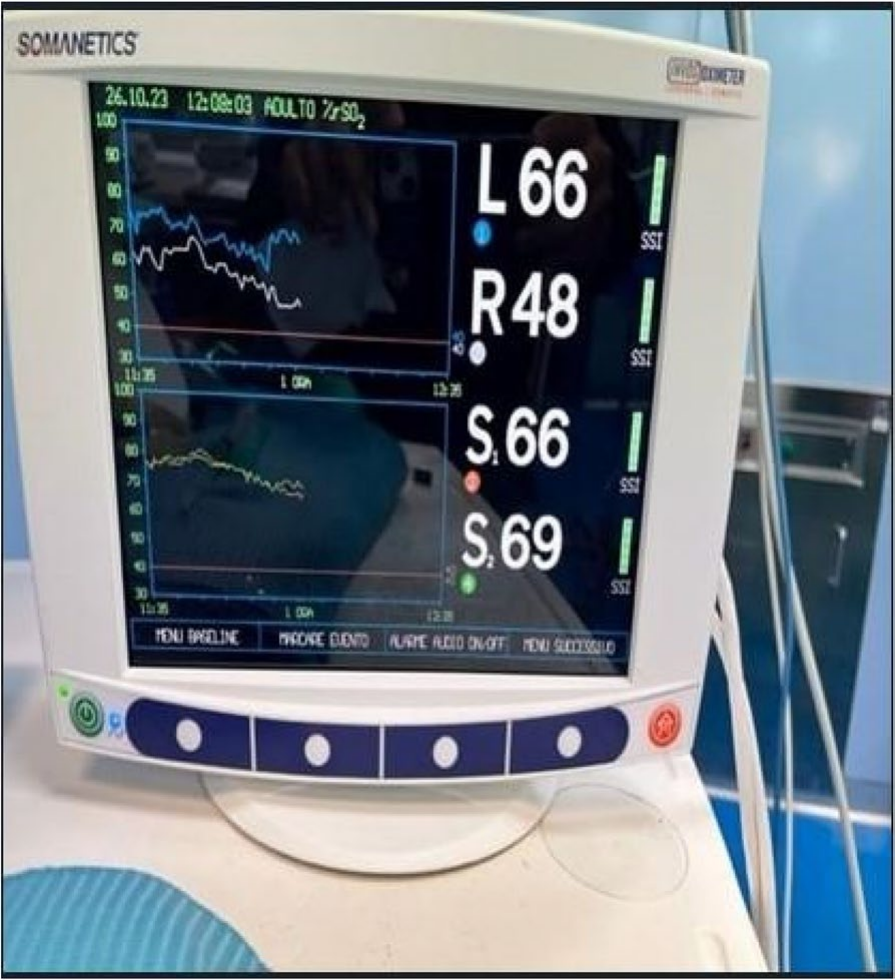
** (abstract A26).** See text for description

### A27 Evaluation of new non-invasive internal jugular venous saturation monitoring in critically ill patients with acute heart failure

#### M. Ducoli, S. Cattaneo, A. Margola

##### ASST Spedali Civili di Brescia, Department of Cardiothoracic Anesthesia and Intensive Care, Brescia, Italy

###### **Correspondence:** M. Ducoli

*Journal of Anesthesia, Analgesia and Critical Care 2024*, **4(1):**A27

**Background**

Tissue oxygenation monitoring is crucial for managing critically ill patients with heart failure. Traditionally, mixed venous oxygen saturation (SVO2) is measured via pulmonary artery catheterization (PAC), an invasive procedure with potential risks. The Mespere Venous Oximetry System allows non-invasive measurement of internal jugular venous oxygen saturation (SVJO2) using NIRS technology. This study aims to evaluate the agreement between SVJO2 measured with Mespere and SVO2 measured with PAC.

**Materials and Methods**

This prospective observational study was conducted at the Cardiothoracic Intensive Care Unit of ASST Spedali Civili di Brescia. Ten patients with a mean age of 66 years, 80% male, were enrolled. All patients were diagnosed with cardiogenic shock, with 50% post-MI and 50% post-cardiotomy (Table 1). Measurements of SvO2 from PAC and SVjO2 from Mespere sensor are taken simultaneously up to to 72 h. Data were averaged every 60 min to 1 value. A total of 478 SVO2 and 478 SVJO2 measurements were obtained. The collected data were analyzed using the Bland–Altman method to assess agreement between the two methods. Pearson's coefficient was calculated for each patient to quantify linear correlation, and overlaid line graphs were constructed to visualize temporal trends. For each patient, informed consent to partecipate in the study was obtained.

**Results**

Bland–Altman analysis showed a bias of -2.72%, with a standard deviation of 7.16%. Limits of agreement ranged from -16.75% to 11.31% (Fig. 1). The Pearson coefficient for all patients was 0.39. In 3 patients, the coefficient exceeded 0.5, indicating good correlation; in 4 patients, it ranged from 0.2 to 0.5, indicating weak correlation; in 2 patients, correlation was negligible; and in 1 patient, it was unreliable (Table 2). Due to limited range of data, distribution is not yet optimal to obtain significant correlation. Tracking of SvO2 and SvJO2 over time showed a reliable trending of measurements, especially in some patients (Figs. 2, 3, 4).

**Conclusions**

The presence of a systematic bias between SVO2 and SVJO2 measurements suggests a consistent discrepancy despite comparability of the two methods. The observed limits of agreement of -16.75% to + 11.31% indicate considerable variability. However, overlaid line graphs of some patients show a clear concurrent trend between measurements, indicating that as SVJO2 increases, SVO2 tends to increase, and vice versa. Despite small patient population, trend evaluation comparison between SvJO2 and SvO2 shows encouraging results for this novel non-invasive technique. Further data are required to enhance data distribution for statistical analysis and to confirm this preliminary results.

**References**

1. Vincent JL, De Backer D (2004) Oxygen transport-the oxygen delivery controversy. Intensive Care Med 30:1990- 1996
2. Scheinman MM, Brown MA, Rapaport E (1969) Critical assessment of use of central venous oxygen saturation as a mirror of mixed venous oxygen in severely ill cardiac patients. Circulation 40:165–172

**Fig. 1:**
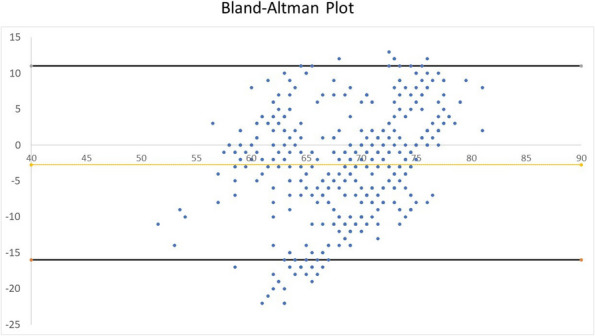
** (abstract A27).** See text for description

**Fig. 2:**
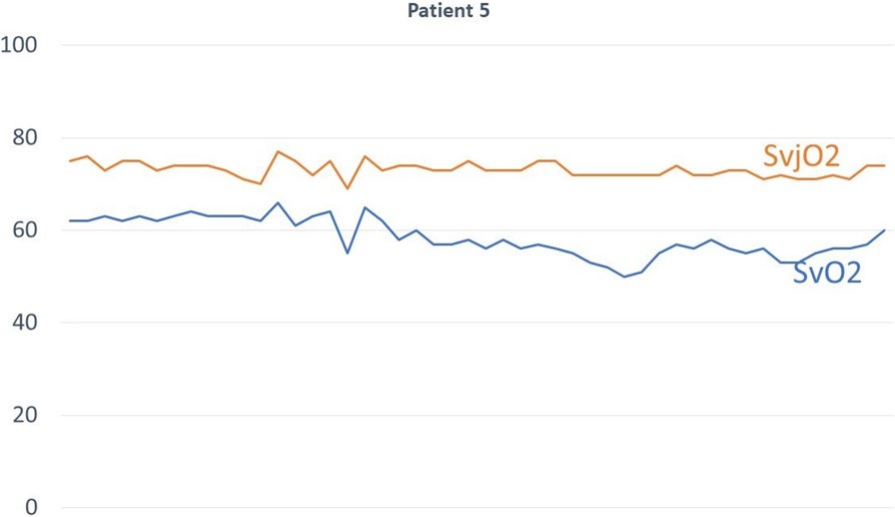
** (abstract A27).** See text for description

**Fig. 3:**
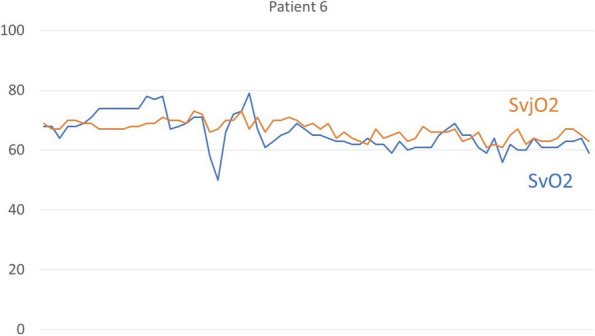
** (abstract A27).** See text for description

**Fig. 4:**
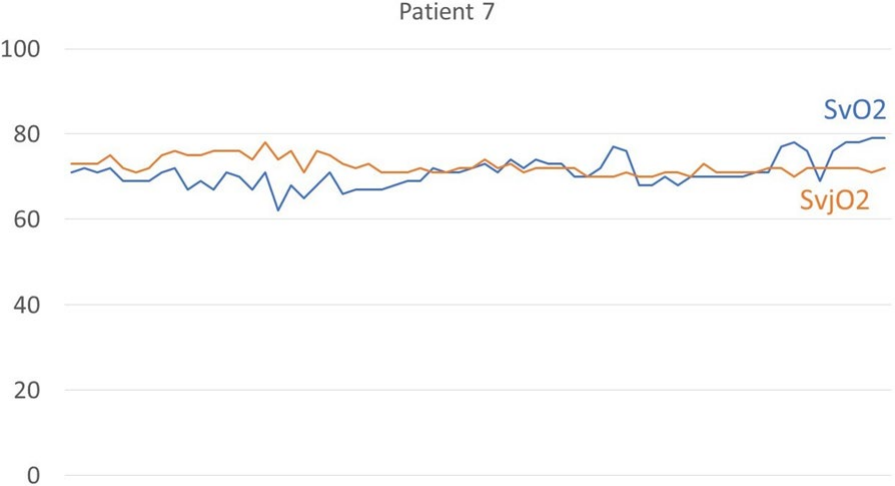
** (abstract A27).** See text for description

**Table 1:** **(abstract A27).** See text for description

	AGE	SEX	DIAGNOSIS	NUMBER OF MEASUREMENTS
PAZIENT 1	75	M	post-cardiotomy cardiogenic shock	6
PAZIENT 2	50	M	post-STEMI cardiogenic shock	35
PAZIENT 3	71	F	post-cardiotomy cardiogenic shock	72
PAZIENT 4	65	M	post-cardiotomy cardiogenic shock	10
PAZIENT 5	51	M	post-STEMI cardiogenic shock	48
PAZIENT 6	73	M	post-STEMI cardiogenic shock	70
PAZIENT 7	71	F	post-cardiotomy cardiogenic shock	64
PAZIENT 8	60	M	post-STEMI cardiogenic shock	72
PAZIENT 9	63	M	post-STEMI cardiogenic shock	29

**Table 2:** **(abstract A27).** See text for description

	Correlation	
Patient 1 (*n* = 6)	NA	NA
Patient 2 (*n* = 35)	0,33	weak
Patient 3 (*n* = 72)	0,14	negligible
Patient 4 (*n* = 10)	0,43	weak
Patient 5 (*n* = 48)	0,53	good
Patient 6 (*n* = 70)	0,57	good
Patient 7 (*n* = 64)	-0,2	negligible
Patient 8 (*n* = 72)	0,34	weak
Patient 9 (*n* = 29)	0,59	good
Patient 10 (*n* = 72)	0,45	weak

### A28 Early leukoencephalopathy during daratumumab treatment in a patient with multiple myeloma

#### G. Giugliano, C. Visani, A. Marra, C. Iacovazzo, G. Servillo

##### Department of Neurosciences, Reproductive and Odontostomatological Sciences, University of Naples, Federico II, Napoli, Italy

*Journal of Anesthesia, Analgesia and Critical Care 2024*, **4(1):**A28

Leukoencephalopathy is a disorder that affects the white matter in the central nervous system (CNS) [1–3]. There are only a limited number of case reports on leukoencephalopathy after daratumumab administration, showing daratumumab-related leukoencephalopathy (DRL) as a late, serious, and likely irreversible adverse event (AE) [4,5].

We report a case of DRL in a 52-year-old woman diagnosed with relapsed IgG-? multiple myeloma (MM). In May 2024, the patient was hospitalized after going to the emergency room for AKI, the diagnostic process placed the suspicion for multiple myeloma. In June 2024 the patient was admitted to the oncology department of our hospital for the continuation of diagnostic process and treatment. On 20th June 2024, based on the result of the bone marrow aspiration, diagnosis of multiple myeloma was made and therapy with daratumumab was started the following day. On June 24th, the patient presented an episode of seizure, numbness, loss of environmental contact and hypertension. An MRI was performed, showing diffuse signal alteration in the white matter, in the cerebellar hemispheres, in the bridge and midbrain, in the mammillary bodies, in the thalams, and the cortico-subcortical frontal-temporo-parietal bilaterally probably due to metabolic toxic distress. The patient was admitted to our ICU, sedated and intubated and started therapy with 1 mg/kg methylprednisolone every 12 h, and leucosamide for seizure treatment, after which she had a rapid improvement of the neurological symptoms within 24 h. On June 26th the patient was alert, conscious and cooperative, able to follow simple orders. Hemodynamics remained prone to hypertension. A new brain MRI (Fig. 1) was performed showing reduction of edema in sub-tentorial location, distributed homogeneously to biemisferic cerebellar white matter, bridge, midbrain, thalamic pulvinar (bilateral and symmetric). In the supratentotial area the MRI confirms the presence of edema of the subcortical white matter. The patient's family had given informed consent to the use of clinical data according to declaration of Helsinki.

Daratumumab is a human IgG-? monoclonal antibody targeting human CD38, with substantial efficacy against newly diagnosed and relapsed/refractory MM, and other plasma cell diseases [6,7]. Because CD38 is also expressed in various brain cells [8], along with the capability of daratumumab to cross the blood–brain barrier [9], this anti-CD38 antibody may directly attack cells responsible for myelin formation, resulting in demyelination [10]. Whereas daratumumab associated AEs involving CNS are relatively rare (e.g., only one case of progressive multifocal leukoencephalopathy reported thus far in the trials involving daratumumab).

**References**

1. Köhler W, Curiel J, Vanderver A (2018) Adulthood leukodystrophies. Nat Rev Neurol 14:94–105. 10.1038/nrneurol.2017.175
2. Servillo G, Bifulco F, De Robertis E, Piazza O, Striano P, Tortora F, Striano S, Tufano R. Posterior reversible encephalopathy syndrome in intensive care medicine. Intensive Care Med. 2007 Feb;33(2):230–6.
3. Servillo G, Striano P, Striano S, Tortora F, Boccella P, De Robertis E, Rossano F, Briganti F, Tufano R. Posterior reversible encephalopathy syndrome (PRES) in critically ill obstetric patients. Intensive Care Med. 2003 Dec;29(12):2323–2326.
4. Chari A, Attaya Suvannasankha A, Fay JW et al. (2017) Daratumumab plus pomalidomide and dexamethasone in relapsed and/ or refractory multiple myeloma. Blood 130:974–981. 10.1182/blood-2017-05-785246
5. Kareem SS, Viswanathan N, Sahebjam S et al. (2022) Leukoencephalopathy during daratumumab-based therapy: a case series of two patients with multiple myeloma. Onco Targets Ther 15:953–962. 10.2147/OTT.S365657
6. Mohyuddin GR, Mian H (2022) Daratumumab in newly diagnosed MM—incorporating lessons learnt from CASSIOPEIA, MAIA and beyond. Nat Rev Clin Oncol 19:3–4. 10.1038/s41571-021-00581-2
7. Kastritis E, Palladini G, Minnema MC et al. (2021) Daratumumab- based treatment for immunoglobulin light-chain amyloidosis. N Engl J Med 385:46–58. 10.1056/NEJMoa2028631
8. Hattori T, Kaji M, Ishii H et al. (2017) CD38 positively regulates postnatal development of astrocytes cell-autonomously and oligodendrocytes non-cell-autonomously. Glia 65:974–989. 10.1002/glia.23139.
9. Vercruyssen M, Hachem GE, Maerevoet M (2018) The daratumumab crosses the blood brain barrier. Clin Lymph Myelo Leuk 18:S289. 10.1016/j.clml.2018.07.229
10. Guerreiro S, Privat A-L, Bressac L et al. (2020) CD38 in neurodegeneration and neuroinflammation. Cells 9:471. 10.3390/cells9020471

**Fig. 1:**
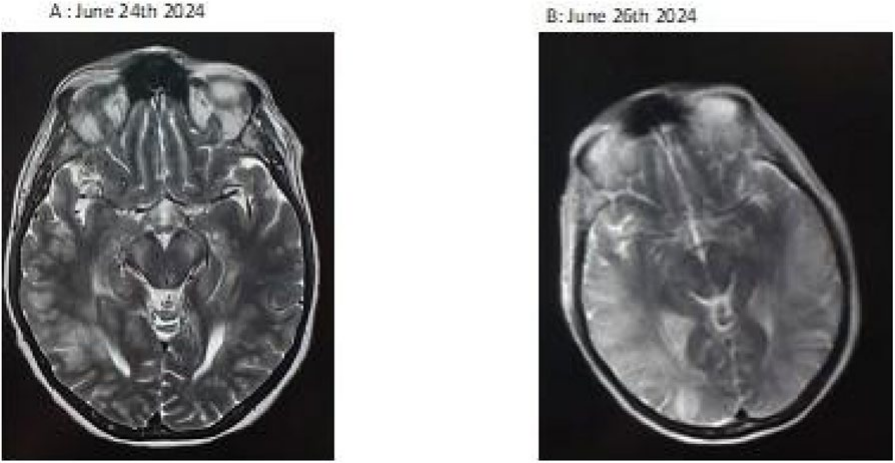
** (abstract A28).** Neuroimaging changes during the course of daratumumab – related leukoencephalopathy. A After the dose of daratumumab, 24th June. B 26th June after the 4th dose of metilprednisolone

## Invasive and non-invasive ventilation

### A29 Regional effects of different spontaneous breathing trials and comparison with the post-extubation phase using electrical impedance tomography

#### M. Riccardo^1^, P. Ferrara^1^, A. Andalò^1^, R. La Rosa^1^, E. Marangoni^2^, F. Montanaro^2^, C.A. Volta^1,2^, G. Scaramuzzo^1,2^, S. Spadaro^1,2^

##### ^1^Dipartimento di Medicina Traslazionale, Università degli studi di Ferrara, Ferrara, Italy; ^2^Unità operativa di Anestesia e Terapia Intensiva, Azienda Ospedaliero Universitaria Di Ferrara, Ferrara, Italy

###### **Correspondence:** M. Riccardo

*Journal of Anesthesia, Analgesia and Critical Care 2024*, **4(1):**A29

**Background**

Several settings are used to conduct spontaneous breathing trials (SBT), but it is unknown which one better simulates the post-extubation state. Electrical Impedance Tomography (EIT) is a non-invasive imaging technique that allows regional ventilation distribution evaluation in both mechanically ventilated and spontaneously breathing patients (1,2). The purpose of this study is to use EIT to evaluate the regional characteristics of ventilation distribution during three different SBTs and to assess which one more closely resembles the post-extubation spontaneous breathing condition.

**Material and Methods**

This is a prospective crossover physiological study on acute respiratory failure patients ready to be weaned conducted in the ICU of the Ferrara University Hospital (Ferrara, Italy). Each patient underwent 3 different SBTs in a random order, with a washout time of 20 min. The tested SBTs were: 1) LowPS (PS 7 cmH2O; PEEP 5 cmH2O); 2) LowPSZEEP (PS 7 cmH2O, PEEP = 0 cmH2O) and 3) ZeroPS (PS 0 cmH2O; PEEP 0 cmH2O) (3–4). During each SBT, airway pressure and flow were collected using a dedicated pneumotachograph and a 32-electrodes EIT device (BB2, Sentec) was used to record EIT images. After 2 h from extubation, a new EIT recording was performed during spontaneous breathing for comparison. Informed consent was obtained according to local regulations.

**Results**

We enrolled 12 patients, with a median age of 73 [68–84] years, a BMI of 25 [23–33] kg/m2 and after 4 [2–8] days of mechanical ventilation. All patients were successfully extubated after the protocol. During all SBTs, patients had a significantly lower global inhomogeneity index (GI), indicating lower heterogeneity in ventilation distribution. A significant lower number of Silent Spaces (SS) was found in the ZeroPS step, while a higher End-Expiratory Lung Impedance (EELI) was present in the LowPS phase. The relative distribution of ventilation significantly changed during the trial in all the regions of interest (Table 1).

**Conclusions**

In patients undergoing a SBT in PSV, ventilation distribution is different as compared to the post-extubation condition. Improved regional compliance and higher FRC is present when PEEP is used. Regional ventilation distribution during SBTs may differ from the one found after extubation.

**References**

1. Scaramuzzo G, Spadaro S, Waldmann AD, Bohm SH, Ragazzi R, Marangoni E, et al. Heterogeneity of regional inflection points from pressure–volume curves assessed by electrical impedence tomography. Crit care. 2019;23:119.
2. Spadaro S, Mauri T, Bohm SH, Scaramuzzo G, Turrini C, Waldmann AD, et al. Variation of poorly ventilated lungs units (silent spaces) measured by electrical impedance tomography to dynamically assess recruitment. Crit Care Lond Engl. 2018;22:26.
3. Cabello B, Thille AW, Roche Campo F, Brochard L, Gomez FJ, Mancebo J. Physiological comparison of three spontaneous breathing trials in difficult-to-wean patient. Intensive Care Med. 2010;36:1171–9.
4. Burns KEA, Soliman I, Adhikari NKJ, Zwein A, Wong JTY, Gomez-Builes C, et al. Trials directly comparing alternative spontaneous breathing trial techniques: a systematic review and meta-analysis. Cri Care Lond Engl. 2017;21:127.

**Table 1:** **(abstract A29).** significant * post-hoc comparison with post SBT step. All values reported as Median [IQR]; GI = global inhomogeneity index; EELI = end expiratory lung impedance; ROI = region of interest

	**LowPS**	**LowPSZEEP**	**ZeroPS**	**Post SBT**	**ANOVA**
GI (AU)	0.71 [0.68–0.78]*	0.73 [0.66–0.78]*	0.73 [0.62–0.77]*	0.77 [0.73–0.83]	*P* < 0.005
Silent spaces, (%)	6.5 [4.4–10]	7.8 [5.5–13]	6.5 [4.7–13]*	13 [6.9–18]	*p* = 0.04
EELI (AU*10^3)	51.3 [42.2–72.2]*	50.6 [42–69.5]	50.4 [41.5–69.2]	49.9 [43.3–67.2]	*p* = 0.05
ROI 1 (% of TV)	14.9 [8.2–18.5]	16.7[9.3–19.8]	13[6.4–17.3]	13.3[5.6–19.6]	p < 0.0001
ROI 2 (% of TV)	32.4[30.5–37.7]	34.0[31.2–39.7]	30.7[29.3–36.3]	32.6[28.2–35.9]	*p* < 0.0001
ROI 3 (% of TV)	35.1[28.6–39.4]	33[29.3–39.9]	36.8[32.7–40.8]	38[31.2–40.1]	*p* < 0.0001
ROI 4 (% of TV)	18.2[11.6–21.7]	16.1[10.2–19.5]	20.2[14.3–22.4]	17[12.2–23.1]	*p* < 0.0001

### A30 Early use of HFNO and P-SILI prevention in acute hypoxemic respiratory failure: a case report

#### F. Franzoi^1^, M. Losito^1^, S. Geppert^1^, S. Tantillo^2^, M. Guarnera^2^, I. Ottaviani^2^, I. Farinelli^2^, N. Cilloni^2^

##### ^1^Dipartimento Medicina e Chirurgia, Università di Bologna, Bologna, Italy; ^2^AUSL di Bologna, Ospedale Maggiore, UOC Terapia Intensiva e HUB Maxiemergenze, Bologna, Italy

###### **Correspondence:** F. Franzoi

*Journal of Anesthesia, Analgesia and Critical Care 2024*, **4(1):**A30

**Background**

The utilization of non-invasive respiratory support in the early management of acute hypoxemic respiratory failure (AHRF) remains a topic of debate. There is evidence suggesting that high flow nasal oxygen (HFNO) may reduce the need for endotracheal intubation, particularly in patients with a PaO2/FiO2 ratio (P/F) less than 200 mmHg (1). Conversely, spontaneous breathing with intense inspiratory effort may predispose to patient self-inflicted lung injury (P-SILI). To mitigate this risk, the assessment of inspiratory effort can be performed through measurement of esophageal pressure swing (δ Pes) (2).

We present a case of AHRF secondary to Mycoplasma pneumoniae infection managed with HFNO and δ Pes monitoring.

**Clinical case**

A 20-year-old personal trainer developed flu-like symptoms following a recent trip to Spain. After a week of persistent high fever, cough, and worsening tachypnea, he presented to the emergency department, where a CT scan revealed bilateral pneumonia with a tree-in-bud pattern. (Fig. 1) Empirical broad-spectrum antibiotic therapy was initiated.

Arterial blood gas analysis demonstrated hypoxemic respiratory failure without hypercapnia. AHRF did not respond to oxygen therapy with a reservoir mask.

Subsequently, the patient was admitted to the ICU and HFNO (50 l FiO2 65%) was initiated. A nasogastric tube with an esophageal pressure transducer was inserted to measure δ Pes. After the placement of the nasogastric tube, the initial esophageal pressure value was 14; we evaluated all factors that can contribute to an increase in swing, such as agitation, fever, and hypoxia. After initiating mild sedation, managing hyperpyrexia, and increasing the inspiratory fraction of oxygen, the esophageal pressure rapidly decreased to values between 10 and 12. Over a 12-h period of HFNO treatment, a reduction in δ Pes from 10–12 to 8 cmH20 was observed. Additionally, arterial blood gas analysis demonstrated progressive improvement in P/F ratio from 147 to 200 mmHg with a reduction in FiO2 to 50%, while maintaining normocapnia. The patient's respiratory discomfort decreased with resolution of fever and tachypnea, and oxygen therapy was transitioned to low-flow nasal cannula.

During the ICU stay, a PCR test for Mycoplasma pneumoniae on a nose-throat swab returned positive. Targeted therapy with Levofloxacin was continued, and the patient was transferred to the internal medicine department the following days.

Informed consent was obtained for data processing for clinical research purposes.

**Conclusion**

This case underscores the potential efficacy of HFNO, supported by δ Pes monitoring, in the management of severe pneumonia complicated by AHRF. Further research is warranted to elucidate the optimal approach to non-invasive respiratory support and prevention of SILI in the early stages of AHRF.

**References**

1. Frat JP, et al.; High-flow oxygen through nasal cannula in acute hypoxemic respiratory failure. N Engl J Med. 2015 Jun 4;372(23):2185–96.
2. Tonelli R, et al.; Early Inspiratory Effort Assessment by Esophageal Manometry Predicts Noninvasive Ventilation Outcome in De Novo Respiratory Failure. A Pilot Study. Am J Respir Crit Care Med. 2020 Aug 15;202(4):558–567.

**Fig. 1:**
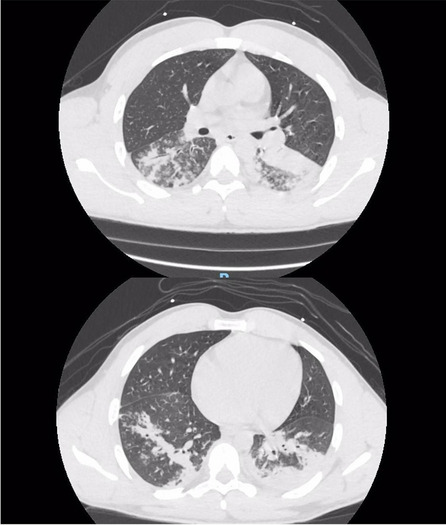
** (abstract A30).** CT scan that revealed bilateral pneumonia with a tree-in-bud pattern

### A31 Estimation of the diaphragm electrical activity and intercostal thickening fraction during different pattern of mechanical ventilation: PSV versus NAVA

#### D.C. Fachechi, G. Spinazzola, G. Maresca, T. Michi, G. Ferrone, E. Piervincenzi, R. Gaspari, M. Antonelli

##### Department of Anaesthesiology and Intensive Care Medicine, Catholic University of the Sacred Heart, Fondazione Policlini, Rome, Italy

###### **Correspondence:** D.C. Fachechi

*Journal of Anesthesia, Analgesia and Critical Care 2024*, **4(1):**A31

**Background**

In critically ill patients, respiratory muscle dysfunction is a major cause of failure to wean from mechanical ventilation (MV). Dysfunction of the diaphragm is common in critically ill patients, ranging from 33 to 95%. [1–5]. To date, the epidemiology of external intercostal muscle dysfunction is unknown [6–7].

However, the introduction of proportional ventilation modes has shown promising results in reducing the incidence of weaning failure [8–10]. The aim of this study is to evaluate the effect of two different ventilator modes (Pressure Support Ventilation, PSV and Neurally Adjusted Ventilatory Assist, NAVA) on intercostal muscles and diaphragmatic thickening fraction (TFic, TFdi) and respiratory drive.

**Materials and methods**

Informed consent was obtained and signed by the patients or their relatives. In our study, we enrolled patients admitted to Intensive Care Unit and on invasive mechanical ventilation for at least 24 h, who had failed at least one weaning attempt. After enrolment, patients received PSV for 30 min, NAVA for another 30 min, and PSV for another 30 min. The primary endpoint of the study was the assessment of TF (diaphragm and intercostal muscle) in three ventilation settings (PSV1, NAVA, PSV2). Secondary endpoints were the evaluation of respiratory drive, the patient-ventilator asynchrony, and the gas exchange during all ventilator setting tested (PSV1, NAVA, PSV2).

**Results**

Six patients were enrolled in the study from April 1 to September 30, 2023. The baseline characteristics of the patients are shown in Table 1. During all ventilator setting tested, no statistically significant differences were shown in terms of diaphragmatic excursion and speed of shortening of the diaphragm (TFdi PSV1 22%, TFdi NAVA 17%, TFdi PSV2 21%). Regarding TFic, we note an activation of the intercostal muscles in all the trials analyzed, although it is not statistically significant (TFic 6% PSV1, 5% NAVA, 4% PSV2) (Table 2). During NAVA trial, the mechanical and neural respiratory rate, the peak airway pressures presented the values significantly higher compared to PSV trials. Conversely, during PSV trials, VTmecc value was significantly higher than that delivered during NAVA. In terms of patient ventilator interaction, NAVA trial showed significantly shorter values of Delaytrinsp and Delaytrexp compared to the PSV trials (Fig. 1), a significantly higher value of Timesynch/Tineu (Fig. 2) and a significantly lower percentage of AI than the two PSV trials (15% PSV1 and PSV2, 0% NAVA, p < 0.01). In terms of blood gas exchanges, no statistically significant differences were highlighted (Table 3).

**Conclusions**

In patients with difficult weaning, intercostal muscle activation does not change with the use of proportional ventilation modes such as NAVA. However, NAVA has been shown to improve patient-ventilator interaction by reducing asynchronies and increasing the ventilator's mechanical support in phase with the patient's neural inspiration. Indeed, although not significant, NAVA shows a decreasing trend in respiratory drive (as measured by PTPEAdi, SwingEAdi and NME) compared to PSV.

**Fig. 1:**
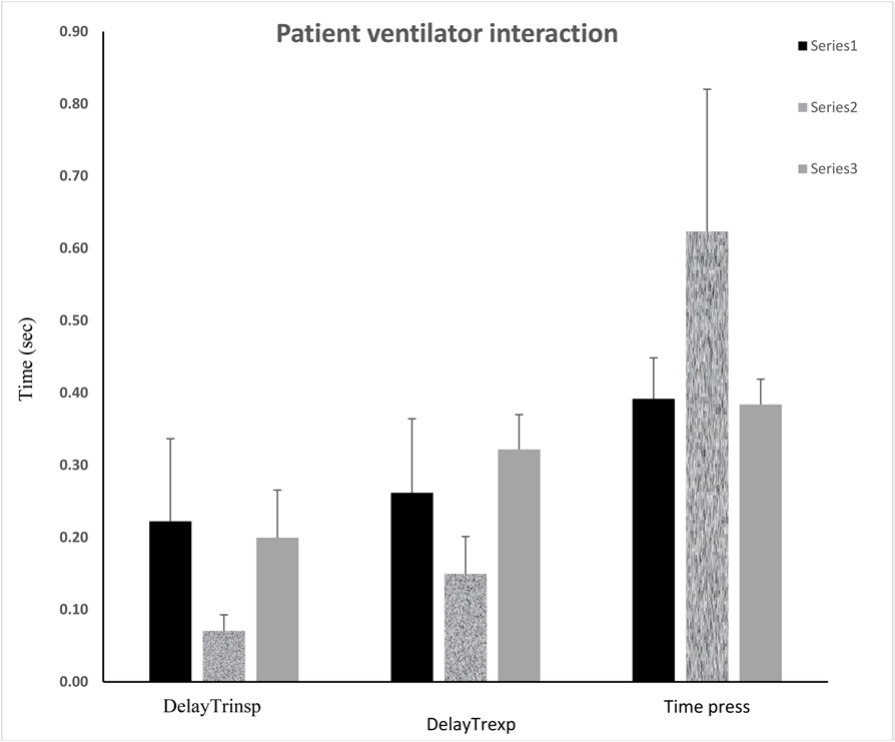
** (abstract A31).** Patience ventilator interaction. **: *p*<0.01, Black column: Pressure Support Ventilation trial1, Shaded gray column: Neurally Adjusted Ventilatory Assist trial, Dark gray column: Pressure Support Ventilation trial2

**Fig. 2:**
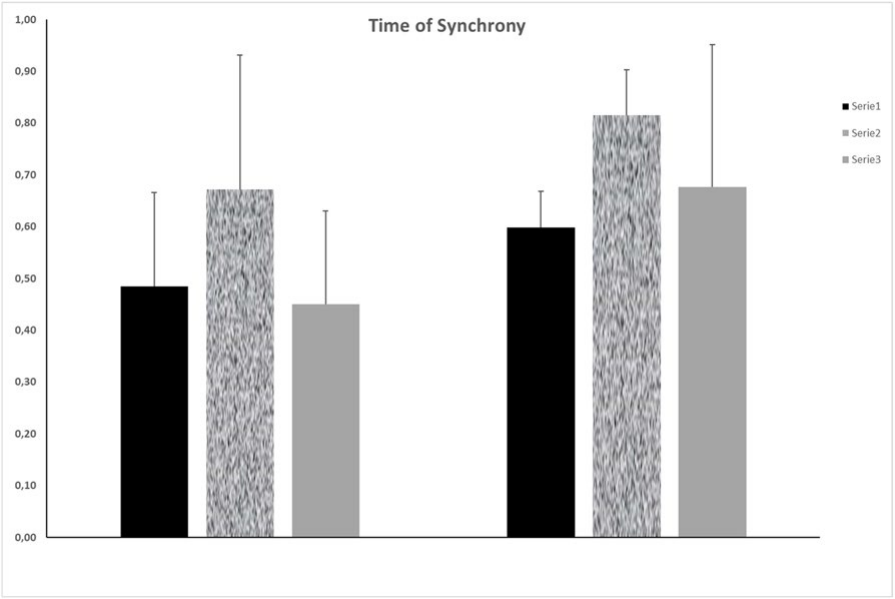
** (abstract A31).** **: *p*<0.01 Black column: Pressure Support Ventilation trial1, Shaded gray column: Neurally Adjusted Ventilatory Assist trial, Dark gray column: Pressure Support Ventilation trial2

**Table 1:** **(abstract A31).** Population

Age (aa)	67,33 ± 7,98
BMI (kg/m^2^)	29,84 ± 9,65
Gender	
Male	3
Female	3
Main cause of intubation	
Septic shock	4
Sepsis	1
COPD exacerbation	1
Comorbidity	2
Hypertension	2
Atrial fibrillation	1
Atrial Flutter	1
DMII	1
Liver transplantation	1
Colon cancer	1
Cytoriduction in ovarian cancer	1
Laparocele	1
Necrotizing fascitis Epilepsy	1
SAPS II	54,67 ± 23,13

**Table 2:** **(abstract A31).** See text for description

	PSV 1	NAVA	PV2	*P*
Diaphragmatic excurtion (mm)	10.65 ± 6.11	12.17 ± 8.87	9.9 ± 5.74	NS
Diaphragm inspiratory slope (cm/s)	2.51 ± 1.03	2.62 ± 1.8	2.25 ± 0.96	NS
Diaphragm thickening fraction(%)	22	17	21	NS
Intercostal muscles thickening fraction (%)	6	5	4	NS

**Table 3:** **(abstract A31).** See text for description

EAB	PSV 1	NAVA	PSV 2	*P*
pH	7.48 ± 0.06	7.48 ± 0.06	7.48 ± 0.05	NS
PaO2(mmHg)	95 ± 17.79	101.33 ± 18.43	93.33 ± 15.17	NS
PaCO2(mmHg)	42.5 ± 6.89	41.5 ± 7.1	43.33 ± 9.41	NS

### A32 Phonatory recovery of tracheostomised patients undergoing invasive home ventilation

#### P. Di Masi^1^, N. Cappellano^2^

##### ^1^Istituto di ricovero e cura a car. cientifico Saverio de Bellis, Castellana Grotte, Italy; ^2^Istituto di ricovero e cura a car. scientifico Saverio de Bellis, Castellana Grotte, Italy

###### **Correspondence:** P. Di Masi

*Journal of Anesthesia, Analgesia and Critical Care 2024*, **4(1):**A32

Neurodegenerative pathologies (ALS, MS, dystrophies) characterize a clinical picture of muscle weakness and ineffective cough that evolves towards progressive ventilatory respiratory failure. Only tracheostomy allows optimal control of the airway. In the more advanced stages, the use of long-term home ventilation becomes inevitable. There are clinical pictures characterized by a preservation of phonatory ability even in the most advanced stages, before tracheostomy. However, often starting to use the tracheostomy tube prevents the maintenance of this very important function in terms of the quality of life of these patients. In our department we have carried out the protected discharge of numerous neurodegenerative pathologies who have been subjected to artificial respiration with a home ventilator due to almost continuous dependence. For 4 out of 21 patients in the last 5 years, the presence of a preserved phonatory capacity prior to tracheostomy was an indication for a phonation protocol on artificial ventilation. Only for these patients was a procedure started that allowed them to undergo artificial ventilation with the emission of a flow through the vocal cords useful for the articulation of words. This protocol involved: 1) deflation of approximately 2/3 or 3/4 of the tracheostomy tube cuff after adequate drainage of the subglottic secretions, 2) maintenance of a VT (± 200-300 ml) with an optimal flow accepted by the patient in PCV mode, 3) calibration of the ventilator alarms (specific reduction of the VTE, VME alarm levels, VMI increase, maintenance of the remaining alarms), 4) pulse oximetry monitoring. No cannulas with fenestrated inner cannula were used (excessive flow with reduced patient compliance). The ventilator parameters were kept the same both in the capsizing phase with phonation and in the normal ventilation phase with the cuff inflated. Each phonation procedure required 4–5 min of adaptation with the elimination of the secretions surrounding the intra-tracheal axis of the cannula which had to remain completely patent to allow the retrograde upward passage of the flow through the vocal cords. The procedure was scheduled during daylight hours for periods limited to 3–5 h. In the 4 patients, after adequate training of the care giver and with the described protocol well executed, a recovery of the phonatory capacity was allowed, which proved to be fundamental in terms of quality of life. For all 4, the fatigue in phonation was even considered lower than in the period before the tracheostomy, thanks to the flows allowed by the PCV mode of the home ventilator. The number of patients for whom phonation on ventilation was possible, although small compared to the number of patients receiving home ventilation, represented an undoubted achievement for the optimization of their quality of life. The same patients who were given a specific satisfaction questionnaire documented the human depth of the recovery of at least the verbal relationship. Cases notoriously destined for the progressive and worsening limitation of motor functions.

Informed consent was obtained.

**Fig. 1:**
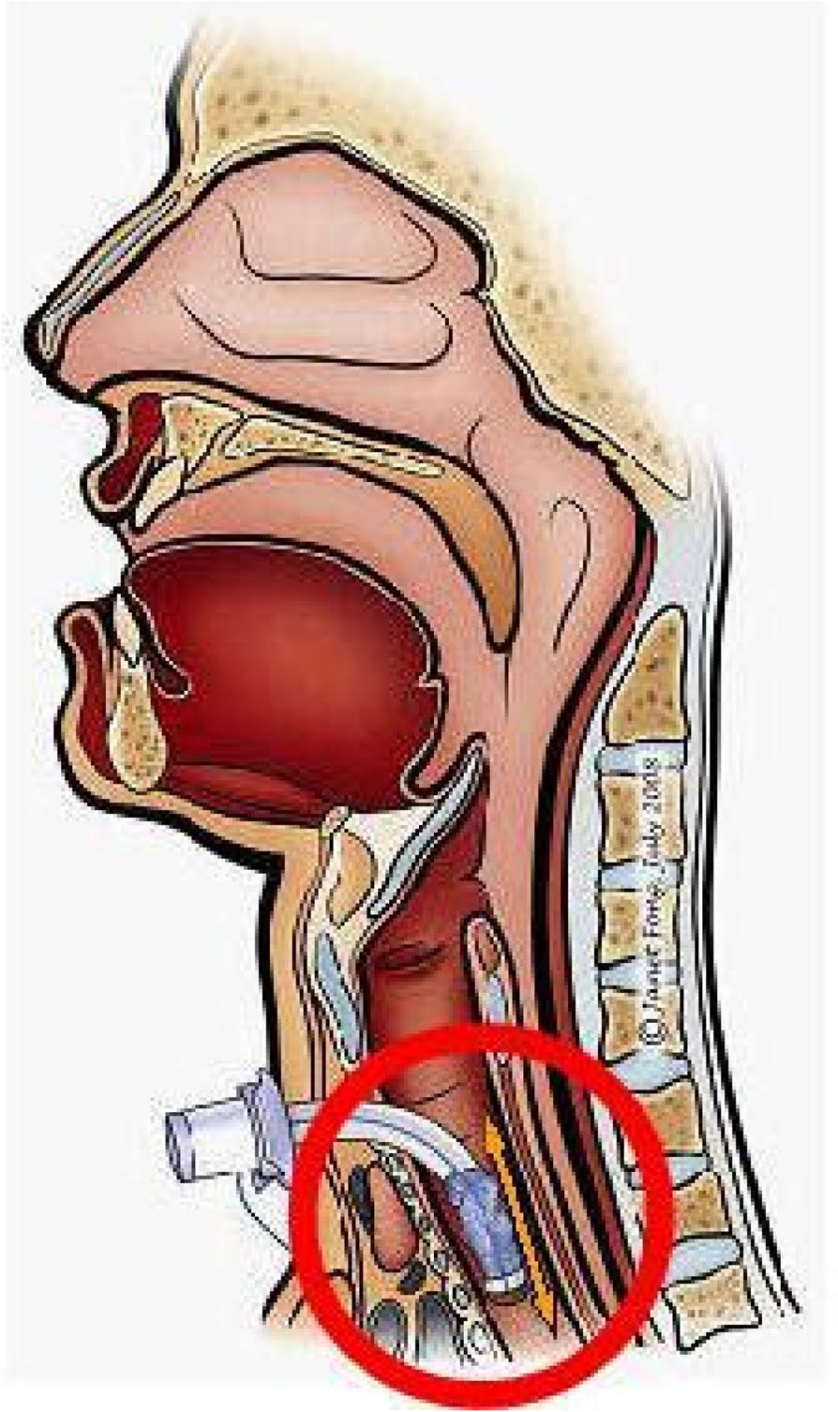
** (abstract A32).** See text for description

### A33 Regional citrate anticoagulation in an experimental model of extracorporeal CO 2 removal, preliminary data

#### L. Delle Cese^1^, M. Battistin^1^, G. Florio^2^, F. Ferrari^3^, L. Vivona^3^, S. Di Pellegrino^5^, V. Vago^5^, D. Dondossola^4^, F. Balestieri^5^, A. Zanella^1,3^, G. Grasselli^1,3^

##### ^1^Centro Ricerche Precliniche, Fondazione IRCCS Ca' Granda—Ospedale Maggiore Policlinico, Milano, Italy; ^2^Dipartimento di Fisiopatologia Medico-Chirurgica e dei Trapianti, Università degli Studi di Milano, Milano, Italy; ^3^Dipartimento Area Emergenza Urgenza Ospedale Maggiore Policlinico di Milano, Milano, Italy; ^4^Unit of Liver Transplant and General Surgery, Maggiore Polyclinic Hospital, IRCCS Ca' Granda Foundation, Milano, Italy; ^5^Università degli Studi di Milano, Milano, Italy

###### **Correspondence:** L. Delle Cese

*Journal of Anesthesia, Analgesia and Critical Care 2024*, **4(1):**A33

**Introduction**

Extracorporeal CO2 removal (ECCO2R) is an advanced life support strategy to control arterial CO2 tension and modulate ventilation, thus allowing potential effort control and ultra-protective ventilation in acute respiratory failure. Since its introduction in 1977 by Kolobow et al. technical advances allowed the translation into clinical practice.

However, to remove a clinically relevant amount of CO2 through the membrane lung (VCO2ML), ECCO2R requires large bore cannulas to provide high blood flow, thus reducing the viability of the treatment for the intensivists. ECCO2R, requiring high dose of heparin for systemic blood anticoagulation, exposes patients to high incidence of haemorrhagic complications.

We designed and tested a novel technique to safely apply regional citrate anticoagulation to ECCO2R (RCA-ECCO2R). Systemic citrate accumulation was prevented by extracorporeal citrate removal through ion-exchange resins (i-ER).

**Method**

Three healthy pigs were connected to a veno-venous circuit featuring a membrane lung (ML) followed by an hemofilter. Blood flow 350 ml/min. Sodium Citrate 136 mM was infused (463 ml/h) before ML. 1500 ml/min of countercurrent dialysate flow exits the hemofilter: 60 ml/min of dialysate was injected before ML to dilute the blood, while the remaining dialysate flows through a anionic-ER (to remove citrate), a cationic-ER (to remove calcium), and another ML (to enhance VCO2ML) before entering the haemofilter creating a closed-loop. Neutral hydroelectrolyte balance was obtained by 2150 ml/h dialysate discard and infusion of dedicated solutions (Fig. 1).

Tidal volume was kept constant at 8 ml/kg throughout the experiment. The respiratory rate was modified to maintain arterial PCO2 at 50 ± 2 mmHg. RCA-ECCO2R lasted 6 h.

**Results**

Citrate infusion and cationic-ER reduced iCa +  + in extracorporeal circuit from 1.36 ± 0.03 mmol/L (inlet) to 0.41 ± 0.03 mmol/L (pre-ML) and 0.23 ± 0.02 mmol/L (outlet). Thromboelastography showed normal coagulation in the inlet blood (R 6.8 ± 1.8 min, MA 71 ± 16) while full anticoagulation in the pre-ML (R 48 ± 18 min, MA 2 ± 2) and outlet (R > 60 min, MA 0 ± 0). Mean respiratory rate decreased from 19 ± 3 breaths/min (baseline) to 10 ± 2 breaths/min with conventional ECCO2R and after 6 h of RCA-ECCO2R was 9 ± 2 breaths/min. PaCO2 was kept stable during the whole experiment (50.1 ± 0.7 mmHg). Arterial pH value was 7.40 ± 0.03 (baseline), 7.41 ± 0.03 (ECCO2R) and 7.39 ± 0.03 RCA-ECCO2R. Sodium concentration was 136,6 ± 2 mmol/L (baseline), 136,3 ± 1,5 mmol/L (ECCO2R), 137,0 ± 2,6 mmol/L (RCA-ECCO2R). No haemolysis was recorded. No vasopressor/inotropes infusion was required during all experiments.

**Conclusion**

From preliminary experimental data, ECCO2R combined with regional citrate anticoagulation proved feasible and safe.

**Fig. 1:**
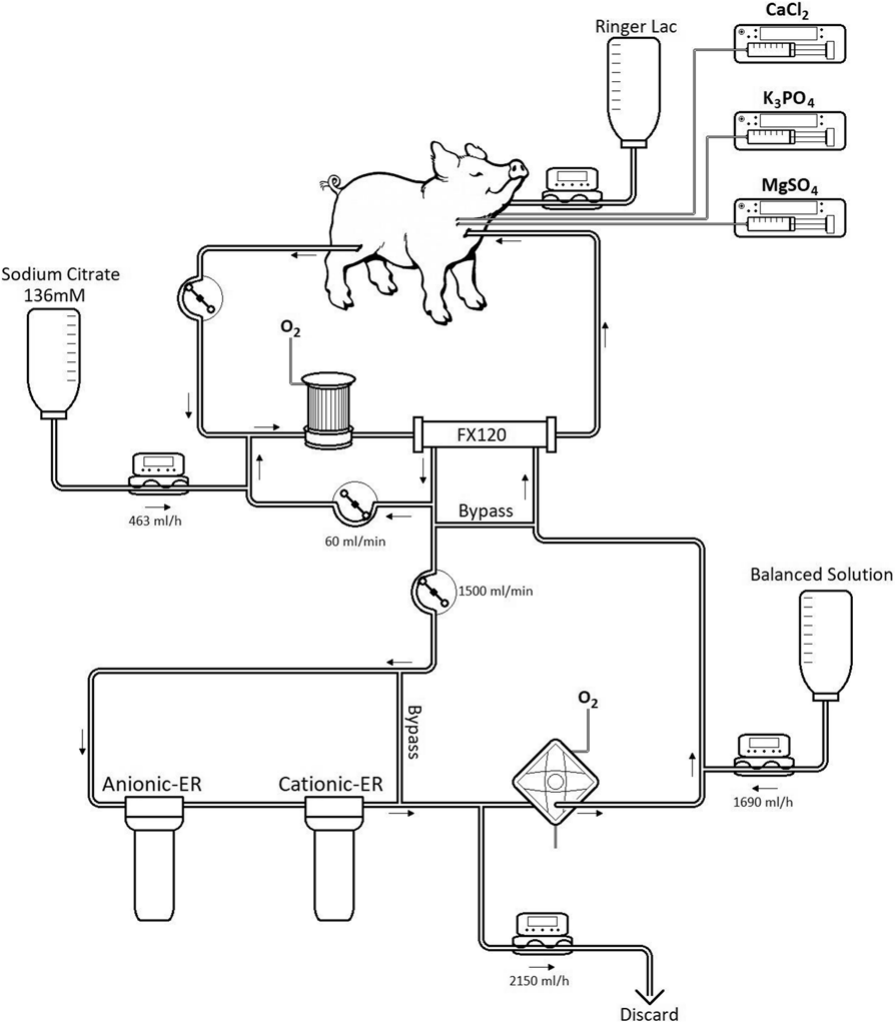
** (abstract A33).** See text for description

## Airway

### A34 Videolaryngoscope and flexible fibroscope: hybrid technique for difficult intubation

#### G. Ottoveggio^1^, B. Verro^2^, M. Lapi^1^, C. Saraniti^2^

##### ^1^Unit of Anesthesia, University Hospital of Palermo, Palermo, Italy; ^2^Unit of Otorhinolaryngology, University Hospital of Palermo, Palermo, Italy

###### **Correspondence:** G. Ottoveggio

*Journal of Anesthesia, Analgesia and Critical Care 2024*, **4(1):**A34

**Introduction**

As recommended by American Society of Anesthesiologists, difficult airway management needs different approaches for tracheal intubation in awake or asleep patient, depending on patient’s feature and anesthetist skills [1]. There are few studies in literature about combined use of videolaryngoscope and flexible fibroscope (FFS) in case of difficult intubation [2].

The study aims to demonstrate the usefulness of this hybrid technique as first-line choice in case of difficult intubation.

**Material and Methods**

A prospective study was conducted on patients undergoing general anesthesia due to ENT disease from January to April 2024 at University Hospital of Palermo, selected according to strict criteria. Ethical committee approval and patient’s informed consent were obtained. Patients with difficult intubation, both anticipated and unanticipated, were intubated by videolaryngoscope and FFS, under general anesthesia. All procedures were performed by the same anesthetist with expertise in FFS management and a second anesthetist keeping the videolaryngoscope in place (Fig. 1). The parameters collected were sex, age, comorbidities, type of surgery, previous general anesthesia, BMI, dentition, interincisive opening distance, thyromental distance, cervical mobility, Mallampati score, ASA, endotracheal tube size, number of intubation attempt, duration of intubation, pre- and post-intubation SpO2.

**Results**

13 consecutive patients were enrolled: 12 anticipated and 1 unanticipated difficult intubation. Most patients were men, mean age 49.08 ± 14.53 (range 23–76 years old), mean BMI 31.46 ± 6.50. Only 1 patient had fixed dental prosthesis (upper teeth), 4 patients had buck teeth. Most patients showed interincisive opening distance > 3 cm (92.31%), thyromental distance > 6 cm (69.23%), normal cervical mobility (69.23%). About Mallampati classification, 5 patients showed score II and 7 patients had score III; 2 patients had mandibular hypoplasia. The most used endotracheal tubes measured 7.0 and 7.5 mm in diameter. Intubation was successful at the first attempt in 12 cases; in case of unanticipated difficult intubation two attempts needed. This procedure lasted on average 12.2 ± 4.11 s, with SpO2 ranging from 97 and 100% (Table 1).

**Conclusion**

Videolaryngoscope provides a panoramic view of pharyngo-laryngeal district allowing a better FFS handling and tip orientation to ensure a better view of the glottis, also in case of blood, secretion, hypertrophic tongue base and laryngeal lesions.

This represents the first case series of difficult intubation management, both anticipated and unanticipated, by the combined use of videolaryngoscope and FFS, after induction of general anesthesia. Anyway, further studies need to confirm these preliminary results and to validate this protocol as first-line choice in case of difficult intubation. Moreover, anesthetist expertise is important.

**References**

1. Apfelbaum JL, Hagberg CA, Connis RT, Abdelmalak BB, Agarkar M, Dutton RP, et al. 2022 American Society of Anesthesiologists Practice Guidelines for Management of the Difficult Airway. Anesthesiology. 2022 Jan 1;136(1):31–81. 10.1097/ALN.0000000000004002.
2. Greib N, Stojeba N, Dow WA, Henderson J, Diemunsch PA. A combined rigid videolaryngoscopy-flexible fibrescopy intubation technique under general anesthesia. Can J Anaesth. 2007 Jun;54(6):492–3. 10.1007/BF03022046.

**Fig. 1:**
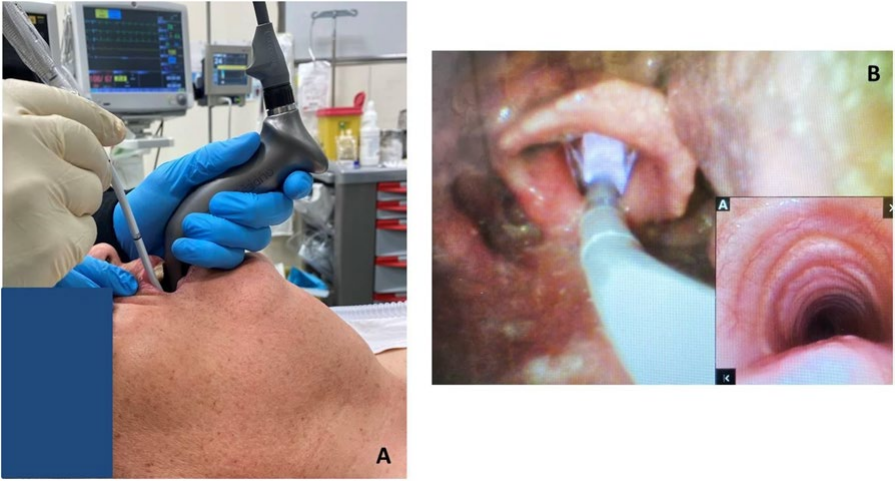
** (abstract A34).** Combined use of videolaryngoscope and flexible fibroscope for intubation: external view (A), endolaryngeal view (B)

**Table 1:** **(abstract A34).** Characteristics and pre operative parameters of included patients

Parameters	N (%)
**Sex**	
Female	4 (30.77)
Male	9 (69.23)
**Age (years)**	
Mean ± SD	49.08 ± 14.53
Range	23 – 76
**Surgery**	
Endoscopic sinus surgery	1 (7.69)
Septoplasty	2 (15.39)
Transoral laser laryngeal microsurgery	4 (30.77)
Tympanoplasty	4 (30.77)
Cochlear implantation	1 (7.69)
Parotidectomy	1 (7.69)
**BMI (mean ± SD)**	31.46 ± 6.50
**Dentition**	
Normal	5 (38.46)
Buck teeth	4 (30.77)
Edentulous	3 (23.08)
**Dental prostheses**	
Fixed	1 (7.69)
Removable	3 (23.08)
Upper teeth	3 (23.08)
Lower teeth	1 (7.69)
**Interincisive opening distance**	
> 3 cm	12 (92.31)
< 3 cm	1 (7.69)
**Thyromental distance**	
> 6 cm	9 (69.23)
< 6 cm	4 (30.77)
**Mallampati score**	
I	1 (7.69)
II	5 (38.46)
III	7 (53.85)
IV	0
**Cervical mobility**	
Normal	9 (69.23)
Hypomobile	4 (30.77)
Fixed	0
**Mandibular hypoplasia**	
Yes	2 (15.39)
Not	11 (84.61)
**ASA**	
I	0
II	7 (53.85)
III	6 (46.15)
IV	0
**Endotracheal tube size**	
5.5	1 (7.69)
6.0	1 (7.69)
6.5	1 (7.69)
7.0	5 (38.47)
7.5	3 (23.08)
**Number of intubation attempt**	
1	12 (92.31)
2	1 (7.69)
**Duration of intubation (seconds)**	
6	2 (15.39)
10	2 (15.39)
12	3 (23.08)
15	3 (23.08)
18	3 (23.08)
**Pre and post intubation SpO2**	
100–97	1 (7.69)
100–98	0
100–99	2 (15.39)
100–100	10 (76.9)
**Total**	**13 (100)**

### A35 Tracheal carina rupture following a mild domestic fall: a case report

#### G. Gazzè^1^, P. Papa^1^, V. Ceccarelli^1^, S. Orlando^1^, M. Paladino^1^, V. Caso^1^, M. Covotta^2^, E.M.A. Forastiere^2^, G. Torregiani^2^

##### ^1^Department of Anesthesia, Intensive Care and Pain Therapy, Policlinico Umberto I, Sapienza University of Rome, Roma, Italy; ^2^Department of Anesthesia, Intensive Care and Pain Therapy, IRCCS—Regina Elena National Cancer Institute, Roma, Italy

###### **Correspondence:** G. Gazzè

*Journal of Anesthesia, Analgesia and Critical Care 2024*, **4(1):**A35

**Background**

Tracheal injuries, including carina ruptures, are exceedingly rare conditions that primarily result from traumatic or iatrogenic events, although spontaneous occurrences are also documented. These pose a substantial challenge for clinicians due to their potential to cause unpredictable and rapid deterioration in patient condition.

**Case Report**

Our clinical case involves a 74-year-old woman weighing 75 kg (BMI 27.8 kg/m^2^), ASA 2, with a history of hypertension and prolonged corticosteroid use (22 years) for giant cell arteritis.

She was admitted to the emergency department of our institute (Policlinico Umberto I, Rome) after experiencing a tactile sensation of bilateral crepitus at the upper thoracic and cervical levels, following a mild chest trauma from a domestic fall.

Initially, the patient presented without respiratory symptoms; however, within the first 35 min post-trauma, SpO2 rapidly decreased from 97% on room air to 93%, despite receiving supplemental oxygen with a FiO2 of 0.6. Throughout this period, her hemodynamic parameters remained stable.

Chest X-ray confirmed extensive subcutaneous emphysema, also identified pneumomediastinum and moderate bilateral pneumothorax. A subsequent chest CT-scan further clarified these findings and additionally revealed a laceration approximately 1 cm in length at the level of the tracheal carina. Finally, fiberoptic bronchoscopy confirmed the presence of a tracheal carina rupture (Fig. 1).

The injury was classified, according to Cardillo's classification [1], as a Level III/B laceration, necessitating prompt surgical intervention due to the high risk of developing mediastinitis.

General anesthesia was induced with propofol, fentanyl, and rocuronium and a left-sided 37 Fr Robertshaw double-lumen tube was placed under fibrobronchoscopic guidance. Subsequently, a right thoracotomy was performed in the 4-5th intercostal space. The carina rupture was surgically repaired using a 3 × 2 cm autologous pericardial flap, secured with non-absorbable continuous sutures. The mediastinal pleura and a mediastinal fat pad were approximated over the repair site. Finally, two large-caliber chest tubes were inserted bilaterally.

Extubation was successfully performed in the ICU six hours after surgery. The pneumothorax, along with subcutaneous and mediastinal emphysema, resolved within a few days, and the chest tubes were removed on the third postoperative day. A bronchoscopy conducted on the seventh postoperative day revealed optimal repair. The patient was discharged the following day. At the six-month follow-up, there was no evidence of tracheal stenosis.

**Conclusions**

In cases of chest trauma of any severity, maintaining a high index of suspicion for tracheal injury is crucial to ensure early diagnosis, which correlates with a better prognosis for the patients.

Respiratory distress, subcutaneous emphysema, and pneumothorax are the most commonly found conditions on physical examination.

Prompt intubation below the injury site, along with early radiodiagnostic and bronchoscopic examinations, are prioritized. The choice of the most appropriate intervention, whether surgical or conservative, depends on the characteristics of the lesion and the clinical status of the patient.

Informed consent to publish had been obtained.

**Reference**

1. Cardillo G, Carbone L, Carleo F, et al. Tracheal lacerations after endotracheal intubation: a proposed morphological classification to guide non-surgical treatment. Eur J Cardiothorac Surg. 2010;37(3):581-7.

**Fig. 1:**
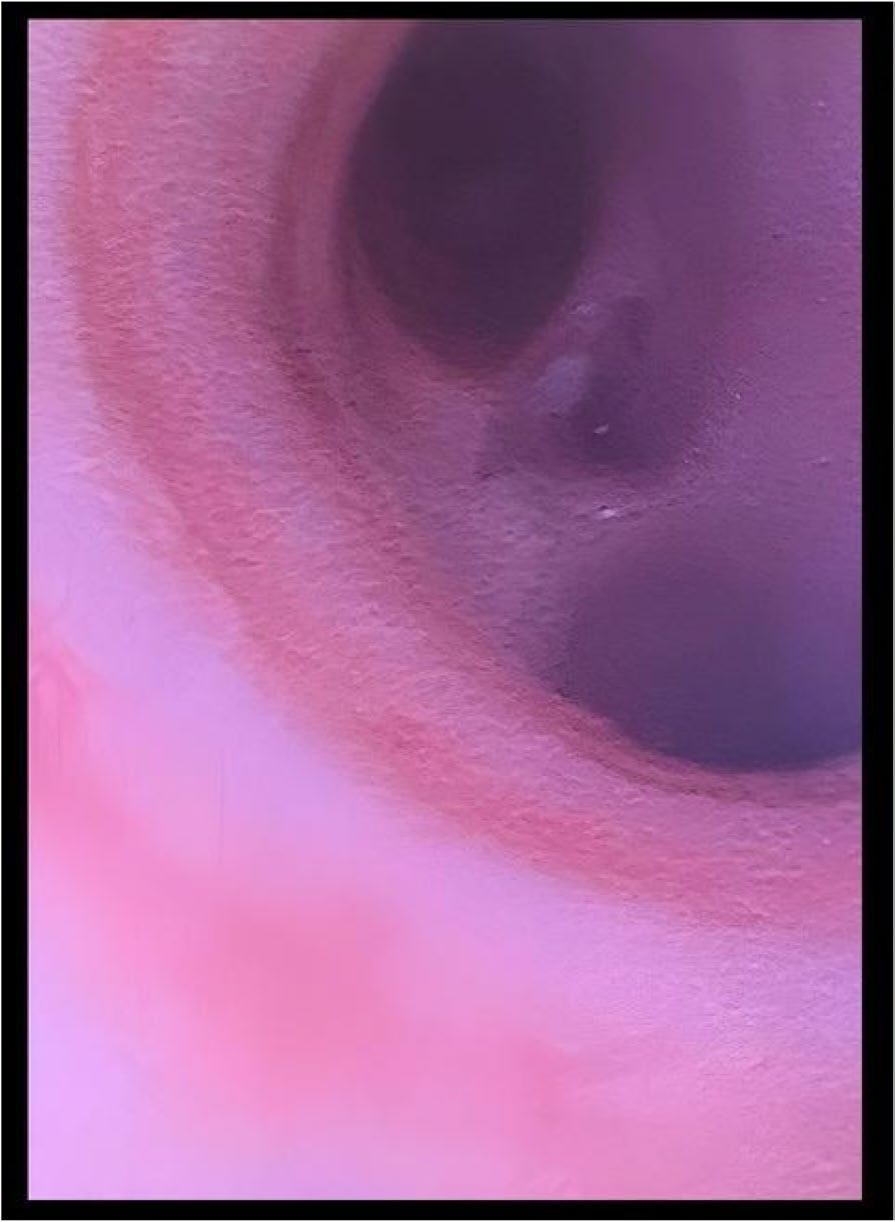
** (abstract A35).** Fiberoptic bronchoscopy confirmed the presence of a tracheal carina rupture

## Neuroanesthesia and neuroreanimation

### A36 Neurological disorders after upper abdominal surgery: a clinical case

#### R. Romano^1^, M. Esposito^1^, A. De Felice^1^, C. Migliaccio^2^, R. Montesano^1^, A. Famiglietti^1^, N. Lisco^1^, V. Amatucci^1^, V. Carucci^1^, A. Lauro^1^, C. Chierego^1^, A. Grasso^1^, G. Granato^1^, G. Azan^1^, A. Manto^3^, G. De Simone^1^

##### ^1^Department of Anaesthesia and Intensive Care for Liver Transplantation, AORN A. Cardarelli, Naples, Italy; ^2^Department of Hepatobiliary Surgery, AORN A. Cardarelli, Naples, Italy; ^3^Department of Neuroradiology, ASL Salerno, Nocera Inferiore, Italy

###### **Correspondence:** R. Romano

*Journal of Anesthesia, Analgesia and Critical Care 2024*, **4(1):**A36

A broad spectrum of neurological disorders has been described after upper abdominal surgery, especially associated with elderly and obese patients, including delirium, cognitive dysfunction, stroke, peripheral neuropathy, and encephalopathies. A small percentage of these disorders has been related to specific nutritional deficiencies, such as copper and Vitamin B12 deficiency myelopathies. However, despite the identification of multiple nutritional deficiencies, their correction does not necessarily lead to dramatic improvement in patients’ neurological status.

We report a case of a 70-year-old woman who underwent Blumgart pancreatico-jejunotomy for a G1 pancreatic ductal adenocarcinoma (PDAC). The patient showed delayed gastric emptying (DGE) with recurrent episodes of vomiting early after surgery initially associated with the evidence of a small pancreatic fistula which did not require further intervention. After a month in the hospital the patient was dismissed with prokinetics agents and weekly appointments for revaluation. Two weeks after the patient was readmitted to the ward in poor conditions reporting low oral intake worsened by persistent vomiting and quickly developed altered mental status, hyposthenia, and horizontal nystagmus. Due to the deterioration of the consciousness the patient was admitted to our Intensive Care Unit and promptly intubated. A Head CT scan was performed excluding any acute cerebrovascular events. An abdomen CT scan showed the presence of gastroparesis with stasis of the gastrografin in the gastric lumen and no progression beyond the gastro-jejunotomy in the absence of mechanical obstruction. Brain magnetic resonance imaging (MRI) was performed few days after exhibiting bilateral and symmetrical hyperintense signal on T2-weighted and FLAIR imaging scans in hemispheric cerebellar areas, medial and posterior thalami, and frontal-parietal and occipital cortex, suggesting a differential diagnosis between Wernicke’s encephalopathy (WE) and Posterior Reversible Encephalopathy Syndrome (PRES). A series of electroencephalograms reported a diffuse non-specific non-epileptiform background slowing with involvement of the reticular activating system (RAS). At the time of the admission to ICU and for the next weeks, there were no signs of uncontrolled hypertension, acute renal failure, infections, which may be associated with PRES. The well-known history of upper abdominal surgery and malnutrition and the pattern of MRI alterations with thalami’s involvement were suggestive of a Wernicke’s encephalopathy. Adequate therapeutic support was provided and supplementation with thiamine administered. Tracheotomy was performed and a PEG was inserted. However, despite treatment and significant improvement of the MRI scans, minor clinical improvements were noted.

Upper abdominal surgery is associated with the development of several neurological disorders, including various forms of encephalopathies. Early diagnosis is crucial, but challenging, and timely management of malnutrition deficiencies is essential. Nevertheless, despite big efforts a successful outcome may not been achieved.

Informed consent was obtained.

### A37 Scalp block in awake surgery for the implantation of external ventricular drainage: a retrospective study

#### A. De Simone, R. Annunziata, A. Longobardi, S. Gomez, D. Sannino, C. Eziandio, E. Petrillo, D. Cappuccio, M. Notaro, C. Coretti, G. Lauro, M.E. Porcelli, R. Castellone, P. Rinaldi, L. Baiano, O. Volino, R. Villani, M. Mariani

##### AORN A Cardarelli, Napoli, Italy

###### **Correspondence:** O. Volino

*Journal of Anesthesia, Analgesia and Critical Care 2024*, **4(1):**A37

Awake Surgery is a widely accepted technique and today it plays an important role in neurosurgery and neuroscience1. In case of brain damage, its use can promote the rapid recovery of neurological functions, which is crucial to re-establish a normal functioning and to improve the quality of life2.

The scalp block is very effective in reducing intraprocedural pain in craniotomies where, associated with general anesthesia, it determines a reduction in the autonomic response to pain, reducing the need for opioids. Use of preincisional scalp blockade grants many benefits, including improved hemodynamic stability, decreased anesthetic and opioid requirements, and enhanced postoperative analgesia.

This technique allows to simultaneously block the major and minor occipital nerves, the supraorbital and supratrochlear nerves, the zygomaticotemporal nerve, the auriculotemporal and the major auricular nerve3.

Insertion of an external ventricular drain (EVD) is arguably one of the most common and most important lifesaving procedures encountered in the neurologic intensive care unit4. Various types of acquired brain injury, such as intracranial hemorrhage with intraventricular extension, subarachnoid hemorrhage, traumatic brain injury, and bacterial meningitis, may benefit from EVD insertion.

For this reason, we wanted to study the benefits of a scalp block in the positioning of external ventricular drainage (EVD). The primary endpoint was to evaluate intraprocedural pain through autonomic responses and the need for sedation or intraprocedural analgesics, as secondary endpoints we evaluated post-operative pain up to 24 h, time to recovery of cognitive functions, hospitalization time, operator satisfaction.

Patients of the neurosurgery department who were candidates for External Ventricular Lead placement who were over 18 years old, not requiring neuroprotection, with GCS > 9 (and at least M5) have been enrolled in the present study and evaluated. We performed a right unilateral ultrasound-guided Scalp Block, administering a mixture composed of lidocaine 1% + ropvacaine 0.4% + dexamethasone 0.02% + clonidine 7.5 mcg/ml.

**Declaration of patient consent**

The authors certify that they have obtained all appropriate patient consent forms. In the form, the patient has given the consent for his/ her images and other clinical information to be reported in the journal.

**References**

1. Shinoura N, Yamada R, Tabei Y, Saito K, Suzuki Y, Yoshida M, Takahashi M, Nakamura O, Takayama Y, Yagi K. [Awake surgery plays a role in neurosurgery and neuroscience]. Brain Nerve. 2008 Aug;60(8):941–7. Japanese.
2. Hiruta R, Futamura M, Fujii M. [Awake surgery for preservation of higher brain functions]. No Shinkei Geka. 2023 May;51(3):540–550. Japanese.
3. Osborn I, Sebeo J. Scalp block during craniotomy: a classic technique revisited. J Neurosurg Anesthesiol. 2010 Jul;22(3):187–94.
4. Dey M, Jaffe J, Stadnik A, Awad IA. External ventricular drainage for intraventricular hemorrhage. Curr Neurol Neurosci Rep. 2012;12:24–33.

### A38 Abdominal pain, neurological symptoms and hyponatremia: do not forget porphyria

#### M.C. Laganà^1^, A. Guglielmi^2,3^, G.M. Mazza^3^, M. Hurpet^1^, G. Sala^1^, G. Salve^3^, A. Bolongaro^3^, G. Tavazzi^1,3^, F. Mojoli^1,3^, M. Pagani^3^, F. Sciutti^3^

##### ^1^University of Pavia, Department of Clinical, Surgical, Diagnostic and Pediatric Sciences, Pavia, Italy; ^2^University of Pavia, PhD in Experimental Medicine, Pavia, Italy; ^3^Intensive Care Department 1, Fondazione IRCCS Policlinico San Matteo, Pavia, Italy

###### **Correspondence:** M.C. Laganà

*Journal of Anesthesia, Analgesia and Critical Care 2024*, **4(1):**A38

**Background**

Porphyrias are a heterogeneous group of metabolic diseases that result from heme biosynthesis pathway deficiencies (Fig. 1-Pathway)[1]. Acute intermittent porphyria(AIP), the most common and severe form of acute porphyria, is caused by a partial deficiency in porphobilinogen deaminase(PBGD), leading accumulation of certain porphyrin precursors, such as aminolevulinic acid(ALA) and porphobilinogen(PBG). Although rare, AIP can be life threatening and clinical signs and symptoms are heterogeneous and non-specific.

**Case report**

A 26-year-old woman was admitted repetitively to Emergency Department(ED) for abdominal pain. Despite laboratory investigations were in range of normality and CT scan was negative, the onset of severe hyponatremia (Na + 108 mmol/L) required admission to ICU. Following stabilization, the patient was transferred to ward to continue testing. However, new symptoms emerged, and she began experiencing migraines with vision loss, spurring renewed urgency in the diagnostic inquiry. Brain MRI (Fig. 2-MRI) showed the presence of Posterior Reversible Encephalopathy Syndrome(PRES). Blood tests revealed persistent hyponatremia (Na +  < 110 mmol/L) and hyperporphyrinuria: urine sample over a 24-h period unveiled elevated levels of total porphyrins (863.2 mcg/24 h;r.r. < 150), including uroporphyrin (665.6 mcg/24 h;r.r. < 25), heptacarboxyporphyrin (25.5 mcg/24 h;r.r. < 5) and coproporphyrin3 (114.8 mcg/24 h;r.r. < 75). She was re-admitted to the ICU with drowsiness, blindness, abdominal pain, hypoglycemia, and hyponatremia. Based on clinical suspicion and the evolution of neurological signs (Fig. 3-Diagnostic algorithm)[2], hemin was administered intravenously for four days at a daily dose of 250 mg, alongside natremia and glycemia correction. Quantitative confirmatory tests, such as ALA and PBG determination, were performed and diagnosis of acute porphyria has been made. EEG showed focal right epileptiform activity, prompting Levetiracetam therapy. A gradual improvement of symptoms was observed, normal vision and neurological state were restored; natremia was stabilized. The patient was transferred to ward for ongoing follow-up care and, after discharge from hospital, she was tested with the following scores: Modified Rankin Scale 0 and Glasgow Outcome Scale Extended 8. She was referred to a specialized porphyria center where the diagnosis of AIP was confirmed with DNA Sequence Analysis.

**Conclusions**

Awareness is fundamental for timely correct diagnosis. AIP should be suspected in young women presenting with persistent abdominal pain, refractory hyponatremia, and neurological symptoms. The quantification of ALA and PBG from a spot urine sample with adequate light protection represents the first line of laboratory testing for acute porphyria. Confirmation of the diagnosis is based on the analysis of urine, plasma and fecal porphyrins and it is required if laboratory tests are positive or if clinical symptoms persist. The diagnosis of porphyria includes: DNA Sequence Analysis, the “gold standard” method to recognize mutations associated with the forms of porphyria; Multiplex Ligation-Dependent Probe Amplification(MLPA), an helpful tool for detecting a single exon or long sequence deletion; and Next-Generation Sequencing(NGS), used predominantly for research rather than for diagnosis. However, these methods require special equipment and expertise and are limited to specialized centers.

Written consent for publication has been obtained from the patient.

**References**

1. Stölzel U, Doss MO, Schuppan D. Clinical Guide and Update on Porphyrias. Gastroenterology. 2019;157(2):365–381.
2. Besur S, Schmeltzer P, Bonkovsky HL. Acute Porphyrias. J Emerg Med. 2015 Sep;49(3):305–12.

**Fig. 1:**
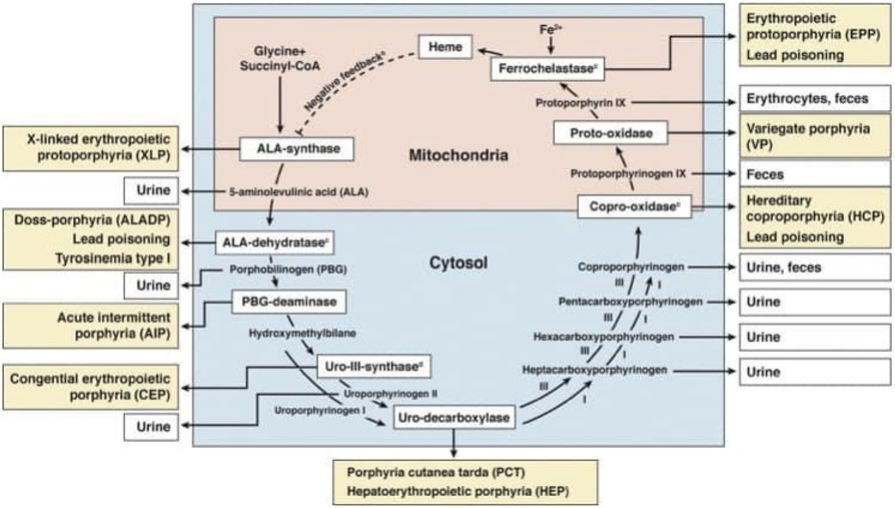
** (abstract A38).** Pathway

**Fig. 2:**
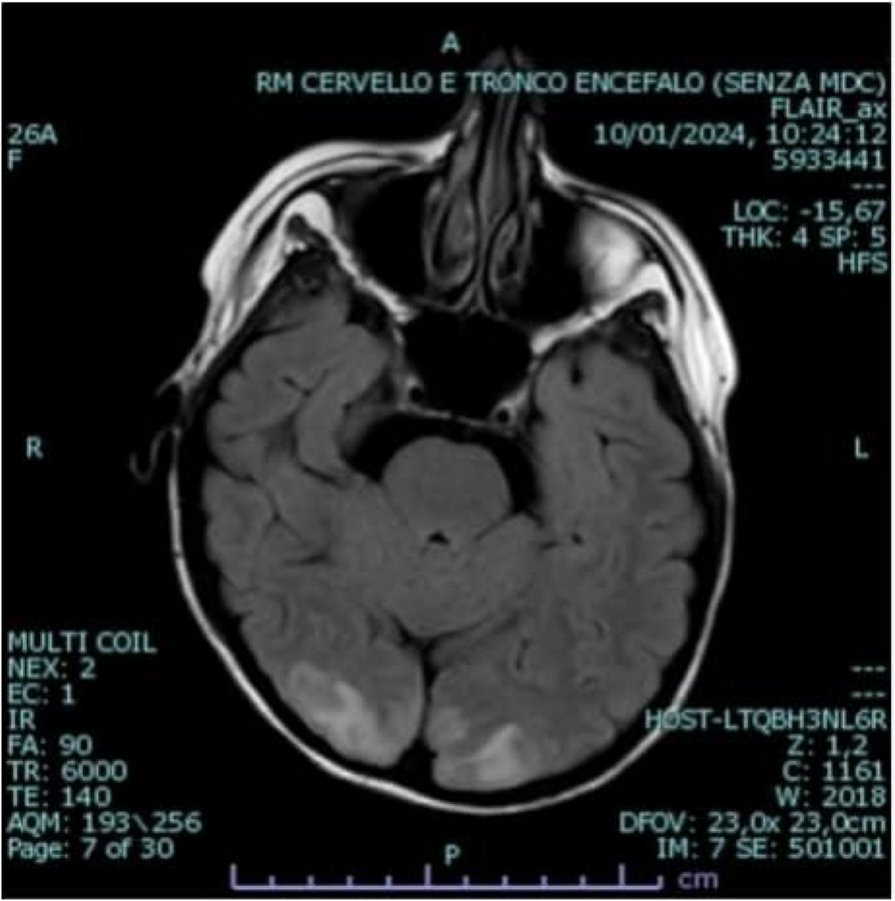
** (abstract A38).** MRI

**Fig. 3:**
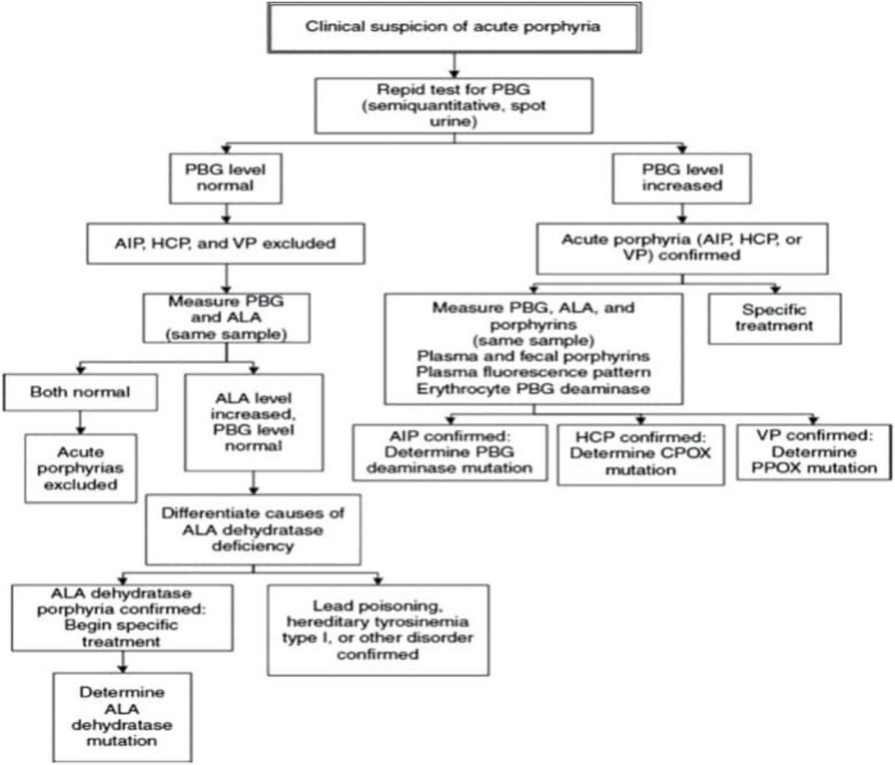
** (abstract A38).** Diagnostic algorithm

### A39 Recovery from major cerebral arteriovenous malformation rupture in a young male: a case report

#### M. Hurpet^1^, A. Guglielmi^2,3^, G.M. Mazza^3^, M.C. Laganà^1^, G. Sala^1^, A. Bolongaro^3^, A.R. Aliberti^3^, A. D'arcamo^3^, D. Radolovich^3^, C. Lo Coco^3^, C. Belotti^3^, M.A. Garlando^3^, G. Tavazzi^1,3^, F. Mojoli^1,3^, F. Sciutti^3^, M. Pagani^3^

##### ^1^University of Pavia, Department of Clinical, Surgical, Diagnostic and Pediatric Sciences, Pavia, Italy; ^2^University of Pavia, PhD in Experimental Medicine, Pavia, Italy; ^3^Intensive Care Department 1, Fondazione IRCCS Policlinico San Matteo, Pavia, Italy

###### **Correspondence:** M. Hurpet

*Journal of Anesthesia, Analgesia and Critical Care 2024*, **4(1):**A39

A healthy 18-year-old male developed severe headache, followed by severe decline in neurological status.

Emergency team found the patient having seizures, with GCS3, requiring intubation.

At ER admission the neurological assessment confirmed GCS 3, pupils were miotic, photo-pupillary, corneal and cough reflexes where absent. Head CT-scan (Fig. 1) revealed cerebellar intracerebral hemorrhage (ICH) with brainstem compression and tetra-ventricular subarachnoid hemorrhage. Angio-CT showed the rupture of a cerebral arteriovenous malformation (AVM) with arterial feeding from the PICA/left vertebral artery and venous drainage in the torcula. The patient immediately underwent posterior cranial fossa decompression with posterior craniotomy, hematoma evacuation and external ventricular drainage positioning (Fig. 2).

After surgery the patient was admitted to the Neuro-ICU where sedation and antiepileptic prophylaxis with leviteracetam were started. The first post-operative neurological assessment showed: GCS3, isochoric-isocyclic miotic pupils with no light reflex, normal pharyngeal and cough reflex.

On day 4 somatosensory and auditory evoked potentials were evaluated and showed normal reactivity. EVD drained hematic liquor and measured an ICP within range. TCCD showed normal velocities. Episodes of diabetes insipidus were treated with desmopressin. After a diagnostic angiography (Fig. 3); the embolization of a pseudoaneurysm created by the rupture of the AVM was performed.

Percutaneous tracheostomy was performed and sedatives where suspended: the patient was able to obey commands without deficit and opened his eyes spontaneously, with convergent strabismus. 16 days after surgery EVD weaning was started with complete removal seven days later.

On day 27 AVM was successfully surgically repaired through microsurgery. After surgery the neurological status was stable. A control angiography performed on day 30 showed re-habitation of the repaired AVM therefore a revision surgery was performed on day 31 without any change to the neurological assessment.

The ICU permanence was complicated by ventilator associated pneumonia and septic shock, successfully treated with vasopressors and antibiotics. The patient was weaned off mechanical ventilation and percutaneous endoscopic gastrostomy was placed before ICU discharge.

The patient was transferred to rehabilitation on day 51 but needed readmission to the neurosurgery ward after 3 days: his neurological status had declined presenting with stupor, with evidence of hydrocephalus at head CT (Fig. 4). The patient was successfully treated with medical therapy with complete neurological recovery after 15 days. He was discharged home after 3 months in the rehabilitation center.

At 6 months the Glasgow Outcome Scale (GOSE) was 8, with complete recovery; a category VII on the Functional Ambulatory Scale. On the Index of Indipendence in Activities of Daily Living scale (ADL) the score was 12 at 6 months and 0 on the Modified Ranking scale (mRANKIN).

**Conclusion**

At admission the patient presented with no brainstem reflexes therefore a negative prognosis was expected.

This case report demonstrates how clinical and surgical intervention can change the prognosis and highlights the recuperative powers of the brain which is especially fast in younger individuals.

Written consent for publication has been obtained from the patient.

**Fig. 1:**
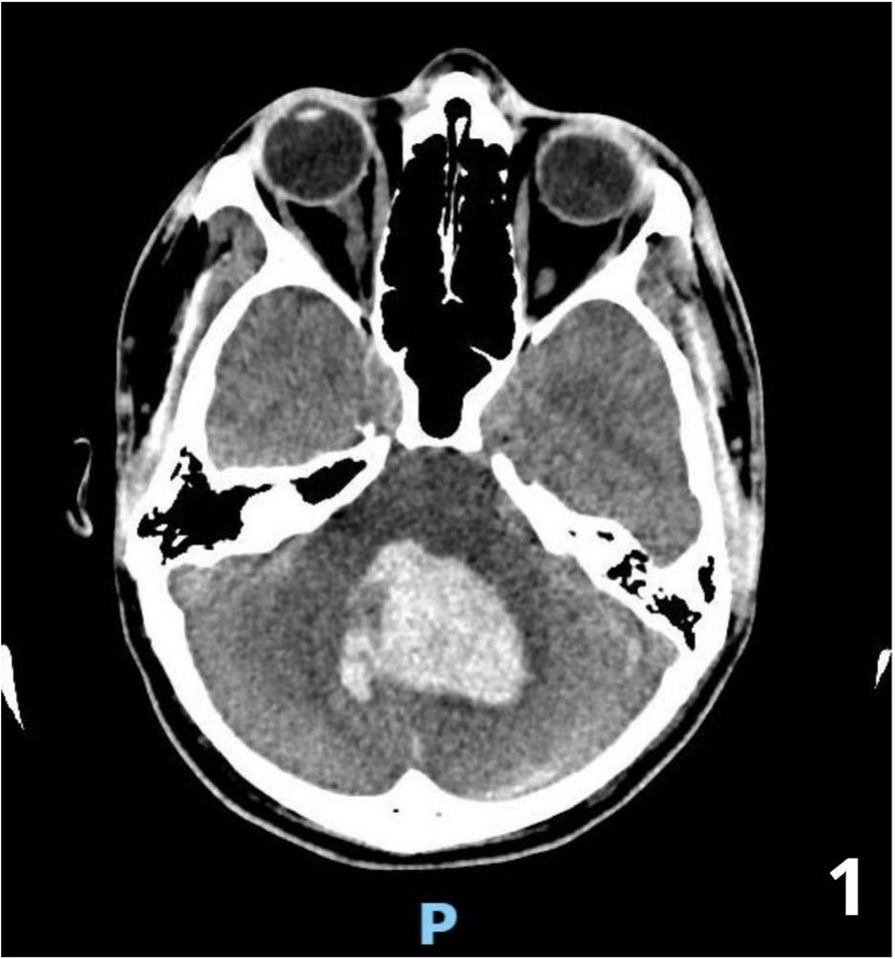
** (abstract A39).** Head CT-scan

**Fig. 2:**
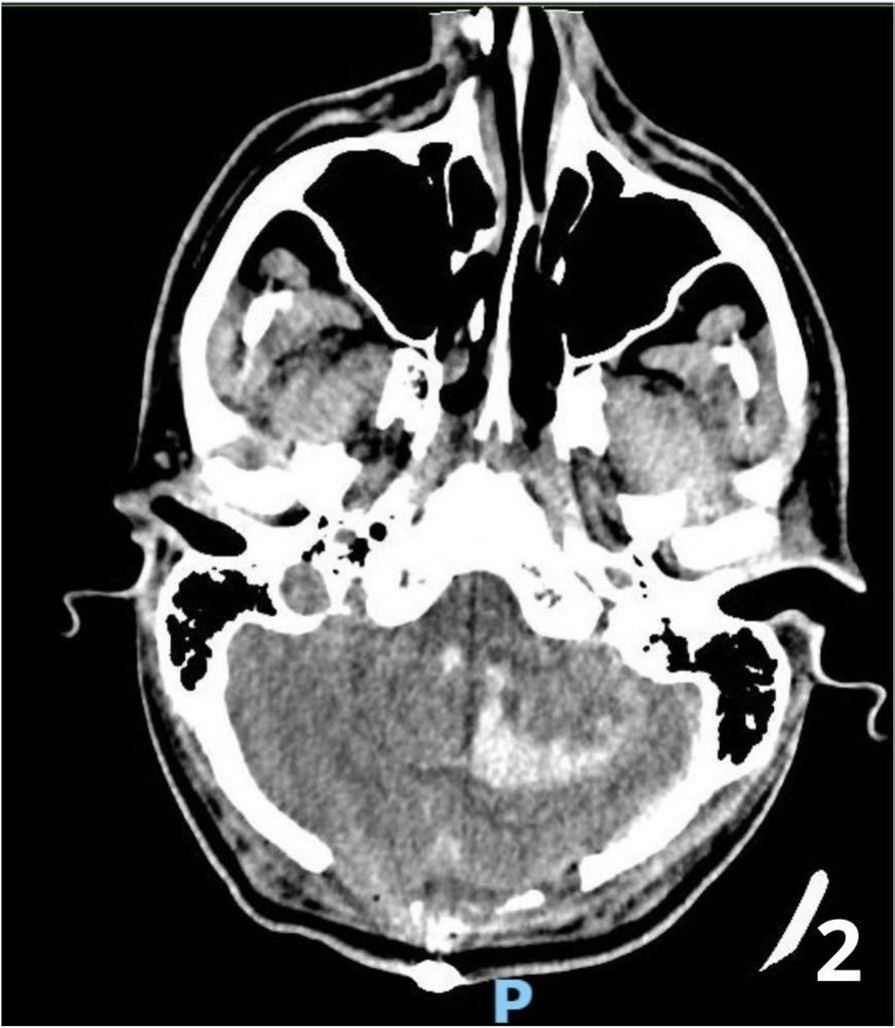
** (abstract A39).** See text for description

**Fig. 3:**
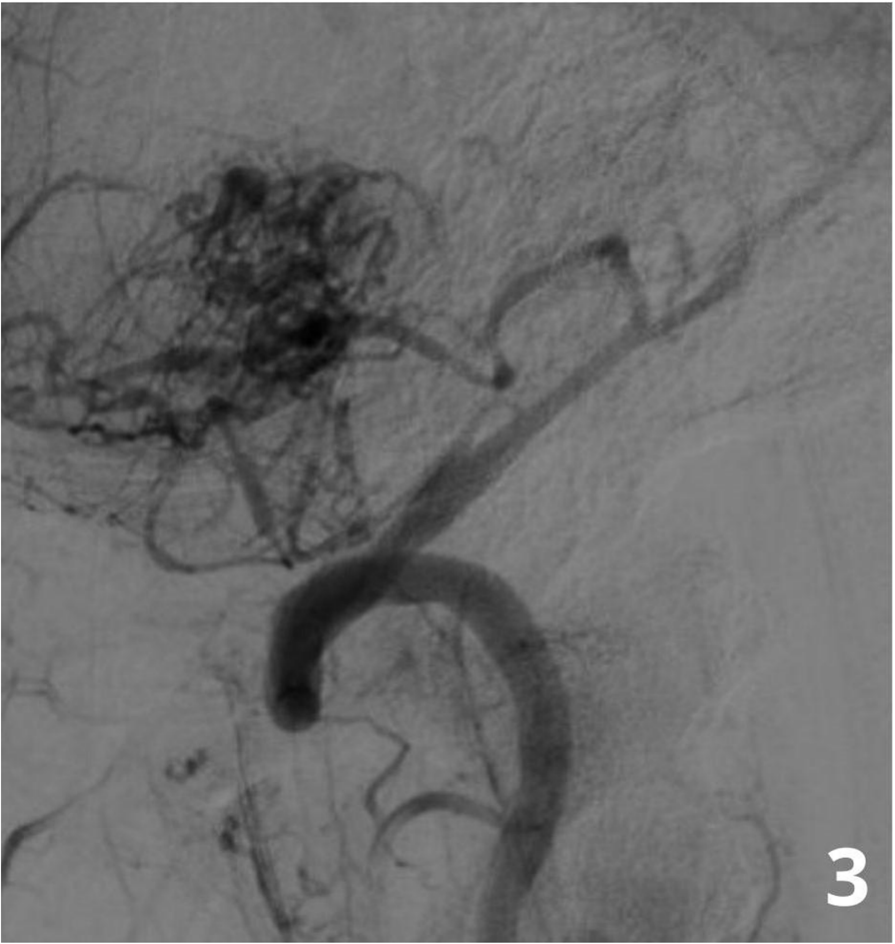
** (abstract A39).** See text for description

**Fig. 4:**
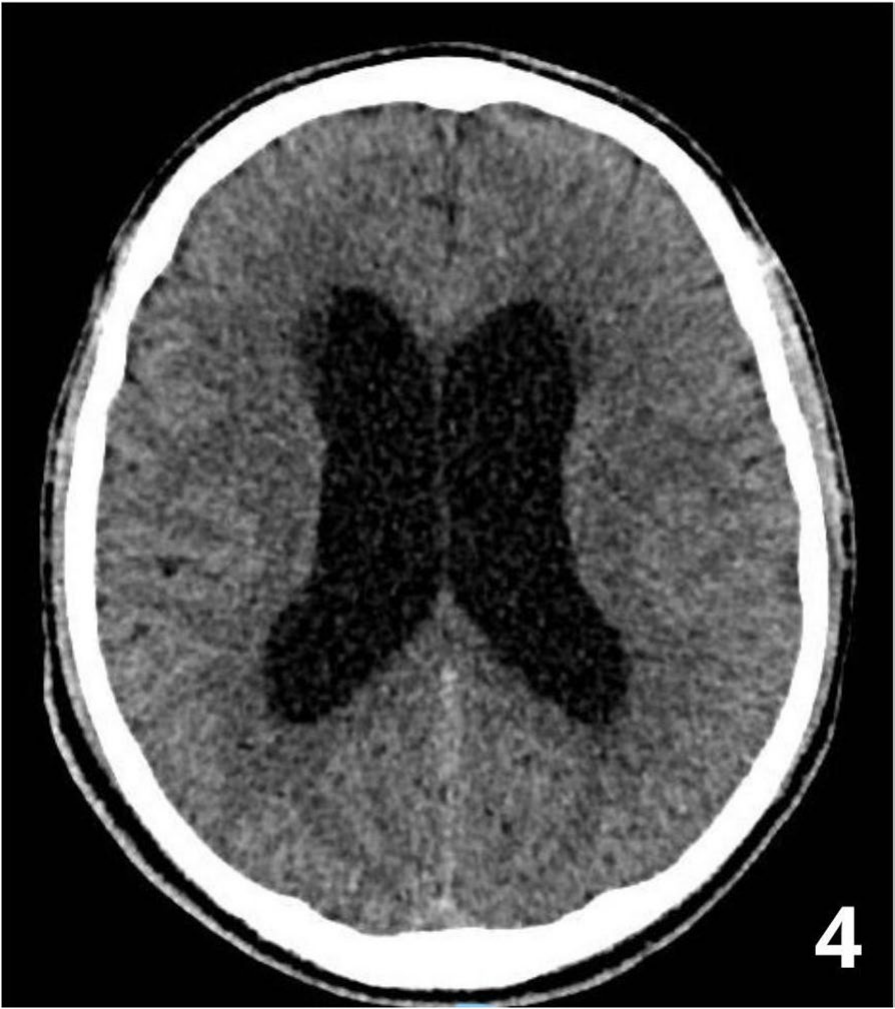
** (abstract A39).** See text for description

### A40 Cerebral hemorrhage associated with genetically based coagulopathy: neurointensive care management

#### F. Gritti, M.C. Visone, F. Di Biase, E.C. Bonagura, G. Merola, D. Di Gennaro, C. Russo

##### A.O.R.N. A. Cardarelli—1 Servizio Anestesia e Rianimazione, Naples, Italy

###### **Correspondence:** F. Gritti

*Journal of Anesthesia, Analgesia and Critical Care 2024*, **4(1):**A40

Cerebral hemorrhage is a well-known clinical problem with significant mortality and morbidity values. In the ICU of the AORN Cardarelli in Naples we treated a cerebral hemorrhage with a rarer etiology than others. A 35-year-old woman arrives in the emergency room on the fourth day postpartum: soporous, arousable by painful stimuli with localization, hyposthenia in the right side (GCS9: E4 V1 M4). She performs a total body CT scan which highlights: extensive left frontal hemorrhagic area from thrombosis of the superior sagittal sinus with venous infarction and intracranial hypertension with initial uncal herniation, associated with diffuse thromboembolism with PE (pulmonary embolism) in the left pulmonary artery, non-occluding thrombosis of the left external iliac vein and focal filling defect of the proximal lumen of the popliteal femoral vein History: first normal pregnancy. Second normal pregnancy with eutocic birth four days earlier and discharge home. The patient was intubated and underwent first a mechanical thrombectomy and subsequent decompressive craniectomy and placement of a caval filter. Our team therefore decided to request a haematological consultation: negative for ADAMS 13 for TTP (thrombotic thrombocytopenic purpura), in the absence of signs of DIC (disseminated intravascular coagulation), hypochromic microcytic anemia and moderate thrombocytopenia. On the morphological examination of the smear, there was no significant alteration of the granulocyte population and mild blood anisopoikilocytosis in the absence of schistocytes and blood fragments, for which genetic screening of coagulation was indicated. On the peripheral smear, no pathological elements (in particular the absence of schistocytes), anti PF4 negative. A thrombophilic screening was carried out, with anti-phospholipid Ab dosage (LAC, anticardiolipin, anti beta2 glycoprotein) and thrombotest (genetic screening on G20210A, factor V Leyden, PAI1, ACE, MTHFR 677) with the detection of three genetic variants:

- p.H1299R(FVR2) in heterozygosity coding for factor V Leyden;
- C677T (MTHFR 677) in heterozygosity in the MTHFR gene, a variant which, especially in homozygosity, can be associated with increased homocysteine levels in the blood and urine.
- 4G/5G in homozygosity in the PAI-1 gene currently not linked to thrombotic risk.

The patient was placed on therapy with fondaparinus 7.5 mg and solumedrol 1 g/day for 5 days, then suspended in the absence of evidence of a dysimmune thrombotic condition. On the sixth day she underwent a dilated tracheotomy with the Ciaglia Blue Rhino technique and subsequently a caval filter was placed.

After 19 days, she was transferred to Neurosurgery awake, conscious, aphasic and with right hemiplegia and finally decannulated and transferred to another rehabilitation facility—The patient expressed consent to the publication of her clinical case as per Italian laws.

**Fig. 1:**
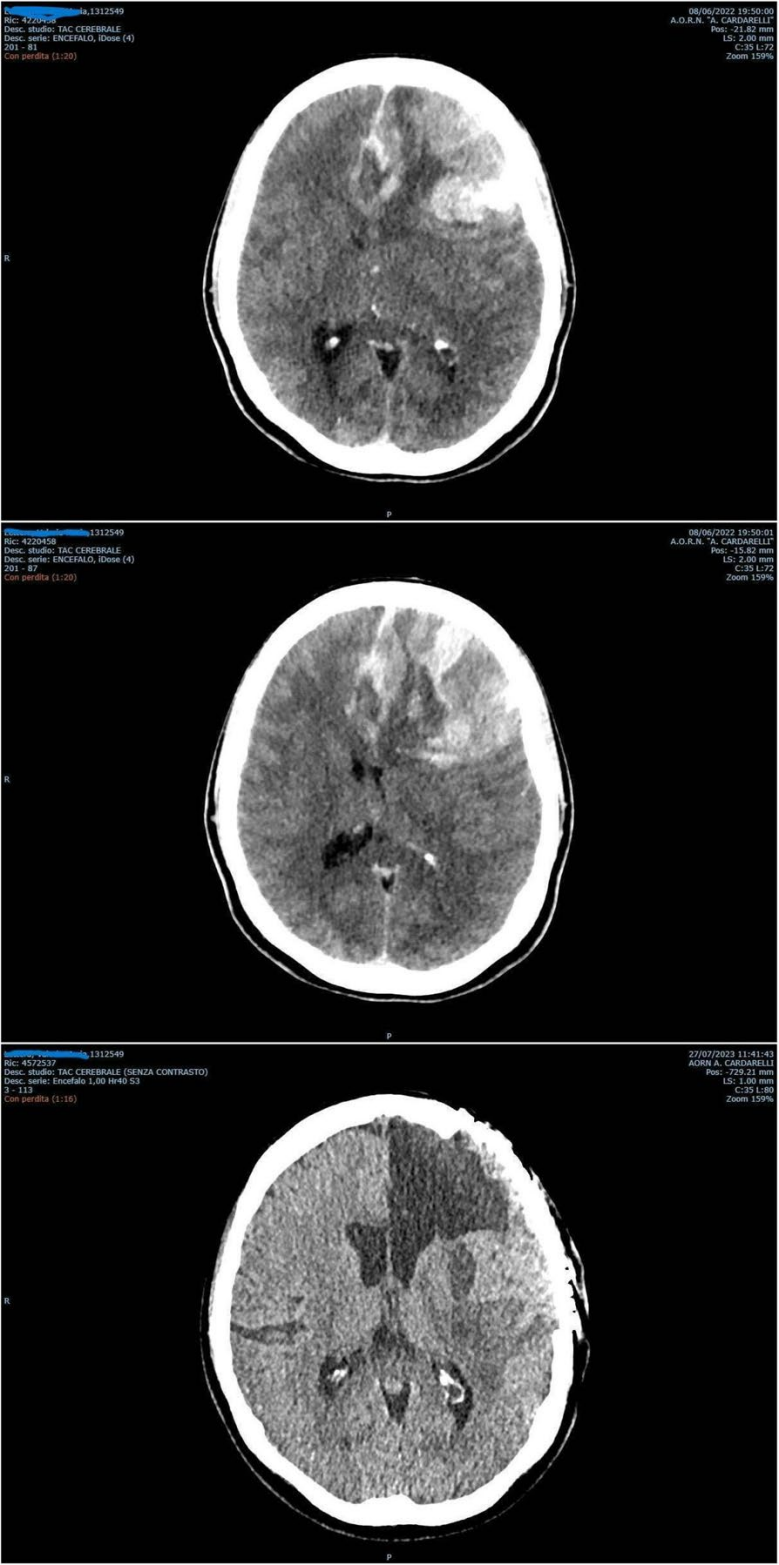
** (abstract A40).** See text for description

### A41 Intra cranial pressure threshold according to mass effect

#### D. Corbella^1^, G. Lando^1^, S. Aresi^1^, A. Viscone^1^, G. Dell'Avanzo^1^, S. Martchenko^1^, M. Aliprandi^1^, M. Di Matteo^1^, A. Lanterna^2^, F. Biroli^3^, R. Zangari^3^, M. Bonfanti^3^, M. Vascello^4^, P. Gritti^1^, L. Lorini^5^

##### ^1^NeuroTrauma Intensive Care Unit, Department of Anesthesia, Emergency and Critical Care, Azienda Socio Sanitaria Territor, Bergamo, Italy; ^2^Neurosurgery Unit, Department of Neurosciences, Azienda Socio Sanitaria Territoriale Papa Giovanni XXIII, Bergamo, Italy; ^3^FROM Fondazione Per la Ricerca Ospedale di Bergamo, Papa Giovanni XXIII Hospital, Bergamo, Italy; ^4^Department of Health Science, Clinical Psychology Unit, Azienda Socio Sanitaria Territoriale Papa Giovanni XXIII, Bergamo, Italy; ^5^CardioVascular Intensive Care Unit, Department of Anesthesia, Emergency and Critical Care, Azienda Socio Sanitaria Terri, Bergamo, Italy

###### **Correspondence:** P. Gritti

*Journal of Anesthesia, Analgesia and Critical Care 2024*, **4(1):**A41

**Background**

Twelve years ago, a seminal paper from Sorrentino et al.[1] defined 22 mmHg of Intracranial Pressure (ICP) as the best-discriminating ICP threshold between Good and Bad Outcome in traumatic brain injury (TBI) patients. Even though Sorrentino did not mean to identify a cut-off for treatment, subsequent guidelines[2, 3] pointed out that threshold as a goal of therapy.

We wander if that threshold is still meaningful in patients that underwent decompressive craniectomy or show midline shift at admission.

**Materials and Methods**

We retrospectively enrolled all consecutive adult and pediatric patients admitted to our Intensive Care Units (ICU) from January 2013 to June 2022 (Ethic Committee approval number 303/20) with a diagnosis of TBI and an ICP monitoring longer than 4 h. Informed consent was waived. We gathered demographic data, Glasgow Coma Scale, pupillary reflex, hypoxia, and hypotension, initial imaging results (Marshall scales), length of hospital and ICU stays, incidence of decompressive craniectomy, Glasgow Outcome Scale Extended (GOSE) scores one-year post-injury.

Outcome was categorized into Good Outcome (GOSE > 3) and Bad Outcome (GOSE score < 4). We collected ICP data from intraparenchymal catheters whose readings were stored in our electronic health record (sample frequency 0.0033 Hz). The time series were manually filtered and resampled on a minute-by-minute basis using linear interpolation. Time series shorter than 60 min were disregarded. For each patient, we calculated the mean ICP during the monitoring period. The optimal ICP threshold was determined using sequential chi-square methods[1], both for the entire population and for subgroups, i.e., decompressive craniectomy, Marshall grade, and dichotomized Marshall grade into Marshal without Midline shift (Marshall Grade < IV) and Marshal with Midline Shift (Marshall Grade > III).

We enrolled 263 patients. Table 1shows admission, treatment, and outcome data in the general population and in the predefined subgroup. Figure 1 represents the distribution of chi-square values across the ICP cut-offs within the predefined groups compared to the general population.

The ICP threshold was around 23–25 in the general population, as well as in patients that had midline shift at admission or underwent decompression. A clear cut-off could not be identified for patients without decompression or midline shift.

**Conclusion**

Our results confirm an ICP threshold between 22 and 25 in the general population [1] and in patients showing radiological signs of intracranial hypertension or judged in need of decompressive craniectomy. This threshold is less evident in the other subpopulations and it showed a ceiling effect around 22–25 maybe due to our local preference toward decompressive craniectomy as a rescue maneuver to control ICP.

**References**

1. Sorrentino E, Diedler J, Kasprowicz M, et al. (2012) Critical thresholds for cerebrovascular reactivity after traumatic brain injury. Neurocrit Care 16:258–266. 10.1007/s12028-011-9630-8
2. Carney N, Totten AM, O’Reilly C, et al. (2017) Guidelines for the Management of Severe Traumatic Brain Injury, Fourth Edition. Neurosurgery 80:6–15. 10.1227/NEU.0000000000001432
3. Hawryluk GWJ, Aguilera S, Buki A, et al. (2019) A management algorithm for patients with intracranial pressure monitoring: the Seattle International Severe Traumatic Brain Injury Consensus Conference (SIBICC). Intensive Care Medicine 45:. 10.1007/s00134-019-05805-9

**Fig. 1:**
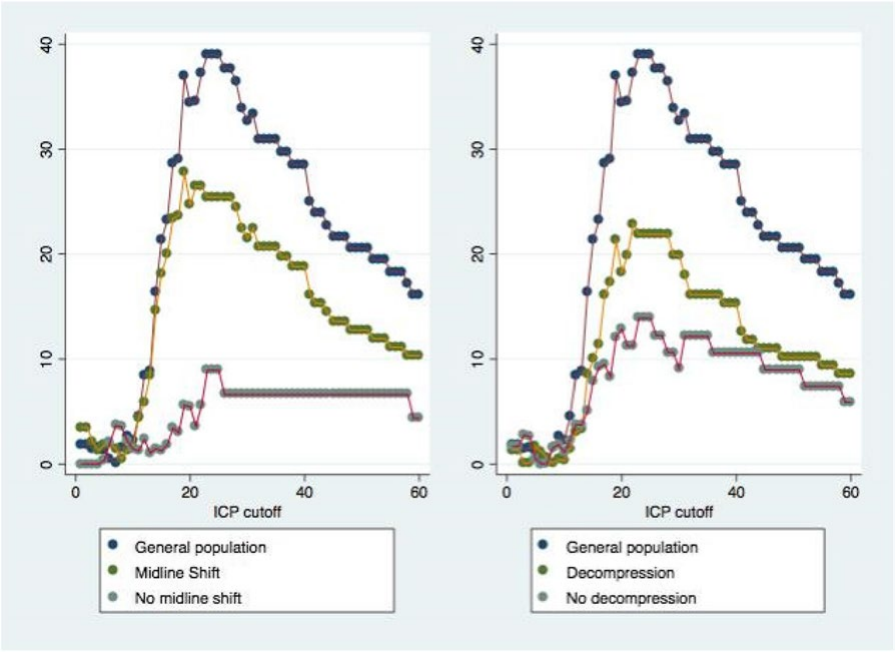
** (abstract A41).** Distribution of chi-square values across the ICP cut-offs within the predefined groups compared to the general population

**Table 1:** **(abstract A41).** Admission, treatment, and outcome data in the general population and in the predefined subgroup

Variable	General population	Patients with DC	Patients without DC	Patients with midline shift	Patients without midline shift
Patients, n	263	137	126	185	78
Age	46 (23 – 69)	47 (24 – 66)	45 (21 – 70)	54 (32 – 70)	26 (13 – 48)
Gender (male)	205 (78.0)	107 (78.1)	98 (77.8)	140 (75.7)	65 (83.3)
Pupillary response at light at admission					
Both reactive	192 (73.0)	89 (67.4)	103 (82.4)	129 (72.1)	63 (80.8)
One reactive	18 (6.8)	13 (9.9)	5 (4.0)	14 (7.8)	4 (5.1)
None reactive	47 (17.9)	30 (22.7)	17 (13.6)	36 (20.1)	11 (14.1)
Glasgow Coma Scale, at admission	7 (3 – 11)	7 (3 – 11)	7 (4 – 12)	7 (3 – 13)	6 (3 – 8)
Hypoxia at the crash scene	38 (14.5)	21 (15.7)	17 (13.5)	23 (12.6)	15 (19.2)
Hypotension at the crash scene	41 (15.8)	22 (16.4)	19 (15.1)	29 (15.9)	12 (15.4)
Length of stay					
ICU	19.0 (8.0 – 31.0)	21 (8 – 32)	18 (8 – 29)	19 (7 – 13)	22 (9 – 34)
Hospital	28.0 (13.0 – 43.0)	33 (11 – 47)	25 (15 – 38)	25 (10 – 42)	31 (19 – 45)
Glasgow Outcome Scale Extended at one year					
Dead in hospital	68 (25.9)	60 (32.4)	8 (10.3)	42 (30.7)	26 (20.6)
Dead at one year	18 (6.8)	15 (11.1)	3 (3.9)	10 (7.3)	8 (6.4)
Vegetative State	16 (6.1)	12 (6.5)	4 (5.1)	12 (8.8)	4 (3.2)
Lower Severe Disability	33 (12.6)	23 (12.4)	10 (12.8)	19 (13.9)	14 (11.1)
Upper Severe Disability	23 (8.8)	18 (9.7)	5 (6.4)	14 (10.2)	9 (7.1)
Lower Moderate Disability	16 (6.1)	10 (5.4)	6 (7.7)	7 (5.1)	9 (7.1)
Upper Moderate Disability	29 (11.0)	14 (7.6)	15 (19.2)	15 (11.0)	14 (11.1)
Lower Good Recovery	37 (14.1)	23 (12.4)	14 (18.0)	12 (8.8)	25 (19.8)
Upper Good Recovery	23 (8.8)	10 (5.4)	13 (16.7)	6 (4.4)	17 (13.5)

We presented continuous variables as median (interquartile range) and categorical variables as numbers and percentages

### A42 Impact of traumatic axonal injury on long-lasting neurobehavioral disorders

#### M.G. Vascello^1^, S. Pizzighello^2^, R. Zangari^3^, C. Agostinis^4^, F. Biroli^3^, D. Corbella^5^, L.A. Lanterna^6^, M.L. Dello Russo^5^, S. Milani^5^, D. Salmi^5^, T. Togni^5^, F. Micheli^5^, G. Cavalleri^5^, L. Urbaz^5^, M. Spada^1^, S. Galeri^7^, F.L. Lorini^5^, S. Gerevini^4^, P. Gritti^5^

##### ^1^Department of Health Science, Clinical Psychology Unit, Papa Giovanni XXIII Hospital, Bergamo, Italy; ^2^Neurorehabilitation Unit, Scientific Institute IRCCS E. Medea, Conegliano-Pieve di Soligo, Italy; ^3^FROM Research Foundation, ASST Papa Giovanni XXIII Hospital, Bergamo, Italy; ^4^Department of Neuroradiology, ASST Papa Giovanni XXIII Hospital, Bergamo, Italy; ^5^Department of Anesthesia and Critical Care Medicine, ASST Papa Giovanni XXIII Hospital, Bergamo, Italy; ^6^Department of Neuroscience and Surgery of the Nervous System, ASST Papa Giovanni XXIII, Bergamo, Italy; ^7^Department of Neuroscience, Rehabilitation Unit, ASST Papa Giovanni XXIII Hospital, Bergamo, Italy

###### **Correspondence:** P. Gritti

*Journal of Anesthesia, Analgesia and Critical Care 2024*, **4(1):**A42

**Background**

The most common neurobehavioral symptoms observed in patients with traumatic brain injury (TBI) during the chronic phase include lack of initiative, impulsivity, irritability, socially inappropriate behavior, and self-centeredness. These symptoms impede TBI patients from establishing and maintaining close personal relationships, friendships, or relationships with work colleagues and hinder their abilities to return to school or work[1, 2]. In literature, demographics (i.e., gender, age, and education) and clinical variables (i.e., Post-traumatic Amnesia duration and Glasgow Coma Scale severity) have been explored as predictors of NBDs.

Few studies provided evidence of a significant relationship between brain lesions and neurobehavioural disorders in chronic TBI[3], particularly concerning non-specific lesions, such as traumatic axonal injury (TAI)[4].

This study explored how different variables, including the occurrence of TAI, could be associated with the development of Neurobehavioural Disorders (NBDs).

**Material and Methods**

We enrolled all consecutive adult patients admitted to the NeuroCritical Care Unit of the Hospital Papa Giovanni XXIII with a diagnosis of TBI and Post-Traumatic Amnesia (PTA). We excluded patients with a pre-traumatic history of developmental, neurological, or psychiatric disorders or a history of alcohol or drug dependency. The Hospital Ethics Committee approved the study (date: November 13, 2016; register number: 291/16), and we collected informed consent.

All these patients underwent a neurobehavioural assessment 12 months post-TBI, including the Head Injury Behavioural Scale (HIBS)[5] administered to their caregivers.

We divided the sample into TAI (n = 28) and non-TAI (n = 26). The diagnosis of TAI was based on a brain CT scan and was defined as the presence of small non-expansive hemorrhagic lesions in the areas of connection between white and gray matter with more than three foci. Multiple linear regression was used to test if sex, age, education, PTA, GCS (i.e., common TBI outcome predictors), and the presence of TAI significantly predicted the occurrence of neurobehavioural disorders as measured by the HIBS total score.

**Results**

We included 54 patients (12 F, 42 M; mean age 46.1, scholarity 10.4 years), 28 TAI, and 26 non-TAI patients. Based on the Glasgow Coma Scale (GCS), they were classified as follows: 10 patients had a complicated mild TBI (i.e., mild with evidence of intracranial bleed or lesion), 11 had a moderate TBI, and 33 had a severe TBI. The most frequent NBDs observed by the caregivers were anger/ difficulty controlling temper, impulsivity, and irritability. The best predictors of these disturbances were education, post-traumatic amnesia (PTA), and TAI, which explained about one-third of the variability of NBDs (adjusted R2 = 0.35).

**Conclusions**

We should closely monitor patients with lower education, longer PTA, and TAI because they will likely develop long-lasting NBDs whose severity can be reduced when early and intensively addressed.

### A43 Rapid escalation in control of no convulsive status epilecticus: a case report of an autoimmune encephalitis

#### M. Albore, A. Vivaldo, E. Ravera, I. Pabon, G. Carlidi, A. Della Selva

##### ASL CN2, Alba, Italy

###### **Correspondence:** M. Albore

*Journal of Anesthesia, Analgesia and Critical Care 2024*, **4(1):**A43

**Rationale**

Refractory status epilepticus is not an uncommon cause of admission to the intensive care unit (ICU). The diagnosis is especially difficult in cases of non-convulsive status epilepticus (NCSE) in which the symptoms can sometimes appear subtle. The etiology often remains unknown despite the use of the most refined investigations.

We report a case of a 55-year-old female patient with hypothyroidism as her only comorbidity. The patient arrived to the emergency department ED for fever and headache with neck stiffness ongoing for approximately 24 h, GCS on arrival was 14 (E4 V4 M6). A non-contrast brain CT was performed, after excluding a subarachnoid hemorrhage, a lumbar puncture (LP) was performed to rule out a meningoencephalitis. The cerebrospinal fluid (CSF) revealed proteinorachia with presence of white blood cells (WBC), erythrocytes and glucose within normal range, in the meantime empiric antibiotic therapy with Ceftriaxone was started. Due to persistent symptoms an electroencephalogram (EEG) was performed with evidence of epileptiform graphoelements in the fronto-temporal regions bilaterally with initial response to the administration of intravenous Diazepam but rapid return to status epilepticus and deterioration of the state of consciousness, with a hig clinical suspicion of NCSE, therapy with Levetiracetam is started without benefit on the EEG trace.

Admission to the Intensive Care Unit was necessary to begin sedation with Propofol which however did not have the result of interrupting the NCSE. A third line agent (Midazolam) in continuous intravenous infusion was started aided by continuous EEG monitoring until the point of burst suppression was met [Image 1]. The interruption of the benzodiazepine infusion resulted in the resumption of epileptic activity, at this point the antiepileptic therapy was potentiated with Lacosamide from the second day and Perampanel from the third day of hospitalization.

Therapy with Acyclovir and dexamethasone was started. A new lumbar puncture was performed with CSF exam still showing proteinorachia and high antibody titer[Image 2]. With the suspicion of autoimmune encephalitis the steroid therapy was potentiated from the fourth day and a five day immunoglobulin cycle was administered at the end of which a definitive improvement of the epileptogenic graphoelements was observed. The clinical suspicion was confirmed by positivity for antibodies against extractable nuclear antigens (ENA)[Image 3]. The sedation was then gradually weanes and stopped, the patient slowly regained consciousness and was extubated. After being transferred to the neurology ward and further observation, she was discharged to a rehabilitation center without any neurological sequelae.

**Conclusions**

NCSE is a clinical condition, sometimes subtle, which if not diagnosed and treated promptly and aggressively, as highlighted by the literature, can lead to serious neurological sequelae. Rapid treatment from the first hours aimed at controlling the seizures up to burst suppression is necessary to avoid a high level of morbility and mortality.

Informed consent was obtained.

**Fig. 1:**
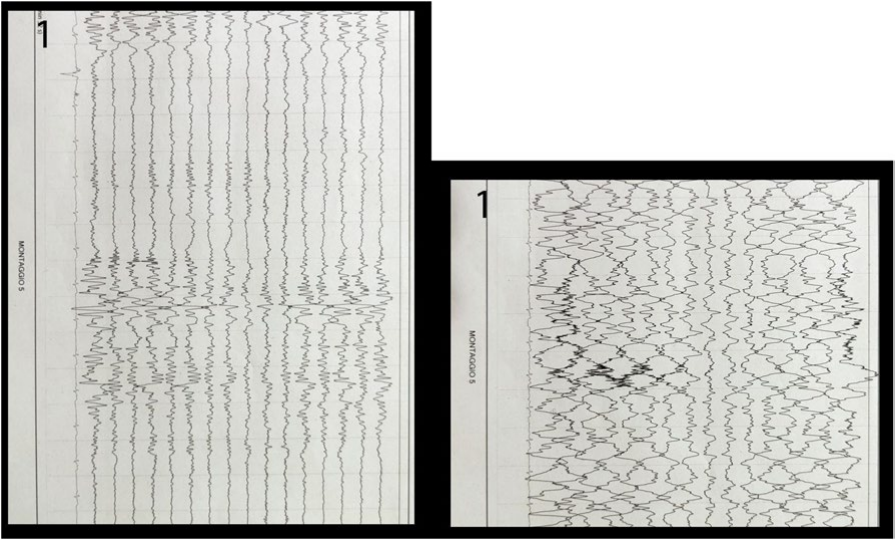
** (abstract A43).** See text for description

**Fig. 2:**
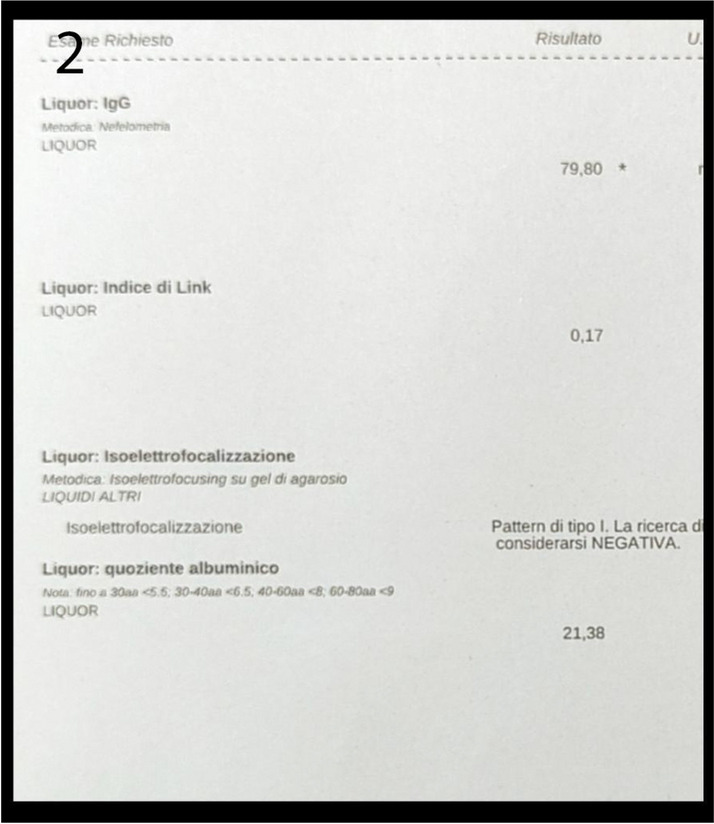
** (abstract A43).** See text for description

**Fig. 3:**
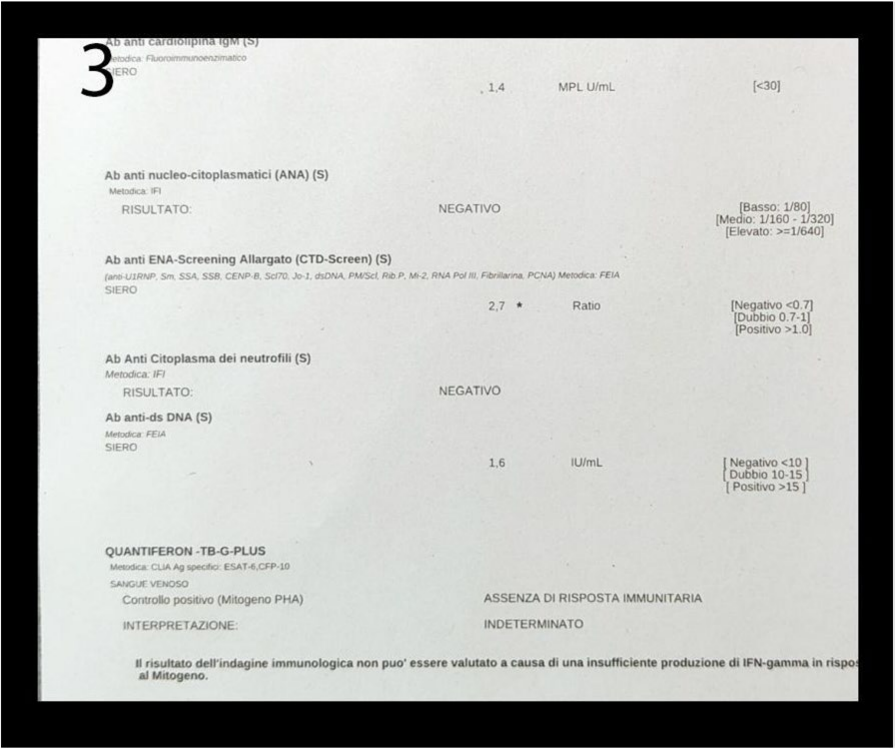
** (abstract A43).** See text for description

## Infections and sepsis

### A44 Use of CytoSorb® as a therapeutic option in a critically ill patient with severe acute respiratory distress syndrome caused by influenza A (H1N1) pneumonia: case report

#### M. Bova^1^, L. Tombolini^2^, R. Russo^1^, A. Carbone^1^, F. Macri^1^, N. Zarrillo^1^

##### ^1^ASL Caserta -PO San Rocco-, Sessa Aurunca-Caserta, Italy; ^2^Universita' Politecnica Delle Marche, Ancona, Italy

###### **Correspondence:** A. Carbone

*Journal of Anesthesia, Analgesia and Critical Care 2024*, **4(1):**A44

Acute respiratory distress syndrome (ARDS) is an acute inflammatory lung process, which leads to protein-rich nonhydrostatic pulmonary edema, refractory hypoxemia, and lung 'stiffness'. In most cases the death is associated more with the contemporaneal multiple organ dysfunction syndrome (MODS) than to the respiratory failure. CytoSorb® is an extracorporeal cytokine adsorber used to reduce circulating mediators leading to uncontrolled systemic inflammation, organ failure, and death in many life-threatening illnesses.

Case Description: We present the case of a 42-year-old female patient, in good health before admission, who was admitted to our Intensive Care Unit with MODS associated with massive bilateral pneumonia caused by H1N1 influenza A. Fever up to 39 °C was present from 4 days at the admission, due to severe hypoxemia and altered state of consciousness on admission, the patient was immediately intubated in analgosedation and mechanically ventilated using a lung-protective ventilation at a FiO2 of 100% with a corresponding SpO2 of 82%. Cycles of prone position were performed since the first day of hospitalization. Due to a severe hemodynamic instability, the rate of norepinephrine had to be increased up to 1 y/kg/min associated to empressin up to 0,03 IU. She also had signs of both systemic inflammation as demonstrated by elevated values of C-Reactive-Protein (396 mg/L), procalcitonin (4,5 ng/ml) and of organ dysfunction, including leukocytopenia (0.800 × 103/uL), low platelets (27,0 × 103/uL), anuria and elevated total bilirubin (4,06 mg/dL). Despite all therapeutic and supportive measures, broad-spectrum empirical antimicrobial therapy (meropenem and ceftaroline), antiviral therapy, lung-protective ventilation, prone position, the patient's condition progressively worsened. In the second day of hospitalization, due to the progressive hemodynamic instability (higher norepinephrine demand) and uncontrolled inflammatory response, a CytoSorb® adsorber was installed inserted into the CRRT circuit in CVVHDF mode with local anticoagulation with citrate. After five cycles of CytoSorb®, the patient became hemodynamically stable, so norepinephrine and empressin requirements decreased and were discontinued one day later. The ventilation parameters and lung function gradually improved, and the patient was eventually discharged to rehab unit after three months from the admission.

**Discussion and conclusion**

In our patient the combined treatment with standard therapy, CRRT, and CytoSorb® hemoadsorption was associated with rapid hemodynamic stabilization and the control of the inflammatory response. It is likely that this effect can be ascribed to the decrease of inflammatory mediators associated with hemoadsorption; unfortunately, in our hospital their measurement is not (yet) feasible, then we cannot demonstrate the change in cytokine levels before and after treatment. We noticed significant decrease in inflammation markers with gradual improvement in patient ventilation parameters and lung function, with a hemodynamic stability as evidenced by decreased vasopressor requirements.

Laboratory findings and trends of norepinephrine and empressin, before and after CytoSorb® procedure, are presented in Table 1 and Table 2 and Figure.

The authors certify that they have obtained all appropriate patient consent forms.

**Table 1:** **(abstract A44).** See text for description

Laboratory parameters	PreCytoSorb®therapy	PostCytoSorb®therapy
Leukocytes	0.800 × 10^3^/μl	23,800 × 10^3^/μl
Platelets	27.0 × 10^3^/μl	206 × 10^3^/μl
Total bilirubin	4.06 mg/dL	1,49 mg/dL
Lactate	8,7 mmol/L	1,3 mmol/L

**Table 2:** **(abstract A44).** See text for description

	I			III			V	VI	
NOREPINEPHRINE	16	15	10	5	3	2	0	0	0
EMPRESSIN	3,5	3,5	2	2	1,5	1,5	1	0,5	0

**Fig. 1:**
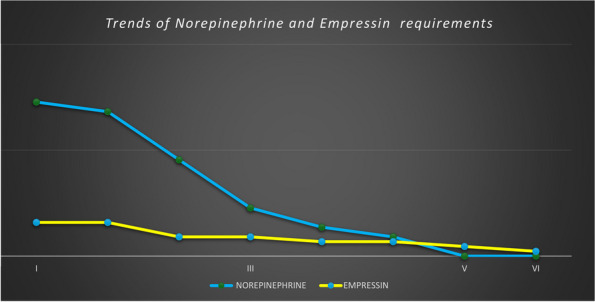
** (abstract A44).** See text for description

### A45 Presentation of angioinvasive Influenza-Associated Pulmonary Aspergillosis (IAPA) in an immunocompetent patient with severe central nervous system involvement

#### A. vivaldo, I.M. Pabon, M. Albore, C. Bertola, G. Carlidi, E. Ravera, A. Della Selva

##### Ospedale Pietro e Michele Ferrero, Verduno, Italy

###### **Correspondence:** A. vivaldo

*Journal of Anesthesia, Analgesia and Critical Care 2024*, **4(1):**A45

**Background**

Influenza-associated pulmonary aspergillosis (IAPA) is increasingly recognized as a co-infection in ICU patients with influenza, contributing to complications and worsened outcomes. IAPA primarily affects immunocompromised individuals and often presents with atypical clinical and radiological features, posing challenges for diagnosis and treatment.

**Case report**

We report the clinical case of a 67-year-old woman with bipolar disorder and no known immunocompromised status, who initially presented with respiratory symptoms and worsening neurological function. Following an initial diagnosis of interstitial pneumonia positive for influenza A positive on nasopharyngeal swab without neurological involvement (negative findings on CT brain and lumbar puncture), the patient's clinical condition quickly progressed to septic shock, necessitating admission to intensive care. The patient developed severe respiratory failure, cardiovascular instability, and acute kidney injury (AKI) requiring continuous renal replacement therapy.

Further medical investigations revealed positive galactomannan, both on serum and bronchoalveolar lavage fluid, in addition to pseudomembrane on bronchoscopy (Image 1) and the presence of Aspergillus Fumigatus in tracheal aspirate and bronchoalveolar lavage cultures.

Neurologically, the patient presented with a persistent comatose state following a non-convulsive epileptic episode diagnosed by electroencephalogram (EEG). Upon resolution of the acute phase, daily evaluation of neurological status revealed a persistent coma status with GCS 4 in the absence of epileptic activity on repeated EEGs.

Radiologic imaging revealed multiple pulmonary cavitation lesions (Image 2) and cerebral findings suggestive of diffuse septic embolization (Image 3).

Specific therapy for aspergillosis, initially started with voriconazole later shift to isavuconazole due to hepatic toxicity, along with amphotericin B, was administered unsuccessfully.

Throughout the hospitalization, the clinical course was complicated by severe thrombocytopenia and multiple episodes of high-frequency atrial fibrillation, contributing to the deterioration of multi-organ failure, ultimately resulting in death on day 34 of hospitalization.

**Conclusion**

IAPA has emerged as a severe complication of influenza, particularly in ICU patients, and can occur even in individuals considered to be at lower risk. This condition can lead to worsened patient outcomes and death. While antifungal therapies have improved, treatment options remain limited, and outcomes for these patients are still unsatisfactory.

Informed consent was obtained from patient’s next of kin.

**Fig. 1:**
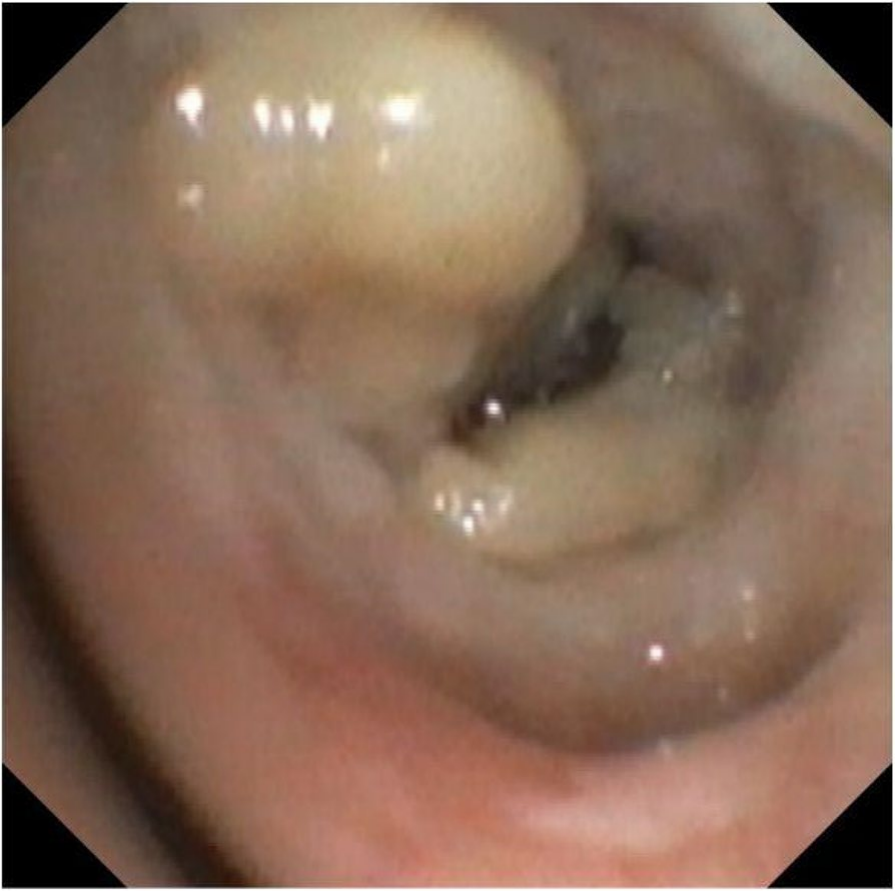
** (abstract A45).** Pseudomembrane at bronchoscopy (collected at 3rd day of ICU recovery)

**Fig. 2:**
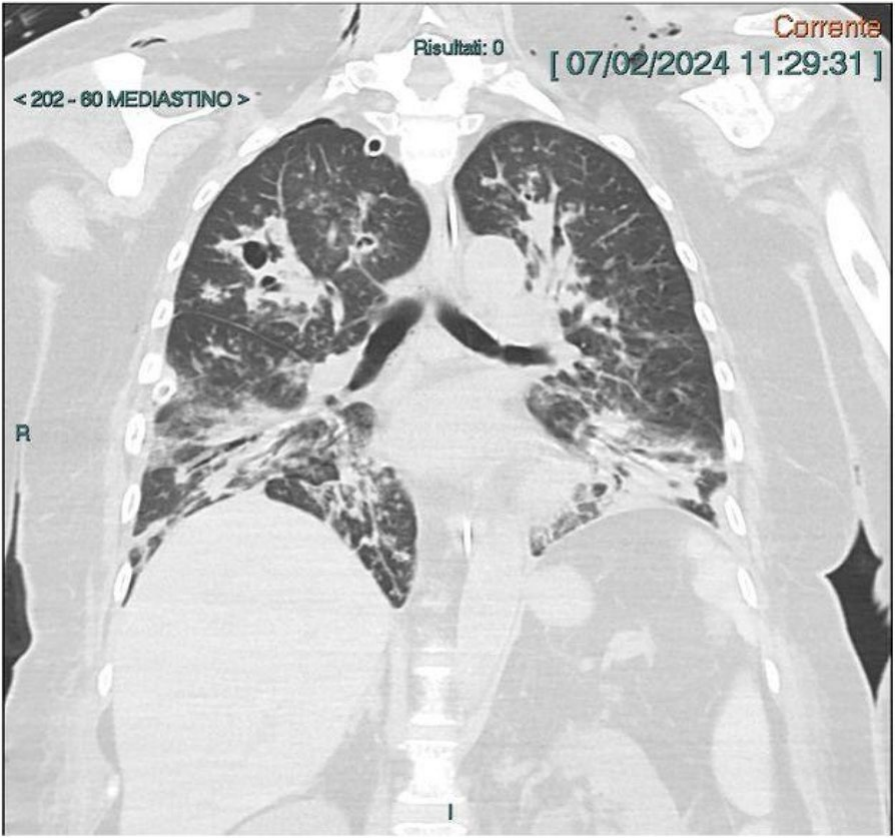
** (abstract A45).** Cavitation in an area of pulmonary consolidation at CT chest coronal view

**Fig. 3:**
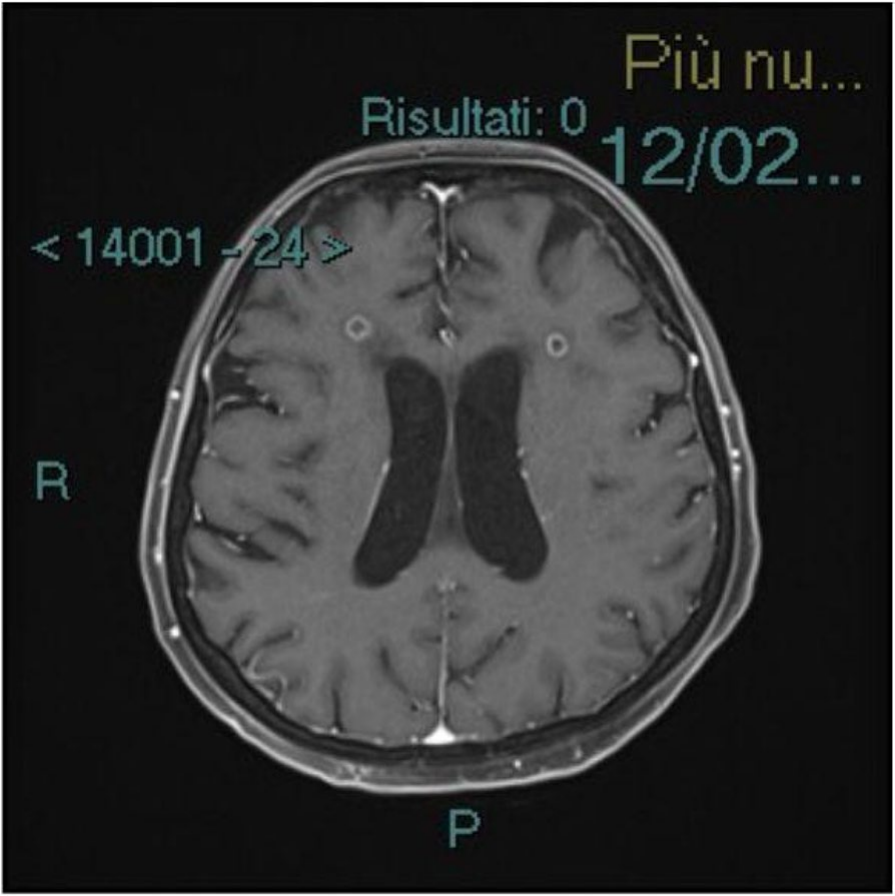
** (abstract A45).** Multiple encephalic lesions suggestive of septic embolization at brain MRI

### A46 Clinical experience with an endotoxin adsorption device in septic patient with acute myeloid leukemia case report

#### K. Valentini^1^, F. Maceri^1^, G. Giannoni^1^, E. Benedetti^2^, M. Picchi^1^, L. De Simone^1^

##### ^1^Azienda Ospedaliero-Universitaria Pisana -Spedali Riuniti Santa Chiara UO Anestesia e Rianimazione, Pisa, Italy; ^2^Azienda Ospedaliero-Universitaria Pisana -Spedali Riuniti Santa Chiara UO Ematologia DAI Oncologico, Pisa, Italy

###### **Correspondence:** K. Valentini

*Journal of Anesthesia, Analgesia and Critical Care 2024*, **4(1):**A46

**Background**

Sepsis is an acute, rapidly evolving syndrome burdened by high mortality.

In recent decades, steady advances in acute myeloid leukemia (AML) diagnostics, treatment regimens, and patient risk stratification protocols have led to increased survival rates. Intensive treatment regimens are used to improve survival rates, which can lead to severe complications and intensive care unit ICU admission. In these patients, there is a significant increase in Gram-negative bacteremia as a cause of septic status. In sepsis caused by Gram-negative bacteria, circulating endotoxin is one of the factors in the septic cascade, which can progress to multiorgan dysfunction syndrome (MODS) and death.

**Case Report**

Male patient, age 57 years old, undergoing allogeneic marrow transplantation, previous, for AML, arrives at our ICU in septic shock, for pan-ileitis in the setting of intestinal GVHD, soporific, dehydrated, with profuse diarrhea and abdominal algic symptomatology, hemodynamics supported by norepinephrine, antibiotic therapy for presence of KPC-CTX-M, Staphylococcus saprophyticus, Staphylococcus Haemolyticus, Escherichia Coli (ESBL).

Informed consent was obtained from the patient’s close relatives upon admission to the ICU.

Patients with hematologic malignancies complicated by septic shock have a very high mortality rate.

In our experience, hemoperfusion therapy with lipopolysaccharide selective sorbent (LPS) and renal replacement therapy (CRRT) in addition to conventional therapy may be a valuable strategy to improve outcome in this type of patients.

Hemoperfusion treatment during CRRT, with citrate-calcium anticoagulation with selective sorbent for Lipopolysaccharide was administered, at the patient's admission to the ICU, ascertained sepsis from multi-resistant gram-negative bacteria.

The patient received two sessions of hemadsorption with sorbent for Lipopolysaccharide during CCRT as renal support. At the end of the sessions, a reduction in procalcitonin (PCT) a marked clinical improvement was observed, with resolution of soporific state and abdominal pain, resumption of consciousness, discontinuation of norepinephrine (NE), restoration of hemodynamics and respiratory function with increase in PaO2/FiO2 ratio, resumption of os feeding. The patient's clinical condition, hemodynamic parameters, and blood chemistry values were monitored until discharge from the intensive care unit (ICU) No treatment-related adverse events occurred with sorbent for Lipopolysaccharide during renal replacement therapy. The ICU stay was for 10 days.

**Conclusion**

According to the guidelines, hemoperfusion therapies are not recommended in the management of sepsis. In our experience in severely immunocompromised patients with confirmed or suspected infectious status, selective, LPS, sorbent hemoperfusion techniques are a valuable adjunct to antibiotic therapy.

Informed consent was obtained.

### A47 Levosimendan vs dobutamine in the management of cardiac dysfunction in patients with septic shock

#### M. Toma, E. Epifani, M. Madia, D. Puscio, G. Pulito

##### Dipartimento Anestesia, Rianimazione e Terapia del dolore, P.O. Vito Fazzi, Lecce, Lecce, Italy

###### **Correspondence:** M. Toma

*Journal of Anesthesia, Analgesia and Critical Care 2024*, **4(1):**A47

On April 20, 2024 a patient, coming from the S.O. of Urology, where she had undergone placement of a left ureteral stent for ureterohydronephrosis, she was admitted to intensive care for a serious case of septic shock (SOFA SCORE 16). The patient's hemodynamics immediately appeared difficult to manage: despite the continuous infusion of Norepinephrine 0.6 ɣ/kg/min and Dobutamine 5 ɣ/kg/min and fluid challeng, a hypotensive picture persisted important supported by a global hypokinesis of the RV (EF 35%) on the echocardiogram with dilated right sections with hypokinetic RV free wall. HemoSphere hemodynamic monitoring was set up (PVC 14, SVRI 2485, SVV 6, dP/dT 600, PPV 6, SVI 18). Endotoxinemia assessed with Quantitative Test endotoxin activity assay (0.94), hemoperfusion began with Toramixin and subsequent initiation of CRRT. The samples taken on venous and arterial blood confirmed the importance of the septic picture (WBC 21000 × 10^3/ųl, PCT > 100 mg/ml, Lactates 15 mmol/l. Empirical antibiotic therapy with Piperacillin/Tazob was set by the infectious disease specialist ctam 4.5 g × 3/day and Fosfomycin 4 g × 4/day, promptly modified given the severity of the clinical picture with Cefatazidime/Avibactam 1.25gr × 3/day.

Despite the extensive care, the haemodynamic picture did not tend to improve in the following days. The appearance of a picture of atrial fibrillation with a high ventricular response, treated with intravenous esmolol, confirmed the severity of the cardiovascular conditions. Yet another echocardiographic check confirmed the persistence of LV hypokinesia (EF 35%). On the third day he decided to suspend dobutamine and start Levosimendan 0.2ɣ/kg/min. In the echocardiographic control following treatment with Sindax, an improvement in the hemodynamic picture was highlighted with suspension of norepinephrine and optimization of global contractility (EF 65%).

With the improvement of the septic condition, the patient was weaned from VM and, after a period in HFNC, weaned from any ventilatory support. The patient was discharged to urology on April 30, 2024, awake and cooperative, in RS with VentiMask FiO2 31%, valid unsupported hemodynamics and regular unstimulated diuresis.

Informed consent was obtained.

### A49 Sepsis after cardiac surgery: preliminary analysis of cytokines gene expression

#### R.P. Radice^1^, G. Martelli^1^, M. D'amora^2^, P. Dambruoso^3^, D. Paparella^3^, R. Mandarano^4^, G. Olivo^4^, M. Scolaro^5^, D. Sarubbi^6^, A. Strumia^6^, M. Calabrese^7^, A. Scarpigliati^7^, F. Greco^8^, M. Nardi^8^, S. Beccaria^9^, A. Costamagna^10^, L. Brazzi^10^, P. Raimondo^11^, G. Paternoster^2^

##### ^1^Università degli studi della Basilicata, Potenza, Italy; ^2^AOR San Carlo, Potenza, Italy; ^3^Ospedale Santa Maria, Bari, Italy; ^4^Cardioanestesia Careggi, Firenze, Italy; ^5^Ospedale del cuore Gaetano Pasquerucci, Massa, Italy; ^6^Policlinico campus Biomedico, Roma, Italy; ^7^Policlinico Gemelli, Roma, Italy; ^8^Casa Cura Sofferenza, San Giovanni Rotondo, Italy; ^9^A.O Ordine Mauriziano, Torino, Italy; ^10^Ospedale Molinette, Torino, Italy; ^11^Policlinico Bari, Bari, Italy

###### **Correspondence:** G. Paternoster

*Journal of Anesthesia, Analgesia and Critical Care 2024*, **4(1):**A49

According to SSC Sepsis is defined as “life-threatening organ dysfunction caused by a dysregulated host response to infection”.Sepsis remains one of the leading causes of morbidity and mortality (17–65%) worldwide and it still remains a challenge to be defined and for which an appropriate cure is desired. Different studies have been conducted on genes coding for inflammatory cytokines whose could predispose to the development of sepsis [e.g., IL-10 and PD1]. This multicentric observational prospective study aims to evaluate the genetic expression kinetics of two molecules involved in the inflammatory process, IL10 and PD1, to search for a possible molecular marker predictive of the development of sepsis. 162 patients scheduled for planned cardiac surgery were enrolled in the study. For each patient 4 blood samples have been collected at 4 different time points. Patients were defined as septic according to SSC guidelines. From each blood sample RNA was extracted and used for a qPCR. 162 patients were enrolled (100 M and 62F), 25 (15%) developed sepsis (15 M and 10F). The results show how the CBP time was longer in septic patients (143 Vs. 105 means in minutes) as the Clamping time (89.6 Vs 76.29 mim). The expression of IL10 highlight how 30 min after the start of the intervention, septic patients showed much lower levels of IL10 expression (p < 0.05). This result, however, is reversed upon entry into the ICU. The same results are confirmed by the expression of PD1, which in septic patients appears to be totally deactivated (p < 0.05). This expression kinetics demonstrates how patients who developed sepsis show a dysregulation of the immune response, which leads to decompensation of the immune system, which is thus unable to respond adequately. These data suggest how CBP and Clamping time influence more patients genetically predisposed for the sepsi.

Informed consent to publication was obtained.

### A50 MDR infections during vv-ecmo in severe ards patients before and during pandemic. a single center cohort study

#### L. Pistidda^1^, D. Pasero^1^, A.M. Muretti^2^, C. Fanelli^1^, A. Puggioni^2^, E. Virdis^2^, G.P. Branca^2^, D. Deffenu^2^, L. Solinas^3^, M. Vidili^3^, F. Mulas^2^, P. Terragni^1^

##### ^1^University of Sassari, Medicine, Surgery and Pharmacy Department, Sassari, Italy; ^2^University Hospital of Sassari, Specialistic Surgery Department, Sassari, Italy; ^3^University Hospital of Sassari, Emergency Department, Sassari, Italy

###### **Correspondence:** L. Pistidda

*Journal of Anesthesia, Analgesia and Critical Care 2024*, **4(1):**A50

**Introduction**

ECMO patients are more susceptible of Multi-Drug resistant (MDR) infection and have potential higher risk of worst outcomes due to comorbidities, immune suppression, frequent need for invasive life support procedures, use of immunomodulators (steroids), extracorporeal support and concomitant needs for organs support 1,2.

Moreover COVID-19 patients requiring mechanical ventilation (MV) have prolonged length of stay in ICU, greater exposure to broad-spectrum antibiotics secondary to HAIs most of the time caused by Multi-Drug resistant (MDR) bacteria(32–50%)3.

**Objectives**

Primary endpoint: incidence of MDR infections in severe ARDS patients who needed a VV-ECMO support. Secondary endpoint: main risk factors associated with MDR infections.

**Methods**

A single center cohort study was conducted in the ICU of the University Hospital of Sassari. The study was approved by our Institution’s Ethics’ Committee, all methods and informed consent were performed in accordance with the relevant guidelines and regulations. Inclusion criteria: patients with severe ARDS and refractory hypoxemia who received VV-ECMO support were included in the study. Qualitative data were summarized with absolute and relative (percentage)frequencies. Median and interquartile range (IQR) were used for quantitative ones; p-value lower than 0.05 was considered statistically significant. Univariable and multivariable analysis were performed to evaluate risk factors for MDR infections. STATA14software was used for statistical computations.

**Results**

From January 2017 to March 2024, 24 consecutive patients were included in the study. Patients’ characteristics were reported in Table 1. Overall, 37.5% received ECMO onsite by our team and then referred to our Regional Center.

Patients affected by ARDS COVID-19related, received a longer duration of NIV and invasive mechanical ventilation compared to ARDS patients without COVID-19 before ECMO support started (Table 1).

On admission all patients were screened for CPE and any resulted colonized with MDR bacteria before ECMO treatment. We observed an overall number of 22 MDR infections on 18 (75%) patients. The most frequent isolation was K. pneumoniae (45.8%), followed by MRSE (20.8%), A. Baumannii (16.7%), P.Aeruginosa (12.5%) and E. Coli (4.17%). Most of the isolations were from lower respiratory samples, BAL (70.8%).

At univariable (OR 1.68, 95% CI 1.02–2.78) and multivariable (OR 1.79, 95% CI 1.02–3.11) analysis the only risk factor that correlated with MDR infections was VV-ECMO duration.

Overall ICU mortality at 60 days was 45.8%. Among these patients, 81% had a MDR infection and 36% received VV-ECMO due to COVID-19.

**Conclusions**

Our study confirms that Hospital-Acquired Infections (HAIs) are common complications in VV-ECMO patients and that duration of extracorporeal mechanical support is associated with increased risk of MDR infections. Further evidence from high-quality prospective studies are warranted to better guide clinicians during the decision-making process.

**References**

1. Nosocomial Infections During Extracorporeal Membrane Oxygenation: Incidence, Etiology, and Impact on Patients’ Outcome – Grasselli et al. – Critical Care Medicine, 2017, 10.1097/CCM.0000000000002652
2. Key characteristics impacting survival of COVID-19 extracorporeal membrane oxygenation – Herrmann et al. for the German ECMO COVID Study Group – Critical Care, 2022, 10.1186/s13054-022-04053-6
3. Multi-Drug Resistance Bacterial Infections in Critically Ill Patients Admitted with COVID-19 – D. Pasero, A. P. Cossu, P. P. Terragni – Microorganisms, 2021, 10.3390/microorganisms9081773

**Table 1:** **(abstract A50).** See text for description

Patients’ Characteristics	
Age (years), *median (IQR)*	52 (46–59)
Gender (female) *N (%)*	4 (16.7)
BMI, *median (IQR)*	28.34 (25–36.7)
PBW (kg), *median (IQR)*	67.7 (63.7–70.5)
SAPS II, *median (IQR)*	22 (18.5–27)
APACHE II, *median (IQR)*	54.5 (45–64)
SOFA score on ICU admission, *median (IQR)*	9.5 (6–12)
NIV before OTI, *N (%)*	12 (50)
Days on MV before vvECMO, *median (IQR)*	7.5 (2.5–11)
Non-COVID-19	6 (2–8)
COVID-19	11 (7–13)
Days on NIV plus MV before vvECMO, *median (IQR)*	10 (3.5–16)
Non-COVID-19	8 (3–13)
COVID-19	18 (11–22) *
PaO_2_/FiO_2_ before vvECMO, *median (IQR)*	74.9 (61.35–78.5)
PaO_2_/FiO_2_ during vvECMO, *median (IQR)*	180 (137.4–228.7)
COVID-19, *N (%)*	7 (29,2)
Pplat before vvECMO, *median (IQR)*	28 (26–29)
Non-COVID-19	28 (27–29)
COVID-19	26 (24–29)
Pplat during vvECMO, *median (IQR)*	25.7 (24.3–27.1)
Non-COVID-19	26 (25.1–27.3)
COVID-19	24.6 (23.7–25.7)
C_stat_ before vvECMO, *median (IQR)*	30 (25.5–39)
Non-COVID-19	31 (30–40)
COVID-19	28 (21.4–37.5)
Days on vvECMO, *median (IQR)*	14.5 (10–21.5)
Non-COVID-19	13 (10–21)
COVID-19	15 (10–22)
**MDR infections, ** ***N(%)***	18 (75)
Klebsiella pneumoniae	11 (45.8)
Pseudomonas aeruginosa	3 (12.5)
Acinetobacter	4 (16.7)
E. Coli	1 (4.17)
MRSE	5 (20.8)
**Microbial culture, ** ***N(%)***	
BAL	17 (70.8)
Bloodstream	10 (41.67)

*BMI* body mass index, *PBW* predictive body weight, *NIV* non-invasive ventilation, *MV* mechanical ventilation, *MDR* multidrug resistance, *BAL* bronco alveolar lavage

^*^Unpaired T-test, p = 0.004

### A51 May an early argipressin-norepinephrine regimen play a role in minimize use of crrt in perioperative septic shock? A 28 months experience in a post-surgical intensive care unit

#### A. Pigliacelli^1^, N. Pavanello^2^, P. Sgarlata^2^, N. D Agostino^2^, F.R. Misiti^2^, S. Argenio^2^, P. Marino^2^

##### ^1^Dipartimento di Anestesia, Rianimazione e Terapia del Dolore. Ospedale Universitario Sant Andrea, Università Sapienza, Roma, Italy; ^2^Terapia Intensiva Post-Operatoria ed Anestesia in Emergenza. Azienda Ospedaliera San Giovanni Addolorata, Roma, Italy

###### **Correspondence:** A. Pigliacelli

*Journal of Anesthesia, Analgesia and Critical Care 2024*, **4(1):**A51

**Introduction**

Septic shock may be identified by lactate levels > 2 mmol/L despite fluid resuscitation and persistent hypotension, requiring vasopressor administration to maintain mean arterial pressure (MAP) > 65 mmHg. [1] Hemodynamic and metabolic changes increase mortality up to 70% if complicated by acute kidney injury (AKI). The Surviving Sepsis Campaign (2021) suggests to add argipressin up to 0.03 UI/min as catecholamine-sparing strategy in case of inadequate MAP during low to moderate norepinephrine infusion (0.25–0.5 mcg/Kg/min). Recent data suggest that “early regimen” argipressin-norepinephrine (starting argipressin 0.03 UI/min within 3 h at 0.1–0.2 mcg/kg/min of norepinephrine) is not associated with a decrease in short-term mortality, shorter intensive care unit (ICU) length of stay and hospitalization but can reduce continuous renal replacement therapy (CRRT). [2] [3] In this abstract we describe results of increasing use of “early regimen” in patients with perioperative septic shock. The endpoint was to evaluate if number of CRRT was decreasing with the escalation of argipressin-norepinephrine “early regimen”.

**Methods**

47 patients’ data were collected retrospectively in a post-surgical ICU during the last 28 months. This Unit handles 1.000 patients/year. Population was homogeneous for gender, age, type of surgery and Sequential Organ Failure Assessment (SOFA) (11.8 ± 1.8). Sepsis source was mainly abdominal, with some urological, vascular and neurosurgical. Patients underwent invasive hemodynamic monitoring and started argipressin within the first 6 h at 0.2 mcg/Kg/min of norepinephrine (2022), progressively adopting an “early regimen” (2023–2024). Number of CRRT was recorded. Chronic kidney failure patients were excluded.

Results We observed a decreased need for CRRT in septic shock when argipressin was associated to norepinephrine at a medium–low dose (2022), which became even more striking with “early regimen” infusion (2023–2024). In 2022 out of 13 patients receiving argipressin-norepinephrine, only one required CRRT. 6 received only norepinephrine, all requiring CRRT (63% no CRRT). In 2023 out of 17 receiving “early regimen”, only one required CRRT. One patient received norepinephrine only, requiring CRRT (88% no CRRT). Trend in 2024 appears even more dramatic: all 10 patients affected by septic shock have been treated with “early regimen” with only one undergoing CRRT (90% no CRRT).

Discussion Despite widespread argipressin use, its potential renal benefits are unclear. Important studies report that argipressin treatment only potentially led to a clinical benefit in terms of AKI-free days. [4] [5] [6] “Early regimen” treatment with argipressin-norepinephrine might be beneficial because involving endocrine replacement, as argipressin levels lower rapidly during hypotension. Moreover norepinephrine-sparing ability may limit immunomodulatory harmful effects. [7] Furthermore, argipressin may better maintain renal perfusion due to V1a receptors binding and reduce harmful fluid overload. [8] In our experience, CRRT has been progressively neglected, therefore we postulated a beneficial effect of “early regimen” argipressin-norepinephrine. Limitations: small sample size, argipressin plasma levels not available, uninvestigated steroids combination, lack of data on mortality.

**Conclusions**

In our experience, argipressin-norepinephrine “early regimen” in post-surgical septic shock, reduced dramatically number of CRRT. Further analysis is required to evaluate impact of early argipressin-norepinephrine on AKI.

**References**

1. Singer M, Deutschman C, Warren Seymour C, Shankar-Hari M, Annane D, Bauer M et al. The third international consensus definitions for sepsis and septic shock. JAMA. 2016; 315(8): 801–810
2. Huang H, Wu C, Shen Q, Xu H, Fang Y, Mao W. The effect of early vasopressin use on patients with septic shock: a systematic review and meta-analysis. J Emerg Med. 2021; 48:203–208
3. Sedhai Y R, Shrestha D B, Budhathoki P, Memon W, Acharya R, Gaire S et al. Vasopressin versus norepinephrine as the first-line vasopressor in septic shock: a systematic review and meta-analysis. J Clin Transl. 2022; 8(3): 185–199
4. Gordon A C, Mason A, Thirunavukkarasu N, Perkins GD, Cecconi M, Cepkova M et al. Effect of early vasopressin vs norepinephrine on kidney failure in patients with septic shock. The VANISH randomized clinical trial. JAMA. 2016; 316(5): 509–518
5. Russell J A, Walley K R, Singer J, Gordon A C, Hébert PC, James D et al. Vasopressin versus norepinephrine infusion in patients with septic shock. N Engl J Med. 2008; 358(9): 877–87
6. Abrahão Hajjar L, Zambolim C, Belletti A, Pinheiro de Almeida J, Gordon A C, Oliveira G et al. Vasopressin versus norepinephrine for the management of septic shock in cancer patients: the VANCS II randomized clinical trial. Crit Care Med. 2019; 47(12): 1743–1750
7. Sacha G L, Lam S W, Wang L, Duggal A, Reddy A J, Bauer S R. Association of catecholamine dose, lactate, and shock duration at vasopressin initiation with mortality in patients with septic shock. Cri Care Med. 2022; (50)4:614–623
8. Xu J, Cai H, Zheng X. Timing of vasopressin initiation and mortality in patients with septic shock: analysis of the MIMIC-III and MIMIC-IV databases. Infectious Diseases. 2023; 23:199

### A52 The impact of continuous renal replacement therapy on linezolid clearance

#### A. Peralta^1^, G. Pandolfo^2^, E. Pistollato^1^, S. Congedi^2^, G. Coniglio^2^, L. Muraro^1^, P. Navalesi^1,2^

##### ^1^Anesthesia and Intensive Care, University Hospital od Padua, Padua, Italy; ^2^Department of Medicine, University Hospital od Padua, Padua, Italy

###### **Correspondence:** G. Pandolfo

*Journal of Anesthesia, Analgesia and Critical Care 2024*, **4(1):**A52

**Introduction**

Linezolid is an oxazolidinone antimicrobial with time-dependent activity and persistent post-antibiotic effects. It is a first-line treatment for multidrug-resistant Staphylococcus aureus and vancomycin- resistant Enterococcus.

Recent evidence deriving from preclinical and clinical studies has reported the need to reach more aggressive pharmacokinetic/pharmacodynamic (PK/PD) targets to maximize clinical efficacy and minimize the onset of drug resistance. PKs of anti-microbial agents are profoundly altered in critically ill patients due to compromised function of vital organs and because of therapeutical devices that can modify metabolic and excretory capacities. Due to its low molecular weight and low protein binding, linezolid is highly predisposed to be affected by extracorporeal clearance such as during veno-venous extracorporeal membrane oxygenation (VV-ECMO) and continuous or intermittent renal replacement therapy (RRT).

We report data on the use of linezolid in three critically ill patients with an infection-related ventilator-associated complication (IVAC), specifically a bloodstream infection following severe pneumonia due to MRSA, requiring RRT.

**Materials and Methods**

2. 1. Drug sampling and assay

The analysis was carried out on the first linezolid dosing. To calculate the parameters of extracorporeal removal, samples were simultaneously collected pre-filter, post-filter, and from the effluent 1 h after the initiation of infusion (Cmax—peak serum concentration) as reported in Table 1.

2.2. Pharmacokinetic analysis 

Plasma linezolid time-concentration data were determined using high-performance liquid chromatography (HPLC). A compartmental analysis was used to estimate pharmacokinetic parameters. Linezolid extracorporeal clearance (ClST-150) was estimated as the product of the dialysate saturation coefficient (DSA) and the effluent flow (Qef):

$$\mathrm{ClST}-150\hspace{0.17em}=\hspace{0.17em}\text{DSA x Qef}$$

The DSA was calculated as the ratio of linezolid concentration evaluated in effluent flow (Cef) over the amount reported in after (Cpre) and before (Cpost) filter samples, according to the following formula:

$$\mathrm{DSA}\hspace{0.17em}=\hspace{0.17em}2\hspace{0.17em}\times \hspace{0.17em}\text{Cef Cpre}\hspace{0.17em}+\hspace{0.17em}\mathrm{Cpost}$$

**Results**

We present three cases of IVAC due to MRSA isolated from respiratory tract sample (bronchoalveolar lavage) and blood samples. Patients’ characteristics and RRT parameters are summarized in Table 2.

**Conclusions**

Vigilant monitoring of linezolid therapy is essential to prevent antibiotic ineffectiveness. Our clinical cases illustrated that the pharmacokinetics of linezolid were significantly affected by CVVHDF resulting in a dose reduction during initial administration. There is a wide interindividual variability in the amount of drug eliminated despite the homogeneity within the examined population (severity of renal impairment) and the type of treatment (CVVHDF) and filter (ST-150) used. A therapeutic drug monitoring could be an effective method to ensure adequate antibiotic exposure. Further studies with a larger sample size are needed.

Informed consent was obtained.

**Table 1:** **(abstract A52).** Pre-filter, post-filter, and effluent linezolid dosing

	**Patient 1**	**Patient 2**	**Patient 3**
pre-filter	12,4 mg/mL	2,88 mg/mL	11,68 mg/mL
post-filter	9,3 mg/mL	2,29 mg/mL	10,50 mg/mL
effluent	9,3 mg/mL	2,45 mg/mL	8,74 mg/mL

**Table 2:** **(abstract A52).** See text for description

	**Patient 1**	**Patient 2**	**Patient 3**
**Sex**	Male	Male	Female
**Age (years)**	75	53	64
**Weight (kg) / BMI (kg/m2)**	75 / 32	96 / 26,9	50 / 22,2
**Diagnosis at admission**	respiratory failure	respiratory failure	double-lung transplant
**APACHE II score**	26	24	26
**SOFA score**	15	13	15
**NYHA**	Class II	Class I	Class III
**Medical history**	type 2 diabetes mellitus on insulin therapy	active smoker	severe pulmonary hypertension
	previous enucleation of left kidney		usual interstitial pneumonia (UIP)
	radical cysto-prostatectomy, residual cutaneous ileal-cystostomy		acute heart failure, mild mitral regurgitation, mild to moderate aortic stenosis
	myasthenia gravis (no treatment)		intermediate (50%) stenosis of the anterior descending artery
			paroxysmal atrial fibrillation on direct oral anticoagulants
			GERD from H. pylori
			iron-deficiency anaemia
			nodular formation in left breast
**acute kidney injury stage (KIDGO criteria)**	AKI G3	AKI G3	AKI G3
**Type of RRT**	CVVHDF	CVVHDF	CVVHDF
**Set**	ST-150	ST-150	ST-150
**Anti-coagulation**	regional citrate anticoagulation (RCA)	none	none
**Blood flow (ml/min)**	120	200	200
**PBP flow (ml/h)**	1000 (Regiocit)	500 (Prismasol 2)	400 (Prismasol 2)
**Dialysate flow (ml/h)**	1100 (Prism0cal B22)	2500 (Prismasol 2)	1500 (Prismasol 2)
**Reinfusion flow (ml/h)**	500 (Phoxilium)	500 (Prismasol 2)	400 (Prismasol 4)
**Pre-dilution (%)**	0	0	0
**Calcium compensation (%)**	100	100	100
**Treatment duration in hours per day**	24	24	24

### A53 Critically ill young patient with influenza a (H1N1)-related pneumonia and other complications

#### A. Usai, G. Olla, S. Pilloni, F.M. Loddo

##### SC Anestesia e Rianimazione, ASL 4 Ogliastra, Lanusei, Italy

###### **Correspondence:** A. Usai

*Journal of Anesthesia, Analgesia and Critical Care 2024*, **4(1):**A53

**Background**

The majority of patients with influenza A (H1N1) have self-limited, mild-to-moderate uncomplicated disease; however, some patients develop severe illness and some die. The major complications associated with influenza A infection are: pneumonia, either primary influenza pneumonia or secondary bacterial pneumonia, Intensive Care Unit (ICU) admission, need for mechanical ventilation, non-invasive mechanical ventilation, acute respiratory distress syndrome (ARDS), septic shock.

**Case report**

A 44-year-old man (in good health before admission) presented to the emergency department with worsening dyspnea and fever up to 39 °C. He showed: acute respiratory distress syndrome (ARDS) with peripheral oxygen saturation (SpO2) 70%; partial pressure of oxygen in the arterial blood (PaO2) was 40 mmHg, and the ratio of the fraction of inspired oxygen (FiO2) to the partial pressure of oxygen in the arterial blood (PaO2) was 51. He performed a chest X-ray which showed ground-glass opacities with areas of consolidation. Finally, he was admitted in ICU, where he performed a rhinopharyngeal swab which was positive for influenza A (H1N1).

The patient also had signs of systemic inflammation indicated by C-reactive protein (CRP) 279 mg/l, procalcitonin 2,33 ng/ml, and lowered leukocyte levels (3,3 × 103 /mcl).

The patient was immediately treated with oseltamivir. In the clinical suspicion of a bacterial infection, he performed a bronchoalveolar lavage; he received broad-spectrum empirical antimicrobial therapy tailored to local prevalence and resistance patterns; then he was treated with targeted antimicrobial therapy; he also needed norepinephrine administration.

Due to failure of non-invasive mechanical ventilation, the patient was administered continuous sedation, muscle relaxation, intubation, and mechanical ventilation with a FiO2 80% and a lung-protective ventilation strategy. Prone positioning was applicated since the second day from admission for at least 16 h daily. Despite all therapeutic and supportive measures, the patient’s conditions got better only on 20st day from admission, when he was gradually weaned from mechanical ventilation. After 32 days from admission the patient was discharged in a rehabilitation center.

**Conclusions**

Some patients with influenza A (H1N1) infection develop severe illness due to complications. Secondary bacterial respiratory infections are well known determinants of poor clinical outcomes in patients with influenza A because they can lead to ARDS and septic shock. Earlier clinical suspicion, early identification of risk factors, use of biomarkers of infection, as well as early empirical use of antibiotics might improve the prognosis of this patients.

Informed consent was obtained.

**References**

1. Viasus et al., Influenza A(H1N1)pdm09-related pneumonia and other complications Enferm Infecc Microbiol Clin. 2012;30(Supl 4):43–48
2. Arranz-Herrero et al., Determinants of poor clinical outcome in patients with influenza pneumonia: A systematic review and meta-analysis, International Journal of Infectious Diseases 131 (2023) 173–179
3. Kovacevic et al., Use of CytoSorb® as a therapeutic option in a critically ill patient with acute respiratory distress syndrome caused by influenza A (H1N1) pneumonia: A case report. Int Journal of Critical Illness & Injury Science. 2020 Oct-Dec; 10(4): 216–219

### A54 Rhombencephalitis due to listeria monocytogenes and cavitary pneumonia caused by nocardia otitidiscaviarum in an immunocompromised patient: a case report with literature review

#### G. Olla^1^, S. Pilloni^1^, A. Usai^1^, M. Fiamma^2^, S. Doneddu^3^, F.M. Loddo^1^

##### ^1^SC Anestesia E Rianimazione, ASL 4 Ogliastra, Lanusei, Italy; ^2^SC Laboratorio Analisi, ASL 3 Nuoro, Nuoro, Italy; ^3^SC Laboratorio Analisi, ASL 4 Ogliastra, Lanusei, Italy

###### **Correspondence:** S. Pilloni

*Journal of Anesthesia, Analgesia and Critical Care 2024*, **4(1):**A54

**Background**

Nocardiosis due to N. otitidiscaviarum is a rare opportunistic disease, accounting for only 2–3% of all nocardia infections [1]. We present the case of a 51-year-old immunocompromised male affected by monoclonal gammopathy of undetermined significance (MGUS), autoimmune hemolitic anemia, and autoimmune hepatitis, who developed a cavitary penumonia sustained by N. otitidiscaviarum and a L. monocytogenes infection resulting in a rhombencephalitis. After extensive research on PubMed with the terms “nocardiosis”, “Nocardia otitidscaviarum”, “Nocardia caviae”, we found 65 cases of human nocardiosis sustained by the species otitidiscaviarum, reported from 1974 to 2023. We also added the terms “Listeria”, “listeriosis” and “rhomboencephalitis” to the research, and, to the best of our knowledge, no cases of N. otitidiscaviarum and L. monocytogenes coinfection have ever been described, apart from our case report.

**Case Report**

At the time of admission to our ICU the patient presented with altered mental status and respiratory failure The patient was initially treated with Meropenem, Levofloxacin and Linezolid as empiric antimicrobial therapy, until N. otitidiscaviarum and L. monocytogenes were isolated from broncho-alveolar lavage (BAL) and blood culture respectively, in the presence of a negative CSF culture [2]. The identification of N.otitidiscaviarum was done by matrix-assisted laser desorption ionization time of flight mass spectrometry (MALDI-TOF). Upon the results of antimicrobial susceptibility tests, definitive antimicrobial therapy was started with intravenous Meropenem 2 g × 3/die, Amikacin 15 mg/kg/die, Trimethoprim-Sulfamethoxazole (TMP-SMX)15 mg/kg/die. TMP-SMX was later shifted to oral administration, for a total of 6 months of therapy. Despite the improved overall clinical status, due to the persistence of a prolonged ventilator dependence, a percutaneous dilatational tracheostomy (PDT) with modified Percu-twist technique [3] was performed on the patient, resulting in a faster weaning from mechanical ventilation and eventually, in the presence of negative BAL and blood cultures, in a complete clinical recovery.

**Conclusions**

N. otitiscaviarum and L. monocytogenes coinfection is a rare event, as seen in our literature review. Prompt and early use of rapid microbiological methods, radiological investigations, and an accurate anamnesis, particularly in the case of L. monocytogenes infections, represent essential conditions for early definitive antimicrobial therapy and a positive clinical outcome.

Informed consent was obtained.

**References**

1. Parengal J., Alebbi S.M., Hamed M.M.M., Alqatami H.M., Ben Abid F., 2021. Disseminated life threatening Nocardia otitidiscaviarum infection in a young female with newly diagnosed systemic lupus erythematosus, case report and review of literature. IDCases 26, e01265. 10.1016/j.idcr.2021.e01265
2. Sahin H., Cengiz F., Tuncel, D., 2021. A rare cause of brainstem encephalitis in an immunocompetent young patient: Case report and review. Neuroimmunology Reports 1, 100,025. 10.1016/j.nerep.2021.100025
3. Pilloni S., Loddo F.M., A technical modifcation for percutaneous dilatational tracheostomy: our experience. Journal of Anesthesia, Analgesia and Critical Care 2023, 3(Suppl 1):A162

### A55 False positive in blood cultures in ICU, single-center retrospective study: incidence and future prospects

#### M. Guarnera, A. Monesi, A. Roppa, S. Tantillo, N. Cilloni

##### AUSL di Bologna, Ospedale Maggiore, Terapia Intensiva e HUB Maxiemergenze, Bologna, Italy

###### **Correspondence:** M. Guarnera

*Journal of Anesthesia, Analgesia and Critical Care 2024*, **4(1):**A55

**Introduction**

In the septic patient it is necessary to guarantee an adequate diagnostics for the severity of the pathology, using correct clinical practices to increase the probability of isolating the pathogens responsible for bacteremia and having the possibility of evaluating their in vitro sensitivity to antimicrobials allowing the transition from therapy empirical to targeted therapy, and it is necessary to avoid the risk of contamination and/or incorrect storage of the samples with consequent difficulties in interpreting the results.1 The microbiological results allow the correct use of antibiotics, according to the Antimicrobial Stewardiship program.2 In this context, false positive results of culture tests are particularly important, and determine: use of inappropriate antibiotic therapy, delay in the diagnosis of infection, increase in the number of laboratory and instrumental tests, increase in vascular access replacement procedures; increased hospital costs, increased hospitalization times, increased patient morbidity and mortality.3 The aim of the study is to evaluate the frequency of false positives in blood cultures of patients admitted to the Intensive Care Unit (ICU).

**Patients and Methods**

The study was conducted in ICU of the Maggiore Hospital in Bologna, between 01/01/2023 and 06/30/2023, with the approval of the Ethics Committee of Bologna. Includes all patients over 18 years who underwent blood culture sampling in the clinical suspicion of sepsis. In order to evaluate the frequency of contaminants the following measurements were carried out:

- N contaminated samples/Number of samples performed X 100
- N contaminated samples/number of positive samples × 100

**Results**

In the period studied, 162 patients were admitted to ICU; number of samples taken was 1930; number of false positives 3.83%, false positives compared to the positive sample: 29.24%.

The isolated species are: S.epidermidis (63.5%), S.capitis (10.8%), S.hominis (8.1%), S.coagulase-negative (8.1%) and others (9.45%) with the collaboration of the microbiology laboratory and infectious disease consultants. The majority of false positivities result from central venous access sampling, such as colonization of the device, or sampling performed at the time of positioning, as sampling error.

**Conclusion**

In the literature it is recommended to have a contaminant frequency of less than 3%. The aim of the study was to verify the incidence of false positives in our setting, to know the extent and the possible sources of preventable errors, such as contamination at the time of sampling in order to introduce improvement actions, through staff training and studies prospective randomized clinical trials, to reduce the incidence of error and the consequences associated with it.

**References**

1. Evans L, Rhodes A, Alhazzani W et al. Surviving Sepsis Campaign: International Guidelines for Management of Sepsis and Septic Shock 2021. Crit Care Med. 2021 Nov 1;49(11):e1063-e1143. 10.1097/CCM.0000000000005337.
2. Expert Opinion Document SIAARTI Maggio 2023. Applicazione Clinica e Razionale dell’antimicrobial stewarship in terapia intensiva. https://www.siaarti.it/news?category_id=30366&block-tags%5Bcat_30448%5D=30448&block-tags%5Bcat_30366%5D=30366
3. Doern GV, Carroll KC, Diekema DJ et al. Practical Guidance for Clinical Microbiology Laboratories: A Comprehensive Update on the Problem of Blood Culture Contamination and a Discussion of Methods for Addressing the Problem. Clin Microbiol Rev. 2019 Oct 30;33(1):e00009-19. 10.1128/CMR.00009-19.

### A56 Rare infections in ICU: clinical cases of epidural abscess

#### M.C. Visone, F. Gritti, F. Di Biase, F. Aronne, I. Bicoku, A. Giaccio, C. Russo

##### ^1^A.O.R.N. A.Cardarelli—1 Servizio Anestesia e Rianimazione, Naples, Italy

###### **Correspondence:** M.C. Visone

*Journal of Anesthesia, Analgesia and Critical Care 2024*, **4(1):**A56

Epidural abscess is an infection characterized by accumulation of purulent material in the epidural space and is considered a serious pathology with high mortality and morbidity. The incidence is rare, 0.2–2 cases/10,000 hospitalizations. Primary or spontaneous SEAs, not related to pyogenic infectious spondylocytis, are due to the hematogenous spread of bacteria or fungi circulating in the epidural space. Once identified, spinal infections should be treated with targeted antibiotic therapy and, if possible, neurosurgical intervention. In our Intensive Care at AORN Cardarelli (Naples, Italy) we have managed 4 cases in a year of which two are of particular interest. The first case is a 59-year-old woman admitted to the emergency room due to suspected drug intoxication; she had GCS 15 with slightly abnormal laboratory tests. After approximately 24 h neurological deterioration, GCS 7.She is sedated, intubated, admitted to intensive care, and a brain MRI is performed which demonstrates triventricular hydrocephalus with signs of transependymal oozing, signal alteration in the lateral ventricles of a purulent nature; An EVD is positioned. Broad-spectrum antibiotic therapy and antifungal therapy begins: Meropenem, Linezolid, Levofloxacin, Amphotericin B-Liposomal. After culture responses we de-escalate the therapy and continue with Linezolid only. After weaning from ventilation and sedation the patient presents tetraplegia, a second spinal MRI is carried out: Thickening at the level of the perimedullary meningeal envelopes in the context of the dorsal-lumbar spinal canal with associated phlogistic collection, phlegmon type which extends into the lumbar canal from the medullary conus up to the cul-de-sac; at this level, even more marked, intense and uneven enhancement is observed, with signs of arachnoiditis and microabscesses. The patient starts Daptomycin and then undergoes L1-L3 decompressive laminectomy with surgical toilet of vast intracanal lumbar phlegmon. Samples are taken for bacteriological test which will be negative. The patient will suffer with a new hydrocephalus and several nosocomial infections with exitus. Second case: 59-year-old woman hospitalized with confusion, drowsiness, hypotension, peripheral cyanosis. In a few hours, septic shock develops, for which she is admitted to the ICU: At entry, microbiological screening and first-level tests are carried out: negative for infectious sources. She also practices CSF cultures. She begins therapy with Meropenem, Linezolid, Aciclovir, Dexamethasone and MRI of the spine is carried out: extra-axial collection, mainly epidural, posterior cervico-dorsal, probably C2-D12. The dural sac was imprinted with marked phenomena of spondyloarthrosis. The neurosurgeon makes no recommendations. Meanwhile, the CSF culture is positive for MSSA, as is the blood culture: Aciclovir and Meropenem are suspended, Linezolid continues, Phosfomycin and Daptomycin are added. The patient will develop other health care-related infections with exitus. For both of these clinical cases, informed consent was collected as required by Italian law.

**Fig. 1:**
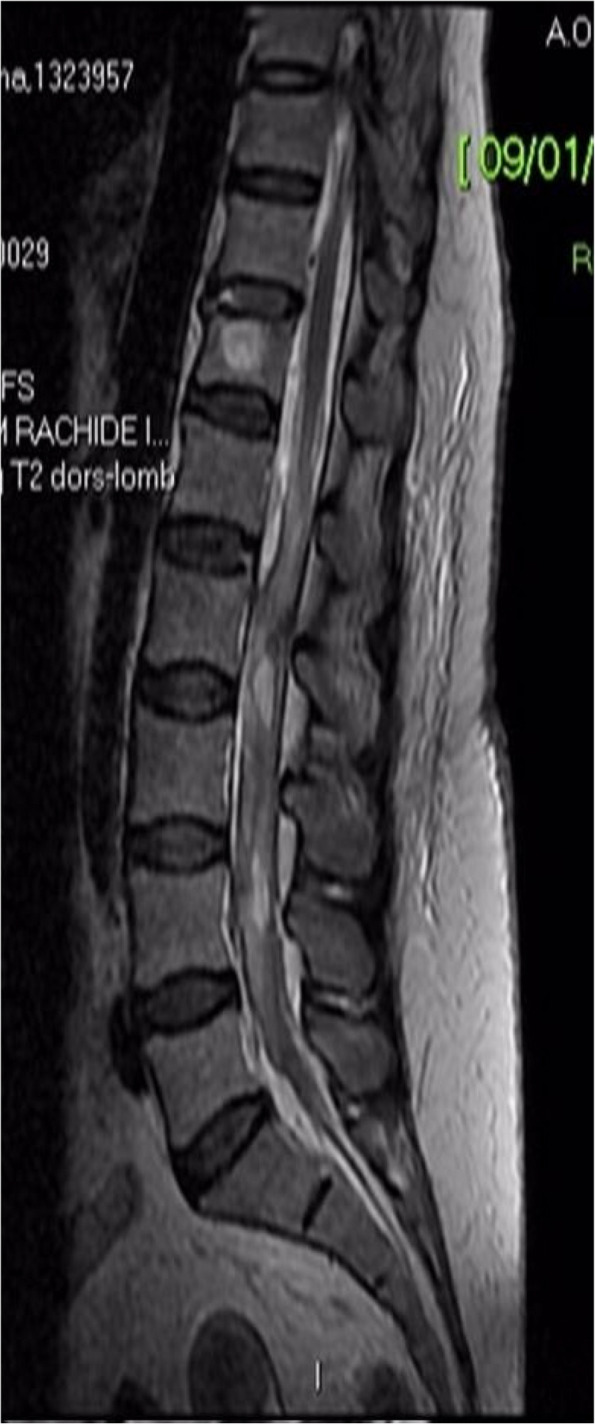
** (abstract A56).** See text for description

**Fig. 2:**
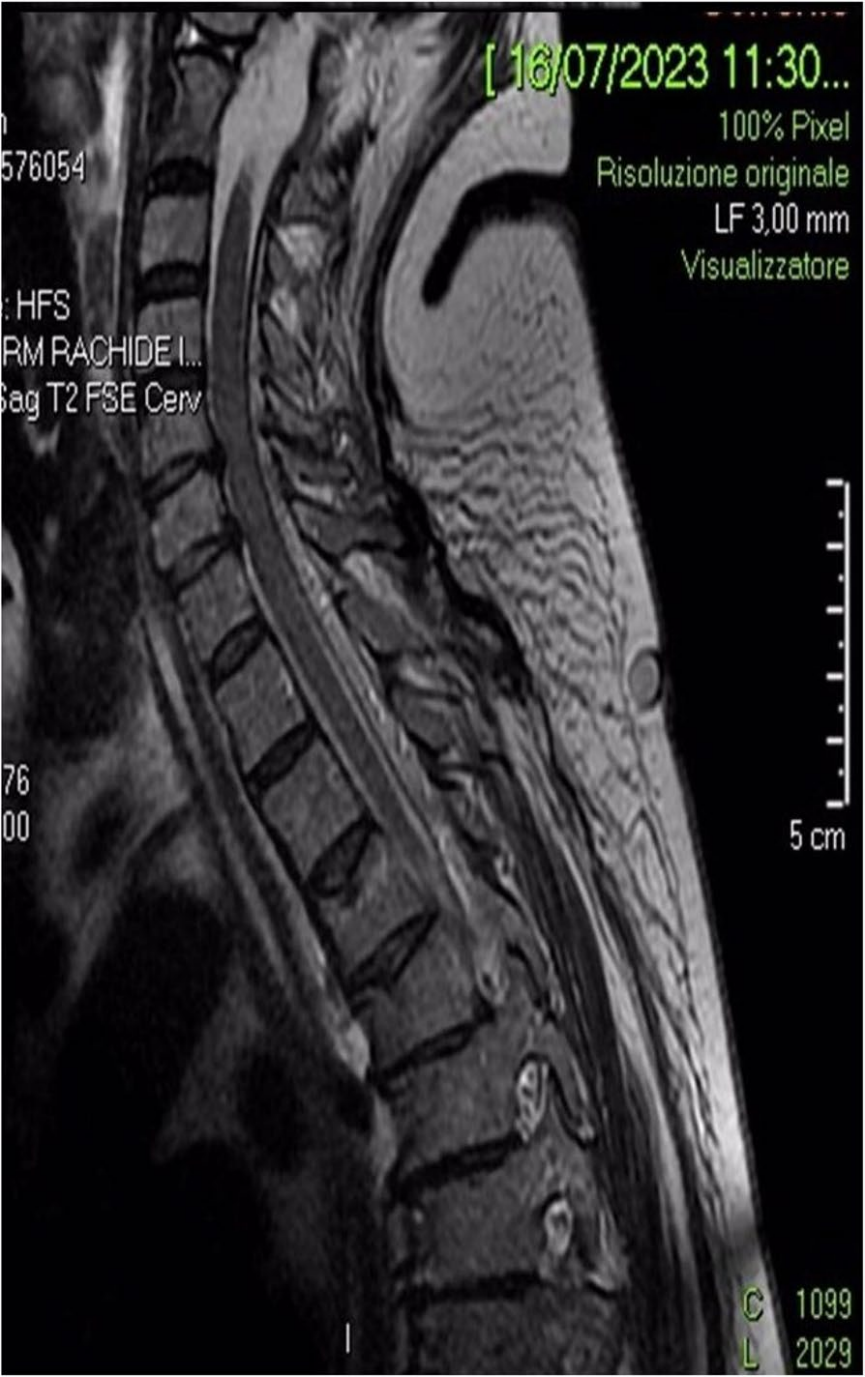
** (abstract A56).** See text for description

### A57 CR-BSI and ICD: a clinical case report

#### D. Di Gennaro, G. Merola, F. Aronne, V. Bossone, I. Bicoku, A. Giaccio

##### A.O.R. N. Antonio Cardarelli—1 Servizio Anestesia e Rianimazione, Napoli, Italy

###### **Correspondence:** D. Di Gennaro

*Journal of Anesthesia, Analgesia and Critical Care 2024*, **4(1):**A57

BSI represents an important clinical issue in the ICU due to its high mortality and comorbidity rates: the main risk factors include prolonged hospitalization, mechanical ventilation, renal replacement therapy, recent surgery and immunosuppression. Extensive use of intravascular catheters is recognized as the most important factor. Catheter-related BSIs, CR-BSIs, account for approximately 35% of cases of the most common BSIs. A 31-year-old female, with past medical history: hemolytic autoimmune anemia, previous bariatric surgery (sleeve gastrectomy and gastro-jejunal by-pass), severe sarcopenic malnutrition, papillary carcinoma treated with thyroidectomy, shows up at the emergency department in A.O.R.N. Hospital A. Cardarelli (Naples,Italy) for severe anemia (HB 5 g/dl) and after having cannulated a central vein, receives blood transfusions.

She was then admitted to the medical department where she had cardiorespiratory arrest with a VF rhythm, then CPR with ROSC at 5 min; then admitted to intensive care. PICCO haemodynamic monitoring began, a cycle of Levosimendan was practiced and the diagnosis of septic shock was made; Noradrenaline began (0, 5 mcg/kg/min) with the following blood tests: T 39*C, WBC 27.53(94%N) CRP 85, PCT 3.3 EAA 0.42, KP CRE rectal swab and entry microbiological screening negative. The potentially infected CVC is replaced and Linezolid (600 mg every 12 h) and Meropenem (2 g every 8 h) are started on empiric therapy, which is then suspended on the sixth day. Subsequently, the patient is alert, conscious and cooperative with adequate spontaneous breathing. However, the hemodynamic parameters are still dependent on norepinephrine at sustained doses. The KP-CRE swab is positive and the laboratory tests are declining: In this phase of hospitalization, numerous episodes of VF occur again for which the consultant cardiologists recommend a coronary angiography study ( result: negative) and give indication for ICD implantation. The infectious disease specialist gives the go-ahead for the implant only after obtaining negative blood cultures. On the twelfth day of hospitalization the patient is not sedated, she breathes spontaneously in O2 therapy. Cultures show colonization on tracheoaspirate for ACIBAU and KPC CRE.

The surveillance blood culture is KP-CRE positive (Fig. 1), so targeted ATB is started with Amikacin (15 mg/kg day) and Colistin (loading dose: 9 MIU + 4.5 MIU every 12 h) After 7 days antibiotic therapy is suspended with normalized laboratory tests. The implantation of the ICD is organized but a positive blood culture for ACI BAU MDR makes a new infectious disease re-evaluation necessary with the indication to practice Cefiderocol (2 g every 8 h per day). As the patient is no longer unstable, she is transferred to Internal Medicine Ward with: negative blood cultures, no signs of sepsis and ongoing antibiotic therapy after 25 days of hospitalization in the ICU. As soon as blood cultures are repeatedly negative, the patient implants the ICD on the 43rd day. She was finally discharged after 46 days of hospital stay. This clinical case is published after having asked the patient for consent as per Italian laws.

**Fig. 1:**
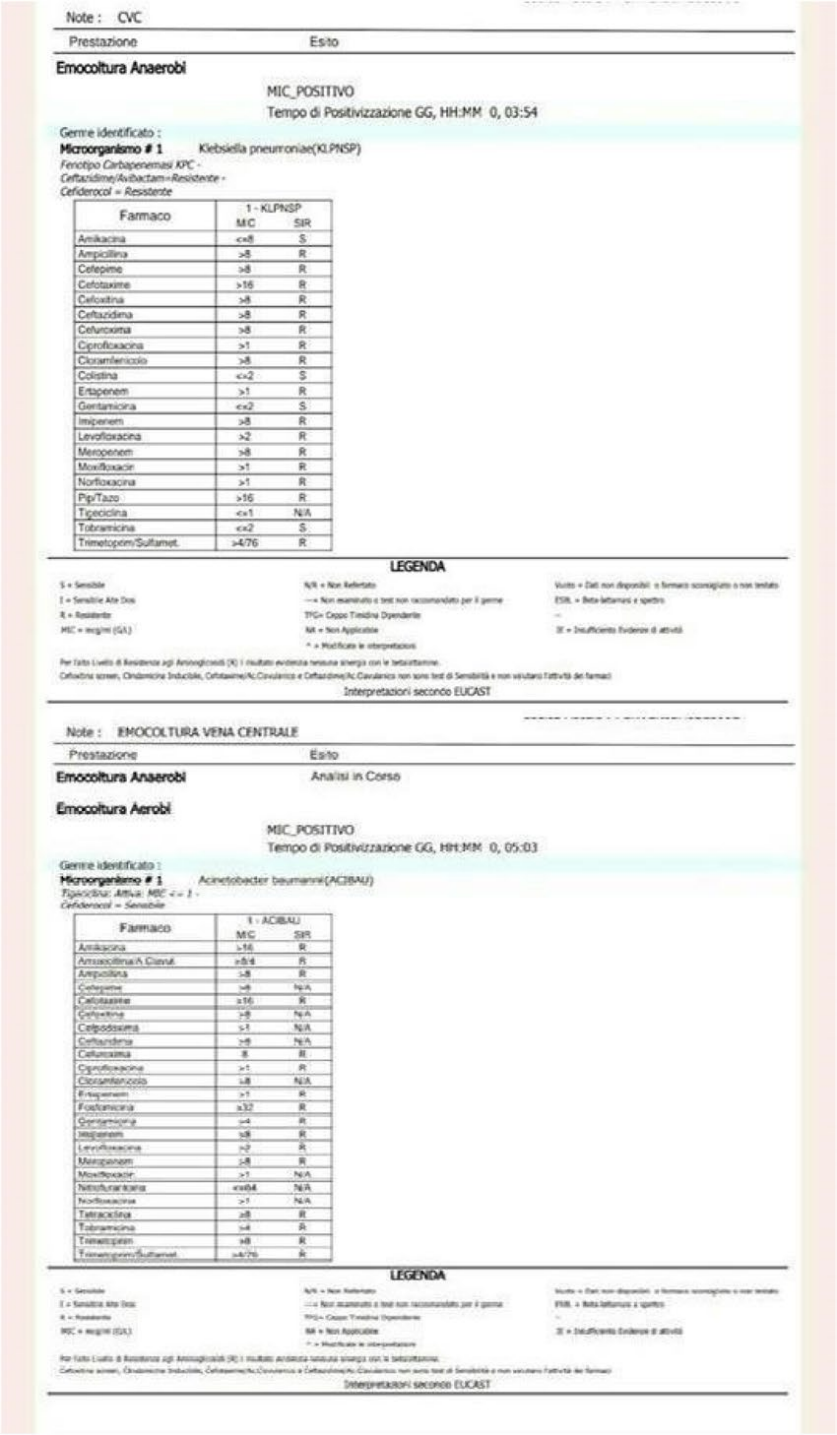
** (abstract A57).** See text for description

### A58 Capnocytophaga canimorsus meningitis complicated by septic shock: use of extracorporeal blood purification techniques

#### R. Giurazza, S. Virno, M.V. Adriani, G. Messina, R.C. De Rosa.

##### AORN Ospedali dei Colli—Department of Anesthesia and Intensive Care—D. Cotugno Hospital, Naples, Italy

###### **Correspondence:** R. Giurazza

*Journal of Anesthesia, Analgesia and Critical Care 2024*, **4(1):**A58

A 48-year-old man was admitted to our Emergency Department with hyperpyrexia, confusion, and rigor nucalis, two days after a dog bite. His medical history included beta thalassemia major, hemochromatosis, liver cirrhosis, HCV infection and previous splenectomy. Due to high suspicion of meningitis, a rachicentesis was performed, with aspiration of turbid cerebrospinal fluid (CSF), with hypercellularity, hyperproteinorrachia, hypoglycorrhachia, and negative Biofire® FilmArray Meningitis Encephalitis (FAME) test. The patient was sedated, intubated and received dexamethasone, as well as empiric antibiotic therapy with meropenem and linezolid. After a few days, blood cultures showed positivity to Capnocytophaga canimorsus; therefore, antibiotic therapy was switched to ceftriaxone. Given a significant improvement in his clinical conditions, the patient was extubated on day 7 (D7) and discharged to a ward on D11. Unfortunately, after only four days, the patient returned to our ICU in septic shock from Acinetobacter baumannii, requiring noradrenaline and vasopressin, with GCS < 9, pharmaco-resistant hyperpyrexia, elevated white blood cells (WBC), C reactive protein (CRP) and procalcitonin (PCT), normal endotoxin activity assay (EAA) and acute kidney injury (AKI). He was sedated, intubated again and mechanically ventilated. Besides antibiotics, we started a continuous renal replacement therapy (CRRT) with the Oxiris® hemofilter, also as an external physical cooling system. Despite improvement of CRP, PCT, renal function and weaning from vasopressors, the patient still showed fever and neurological impairment. Since the EAA on D24 resulted 0.9, we started two cycles of Toraymyxin® hemoperfusion. After these, the fever disappeared, EAA was normalized and his neurologic conditions improved. The patient was extubated on D27 and transferred again on D39. Written informed consent was obtained for the publication of this report.

C. canimorsus is an opportunistic Gram-negative bacterium, commonly found in the oral cavity of dogs, that can rarely cause meningoencephalitis in selected patients, such as those with beta thalassemia major, cirrhosis, hemochromatosis and splenectomy (1). Since C. canimorsus is not included in the FAME panel, clinicians should strongly suspect it in predisposed patients after dog bites. Different extracorporeal therapies have been proposed in septic shock, to reduce circulating levels of cytokines and endotoxin(2,3). We used the Oxiris® hemofilter in the first phase of cytokine storm, AKI and normal EAA. In contrast, Toraymyxin® hemofilter was used in a second moment, with elevated EAA and normal renal function. One hemofilter doesn’t exclude another, but the use of different filters must be linked to blood circulating levels of sepsis mediators, EAA and AKI.

**References**

1. Butler T. Capnocytophaga canimorsus: an emerging cause of sepsis, meningitis, and post-splenectomy infection after dog bites. European Journal of Clinical Microbiology & Infectious Diseases. 2015 Jul 1;34(7):1271–80.
2. Malard B, Lambert C, Kellum JA. In vitro comparison of the adsorption of inflammatory mediators by blood purification devices. Intensive Care Med Exp. 2018 Dec 4;6(1):12.
3. Shoji H, Opal SM. Therapeutic Rationale for Endotoxin Removal with Polymyxin B Immobilized Fiber Column (PMX) for Septic Shock. Int J Mol Sci. 2021 Feb 23;22(4):2228.

### A59 A secondary retrospective analysis investigating the diagnostic and prognostic value of activated neutrophils (NEUT-RI) in intensive care patients with and without sepsis

#### A. Menozzi^2^, M. Gotti^1^, L. Isidori^1^, S. Pastori^3^, V. Roccaforte^3^, E.M. Mantovani^1^, M. Iezzi^2^, F. Vetrone^1^, L. Foggetti^1^, G. Sabbatini^1^, A. Galimberti^1^, P. Formenti^1^, A. Pezzi^1^

##### ^1^SC Anestesia, Rianimazione e Terapia Intensiva, ASST Nord Milano, Ospedale Bassini,, Cinisello Balsamo, Italy; ^2^Scuola di Specializzazione in Anestesia, Rianimazione, Terapia del dolore; Univeristà degli studi di Milano-Bicocca, Milano, Italy; ^3^S.C. Analisi Chimico Cliniche e Microbiologiche, ASST Nord Milano, Ospedale Bassini, Cinisello Balsamo, Italy

###### **Correspondence:** A. Menozzi

*Journal of Anesthesia, Analgesia and Critical Care 2024*, **4(1):**A59

**Background**

Early identification and effective management of sepsis are crucial for favorable patient outcomes. In this regard, assessing neutrophil activation status (NEUT-RI) has emerged as a promising and easily interpretable parameter. This study aimed to evaluate the predictive value of NEUT-RI in diagnosing sepsis and its prognostic significance in predicting 28-day mortality.

**Materials**

This study conducted a secondary retrospective observational analysis, gathering clinical data upon ICU admission. A control group consisting of critically ill patients not meeting sepsis criteria was also included. Patients were categorized into subgroups based on renal function for biomarker assessment, and 28-day outcomes were documented for both septic and non-septic individuals.

**Results**

The study included 200 ICU patients. Significant differences were observed between the 'septic' and 'non-septic' groups for NEUT-RI (53.80 [49.65–59.05] vs. 48.00 [46.00–49.90], p < 0.001) (Table 1), as well as for PCT and CRP (PCT 8.83 [0.82—45.88] vs. 0.48 [0.29—1.64], p < 0.001; CRP 20.79 [12.54—118.91] vs. 6.68 [1.54–21.70], p < 0.001). The performance of each inflammatory parameter for the diagnosis of sepsis and their best cut-off values were described in Table 2, while the comparisons in AUROC were shown in Fig. 1. There were not statistically significant differences between AUROC of the three parameters (NEUT-RI vs PCT p = 0.83, NEUT-RI vs CRP p = 0.29, DeLong’s test for two correlated ROC curves). The CPR specificity is statistically different from the NEUT-RI and the PCT specificity (p = 0.01 and p = 0.007 respectively, specificity test for two correlated ROC curves) Regarding the 28-day outcome, overall mortality was 17.5%, with a trend towards higher mortality in the 'septic' compared to the 'non-septic' group (Table 3). NEUT-RI and PCT at ICU admission were higher in deceased septic patients compared to survivors (58.80 [53.85–73.10] vs 53.05 [48.90–57.22], p = 0.005 and 39.56 [17.39–83.72] vs 3.22 [0.59–32.32], p = 0.002, respectively).

**Conclusions**

The inflammatory biomarkers examined in this study provide valuable assistance in the prompt diagnosis of sepsis. Elevated NEUT-RI levels show particular promise for early sepsis detection and distinguishing severity upon admission.

**Fig. 1:**
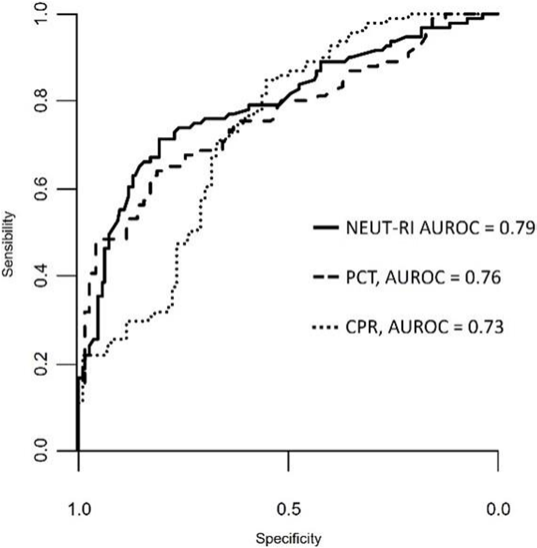
** (abstract A59).** See text for description

**Table 1:** **(abstract A59).** Comparisons of patients’ characteristics and inflammatory parameters divided in “septic” and “non-septic” patients (Panel A), and, among septic, in “not complicated sepsis” or “septic shock” patients (Panel B)

	A			B		
	**Septic**	**Non septic**	*P*	**Not complicated sepsis**	**Septic shock**	*P*
	(*n* = 89)	(*n* = 111)		(*n* = 47)	(*n* = 42)	
Age	73.00 [63.00—79.00]	73.00 [56.00—79.00]	0.454	74.00 [64.00—78.00]	73.00 [62.75—79.75]	0.808
Male%	48.4	52.3	0.679	59.6	35.7	0.042
Creatinine	1.57 [0.85—2.85]	1.00 [0.77—1.39]	< 0.001	1.16 [0.77—1.64]	2.18 [1.67—3.67]	< 0.001
CPR	20.79 [12.54—118.91]	6.68 [1.54—21.70]	< 0.001	18.79 [7.78—149.16]	23.03 [17.97—87.09]	0.7244
PCT	8.83 [0.82—45.88]	0.48 [0.29—1.64]	< 0.001	1.63 [0.40—12.09]	32.59 [8.83—100.00]	< 0.001
NEUT-RI	53.80 [49.65—59.05]	48.00 [46.00—49.90]	< 0.001	51.5 [47.80—56.30]	56.20 [52.30—61.92]	0.0054

**Table 2:** **(abstract A59).** NEUT-RI, PCT and CPR values for the detection of septic and non-septic patients

	AUROC	Cut-off	Sens	Spec	PPV	NPV
	(95% CI)		(95% CI)	(95% CI)	(95% CI)	(95% CI)
NEUT-RI	0.79	50.75	70.9	80.7	75.6	77.2
	[0.73—0.86]		[60.4—79.7]	[71.6–88.1]	[65.1–84.2]	[68.4–84.5]
CPR	0.73	7.38	84.6	56.2	65.8	77.8
	[0.66—0.80]		[75.8–91.2	[45.5–64.0]	[56.5–74.3]	[65.5–87.3]
PCT	0.76	2.17	62.9	82.9	82.3	63.7
	[0.69—0.84]		[52.8—74.2]	[76.4–90]	[71.2–90]	[52.9–73.6]

**Table 3:** **(abstract A59).** Comparison of inflammatory parameters for 28-day outcomes between "survivors" and "deceased" in the "septic" and “non-septic” patient group

	**Septic**			**Non-septic**		
	**Alive**	**dead**	*P*	**Alive**	**dead**	*P*
	(*n* = 78)	(*n* = 11)		(*n* = 95)	(*n* = 16)	
**PCR**	21.52 [11.48–137.89]	19.06 [14.10–36.49]	0.772	7.28 [1.88—21.70]	1.06 [0.41—80.00]	0.477
**PCT**	3.22 [0.59 -32.32	39.56 [17.39–83.72]	0.002	0.46 [0.28—1.21]	0.72 [0.57—1.10]	0.453
**NEUT-RI**	53.05 [48.90–57.22]	58.80 [54.45–73.35]	0.005	47.90 [45.80—49.82]	45.60 [44.00—47.60]	0.184

## Loco-regional anaesthesia

### A60 Should the administration of 1 mg of intrathecal morphine be considered an error or a learning opportunity?

#### M.R. Capuano^1^, E. Spasari^1^, G. D'Agostino^2^, M. Vargas^1^, I. Russo^3^, R. Damonte^1^, L. Gatta^1^, R. D'Angiò^1^, A. Coviello.^1^

##### ^1^Department of Neurosciences, Reproductive and Odontostomatological Sciences, University of Naples Federico II, Naples, Italy; ^2^ Department of Anesthesia and Intensive Care San Giuliano Hospital of Giugliano, Naples, Italy; ^3^ Department of Public Health, School of Medicine, University of Naples Federico II, Naples, Italy.

###### **Correspondence:** M.R. Capuano

*Journal of Anesthesia, Analgesia and Critical Care 2024*, **4(1):**A60

**Background**

The use of Intrathecal morphine has been well-known to provide postoperative pain relief since 1979 [1].

Despite its potential side effects, Intrathecal morphine remains an effective analgesic that can provide pain relief for up to 36 h.[2].

Studies have shown that the incidence of side effects, such as nausea, vomiting, pruritus, urinary retention, or respiratory depression, increases with the dose of Intrathecal morphine used during the perioperative period. [3].

To avoid side effects, it is crucial to pay close attention during the preparation of the mixture, since excessive dosage can be the main cause of major side effects.[4].

In medicine, human errors are possible and common, especially among trainees, but they offer valuable learning moments for improvement.[5] [Fig. 1].

**Case report**

A 37-year-old male patient weighing 100 kg and measuring 1.78 m in height, with a physical status of ASA II, was scheduled for reconstruction of the anterior cruciate ligament of the right knee. During the procedure, a trainee prepared the anesthetic blend and performed the puncture. However, after a few minutes from spinal anesthesia, it was noticed that the patient had become drowsy, and his respiratory rate varied from 10 to 14 bpm, with stable hemodynamics and oximetry around 95%. Upon revision with the senior anesthesiologist, it was discovered that the trainee had inadvertently given a dose of 1 mg of morphine intrathecally. The patient was treated with 0.4 mg of naloxone i.v. followed by a continuous infusion of 0.2 mg/h until the symptoms subsided. After 45 min of surgery, the patient was admitted to the post-anesthesia recovery unit, where his arterial blood pressure, respiratory rate, oxygen saturation, and cardiac frequency were stable during all observation time, Urinary retention was the only side effect, and it was resolved by urinary catheterization. During the following 24 h, the patient remained hemodynamically stable and did not experience any other complications until his discharge from the hospital. His NRS value was around 0 until discharge.

Once our team discovered the mistake, we monitored the situation and administered patient oxygen therapy through nasal cannulas; we conducted a real-time search of the literature on intrathecal morphine overdosing and the situation was handled calmly since we found several articles on the use of even higher dosages in patients over forty years. We followed the recommendations provided by the literature to manage the situation in the best possible way.

**Conclusions**

Our report suggests that a high dose of intrathecal morphine may be the most effective way to manage pain after surgery with minimal side effects, but it also warns about the risk of administering the wrong dose of medication during anesthesia.

However, the optimal dosage of morphine is still uncertain and requires further research.

This case highlights the significance of efficient teamwork and communication among all team members, particularly when multiple operators are involved in a procedure and the person administering the medication hasn't prepared it himself.

**Consent to publish**

Written informed consent was obtained from the patient.

**References**

1. J. K. Wang, L. A. Nauss, and J. E. Thomas, "Pain relief by intrathecally applied morphine in man," Anesthesiology, vol. 50, no. 2, pp. 149–151, 1979.
2. M. V. Koning, M. Klimek, K. Rijs, R. J. Stolker, and M. A. Heesen, "Intrathecal hydrophilic opioids for abdominal surgery: a meta-analysis, meta-regression, and trial sequential analysis," British Journal of Anaesthesia, vol. 125, no. 3, pp. 358–372, 2020.
3. Gwirtz KH, Young JV, Byers RS, et al. The safety and efficacy of intrathecal opioid analgesia for acute post-operative pain. Anesthesia & Analgesia1999;88: 599–604.
4. T.L.Rodziewicz,B.Houseman,and J.E.Hipskind,Medical Error Reduction and Prevention Continuing Education Activity"
5. R.Tevlin, E.Doherty, O.Traynor, "Improving disclosure and management of medical error—an opportunity to transform the surgeons of tomorrow", National Library of Medicine

**Fig. 1:**
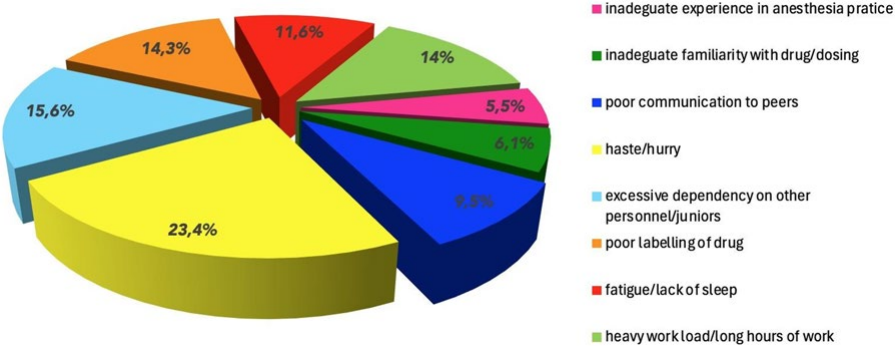
** (abstract A60).** Factors causing errors in drug administration

### A61 Comparison between peng block and perineural block of the lower limb in the pain management and post-operative recovery in elderly patients undergoing surgery for femur fracture

#### A. Zumpano^1^, C. Stummo^2^, G. Stagliano^1^, A. Monardo^1^

##### ^1^Presidio Ospedaliero Giovanni Paolo II, Lamezia Terme, Italy; ^2^ Azienda Ospedaliera Regionale San Carlo, Potenza, Italy

###### **Correspondence:** G. Stagliano

*Journal of Anesthesia, Analgesia and Critical Care 2024*, **4(1):**A61

**Introduction**

Femoral fracture has an high impact on the quality of life of the elderly patient, and requires a multimodal approach to optimize levels of care. The association of different local–regional anesthesia techniques has proven useful in the management of such patients. This study compared two loco-regional anesthesia techniques: the PENG block and the triple perineural block of femoral, obturator and femorocutaneous nerves. The main outcome of the study was to evaluate the effectiveness in analgesia.

**Materials and methods**

From April to October 2023 were recruited 37 patients who had given informed consent, aged between 65 and 90 that underwent surgery for femur fracture. The patients were divided into two groups, group A received the PENG block using 20 ml of Levobupivacaine 0,5% + Lidocaine 2%; group B received the triple perineural block with 10 ml of Levobipivacaine 0,5% + Lidocaine 2% on femoral nerve, 7 ml of Levobupivacaine 0,5% on obturator nerve and 5 ml of Levobupivacaine 0,5% on femorocutaneous nerve. In both groups, 15 min after the execution of the peripheral blocks, sub-arachnoid anesthesia was performed in a sitting position. The patients were evaluated at the end of surgery at 6 h, 12 h and 24 h with the Bromage scale and with the NRS scale.

**Results**

None of the patients complained of pain during surgery, and was observed a good hemodynamic stability. At the end of surgery Bromage 1 was observed in group A, Bromage 2 in group B. In the post-operative pain assessment, none of the patients required opioid drugs; the best pain control was obtained by adding Paracetamol. Comparing the mean of the NRS data, an increase was observed in both groups at 12 h post-operatively, higher in group B. Even at 24 h post-operatively, they are still higher in group B.

**Discussion**

The two techniques are comparable in pain control. In group A, an early remission of the motor block was observed even when residual paresthesia was present in group B, so an early mobilization was observed in group A. It was also observed that the ALR technique of group A, with a single puncture and a lower dosage of local anestetics, was better tolerated by the patients. Furthermore, we observed pain and burning localized on the surgical wound in group A after the complete remission of the effect of the subarachnoid anesthesia, this was related to the lack of involvement of the femoral-cutaneous nerve in the PENG-BLOCK.

**Conclusion**

Local–regional anesthesia techniques are extremely important in the peri-operative management of fragile patients. Those garantee a better analgesia and an overall reduction in the dosage of painkillers drugs. In particular, the PENG block technique makes the execution of the block faster and easier and allows a reduction in the dosage of local anesthetics administered.

### A62 Double tap block in high-risk patient for emergency laparotomy: the importance of ultrasound techniques

#### E. Trimarchi^1^, V.F. Tripodi^1^, V. Marando^1^, S. Di Stefano^1^, C. Mazzeo^2^, F. Fleres^2^, E. Cucinotta^2^, A.T. Mazzeo^1^

##### ^1^Anesthesia and intensive Care Section, Human Pathology Department Gaetano Barresi, G. Martino University Hospital, Messina, ITALY; ^2^ General Surgery Section, Human Pathology Department Gaetano Barresi, G. Martino University Hospital, Messina (Italy), Messina, Italy

###### **Correspondence:** E. Trimarchi

*Journal of Anesthesia, Analgesia and Critical Care 2024*, **4(1):**A62

**Background**

A 63-year-old patient is admitted with a distended and painful abdomen. The patient has a BMI value of 21 and is suffering from arterial hypertension being treated with ACE-i, COPD and pulmonary emphysema, severe aortic insufficiency, left atrial dilatation, subrenal abdominal aorta aneurysm (45 mm) and occlusion of the right external iliac artery up to the femoral artery; Atrial fibrillation treated with Rivaroxaban. He also takes ASA. Already underwent right unilateral nephrectomy for urothelial heteroplasia and with grade III hydronephrosis in the left kidney.

An occlusive pathology is documented by abdominal CT [Fig. 1], which shows marked distention of the jejuno-ileal loops with multiple air-fluid levels due to hernial involvement of very distended and leveled intestinal loops in the right inguinal canal with wall thickening and vascular suffering. An indication is therefore given for urgent surgery that cannot be postponed.

**Case report**

The patient arrives in the OR alert and cooperative with spontaneous eupneic breathing and with diffuse abdominal pain (VAS 5). HR ~ 112 b/min, PA 145/85 mmHg, SpO2 ~ 92%. He presents asthenia and hyperpyrexia, poor functional capacity (MET < 4) despite preserved pump function (FE > 60). Informed consent was obtained.

After his informed consent, and careful ultrasound evaluation, using a high-frequency linear probe (2–10 MHz), a parietal block of the transversus abdominis plane (Lateral TAP Block) is performed using a 90 mm reflective echo needle. 20 ml of long-acting anesthetic Ropivacaine 0.50% are administered with the addition of dexamethasone 4 mg with adjuvant action1,2. Subsequently, a block of the ileo-hypogastric and ileo-inguinal nerves is performed (Anterior TAP Block), with the aim of anesthetizing the right inguinal canal, administering 10 ml of Ropivacaine at a concentration of 0.375% with the addition of 4 mg of dexamethasone with an adjuvant action2,3. Intravenous infusion of Dexmedetomidine is started, started after a bolus of 1 mcg/kg IBW, and subsequently at a rate of 0.6–0.9 mcg/kg/h.

Surgery will consist of inguinal hernioplasty with partial resection of the loops with lateral-lateral anastomosis [Fig. 2]. At the end of the operation, the patient is alert, hemodynamically stable, SpO2 ~ 95% with a FiO2 32% and after a short period of observation in the recovery room, he returns to the ward.

**Conclusion**

The patient, presenting numerous comorbidities, many of which were not adequately investigated due to the non-deferrability of the emergency, is at high risk of intra and post-operative complications and could be a candidate for hospitalization in intensive care in case of general anesthesia. Furthermore, neuraxial anesthesia is absolutely contraindicated due to the intake of NOACs on the same day of the operation. The execution of the Double TAP block (Lateral + Anterior)2,4 [Fig. 3] make it possible to anesthetize the skin, muscles and tissues of the inguinal canal, furthermore the continuous conscious sedation using Dexmedetomidine allowed to maintain spontaneous breathing and manipulation of the bowels until ischemic loops resection. The patient is returned to the ward without pain.

**Fig. 1:**
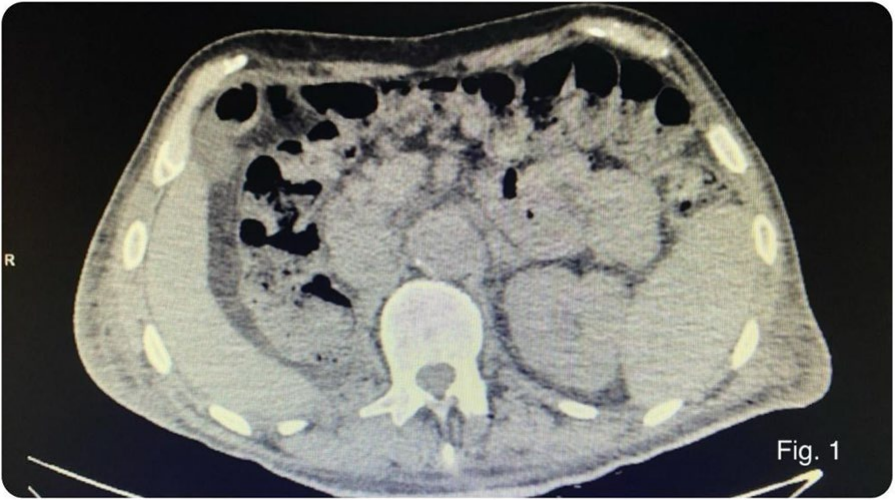
** (abstract A62).** Abdominal CT

**Fig. 2:**
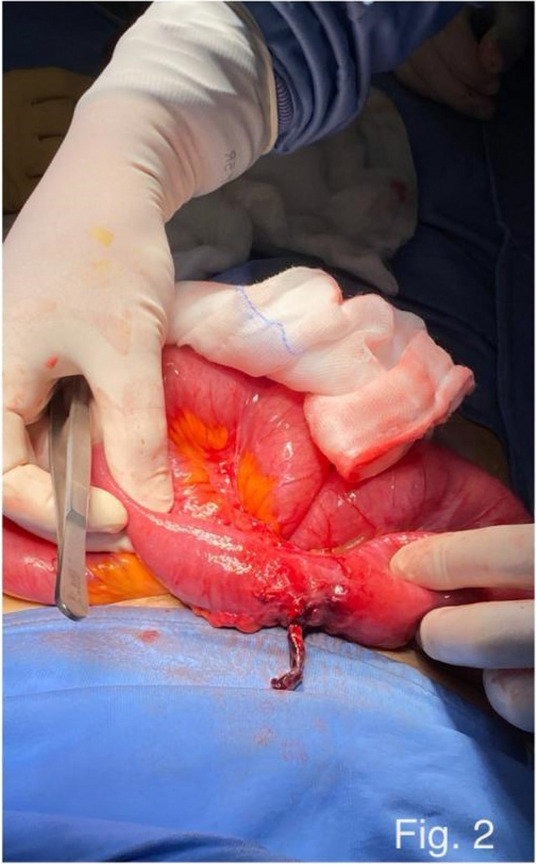
** (abstract A62).** Surgery

**Fig. 3:**
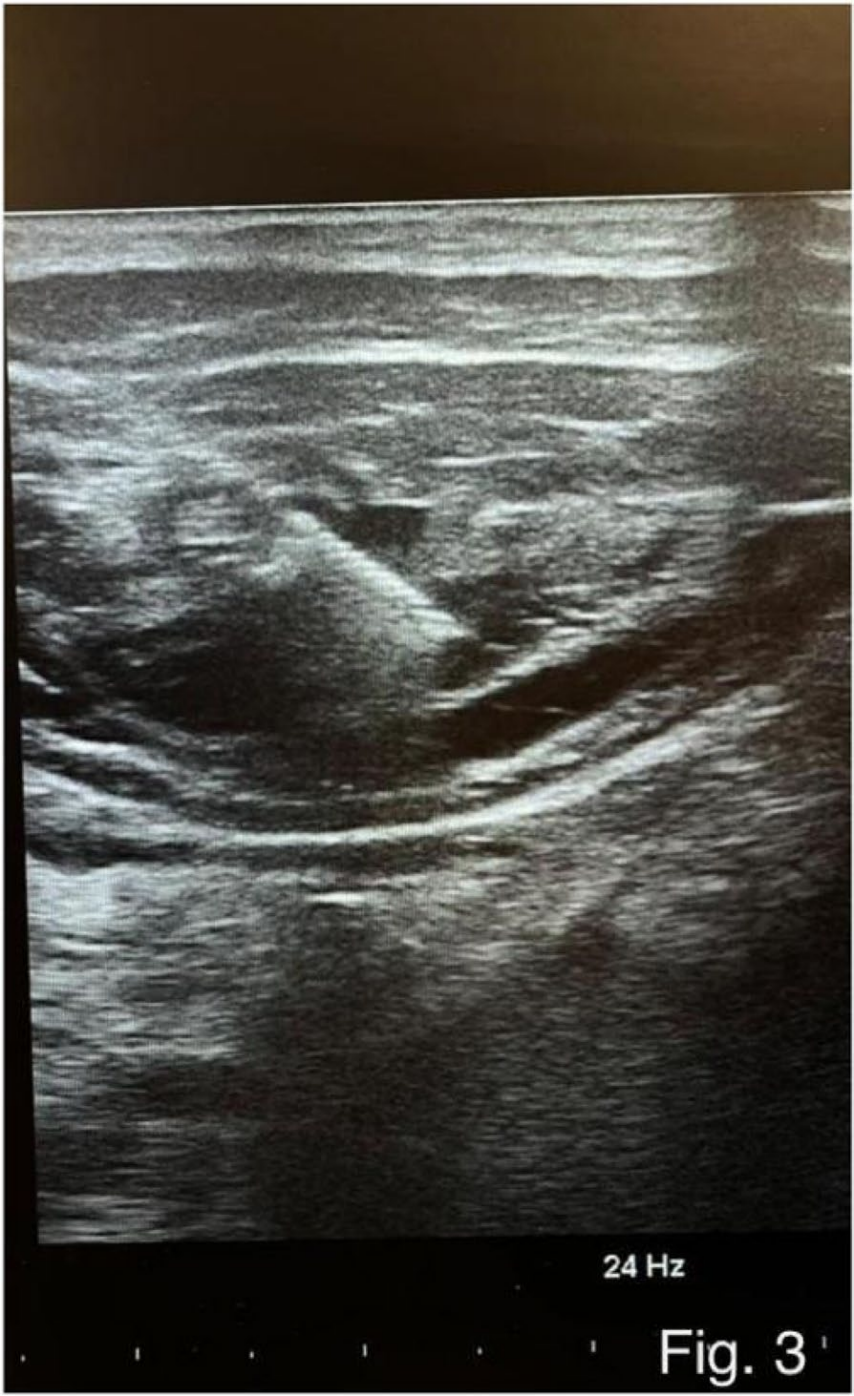
** (abstract A62).** Double TAP block (Lateral + Anterior)

### A63 Awareness neuraxial anesthesia vs general anesthesia in laparoscopic colon-rectal surgery: investigation of respiratory function through lung ultrasound

#### G. Secco^1^, B. Basta^2^, E. Bonvecchio^2^, F. Tiziana^2^, C. Magistro^3^, G. Marino^4^, D. Vailati^5^

##### ^1^Università di Milano, Milano, Italy; ^2^ASST Melegnano-Martesana, Vizzolo Predabissi, Italy; ^3^ASST Melegnano-Martesana, Vizzolo Predabissi, Italy; ^4^ASST Melegnano-Martesana, Vizzolo Predabissi, Italy; ^5^ASST Melegnano-Martesana, Vizzolo Predabissi, Italy

###### **Correspondence:** G. Secco

*Journal of Anesthesia, Analgesia and Critical Care 2024*, **4(1):**A63

**Background**

Neuraxial anesthesia (NA) and specifically thoracic spinal anesthesia (TSA) offers several theoretical advantages over General Anesthesia (GA) [1]. Specifically it is well known that mechanical ventilation can cause pulmonary complications. We present a case series to compare TSA with the gold standard GA in elective colorectal laparoscopic surgery evaluating the impact on respiratory functions through lung ultrasound (LUS).

**Materials and Methods**

We extracted data from the medical records of 30 consecutive patients undergone laparoscopic right and/or left colon resection surgery in Vizzolo-Predabissi Hospital-ASST Melegnano Martesana-Italy, who underwent NA or GA.

GA was performed according to iv Target Control Infusion (TCI) of Propofol and Remifentanyl. TCI effect compartment concentration (Ce) was achieved by Schnider's model for Propofol (Ce 1–2.5 ng/ml) and by Minto's model for Remifentanil (Ce 2–12 ng/ml), using infusion pumps. Adjustment of the infusion rate was realized in order to mantain the Bispectral Index between 40 and 60. Mechanical ventilation was performed using tidal volumes between 6 to 8 ml/kg and starting positive end espiratory pressure (PEEP) = 5 cm H2O; individualised PEEP was calculated in each patient according to best PEEP trial. FiO2 was maintained between 40 and 50%.

Spinal anesthesia was performed among T8 and T11 level, by a 25 Gauge pencil point (Sprotte) spinal needle. A total volume of 4 ml was injected, 10 mg (2 ml) of 0.5% Levo-bupivacaine with 10 mcg (2 ml) of dexmedetomidine. Sedation was obtained by continuous intravenous dexmedetomidine infusion. Oxygenation was optimized in order to obtain SpO2 > 95% using nasal cannula. Written informed consent was obtained with special regard to off-label intrathecal dexmedetomidine use.

Arterial blood gas (ABG) samplings were collected at baseline and at discharge from recovery room. Patients underwent LUS before the procedure and 2 h after the end, a quantitative LUS score was attributed [2].

Inclusion criteria: Age > 18 years, ASA (American Society of Anesthesiologists) status > 1 and METs (Metabolic Equivalent of Task) < 10.

Exclusion criteria: ASA = 1, METs >  = 10, Laparotomic or emergency surgery.

The study approved and registered on ClinicalTrial.gov with the identifier code NCT06360666.

**Results**

The population analysis did not reveal statistical differences between the two groups in terms of age, comorbidities, ASA class and METs (Table 1 and Fig. 1). The average duration of the surgical procedure was 194 ± 49.1 min (NA 180 ± 27.8 vs. GA 202.4 ± 63.7, p = 0.35). ABG parameters showed a reduction in the postoperative values of P/F ratio (NA 457.7 ± 123.8 vs GA 346 ± 53.8, p = 0.004) (Table 2). LUS reveals a higher prevalence of atelectasis in the postero-inferior regions of lung and after surgery showing a difference in the LUS score between the two groups after surgery procedure 4,8 ± 3,34 (NA 2 ± 1,6 vs 6 ± 3,2 GA, p = 0.04) (Fig. 2). In both groups, oxygen supply was not required in postoperative period.

**Conclusions**

TSA ensures effective anesthesiological coverage for laparoscopic abdominal surgery. Moreover, it appears to reduce lung atelectasis and its impact on respiratory exchanges.

**Fig. 1:**
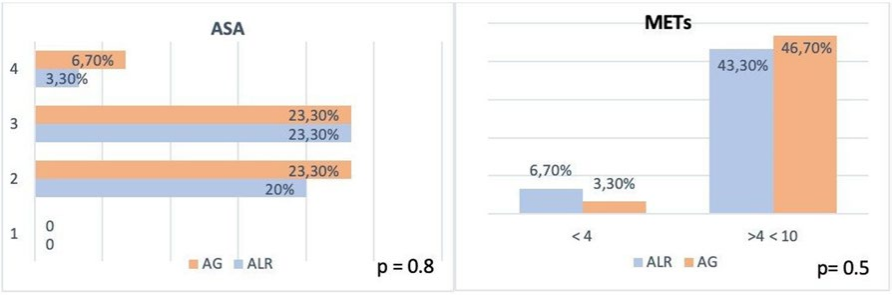
** (abstract A63).** ASA and METs scale distribution

**Fig. 2:**
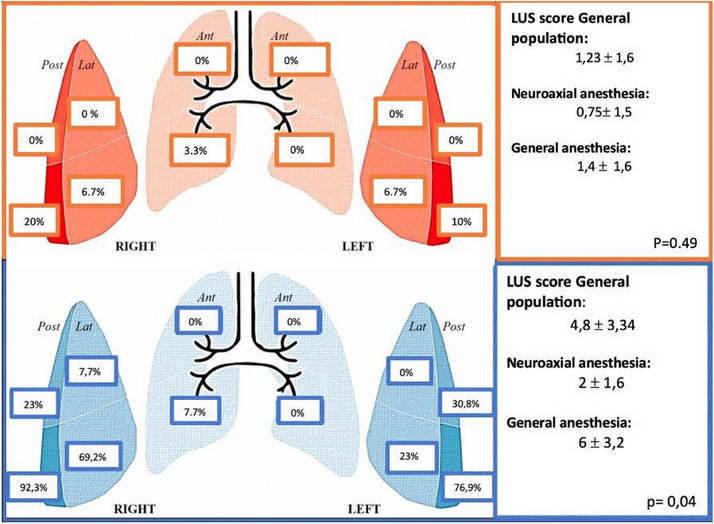
** (abstract A63).** LUS values and distribution before and after surgery

**Table 1:** **(abstract A63).** Population characteristics

	**General population (n 30)**	**Neuroaxial anesthesia (n 15)**	**General anesthesia (n 15)**	***P*-value**
*Age*	78 (75–82)	79 (73–85)	78 (74–82)	ns
*Sex (female)*	70%	80%	60%	ns
*BMI*	24,7 ± 4.1	25,4 ± 4.7	24 ± 3.3	ns
*Active smoke*	43.3% (13)	16.7% (5)	26,7% (8)	ns
*Hypertension*	73.3% (22)	73.3 (11)	73.3 (11)	ns
*CAD*	30% (9)	13.3% (2)	46.7% (7)	ns
*DM*	26.7% (8)	26.7% (4)	26.7% (4)	ns
*Stroke*	10% (3)	6.7% (1)	13.3% (2)	ns
*Atrial Fibrillation*	30% (9)	26.7% (4)	33.3% (5)	ns
*COPD*	13.3% (4)	13.3% (2)	13.3% (2)	ns
*Cognitive Impairment*	6.7% (2)	6.7% (1)	6.7% (1)	ns

*BMI* Body Mass Index, *CAD* Coronary artery disease, *DM* Diabetes Mellitus, *COPD* Chronic obstructive pulmonary disease, *ns* not significant

**Table 2:** **(abstract A63).** Arterial Blood Gas values

Arterial Blood Gas	General population (n 30)	Neuroaxial anesthesia (n 15)	General anesthesia (n 15)	*p*-value
*PH*	7.41 ± 0.05	7.39 ± 0.05	7.43 ± 0.04	ns
*pCO*_*2*_	36.5 ± 4.2	37.7 ± 4	35.2 ± 4.1	ns
*pO2*	79.8 ± 10.9	81.5 ± 11.6	77.8 ± 10.4	ns
*P/F*	380 ± 52.4	388 ± 55.2	371 ± 50	ns
*HCO*_*3*_*-*	23.6 ± 1.6	23.6 ± 1.6	23.6 ± 1.7	ns
*Lactate*	1.2 ± 0.36	1.24 ± 0.4	1.2 ± 0.33	ns
*Base Excess*	-0.89 ± 1.9	-0.98 ± 1.8	0.79 ± 2.1	ns
*PH after surgery*	7.38 ± 0.03	7.37 ± 0.03	7.39 ± 0.03	ns
*pCO*_*2*_* after surgery*	37.7 ± 4.1	38.7 ± 3.2	36.6 ± 4.8	ns
*pO2 after surgery*	108 ± 55.9	129.8 ± 62.7	85.4 ± 37.3	0.03
*P/F after surgery*	403.8 ± 110.6	457.7 ± 123.8	346 ± 53.8	0.004
*HCO*_*3*_*- after surgery*	22.2 ± 1.7	22.4 ± 1.5	22.1 ± 2	ns
*Lactate after surgery*	1.2 ± 0.36	1.14 ± 0.37	1.3 ± 0.36	ns
*Base Excess after surgery*	-2.7 ± 2.2	-2.4 ± 2.1	-2.9 ± 2.4	ns

### A64 General anesthesia vs regional anesthesia in anticoagulated patients with intertrochanteric fractures undergoing gamma nail fixation: a prospective study

#### E. Scalamandré^1^, M.B. Mascia^2^, M. Mazzocchi^3^, D. Passador^4^, E. Pariani^5^, A. Locatelli^6^

##### ^1^Università Degli Studi di Pavia, Pavia, Italy; ^2^IRCSS Policlinico San Matteo, Pavia, Italy; ^3^IRCSS Policlinico San Matteo, Pavia, Italy; ^4^IRCSS Policlinico San Matteo, Pavia, Italy; ^5^IRCSS Policlinico San Matteo, Pavia, Italy; ^6^IRCSS Policlinico San Matteo, Pavia, Italy

###### **Correspondence:** E. Scalamandré

*Journal of Anesthesia, Analgesia and Critical Care 2024*, **4(1):**A64

**Background**

Hip fractures are a major cause of morbidity and mortality among the elderly. Intertrochanteric and subtrochanteric hip fractures bear a 30-day mortality of 7.7% and a 1-year mortality of 26%. 1

Surgical delay is an independent risk factor for development of delirium in patients with hip fractures 2.

and most recent guidelines state that the anesthesiologist should facilitate surgery within 36 h from hip fractures.3

Subarachnoid anesthesia is currently the standard of care in most centers and recent studies suggest it is associated with lower postoperative morbidity and mortality 4. However, an increasing number of patients are either on anticoagulant or antiplatelet therapy (up to 30/40%) 3,5 and current ASRA guidelines recommend waiting from 72 h (for most NOAC) up to 10 days (for Prasugrel) to perform subarachnoid anesthesia safely. 6 It is therefore imperative to find an alternative to SA in patients undergoing hip fracture repair.

**Methods**

In this study we enrolled (after collection of informed consent) 9 patients on anticoagulant or antiplatelet therapy undergoing femur fixation with gamma nail, mean age 82.8, mean NHFS 5. 4 patients underwent General Anesthesia associated to analgesic PENG block (levobupivacaine 0.25% 20 ml + desamethasone 4 mg), while the other 5 received Regional Anesthesia associated to MAC (Monitored Anesthesia Care). The Peripheral nerve blocks performed in the RA group were Femoral Nerve Block (Ropivacaine 0.75% 20 ml), Gluteal Sciatic Nerve block (Lidocaine 1% 15 ml) and Lateral Femoro-Cutaneous Nerve Block (Lidocaine 1% 15 ml). All patients underwent fixation within 48 h from the event.

We then monitored these patients with respect to:

- Adequacy of anesthesia (including surgical satisfaction) and post-operative analgesia (measured with NRS scale and average opioid consumption)
- Intraoperative hemodynamic stability, assessed through non-invasive hemodynamic monitoring (Edwards-ClearSight™)
- Post operative complications (respiratory, cardiac, renal and neurological), focusing particularly on post-operative delirium. Dosage of hsTNI was acquired at 24 h and 48 h postoperatively, while significant renal complication was measured with > 50% increase of serum creatinine levels compared to baseline.

**Results**

Our results indicate that both techniques granted an adequate level of anesthesia and post operative analgesia, low 10-days complication rate and a comparable level of intraoperative hemodynamic stability. 1 patient in the RA group suffered from acute-on-chronic heart failure 24 h postoperatively.

**Conclusion**

Our cohort is limited, and further studies will be necessary to establish which between General anesthesia and Regional anesthesia is the best management for gamma-nail fixation when subarachnoid anesthesia isn’t an option.

Our results suggest Regional Anesthesia + MAC might be associated to lower impact on cognitive function (delirium is identified as the chief cause of post-operative mortality in hip fracture) and may allow for a better post-operative pain control.

On the other hand, this type of management requires greater operator skills and patient preparation time compared to GA.

### A65 Aiming high with regional anesthesia: a case report of a scapular region reconstructive surgery

#### L.M. Remore, F. Costa, M. Ricci, L. Pierantoni, A. Ruggiero, G. Pascarella, R. Cataldo, M. Carassiti, E.F. Agrò

##### Università Campus Bio-Medico di Roma, Roma, Italy

###### **Correspondence:** M. Ricci

*Journal of Anesthesia, Analgesia and Critical Care 2024*, **4(1):**A65

**Introduction**

The use of regional anaesthesia (RA) alone to provide intra and postoperative pain management in upper dorsal region reconstructive surgery has not yet been described.

We present the first reported case of RA for resection of a left scapular basalioma with dorsal intercostal artery perforator (DICAP) flap reconstruction in a patient presenting an untreated, high virulence HCV infection.

**Discussion**

A 62 year old male patient with an untreated, high virulence HCV infection contracted by a contaminated blood transfusion 20 years ago was presented for surgery.

Subarachnoid anesthesia was performed at T12-L1 with paramedian approach after US evaluation using a solution of 5 ml saline containing ropivacaine 10 mg, fentanyl 15 mcg, dexmedetomidine 5 mcg. Pin prick test confirmed anesthesia level at C6 without ROM affection. A bilateral ESP Block was performed at T6 using 30 ml of a 0,2% ropivacaine solution for each side. Sedation was administered during the surgery in prone position with midazolam 0,05 mg/kg and remifentanil 0,1 mcg/Kg/min.

**Results**

The surgery was performed without the need to administer extra opioids. Registered NRS was 0 at both PACU and 6 h post-op. Maximum registered NRS during hospital stay was 3, no opioid was administered post-op and the patient has been home discharged two days later.

**Conclusions**

RA is a promising strategy for intra and postoperative pain control for upper dorsal reconstructive surgery. We look forward to compare RA vs GA in a case–control study.

Informed consent was obtained.

**Fig. 1:**
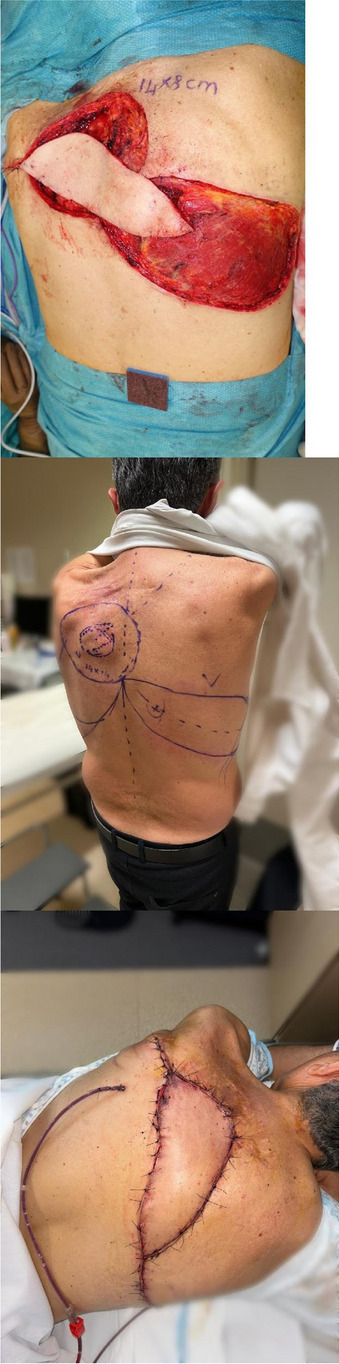
** (abstract A65).** See text for description

### A66 Ultrasound evaluation reduces the incidence of difficult spinal anesthesia: a prospective observational study

#### F. Costa^1^, M. Ricci^1^, F. Gargano^1^, L.M. Remore^1^, A. Strumia^1^, D.B. Romualdo^2^, A. Ruggiero^1^, A. Hazboun^1^, G. Pascarella^1^, M. Carassiti^1^, R. Cataldo^1^, E.F. Agrò^1^

##### ^1^Università Campus Bio-Medico di Roma, Roma, Italy; ^2^Humanitas Gavazzeni, Bergamo, Italy

###### **Correspondence:** M. Ricci

*Journal of Anesthesia, Analgesia and Critical Care 2024*, **4(1):**A66

**Introduction:**

Spinal anesthesia (SA) is a blind technique based on anatomical landmarks consisting in the injection of a local anesthetic in the subarachnoid space. Although it is considered a safe procedure, it may give complications including headache and spinal hematoma, whose incidence increases in difficult SA characterized by a higher number of attempts.

**Objective of the study:**

Our aim was to analyze the impact of pre-procedural Ultrasound (US), in both general population and those with predicted difficult SA, in reducing the incidence of difficult SA, defined as the need for a second skin puncture in the same intervertebral space or in another one.

**Materials and Methods:**

Data were collected using an anonymous questionnaire completed by the operator performing the block. The questionnaire consisted of 3 sections; the first evaluated the presence of difficult SA predictors using the neuraxial block assessment (NBA) score. This score includes the following items: visibility (3 points) and/or palpability (5 points) of the spinous processes, column deformities (5 points), history of difficult SA (4 points). An NBA score > 6 indicates a high probability of difficult SA.

The second section examined whether or not the operator used preprocedural US. The third section looks at the difficulty of SA, the experience of the operator and patients’ position.

**Results:**

We enrolled 824 patients. Among them, 382 underwent preprocedural US evaluation and 442 did not.

US assisted SA was associated with a significant lower risk of failure (1.6% vs. 8.1%) and difficult procedure (13% vs. 87%); p < 0.001. A subgroup analysis was performed on 400 patients with difficult SA predictors (NBA score > 6). In this case, the difference in failed SA between US assisted and blind procedures was even greater (1.6% vs. 16.2%, respectively); p < 0.001. A similar trend was observed for the incidence of difficult SA (15% vs. 41.8); p < 0.001.

**Conclusions:**

Ultrasound evaluation can significantly reduce the incidence of failed and difficult spinal anesthesia, especially in those patients with predicted difficult SA. This may lead to save time, increase patient comfort and reduce the risk of complications.

Informed consent was obtained.

### A67 Intervention delay and postoperative after femur surgery: don't put off until tomorrow what you can do today

#### A. Poles^1,2^, F. Matteo^1,2^, C. Matteo^1,2^, C. Maura^1,2^, M. Federica^1,2^, G. Maria^1,2^, M. Giulia^1,2^, T. Gabriella^2^, B. Tiziana^1,2^

##### ^1^Department of Medicine (DMED), University of Udine, Udine, Italy; ^2^Department of Anesthesia and Intensive Care Medicine, ASUFC University Hospital of Udine, Udine, Italy

###### **Correspondence:** A. Poles

*Journal of Anesthesia, Analgesia and Critical Care 2024*, **4(1):**A67

**Background**

Timing is one of the most important elements in the management of patients with hip-fracture; clinical guidelines recommend reparative surgery within 24–48 h from the hospital admission [1]. Surgical delay is associated with an increase in perioperative complications therefore reducing it, may help to prevent complications and mortality [2,3]. At the same time, appropriate care and multimodal approach (e.g. early mobilization, medication optimization, adequate coverage with analgesia, appropriate management of osteoporosis and nutritional assessment) are associated with better outcomes, especially in older adults [4].

This single-center retrospective study is aimed at evaluate whether the length of postoperative hospital stay is affected by any delay in surgery timing.

**Materials and Methods**

Inclusion criteria referred to patients aged more than 65 years who underwent urgent surgery for hip-joint fracture, managed with loco-regional anesthesia (epidural catheter, spinal anesthesia or peripheral nerve block). Patients were enrolled at Santa Maria della Misericordia hospital, Udine, Italy, between January 1st and May 31st 2023. Patients who suffered high energy trauma like explosion, precipitation or road crashes and polytraumatic injuries (fractures in more than two body districts) were rejected.

**Results**

Eligibility criteria were met by 156 patients. Mortality was about 9 patients (5.77%) overall. Further stratification of the population showed that 3 patients (1.92%) died within the first month from surgery, whereas 6 more patients (3.85%) died between 31 and 60 days from surgery.

Considering only the 147 patients who survived the operation, the mean trauma-to-surgery delay was of 4.03 ± 2.93 days with a mean postoperative recovery of 14.9 ± 7.1 days (Figs. 1 and 2). Spearman’s rank correlation analysis between trauma-to-surgery delay and postoperative recovery showed a p-value of 0.028.

Patients who died within the first month from surgical intervention, underwent surgery at 3.67 ± 2.08 days after trauma, with a mean postoperative recovery of 21.7 ± 11.0 days. Patients who died between 31 and 60 days from surgical intervention underwent surgery at 5.17 ± 3.06 days after hospital admission with a mean postoperative recovery of 20.2 ± 10.2 days.

**Conclusions**

According to our data, in the specific setting of hip-fracture, an increased delay between hospital admission and surgery is associated with a prolonged postoperative hospital stay (p < 0.05).

Second, mortality appears to double up during the second moth after surgery, when compared with the first month (6 patients versus 3); the consensual increase in trauma-to-surgery interval between the two subgroups (3.67 days versus 5.17 days) might show once more, accordingly to the known literature [5], that any delay in surgery could be associated with increased mortality.

Further, the hospital recovery appears to be similar in both subgroups of patients who died after surgery (21.7 days by mean, in whom died within the first month; 20.2 days by mean in whom died during the second month) but significantly longer than patients who survived surgery (14.9 days by mean). A postoperative recovery over than 15 days could be a predictor for in-hospital death after hip-surgery. Further analysis are thus necessary.

The consent was waived due to retrospective nature of the study.

**Fig. 1:**
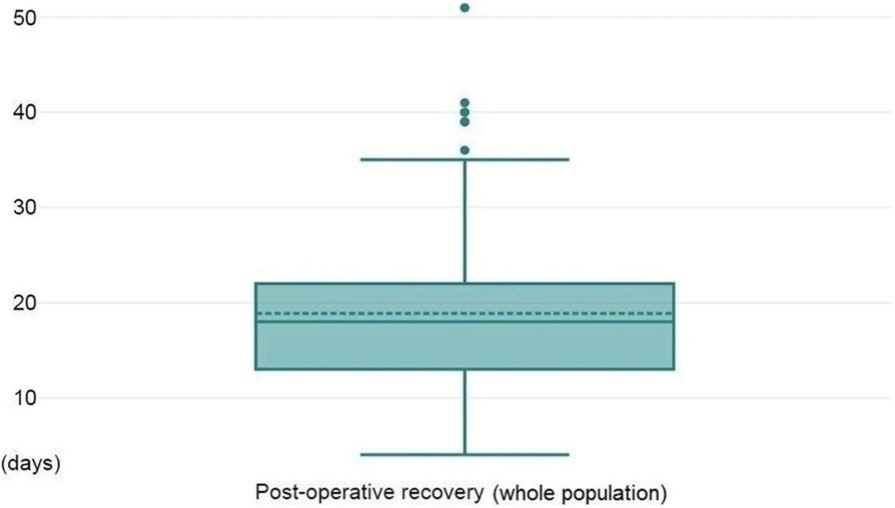
** (abstract A67).** Postoperative recovery length after surgery

**Fig. 2:**
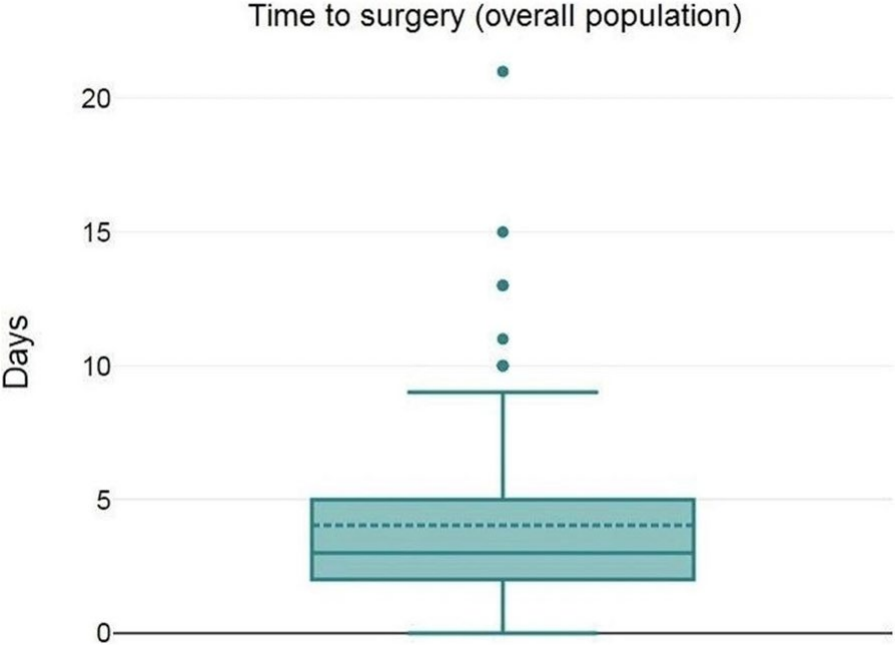
** (abstract A67).** Trauma-to-surgery interval

### A68 Post-operative analgesia in total hip arthroplasty surgeries: pericapsular nerve group block vs fascia iliaca block

#### N. D'Abrunzo^1^, I. Piccione^1^, D. Cirillo^1^, M.S. Barone^1^, G. Ranieri^2^, A. Coviello^1^

##### ^1^Department of Neurosciences, Reproductive and Odontostomatological Sciences, University of Naples Federico II, Napoli, Italy; ^2^Ospedale Isola Tiberina, Gemelli Isola, Roma, Italy

###### **Correspondence:** N. D'Abrunzo

*Journal of Anesthesia, Analgesia and Critical Care 2024*, **4(1):**A68

**Background**

The rise in average life expectancy and lifestyle-related diseases has led to an increase in total hip arthroplasty (THA) surgeries[1]. Effective postoperative pain management is essential for optimizing rehabilitation and minimizing complications[2,3]. Recent advancements in loco-regional anesthesia techniques, such as the Pericapsular Nerve Group (PENG) block, offer potential improvements over traditional methods like the Fascia Iliaca (FI) block[4].

This study aims to compare the efficacy of the PENG block with the FI block in managing postoperative pain in patients undergoing THA.

**Methods**

We conducted a prospective comparative study at the Department of Neuroscience and Reproductive and Odontostomatological Sciences at the University of Naples Federico II.

From May 2023 to February 2024, 30 patients scheduled for elective primary THA due to osteoarthritis or other degenerative conditions, with an American Society of Anesthesiologists physical status classification between II and III, were enrolled.

After obtaining informed consent, patients were clustered into two groups: one group received the FI block with a mixture of 0.5% ropivacaine and 4 mg dexamethasone in 20 mL and the other the PENG block with a mixture of 0.25% ropivacaine and 4 mg dexamethasone in 40 mL. After performing the loco-regional anesthesia, midazolam was administered at a dose of 0.025–0.5 mg/kg IBW to achieve a Richmond Agitation-Sedation Scale (RASS) score of -1 or 0. Pain levels were assessed using the Numeric Rating Scale (NRS) at various postoperative intervals (immediately after surgery, and at 6, 12, 18, and 24 h). Data on analgesic consumption, side effects, and mobility were also collected.

**Results**

Patients in the PENG block group exhibited significantly lower pain scores compared to those in the FI block group in the first 24 h post-surgery (Table 1). The mean NRS score was 2.1 in the PENG block group versus 3.5 in the FI block group immediately after surgery (p < 0.05). At 24 h, the scores were 1.2 for PENG and 2.8 for FI (p < 0.05). However, there was no significant difference in the consumption of analgesics between the two groups throughout the study period. No major complications or side effects were reported, affirming the safety of both techniques.

**Conclusions**

The PENG block demonstrates a superior efficacy in reducing immediate postoperative pain in THA patients compared to the FI block. The lower pain scores associated with the PENG block may contribute to faster initial recovery and reduced hospital stay, though both blocks were effective and safe for postoperative pain management. Further studies with larger sample sizes and long-term follow-ups are recommended to confirm these findings and to assess the impact of pain management on rehabilitation outcomes.

**References**

1. Hasan K, Shankar S, Sharma A, Carter A, Zaidi R, Cro S, Skinner J, Goldberg A. Hip surgery and its evidence base: progress over a decade? J Orthop Traumatol. 2016 Dec;17(4):291–295. 10.1007/s10195-016-0421-z. Epub 2016 Jul 21. PMID: 27443626; PMCID: PMC5071242;
2. ⁠Anger M, Valovska T, Beloeil H, Lirk P, Joshi GP, Van de Velde M, Raeder J; PROSPECT Working Group* and the European Society of Regional Anaesthesia and Pain Therapy. PROSPECT guideline for total hip arthroplasty: a systematic review and procedure-specific postoperative pain management recommendations. Anaesthesia. 2021 Aug;76(8):1082–1097. 10.1111/anae.15498. Epub 2021 May 20. PMID: 34015859;
3. Zhang B, Rao S, Mekkawy KL, Rahman R, Sarfraz A, Hollifield L, Runge N, Oni JK. Risk factors for pain after total hip arthroplasty: a systematic review. Arthroplasty. 2023 Apr 3;5(1):19. 10.1186/s42836-023-00172-9. PMID: 37009894; PMCID: PMC10069042;
4. ⁠Girón-Arango L, Peng PWH, Chin KJ, Brull R, Perlas A. Pericapsular Nerve Group (PENG) Block for Hip Fracture. Reg Anesth Pain Med. 2018 Nov;43(8):859–863. 10.1097/AAP.0000000000000847. PMID: 30063657.

**Table 1:** **(abstract A68).** Patient in the first 24 h post-surgery

*N* = 30	VAS score					PONV					Urinary retention				
	**FICB (N = 15)**		**PENG (N = 15)**		**UMW**	**FICB (N = 15)**		**PENG (N = 15)**		**χ**^**2**^	**FICB (N = 15)**		**PENG (N = 15)**		**χ**^**2**^
**Time**	**Median**	**IQR**	**Median**	**IQR**	***p***	***N***	***%***	***N***	***%***	***p***	***N***	***%***	***N***	***%***	***p***
*Surgery end*	0.00	0.00–0.00	0.00*	0.00–0.00	*0.350*	0	0%	0	0%	^*§*^	0	0%	0	0%	^*§*^
*6 h*	0.00	0.00–5.00	0.00	0.00–1.00	*0.554*	0	0%	0	0%		0	0%	0	0%	
*12 h*	0.00	0.00–0.00	1.00*	0.00–4.00	*0.0057*	0	0%	0	0%		0	0%	0	0%	
*18 h*	0.00	0.00–0.00	0.00	0.00–2.00	*0.142*	0	0%	0	0%		0	0%	0	0%	
*24 h*	0.00	0.00–0.00	1.00*	0.00–3.00	*0.022*	0	0%	0	0%		0	0%	0	0%	
*p*	*0.0051*		0.024*			^*§*^			^*§*^		^*§*^		^*§*^		
	**Antiemetic request**					**Analgesic request**					**Block recovery**				
	**FICB (N = 15)**		**PENG (N = 15)**		**χ**^**2**^	**FICB (N = 15)**		**PENG (N = 15)**			**FICB (N = 15)**		**PENG (N = 15)**		**χ**^**2**^
**Time**	***N***	***%***	***N***	***%***	***p***	***N***	***%***	***N***	***%***	***p***	***N***	***%***	***N***	***%***	***p***
*Surgery end*	0	0%	0	0%	^*§*^	0	0%	0	0%	^*§*^	0	0%	0	0%	^*§*^
*6 h*	0	0%	0	0%		0	0%	0	0%		0	0%	0	0%	
*12 h*	0	0%	0	0%		0	0%	0	0%		0	0%	0	0%	
*18 h*	0	0%	0	0%		0	0%	0	0%		0	0%	0	0%	
*24 h*	0	0%	0	0%		0	0%	0	0%		0	0%	0	0%	
*p*	^*§*^		^*§*^			^*§*^		^*§*^			^*§*^		^*§*^		
	**Time to personal care recovery**					**Other postoperative Outcomes**									
	**FICB (N = 15)**		**PENG (N = 15)**		**χ**^**2**^						**FICB (N = 15)**		**PENG (N = 15)**		**UMW**
**Time**	***N***	***%***	***N***	***%***	***p***		**Variable**				***Median***	***IQR***	***Median***	***IQR***	***p***
*Surgery end*	0	0%	0	0%	^*§*^		RASS score				0.00	0.00–0.00	0.00	0.00–0.00	°
*6 h*	0	0%	0	0%			Hollmen score				4.00	4.00–4.00	4.00	4.00–4.00	0.350
*12 h*	0	0%	0	0%			Bromage score				1.00	1.00–1.00	1.00	1.00–1.00	°
*18 h*	0	0%	0	0%											
*24 h*	0	0%	0	0%							***Mean***	***SD***	***Mean***	***SD***	***p***
*p*	^*§*^		^*§*^				Postoperative Hb (g/dL)				10.67	1.60	11.57	1.34	0.106
							Hb variation (g/dL)				2.79	1.23	2.13	1.06	0.123
							Surgery duration (min)				104.00	30.31	112.67	34.12	0.468
							Time to recovery (min)				427.33	210.76	494.87	128.26	0.300

### A69 Gluteal acute compartment syndrome in a patient undergoing neuraxial anesthesia: a case report

#### I. Piccione^1^, G. D'Agostino^2^, M.S. Barone^1^, G. Ranieri^3^, C. D'Errico^4^, N. D'Abrunzo^1^, D. Cirillo^1^, A. Coviello^1^

##### ^1^Department of Neurosciences, Reproductive and Odontostomatological Sciences, University of Naples Federico II, Napoli, Italy; ^2^Department of Anesthesiology and Critical Care, Ospedale San Giuliano, Giugliano in Campania (NA), Italy; ^3^Ospedale Isola Tiberina, Gemelli Isola, Roma, Italy; ^4^Anesthesia service in multi-specialty surgery, TIPO OTI, AORN Antonio Cardarelli, Napoli, Italy

###### **Correspondence:** I. Piccione

*Journal of Anesthesia, Analgesia and Critical Care 2024*, **4(1):**A69

**Background**

Acute compartment syndrome (ACS) is defined as increased pressure within a muscle fascial compartment which diminishes perfusion pressure, subsequently leading to local tissue ischemia and hypoxia [1, 2]. The syndrome is a potential limb and life-threatening complication following trauma if diagnosis is missed and left untreated [3]. The occurrence ACS in the context of elective surgeries is rare but significant, with an incidence reported as 3.1 per 100,000 annually. The incidence of ACS is notably higher in individuals with multiple risk factors such as those present in this case: male sex, hypotension, obesity, reduced mobility, and prolonged surgical duration [4]. Current diagnosis of ACS is based on clinical findings and intramuscular pressure (IMP) measurement and is targeted at identifying safe thresholds for when fasciotomy can be avoided [5]. Literature suggests that nerve block-induced insensitivity might delay ACS diagnosis by masking symptoms like severe pain [6, 7]. In this case emphasizes the poor response to escalating pain postoperatively facilitated an earlier suspicion and investigation into ACS, contradicting common concerns about diagnostic delays.

**Case report**

The patient of our clinical case report is a 69-year-old male with a complex medical history including well-controlled hypertension, obesity, senile dementia, HBV, psoriatic arthritis, poliomyelitis, and right knee arthrosis underwent a right total knee arthroplasty (TKA). A combined spinal-epidural anesthetic was successfully administered without complications, achieving a sensory block with pin prick and ice test was achivied from T8 to S4 (Holman scale 4) and the motor block from T12 to S4 (Bromage scale 1). The full mobility was achivied within postoperative first hour. Postoperative pain management carried out with epidural continue infusion of Ropivacaine 0,2%—8 ml per hour. After the initial 24 postoperative hours, the patient reported a pain level of 9/10 on the VAS pain scale, which was treated with a bolus of Ropivacaine 0.2% at 10 ml (administered three times every one hour). The VAS pain scale after the rescue dose was 7/10. Despite the inadequate response to the epidural bolus, it confirmed the accurate placement of the epidural catheter while also raising suspicion of ACS. Further clinical monitoring revealed swelling and redness in the gluteal region (Fig. 1). Simultaneously, laboratory data showed elevated creatine phosphokinase levels. The intercompartment pressure of 28 mmHg, along with diagnostic imaging confirming edema in the muscle compartments, supported this suspicion. However, fasciotomy was avoided due to spontaneous symptom resolution.

**Conclusions**

The incidence of ACS has been documented to be 7.3 per 100,000 men and 0.7 per 100,000 women. Despite this, diagnosing ACS is rarely straightforward and requires a high index of suspicion and clinical awareness. Delayed diagnosis of ACS can have deleterious consequences. To our knowledge, the possibility of masking ACS after peripheral nerve blocks for limb injuries is still controversially discussed. Despite the existing literature, our study demonstrates that epidural anesthesia has contributed to accelerating the diagnostic process rather than masking it due to sensory block. Our findings challenge the conventional belief and underscore the valuable role of epidural anesthesia in facilitating accurate diagnosis.

Informed consent was obtained.

**References**

1. Tiwari, A., Haq, A. I., Myint, F., & Hamilton, G. (2002). Acute compartment syndromes. The British journal of surgery, 89(4), 397–412.
2. Raza H, Mahapatra A. Acute compartment syndrome in orthopedics: causes, diagnosis, and management. Adv Orthop. 2015;2015:543412.
3. Agius, C., & Cole, E. (2021). Acute compartment syndrome (ACS)—a case of delayed diagnosis. International journal of orthopaedic and trauma nursing, 42, 100845.
4. Shadgan B, Pereira G, Menon M, Jafari S, Darlene Reid W, O'Brien PJ. Risk factors for acute compartment syndrome of the leg associated with tibial diaphyseal fractures in adults. J Orthop Traumatol. 2015;16(3):185–192.
5. Rorabeck C. H. The treatment of compartment syndromes of the leg. Journal of Bone and Joint Surgery. 1984;66(1):94–97.
6. Hilber N, Dodi A, Blumenthal S, Bruppacher H, Borgeat A, Aguirre J. The Impact of Regional Anesthesia in Masking Acute Compartment Syndrome after Limb Trauma. Journal of clinical medicine. 2024;13(6):1787.
7. Tran AA, Lee D, Fassihi SC, Smith E, Lee R, Siram G. A systematic review of the effect of regional anesthesia on diagnosis and management of acute compartment syndrome in long bone fractures. Eur J Trauma Emerg Surg. 2020;46(6):1281–1290.

**Fig. 1:**
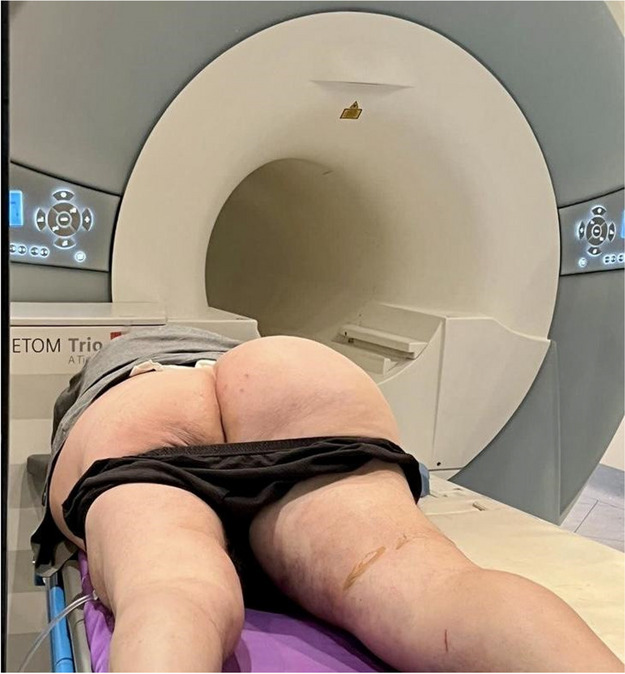
** (abstract A69).** Evidence of swelling and redness in the gluteal region

### A70 The use of intrathecally atropine in Enhanced Recovery After Surgery (ERAS) in gynecologic oncology

#### D. Conti^1^, F. Perelli^2^, G. Eleonora^1^, C. Stera^1^, F. Bianchi^1^, E. Fera^2^, P. Cecilia^1^, M. Pazzi^1^, S. Pisaneschi^2^, F. Ioia^3^, M. Giusti^2^, A. Mattei^2^, V. Pavoni^1^

##### ^1^Emergency Department and Critical Care Area, Anesthesia and Intensive Care Unit, Santa Maria Annunziata Hospital, Firenze, Italy; ^2^Department of obstetrics and gynecology, Santa Maria Annunziata Hospital, Firenze, Italy; ^3^Department of Nursing science, Santa Maria Annunziata Hospital, Firenze, Italy

###### **Correspondence:** D. Conti

*Journal of Anesthesia, Analgesia and Critical Care 2024*, **4(1):**A70

**Background**—Atropine is a competitive, reversible antagonist of muscarinic receptors with multiple clinical uses. (1,2) However, the benefit of intrathecally atropine administration in term of reducing postoperative pain and nausea and vomiting (PONV) in the context of ERAS in gynecologic oncology have not been evaluated.

**Methods** – In the study patients undergoing ERAS laparoscopic surgery for ovarian cancer from January 2022 to March 2023 was retrospectively considered. In all patients neuraxial analgesia before general anaesthesia induction was effected. The sample was divided into two study groups. In the first only intratecal morphine 60–70 µg with Levobupivacaine 2,5 mg was administered (group 1 N = 34), in the second as well as the opioid and local anaesthetic also atropine 0,1 mg was added (group 2 N = 29). In both groups PONV prophylaxis with two or three antiemetic drugs as guided by the Apfel score (3), is routinely administered. Acetaminophen (8 mg/kg every 8 h postoperative), and non-steroidal anti-inflammatory drugs are also used in all patients.

**Results** – The two study groups were omogeneous for demographic characteristics, and adverse events. (p = NS for all topic). (Fig. 1) Group 2 showed a trend towards a lower PONV (23,52% vs 9.09% p 0.11). Furthermore in both groups no differences in terms of numeric rating scale (NRS) score at 8,16,24,32,40,48 h were recordered. (respectively, p = 0.25, 0.23, 0.26, 0.42, 0.64, 0.44).

**Conclusions** – Although preliminary data were processed, the intrathecally atropine administration seems to favor a trend in lower PONV. However, a larger sample size is required for a definitive conclusion on this topic.

Informed consent was obtained.

**References**

1. McLendon K, Preuss CV. Atropine. 2023 Jun 23. In: StatPearls [Internet]. Treasure Island (FL): StatPearls Publishing; 2023 Jan–. PMID: 29262018.
2. Baciarello M, Cornini A, Zasa M, Pedrona P, Scrofani G, Venuti FS, Fanelli G. Intrathecal atropine to prevent postoperative nausea and vomiting after Cesarean section: a randomized, controlled trial. Minerva Anestesiol. 2011 Aug;77(8):781–8. PMID: 21730925.
3. Choy R, Pereira K, Silva SG, Thomas N, Tola DH. Use of Apfel Simplified Risk Score to Guide Postoperative Nausea and Vomiting Prophylaxis in Adult Patients Undergoing Same-day Surgery. J Perianesth Nurs. 2022 Aug;37(4):445–451.

**Fig. 1:**
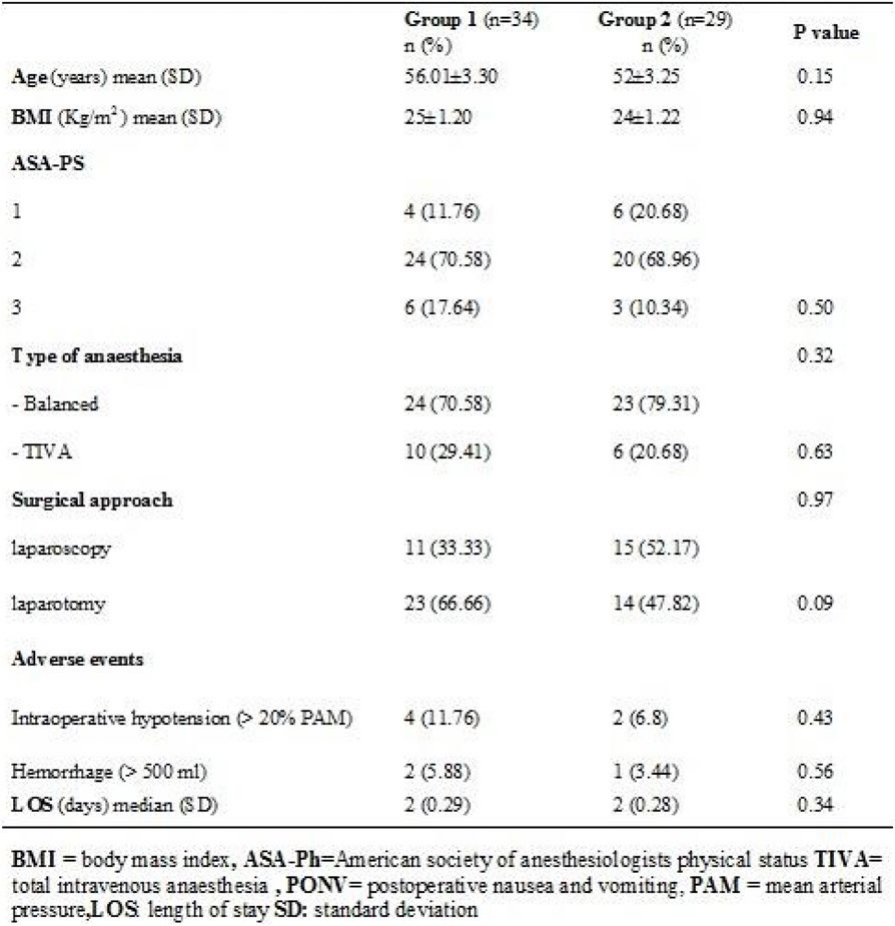
** (abstract A70).** Descriptive characteristics, type of surgery and adverse events of the two study groups

### A71 PENG block for managment of neck hip fracture: a case report on eldery women with high aortic stenosis

#### G.C. Papa^1^, F. Bergamini^2^, F. Lentini^2^, G. Razionale^2^, C. Capra^3^

##### ^1^Department of Anaesthesiology, Reanimatology, Intensive Care Medicine and Pain Therapy, Resident Physician Doctor—Univ, milano, Italy; ^2^ Department of Anaesthesiology and Pain Therapy ASST ovest Milan, Magenta, Italy; ^3^ Department of Anaesthesiology, Reanimatology and Intensive Care Medicine, ASST ovest—MIlan, Magenta, Italy

###### **Correspondence:** G.C. Papa

*Journal of Anesthesia, Analgesia and Critical Care 2024*, **4(1):**A71

**Background**

Femoral neck fracture is the most frequent cause of surgery in elderly patients. Cannulated screw fixation surgery can be performed under neuraxial anesthesia, loco-regional anesthesia or general anesthesia. In severe geriatric valvulopathic patients, anesthesia management with peripheral blocks combined with ketamine sedation should be preferred.

**Material And Methods**

A 90-year-old female patient presented left femoral neck fracture and an underlying aortic steno-insufficiency, with prevalent severe aortic stenosis (transvalvular gradient of 57 mmHg) and mild insufficiency, she had a PM implant for severe bradycardia, and was in home therapy with cardioaspirin, her BMI was 19.53 kg/m2 and an ASA score of 4.

The preoperative blood tests resulted as follows: hemoglobin 12.6 g/dL, platelets 136,000, PT 0.91, aPTT 1.01, glucose 100 mg/d, and inflammatory markers within normal range. Renal and hepatic function also in normal range.

The degree of aortic stenosis discouraged neuroaxial or general anesthesia, therefore, we decided to perform a PENG block (pericapsular nerve group) associated with femoral nerve block using ENS (Electric Nervous Stimulation) and ultrasound-guided technique. We administered carbocaine 1.5% 20 ml and ropivacaine 0.375% 16 ml. During the procedure, the patient was sedated with an initial bolus of ketamine 20 mg (0.25 mg kg-1) and midazolam 1 mg. We administered omeprazole 40 mg, dexamethasone 4 mg and ibuprofen 400 mg for pain control. During surgery, the patient did not complain of pain, she did not present any psychomotor agitation and always maintained stable hemodynamic parameters. At the time of cephalic screw placement, the patient complained of pain therefore it was necessary to perform a second bolus of ketamine 20 mg with immediate benefit. At the end of the surgical procedure the patient was taken to the ward where no antalgic therapy was required throughout the next 12 h.

**Conclusion**

Our case demonstrates that PENG block combined with ketamine sedation allows management of cannulated screw fixation in elderly valvulopathic patients while maintaining good control of hemodynamic parameters, without high risk of complications. The patient complained of pain only during cephalic screw placement, which was easily managed with a ketamine bolus administration.

Informed consent was obtained.

### A72 Clavicle surgery: superficial clavipectoral and cervical fascia blocks, in combination with dexmedetomidine

#### S. Limpido, G. Bruno, R. Miccio, M. Iannazzone

##### Ospedali Riuniti Penisola Sorrentina, Sorrento, Italy

###### **Correspondence:** S. Limpido

*Journal of Anesthesia, Analgesia and Critical Care 2024*, **4(1):**A72

**Backgrounds:**

The traditional locoregional anesthesia technique for clavicle fracture surgeries is the association of interscalene block with superficial cervical plexus block under ultrasound guidance. This combination can be effective in clavicle fracture surgery, but interscalene brachial plexus block is prone to complications such as diaphragmatic paralysis and phrenic nerve block or Horner's syndrome. Therefore our study explored a novel effective and safe locoregional anesthesia technique for clavicle fracture surgeries, avoiding the complications of general anesthesia and interscalene brachial plexus block: The association of clavipectoral fascial plane block combined with superficial cervical plexus block under ultrasound vision, associated with analgosedation with dexmetodimine.

**Methods**:

Selected 42 pcs with unilateral clavicle fracture (ASA I-IV).

7 ml of ropipvacaine 0.56 was injected for the superficial cervical plexus under ultrasound vision. A further 10 ml was injected at the fracture rim. They were then injected in equal parts, totalling 15 ml of 0.56% ropivacaine, medially and laterally to the fracture rim. At the start of surgery, once the level of anesthetic plan suitable for all patients was obtained, dexmedetomidine was administered as a continuous infusion at 1mcg/kg/min.

**Results:**

The efficacy of the anesthetic plan was evaluated with a pinprick test 30 min after the execution of the block. The level of intraoperative sedation assessed with RASS scale The postoperative pain assessment was assessed with VAS visual analogue scale at 6, 12 and 24 h. No adverse reactions such as systemic toxicity to local anesthetics, nerve injury, pneumothorax, hemothorax, diaphragmatic paralysis, or peridural anesthesia were recorded.

**Conclusions:**

Clavipectoral fascia block combined with superficial cervical plexus block, in association with analgosedation with dexmedetomidine, is an easy-to-perform, safe and effective technique for achieving the desired anesthetic plane for surgery and for postoperative pain control in clavicle surgery.α

Informed consent was obtained.

### A73 Erector Spinae Plane (ESP) bilateral thoracic ultrasound-guided block versus wound infiltration in instrumented spinal surgery: preliminary results of a prospective study

#### F. Merciai^1^, A. Iuorio^1^, G. Gargiulo^1^, G. Torretta^1^, M. Limone^1^, A. Sibilio^1^, P. Fusco^1^, A. Persico^1^, S. Ingino^1^, F. Blasio^1^, P. Donnarumma^1^, A. Rapana'^1^, A. Storti^1^, A. Alfieri^2^

##### ^1^AORN San Giuseppe Moscati, Avellino, Italy; ^2^AORN Cardarelli, Napoli, Italy

###### **Correspondence:** F. Merciai

*Journal of Anesthesia, Analgesia and Critical Care 2024*, **4(1):**A73

Background

Posterior spinal surgery is usually followed by a difficult management of postoperative pain [1]. The Erector Spinae Plane block (ESPb) is a paraspinal interfacial plane block targeting the dorsal and ventral rami of spinal nerves used for perioperative pain management [2].

The aim of the study was to investigate the effectiveness of ESP block for pain control in patients undergoing instrumented spinal surgery compared by pain scores using the Numeric Rating Scale (NRS: NRS 0 = no pain, 10 = worst pain imaginable). Secondary outcomes were intra-operative opioids consumption, rescue analgesia dose and incidence of side effects up to 72 years after surgery.

Material and methods

This is a prospective, observational, single-center study which will compare patients undergoing instrumented spinal surgery at Moscati hospital in Avellino [Fig 1].

Patients will be enrolled prospectively and will be randomly divided into two groups based on the treatment received: Esp-Block (group (E) and Wound Infiltration (group (I).

Inclusion criteria are:

- patients aged between 18 and 70;
- posterior instrumented spinal surgery (> or equal to 2 levels);
- ASA I—II – III.

The sample size was calculated assuming a power of 0.80 and a significance level of 0.05. With these parameters and considering the worst standard error condition with 50% additional analgesic dose request for the control group, we estimated that a total of 89 patients would lead to statistically significant results for differences greater than 26%.

In the case of patients treated with ESP Block (group E), once the patient has been induced and pronated, we proceed with the block by administering, bilaterally, a 0.375% ropivacaine solution in a volume of 0.3 ml/kg.

In the case of patients treated with wound infiltration (group I), at the end of the surgical procedure, perifascial infiltration of the wound is performed with ropivacaine 0.375%.

The statical model included as dependent variables the pain scores reported by patients at different times (pre-treatment, post-treatment, 30 min, 2 h, 12 h, 24 h, 48 h and 72 h after treatment). The main independent variable was the type of treatment received.

Results

The model parameters indicate that the intercept, representing the mean pain score in the ESP-block group, is significantly different from zero (t-value = 7.52, p < 0.001), indicating that there is a difference in pain scores before the treatment. Furthermore, the coefficient for the Wound Infiltration treatment is significantly different from zero (t-value = 2.15, p = 0.045), suggesting that there is a significant difference in pain scores between the two treatments.

The correlation between the intercept and Wound Infiltration treatment is negative (-0.655), indicating that patients in the Wound Infiltration group tend to have higher pain scores than patients in the ESP-block group.

Conclusion

ESP-block used in a multimodal opioid-sparing analgesia regimen provides reduction in opioid consumption with a low incidence of side effects.

Informed consent was obtained.

**References**

1. Bajwa SJ et al. J Craniovertebral Junction and Spine. 6:105–10; 2015
2. Forero M et al. Regional Anesthesia Pain Med 41:621–627; 2016.

**Fig. 1:**
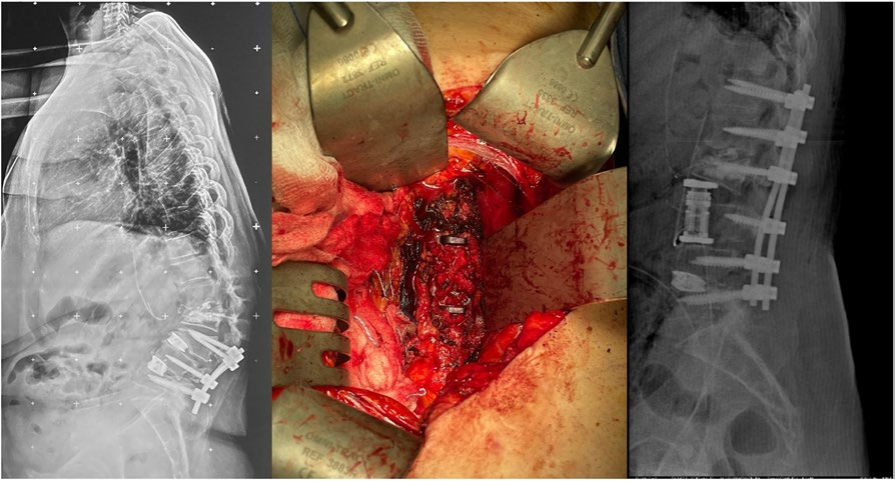
** (abstract A73).** Undergoing instrumented spinal surgery

### A74 Locoregional anesthesia for removing subclavicular lesion in patient with dermatomyositis

#### G. Torregiani^2^, R.L. Gentile^1^, S. Orlando^1^, G. Gazzè^1^, P. Papa^1^, V. Ceccarelli^1^, L. Coria^1^, F. Pelle^3^, C. Bonarrigo^2^, M. Alonzi^2^, G. Fedele^2^, M. Covotta^2^, E. Forastiere.^2^

##### ^1^Department of Anesthesiology, Critical Care and Pain Medicine, Sapienza University of Roma, Rome, Italy, ROMA, Italy; ^2^Unit of Anesthesiology and Intensive Care, IRCCS Regina Elena National Cancer Institute, Rome, Italy, Roma, italy; ^3^Department of Surgery, IRCCS Regina Elena National Cancer Institute, Rome, Italy, Roma, Italy.

###### **Correspondence:** R.L. Gentile

*Journal of Anesthesia, Analgesia and Critical Care 2024*, **4(1):**A74

**Background**

Distal girdle muscular dystrophy (LGMD) is a genetic disease characterized by weakness and atrophy of the muscles of the limbs, patients may present respiratory muscle weakness. The most common presenting symptoms are weakness of the hips and proximal leg muscles. The association with dermatomyositis which is an autoimmune inflammatory disease that affects the skin and muscles can get worse the picture of muscle weakness. Informed consent to publish had been obtained.

**Case report**

A 50-year-old female patient with LGMD onset at the age of 20 and complicated by recently diagnosed dermatomyositis.

ln history (2011) the patient was diagnosed with melanoma. In April 2023, a subclavian lymp node with diagnosis of melanoma recurrence was found. The neurologist emphasized that the patient’s neurological worsening was secondary to her oncological problem and refractory to medical therapy. Therefore, surgery to remove the subclavicular lesion was deemed the only therapeutic alternative for resolving dermatomyositis.

On the day of surgery, the patient underwent the surgical procedure with ultrasound-guided regional blocks: clavipectoral band block using 10 ml of ropivacaine 0.5% followed by pectoral nerve block I (PECS I) using 15 ml of ropivacaine 0.5%. Sedation was accomplished by administering midazolam 2 mg, ketamine 60 mg in refracted boluses and dexmedetomidine 1 mcg/kg over 10 min and then in continuous infusion at 0.7 mcg/kg/h. Twenty minutes after block and following local infiltration with lidocaine 2% 5 ml of the incision site, the subclavicular lymp node package was removed without complications. Pain control was achieved with paracetamol 1 g and ketorolac 30 mg. Vital parameters were normal and Numerical Rating Scale was 0, so she was transferred to the ward.

**Conclusion**

The use of locoregional anesthesia techniques is known in other types of neuromuscular disorders (1). In this rare condition of dystrophy, regional block associated with sedation allowed the performance of a safely and successful surgery avoiding risk of using neuromuscular blocking agents in a patient with muscle weakness and extremely high value of creatine phosphokinase (4441 mU/ml).

Informed consent was obtained.

**Reference**

1. Budget M, Eren I, Kucukay S. Regional anaesthesia in a Duchenne muscular dystrophy patient for upper extremity amputation. Agri. 2014;26(4):191-5

### A75 Use of the Dual Subsartorial Block (DSB) for the control of postoperative pain in patients undergoing knee arthroprothesis—case update

#### A. Fruncillo^1^, L. Baccari^1^, F. Barra^1^, D. Giordano^1^, P. Russo^1^, C. Chiumiento^2^, F. Chiumiento^1^

##### ^1^Asl Salerno- UOC Anestesia e Rianimazione Ospedale Maria SS. Addolorata, Eboli, Italy; ^2^Università degli Studi di Salerno—Scuola Spec.ne Anestesia e Rianimazione, Salerno, Italy

###### **Correspondence:** A. Fruncillo

*Journal of Anesthesia, Analgesia and Critical Care 2024*, **4(1):**A75

**Background**

The saphenous nerve, a branch of the femoral nerve, provides sensitivity to the inside of the leg, foot, and knee. To relieve pain after knee surgery, several nerve block techniques are used. In our center we have been using the dual subsartorial block since 2021 with excellent results, which has proven to be particularly effective and safe.

This technique combines two blocks: one in Scarpa's triangle and one in the adductor canal. Ultrasound guidance facilitates the precision and success of the operation, reducing post-operative pain and promoting faster recovery.

Traditional blocks of the femoral, obturator, and sciatic nerves, responsible for innervation of the anterior, posteromedial, and posterior regions of the knee, respectively, provide complete analgesia in their respective territories but generally cause unwanted motor blocks.

The aim of the study was to evaluate, after the first experience in the 2021–2022 study, the effectiveness of the double subsartorial block for post-knee arthroplasty analgesia by associating a postoperative intravenous analgesic therapy.

**Materials and methods**

From May 2022 to April 2024 an additional 34 patients undergoing knee arthroplasty who received a double subsartorial block were enrolled. The block was performed with the patient in the supine position and the thigh rotated laterally using Ropivacaine 0.25% 20 ml and Dexamethasone 4 mg in two injections: The first injection is performed using Ropivacaine 0.25% 20 ml and Dexamethasone 4 mg at level of the distal femoral triangle and directly targets the saphenous and vastus medialis nerves. In the second injection, ropivacaine 0.25% 20 ml + dexamethasone 4 mg were injected perivascularly under the vastus adductor membrane. Injected at this level, the mixture will travel along the femoral vessels to block the popliteal plexus and then the posterior divisions of the obturator, tibial, common peroneal and sciatic nerves (posterior region of the knee). Patients were assessed for postoperative pain at regular intervals (3–6-12–18-24 h).

**Results**

Mean duration of analgesia was 20 +—4 h. During the first 24 h paracetamol 1 g + ketorolac 30 mg iv × 3 were administered to all patients. No s.e. observed. In 3 cases it was necessary to administer morphine IV due to lack of pain relief, probably due to a block not correctly performed.

In 9 cases, motor block occurred such that active mobilization was not possible on the same day of surgery. All other patients were mobilized the same day.

Conclusions: DSB is an effective and safe technique for post-knee arthroplasty analgesia. It can provide prolonged pain relief without causing significant motor blockages and can promote faster rehabilitation. The use of post-operative intravenous pain therapy has made possible to drastically reduce the use of opioids in the post-operative period and has contributed to better patient compliance with early mobilization.

**References**

1. Sonawane K, Dixit H, Mistry T, Balavenkatasubramanian J (2021) Anatomical and Technical Considerations of “Dual Subsartorial Block” (DSB), A Novel Motor-sparing Regional Analgesia Technique for Total Knee Arthroplasty. Open J Orthop Rheumatol 6(1): 046–056.
2. '(DSB)': an innovative procedure-specific, motor-sparing and opioid-sparing regional analgesia technique for total knee replacement surgery—a pilot study. Clin Anesth. 2021;69:110149. [PubMed]

**Fig. 1:**
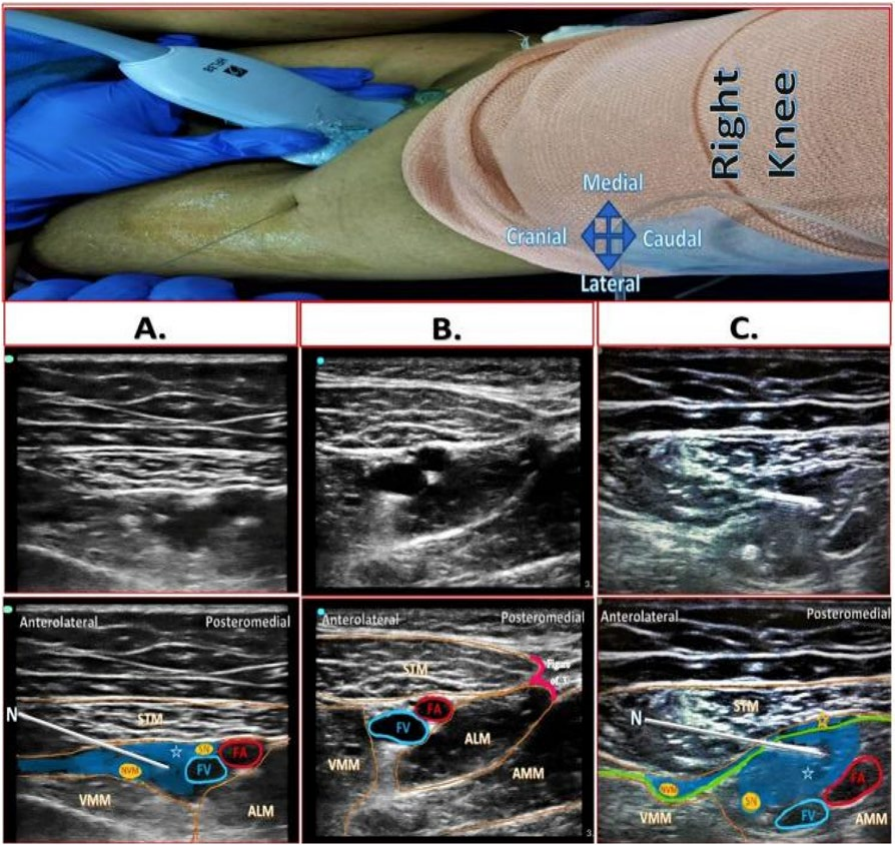
** (abstract A75).** See text for description

## Obstetrical and perinatal

### A76 Anesthetic management of elective caesarean section in a patient affected by catecholaminergic polymorphic ventricular tachycardia

#### G. Salve^1^, T. Fortuna^1^, F. Cantagalli^1^, F. Angelucci^2^, M. Di Carlo^2^, F. Marinangeli^2,3^, F. Venturoni^1^

##### ^1^Ospedale G. Mazzini, Teramo, Italy; ^2^Università degli Studi dell'Aquila, Scuola di specializzazione Anestesia e Rianimazione, L'Aquila, Italy; ^3^Ospedale San Salvatore, L'Aquila, Italy

###### **Correspondence:** G. Salve

*Journal of Anesthesia, Analgesia and Critical Care 2024*, **4(1):**A76

**Background**

Catecholaminergic Polymorphic Ventricular Tachycardia (CPVT) is a rare malignant hereditary arrhythmogenic syndrome, characterized by potentially lethal exercise or emotion-induced adrenergic-dependent tachyarrhythmias, in the absence of structural heat disease. A family history of syncope or cardiac arrest and sudden death is normally present [2]; RYR2, CALM1, CALM2, CALM3, KCNJ2-related CPVT are inherited in an autosomal dominant manner. CASQ2-related CPVT is typically inherited in an autosomal recessive manner. Stress test is needed for diagnosis. Therapy consists in avoiding stress and intense physical activity, beta-blockers (Nadolol), flecainide and implantation of Loop recorder, Pacemaker, or Implantable Cardioverter Defibrillator (ICD). Anesthetic management require scrupulous evaluation and management [1,2,3]; few cases are reported in literature [4].

**Case report**

We present the case of a 25-year-old pregnant woman (39 weeks) with CPVT who underwent an elective Cesarean Section (CS). CVPT was diagnosis at the age of 20, a genetic counseling evidenced a RYR2 mutation, a loop recorder was implanted and a therapy with Nadolol was started. During pregnancy she continued beta-blocker therapy, and, according to the cardiologist, an elective CS was scheduled to avoid labour emotional and physical stress. Before the CS an anesthesiologic consult was done, a Subarachnoid Anesthesia (SA) was scheduled and was suggest skipping the Nadolol dose close before the CS to reduce the risk of hypotension after SA. In the operating room, the patient was equipped with two large bore peripheral venous catheters, defibrillator, ECG, Peripheral oxygen Saturation (SpO2), Non-Invasive Blood pressure (NIBP) monitoring, the anesthesia machine, and drugs for general anesthesia and emergency were readily available. The obstetric and the pediatric consultant were in the operating room for the newborn. After a fluid loading a subarachnoid spinal anesthesia in the L3-L4 space with Hyperbaric Bupivacaine 0.5% (12 mg) was performed. Hypotension occurred and the patient needed crystalloids and phenylephrine. No maternal tachyarrhythmias emerged. The newborn presented good tone, good breathing efforts and good heart rate. Postoperative pain was managed with acetaminophen, ketorolac, and rescue morphine. The patient was discharged from operating room in obstetric ward with ECG, NIBP and SpO2 continuous monitoring; two days after CS the patient was discharged from hospital.

**Conclusions**

We successfully manage a CS of a pregnant woman with CPVT with SA, no tachyarrhythmias emerged during or after the procedure.

Informed consent was obtained.

**References**

1. Campbell G, Arfanis K, Smith AF. Distraction and interruption in anaesthetic practice. Br J Anaesth. 2012 Nov;109(5):707–15. 10.1093/bja/aes219. Epub 2012 Jul 1. PMID: 22750727.
2. Leenhardt A, Lucet V, Denjoy I, Grau F, Ngoc D, Coumel P. Cate- cholaminergic polymorphic ventricular tachycardia in children. Circulation 1995; 91: 1512–9.
3. Napolitano C, Mazzanti A, Bloise R, Priori SG. Catecholaminergic Polymorphic Ventricular Tachycardia. 2004 Oct 14 [updated 2022 Jun 23]. In: Adam MP, Feldman J, Mirzaa GM, Pagon RA, Wallace SE, Bean LJH, Gripp KW, Amemiya A, editors. GeneReviews® [Internet]. Seattle (WA): University of Washington, Seattle; 1993–2024. PMID: 20301466.
4. Chan T, Dob D. The anaesthetic management of a parturient with polymorphic catecholamine-sensitive ventricular tachycardia. Int J Obstet Anesth 2002; 11: 122 – 4.

### A77 Case report: an application of the ultrasound—guided pudendal nerve block in the obstetrics setting

#### G. Ranieri, P. Ciocchetti, F. Rucci, C. Todde, C. De Bartolomeo, L. Pellas, A. Zagari, M. Aversano, M.C. Ferrante, F. Prencipe, G. Nardo, F. Tamburi, A. Coviello, A.U. De Siena, M.G. Frigo

##### Ospedale Isola Tiberina, Gemelli Isola, Roma, Italy

###### **Correspondence:** G. Ranieri

*Journal of Anesthesia, Analgesia and Critical Care 2024*, **4(1):**A77

**Background:**

The pudendal nerve (PN) is a sensory-motor nerve: its sensory distribution covers the lower two thirds of the labia majora and the perineum [Fig. 1] (1,2). After its origin from the sacral branches S2-S3-S4, PN exits from pelvis, through the greater sciatic foramen below the piriform muscle, with the homonymous vein and artery; it turns around the ischial spine above the sacrospinous ligament and enters again in pelvis through the lesser sciatic foramen. At the end, it enters in the ischio-anal fossa (Alcock canal) where it gives its terminal branches: inferior rectal nerve, perineal nerve and the dorsal nerve of clitoris or penis (3,4). Initially performed with an anatomical landmark based approach on the palpation of the ischiatic spine, recently the ultrasound (US) guided technique was proposed with three different approaches: the posterior one, at Alcock canal, and the transperineal approach(3–5).The main indications are urology, gynecology, obstetrical procedures, and pudendal neuralgia treatment (1,6,7).

**Case Report:**

In our case, we are discussing about the application of the PN block in obstetrical settings. A multipara 32-years-old female at 39 weeks of gestational age, with language barrier, accessed to our center with a spontaneous labor. The initial Bishop score was 8 (Dilatation 4 cm, effacement 80%, station -2, medium consistency and mid position) with a cephalic presentation; according to our protocol, we performed a combined spino-epidural technique at level L2-3, with atraumatic Withacre 25G needle and Thuoy 16G plus a 18G catheter, to administer intrathecal long-lasting local anesthetic and opioids (ropivacaine mg 0.2% 1 ml + sufentanyl 2.5 mcg in saline for a total volume of 5 ml). One hour later, the woman presented spontaneous rupture of the amniochorionic membranes and the Bishop become 12 (complete dilatation, fetal head at -1/0) and a top-up epidural dose became necessary to control the pain but we noticed epidural catheter was obstructed. Due to the poor women’s cooperation, the replacement of the catheter was not possible. After few minutes, she had a vaginal delivery with a 4th perineal laceration. After the spontaneous placental delivery and the acquiring of written informed consent, we performed a bilateral US PN block with transperineal out of plane approach [Fig. 2–3]. In lithotomy position, a mixture of ropivacaine 0,5% 4 mL and mepivacaine 2% 4 mL for each side was injected. After 10 min, the pinprick test resulted negative in the perineal area; the laceration repair was performed in the labor box by the gynecologist, under standard vital parameters monitoring. The patient felt no pain during the procedure, no sedation was needed, and she was satisfied of early skin-to-skin contact with her newborn.

**Conclusions:**

The PN block is a safe and effective anesthetic technique for perineal repair. In this case report, we have reported our experience about the usefulness of this technique in the absence of adequate epidural analgesia during perineal laceration repair. The use of US, allowing pudendal artery pulsation localization, makes the technique safer and quicker in expert hands, reducing accidental artery puncture.

Informed consent was obtained.

**Fig. 1:**
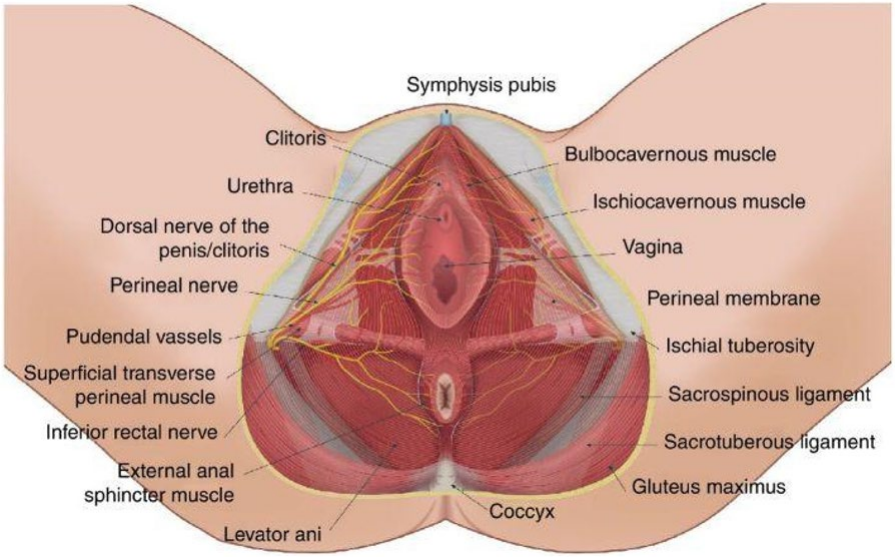
** (abstract A77).** Anatomy of pudendal and its terminal branches

**Fig. 2:**
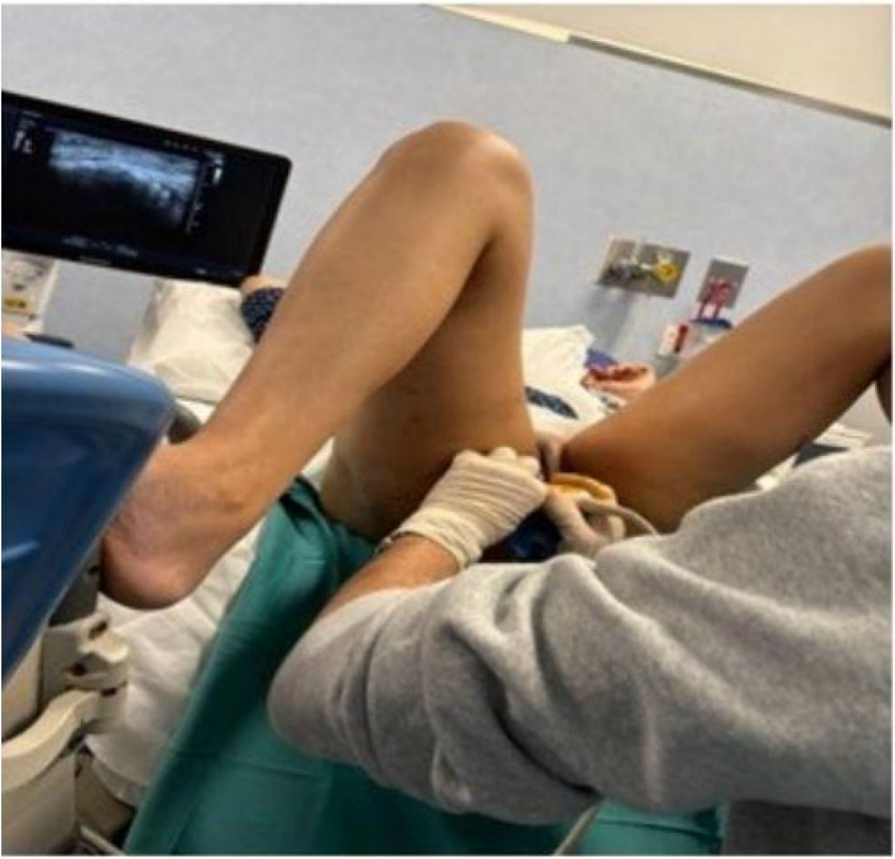
** (abstract A77).** Lithotomy position for Ultrasound Transperineal Pudendal Nerve Block Approach

**Fig. 3:**
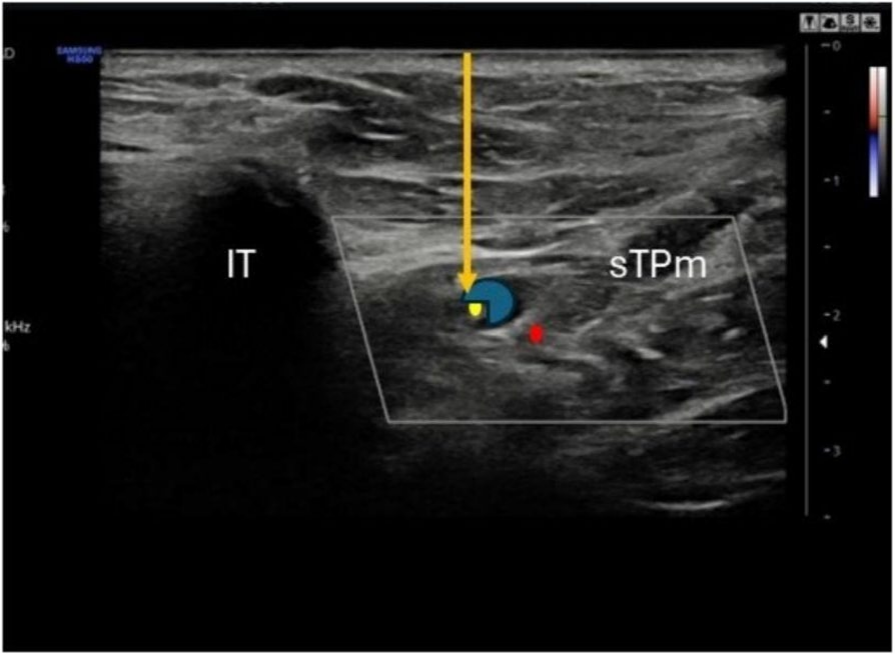
** (abstract A77).** Ultrasound image of the pudendal nerve block. IT = ischial tuberosity, sTPm = superficial transperineal muscles, yellow point = pudendal nerve, red point = pudendal artery, orange arrow = needle path, blue = local anesthetic distribution

### A78 Morbidity and mortality after major surgery in preterm children: the *regina* margherita hospital experience

#### S. Pourshayesteh, M. Cavagnino, A. Conio, S. Quaglia.

##### Dipartimento di Anestesia e Rianimazione, Città della Salute e della Scienza, Torino, Italy

###### **Correspondence:** S. Pourshayesteh

*Journal of Anesthesia, Analgesia and Critical Care 2024*, **4(1):**A78

This paper aims to analyze the outcomes of preterm children with low birth weight, born with congenital or acquired pathologies for which surgery was necessary within one month of life.

Preterm deliveries are those that occur at less than 37 weeks gestational age after spontaneous labour with intact membranes, preterm premature rupture of the membranes and labour induction or caesarean delivery for maternal or fetal indications.

Approximately 15 million babies are born preterm annually worldwide, indicating a global preterm birth rate of about 11%1.

In our experience at Regina Margherita Hospital, reference center of the Piedmont and Valle d'Aosta region in Italy for pediatric disease, from January 2021 to April 2024, 26 preterm children (GA 30 weeks ± 4) with surgical needs were born weighing less than 2000 g (1403 g +—455 g). Of these, nine children underwent thoracic surgery to correct esophageal atresia, twelve were treated for abdominal pathologies such as necrotizing enterocolitis, volvulus or anomalies of the cloaca or anus, duodenal atresia or gastroschisis, five had an Ommaya reservoir implanted for cerebral hemorrhages causing hydrocephalus.

In some cases, the surgical pathology was part of a broader picture of syndromic anomalies such as Trisomy 21 (7,7% of patients) and Tetralogy of Fallot (3,8%), associated with pathological features linked to prematurity such as RDS (23%), jaundice (15%), retinopathies (15%), patent Botallus ductus arteriosus (23%).

After surgery, all children were admitted to the Pediatric Intensive Care Unit (PICU), sedated with Midazolam (0.05—0.3 mg/kg/h) and/or Fentanyl (1–2 mcg/kg/min). In two cases of correction of esophageal atresia, the placement of an ESP catheter was used for analgesia.

Protective ventilation was guaranteed (4–6 ml/kg) with minimal PEEP (4–8 cmH2O) and minimal FiO2 to obtain SpO2 > 90–94% and adequate respiratory exchanges, tolerating mild hypercapnia without significant impact on pH.

Seven patients required amine support with dopamine or adrenaline which was rapidly weaned in less than 72 h.

The average length of stay in the PICU was 7 +—10 days. During this time, some complications were encountered: one child developed Ventilator-Associated Pneumonia due to Methicillin – Resistant Staphylococcus Aureus, two extubation failures due to chylothorax with the need for reintubation, in one case an emergency pericardiocentesis was necessary due to cardiac tamponade, in three cases cerebral hemorrhage was found and in one case was necessary to implant an Ommaya reservoir.

Sadly, in two cases the patients died (one due to devastating cerebral hemorrhage and the other due to cardiovascular collapse).

After their stay in the PICU, the children were transferred to the Neonatal Intensive Care Unit (NICU, a unit managed by pediatricians) where they completed the post-operative and perinatal period. The average NICU stay was 63 +—36 days.

In conclusion, the increased survival of premature children with low birth weight represents a resuscitation and anesthetic challenge for surgical patients, the objective is the lowest onset of complications to guarantee minor mortality and better quality of life.

Informed consent was obtained.

**Reference**

1. Walami S R, Global burden of preterm birth. Int J Gynaecol Obstet, 2020 July; 150(1):31-33

### A79 Impact of timing of neuraxial analgesia on obstetric, anesthesiologic, and neonatal outcomes in induced labour

#### I. Bitetti, S. Poma, C. Baldi, F. Broglia, M. Ciceri, M. Fuardo, F. Morgante, S. Pellicori, E.M. Roldi, M.P. Delmonte, A. Locatelli

##### IRCCS Fondazione Policlinico San Matteo, Pavia, Italy

###### **Correspondence:** S. Poma

*Journal of Anesthesia, Analgesia and Critical Care 2024*, **4(1):**A79

**Background:** Safety of early administration of neuraxial analgesia has been widely investigated in spontaneous labour (1). Less evidence exists for its use in induced labour. Induction has become a common procedure, associated with a slower progression of labour, greater and earlier pain, which results in an increased and earlier demand for analgesia. (2) The objective of this study is to assess the impact of early labour analgesia on obstetric and neonatal outcomes in induced labour.

**Materials and methods:** A one-year retrospective observational study was conducted with approval of ethics committee. After gaining patients’ consent, primiparous women with a full-term pregnancy whose labour was induced with Cook balloon or prostaglandins were enrolled if they requested neuraxial analgesia. Patients were divided in two groups according to repertorised cervical dilatation at the start of analgesia: Group A < 4 cm (latent stage according to the guidelines of the National Institute for Health and Care Excellence) and Group B between 4 and 6 cm. Outcomes in the early and late group were compared. The following variables were collected: duration of labour, incidence of caesarean section, use of oxytocin, onset of abnormal fetal heart rate within 30 min after a top-up, incidence of episiotomy, extent of blood loss, need for repositioning of the epidural catheter due to inadequate analgesia, onset of breakthrough pain, defined as the need for 'rescue' top-ups within 60 min from the previous one, Apgar score (at 1 and 5 min), neonatal umbilical pH and neonatal intensive care unit admission. 107 patients were included in the study: 50 in Group A, 57 in Group B. The groups were homogeneous in terms of demographic, anthropometric and obstetric characteristics. The results are summarised in Table 1.

**Conclusions**: Early neuraxial analgesia in induced labour does not appear to affect obstetric outcomes, duration of labour and type of delivery. No significant difference in the adequacy of pain control was observed. Neonatal outcomes were similar regardless the timing of analgesia. The only significant difference detected was a reduced incidence of abnormal fetal heart rate in patients who received early analgesia, which suggests a possible protective role of this practice. If confirmed, this result advocates, beyond the safety, a possible benefit of administering neuraxial analgesia during the latent phase of labour.

**References**

1. Sng BL, Leong WL, Zeng Y, Siddiqui FJ, Assam PN, Lim Y, Chan ES, Sia AT. Early versus late initiation of epidural analgesia for labour. Cochrane Database Syst Rev. 2014; CD007238. https://doi.wiley.com/10.1002/14651858.CD007238.pub27.
2. Vahratian A, Zhang J, Troendle JF, Sciscione AC, Hoffman MK. Labor progression and risk of cesarean delivery in electively induced nulliparas. Obstet Gynecol. 2005;105:698–704.

**Table 1:** **(abstract A79).** Comparison between the 2 study groups for outcomes investigated

	Group A	Group B	*p*-value
Duration first stage	165 (120 – 278)	165 (90—285)	0,6632
(minutes; median [25p-75p])			
Duration second stage	45 (30—65)	55 (27—88)	0,1736
(minutes; median [25p-75p])			
Caesarean Section^a^	12 (24.0%)	8 (14.0%)	0,1132
Maximum oxytocin rate	1.14 (± 0.6)	0.91 (± 0.46)	0.066
(IU/h; mean [± ds])			
Fetal heart rate abnormalities^a^	2 (4.0%)	10 (17.9%)	**0.025**
Episiotomy^a^	21 (56.8%)	25 (52.1%)	0,4639
Blood loss > 500ml^a^	23 (46%)	25 (43.9%)	0,2729
Peridural catether repositioning^a^	3 (6.0%)	0 (0.0%)	0.061
Rescue epidural top-ups^a^	3(6.0%)	1(1.8%)	0,1722
Apgar score at 1 minute^b^	9 (9—9)	9 (8—9)	0.069
Apgar score at 5 minutes^b^	10 (9—10)	10 (9—10)	0,6097
Neonatal umbelical pH^b^	7.28 (7.23—7.33)	7.27 (7.25—7.33)	0,2722
Neonatal Intensive Care Room^a^	5(10.0%)	4 (7.1%)	0,4153

^a^number/percentage

^b^numbers; median [25p-75p]

### A80 Postpartum haemorrhage: time for personalised therapy?

#### F. Barbati^1^, S. Costantini^2^, E. Corno^1^, D.M. Locane^1^, M. Maio^1^

##### ^1^AOU Città della Salute e della Scienza, Torino, Italy; ^2^ Università degli studi- UNITO, Torino, Italy

###### **Correspondence:** M. Maio

*Journal of Anesthesia, Analgesia and Critical Care 2024*, **4(1):**A80

Postpartum haemorrhage (PPH) is the leading cause of maternal death and constitutes an obstetrical emergency. Viscoelastic point-of-care tests (POCT) of coagulation offer real-time global assessment of haemostasis and are increasingly playing a significant role in managing bleeding across various clinical settings. Here we present the case of a 36 year-old multiparous (G4P1 + 2) pregnant woman admitted to our maternity hospital at 34 + 5 weeks of pregnancy (wop) due to premature rupture of membranes. Obstetric ultrasound indicated an apparently normal placenta, anterolateral to the body of the uterus. After developing severe pelvic pain around the site of the previous caesarean section, considering the gradual increase in uterine contractile activity and breech presentation, she underwent a caesarean section at 35 + 1 wop. The surgical procedure was complicated by failure of the placenta to deliver, with clinical evidence of probable placenta accreta spectrum disorders (PASD), resulting in rapid blood loss (1600 ml) with maternal haemodynamic instability. Haemodynamics was supported by rapid volemic filling with crystalloids (1500 ml) and phenylephrine infusion (maximum 0.10 mcg/kg/min). The type and timing of laboratory monitoring and the administration of blood derivatives were based on the Italian multidisciplinary protocol for managing PPH with coagulation POCT systems. This led to the subsequent transfusion of only 3 units of red blood cells before achieving haemodynamic stability; no other blood components or derivatives were necessary, as serial viscoelastic POCT (Fig. 1) and conventional laboratory tests consistently showed values in the normal range (Fig. 2). At the same time, surgical control of bleeding was obtained through a caesarean hysterectomy, leaving the placenta in situ. PASD was confirmed by anatomopathological examination.

Our case confirmed that the use of coagulation POCT systems can guide transfusion therapy by tailoring it to individual needs, promptly detecting coagulation disorders and correcting them only when necessary.

This saves costs, avoids the administration of unnecessary amounts of coagulation products and improves the outcome of PPH in the context of PASD.

Although randomized controlled trials highlighting the long-term benefits of this approach and robust costeffectiveness analyses are lacking, the existing scientific literature and clinical experience, such as the case presented here, lead us to suggest that colleagues, whenever feasible, adopt coagulation POCT-guided transfusion therapy for the treatment of postpartum haemorrhage. Otherwise, there is a risk of developing two tiers of maternity services, where smaller hospital units with fewer resources may not justify the costs of PPH treatment algorithms.

Informed consent for the publication was obtained.

**Fig. 1:**
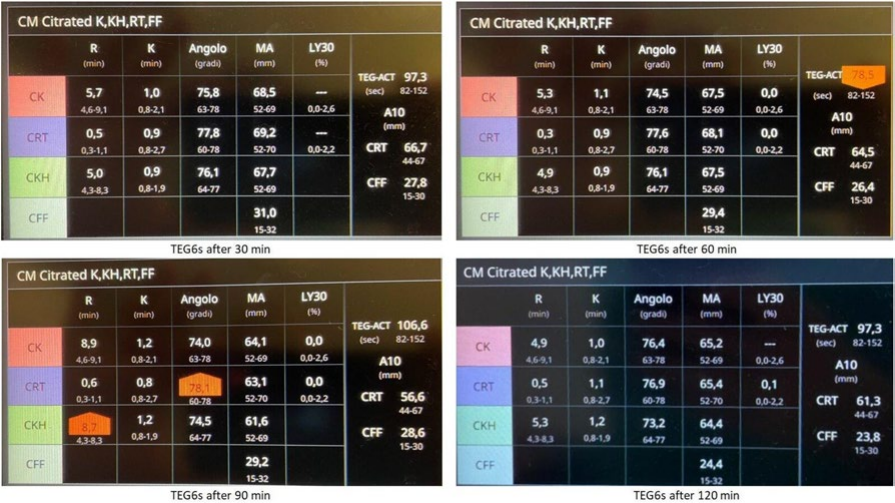
** (abstract A80).** TEG6s after 30, 60, 90 and 120 minutes

**Fig. 2:**
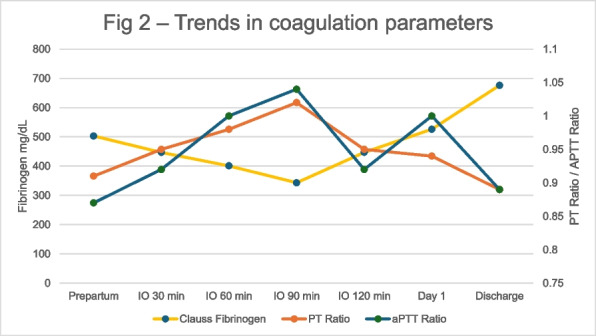
** (abstract A80).** Trends in coagulation parameters

### A81 The fontan pregnancy: a case series

#### E. Menaldo^1^, A. Zito^1^, S. Baino^1^, F. Petey^2^, G. Maglione^1^, M. Maio^1^

##### ^1^AOU Città della Salute e della Scienza, Torino, Italy; ^2^Università degli studi- UNITO, Torino, Italy

###### **Correspondence:** E. Menaldo

*Journal of Anesthesia, Analgesia and Critical Care 2024*, **4(1):**A81

**Introduction:**

With the introduction of the Fontan procedure in 1971, patients with a congenital single ventricle greatly improved survival. As a consequence, many more women in relatively good conditions may aspire to maternity. However, Fontan maternal complications include adverse events such as arrythmias, thromboembolic events, heart failure and obstetric hemorrage. Given the lack of population, maternal mortality is low. The Fontan pregnancy is complicated by miscarriage, perinatal morbidity and mortality, due to prematurity from placental insufficiency.

**Material and methods:**

Three Fontan pregnant women came to our multidisciplinary clinic. Written consent was acquired for the use of patients data. Patients were monitored every 21 days with a gynecological, cardiological and anesthesiological periodical control. NT-pro-BNP, echocardiogram, clinical examination were performed at each visit. By now, two women had a scheduled anticipated vaginal delivery. Demographic and cardiological data were recorded in Table 1.

No cardiac adverse events were recorded during pregnancy. Induction of delivery was performed in both cases with the use of CRB, amniorhexis and oxytocin. Epidural analgesia was performed at the beginning of labor. We used repeated doses of ropivacaine 0,1% up to 10 ml and fentanyl 50 mcg. Hemodynamical monitoring was performed through radial access. Small fluid boluses were given to maintain preload and CO. No need of vasopressors was recorded. The mean time of labor was 55 min. Valsalva maneuvres were minimized, and only one patient needed an instrumental delivery. Endocarditis prophylaxis was administered. One patient had a post-partum hemorrage, which required blood transfusion and low dose phenylephrine.

Both babies had good Apgar scores 9/9, with a mean body weight of 1870 g.

**Discussion:**

There is an extremely wide complexity and variability of the post-Fontan long-term outcomes. Both our patients were in a relatively good condition at term, with a preserved function of the systemic ventricle. Vaginal delivery is preferable in Fontan parturients in good condition, as it is associated with less blood loss, less risk of infection and thrombo-embolic events. Epidural analgesia is mandatory, also for the necessity of instrumental delivery; a careful titration of sympathectomy, peripheral vasodilation and reduced venous return is perfectly achieved with small doses of epidural anesthetics. As anticipated by literature, obstetric hemorrage was expected (50%), due to use of anti-platelet and anti-coagulant drugs and to the uterine atony following the underdosage of uterotonic drugs for fear of cardiovascular complications. Endocarditis prophylaxis is recommended in Fontan pregnancies, for the prosthetic materials used in the procedures.

Low neonatal weight was probably due to a combination of early induction of delivery and maternal use of beta-blockers.

**Table 1:** **(abstract A81).** Demographic and cardiological data

Age (mean ± SD)	31 ± 1	Basal NT-pro-BNP (mean ± SD)	23,1 ± 1,4
PARA	0000	NT-pro-BNP at delivery (mean ± SD)	57 ± 22,6
Pre-gravidic BMI (mean ± SD)	25,5 ± 5,2	NYHA at delivery (mean ± SD)	2
BMI at delivery (mean ± SD)	27,9 ± 3,5	Mean GW (mean ± SD)	36 + 3

### A82 Pregnancy in patients with cardiac disease: results from the cardio-obstetric model of care

#### E. Menaldo^1^, S. Ditaranto^1^, E. Balzani^2^, A. Giaccone^1^, M. Verra^2^, M. Maio^1^

##### ^1^AOU Città della Salute e della Scienza, Torino, Italy; ^2^Università degli studi -UNITO, Torino, Italy

###### **Correspondence:** E. Menaldo

*Journal of Anesthesia, Analgesia and Critical Care 2024*, **4(1):**A82

In the last few decades, an increasing number of women with cardiac diseases reached the childbearing age, as a results of improvements in diagnostic and terapeutic options. Pregnancy may exacerbate or develop maternal cardiac problems, causing a significant increase in maternal morbidity and mortality. As recently advocated, pregnancy in women with cardiac diseases must be periodically controlled by a Cardio-Obstetric Team. In our institution we developed a multidisciplinary team composed by Gynecologists, Cardiologists, Internal Doctors and Anesthesiologists in order to monitor pregnancies from the pre-conceptional counselling to the post-partum period, to detect and treat any maternal decompensation, to plan the ideal time and mode of delivery, the best anesthetic technique and the indications and dosage of anti-coagulants and cardiovascular treatments.

With the acquisition of written consent, we retrospectively analysed data from 445 pregnancies from January 2007 to December 2023. Demographic data (Table 1), Delivery mode (Table 2), Maternal complications (Table 3) and Neonatal outcome (Table 4) were analysed. We considered 2014, the year of institutionalization of the multidisciplinary team, as a cut-off in delivery mode and anaesthetic management.

**Discussion**

As for demographic data, no significant differences between years was noted. A visible increase, yet not significant, in vaginal delivery is clear, in accordance to the 2011 and 2018 ESC guidelines. Labor analgesia, especially epidural, has well established. General anaesthesia has nowadays only very few indications. Admission to ICU has sensibly reduced, due to the diminished number of CS; no differences between years in ICU admission due to cardiac complication was recorded. Neonatal data show overall good Apgar scores, with few cases of low neonatal weight, mainly due to extreme prematurity.

The institution of a multidisciplinary Cardio-Obstetric team has greatly improved the management of pregnant patients with heart diseases. The accordance to specific guidelines resulted in an increase of planned vaginal deliveries under epidural analgesia with a reduction in maternal ICU admission.

**Table 1:** **(abstract A82).** Demographic data

Number of pregnancies	445
AGE (mean ± SD)	32,4 ± 5,7
Nulliparous/pluriparous	248/197
BMI	%
< 18,5	11,6
18,5–24,9	56,4
25–29,9	24,7
> = 30	7,3
mWHO Class	%
I	21
II	35,2
II-III	13,1
III	25,1
IV	3,2
modified	0,7
na	1,8
ASA stratification	%
II	31
III	60,6
IV	8,4
Congenital/acquired heart disease	267/178
GW at Delivery	%
Pre-term	21,54
< 32 GW	2,51
Term	77,82
Post-term	0,62

**Table 2:** **(abstract A82).** Delivery mode

	2007- 2013	2014–2023
VAGINAL DELIVERY (%)	48%	57%
Analgesia		
Epidural (%)	55	63
Endovenous (%)	3,5	2,6
None (%)	41,5	34,4
CESAREAN SECTION (%)	52	43
Anaesthesia		
General	26	16
Epidural	26	25
Spinal	48	59

**Table 3:** **(abstract A82).** Maternal complications

	2007- 2013	2014–2023
ADMISSION TO ICU (%)	33	20
Cardiac Complications (%)	60	58

**Table 4:** **(abstract A82).** Neonatal outcome

APGAR score 1’ < 7, %	8,31	APGAR score 5’ < 7, %	1,66
Low Weight (< 2500 g), %	24,89	Pre-term (%)	65,6
		Term (%)	34,4
Normal Weight (> 2500 g), %	75,11		

### A83 Pregnancy in a case of emery-dreyfuss muscolar dystrophy

#### M. Maio, E. Menaldo, A. Zito, M. Cedrone, M. Mortara

##### AOU Città della Salute e della Scienza, Torino, Italy

###### **Correspondence:** M. Maio

*Journal of Anesthesia, Analgesia and Critical Care 2024*, **4(1):**A83

**Introduction:**

Emery-Dreyfuss muscolar dystrophy is a genetic pathology characterized by: joint contractures from early childhood, progressive muscolar weakness and cardiac involvement. It has great clinical variability, and cardiac impairment ranges from palpitations to congestive heart failure with variable rythm disturbances; to prevent sudden cardiac death, there is a general consensus in positioning implantable cardioverter-defibrillator (ICD). Respiratory function may be impaired as well. Pregnancy in women affected by E-D Syndrome is quite a challenge. Case reports register peri-partum heart failure, pre-eclampsia and HELLP syndrome complicating pregnancies.

**Case report:**

After the acquisition of written consent from the patient, we describe the management of a 30 ys old primipara affected by EDMD from LMNA gene mutation with a positive family history of hereditary cardiac disease. Her mother, affected, died young for sudden cardiac death.

At first admission to our multidisciplinar clinic she was at 13 + 2 GW. She was mWHO III class; she had wasting of her upper arms, walked with waddling gait and had postcervical muscles contractures that completely impaired neck hyperextension. She was implanted an ICD in 2016 for ventricular arrythmias and had permanent atrial fibrillo/flutter. Her basal NT-pro-BNP was 392 ng/l. For initial hypoventilation, she was on nocturnal NIV. She was on Nadololo 40 mg od, flecainide 50 mg bd and NOAC.

Pregnancy was spontaneous, singular. She rapidly developed lower limb weakness and astenia. She began to move in wheelchair. Cough peak flow was valid (> 400 l/min). For these reasons she had a neurological indication to CS. CPK dosage was normal.

As for cardiac condition, she was asymptomatic for all the pregnancy. At 32 GW, her cardiac function was preserved; the EF was 66%. NT-pro-BNP was 136 ng/l. The ECG showed atypical atrial flutter and monofocal atrial tachycardia.

We performed a 21-days interval between the controls, in order to early detect signs of worsening of the patient clinical conditions and, possibly treat them.

A difficult neuraxial access was due to lumbar hyperlordosis and to the poor mobility of the patient. However, we were aware of the risk of malignant hypertermia and of the expected difficulties in oral intubation.

She was on LMWH 6000 UI bd, ASA 75 mg od, flecainide 75 mg bd, nadololo 40 mg od. We planned CS at 36 + 1 GW. Multiparametric and invasive haemodynamical monitoring was established before spinal anesthesia. We mantained hemodinamical stability with cristalloid infusion and low dose phenylephrine. A baby girl was delivered, weighting 2750 g, with Apgar scores of 9/9. Blood loss was 400 ml. No post-operative complications were registered.

The post-partum visit showed a stable neurological and cardiovascular condition, the patient could reduce flecainide dosage as before pregnancy.

**Discussion:**

EDMD is a rare condition. However, the number of affected women who will undergo pregnancy in future will increase, due to andvances in treatments. Pregnancy may precipitate the clinical signs of the disease. Consequently, patients require lifelong treatment and observation. Only a multidisciplinar approach, with gynecological, cardiological, internal medicine, anesthesiological and neurological periodical control of the patient can successfully determine an uncomplicated pregnancy.

Informed consent was obtained.

### A84 Quadratum lumborum block for post caesarean section pain in a pregnant woman affected by tethered cord syndrome: a case report

#### G. Ranieri^1^, A. Laffi^2^, A. de Siena Uriel^3^, C. Todde^1^, M. Aversano^1^, M.C. Ferrante^1^, C. De Bartolomeo^1^, A. Zagari^1^, F. Prencipe^1^, L. Pellas^1^, F. Rucci^1^, G. Nardo^1^, P. Ciocchetti^1^, M.G. Frigo^1^

##### ^1^UOSD Anestesia e Rianimazione in Ostetricia DEA emergenza Ospedale Isola Tiberina—Gemelli Isola, Roma, Italy; ^2^Dipartimento di Patologia Chirurgica, Medica, Molecolare e area Critica Pisa, Università di Pisa, Pisa, Italy; ^3^Department of Neurosciences, Reproductive and Odontostomatological Sciences, University of Naples, Federico II, Napoli, Italy

###### **Correspondence:** A. Laffi

*Journal of Anesthesia, Analgesia and Critical Care 2024*, **4(1):**A84

**Background**

The literature reports few evidence about spinal dysraphism, spina bifida, tethered cord syndrome, and neuraxial anesthesia (NA)1–5. Quadratus lumborum nerve block III (QLIII) is a method which aims to accomplish both somatic and visceral analgesia for any type of operation that requires intra-abdominal visceral pain coverage from T4 to T12-L1 dermatomes5–8. We present the use of QLIII in management of post cesarean section (CS) pain in a pregnant patient affected by spina byfida and tethered cord syndrome (TCS).

**Case report**

A 39-year-old multipara pregnant woman at 38 weeks of gestational age was evaluated at Isola Tiberina-Gemelli Hospital to perform an elective CS in 2024. She was affected by myelomeningocele, corrected two days after birth, scoliosis, and neurological bladder. The surgery and the illness also caused a TCS. The woman expressed the written consent for anonymous analysis and presentation of her data. Our and previous gynecologists ranked her unable to perform a spontaneous or operative vaginal delivery. She referred us that the previous two CSs were performed under general anesthesia (GA); postoperative pain, nausea, vomit, and movement restriction led her to develop a diagnosed post-trauma distress syndrome. A magnetic resonance imaging staged medullary cone end at level S1-S2, the fusion between the dura mater and the posterior wall of the vertebral canal from L1-L2 to S1-S2, and a ligamentum flavum hypertrophy that reduces the medullary canal area from L2-L3 (Fig. 1). After a multidisciplinary discussion on the NA feasibility, the risk of procedural neurological damage was considered too high for this technique. The anesthesiologist opted for QLIII to manage the postoperative CS pain.

After the end of CS, a curved array transducer was placed in axial plane on the patient’s side just cranial to the iliac crest with the patient under GA in prone position. The sonographic widow obtained was: L4 transverse process, the erector spinae posteriorly, quadratus lumborum (QL) laterally, and psoas anteriorly (Fig. 2). A Stimuplex Ultra 360 20G 100 mm needle was inserted using an in-plane technique from the posterior end of the transducer through the QL muscle. A solution of 20 mL for each side of ropivacaine 0,375%, dexmedetomidine 25 µg, and dexamethasone 4 mg was injected in the fascial plane between the QL and psoas muscles. No complications occurred during the procedure. The patient referred that the verbal analogue pain score was less than 3 in the postoperative first day. She was very satisfied of the early mobilization and the possibility to breastfeed very early her child.

**Conclusion**

This pregnant patient presented a TCS with neurological problem that have made impossible a safe NA and vaginal delivery. We can say that the QLIII represented a safe and effective alternative to NA to manage the postoperative pain following the CS in this patient. The use of QLIII had satisfied the patient, which had a fast recovery and an early contact with her child.

Informed consent was obtained.

**Fig. 1:**
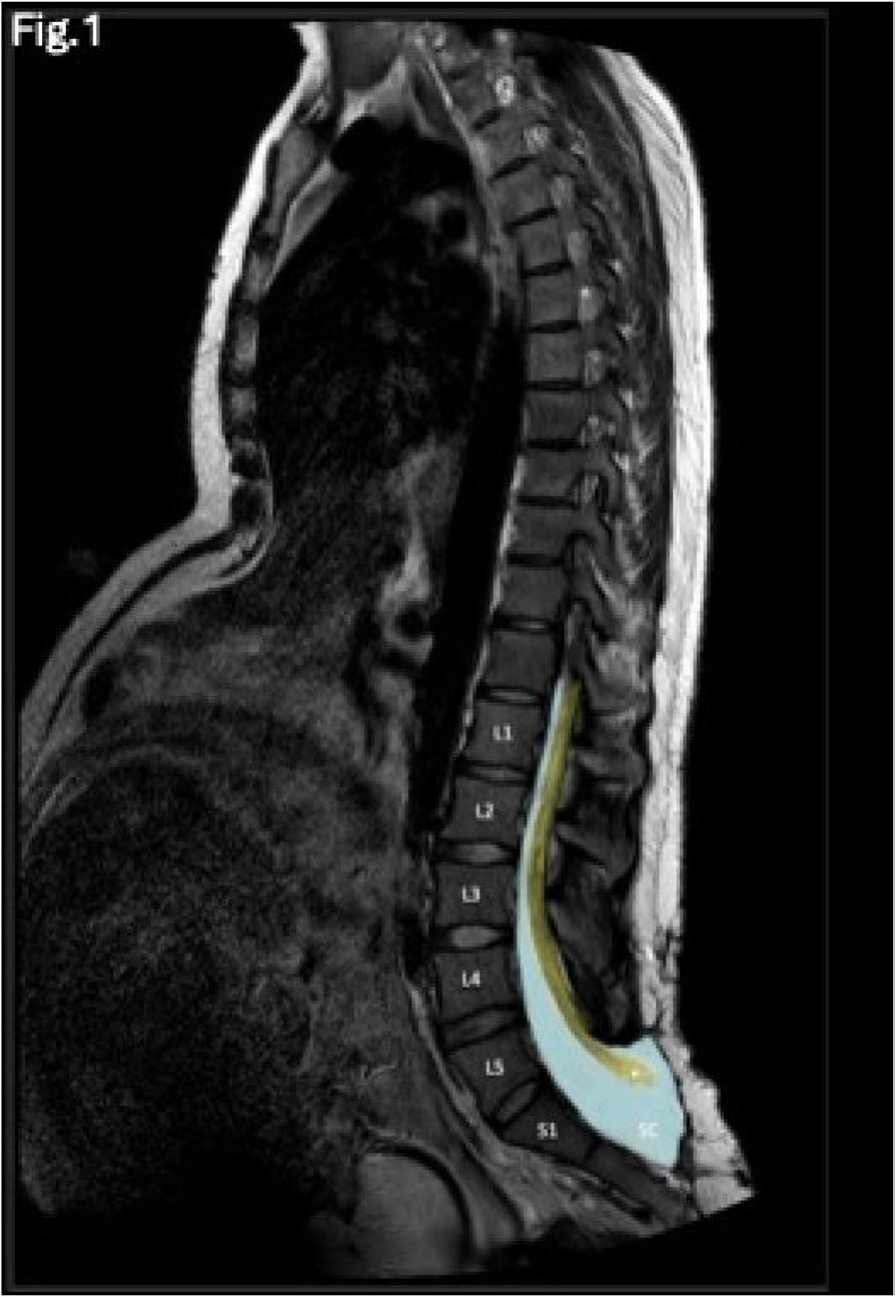
** (abstract A84).** See text for description

**Fig. 2:**
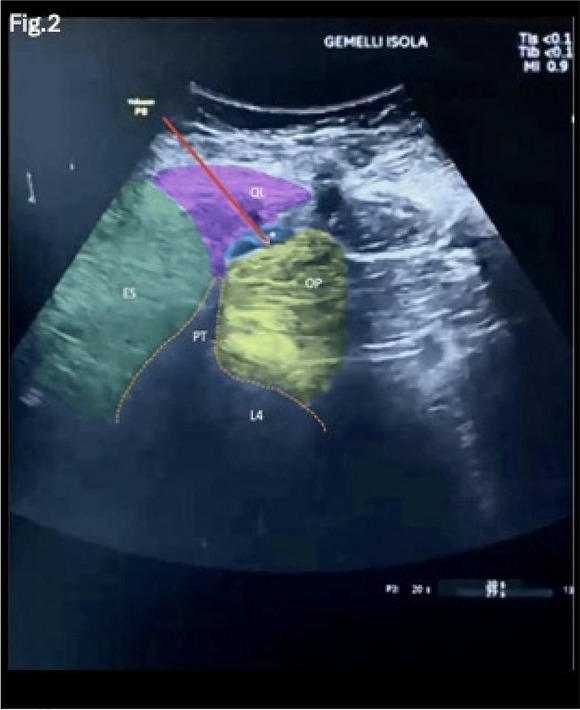
** (abstract A84).** See text for description

### A85 Combined Spinal-Epidural (CSE) analgesia versus epidural analgesia in labor pain management

#### G. Grasso, F. Esposito, V. Di Conza, G. Servillo

##### AOU Federico II, Napoli, Italy

###### **Correspondence:** G. Grasso

*Journal of Anesthesia, Analgesia and Critical Care 2024*, **4(1):**A85

**Background**

Analgesia during labor is commonly used to relieve pain and make the delivery experience more comfortable for the mother. There are several anesthesia options available during labor and delivery. Combined Spinal-Epidural (CSE) Analgesia and Epidural Analgesia are widely used techniques in labor pain management. Understanding their differencies in terms of efficacy, safety, maternal and fetal oucomes is crucial for optimizing obstetric care.

**Methods**

Our study was performed prospectively on 60 pregnant women who requested painless childbirth in a period of six months. All of these patients were ASA I and II. Pregnant women had no controindications for regional anesthesia, had active contractions and with 3–4 cm cervical dilation. They were randomly divided in two groups, CSE group and Epidural group.

In the CSE group we have administered a very low intrathecal dose of opioid without local anesthetic; then we have administreted local anesthetic in epidural space.

In the Epidural group we have admnistered local anesthetic and opioid in epidural space.

**Results**

CSE offers rapid onset of pain relief due to the intrathecal component, complemented by the continuous epidural infusion of local anesthetic. It consists of injecting a small amount of opioid into the intrathecal space, followed by the placement of an epidural catheter. The analgesic effect is faster than with epidural anesthesia with a lower dose of opioid and local anesthetic, it can be used for labour and delivery. It may be associated with less loss of motor sensation than epidural anesthesia, allowing for greater mobility and more maternal satisfaction.

It can be used successfully if you need rapid anesthesia for cesarian section.

Peridural Analgesia is given through a catheter placed in the epidural space. The analgesic effect may have a slower onset. It can be used for labor and for delivery. Provides continuous pain control through medication in the epidural catheter. It may be associated with a more pronounced motor block because higher doses of local anesthetic and opioid are used for pain relief.

Both tecniques can be associated to a risk of itching due to opioid use.

**Conclusions**

Both techniques are very valid for pain control in parturients; the choice depends on the patient's preferences, specific clinical indications, and the anesthesiological practice of the healthcare institution. It is up to the anesthesiologist and the patient to discuss together and make an informed decision based on their individual circumstances.

**Keywords**

Combined Spinal-Eidural Analgesia, Epidural analgesia, labor analgesia, pain releif, obstetric anesthesia, opioid, local anesthetic.

### A86 Obesity and pregnancy: an ongoing debate

#### S. Congedi^1^, M. Stella^2^, N. Sella^2^, A. Boscolo^1,2^, F. Calabrese^2^, P. Navalesi^1,2^

##### ^1^Department of Medicine (DIMED), University of Padua, Padua, Italy; ^2^Azienda Ospedale—Univerisità Padova, Padua, Italy

###### **Correspondence:** S. Congedi

*Journal of Anesthesia, Analgesia and Critical Care 2024*, **4(1):**A86

**Introduction**

Obesity, defined as Body Mass Index (BMI) > 30 kg/m2, is a growing global epidemic (1). Maternal obesity is a significant non-communicable disease risk factor, linked to a variety of adverse obstetric outcomes and higher cesarean delivery rate (2). Over general anesthesia, regional anesthesia can offer some advantages in laboring parturients, but in this specific population the higher amount of subcutaneous and epidural fat can pose a significant challenge to successful epidural catheter placement (3–4). However, specific data concerning the impact of obesity on labor anesthesia procedures are lacking. This study aims to investigate in a population of obese and non-obese pregnant patients: i) the positioning of the epidural catheter and ii) the requirement of operative vaginal delivery or cesarean section (CS) vs spontaneous delivery.

**Materials and Methods**

This single-center observational study evaluated for enrollment all consecutive adult laboring patients admitted at the obstetrics and gynecology service of the University Hospital of Padua, (between February and April 2023). Exclusion criteria were: i) intrauterine fetal deaths, ii) < 25th weeks of gestation, iii) elective CS, iv) lack of informed consent. Concerning pre-pregnancy BMI, the patients were enrolled in two different groups: 'non-obese' (BMI < 30 kg/m2) or 'obese' (BMI > 30 kg/m2) group. For each enrolled patient informed consent was obtained and the following data were collected: i) anthropometric features (age, pre-pregnancy BMI), ii)obstetric medical history, iii) features regarding the positioning of the epidural catheter (centimeters distance of the epidural space from the skin, number of attempts and bolus, adverse events such as vomit, nausea and dural puncture, time elapsed between the last bolus and delivery), iv) type of delivery and perinatal newborns outcomes (APGAR score, NICU recovery).

**Results**

191 patients were enrolled, of which 42 (22%) were obese with a mean pre-pregnancy BMI of 32.6 ± 4.1 kg/m2. No significant differences were detected among obese and no-obese groups regarding previous obstetric medical history and epidural catheter positing, except for the attempts needed. Pre-pregnancy BMI > 30 kg/m2 was associated with a higher effort required for a successful epidural catheter placement (p-value 0.04). Finally, no differences emerged in our cohort concerning delivery methods and perinatal newborns outcomes.

**Conclusions**

Despite previous results, in Our study no significant differences were detected in delivery methods between obese and non-obese patients. These results contribute to the ongoing debate on the impact of pre-pregnancy obesity on labor analgesia strategy, without leaving out the BMI relevance in the preoperative anesthesia evaluation.

**References**

1. Nguyen DM, El-Serag HB. The epidemiology of obesity. Gastroenterology Clinics of North America. 2010;39(1):1–7.
2. Gaillard R. Maternal obesity during pregnancy and cardiovascular development and disease in the offspring. Eur J Epidemiol. 2015;30(11):1141–1152.
3. Stiffler KA, Jwayyed S, Wilber ST, Robinson A. The use of ultrasound to identify pertinent landmarks for lumbar puncture. American Journal of Emergency Medicine. 2007 Mar;25(3):331–4.
4. Carvalho JCA. Ultrasound-facilitated epidurals and spinals in obstetrics. Anesthesiology Clinics. 2008;26(1):145–158

### A87 Trasmuscular – Quadratus Lumborum Block (T-QLB) VS Erector Spinae Plane Block (ESPB) in cesarean section—two valid analgesic alternatives to the epidural catheter?

#### D.B. Cecilia, G. Ranieri, F. Maria Grazia, R. Federico, T. Cristina, Z. Antonina, A. Marco, P. Francesca, F. Maria Cristina, P. Luca, N. Gabriella, A.U. de Siena, P. Ciocchetti

##### Ospedale ISOLA Tiberina—Gemelli Isola, Roma, Italy

###### **Correspondence:** D.B. Cecilia

*Journal of Anesthesia, Analgesia and Critical Care 2024*, **4(1):**A87

**Background**

Cesarean section (CS) is related to moderate or severe acute postoperative pain, with negative consequences on maternal and fetal well-being (1).

Ultrasound guided (US-g) locoregional anesthesia plays an important role in post-operative pain management. Erector Spinae Plane Block (ESP), and trasmuscular – Quadratus Lumborum Block (t-QLB) have been studied as alternatives to opioids administration with heterogeneous results regarding post-operative pain control (2–4).

This study aims to assess the analgesic efficacy of US-g t-QLB and thoracic ESP (tESP) compared to patient – controlled epidural analgesia (PCEA) in women undergoing CS under combined spinal – epidural anesthesia (CSE).

**Materials and methods**

This is a prospective mono-centric study. Parturients scheduled for elective CS, due to previous CS or maternal request, under CSE (intratecal administration of ropivacaine 10 mg + sufentanil 3 mcg + saline for a 5 ml total volume) were divided into three equal groups. Exclusion criteria was the patients’ deny for the study. After CS, a PCEA pump (0,05% Ropivacaine, Sufentanil 1 mcg/ml, total volume 200 ml – bolus of 8 ml with a lockout of 60 min) was connected to the epidural catheter. Then, in lateral position [Fig. 1–2], group A received t-QLB, group B received t-ESPB at T11; no block was performed in the group C. For each patient, the anesthetic block solution consisted in 0,3% ropivacaine + 4 mg dexamethasone in 20 mL of saline solution for each side.

The primary outcome was the pain verbal analogue scale (VAS) at 6, 12, 18, and 24 h after surgery. The secondary outcomes were: time to first required PCEA bolus (hours), the number of total PCEA doses performed, and the adverse events incidence after 24 h. Continuous parametric data were presented as mean and standard deviations (SD) and the non-parametric ones as median and interquartile range (IQR); ANOVA for parametric data and Kruskal–Wallis (K-W) for non-parametric ones were performed. Dichotomous data were presented as absolute and relative frequencies and compared with X^2^. The statistical significance was set at a p-value < 0.05.

**Results**

60 parturient were enrolled and divided in three groups of 20 patients. No differences were found for demographic and clinical history data between groups (Table 1). All the patients were admitted to the final analysis.

Group A and Group B felt less pain than group C at any time of evaluation (Fig. 3).

Group A and B needed less PCEA bolus compared to the group C (group A, median 4 (4–4); group B, median 5 ( 4–6); group C median 15 (13–15) – K-W 37.9; p < 0.01) (Fig. 4). Moreover, group C showed a lower first time PCEA request dose (Fig. 5).

T-QLB and t-ESP resulted in a lower adverse effects incidence compared to group C for the postoperative pain management (X^2^ 32.6; p < 0.01) (Fig. 6).

**Conclusions**

T-QLB and TESPB appear to be more effective then PCEA in reducing post-operative pain in the context of multimodal and opioid—sparing analgesia after CS.

More studies are needed to clarify the efficacy and safety of t-ESP and t-QLB in parturient undergoing CS.

**Fig. 1:**
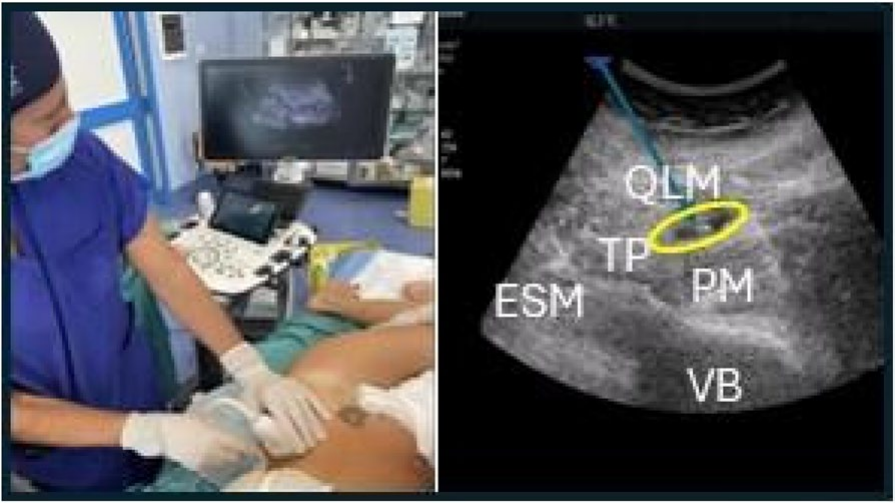
** (abstract A87).** Left: Pregnant in lateral position and t-QLB approach. Right: Sonoanatomy. Blue Arrow = needle path, Yellow circle = local anesthetic distribution; QLM = Quadratus Lumborum Muscle TP = Transverse Process, PM = Psoas Muscle. VB = Vertebral Body, ESM = Erector Spinae Muscle

**Fig. 2:**
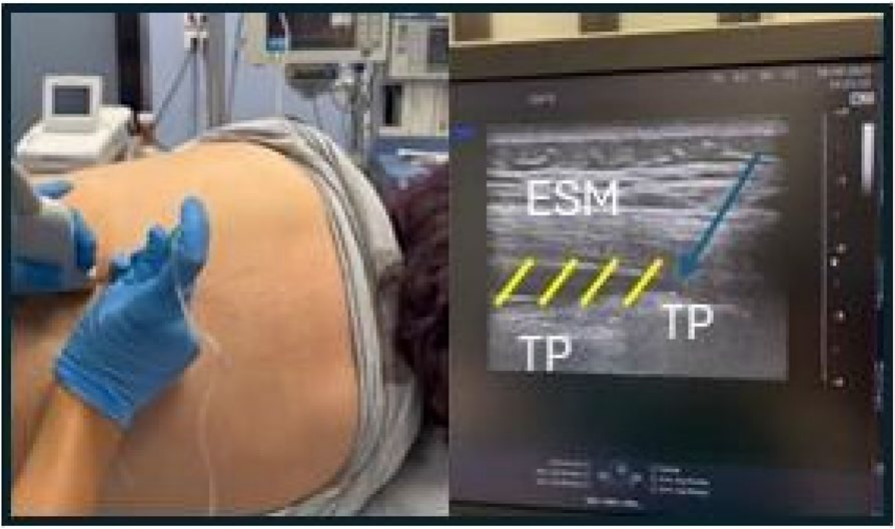
** (abstract A87).** Left: Pregnant in lateral position and TESPB in plane cranial to caudal Approach. Right: Sonoanatomy. ESM = Erector Spinae Muscle; Yellow Lines = Local Anesthetic Distribution in the plane between Erector Spinae Muscle and Trasverse Process; TP = Transverse Process

**Fig. 3:**
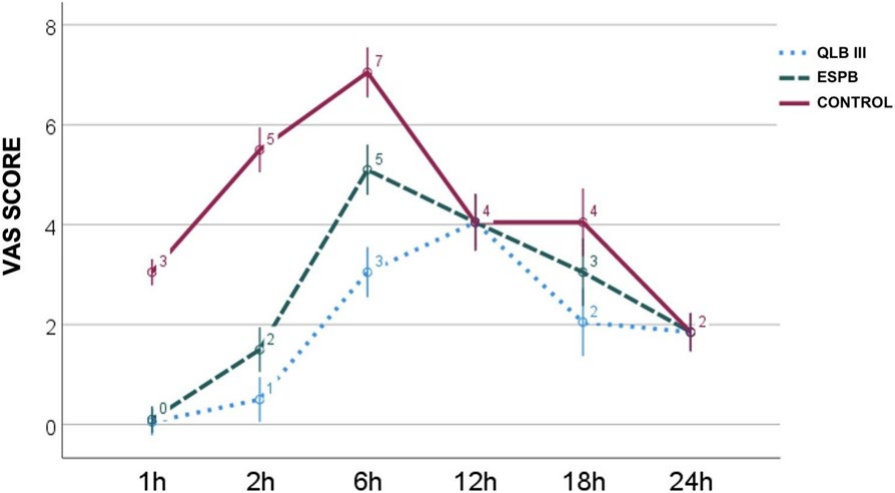
** (abstract A87).** Pain evaluation with VAS Score

**Fig. 4:**
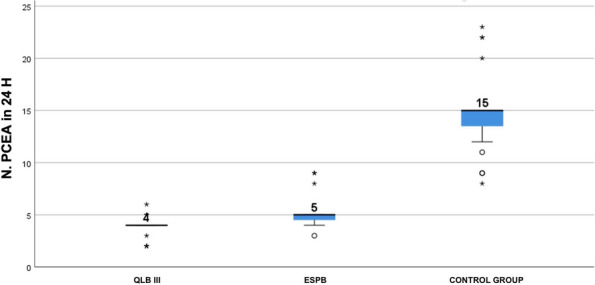
** (abstract A87).** PCEA boluses in the 24h post CS

**Fig. 5:**
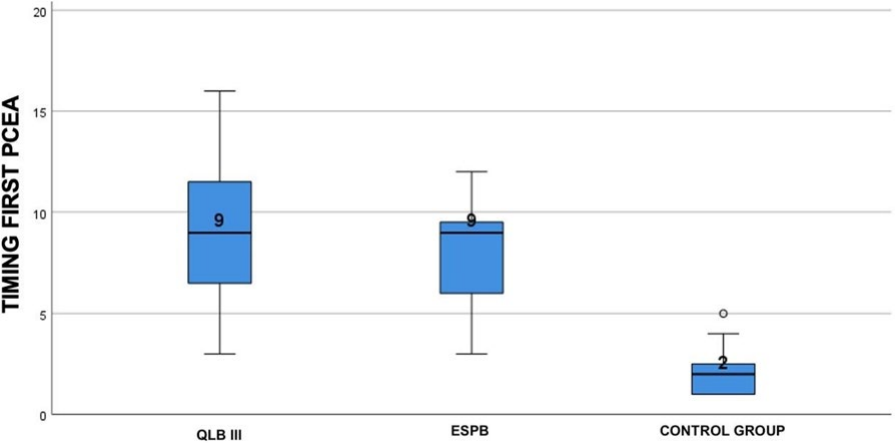
** (abstract A87).** Time for PCEA bolus

**Fig. 6:**
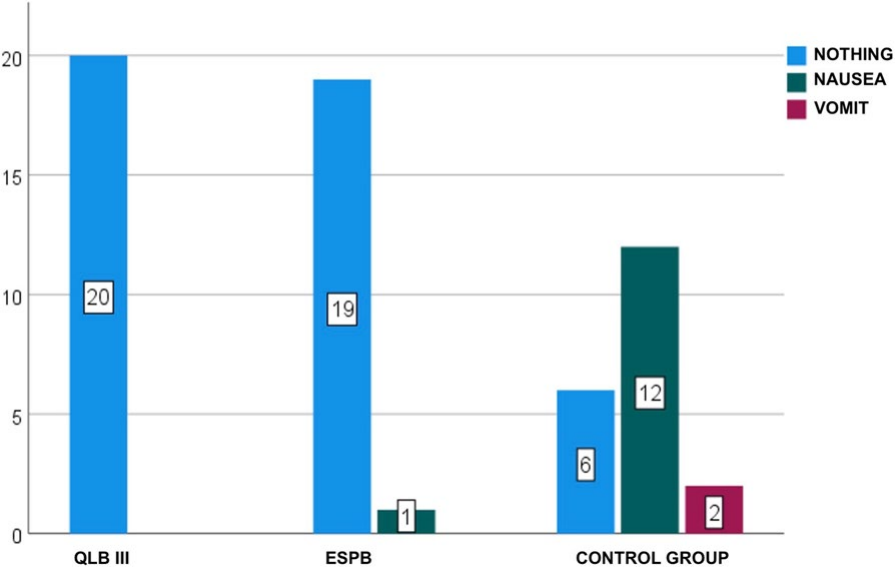
** (abstract A87).** Incidence of adverse effects

**Table 1:** **(abstract A87).** Demographic and clinical history data between groups

**N = 60**		**QLBIII (N = 20)**		**ESP (N = 20)**		**Control (N = 20)**			
		**N**	**(%)**	**N**	**(%)**	**N**	**(%)**	**χ**^**2**^	***p*-value**
**CS indications**	Previous CS	16	80.0%	17	85.0%	15	75.0%	0.63	0.732
	Maternal request	4	20.0%	3	15.0%	5	25.0%	0.63	0.732
**Comorbidities**	Total	11	55.0%	12	60.0%	11	55.0%	0.14	0.935
	Pregnancy Cholestasis	1	5.0%	1	5.0%	1	5.0%	0.00	1
	Gestational diabetes	3	15.0%	2	10.0%	1	5.0%	1.11	0.574
	Dysthyroidism	6	30.0%	8	40.0%	7	35.0%	0.44	0.803
	Gestational hypertension	0	0.0%	1	5.0%	1	5.0%	0.00	1
	Previous DVT	1	5.0%	0	0.0%	1	5.0%	0	1
		**Mean**	**SD**	**Mean**	**SD**	**Mean**	**SD**	**ANOVA**	***p*-value**
**Gestational age**	Week	38	1	38	1	38	1	0.00	1
**BMI**	kg/m^2^	26.2	2.6	27.0	2.6	25.3	2.4	2.25	0.115

*QLBIII* quadratum lumborum block III, *ESP* Erector spinae block, *CD* Cesarean delivery, *DVT* deep venous thrombosis, *SD* standard deviations, *BMI* Body Mass Index

## Paediatric and neonatal perioperative medicine

### A88 Reversed ultrasound-guided dorsal penile nerve block (RUS-DPNB): a new fascial ultrasound-guided dorsal penile nerve block in children

#### M. Lui^1^, M. Fischer^1^, D. Colosimo^2^, P. Rufini^2^, C. Greco^2^, E. Mongelli^2^, S. Cumbo^2^, Z. Ricci^2^

##### ^1^Department of Anesthesia and Critical Care Azienda Ospedaliero-Universitaria Careggi, University of Florence, Florence, Italy; ^2^ Anesthesia and Critical Care Meyer Children's Hospital IRCCS Florence Italy, University of Florence, Florence, Italy

###### **Correspondence:** M. Lui

*Journal of Anesthesia, Analgesia and Critical Care 2024*, **4(1):**A88

**Introduction:** Pain relief in children undergoing urological surgery is often an unmet critical need. Pediatric penile surgery is characterized by elevated intra and post-operative pain levels. Currently, blind landmark-based dorsal penile nerve block (DPNB) is still widely adopted. Literature data report a significative failure rate as well as potentially disabling complications adopting this technique. Novel ultrasound (US)-guided nerve blocks have recently been proposed and gradually introduced in clinical practice. Reversed ultrasound-guided DPNB (RUS-DPNB) represents a new alternative but studies proving its safety and efficacy are still needed.

Objective: we aimed to evaluate the application of the RUS-DPNB vs other available regional anesthesia techniques in our center in a population of pediatric patients undergoing minor penile surgery. As secondary objectives we attempted to evaluate RUS-DPNB effectiveness for intra and post-operative pain relief in terms of VAS/FACES scales after 3, 6 and 24 h from surgery; emergence delirium complication was collected as well.

**Study design**: an observational prospective monocentric study of 57 pediatric (< 18 years) patients, classified ASA 1–2, undergoing minor penile surgery (< 2 h), admitted at Meyer Children’s Hospital between February and May 2024. Local ethics committee approved the study and informed consent was collected from patients’ relatives. Patients were divided in two groups: RUS-DPNB vs other regional anesthesia techniques (choice based on operators’ decisions). Clinical and hemodynamic parameters were monitored intraoperatively; need of additional analgesic drugs was noted on record. Patients were later assessed via VAS or FACES scale application at pre-set times of 3, 6 and 24 h after surgery.

**Results**: overall, 57 pediatric patients were included, 33 of them underwent RUS-DPNB and 24 other regional techniques (10 Caudal, 8 Dalens, 6 Pudendal). Median age and weight resulted 4 years (IQR 2–7) and 22 kg (IQR 15–36), respectively. Median basal hemodynamic values were 100 bpm for HR (IQR 86–120) and 99 mmHg (IQR 90–110) for Mean Blood Pressure (MBP) without significant differences between groups. Nerve Block’s execution resulted significantly longer for RUS-DPNB vs other: 2 min (IQR 2–5) vs. 2 min (IQR 1–2) (p 0.01). The levobupivacaine dose pro-Kg was significantly lower in the RUS-DPNB group: 0.34 (IQR 0.25–0.38) vs. 0.67 (IQR 0.53–1.98) mg/kg (Fig. 1). Overall, 6 patients in the RUS group (18%) and 7 (29%) in the non-RUS group required additional fentanyl (1 mcg/kg) administration for inadequate pain control (p = 0.35 at Fisher’s exact test) (Fig. 2). Nonetheless, we reported a positive delirium (> 12 pt) score for 8 RUS-DPNB patients (24%) vs 1 (4%) in other blocks group (p 0.06). In the postoperative phase VAS/FACES scores resulted significantly lower, over time in the RUS patients (p 0.04) (Fig. 3).

**Conclusion**: In a heterogenous group of children undergoing penis surgery, the RUS-DPNB allowed the administration of significantly lower local anesthetic doses with acceptable post operative pain levels with respect of other techniques pooled together. A subgroup analysis on simple circumcision enrollment completion is warranted.

**Fig. 1:**
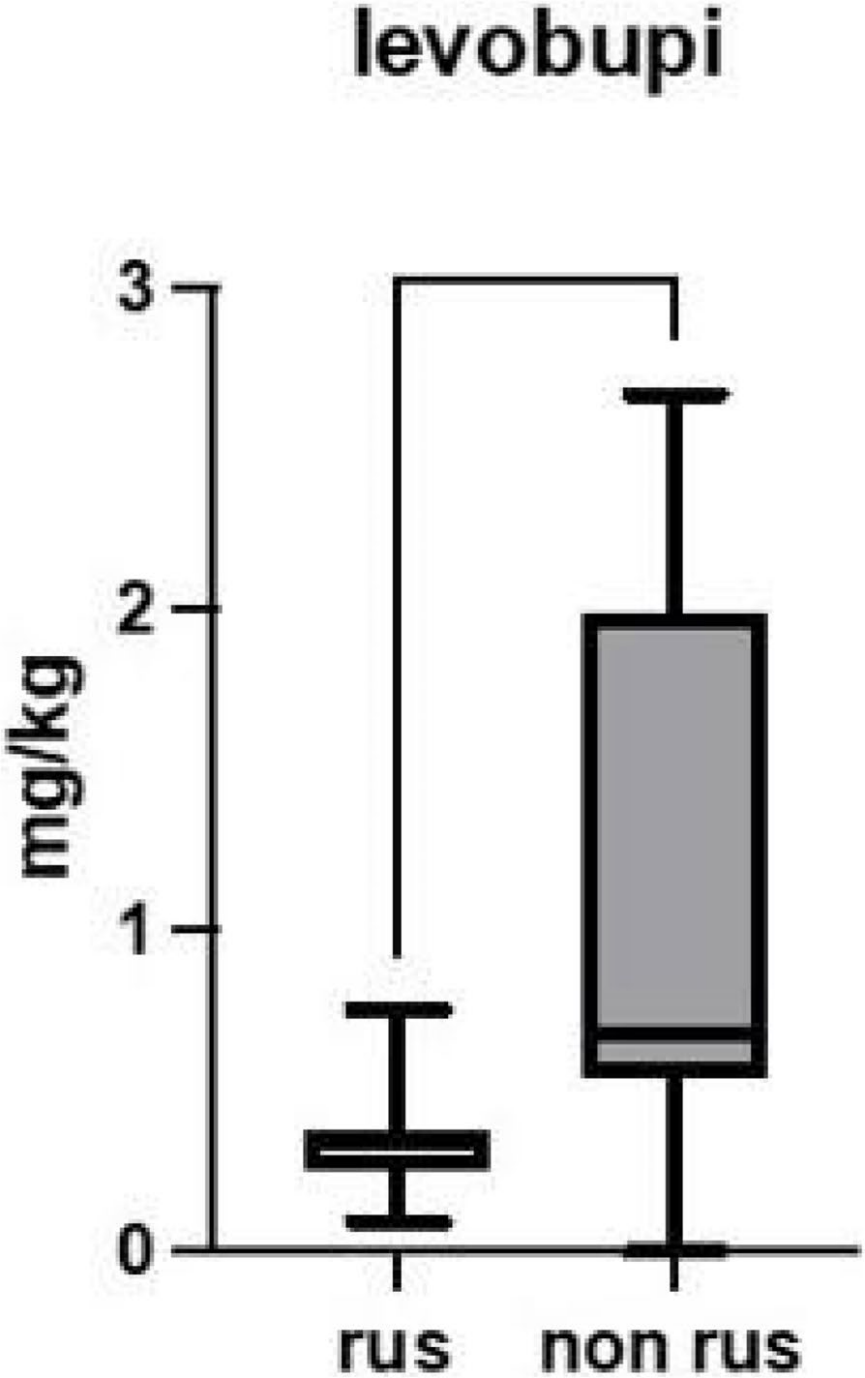
** (abstract A88).** See text for description

**Fig. 2:**
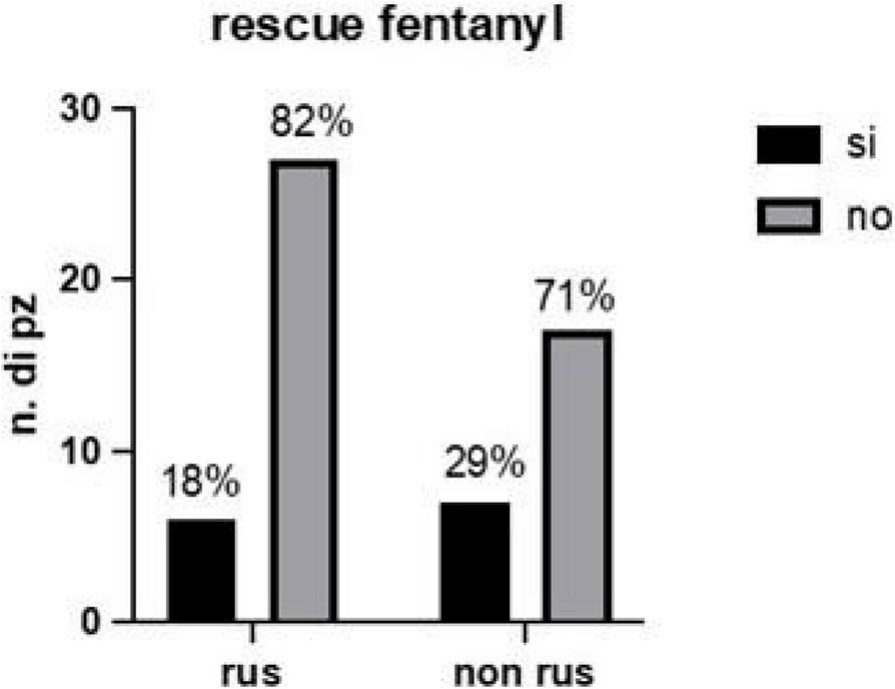
** (abstract A88).** See text for description

**Fig. 3:**
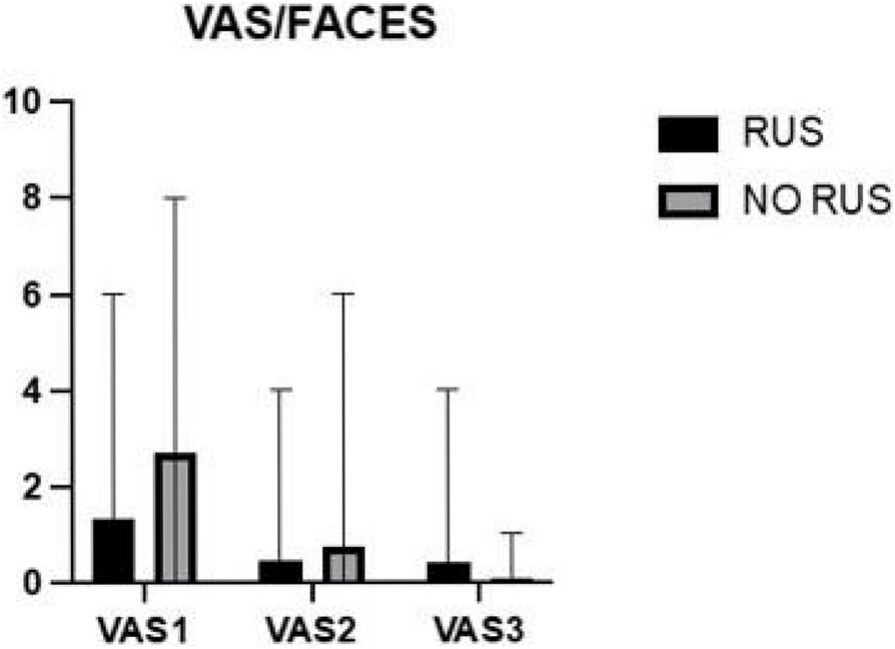
** (abstract A88).** See text for description

## Veterinary anaesthesia

### A89 Effect of vatinoxan on anesthetic parameters in guinea pigs (*Cavia Porcellus*) undergoing orchiectomy

#### M. Serpieri, G. Bonaffini, C. Ottino, G. Quaranta, M. Mauthe von Degerfeld

##### Centro Animali Non Convenzionali (CANC)—Dept. of Veterinary Sciences, University of Turin, Grugliasco (TO), Italy

###### **Correspondence:** M. Serpieri

*Journal of Anesthesia, Analgesia and Critical Care 2024*, **4(1):**A89

Alpha2-adrenoceptor agonists, such as medetomidine, are frequently employed with dissociatives in veterinary anesthesia. However, these agents can elicit cardiovascular adverse effects, including vasoconstriction and bradycardia, potentially compromising tissue perfusion. Those effects could impair anesthesia in guinea pigs, with already are more susceptible to anesthetic risk compared to dogs. The vasoconstrictive impact of medetomidine can be mitigated by vatinoxan, a selective alpha2-adrenoceptor antagonist with limited central nervous system penetration, confining effects to tissues outside the blood–brain barrier. Recently, Zenalpha®, a novel formulation comprising medetomidine and vatinoxan in a fixed ratio (1:20), has been introduced. This study aimed to evaluate vatinoxan's effects in Zenalpha® on guinea pigs undergoing orchiectomy with a ketamine- medetomidine protocol.

Twenty-four guinea pigs were divided into two groups. Baseline heart rate (HR), respiratory rate (RR), and rectal temperature (T°) were recorded (T0). Anesthesia was induced with intramuscular ketamine (40 mg/kg) and either medetomidine (0.4 mg/kg, Group KM) or medetomidine-vatinoxan (Zenalpha®: 0.4 mg/kg—8 mg/kg, Group KZ). Time to loss of righting reflex (LRR) was noted, and vital signs (HR, RR, peripheral oxygen saturation [SpO2], and T°) were monitored every 5 min. Orchiectomy via a scrotal approach followed, with surgical duration recorded. Subsequently, atipamezole (2 mg/kg) was administered intramuscularly, and head lift time (HL) and time to resumption of righting reflex (RRR) were recorded.

Between-group comparisons were made using Wilcoxon rank sum test, while within-group variations between baseline and T5 were assessed using Wilcoxon signed-rank test for HR and T°.

No significant between-group differences were noted. However, Group KM exhibited a notable decrease in HR (T0-T5) (p = 0.006), unlike the Group KZ (p = 0.116). Smooth recovery without complications was observed in all animals. Tables 1 and 2 present data on LRR, surgical time, HL, RRR, HR, RR, SpO2, and T°.

The absence of significant between-group differences implies that Zenalpha® did not significantly affect cardiovascular parameters in this guinea pig cohort, despite a more marked HR reduction in the KM group versus baseline. Both groups maintained stable anesthetic parameters with no complications. However, vatinoxan's inclusion did not produce clinically meaningful benefits in the ketamine-medetomidine combination. The 1:20 medetomidine-to-vatinoxan ratio may not elicit effects in guinea pigs similar to those observed in dogs, and further studies on dosages and drugs’ ratios would be needed.

**Trial registration**

Protocol n° 0000210/2024, University of Turin.

**References**

1. Jalanka HH, Roeken BO. The use of medetomidine, medetomidine-ketamine combinations, and atipamezole in nondomestic mammals: a review. J Zoo Wildl Med. 1990; 259–282.
2. Pypendop BH, Verstegen JP. Hemodynamic effects of medetomidine in the dog: a dose titration study. Vet Surg. 1998; 6:612–22.
3. Bennett K, Lewis K. Sedation and Anesthesia in Rodents. Vet Clin North Am Exot Anim Pract. 2022; 25:211–255.
4. Salla KM, Turunen HA, Kallio-Kujala IJ, Pekkola V, Casoni DC, Lepajoe J, Björkenheim P, Raekallio MR, Vainio O. Effects of vatinoxan in dogs premedicated with medetomidine and butorphanol followed by sevoflurane anaesthesia: a randomized clinical study. Vet Anaesth Analg. 2022; 49:563–571.

**Table 1:** ** (abstract A89).** Results for loss of righting reflex (LRR), surgical time, head lift time, and resumption of righting reflex (RRR) in groups KM and KZ

Variabili	Group KM	Group KZ	*p* value
LRR (min)	3 (2–3)	3(2–4)	0,976
Surgical time (min)	13 (9–14)	12 (10–16)	0,974
HL (min)	9 (6–14)	12 (10–14)	0,228
RRR (min)	25 (24–27)	25 (20–28)	1

**Table 2:** ** (abstract A89).** Results for heart rate (HR), respiratory rate (RR), peripheral oxygen saturation (SpO2), and rectal temperature in groups KM and KZ

	Time Points			
Variable	T0	T5	T10	T15
HR (beats/min)				
Group KM	250 (240–283)	205 (190–222)	202 (183–211)	197 (181–203)
Group KZ	218 (189–260)	100 (193–203)	188 (184–194)	185 (176–192)
p value	0,156	0,509	0,248	0,119
RR (breaths/min)				
Group KM	120 (99–153)	58 (48–88)	52 (44–77)	48 (40–59)
Group KZ	126 (103–163)	54 (48–68)	48 (47–64)	48 (47–61)
p value	0,623	0,639	0,467	0,861
SpO2 (%)				
Group KM	-	92 (93–95)	100 (100–100)	100 (99–100)
Group KZ	-	96 (94–100)	100 (100–100)	100 (98–100)
p value	-	0,09	1	0,401
T° (°C)				
Group KM	37,9 (37,8–38,3)	37,5 (36,7–38,2)	37,5 (36,0–38,2)	37,0 (36,2–38,1)
Group KZ	38,1 (37,5–38,7)	37,9 (36,4–38,5)	37,6 (36,4–38,3)	37,2 (36,4–38,0)
p value	0,645	0,698	0,476	0,757

### A90 Corneal lesions and tear film evaluation in dogs in general anesthesia for non-ophthalmic surgeries receiving two different treatments for corneal protection

#### C. Di Palma, F. Micieli, M. Saggiomo, V. Palumbo, B. Lamagna

##### Dipartimento di Medicina Veterinaria e Produzioni Animali, Università degli Studi di Napoli Federico II, Napoli, Italy

###### **Correspondence:** C. Di Palma

*Journal of Anesthesia, Analgesia and Critical Care 2024*, **4(1):**A90

**Background**

Corneal abrasions are the most common ocular complication in humans during general anesthesia (GA) for non-ophthalmic surgery. Anesthesia induced corneal lesions have also been reported in veterinary medicine (1, 2).

**Objective**

To evaluate the quality and production rate of precorneal tear film, and to determine the incidence of corneal lesions in dogs receiving two different ocular treatments during GA for non-ophthalmic surgeries: 0,25% hyaluronic acid eye drops or topical 2% pilocarpine instillation.

**Study design**

Blind, prospective, clinical study (PG/2021/0000969; 07/01/2021).

**Materials and Methods**

An ophthalmological examination (slit-lamp biomicroscopy, STT-1, BUT, IOP, fluorescein-lissamine green staining, tear osmolarity) was performed before preanesthetic medication in dogs undergoing elective surgery. The consent of the dog owners was required. Dogs were randomly allocated to receive 0,25% hyaluronic acid (GH) or 2% pilocarpine (GP) as topical ocular treatment during GA. STT-1 was performed immediately after intubation (T-int) and hourly (T-1 h, T-2 h) until the end of anesthesia, just before instilling one drop of the assigned treatment according to randomization. The same ophthalmologist, blind to the treatment, performed the complete ophthalmological examination after extubation (T-est) and 24 h after GA (T-24 h).

**Results**

Thirty dogs (60 eyes) were enrolled. STT-1 values showed a decrease in both groups immediately after intubation, but GP showed a rapid increase in tear production with a significant difference between the groups at T- 1 h, T-2 h and T-est. No statistically significant differences were found between the two groups for the other values (BUT, IOP, tear osmolarity). No corneal ulcerations were detected, while 11.7% of eyes at T-est and 15% at T-24 h presented corneal abrasions. There were no statistically significant differences between the two groups in the incidence of corneal abrasions.

**Conclusions**

The topical eye treatments tested prevent corneal ulcers. According to the medical literature, several prophylactic strategies can prevent exposure keratopathy by using lubricant eye drop formulations during GA (3). The topical pilocarpine administration increased tear production but did not eliminate corneal complications during GA. In our study, tear osmolarity did not change after the exposure keratopathy associated with GA.

**References**

1. Dawson C, Sanchez RF. A prospective study of the prevalence of corneal surface disease in dogs receiving prophylactic topical lubrication under general anesthesia. Vet Ophthalmol. 2016 Mar;19(2):124–9.
2. Scarabelli S, Timofte D, Malalana F, Bardell D. Corneal abrasion and microbial contamination in horses following general anaesthesia for non-ocular surgery. Vet Anaesth Analg. 2018 May;45(3):278–284.
3. Grixti A, Sadri M, Watts MT. Corneal protection during general anesthesia for nonocular surgery. Ocul Surf. 2013 Apr;11(2):109–18.

### A91 Butorphanol-alfaxalone-midazolam-sevoflurane anaesthesia in dogs suffering from various cardiac pathologies undergoing cardiac computed tomography: a case series

#### M. Moretti, G. Bertolini, A. Costa, E. Bortolami

##### Clinica Veterinaria San Marco, Veggiano, Padova, Italy

###### **Correspondence:** M. Moretti

*Journal of Anesthesia, Analgesia and Critical Care 2024*, **4(1):**A91

**Background**

Several publications evaluated cardiac computed tomography (CCT) in dogs under general anaesthesia [1, 2, 3, 4], but none of them focused on the anaesthetic protocol.

**Case series**

Twenty-three client-owned dogs (11 males, 12 females; ASA III or IV, body weight and age 29.2 ± 16.4 kg and 101.1 ± 45.4 months, respectively) of different breeds underwent an electrocardiogram gated CCT. Animals were sedated with intramuscular butorphanol (0.3 mg/kg); anaesthesia was induced with intravenous alfaxalone to effect and midazolam (0.1 mg/kg) and maintained with sevoflurane delivered in air and oxygen. Ringer's lactate was infused intravenously (5 ml/kg). Volume controlled ventilation with tidal volume set at 8–12 ml/kg was used. During anesthesia, heart rate (HR), respiratory rate, invasive arterial blood pressure, oxygen saturation, end-tidal carbon dioxide (ETCO2), and end-tidal sevoflurane concentration were monitored. Butorphanol premedication resulted in moderate sedation and decreased HR; alfaxalone dose was 1.2 ± 0.3 mg/kg; induction of anaesthesia was smooth and in 20 patients caused a self-limiting increase in HR, without hypotension. Ranges of cardiovascular variables were as follows: HR (33–188 bpm), mean arterial pressure (50–108 mmHg). Oxygen desaturation was not reported. Self- limiting (mainly ventricular) cardiac arrhythmias (10/23), moderate hypotension (7/23) and hypertension (1/23) were reported during anaesthesia. Mean duration of anaesthesia was 54 ± 10 min. Quality of recovery was considered good. One patient was euthanized one hour after the CCT. CCT images were free of motion artifacts in 20/23 of the exams.

**Conclusions**

This anaesthetic protocol provided good haemodynamic stability in dogs with heart disease.

**References**

1. Kim J, Lee S, Hwang J, Yoon J. Clinical utility of a new protocol of cardiac computed tomography in dogs. Vet Med Sci. 2023;9(2):645–652.
2. To A, Hostnik ET, Rhinehart JD, Scansen BA. Electrocardiography-gated cardiac CT angiography can differentiate brachycephalic dogs with and without pulmonary valve stenosis and findings differ from transthoracic echocardiography. Vet Radiol Ultrasound. 2019;60(2):145–158.
3. Auriemma E, Armienti F, Morabito S, Specchi S, Rondelli V, Domenech O, Guglielmini C, Lacava G, Zini E, Khouri T. Electrocardiogram-gated 16-multidetector computed tomographic angiography of the coronary arteries in dogs. Vet Rec. 2018;183(15):473.
4. Scollan KF, Bottorff B, Stieger-Vanegas S, Nemanic S, Sisson D. Use of multidetector computed tomography in the assessment of dogs with pericardial effusion. J Vet Intern Med. 2015;29(1):79–87.

## Follow-up/outcomes

### A92 Collecting data of Occupational Stress Indexes (OSI) within the Intensive Care Unit (ICU)

#### F. Alfonsi^1^, C.M. Petrangeli^1^, R. Giordano^1^, F. Semenzato^2^, F.M. Petrangeli^3^, F. Claro^1^, V. De Angelis^1^, F. Frisardi^1^, S. Verrengia^1^, I. Brandolini^1^, D.J. Brunetti^1^, M. Silvi^1^, R. Adorno^1^, M. Martucci^1^, T. Galli^1^, C. Manni^1^, M. Bernardo^1^, G. Onori^1^, D. Cipollone^1^, F. Leonardis^1^

##### ^1^UOSD Terapia Intensiva, Policlinico Tor Vergata, Roma, Italy; ^2^Facoltà di Medicina e Chirurgia, Università Tor Vergata, Roma, Italy; ^3^Dipartimento di Matematica, Università Sapienza, Roma, Italy

###### **Correspondence:** C.M. Petrangeli

*Journal of Anesthesia, Analgesia and Critical Care 2024*, **4(1):**A92

**Background**

The quality life of Intensive Care Unit Doctors (ICUDocs) can be affected by a set of several factors: burnout, physical stress, psychological stress, long working hours, heavy workload, sudden and continuous management of emergencies, emotional load, continuous confrontation with death. These are fundamental aspects in determining the increase of the level of occupational stress, as well as assessing the quality of life in terms of physical, emotional, social demotions, in addition to the occupational ones.

Our study aims to identify the areas of greatest occupational stress, with the objective to promote methods of work and support for the ICUDocs, enhancing the quality of professional time while reducing related stress factors.

**Materials and methods**

The Occupational Stress Indicator self-administered test is structured into seven sections: a biographical questionnaire and four specific sections—stress sources, individual characteristics, coping strategies, and stress effects. Furthermore, each section is divided into subcategories that measure different levels of stress.

In the study were included ICUDocs with > or < 10 years of activity in the ICU, divided by gender.

From the data collection, personal and group profiles were produced to investigate the phenomenon of occupational stress. Effective analysis and treatment of stress are important aspects of managing medical resources.

**Results**

The study highlights that ICUDocts report low quality of life and emotional fatigue. The four areas investigated appear to be correlated, the individual characteristics (in the six areas of investigation) are determinant in the activated stress areas and are related to various coping strategies. The area of relationships with other people is found to be significantly related to the ability to manage clinical emergencies.

**Reference**

1. C. L. Cooper, S. J. Sloan, S. Williams: Occupational Stress Indicator (Italian edition: S. Sirigatti, C. Stefanile). Organizzazioni speciali 2002.

## New technologies for diagnosis and treatment

### A93 Release velocity improvement with a new metronome-guided chest compression protocol: results of the multicenter ritmico study

#### R. Cresta^1^, M.L. Caputo^2^, G. Monachino^3^, S. Tomola^4^, P. Pinetti^4^, P.L. Ingrassia^4^, A. Cortegiani^5,6^, M. Ippolito^5^, A. Gargano^5^, C. Metelmann^7^, B. Metelmann^7^, C.R. Hölzing^7^, A. Currao^8,9^, E. Baldi^8,9^, S. Savastano^8,9^, J. Ganter^10^, M. Müller^11^, C. Benvenuti^1^, A. Auriccchio^2^, F. Faraci^3^

##### ^1^Ticino Cuore Foundation, Lugano, Switzerland; ^2^Cardiocentro Ticino Institute, Lugano, Switzerland; ^3^University of Applied Sciences and Arts of Southern Switzerland (SUPSI), Department of Innovative Technologies (DTI), Lugano, Switzerland; ^4^Centro di Simulazione (CeSi), Centro Professionale Sociosanitario Medico-Tecnico, Lugano, Switzerland; ^5^Department of Anesthesia, Analgesia, Intensive Care and Emergency. University Hospital Policlinico Paolo Giaccone, Palermo, Italy; ^6^Department of Precision Medicine in Medical, Surgical and Critical Care (Me.Pre.C.C.), Palermo, Italy; ^7^Department of Anaesthesia, Intensive Care Medicine, Emergency Medicine and Pain Medicine, University Medicine Greifswald, Greifswald, Germany; ^8^Division of Cardiology, Fondazione IRCCS Policlinico San Matteo, Pavia, Italy; ^9^Cardiac Arrest and Resuscitation Science Research Team (RESTART), Fondazione IRCCS Policlinico San Matteo, Pavia, Italy; ^10^Department of Anaesthesiology and Critical Care, Medical Center; University of Freiburg, Faculty of Medicine, Freiburg, Germany; ^11^Department of Anaesthesiology and Critical Care and Emergency Medicine, St. Josefs Hospital, Freiburg, Germany; ^12^Ticino Canton Emergency Medical Services Federation (FCTSA), Bellinzona, Switzerland

###### **Correspondence:** R. Cresta

*Journal of Anesthesia, Analgesia and Critical Care 2024*, **4(1):**A93

**Background:** The emergency medical services treat over 350,000 out-of-hospital cardiac arrests in the U.S. and over 250,000 in Europe every year. Outcomes vary widely and quality of cardiopulmonary resuscitation (CPR) is fundamental to increase survival. The chest release phase is essential to obtain adequate heart and brain perfusion [1]. However, no studies describe how to improve this parameter during resuscitation.

**Materials and methods**: We developed and validated a double-click metronome to improve chest release velocity during CPR. The new metronome uses two different tone pitches. The first click corresponds to the end of chest compression and the second to the end of chest release. Subjects with different levels of expertise in CPR and with a valid basic life support & defibrillation (BLS-D) certification have been included in the study from 5 European centers. All subjects signed the informed consent. In a randomized protocol, each subject performed two rounds of 4-min chest compressions on a manikin starting either with the standard or the modified metronome. CPR quality was monitored continuously during simulations through an automated external defibrillator (AED) with an integrated feedback device which recorded three CPR parameters: compression depth, rate and chest release velocity. CPR simulation was guided only by the external metronome, all audio prompts from the AED were silenced. CPR parameters were compared using Wilcoxon signed-ranks test. The impact of subject-specific factors and of the simulations order on the difference in CPR quality was evaluated using stepwise multilinear regression.

**Results:** 503 volunteers were included in the study (age: 34.4 + -11.6 years; sex: 54.5% male). Comparison between the two procedures (Fig. 1) showed a significant increase in median chest compression depth (+ 2.49%, p < 0.001), rate (+ 0.09%, p < 0.001), and release velocity (+ 1.51%, p < 0.001) with the modified metronome. The order of the two simulations turned out to be an influencing factor. Chest compression depth increased more when using the modified metronome for the second simulation. Chest release velocity and compression rate increased more when it was used first.

**Conclusions:** The double-click metronome significantly increased CPR quality in terms of compression depth and release velocity. The increase in compression rate (even if inside guidelines intervals) and the influence of the simulations order could be due to the lack of familiarity with the new rhythm and/or to muscular fatigue and needs further evaluation. Our study represents the first trial trying to develop a specific protocol to improve chest compression release velocity. Further demonstration of the efficacy of this system in real resuscitations is necessary to assess the potential impact on the outcome of out-of-hospital cardiac arrest.

**Grant**

Swiss Heart Foundation Grant Reference number FF22024.

**Reference**

1. Kovacs, A., Vadeboncoeur, T. F., Stolz, U., Spaite, D. W., Irisawa, T., Silver, A., & Bobrow, B. J. (2015). Chest compression release velocity: Association with survival and favorable neurologic outcome after out-of-hospital cardiac arrest. Resuscitation, 92, 107–114. 10.1016/j.resuscitation.2015.04.026

**Fig. 1:**
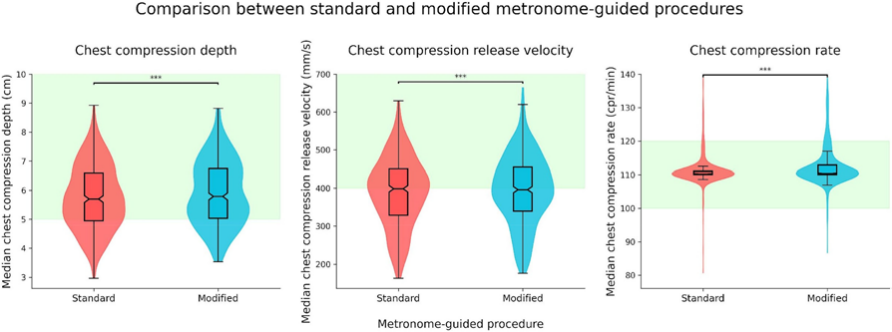
**(abstract A93).** Comparison between standard and modified metronome-guided procedures

### A94 A novel device for the assessment of cough in critically ill patients: a pilot in vitro and healthy volunteer study

#### A. Schiavi^1^, A. Collalti^1^, T. Pettenuzzo^2^, F. Zarantonello^2^, P. Navalesi^2^

##### ^1^Department of Medicine, University of Padua, Italy, Padova, Italy; ^2^Institute of Anesthesia and Intensive Care, University Hospital of Padua, Italy, Padova, Italy

###### **Correspondence:** A. Schiavi

*Journal of Anesthesia, Analgesia and Critical Care 2024*, **4(1):**A94

**Background**

Extubation success requires patients to be able to cough and clear airway secretions. Current clinical tools to assess the efficacy of cough suffer from some limitations (1). To address this gap, we designed a microcontroller-powered accelerometer and gyroscope measuring, based on the second law of motion, the angular velocity and acceleration and positioned on the distal end of a ventilatory interface. The aim of this pilot study is assessing the performance of this device through the comparison with an experimental surrogate of the peak cough expiratory flow (PCEF) generated in an in vitro and in vivo setting.

**Materials and methods**

The in vitro setup included a cough assist device (ATOS 70, Vitalaire Italia) generating different PCEF rates by adjusting inspiratory and expiratory pressures, inspiratory and expiratory times, and the inspiratory pause.

The device was connected to a test lung via a standard ventilator tubing, integrated with a flowmeter (FluxMed GrE, MBMed), a heat and moisture exchange (HME) filter, and the experimental accelerometer/gyroscope. Data were captured and processed by a microcontroller and a custom Python code (Python Software Foundation). Eleven settings were evaluated, each repeated 10 times for a total of 110 data points.

The in vivo experiment was conducted on two healthy volunteers. A face mask was positioned and the subject was asked to cough 10 times with each of three subjective strength levels (low, medium, and high) for a total of 30 data points. The face mask was connected, in series, to the HME filter, the flowmeter, and the accelerometer/gyroscope.

The outcome variables measured by the accelerometer/gyroscope were, for both acceleration (g) and angular velocity (deg/s), energy-acc (maximal acceleration on one of the axes), abs-acc (sum of the three axes’ absolute acceleration values), energy-rot (maximal perturbation angular velocity on one of the axes), abs-rot (sum of the three axes’ absolute velocity values), and the perturbation power spectral density (W/Hz).

The correlation between PCEF and each of the six output variables was analyzed by Spearman correlation coefficient. We fitted linear mixed-effect models to test the association between PCEF and each of the six output variables, including setting and subject as random effects for the in vitro and in vivo setting, respectively.

**Results**

In the in vitro study (Fig. 1 ), we observed a significant and moderate-to-strong correlation between PCEF and each of the outcome variables. In the linear mixed-effect model, PCEF was significantly associated with each of the output variables (Table 1). Similar findings were found in the in vivo tests (Fig. 2, Table 2).

**Conclusions**

In both the in vitro and in vivo tests, we found a significant correlation and association between the measurements provided by the experimental accelerometer/gyroscope and PCEF. Further studies are needed to confirm these findings and assess whether this device may offer a non-invasive means to study the characteristics of cough in intubated and non-intubated critically ill patients, potentially aiding in assessments of readiness for extubation.

Informed consent was taken from the two test subjects.

**Fig. 1:**
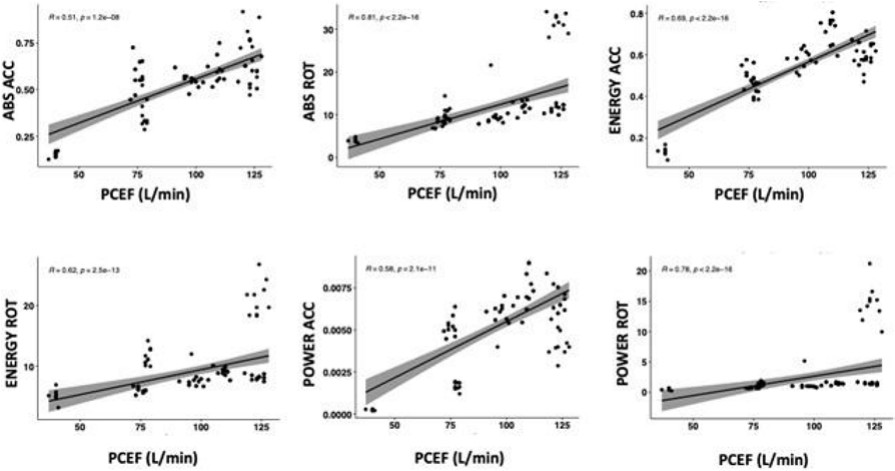
**(abstract A94).** See text for description

**Fig. 2:**
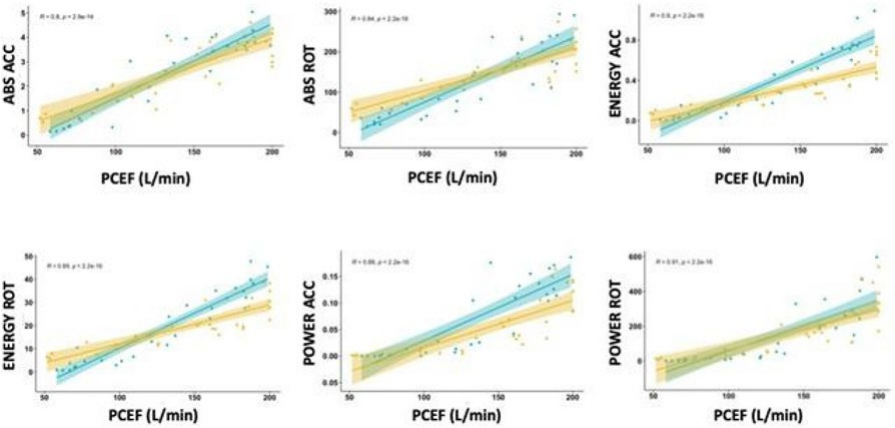
**(abstract A94).** See text for description

**Table 1:** ** (abstract A94).** Linear mixed effects model results for in vitro study

OUTCOME VARIABLES	ESTIMATE (STANDARD ERROR)	*P*-VALUE
Abs Acc	0.026 ± 0.001	< 0.001
Energy acc	0.005 ± 0.000	< 0.001
Abs rot	1.345 ± 0.109	< 0.001
Energy rot	0.232 ± 0.016	< 0.001
Power acc	0.001 ± 0.000	< 0.001
Power rot	2.614 ± 0.245	< 0.001

Estimates (standard errors) and p-values from the linear mixed-effect models to test the association between the surrogate of the peak cough expiratory flow and each of the six output variables, including setting as random effect. *P*-values < 0.05 were considered statistically significant

**Table 2:** **(abstract A94).** Linear mixed effects model results for in vivo study

OUTCOME VARIABLES	ESTIMATE (STANDARD ERROR)	P-VALUE
Abs acc	4.547e-03 ± 6.756e-04	< 0.001
Energy acc	5.107e-03 ± 4.984e-04	< 0.001
Abs rot	0.244 ± 0.024	< 0.001
Energy rot	0.129 ± 0.016	< 0.001
Power acc	5.935e-05 ± 7.549e-06	< 0.001
Power rot	0.102 ± 0.015	< 0.001

Estimates (standard errors) and p-values from the linear mixed-effect models to test the association between the surrogate of the peak cough expiratory flow and each of the six output variables, including the healthy volunteer as random effect. P-values < 0.05 were considered statistically significant

### A95 A new semi-automatic ct-related technique for diagnosis of pleural effusion in critically ill patients with ards

#### S. Pilloni^1^, G. Olla^1^, A. Usai^1^, R. Prost^2^, M. Pinna^2^, F.M. Loddo^1^

##### ^1^SC Anestesia E Rianimazione, ASL 4 Ogliastra, Lanusei, Italy; ^2^SC Radiologia Ospedaliero-TERRITORIALE, ASL 4 Ogliastra, Lanusei, Italy

###### **Correspondence:** S. Pilloni

*Journal of Anesthesia, Analgesia and Critical Care 2024*, **4(1):**A95

**Background**

Pleural effusion is common in critically ill patients admitted to intensive care unit (ICU). Thoracentesis may improve respiratory status, however, indications for this procedure are unclear [1]. Thoracic ultrasound (TUS) guidance is strongly recommended for diagnosis and treatment of all pleural effusions. Ultrasonographically significant pleural effusion (separation between parietal and visceral pleurae > 20 mm) may undergo thoracentesis. TUS is essential during thoracentesis and chest tube drainage as it increases safety and decreases life-threatening complications [2].

## Materials and methods

From 2022 to 2024, 44 thoracenteses were performed in our intensive care unit on 44 patients hospitalized for pneumonia, septic shock and polytrauma. Of these, 16 (13 males and 3 females) had pleural effusion associated with severe ARDS for which they had undergone High Resolution Computed Tomography (HRCT). Unenhanced HRCT images were acquired with patient in the supine position (arms on the sides, free breathing). CT examinations were performed using a 64-slice scanner. The original images were loaded into image analysis software. The software’s automated lung volume extraction was evaluated and manually corrected by radiologist, as to match the chest cavity. After automated separation of right and left lung, attenuation analysis of the masked volume was launched.

## Results

Generated attenuation map was qualitatively evaluated for correspondence with the lung and soft tissue window of the scan, then the volumes of the whole chest cavity, consolidated parenchyma and pleural effusion were extracted on each side. The data have been collected in Table 1, which indicates the total volumes (ml) of the right and left lungs (VLR and VLL), the percentage of atelectasis (ATE) and volumes (ml) of pleural effusion (EFF).

**Conclusions**

The analysis of HRCT with this semi-automatic reconstruction method can be valid in the study of the lung affected by ARDS, allowing the exploration of the pleural effusion and the parenchyma not affected by effusion and not explorable with TUS. Furthermore, this semi-automatic CT-related technique can be complementary to bedside thoracic ultrasound since this method can visualize with great precision the amount of non-aerated lung parenchyma by quantifying the atelectatic and the effused part. This can help the clinician decide whether to prioritize pleural drainage or atelectatic lung recruitment.

**References**

1. Fjæreide KW, Petersen PL, Mahdi A, Crescioli E, Nielsen FM, Rasmussen BS, et al. Pleural effusion and thoracentesis in ICU patients: A longitudinal observational cross-sectional study. Acta Anaesthesiol Scand. 2023;67(7):943–52.
2. Roberts ME, Rahman NM, Maskell NA, Bibby AC, Blyth KG, Corcoran JP, et al. British Thoracic Society Guideline for pleural disease. Thorax. 2023;78(11):1143–56.

**Table 1:** **(abstract A95).** Data collected after lung volume segmentation

Pt	VLR (mL)	ATE (%)	EFF (mL)	VLL (mL)	ATE (%)	EFF (mL)
1	3158	9,81%	1439	3054	10,40%	1131
2	1154	16,40%	273	1233	7,60%	84
3	1796	8,70%	278	1440	11,30%	469
4	1326	10,40%	248	1588	10,70%	750
5	2025	18,90%	292	1567	3,80%	0
6	1267	31,30%	386	894	31,50%	300
7	4175	15,30%	1492	3554	16,50%	1489
8	1883	10,40%	503	819	14,10%	412
9	2172	8,70%	578	1550	11%	411
10	3178	3%	0	3514	17%	2649
11	1065	18%	83	921	23%	138
12	1414	14,20%	412	1252	21%	576
13	1027	15,40%	276	969	18,70%	253
14	1267	3,30%	0	1140	20%	452
15	1640	9%	604	1587	15%	544
16	2652	7,30%	344	2192	10,10%	162

### A96 Treatment of refractory myasthenia gravis via double-filtration plasmapheresis

#### R. Corallini, R. Grugno, S. Leonardi

##### IRCCS Centro Neurolesi Bonino-Pulejo, Messina, Italy

###### **Correspondence:** R. Corallini

*Journal of Anesthesia, Analgesia and Critical Care 2024*, **4(1):**A96

**Background**

Myasthenia Gravis (MG) is a rare autoimmune disease involving neuromuscular transmission impairment. The most common clinical manifestations include ptosis, diplopia, dysphagia, dysarthria, respiratory difficulty, and motor deficits. Apheresis sessions of Double-Filtration Plasmapheresis (DFPP) are useful for reducing antibody title when pharmacological therapy results ineffective. Additionally, DFPP is safer than conventional Plasma Exchange therapy {1}.

**Case Report**

An 83-year-old patient with type III Myasthenia Gravis, seropositive for Ab AChr and Anti MuSK, was admitted to the neurology department in December 2023 for high-dose corticosteroid treatment. Subsequently, due to inadequate therapeutic response, pharmacological therapy with Eculizumab 900 mg was initiated, without favorable outcome.

Due to worsening clinical conditions, in February 2024, the patient underwent endotracheal intubation (IOT) and was admitted to the intensive care unit of PO Piemonte IRCCS Bonino Pulejo, where, considering the criticality of his neurological condition (soporific state, IOT + VAM, Mingazzini I for a few seconds, no spontaneous movement in the lower limbs), and assessing the blood antibody levels (ARAB RIA method 8.40 nmol/L; Ab anti MuSK RIA method < 0.01 nmol/L), a cycle of three DFPP sessions was prescribed, performed on alternate days in the same department.

Following the first treatment, an immediate clinical improvement was observed: the patient was awake and cooperative, intubated with good respiratory dynamics, and stable hemodynamically. Neurologically, he showed marked hyposthenia of the orbicular muscles of the eye (yet present); he lifted his head from the bed surface for a few centimeters and for a few seconds. He maintained the Mingazzini I position for about 5 s with no spontaneous movements in the lower limbs.

After the second session, a further decrease in antibody title was noted, with progressive improvement in motor deficits: ARAB RIA method 6.80 nmol/L; Ab anti MuSK RIA method < 0.01 nmol/L. The patient was awake and cooperative, undergoing weaning from IOT, following simple commands, lifting his head from the bed surface by about 20°, the right upper limb by 120°, and the left upper limb by 30°.

With the final treatment, a halving of baseline autoantibody levels was observed, ARAB and Ab anti MuSK 4.30 nmol/L; < 0.01 nmol/L respectively (Fig. 1). 3500 ml of plasma were treated for each procedure. There was no need to transfuse the patient with PFC. Albumin levels remained stable in all DFPP sessions.

Informed consent was obtained from all participants.

**Conclusions**

In this clinical case reported of Myasthenia Gravis refractory to pharmacological treatments, the application of DFPP technique has proven to be safe and effective in reducing antibody titers and improving the patient's neurological condition.

**Reference**

1. Altobelli C, Pollastro RM, Marano E, et al. La filtrazione a cascata nel trattamento della miastenia grave: caso clinico [DFPP in myasthenia gravis: case report]. G Ital Nefrol. 2015 Jan Feb;32(1):gin/32.1.5. Italian. PMID: 25774582.

**Fig. 1:**
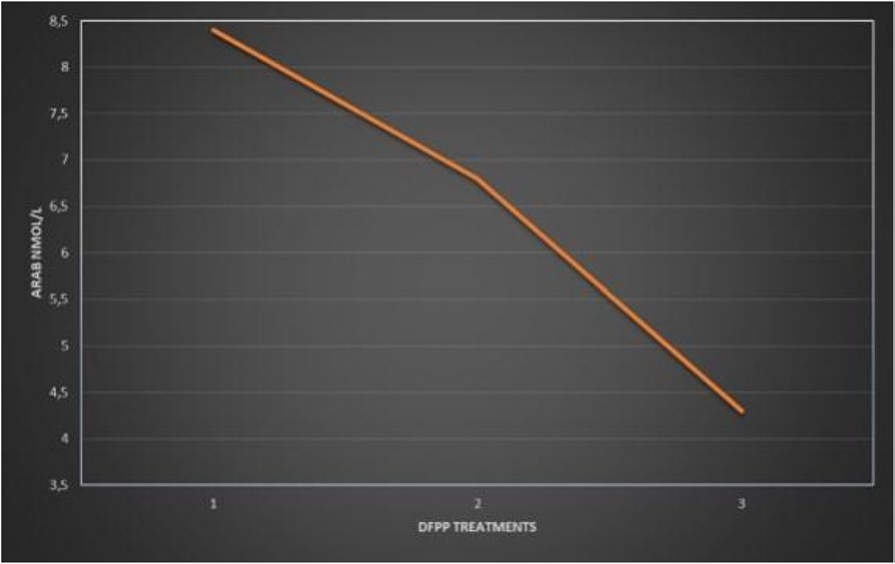
**(abstract A96).** ARAB trend

## Simulation

### A97 Bridging the gap: cross-border collaboration enhances pediatric anesthesia training

#### C. Ebm^1^, S. Brusa^1^, D. Shiffer^2^, B. Dannenberg^3^, R. Furlan^1^

##### ^1^Humanitas University, Milan, Italy; ^2^ICH Humanitas Reseach Hospital, Milan, Italy; ^3^ Stanford University, Stanford, USA

###### **Correspondence:** C. Ebm

*Journal of Anesthesia, Analgesia and Critical Care 2024*, **4(1):**A97

**Introduction**: Anesthesiologists, who may find themselves managing unexpected pediatric or neonatal emergencies, often confront obstacles due to insufficient training, exacerbated by factors like limited equipment access and a scarcity of pediatric anesthesia specialists. Yet, successful management of pediatric emergencies demands expertise, practical experience, and self-assurance – qualities that can be strengthened through specialized training provided by international experts (1). This study seeks to address this pressing need by examining a cross-border pediatric emergency simulation curriculum crafted to enrich the pediatric anesthesia competencies of anesthesiologists working in hospitals with limited resources.

**Methodology:** In a collaborative effort between Humanitas University, Italy, and Stanford University, USA, a year-long study was conducted to assess the impact of an international curriculum on anesthesiologists' preparedness for pediatric emergencies. Led by a team of international expert faculty, the curriculum comprised online modules and onsite simulations. Evaluation methods included focus group discussions and a satisfaction survey. For the satisfaction survey a Likert Scale of 0–5 was used (0 very dissatisfied, 5 very satisfied).

**Results:** Survey responses, representing a 53% participation rate, underscored positive feedback, particularly highlighting the instructors' expertise (rated at 4.9), the practical components of the curriculum (rated at 4.8), and the level of engagement in the course (rated at 4.8). Out of 104 participating anesthesiologists, 55 (52%) completed the online module, with 30 (54%) participating in intensive onsite simulations. Focus group responses confirmed the need for more simulation based exercises for high-risk, low frequency events, such as pediatric emergencies.

**Conclusion:** Despite limited experience in this specialized field, anesthesiologists frequently encounter unexpected pediatric or neonatal emergencies. This cross-border pediatric anesthesia curriculum effectively addresses this training gap, equipping anesthesiologists in low-volume hospitals with confidence needed to manage pediatric cases more effectively. Through global collaboration and innovative training methods, this initiative holds promise for enhancing pediatric emergency care and ultimately improving patient outcomes on a worldwide scale.

**Reference**

1. Everett et al. Ten years of simulation-based training in pediatric anesthesia: The inception, evolution, and dissemination of the Managing Emergencies in Pediatric Anesthesia (MEPA) course. Paediatr. Anaesth. (2017)

## Ambulatory anaesthesia and NORA

### A98 Remimazolam, a new benzodiazepine in the endoscopic field: pilot study

#### V. Noseda, S. Puglisi, S. Stropeni, S.F. Alongi

##### ASST Lecco Ospedale A. Manzoni, Lecco, Italy

###### **Correspondence:** V. Noseda

*Journal of Anesthesia, Analgesia and Critical Care 2024*, **4(1):**A98

**Introduction**

A new ultra-short-acting benzodiazepine called Remimazolam Besylate is indicated, for intravenous use, for procedural sedation in Europe, UK, USA and for anesthesia in Asia.

The molecule has a rapid sedative effect after the first administration and an equally rapid recovery time from the last dose administered. The patient can be awakened more quickly than with other benzodiazepines such as Midazolam, bringing the drug closer to the so-called soft drugs for procedural sedation.

**Scope**

The aim of this pilot study is to observe the efficacy of Remimazolam during endoscopic E.R.C.P. procedures (endoscopic retrograde cholangio-pancreatography).

**Methods**

This observational study was conducted at Manzoni Hospital in Lecco, the data was collected between January and May 2024.

The patients studied were aged over 18 years, ASA class II-III and underwent an ERCP procedure associated or not with ultrasound endoscopy.

The patient, in a supine position, had his vital parameters monitored including EtCO2, oxygen was administered with nasal cannulas and local anesthetic spray into the oral cavity.

Sedation occurred with the administration of Remimazolam in association with opioids according to the technical data sheet. The scheme included Fentanyl 50–100 mcg followed 1–2 min later by Remimazolam with an initial dose of 5 mg if age less than 65 years or 2.5–5 mg if age greater or equal to 65 years, ASA III-IV, weight less than 50 kg.

If necessary, additional doses of Remimazolam (1.25–2.5 mg) were followed at 2 min intervals until the desired level of sedation was achieved (Modified Observer's Alertness/Sedation Scale—MOAA/S = 1–2) for a maximum of 5 additional doses. If the desired level of sedation was not achieved, another sedative medication was considered after 15 min. Informed consent was obtained.

**Results**

Thirty patients undergoing ERCP were studied, including 19 ASA III, 11 ASA II, 12 females and 18 males.

The use of Remimazolam combined with opioids was effective in 15 patients (50%) without the need for additional sedative drugs. In this group, in two cases there was a brief desaturation (SpO2 < 90%) which resolved itself. At the end of the procedure all patients were promptly awakened (MOAA/S = 5) and only in one case was Flumazenil administered.

The duration of the procedure varied from a minimum of 10 min to a maximum of 53 min.

The total dosage of Remimazolam per patient ranged from a minimum of 5 mg to a maximum of 22.5 mg.

The patient's satisfaction upon awakening was excellent, as well as the operator and the anesthetist.

In the remaining cases (50%) Propofol was used as an additional drug to ensure adequate sedation.

**Conclusions**

Remimazolam is safe for achieving adequate sedation for ERCP procedures, quick awakening and excellent satisfaction for both the operator and patients. The drug is easy to handle but to deepen the knowledge of this new soft drug and to compare Remimazolam with other sedation drugs, the study of additional patients is necessary.

### A99 Safety in procedural sedation/analgesia (sap) in digestive endoscopy (ed), an experience of a mutidisciplinary course

#### C. Tani, R. Nardini, M. Luchini, R. Spina

##### SOC Anestesia E Rianimazione Ospedale S.Giuseppe, Empoli, Italy

###### **Correspondence:** C. Tani

*Journal of Anesthesia, Analgesia and Critical Care 2024*, **4(1):**A99

**Introduction:** The increasing of diagnostic and therapeutic possibilities of digestive endoscopic procedures. This combined with an increasingly complex typology of patients due to multiple pathologies, the request of gastroenterologists and the patients themselves to perform procedures in sedoanalgesia for greater comfort but also for greater safety and effectiveness of the procedure, has led at a national and international level to drafting of guidelines and consensus for the execution of procedural sedation/analgesia (SAP) in Digestive Endoscopy (ED). The procedural sedation is the administration of one or more pharmacological agents or facilitate a diagnostic or therapeutic procedure while targeting a state during which airway patency, spontaneous respiration and haemodynamic stability are preserved while alleviating anxiety and pain. From the SIAARTI BPCs for analgo-sedation in digestive endoscopy: Quality and safety of SAP go hand in hand, because adverse events are increasing and mortality in this area is higher than that relating to general anesthesia and anesthesia loco-regional. The main complications during SAP… originate from poor competence, inadequate settings, summary monitoring.

European and national societies have already developed evidence-based and consensus-based guidelines for sedation and monitoring in gastrointestinal endoscopy that give a comprehensive outline of structural requirements, medication options, patient monitoring and discharge, and the role of endoscopy staff.

**Methods:** Incorporating the indications of the European guidelines and the good practices of SIAARTI already mentioned, the SOC of Anesthesia and Resuscitation of the S.Giuseppe hospital in Empoli (FI), in collaboration with the SOC of Gastroenterology and Digestive Endoscopy has established a course of training for SAP in digestive endoscopy carried out in collaboration between anesthetists, resuscitators and gastroenterology doctors. The training was divided into the following themes: Definition of SAP. Principles of sedation. Pre-procedural patient assessment. Basic clinical and instrumental monitoring. Pharmacokinetics and pharmacodynamics of commonly used drugs. Maintenance of airway patency and use of different oxygenation and ventilation techniques. Complications of SAP, their early recognition and initial treatment. Management of the recovery room and post-procedural discharge criteria. Medico-legal aspects and liability profiles.

**Results**: The course involved three hospital units for a total of 49 trained gastroenterology doctors divided into three lessons for 3 h of course each. The staff was subsequently supported in the early stages by personnel expert in SAP in clinical practice.

**Conclusions:** The course strengthened the effectiveness and safety of SAP in digestive endoscopy already active in our hospitals. This has allowed and consolidated the data indicating a reduction in waiting lists for sedation procedures, greater effectiveness of the procedures, greater patient satisfaction, a greater safety profile and a better allocation of available resources and in particular personnel doctor Anesthetist resuscitator. Common training practice standards for all methods of sedation used in endoscopy have been shown to be beneficial in improving clinical practice as well as structural quality. The limitations of the course were a reduced practical part and a lack of involvement of the nursing staff.

### A100 Procedural sedation for individuals with Autism Spectrum Disorder (ASD) in a hospital program for people with disabilities

#### E. Valeri^1^, C. Benassai^1^, M.E. Berni^1^, M. Luchini^1^, G. Vannini^2^, F. Cei^2^, D. Coletta^2^, K. Franchini^3^, R. Tarquini^2^, R. Spina^1^

##### ^1^SOC Anestesia e rianimazione ospedale S.Giuseppe, Empoli, Italy; ^2^SOC Medicina Interna 1 ospedale S.Giuseppe, Empoli, Italy; ^3^Dipartimento infermieristico Ospedale S.Giuseppe, Empoli, Italy

###### **Correspondence:** M. Luchini

*Journal of Anesthesia, Analgesia and Critical Care 2024*, **4(1):**A100

**Introduction:** Autism is a neurodevelopmental disorder that typically appears in childhood, affecting social interaction, communication, characterized by narrow interests, stereotypies, and often cognitive deficits of varying severity. In Italy, around 600,000 individuals have Autism Spectrum Disorder (ASD), with one in 77 children between the ages of 7 and 9 being diagnosed with the disorder. People with ASD, as well as those with intellectual disabilities, often face difficulties accessing healthcare due to their limited ability to cooperate. This, in turn, leads to poorer health states and lower life expectancies. Individuals with autism die 16 years earlier than the general population and die up to 30 years earlier when a severe intellectual disability is associated. To address this issue, in 2000, the S. Carlo Hospital in Milan created DAMA (Disabled Advanced Medical Assistance) pathway, designed to provide dedicated healthcare for individuals with disabilities. In 2017, a regional project called PASS (Percorsi Asistenziali per Soggetti con bisogni Speciali) was launched in Tuscany, to create tailored care pathways for individuals with disabilities.

**Methods:** The DAMA-PASS clinic has been active at the S. Giuseppe hospital in Empoli since 2015, managed by a multidisciplinary team made up of anesthesiologists, internists, facilitator nurses, and specialists who are involved case-by-case. The presence of the anesthesist is essential for those patients for whom it is not possible to obtain collaboration in any way except with sedation, absolutely avoiding physical restraint. After obtaining informed consent from the legal administrator of the patient, procedural sedation may be required for various procedures and even for obtaining venous access for a blood sample in patiens absolutely not collaborative. In this case oral sedation is performed with midazolam in the range of 0.3 mg/kg to 1.3 mg/kg (average 0.7 ± 0.2) mg/kg body weight.

**Results:** We have taken care of 576 patients, including 159 patients with ASD (27.60% of the total patients). 108 patients of the Not ASD group requires any type of sedation. 87 patients of the ASD group requires any type of sedation. Only 34 patients of not ASD group require oral sedation. 61 patients of ASD group require oral sedation.

**Conclusions:** Patients with ASD have difficulty in socialization and communication, which impairs healthcare management. Physical restraint even for a blood draw increases the resistance and fear of healthcare environment, with greater difficulties in subsequent accesses. Our data show that patients with ASD required sedation to a greater extent than the not ASD (54,71% versus 25,96%, p < 0,0001). Furthermore the 38,36% of the patients with ASD need oral sedation in comparison with the 8,17% of not ASD. The greater difficulty of even simple physical contact justifies the use of oral sedation to a much greater extent than the not ASD (p < 0,0001). Autism spectrum disease rapresent a new challenge for the healtcare system to assure the accessibily to medical treatments.

### A101 Remimazolam: a new benzodiazepine for procedural sedation

#### D. Gammaldi

##### Casa di Cura Tortorella, Salerno, Italy

*Journal of Anesthesia, Analgesia and Critical Care 2024*, **4(1):**A101

Remimazolam is a new ultra-fast-acting benzodiazepine, available for procedural sedation in Italy for a few months. It has a high organ-independent elimination clearance and is rapidly metabolised, by non-specific esterases (carboxylesterase 1A—CES 1A), to CNS7054, an inactive metabolite (as opposed to the active metabolite of Midazolam) with a binding affinity of 300 to 400 times reduced at the level of the Aminobutyric acid (GABA) type A gamma receptor. After administration, plasma concentrations of remimazolam decrease predictably and rapidly and, with adequate dosing, there is no prolonged sedative effect.

Used as a sedative for procedural sedation, it produces rapid sedation and lucid recovery with low probability of blood pressure perturbation and respiratory depression [1,2–4].

To induce and maintain procedural sedation in adults, the dosage of Remimazolam must be titrated and individualized to achieve the desired clinical response.

IV administration at a dose of 5 mg followed by additional doses of 2.5 mg with an interval of approximately 2 min between doses is recommended.

The recommended dosage should be reduced in patients with ASA class III/IV.

Possible side effects may be hypoxia, bradycardia, hypotension, nausea and vomiting [3,5,6].

It shows a better safety profile when compared with propofol (under sedation or general anesthesia), with lower incidence of hypotension, less need for treatment for bradycardia and absence of pain during injection [4,8,6].

Not currently available in the pediatric population.

The Author's experience can be traced back to 31 cases (23 F and 8 M) with ASA Status between I and III.

Often in association with Fentanyl, leaving patients breathing spontaneously with optimal maintenance of basic saturation and with a variable heart rate of around 15/20%. Only one case of hypotension, not significant, regressed spontaneously.

In no case was it necessary to resort to the natural antagonist (Flumazenil), having mainly obtained spontaneous awakening (23/31) or on call (8/31).

In conclusion, Remimazolam is an ultra-short-acting benzodiazepine. The main advantages include rapid onset, predictable duration of action, availability of a reversal drug and maintenance of stable hemodynamics, which makes it drug to be used in various clinical practices. However, further clinical studies are essential to comprehensively evaluate its efficacy and safety profile before its widespread application in various clinical settings.

## Cardio-Thoraco-Vascular

### A102 Effects of washing of endovascular catheters during the placement of thoracoabdominal endoprosthesis

#### F. Pica^1^, P. Raimondo^1^, L. Dispoto^2^, S. Lenoci^1^, G. Rubino^1^, M.A. Villani^1^, A. Stripoli^1^, A. Armenise^1^, G. Colantuono^1^, G. Fiore^1^, D. Angiletta^3^, F. Puntillo^2^, S. Grasso^2^

##### ^1^Department of Anesthesia and Intensive Care II, Policlinico di Bari, Bari, Italy; ^2^Department of Precision-Regenerative Medicine and Jonic Area (DiMePRe-J), Section of Anesthesiology and Intensive Care, Bari, Italy; ^3^Department of Vascular Surgery, Bari, Italy

###### **Correspondence:** L. Dispoto

*Journal of Anesthesia, Analgesia and Critical Care 2024*, **4(1):**A102

**Introduction**

The main aspects of the anesthetic management of the thoraco-abdominal endoprosthesis placement procedure concern the prevention of spinal cord injury, the management of intraoperative coagulation and fluid balance.

**Objective**

The objective is to evaluate the impact that the fluids used for washing endovascular catheters performed during the endoprosthesis placement procedure has on respiratory exchanges and coagulation.

**Methods**

The protocol for the management of intraoperative coagulation included an initial bolus of 5000 I.U. of sodium heparin, ACT measurement every 20 min with a target between 150 and 250 s, any additional doses of 30–50 U/Kg of sodium heparin. Respiratory exchanges were assessed via pre and postoperative chest x-ray, LUS and EGA. Flushing of endovascular catheters was monitored via a system that provided fluids at constant pressure.

**Results**

9 patients were enrolled. With an initial bolus of 5000 IU and a second average bolus of 2600 IU of heparin, the range of ACT values remained between 109 and 170 s. The final water balance was positive in all patients (ranging between 300 and 3500 ml). In all patients, the LUS score increased with no statistical difference between the pre- and post-procedure PaO2, while there was a statistical difference between the pre- and post-procedural hemoglobin, hematocrit and PTT values: 5 out of 9 patients were transfused. No complications were reported.

**Conclusions**

The study shows that the positive fluid balance, determined by the liquids introduced intra-arterially, leads to a worsening of the LUS without affecting respiratory exchanges. Washing endovascular catheters allows ACT values to be maintained well below the values reported in the literature (target 200–250 or > 300'').

Informed consent was obtained.

**Fig. 1:**
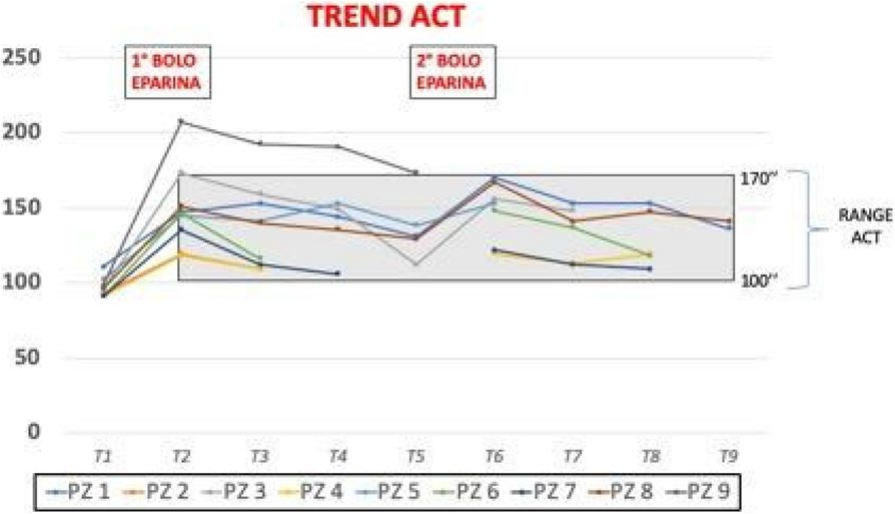
**(abstract A102).** See text for description

### A103 TEG guided administration of bivaluridin in two heparin allergic patients undergoing to triclip and mitraclip implantation: our experience

#### F. Lombardi^1,2^, P. Raimondo^3^, K. Lucarelli^4^, F. Troisi^4^, V. Bellomo^4^, V. Casamassima^4^, M. Grimaldi^4^, V. Pati^3^, M.E. Imbriani^3^, E. Rollo^3^, A. Mascia^3^, F. Puntillo^3^, S. Grasso^3^

##### ^1^U.O.C. Medicina Perioperatoria, Dipartimento di Emergenza e Urgenza, Ospedale Generale Regionale F. Miulli, Acquaviva delle Fonti, Italy; ^2^U.O.C. Cardiologia, Università Cattolica Sacro Cuore, Roma, Italy; ^3^Anesthesia and intensive care unit II—Department of Precision and Regenerative Medicine and Jonica Area, Bari, Italy; ^4^ U.O.C. Cardiologia, Ospedale Generale Regionale F. Miulli, Acquaviva delle Fonti, Italy

###### **Correspondence:** V. Pati

*Journal of Anesthesia, Analgesia and Critical Care 2024*, **4(1):**A103

**Background**

Interventional cardiology procedures involving the right and left sides of systemic circulation require full anticoagulation to prevent thrombus formation on catheters and devices with potential development of embolic complications. Heparin represent the first choise for anticoagulation in the Cath Lab, but in patients with its contraindication there is bivalirudin, a direct thrombin inhibitor with short half-life, used without a defined drug management protocol. Activate Clotting Time (ACT) can be used to monitor bivalirudin administration, but it is not routinely recommended.

The aim of this case report is to define and evaluate the results of ACT/Thromboelastography(TEG) guided administration of bivalirudin during mitral and tricuspid Transcatheter Edge-to-Edge Repair (TEER).

**Case report**

We enrolled two patients underwent to percutaneous correction of valve regurgitation: an 87-year-old lady with massive functional tricuspid regurgitation, pulmonary hypertension, ASA III, NYHA III (A) and a 79-year-old lady with severe functional mitral regurgitation, ASA III, NYHA II (B), both allergic to sodium heparin and to low molecular weight heparins. The procedures were successfully completed using bivalirudin anticoagulation.

The bivalirudin infusion rate during the Triclip and Mitraclip (Abbott, Chicago, IL) procedures was modulated according to the value of the ACT following a team-agreed protocol with targed ACT > 250 s and to the kaolin thromboelastograph reaction time (KTEG R-time) < 28 min (or 3–4 times baseline value)^1^.

For both patients- according to body weight and kidney function- the initial bolus of bivalirudin 0.75 mg/kg has been followed by continuous infusion of 1.75 mg/kg/hour modulated according to the ACT value (Fig. 1), double-checked at closed intervals and TEG before, during and in the hours following the procedure; bivalirudin was also diluted in the continuous washing solutions of the transcatheter equipment to increase the margin of safety. The KTEG R-time value (Fig. 2) showed the greatest change in response to bivalirudin bolus and infusion with smaller reductions in the time after the R point to reach a certain level of clot strength or angle of the rate of clot formation.

**Conclusions**

This experience, although with several limitations, supports the utility of these tests to guide the administration of bivalirudin as an alternative to heparin. Before, during and after percutaneous intervention, ACT and TEG guided the coagulation state of the patient, monitoring the effect of bivalirudin and the function of the coagulation system components, to modulate and correct the target dose, to avoid hemorrhagic complication, until the end of the drug effect.

Informed consent was obtained.

**Fig. 1:**
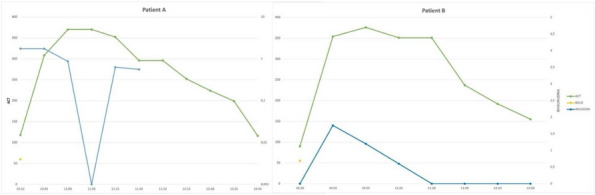
**(abstract A103).** Trend of ACT, Bolus and Continuous Infusion Patient A and B

**Fig. 2:**
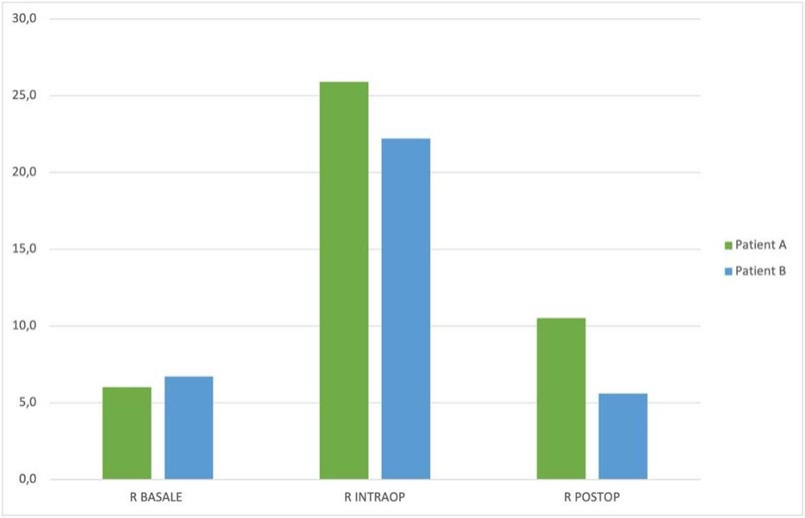
**(abstract A103).** Reaction Time Monitoring Patient A and B

### A104 Anesthesia for awake video-assisted thoracic surgery: a case report

#### A. Di Fuccia, G. Lauro, M. Sellini, V. Maffei, M.C. Mazza, G. Scognamiglio

##### Department of Elective Surgery, Postoperative Intensive Care Unit and Hyperbaric Oxygen Therapy, A.O.R.N. A. Cardarelli, Naples, Italy

###### **Correspondence:** A. Di Fuccia

*Journal of Anesthesia, Analgesia and Critical Care 2024*, **4(1):**A104

**Background**

Traditionally, video-assisted thoracic surgery (VATS) is performed under general anesthesia with selective ventilation and endotracheal intubation. Although little data exists on awake video-assisted thoracic surgery (VATS), it has been reported to be safe and feasible for patients with various thoracic diseases, including those who have respiratory dysfunction. Adequate anesthesia and analgesia obtained from thoracic epidural anesthetic (TEA) or echo-guided blocks allow VATS to be performed in awake patients in spontaneous ventilation. Patients who undergo awake VATS may also benefit from the efficient contraction of the dependent hemidiaphragm and preserved hypoxic pulmonary vasoconstriction during surgically-induced pneumothorax. Potential hazards include paradoxical respiration and mediastinum shift after surgery induced pneumothorax, which may cause progressive hypoxia, hypercapnia and hypotension. Preliminary results from early case studies have indicated certain benefits, including greater patient satisfaction, less nursing care, less sore throat, earlier resumption of oral intake, lower rate of morbidity, reduced perioperative pain, reduced cost, and shorter hospital stay. (1).

**Case report**

A 76-year-old patient, due to a chest CT finding of a nodular formation with irregular margins in the right upper lobe, had to undergo lobectomy following an immediate positive evaluation for tumor, but the spirometry data, according to American guidelines, did not allow such eradication, so he underwent atypical resection. The patient, with positive anamnesis for ischemic heart disease, arterial hypertension, type II diabetes mellitus, chronic renal failure, pulmonary hypertension, but above all for COPD in home oxygen therapy, was subjected to pulmonary resection in an awake state. The patient sedated with Midazolam 5 mg and Fentanest 200 gamma, after positioning in lateral decubitus, the ESP block at the T6-T7 level and block of the pectoserratus was carried out, both with a mixture of local anesthetic of Ropivacaine 0.375% 20 ml. During the operation, the patient was breathing spontaneously with O2 support 5 l/min via nasal cannula with an average peripheral saturation of 92%, the only two episodes of desaturation below 85% were during the iatrogenic pneumothorax and during the lung resection. When the saturation level became stable, 50 mg of Propofol bolus was used to increase sedation at that moment. The postoperative period was uncomplicated and the patient was discharged on the third postoperative day with a saturation of 92% on room air. Patient signed written informed consent.

**Conclusion**

The non-intubated procedures try to minimize the adverse effects of tracheal intubation and general anesthesia, the awake approach represents another step forward in the minimally invasive strategies of treatment. Anesthesiologists should be acquainted with the procedure to be performed.

**Reference**

1. Kao MC, Lan CH, Huang CJ. Anesthesia for awake video-assisted thoracic surgery. Acta Anaesthesiol Taiwan. 2012 Sep;50(3):126-30. 10.1016/j.aat.2012.08.007. Epub 2012 Sep 8. PMID: 23026172.

### A105 Management of a refractory thrombosis after mechanical mitral valve replacement: a case report

#### C. Matellon^1^, A. Capone^2^, S. Cattaneo^1^

##### ^1^Asst Spedali Civili Di Brescia, Brescia, Italy; ^2^Universita' Degli Studi Di Brescia, Brescia, Italy

###### **Correspondence:** C. Matellon

*Journal of Anesthesia, Analgesia and Critical Care 2024*, **4(1):**A105

**Background**

The annual rate of prosthetic valve thrombosis after Mechanical Hearth Valve (MHV) implantation ranges from 0.1% to 5.7%, with higher rates observed with specific valve types, in the early perioperative period, with MHVs implanted in the mitral and tricuspid position, and in association with subtherapeutic anticoagulation. [1–3].

**Case Report**

A 65-year-old female suffering from degenerative mitral regurgitation underwent MHV implantation. On the 1st postoperative day (POD), low-molecular-weight heparin (LMWH) was started to prevent thrombosis. On the 13th (POD) mediastinitis occurred and a surgical revision was necessary. LMWH had been stopped less than 24 h before and it was not administered immediately after to avoid post-surgical bleeding. The patient was admitted to the Post-Cardiosurgical Intensive Care Unit, where she rapidly became awake, eupneic after the extubation and hemodynamically stable. A few hours later, she developed dyspnea, acute cardiac failure with pulmonary oedema, anuria and fever. She was re-intubated and a trans-esophageal echocardiography showed a big mobile mass attached to the prosthetic valve (Fig. 1–2). Unfractionated heparin was administered (2500 IU, then 500 IU/h); then epinephrine and norepinephrine infusion started. To treat possible endocarditis with septic shock, also arginine-vasopressin and antibiotic therapy were started.

48 h later, echocardiography did not change, so recombinant tissue plasminogen activator was administered, but the mass did not reduce.

4 days later, respiratory and cardiocirculatory situations improved and a cerebral CT scan showed many little ischemic injuries with minimal hemorrhagic evolution in the right temporal lobe and basal nuclei.

After a collegial review with cardio-surgeons and neurologists, a new CT scan was performed and a new frontal ischemic injury was found. Considering that the cardiorespiratory situation was stable, but neurological damage was evolving, the patient, in 21st POD underwent MHV removal and mitral biological valve implantation: a big thrombotic mass was found attached to the major part of MHV. Afterwards, the patient had a good recovery without any neurological disability, except for a minimal hyposthenia at the left superior limb.

Haematological tests performed in the 48th POD revealed anti-B2-GPI antibodies positivity; nevertheless, the patient had never had any thrombotic events before.

**Conclusions**

Anti-coagulation therapy is a fundamental part of post-operative treatment after mitral valve replacement; the approach to refractory intra-cardiac thrombosis should be individualized and multidisciplinary, especially in case of cerebral embolism and risk of major bleeding.

**Informed consent**

Written informed consent to publication was obtained from the patient.

**References**

1. Lin SS et al. Prediction of thrombus-related mechanical prosthetic valve dysfunction using transesophageal echocardiography, The American Journal of Cardiology 2000; 86(10):1097–1101.
2. Dangas GD et al. Prosthetic Heart Valve Thrombosis, Journal of the American College of Cardiology 2016; 68(24):2670–2689.
3. Falk, V et al. 2017 ESC/EACTS Guidelines for the management of valvular heart disease, European Journal of Cardio-Thoracic Surgery 2017;52(4):616–664.

**Fig. 1:**
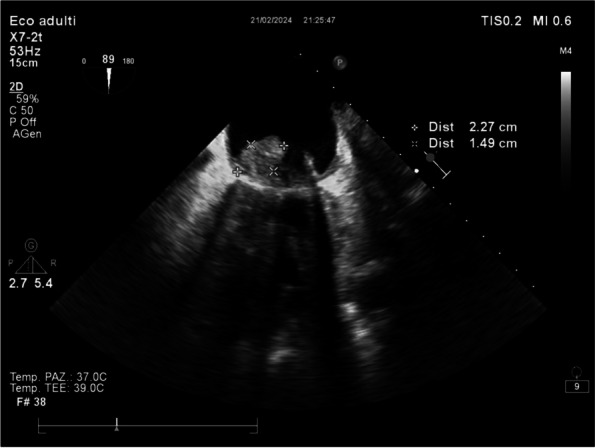
**(abstract A105).** Left atrial thrombus visualized in transesophageal echocardiogram (TEE) in midesophageal mitral commissural view

**Fig. 2:**
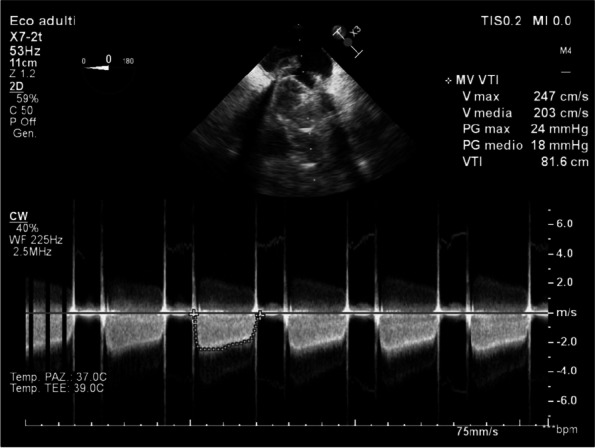
**(abstract A105).** Continuous wave Doppler across the mitral valve with dense mitral stenosis envelope. Velocity time integral mean gradient was 18 mmHg

### A106 Anesthesiological expertise and outcome after cea procedures: a monocentric retrospective analysis

#### L.A. Legnani^1^, L. Guzzetti^1^, G. Piffaretti^2^, M. Tozzi^2^, N. Rivolta^2^, M. Franchin^2^, M.C. Cervarolo^2^, G. Selmo^1^, F. Baggio^1^, C. Novazzi^1^, A. Bacuzzi^1^

##### ^1^Anesthesia Unit, Emergency and Urgency Department, Circolo University Hospital, Asst Settelaghi, Varese, Italy; ^2^Vascular Surgery Unit, Department of Surgery and Morphological Sciences, University of Insubria, Asst Settelaghi, Varese, Italy

###### **Correspondence:** L.A. Legnani

*Journal of Anesthesia, Analgesia and Critical Care 2024*, **4(1):**A106

**Background**

Intraoperative anesthesiological management is a key part of high-quality surgical care.

We sought to examine the association between anesthesiologist expertise and postoperative neurological outcome in patients undergoing CEA, assuming that anesthesiologist expertise could be considered proportional to procedures volume.

**Materials and methods**

A retrospective observational cohort study was conducted at our University Hospital in Varese.

248 patients undergoing CEA from 2018 to 2021 were recruited, 228 patients were eligible.

Preoperative clinical presentation, intraoperative data, clinical situation at discharge and at 6/12-months postoperative follow-up have been researched.

Primary endpoint: finding an association between postoperative neurological adverse events and anesthesiologist volume.

It has been considered post-operative neurological adverse event the presence of neurological symptoms that appeared post-operatively or in the 30 days following surgery and not present at the pre-operative neurological evaluation.

Postoperative neurological adverse events were classified using the NIHSS scale.

The degree of residual disability was classified using the modified Rankin scale (mRS).

Anesthesiologists were divided into two groups – senior and junior—depending on the interquartile range of CEA procedures volume of each anesthesiologist during the study period.

**Results**

Of the 228 patients, 151 (66.2%) were men, 77 (33.8%) women. The most represented age group for both genders was 70–79 years old.

137 patients were asymptomatic, 91 symptomatic.

The percentages of symptomatic and asymptomatic patients, types of anesthesia, and mean clamping times were similar between males and females, with no gender differences.

Postoperative neurological complications were detected in 10 cases.

CEA procedures were conducted by 32 anesthesiologists and 10 surgeons.

Anesthesiologists were divided into two groups, senior and junior, based on the interquartile range of procedures supported (median = 5), selected as cutoff point.

29 procedures (12.7%) were conducted by junior anesthesiologists, 199 (87.3%) by senior.

In the 29 procedures conducted by junior anesthesiologists, neurological complications occurred in 3 cases (10.3%), all symptomatic. In the 199 procedures conducted by senior, neurological complications occurred in 7 cases (3.5%), 4 of which symptomatic.

**Discussion**

The volume-outcome association has been widely demonstrated for surgeons, while limited data are available about the same association for anesthesiologists.

From our data analysis, it was not possible to demonstrate a statistically significant difference between neurological complications in the group of operations conducted by a senior or junior anesthesiologist (p > 0.05).

However, the percentage of complications in the group of anesthesiologists involved in the study shows a decreasing trend as the volume of operations increases (Fig. 1).

**Conclusions**

It is difficult to establish the relative weight of each individual factor (patient, surgeon, anesthesiologist) on the final outcome, and several variables were not subject to analysis. However, to analyse the relationship between anesthesiologist expertise and patient outcome can provide useful informations for improving anesthesiological management and therefore the quality of care.

Informed consent was obtained.

**Fig. 1:**
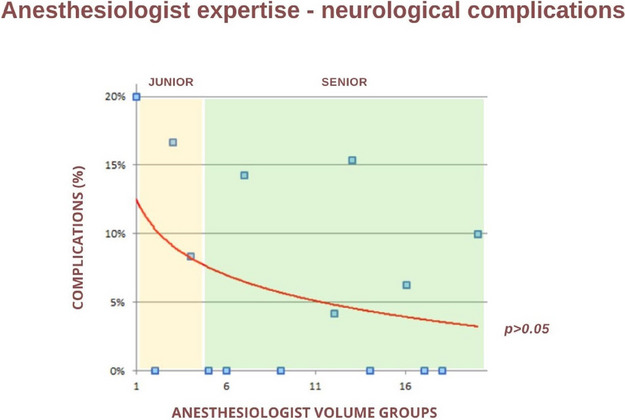
**(abstract A106).** See text for description

### A107 Landiolol in critical ill cardiac patients: preliminary experience

#### V. Caruso^1^, G. Giovenale^1^, J. Cattani^2^, N. Gallina^2^, R. Bosso^1^, G. Moro^1^, V. Daffara^1^, T. Esposito^1^, M. Defilippi^1^, P. Federico^1^

##### ^1^Cardiothoracic Anesthesia and Intensive Care, Azienda Ospedaliero-Universitaria Maggiore della carità, Novara, Italy; ^2^University of Eastern Piedmont, Department of Translational Medicine, Novara, Italy

###### **Correspondence:** J. Cattani

*Journal of Anesthesia, Analgesia and Critical Care 2024*, **4(1):**A107

**Introduction:** Tachyarrhythmia are major source of mortality and morbidity in critical ill patients [1]. Landiolol is an ultra-short acting, I.V. beta-1-blocker, that shares similarities to esmolol, such as the metabolism pathway; however, landiolol presents faster pharmacokinetics, acts with higher ‘potency’, and enjoys higher cardioselectivity. Furthermore, unlike esmolol, landiolol has limited impact on blood pressure which proves to reduce the heart rate without undesired drop of arterial blood pressure [2]. The aim of our study is to evaluate the effectiveness of landiolol in a population of critically ill patients.

**Materials and methods:** this is a retrospective observational study conducted in the cardiovascular ICU (CVICU) of Azienda Ospedaliera Universitaria “Ospedale Maggiore della Carità”, Novara. Data were collected among patients admitted to the CVICU between October 2023 and April 2024. The main inclusion criteria was: patients aged > 18 years who received landiolol. All data were recorded in an institutional database, informed consent having been obtained. Quantitative data are presented as means (standard deviation), and qualitative data are presented as frequencies and percentages. Comparison between repeated measures was performed using Wilcoxon signed-rank test. A p value < 0.05 was considered statistically significant. The primary endpoint was efficacy of landiolol in reducing heart rate (HR) more than 10% of the baseline (HR T0).

**Results:** 16 patients received landiolol, one was excluded because of rapid hemodynamic deterioration needing emergent cardiac surgery. The study population is composed by 15 patients. The mean age was 66 years (SD ± 16). Other characteristics are shown in Table 1. The mean landiolol dose was 5.57 mcg/kg/min (SD ± 6.87), with a mean therapy duration of 28,5 h (SD ± 18.95).

60% of patients reached the primary endpoint within the first 2 h (HR T0 107 bpm ± 25 and HR T2 93 bpm ± 17, p value 0.01), and 40% within 24 h (HR T24h 87 bpm ± 15, p value 0.03). VIS score (T0 5.1 ± 8.4 and T24 3.7 ± 6.7, p value 0.7), MAP (T0 79 mmHg ± 13 and T24 80 mmHg ± 13, p value 1), and lactates (T0 1.6 mmol/L ± 1.1 and T24 1.3 ± 1.2, p value 0.2) didn’t differ significantly between the start of landiolol and the first 24 h. Only one patient needed the interruption of landiolol infusion because of hypotension. Bridging with oral beta blockers was performed in 73% of our patients.

**Conclusion:** based on these preliminary results, we confirm the effectiveness of landiolol in reducing heart rate. Furthermore, it doesn’t seem to affect hemodynamics and need for vasopressors. It may also be used for bridging with long term therapies with oral beta blockers.

**References**

1. L. C. Herzog L, Arrhythmias accompanying cardiac surgery. In: Lynch C, editor. Clinical Cardiac Electrophysiology. 3rd edition. Philadelphia, Pa, USA: JB Lippincott; 1994. p. p. 231.
2. G. Plosker, Landiolol: a review of its use in intraoperative and postoperative tachyarrhythmias. Drugs 2013;73:959–977.

**Table 1:** **(abstract A107).** Characteristics of study population.

Number of analysed patient		15 (100%)
Sex	Female	5 (33%)
	Male	10 (67%)
Diagnosis	After cardiac surgery	10 (67%)
	AHF	1 (7%)
	AMI	2 (13%)
	Other	1 (7%)
	Septic shock	1 (7%)
Arrhythmia	AF	8 ( 53%)
	AFL	1 ( 7%)
	Other	6 ( 40%)
Primary choice	NO	10 (67%)
	YES	5 (33%)
Concomitant antiarrhythmic agents	Amiodarone	9 ( 60%)
	None	5 ( 33%)
	Other	1 (7%)

### A108 Clevidipine in management of arterial hypertension during VV-ECMO weaning—a case report

#### M.P.M. Follino^1^, A. Bucci^2^, A. Di Rienzo^2^, S.M. Maggiore^1,3^

##### ^1^Departmen of Anesthesiology, Critical Care Medicine and Emergency, SS. Annunziata Hospital, Chieti, Italy; ^2^Department of Cardiac Anesthesia and Cardiac Intensive Care Unit, SS. Annunziata Hospital, Chieti, Italy; ^3^Department of Innovative Technologies in Medicine & Dentistry, Section of Anesthesia and Intensive Care, SS. Annunziata, Chieti, Italy

###### **Correspondence:** M.P.M. Follino

*Journal of Anesthesia, Analgesia and Critical Care 2024*, **4(1):**A108

Clevidipine (Cleviprex®) is a Ca2 + antagonist administered intravenously and used to reduce blood pressure when usual treatment is not effective. Overall, the pharmacological profile and clinical efficacy of clevidipine make it a valuable tool in the management of arterial hypertension, particularly in the perioperative management of adult patients undergoing cardiac surgery.

In this case report, we present a case in which clevidipine was used in the management of an acute episode of arterial hypertension poorly controlled by common therapies.

The case concerns a 51-year-old male patient hospitalized for STEMI due to severe residual coronary artery disease of LAD and Cx post primary PCI-DES on RAC.

Medical history: arterial hypertension, dyslipidemia, grade I obesity, DM2, smoking and family history of CAD. After a three-vessel CABG surgery (LIMA-LAD; GSV-DB; GSV-IB) and discharged by the Cardiac ICU the next day, he returned to Cardiac ICU after three days post-surgery for respiratory distress on a septic basis due to HAP and hypertension nonresponding to drugs. After a failed NIV trial, orotracheal intubation and mechanical ventilation were performed. For worsening hypoxemia (PaO2/FiO2 < 100), VV-ECMO was undertaken. Weaning from VV-ECMO performed on the seventh day of treatment with PaO2/FiO2 = 215.

Failure to complete respiratory weaning due to refractory arterial hypertension, despite analgosedation and pharmacological treatment with clonidine, doxazosin, nitroglycerin, nifedipine, labetalol (in the suspicion of pheochromocytoma), diuretics and β-blockers at maximum dosage (Fig. no.1 and Table no. 1). Clevipidine was then started in continuous infusion (0.5 mg/ml) with an initial dosage of 2 mg/h up to 8 mg/h (titration as per the technical data sheet) combined with continuous infusion of labetalol at 75 mg/h and clonidine 150 μg × 3/day i.v. with clear improvement in hemodynamics, side effects have not been reported (Fig. no. 2 and Table no. 1). Excluding other causes of secondary hypertension (CT angiography of renal arteries and urinary dosage of catecholamines), the patient had a renal arteries sympathectomy with radiofrequency. The infusion of clevipidine and labetalol continued for a further 7 days until re-conversion of oral therapy with nifedipine, atenolol and doxazosin. Once the blood pressure was well controlled, weaning was gradually carried out from analgosedation and mechanical ventilation.

In conclusion, our experience confirms what is already present in the literature: clevipidine is indicated in the control of arterial hypertension in the perioperative period, especially in cardiac surgery patients, in this case used beyond the time range without any problems.

For this abstract, informed consent was acquired.

**Fig. 1:**
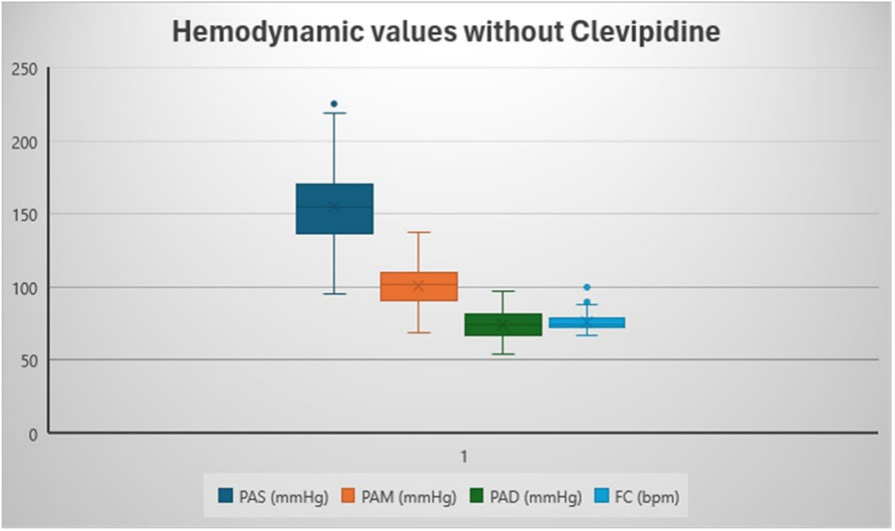
**(abstract A108).** See text for description

**Fig. 2:**
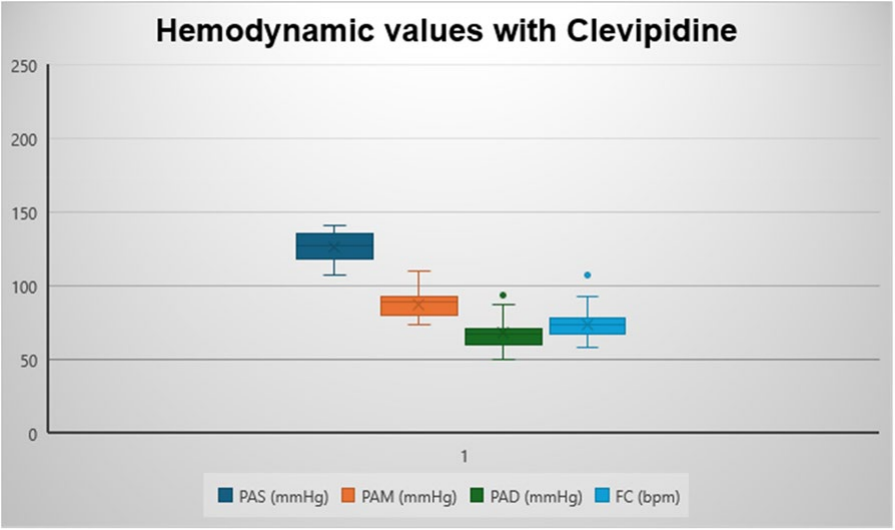
**(abstract A108).** See text for description

**Table 1:** **(abstract A108).** See text for description

Table no. 1		**Hemodynamic values without Clevipidine infusion—Median (IQR)**	**Hemodynamic values with Clevipidine infusion—Median (IQR)**
	**SAP (mmHg)**	155 (137–170)	127 (119–136)
	**MAP (mmHg)**	102 (91–110)	89 (81–93)
	**DAP (mmHg)**	74 (68–81)	67 (61–71)
	**HR (bpm)**	74 (72–79)	74 (68–78)

### A109 Airway bleeding in aortic dissection-related coagulopathy

#### G. Cosenza^1^, L.M. Bottazzo^1^, G. Sepolvere^2^, F. Di Zazzo^2^, M. della Valle^2^, L. Merola^2^, S. de Sarno^1^, F. Coppolino^1^, V. Pota^1^, P. Sansone^1^, M.B. Passavanti^1^, M.C. Pace^1^

##### ^1^Department of Woman, Child, General and Specialistic Surgery, University of Campania L. Vanvitelli, Napoli, Italy; ^2^Department of Anesthesia and Cardiac Surgery Intensive Care Unit, San Michele Hospital, Caserta, Italy

###### **Correspondence:** G. Cosenza

*Journal of Anesthesia, Analgesia and Critical Care 2024*, **4(1):**A109

**Background**

Hemorrhage is a common complication in patients with Stanford-style type A aortic dissection (ATAAD).

It is due both to disease-associated factors such as damage to the aortic wall with tissue factor release with consumption of coagulation factors, fibrinolysis and platelet dysfunction and to surgery-associated factors such as blood contact with the walls of the extracorporeal circulation and aspiration, which cause platelet dysfunction, hypothermia, dilution and consumption of factors and persistence of circulating heparin (1).

The perioperative diagnosis of these abnormalities consists of both conventional examinations (INR, PT, PTT, fibrinogenemia) and viscoelastic tests (VET), aggregometry and DOAC testing.

**Case Report**

A 50-year-old male patient undergoing surgery for ATAAD is admitted to the Intensive Care Unit. Informed consent to publish had been obtained.

On the third day of hospitalization, he presented respiratory failure with desaturation and worsening of blood gas parameters.

The first diagnosis hypothesized was that of lung injury associated with massive transfusion (TRALI), due to the high administration of these in the operating room due to the hemorrhagic shock that the patient presented at admission.

A bronchoscopy is performed which shows profuse hemorrhage of the upper airways and the presence of a voluminous clot at the level of the left main bronchus, which completely occluded the bronchial lumen (Fig. 1).

It is therefore necessary to perform a VET that demonstrates a deficiency of coagulation factors and fibrinogen, compatible with a consumption coagulopathy.

In accordance with guidelines on patient blood management (PBM) (2), fresh frozen plasma is administered at a dosage of 15 ml/kg, until the coagulation factor deficiency is corrected, and fibrinogen concentrate at a dosage of 30 mg/kg in order to obtain a fibrinogenemia > 1.5 g/dl.

After correcting the abnormalities of hemostasis and clearing the airways of clots, we proceed to gradual weaning by mechanical ventilation.

**Conclusions**

The use of VET and PBM guidelines can be crucial in monitoring and correcting hemostasis abnormalities, while reducing the risk of bleeding complications and improving clinical outcome.

This case demonstrates the importance of considering upper respiratory tract hemorrhage as a possible cause of respiratory distress and delayed weaning in patients with ATAAD.

Rapid identification and effective treatment of respiratory complications, such as bronchoscopy to remove the obstructive clot, are essential.

Informed consent was acquired.

**References**

1. Erdoes G, Ahmed A, Kurz SD, Gerber D, Bolliger D. Perioperative hemostatic management of patients with type A aortic dissection. Front Cardiovasc Med. 2023 Nov 20;10:1,294,505. 10.3389/fcvm.2023.1294505. PMID: 38,054,097; PMCID: PMC10694357.
2. Mitra B, Jorgensen M, Reade MC, Keegan A, Holley A, Farmer S, Harvey N, Winearls J, Parr M, French CJ; Clinical and Consumer Reference group for the update of Patient Blood Management Guidelines (Module 1: Critical Bleeding/Massive Transfusion). Patient blood management guideline for adults with critical bleeding. Med J Aust. 2024 Mar 4; 220(4):211–216. 10.5694/mja2.52212. Epub 2024 Jan 28. PMID: 38,282,333.

**Fig. 1:**
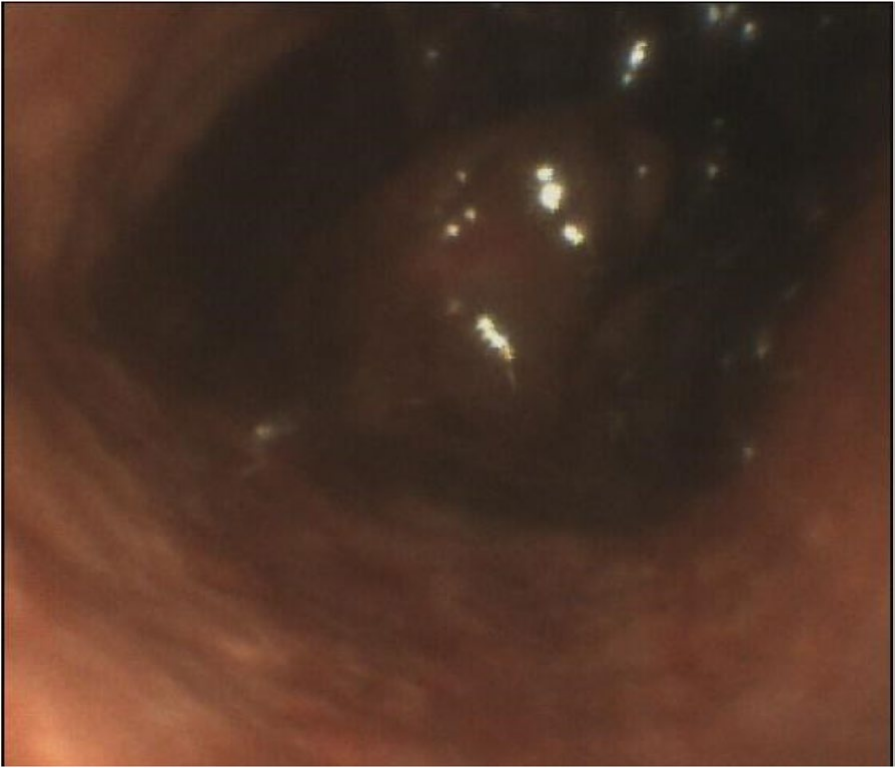
**(abstract A109).** Clot occluding the left main bronchus

### A110 Anesthetic management of an elderly patient with left hemithorax chondrosarcoma: a case report

#### G. Torregiani^2^, V. Ceccarelli^1^, G. Tola^2^, G. Fedele^2^, S. Orlando^1^, P. Gaglioti^1^, G. Gazzè^1^, P. Papa^1^, M. Covotta^2^, E. Forastiere^2^, C. Coccia^2^

##### ^1^Sapienza University of Rome, Rome, Italy; ^2^IRCCS Regina Elena National Cancer Institute, Rome, Italy

###### **Correspondence:** V. Ceccarelli

*Journal of Anesthesia, Analgesia and Critical Care 2024*, **4(1):**A110

**Background**

The term Chondrosarcoma (CS) encloses a group of heterogeneous primary malignant cancers of bone, characterized by the formation of cartilaginous neoplastic tissue [1]. CS are chemotherapy and radiation resistant, so surgery is the first choice.

**Case Report**

A 84-years-old male patient (ASA 3) with left hemithorax CS, grade 2, admitted to the Thoracic Surgery unit of the IRCCS -Regina Elena National Cancer Institute, Rome, undergoing 'En Bloc' resection of the tumor with sternal manubrium, proximal third of the sternal body, right sternoclavicular joint and chondro-sternal joints of I-II-III rib, middle proximal third of the clavicle, upper left lung lobe, soft tissue of chest wall and neck (Fig. 1 a, b). Reconstruction was performed with bipedicled deep inferior epigastric perforator (DIEP) flap (Fig. 1c). Patient was intubated with bi-lumen endotracheal tube to allow selective exclusion of the left lung during surgery. Cerebral oximetry was monitored to avoid a desaturation greater than 20% of the basal during clamping of neck vessels. The advanced hemodynamic monitoring was placed to evaluate the cardiac index (CI) trend and to target the fluid therapy. The cerebral and hemodynamic parameters are summarized in Table 1. Arterial blood gases (ABGs) analyses at regular intervals were performed (Table 2). We observed a significant reduction of CI and cerebral oximetry so a continuous infusion of norepinephrine at low doses (0.08 mcg/kg/min) was employed. No significant respiratory changes were observed. A total of 13,000 ml of crystalloid solutions were administered, 2000 ml of saline and 11,000 ml of balanced solutions. Urine output was 3750 ml. The blood loss was 1500 ml. Two packed of concentrated blood cells were transfused during surgery. At the end of the surgery, the patient was transferred sedated and intubated to the Intensive Care Unit (ICU). Patient was extubated the next day and High Flow Nasal Cannula was placed (50L/min, 30%). The postoperative course in ICU was regular and the patient was transferred to the Thoracic surgery ward 6 days later.

**Conclusion**

The anaesthetic management of patients undergoing these highly destructive surgeries is a challenge not only for the high risks associated with the surgery itself but also for the difficulty of adapting the anesthetic choices to the needs of four different surgical teams (i.e. thoracic, orthopedic, vascular and plastic).

Informed consent to publish had been obtained.

**Reference**

1. Chow WA. Chondrosarcoma: biology, genetics, and epigenetics. F1000Res. 2018;7:F1000.

**Fig. 1:**
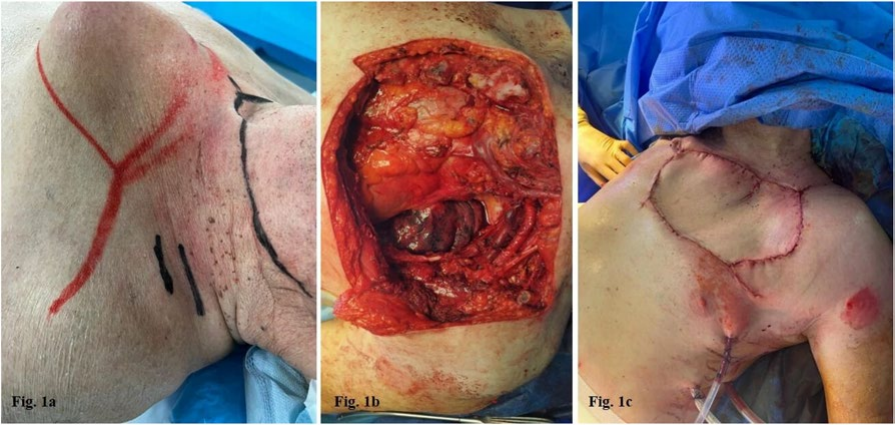
**(abstract A110).** En bloc resection of CS and reconstruction with DIEP flap

**Table 1:** **(abstract A110).** Cerebral and hemodynamic parameters at regular intervals after induction

	**After induction**	**2 h**	**4 h**	**6 h**	**8 h**	**10 h**	**12 h**	**14 h**
Cerebral Oximetry	L 82 R 80	L 80 R 82	L 77 R 77	L 69 R 69	L 78 R 75	L 75 R 70	L 74 R 70	L 78 R 70
C.I. (L/min/m2)	2.6	3.6	2.6	3.1	2.6	2.5	2.4	3.1
FC (bpm)	90	90	90	103	101	101	103	98
MAP (mmHg)	105	80	77	78	83	82	87	77
SpO2 (%)	99	99	98	100	100	100	100	100

**Table 2:** **(abstract A110).** ABGs parameters at regular intervals after induction

	**After induction**	**2 h**	**4 h**	**6 h**	**8 h**	**10 h**	**12 h**	**14 h**
pH	7.35	7.31	7.36	7.30	7.33	7.39	7.41	7.41
pCO2 (mmHg)	46	48	40	44	42	42	42	43
pO2 (mmHg)	113	98	95	133	212	201	216	217
Lac (mmol/L)	0.8	1.5	2.1	3.9	3.3	3.1	3.3	3.7
Hb (g/dL)	14	13	12.4	11.8	11.6	12.9	12.7	11.3
HCO3- (mmol/L)	25.4	24.2	22.6	21.6	22.1	25.4	26.6	27.3
BE (mmol/L)	-0.6	-2.5	-2.7	-4.8	-3.7	0.3	1.7	2.2

## Metabolism, nutrition and renal therapies

### A111 Ammonium (NH4 +): A new biomarker of renal tubular function? Investigation on healthy volunteers and renal transplanted patients

#### L.M. Titherington^1^, V. Giglioni^1^, I. Mattei^1^, Z. Ricci^2^, F. Peyronel^3^, G. Villa^1^, G. Lascialfari^1^, L. Ricci^1^, S. Romagnoli^1^

##### ^1^Department of Anesthesia and Critical Care Azienda Ospedaliero-Universitaria Careggi, University of Florence, Florence, Italy; ^2^Anesthesia and Critical Care Meyer Children Hospital IRCCS, University of Florence, Florence, Italy; ^3^Nephrology and Dialysis Unit, Meyer Children University Hospital—IRCCS, Florence, Italy

###### **Correspondence:** L.M. Titherington

*Journal of Anesthesia, Analgesia and Critical Care 2024*, **4(1):**A111

**Background**

Renal function assessment conventionally relies on serum creatinine measurement to estimate glomerular filtration rate (eGFR), usually overlooking tubular function. Despite its widespread use, urine output (UO) as an indicator of renal insufficiency is limited by various clinical variables. One of the main roles of renal tubules is acid–base regulation, which is achieved through reabsorption of filtered HCO3-, excretion of titratable acid, and synthesis and excretion of ammonium (NH4 +), eventually regenerating bicarbonate. NH4 + is mainly produced in the proximal tubule via glutamine catabolism and its net secretion into the luminal fluid is enhanced in metabolic acidosis^1^. Therefore, measuring NH4 + urinary concentration can provide important information on the renal tubules’ ability compensate in states of metabolic acidosis and provide clinical indications of tubular function. However, despite its potential significance, urinary NH4 + concentration is not routinely measured in clinical laboratories^2^.

**Materials and Methods**

This study aimed to directly measure urinary NH4 + concentration using the point-of-care or semi-continuous analyser Kidney INstant monitorinG (KING®, Kures, Milan, Italy) and compare values in 34 healthy volunteers and 16 renal transplant recipients. The urinary NH4 + concentration in the renal transplant recipient patients was evaluated 24 h after transplantation, a period in which renal function is most likely to be impaired due to acute tubular necrosis following ischemia–reperfusion injury.

**Results**

Healthy volunteers exhibited a median urinary NH4 + concentration of 19.15 mEq/L (IQR 16.08), whereas renal transplant recipients on day 1 had a median concentration of 4.98 mEq/L (IQR 4.01), with p < 0.01, as indicated in Table 1. Preliminary analysis comparing day 1 and 10 post-transplant also shows an increase in median NH4 + concentration, from 4.98 mEq/L to 6.79 mEq/L, p = 0.36. See Figs. 1 and 2.

**Conclusion**

There are many limitations in using serum creatinine and eGFR as measures of renal function^3^4. Moreover, recently discovered biomarkers are not currently used in routine practice due to costs (e.g. TIMP-2-IGFBP). Although direct measurement of urinary NH4 + is not currently standard practice, it could offer valuable insights into tubular acid excretory function. Our findings reveal a statistically significant difference in NH4 + concentration between healthy volunteers and individuals with impaired renal function, highlighting the potential of low-cost urinary NH4 + measurement. Further research is necessary to establish clinically relevant cut-off values.

**References**

1. Nagami GT. Luminal secretion of ammonia in the mouse proximal tubule perfused in vitro. J Clin Invest. 1988;81(1):159. 10.1172/JCI113287
2. Gruzdys V, Cahoon K, Pearson L, Raphael KL. Measurement of Urinary Ammonium Using a Commercially Available Plasma Ammonium Assay. Kidney360. 2022;3(5):926–932. 10.34067/KID.0000262022
3. Alaini A, Malhotra D, Rondon-Berrios H, et al. Establishing the presence or absence of chronic kidney disease: Uses and limitations of formulas estimating the glomerular filtration rate. 2017;7(3):73–92. 10.5662/wjm.v7.i3.73
4. Thomas D, Zachariah S, Elamin A, Elamin E, Luay A, Hashim O. Limitations of serum creatinine as a marker of renal function. Sch Acad J Pharm. 2017;6(5):168–170. 10.21276/sajp

**Fig. 1:**
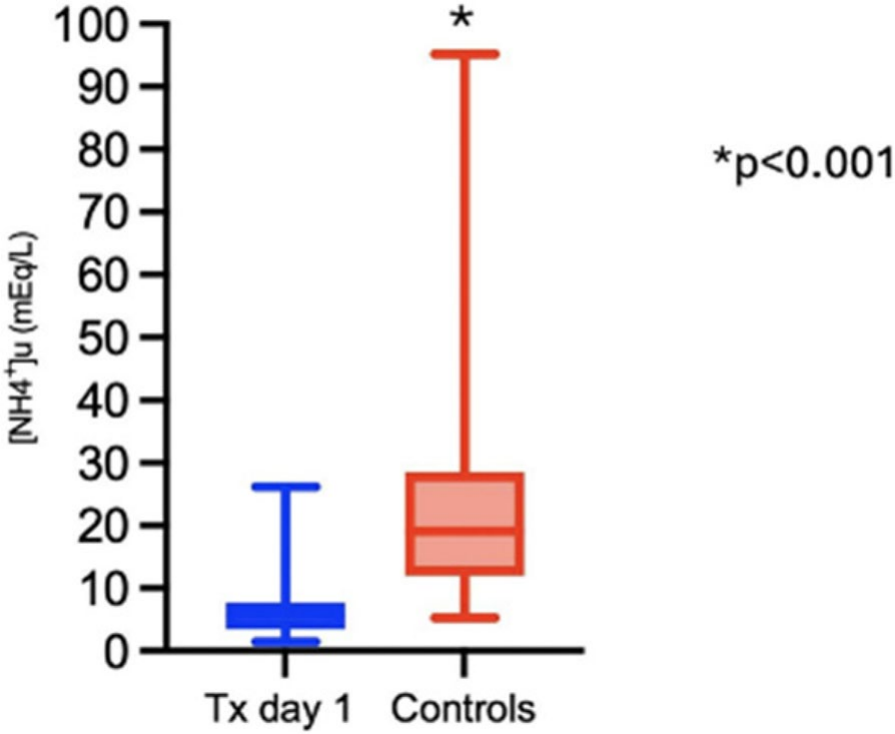
**(abstract A111).** [NH4^+^]_u_ in translated patients in day-1 and controls. Median (IQR) and min to max

**Fig. 2:**
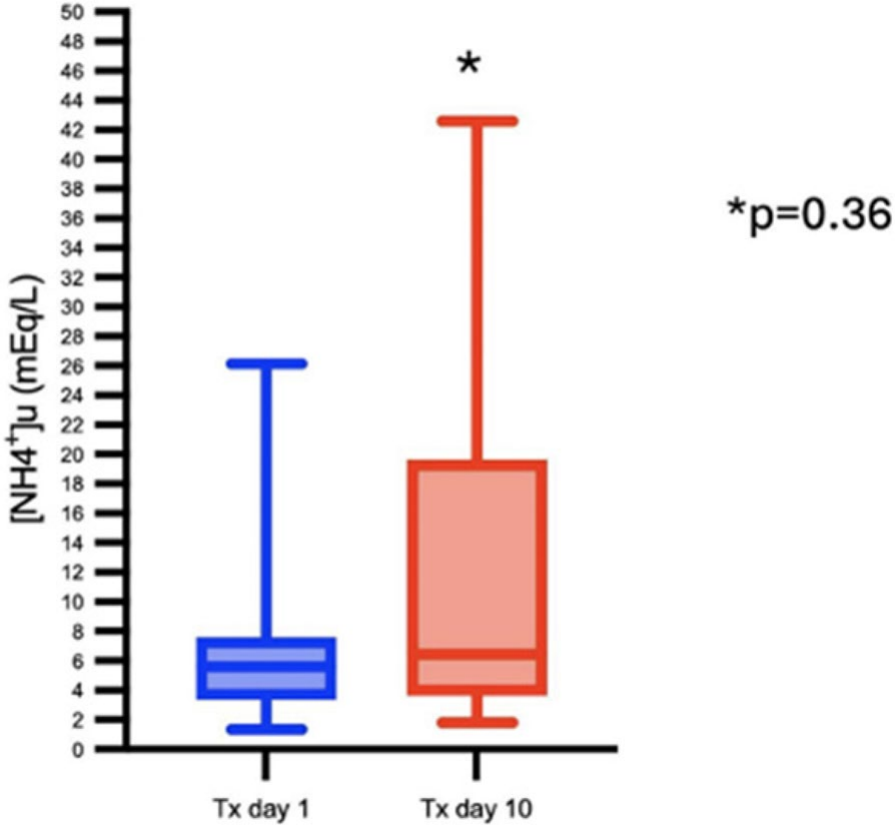
**(abstract A111).** [NH4^+^]_u_ in translated patients in day-1 and day-10. Median (IQR) and min to max

**Table 1:** **(abstract A111).** See text for description

[NH4^+^]_u_ mEq/L	Tx day 1 (n = 16)	Tx day 10 (n = 17)	Controls (n = 34)
Median (IQR)	4.98 (4.01)	6.79 (13.99)^*^	19.15 (16.08)^**^
Min	0	1,81	5,29
Max	26,16	42,59	95,17

*IQR* Interquartile range, *Min* minimum value, *Max* maximum value, *Tx* renal transplantation, *[NH4*^*+*^*]*_*u*_ urinary ammonium concentration

^*^p<0.01 Tx day 1 vs. controls

^**^p=0.36 Tx day 1 vs. Tx day 10

### A113 Successful use of cytosorb hemoadsorption in a patient with neuroleptic malignant syndrome, sepsis and acute renal failure

#### M. Leonardi, M. Grassi Bertazzi, M. Sidoti, M. Raciti, D. Grasso, B. Lanzafame, G. Scrofani, D. Sapienza, S. Borraccino, C. Di Tommaso, O. Sciuto, G. Bucolo, M. Modafferi

##### Ospedale Cannizzaro, Catania, Italy

###### **Correspondence:** M. Leonardi

*Journal of Anesthesia, Analgesia and Critical Care 2024*, **4(1):**A113

**Case presentation**

A 39-year-old woman was admitted to the Emergency Department for psychomotor agitation and a high temperature 39 °C. Vital signs were: GCS 15, NIBP 140/50 mmhg, SpO2 98%. Known medical history included intellectual disability and febrile seizures during her childhood. After 24 h, her clinical condition started to rapidly deteriorated, including altered mental status (GCS 8), respiratory failure (SpO2 < 87%), hypotension (NIBP < 80/40 mmhg), ongoing fever, and imminent renal failure( urinary output < 0,5 ml/kg/h). Norepinephrine infusion was initially started at 0.05 µg kg/min had to be increased to 0.15 µg /kg/min. The patient was then transferred to the ICU. Broad spectrum antibiotics were prescribed. Neuroleptic malignant syndrome (NMS) was suspected to be the main cause of symptoms.

Bicarbonate was administered to alkaline her urine, diuresis was stimulated with high levels of furosemide and crystalloid was infused in order to replace the intravascular volume. Despite, she went on to develop multiple organ dysfunction including ira, while hyperthermia was refractory to paracetamol. CVVHDF was instituted.

During the first three days of CVVHDF, body temperature decreased to 37 °C. However, serum myoglobin and creatine phosphokinase (CPK) plasma levels were still high, while the anuria persisted despite continuous administration of diuretics, adequate volume replacement and a stable mean arterial pressure. Given this critical condition, four cycles of plasmapheresis were performed as an attempt to partially reduce serum myoglobin levels, without effect however. On day 10 of her ICU stay, the decision was made to install a CytoSorb haemoadsorption cartridge into the CVVHDF circuit in a pre-dialyzer position to reduce myoglobin plasma concentrations and to support the standard therapy. Two consecutive CytoSorb treatments (24 h per treatment) were run at a blood flow rate of 100 ml/min using citrate anticoagulation. This was followed by a continued decrease in serum myoglobin levels from 34,236 µg/l to 8607 µg/l after the first cycle, reaching 567 µg/l at the end of the treatment (Table 1). Spontaneous diuresis recurred on day 20 with gradual increasing volumes thereafter. Cytosorb treatment as did renal function as evidenced by a gradual decrease in creatinine (3 to 1.28 mg/dl) and blood urea nitrogen (93 to 25 mg/dl) levels throughout the treatment.

In our patient, plasmapheresis as well as CVVHDF were not able to significantly reduce myoglobin levels nor to improve the patients hyperinflammatory state. We therefore instituted adjunctive CytoSorb therapy with the aim to lower myoglobin but also to counteract the sepsis-related hemodynamic instability and to control hyperinflammation. In line with previous publications, from the start of hemoadsorption therapy in our patient we saw a rapid and clear reduction in myoglobin, reduction in vasopressor dosages and control of the overwhelming inflammatory response [4, 5, 10]. Tihis case emphasizes another potential indication for CytoSorb in a rare but severe syndrome such as neuroleptic malignant syndrome, in which extracorporeal treatment such as plasmapheresis may be ineffective.

Informed consent was obtained.

**Table 1:** **(abstract A113).** See text for description.

	Cytosorb treatment				Cytosorb treatment		
	Day 0	Day 1	Day 2	Day 3	Day 4	Day 5	
Myoglobin (mg/L)	34,326	25,000	8607	4033	1122	567	
BUN (mg/dL)	93	63	51	56	27	25	
Creatinine (mg/dL)	3	2,49	1,52	1,5	1,53	1,28	1

### A114 Doege-potter syndrome and management of perioperative persistent hypoglycaemia: a case report

#### L. Farina^1^, G. Ferrara^2^, A. Discenza^3^, A. Basta^4^, S. D addato^5^, L.P. Cantatore^6^, E.P. Ricci^7^, A. Cotoia^8^

##### ^1^Università degli Studi, Foggia, Italy; ^2^Università degli Studi, Foggia, Italy; ^3^Università degli Studi, Foggia, Italy; ^4^Università degli Studi, Foggia, Italy; ^5^Università degli Studi, Foggia, Italy; ^6^Università degli Studi, Foggia, Italy; ^7^Università degli Studi, Foggia, Italy; ^8^Università degli Studi, Foggia, Italy

###### **Correspondence:** L. Farina

*Journal of Anesthesia, Analgesia and Critical Care 2024*, **4(1):**A114

**Background:** Surgical stress is typically characterized by hyperglycaemia (named also “surgical diabetes”). Unlike some paraneoplastic syndromes such as the Doege-Potter Syndrome (DPS) are linked to persistent hypoglycaemia even during surgical procedures. Furthermore, low doses of prednisone are not always effectives at relieving hypoglycaemia and complete tumor resection remains the only definitive treatment. We present a case report of DPS managed with Enhanced Recovery After Surgery (ERAS) to control perioperative glycaemia.

**Case Report:** A 84 years old female, with negative medical history, was admitted to Emergency Department for severe hypoglycaemia (glycaemia 37 mg/dl) and loss of consciousness. Chest X-Ray and total Body CT scan showed a pleural neoformation (10.6 × 14.9x11cm) (Fig. 1).

Biopsy samples were performed and Doege-Potter Syndrome was diagnosed. During the hospitalization intravenous glucose infusion was admistered to reach a glycaemic range of 55 to 79 mg/dL and the mass removal surgery excision was planne. Perioperative anesthetic management to maintain euglicemia consisted in intravenous glucose solution administration at 30 ml/h from 18.00 the day before surgery and progressively reduced at 20 ml/h until 2.00 a.m on the day of surgery. The oral administration of maltodextrins was 800 ml the night before surgery followed by 400 ml on the day of surgery as suggested by ERAS protocol. The Prednisone 50 mg was also administered 4 h before surgery (Fig. 2). The postoperative glycemic values were 92 to 112 mg/dL.

**Conclusion:** The administration of maltodextrins in combination with continuous infusion of glucose showed to be useful in maintaining the euglicemia in surgical patient with Doege-Potter syndrome.

Informed consent was obtained.

**Fig. 1:**
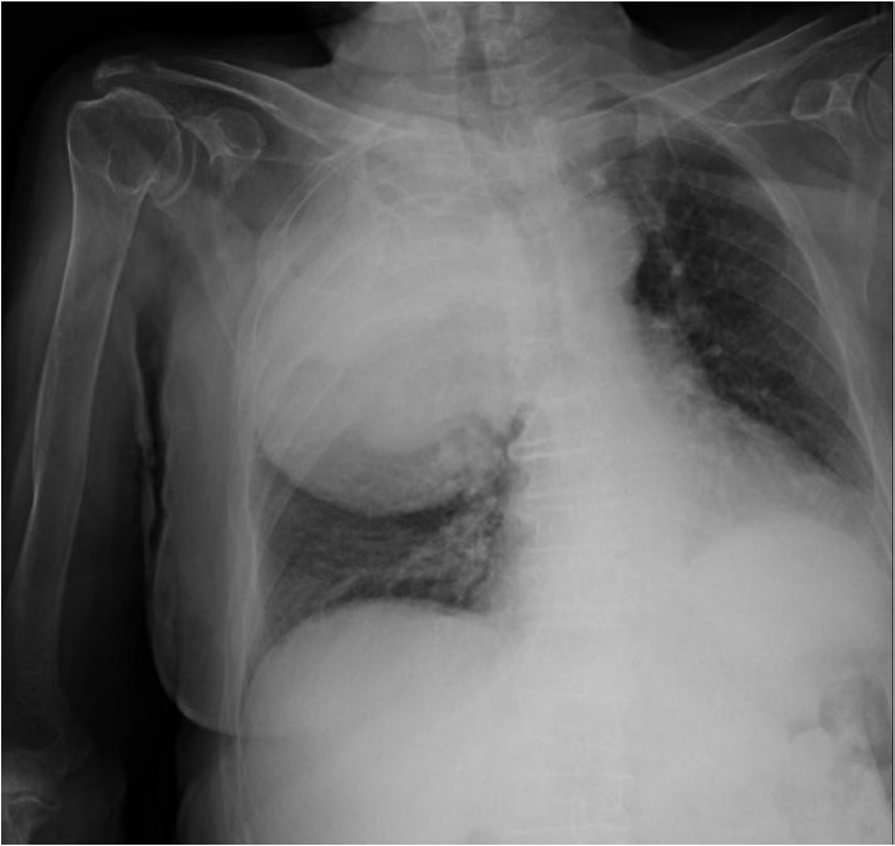
**(abstract A114).** Patient’s chest x-ray showing intrathoracic neoformation

**Fig. 2:**
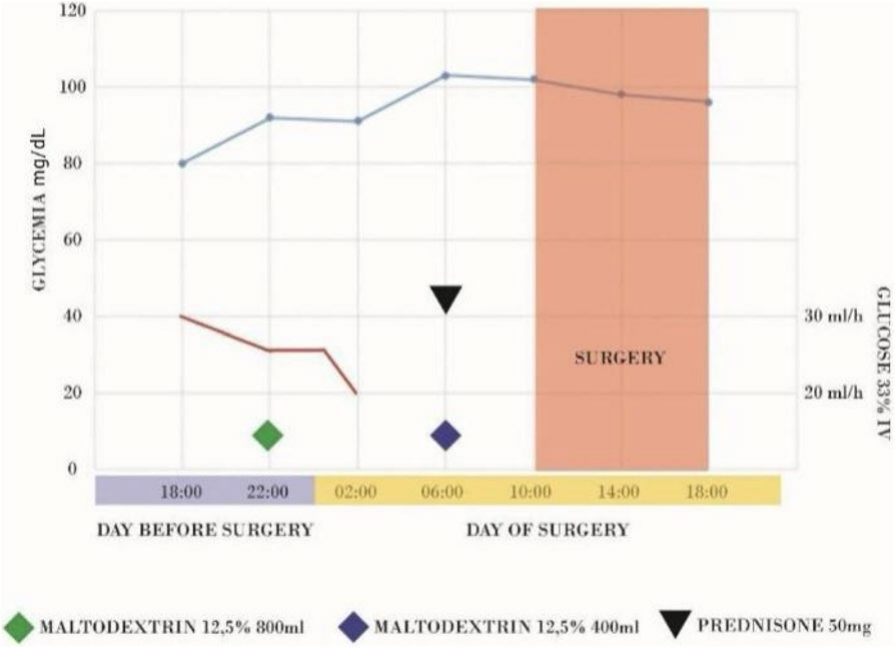
**(abstract A114).** Perioperative glycaemia management. Administration of IV Glucose 33% (red line). Glycemia value (blue line)

### A115 Use of cytosorb for treatment of Car-T therapy-induced CRS

#### M.S. Cassatella, C. Alfonso, C. Pierdomenico, D. Valentina, G. Marco, G. Grazia, S. Michele, S. Grazia, Z. Antonio, D. Michele

##### Ospedale Dimiccoli, Barletta, Italy

###### **Correspondence:** M.S. Cassatella

*Journal of Anesthesia, Analgesia and Critical Care 2024*, **4(1):** A115

**Background**

The advent of CAR-T cell therapy has notably progressed blood-related cancers treatment. Yet, the therapy's downsides, like cytokine release syndrome (CRS) and CRS-linked coagulopathy, can be severe and fatal [1;2;3]. CRS causes systemic symptoms and it can swiftly progress to cytopenia, hypotension, tachycardia, tachypnea, hypoxemia, arrhythmias, capillary permeability, coagulation disorders, respiratory insufficiency, shock, and multi-organ dysfunction [1;2;3]. The typical treatment for CRS involves vital support, transfusions, and Tocilizumab, a monoclonal antibody that binds to interleukin-6.

**Case Report**

CytoSorb is a sorbent cartridge made of a polystyrene-divinylbenzene copolymer coated with polyvinylpyrrolidone, certified for modulating the blood concentration of inflammatory cytokines too, which can be a supportive treatment in this therapy’s complication.

A 43-year-old patient, suffering from IgG lambda multiple myeloma since April 2022, underwent Teclistamab treatment from 2023, initiating subsequent CAR-T therapy. On the following day post-initiation, the patient presented at the Emergency Department with hyperpyrexia and thoracic pain, progressing to hypotension, cardiogenic shock, and subsequent cardiocirculatory arrest, revived after a cycle of cardiopulmonary resuscitation. Consequently, the patient was transferred to the intensive care unit (ICU), on mechanical ventilation and administration of high-dosage catecholamines, as well as broad-spectrum antibiotics. Initial hematochemical analyses revealed: Procalcitonin (PCT) > 100 ng/ml; leukopenia (0.42*103/uL); thrombocytopenia (10*103/uL); and anaemia (HB 8.1 g/dl). Cultures yielded negative results. A diagnosis of CAR-T induced CRS was established, prompting commencement of transfusion support and Tocilizumab therapy.

On day 2, persistent hemodynamic instability, elevated VIS, PCT > 100 ng/ml, and the emergence of acute renal failure (creatinine 2.5 mg/dl) necessitated the patient's initiation onto Continuous Veno-Venous Hemodiafiltration (CVVHDF), with CytoSorb therapy.

On day 3, during the second CytoSorb cycle, a significant reduction in PCT levels, concomitant with reduced catecholamine dosages, were observed, with improving respiratory parameters.

By the day 4, following additional CytoSorb administration, hematologic tests indicated further improvement in inflammatory indices. Norepinephrine administration was discontinued, and respiratory weaning was initiated.

On day 5, CVVHDF and CytoSorb were discontinued, the patient exhibited normal hemodynamic and was extubated.

**Conclusion**

Final hematological assessments on the 6th day showed a continued reduction in inflammatory markers, concurrent with improved blood cell count, apyrexia, optimal respiratory exchange, and normalized blood pressure readings. Patient was discharged from ICU (Figs. 1,2,3,4).

In conclusion, we managed to stabilize a critical situation by using CytoSorb as a supportive therapy within the context of CAR-T-induced CRS. We believe that the use of CytoSorb represents a valid supportive treatment for the potentially life-threatening complications of CRS.

Informed consent was obtained.

**Refences**

1. Dibas A, Rhiel M, Patel VB,et Al. Cell-Based Models of 'Cytokine Release Syndrome' Endorse CD40L and Granulocyte–Macrophage Colony-Stimulating Factor Knockout in Chimeric Antigen Receptor T Cells as Mitigation Strategy. Cells. 2023 Nov 6;12(21):2581.
2. Zhang X, Z hu L, Zhang H, et Al. CAR-T Cell Therapy in Hematological Malignancies: Current Opportunities and Challenges. Front Immunol. 2022 Jun 10;13:927,153.
3. Schubert ML, Schmi tt M, Wang L, et Al. Side-effect management of chimeric antigen receptor (CAR) T-cell therapy. Ann Oncol. 2021 Jan;32(1):34–48.

**Fig. 1:**
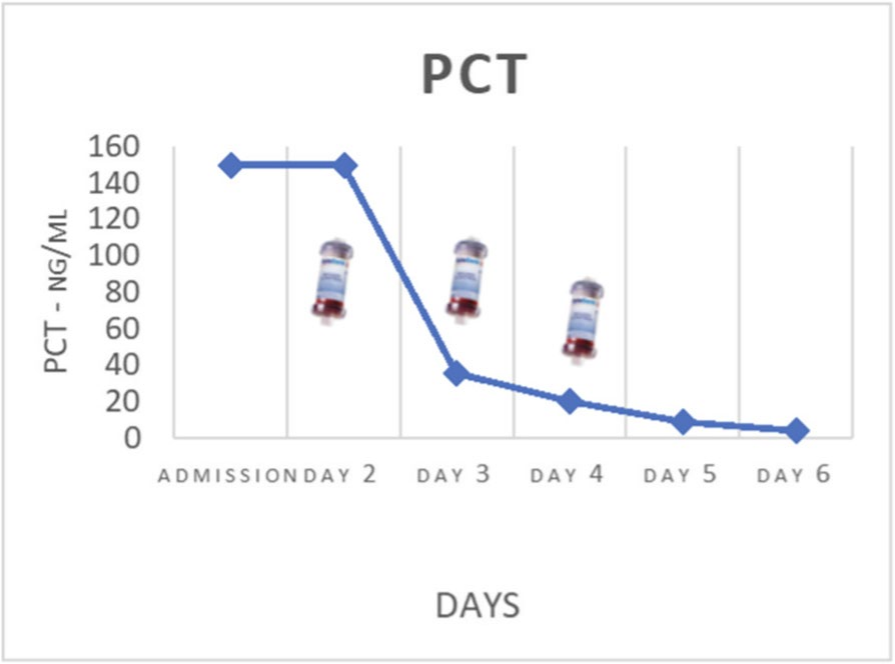
**(abstract A115).** Procalcitonin during days

**Fig. 2:**
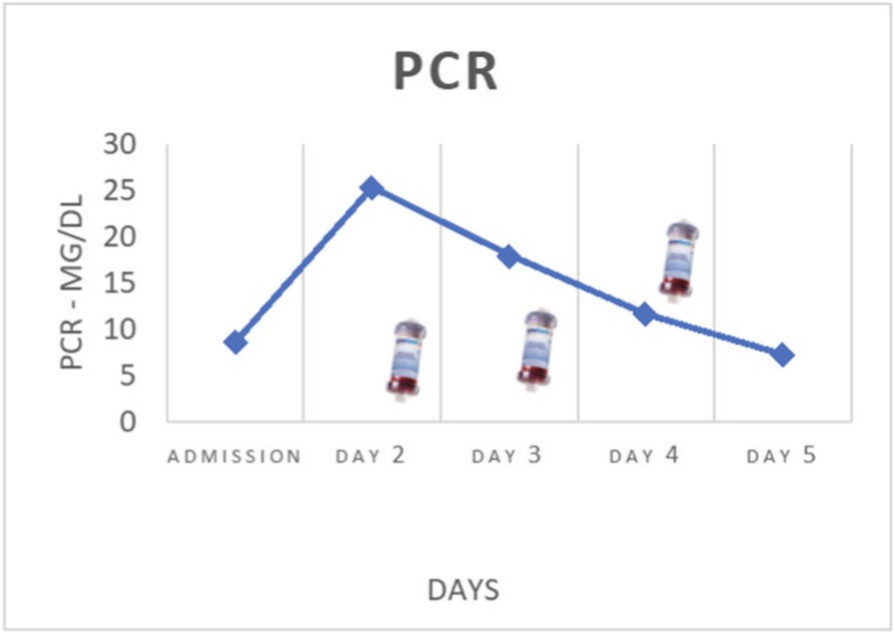
**(abstract A115).** C-reactive Protein during days

**Fig. 3:**
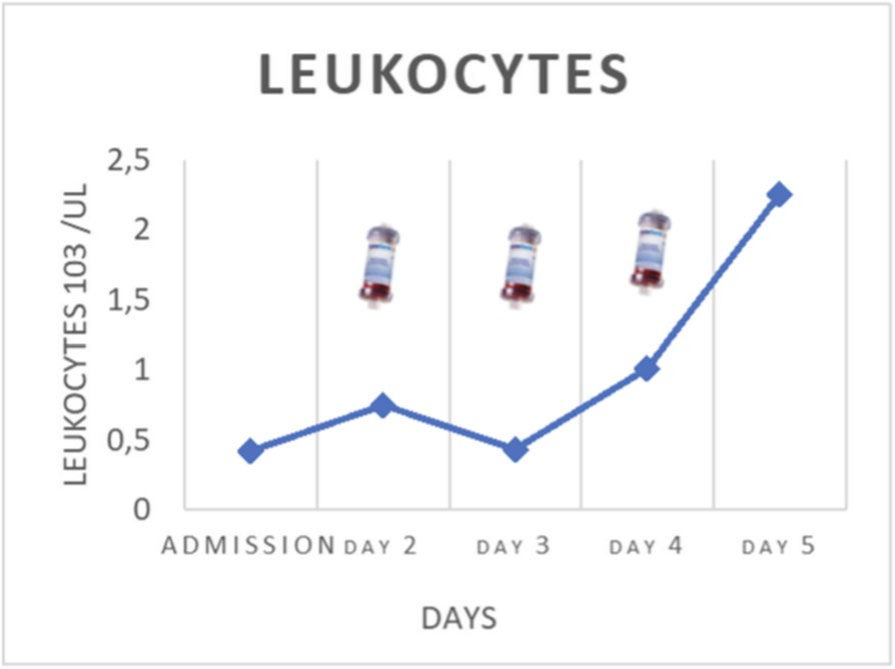
**(abstract A115).** Leukocytes during days

**Fig. 4:**
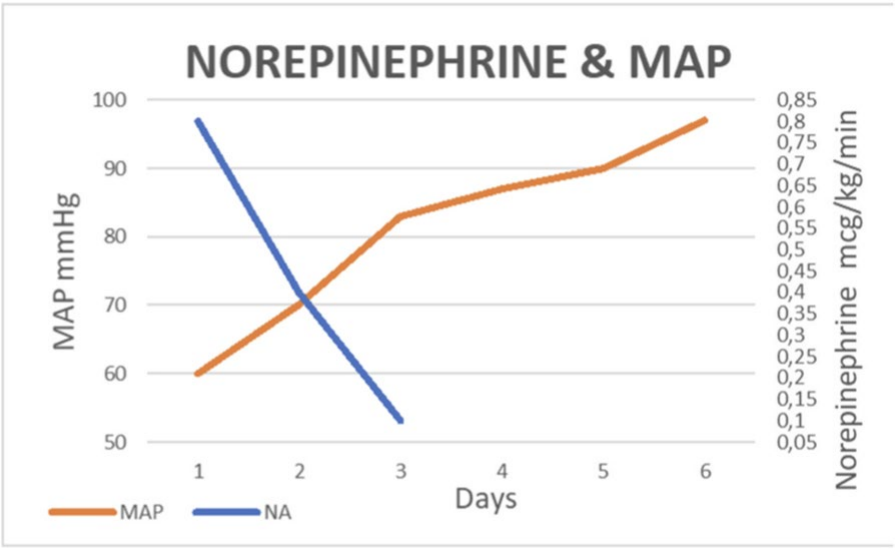
**(abstract A115).** Mean Arterial Pressure and Norepinephrine during days

## Infections and sepsis

### A116 Never underestimate an epiglottitis, mostly in adults: our experience

#### F. Falso, S. Ferraro, D. Abbenante, S. Talia, R.C. De Rosa

##### AORN Ospedali dei Colli—Department of Anesthesia and Intensive Care—D. Cotugno Hospital, Naples, Italy

###### **Correspondence:** F. Falso

*Journal of Anesthesia, Analgesia and Critical Care 2024*, **4(1):**A116

A 36-year-old man, with history of asthma, was admitted to the emergency department with fever, dysphagia to solids and liquids and hoarseness, after two days of sore throat treated only with analgesics. The laboratory exams showed severe leukopenia (110 cells/mcL, 10 neutrophils/mcL), elevated C reactive protein (37.7 mg/dL), procalcitonin (76.9 ng/mL) and positive nasal swab to SARS-CoV-2. A neck CT scan showed an irregularly thickened hypopharynx, airway caliber reduction and multiple lymphadenopathies. An ear nose and throat (ENT) consultation with laryngoscopy didn’t show significant anomalies and prescribed follow-up. The patient was admitted to the intensive care unit (ICU) due to the risk of acute decompensation. We prescribed an empiric antibiotic therapy with IV meropenem 1 g tid, linezolid 600 mg bid, azithromycin 500 mg/die and liposomal amphotericin B 240 mg/die, as well as filgrastim 30 MUI/day for five days and IgM-enriched human intravenous immunoglobulin (Pentaglobin®) 5 ml/kg/day. A Biofire® FilmArray® Pneumonia Plus (FAPN) test, performed on sputum, showed positivity to Staphylococcus aureus, Streptococcus pneumoniae and Haemophilus influenzae. Neoplastic causes of agranulocytosis were excluded. On day 4 (D4), the dyspnea, stridor and airway obstruction suddenly worsened. So, after a laryngoscopy showing signs of severe epiglottitis, we immediately intubated the patient and the ENT surgeon proceeded to urgent surgical tracheostomy. A period of sedation and mechanical ventilation was necessary. On D6, a magnetic resonance imaging (MRI) showed colliquation of some neck lymph nodes and mediastinitis. In the following days, after normalization of WBC, reduction of CRP and PCT and improvement of general clinical conditions, drainage of colliquated cervical lymph nodes, a weaning from sedation and mechanical ventilation was started with gradual success. On D29 the patient was transferred to an ordinary ward in valid spontaneous breathing through tracheostomy. In the case we described, the patient had two very serious life-threatening conditions: [1] epiglottitis and [2] severe leukopenia with agranulocytosis. Regardless of the apparent stability, epiglottitis can suddenly deteriorate to life-threatening airway obstruction (1). Its most common causes include H. influenzae, S. aureus and S. pneumoniae. With signs of upper airway obstruction, the main target is to guarantee airway patency, necessarily preceding diagnostic evaluation (2). Thus, the patient must be appropriately monitored and promptly intubated by highly skilled operators. Most deaths are the result of cardiorespiratory arrest, secondary to unsecured airway obstructions (3). The clinical picture was exacerbated by the patient’s immunosuppression, probably induced by abuse of analgesics and coronavirus infection. The patient gave written informed consent for the publication of this report.

**References**

1. Ames WA, Ward VMM, Tranter RMD, Street M. Adult epiglottitis: an under-recognized, life-threatening condition. Br J Anaesth. 2000 Nov;85(5):795–7.
2. Park KW, Darvish A, Lowenstein E. Airway Management for Adult Patients with Acute Epiglottitis. Anesthesiology. 1998 Jan 1;88(1):254–61.
3. Bridwell RE, Koyfman A, Long B. High risk and low prevalence diseases: Adult epiglottitis. Am J Emerg Med. 2022 Jul;57:14–20.

### A117 Lemierre’s syndrome: a clinical challenge

#### P. Coppola, A. Galdieri, L. Falco, V. Galluccio, L. Lombardi, E. Faraone, S. Esposito, V. Narni Mancinelli, A. Napolitano, V. Vincenti

##### UOC di Anestesia e Rianimazione OO.RR, Area Nolana—ASL Napoli 3 Sud, Nola, Italy

###### **Correspondence:** P. Coppola

*Journal of Anesthesia, Analgesia and Critical Care 2024*, **4(1):**A117

**Background**

Lemierre’s syndrome is a rare, life-thretening complication of an acute oropharyngeal infection causing thrombophebitis of the internal jugular vein and sepsis. It typically affects healthy young adults. The causative agent is typically an anaerobic Gram-negative germ. The mechanism is unclear1, but can carry a high mortality rate if not recognized and treated aggressively2.

**Case Report**

Female, 18 y.o. Recent febrile episode with lateral cervical lymphadenopathy, cough, with diagnosis of mononucleosis (positive virology: EBV VCA IgM; EBV VCA IgG), treated at home with a third generation cephalosporin for 5 days, thereafter apparent remission. She came to the emergency room for persistent fever, drowsiness, cough and asthenia. The clinical examination showed hyperemia of the oropharynx, abnormal reduction of vesicular murmur bilaterally, signs of septic shock, SBP < 70 mmHg, HR > 110 min, oliguria. Laboratory tests showed: WBC 7000, relative neutrophilia (97%) and lymphocytopenia (< 6%), thrombocytopenia (25,000/mcL), PCT > 36 ng/ml, CRP > 18 mg/dL, pa02 55 mmHg (Venturi mask with fi02 0,60), Sp02 86%, lactates 1,5 mmol/L, D-Dimer > 6000 mcg/L; fibrinogen > 400 mg/dL; EAA 0.82; SOFA SCORE 13, Sars-Cov 2 negative. On CT: bilateral pulmonary infiltrates, splenomegaly, hepatomegaly, multiple lymphnode swellings. Negative echocardiogram for pulmonary embolism. Upon admission to the Intensive Care Unit, blood culture sampling, antibiotic therapy with meropenem, vancomycin and levofloxacin (suspended at negative result for Legionella P.), midline in the right cephalic vein, sedation with dexmedetomidine 0.4–0.6 mcg/kg/h, norepinephrine 0.15–0.20 mcg/kg/min, treatment with human immunoglobulins, alternating NIV with HFNO and alternating decubitus positions (right, left, prone). Furthermore, the blood FilmArray was negative for the most common germs and yeasts tested by the method. 48 h after admission, she showed a progressive improvement in her general conditions: more reactive, SBP > 100 mmHg, HR < 100 min, resumption of diuresis, TC < 38° C, sp02 94%, pa02 126 mmHg, lactates < 2 mmol/L, half PCT values (< 12 ng/ml), increase of platelets (> 100.000/mcL), reduction of D-Dimer 636 µg/ L, fibrinogen 207 mg/dL. On the third day laboratory reported the presence in the blood cultures of an anaerobic Gram negative germ (Veillonella Parvula), sensitive to meropenem and metronidazole. Vancomycin was suspended, continuing the treatment with meropenem. A neck ultrasound, performed for pain, showed thrombophlebitis of the left internal jugular vein (never site of venipuncture), confirmed by a CT angiography of the neck vessels, giving rise to the picture of Lemierre's syndrome. 8 days after admission, she was transferred to the Internal Medicine department for continued treatment. Informed consent to publish had been obtained.

**Conclusion**

Probably the Epstein-Barr virus infection and the associated pharyngo-tonsillitis that affected the young patient may have favored thrombophlebitis of the internal jugular vein. An understanding of Lemierre’s syndrome can assist emergerncy clinicians in diagnosing and managing this potentially deadly disease3.

**References**

1. Tiwari A. Lemierre's Syndrome in the 21st Century: A Literature Review. Cureus. 2023 Aug 18;15(8):e43685.
2. Gore MR. Lemierre Syndrome: A Meta-analysis. Int Arch Otorhinolaryngol. 2020 Jul;24(3):e379-e385.
3. Carius BM. High risk and low prevalence diseases: Lemierre's syndrome. Am J Emerg Med. 2022 Nov;61:98–104

### A118 Hemodynamic management during septic shock in patient with obstructive hypertrophic cardiomyopathy and subarachnoid hemorrhage

#### D.J. Brunetti^1^, F. Leonardis^1^, M. Dauri^2^, D.G. Biasucci^2^

##### ^1^Terapia Intensiva, Policlinico Tor Vergata, Roma, Italy; ^2^Dipartimento di scienze cliniche e medicina Traslazionale, Università di Roma Tor Vergata, Roma, Italy

###### **Correspondence:** D.J. Brunetti

*Journal of Anesthesia, Analgesia and Critical Care 2024*, **4(1):**A118

62-year-old female patient, operated 7 years ago for kidney transplant for PKD and hypertrophic obstructive cardiomyopathy, admitted to intensive care for rupture of aneurysm of the left ICA and subsequent subarachnoid hemorrhage.

In the three days following the hemorrhage, poor neurological status persists with the impossibility of extubation. After four days, fever occurs, CT 38.5°, WBC 13 thousand/µL, PCT 21 ng/ml and CRP 187 ml/L, serum creatinine 1.5 with initial phase of oliguria and output urinary flow reduced to 0.2 ml/kg/h for approximately 3 h associated with marked hypotension BP 75/40 (MAP 52) mmHg, sinus tachycardia with HR 140 bpm and SpO2 95% (Tab 1).

Fluid challenge is carried out with Ringer's Lactate 2000 cc with no benefit on haemodynamics and therefore norepinephrine infusion 0.2mcg/kg/min is started with minimal benefit: BP 90/50 (MAP 63 mmHg), HR 135 bpm. At the same time, microbiological are collected and broad-spectrum antibiotic therapy is started.

After an hour, sinus tachycardia persists, HR 140 bpm and MAP < 65 mmHg. After a further unsuccessful increase in the dosage of Norepinephrine to 0.3mcg/Kg/min, the infusion of Argipressin 1.8Ui/h is started followed by a modest optimization of blood pressure: 105/55 mmHg (HR 130 bpm) (Fig. 1).

To ensure control over the heart rate, infusion of Landiolol is started at a dosage of 1mcg/Kg/min until the dosage of 5mcg/Kg/min is reached within an hour with a good response on the heart rate, regularization at 86 bpm with BP 130/ 70 mmHg and optimization of echocardiographic parameters (Fig. 2-3).

An hour later there HR 82 bpm, BP 136/71 mmHg. At this point we proceed to reduce the dosage of norepinephrine to 0.15mcg/kg/min.

48 hours after the start of the shock phase, microbiological tests indicate a positivity of the blood samples for E. Coli sensitive to Ceftazidime/Avibactam.

Informed consent was collected from the patient's family members.

**Discussion**

The Surviving Sepsis Campaign proposes to start AVP in septic shock when the norepinephrine dose is between 0.25 and 0.5 mcg/kg/min [1–2]. In our clinical case, Argipressin was inserted when the dosage of Noradrenaline was greater than 0.3mcg/Kg/min, resulting in a satisfactory hemodynamic response. However, the desire to have greater control over hemodynamics was due to the need to guarantee an acceptable cerebral perfusion pressure for which, given the obstructive hypertrophic cardiomyopathy and given the echocardiographic evidence (Fig. 3) of a distolic dysfunction, it was not recommendable further increase peripheral resistance or increase the pre-load. In fact, diastolic dysfunction in septic shock is not a rare occurrence and is considered an early predictor of mortality [3–4]. Therefore, the use of Landiolol probably allowed us to reduce the heart rate not only allowing better control of the rhythm but also improved cardiac performance by improving the diastolic function and consequently the stroke volume (SV) and finally allowed us to reduce the dosage. of norepinephrine [5–6].

**Conclusions**

In our experience, in cases of septic shock and unstable hemodynamics, especially when conditions of previous cardiac dysfunction exist, the use of Argipressin and Landiolol can facilitate the decrease in catecholamines, thus improving cardiac performance and possibly reducing mortality.

**Fig. 1:**
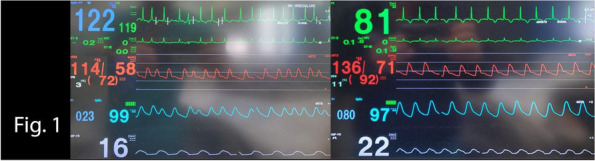
**(abstract A118).** See text for description

**Fig. 2:**
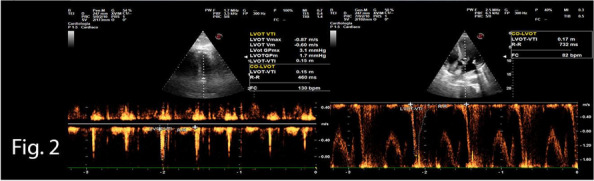
**(abstract A118).** See text for description

**Fig. 3:**
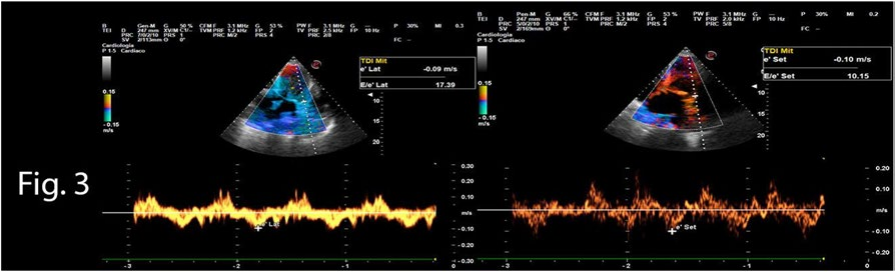
**(abstract A118).** See text for description

**Table 1:** **(abstract A118).** See text for description

Parameter	T0 (Shock phase)	T1 (Noradrenaline 0.3mcg/Kg/min)	T2 (Argipressin 1.8Ui/h)	T3 (Landiolol 5mcg/Kg/min)
PA	75/40 (52) mmHg	90/50 (63) mmHg	114/58 (72) mmHg	136/71 (92) mmHg
FC	140 bpm	138	122 bpm	81 bpm
Lattati	10	10	9	4
LVOT VTi	0,09 m	0,11	0,15	0,17
CI	1,6L/min/m2	2 L/min/m2	3,1 L/min/m2	4,3 L/min/m2
FE	22%	-	25%	35%
E/A	2,1	-	2	1,5
E/E’	19	-	17,3	10,1

### A119 Early platelet dysfunction in patients with sepsis and septic shock in intensive care unit: a prospective study

#### A. Blandina^1^, M.G. Bocci^2^, E. De Candia^3^, D. Natalini^3^, E. Cingolani^4^, F. Botta^1^, M. Caravella^1^, S. Carelli^3^, A.M. Ionescu^5^, R. Maviglia^3^, C. Gori^4^

##### ^1^Università Cattolica del Sacro Cuore, Roma, Italy; ^2^INMI L. Spallanzani IRCCS, Roma, Italy; ^3^Fondazione Policlinico Universitario Agostino Gemelli IRCCS, Roma, Italy; ^4^Azienda Ospedaliera San Camillo, Roma, Italy; ^5^Ospedale Belcolle Viterbo, Viterbo, Italy

###### **Correspondence:** A. Blandina

*Journal of Anesthesia, Analgesia and Critical Care 2024*, **4(1):**A119

**Background**

Coagulopathy plays an important role in progression from sepsis to septic shock and represents an independent factor of adverse outcome. Platelets are essential mediators in inflammation acute phase and acquired immune responses. Thrombocytopenia in septic shock patients is well documented. However, little is known about platelet function in sepsis early phase. This study aims to:

- investigate the correlation between platelet dysfunction and sepsis markers
- compare viscoelastic tests and reference coagulation methods (LTA, Light Transmission Aggregometry)
- evaluate platelet function by measuring the expression of p-selectin, fibrinogen receptor (PAC-1) and CD40L count.

**Materials and Methods**

The prospective, single-centre study enrolled 10 adult patients suffering from sepsis or septic shock who were hospitalized at A. Gemelli University Polyclinic Foundation ICU, and 7 healthy adult volunteers, since July 2019. For each patient, after obtaining informed consent, samples were taken at the time of admission (T0), after 48 h (T1) and after 7 days (T2). Blood samples from controls were taken only at T0. Blood samples were used to analyse:

- Platelets count
- Global platelet function by thromboelastography (TEG)
- Platelet aggregation with ADP and with epinephrine by optical aggregometry
- Platelet activation with p-selectin and fibrinogen receptor (Pac-1) dosage by flowcytometry and CD40L dosage by ELISA test.

These results were compared with standard coagulation test and were correlated with sepsis markers (procalcitonin) and SOFA score. p < 0.05 was considered statistically significant.

**Results**

Comparison of the median platelet count between patients and controls showed no significant differences in the two groups at T0; however, the patients’ platelet count underwent a progressive reduction over time, with a peak at T1 (Fig. 1). Moreover, patients showed a dysfunction of platelet aggregation, reduction of fibrinogen receptor expression and reduced dosage of CD40L compared to controls as early as T0 (Fig. 2-4). In our study, platelet disease also appears to be associated with the degree of severity of sepsis, independently of platelet count. High values of SOFA and procalcitonin were associated with lower values of platelet aggregation in viscoelastic tests and aggregometry, in particular at time T0 (Fig. 5).

**Conclusions**

Analysing platelet disease with different tests may increase diagnostic and therapeutic power in septic shock patients admitted to ICU. The severity of platelet disease, correlating with sepsis markers, could be an early index of pathology independently of platelet count. Furthermore, platelet dysfunction appears to precede thrombocytopenia; this finding could provide valuable information for early treatment of coagulative abnormalities in septic patients.

**Fig. 1:**
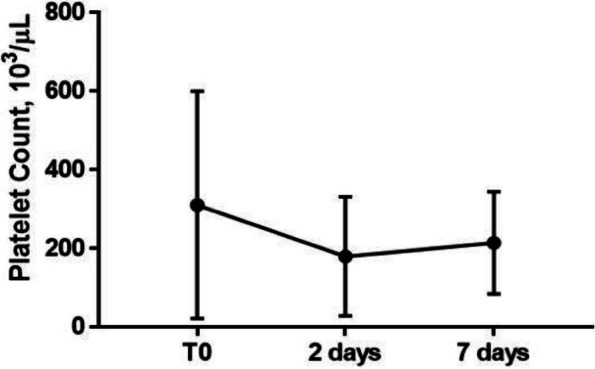
**(abstract A119).** Results of medians and interquartile ranges of platelet count of patients at three times

**Fig. 2:**
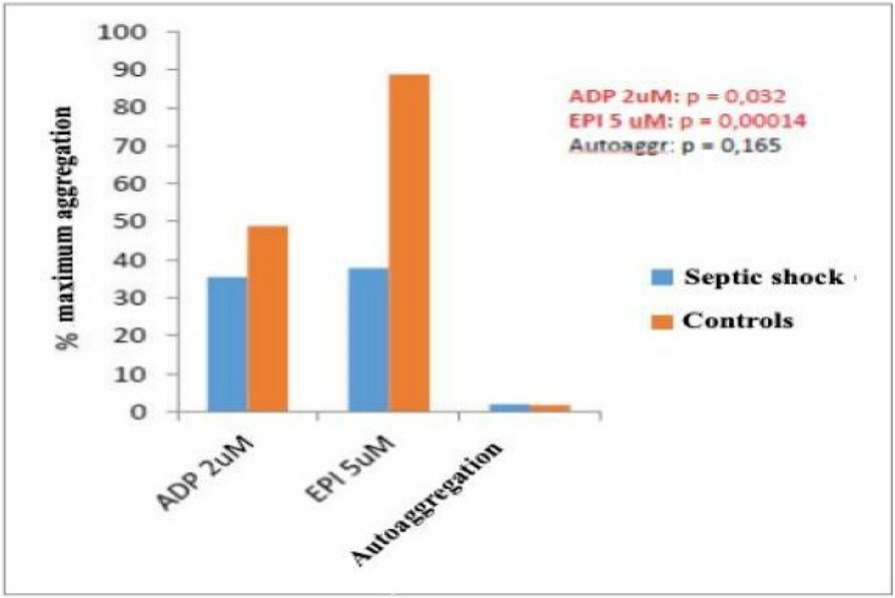
**(abstract A119).** Median results of T0, T1, T2 times of patients compared with median results of healthy volunteers

**Fig. 3:**
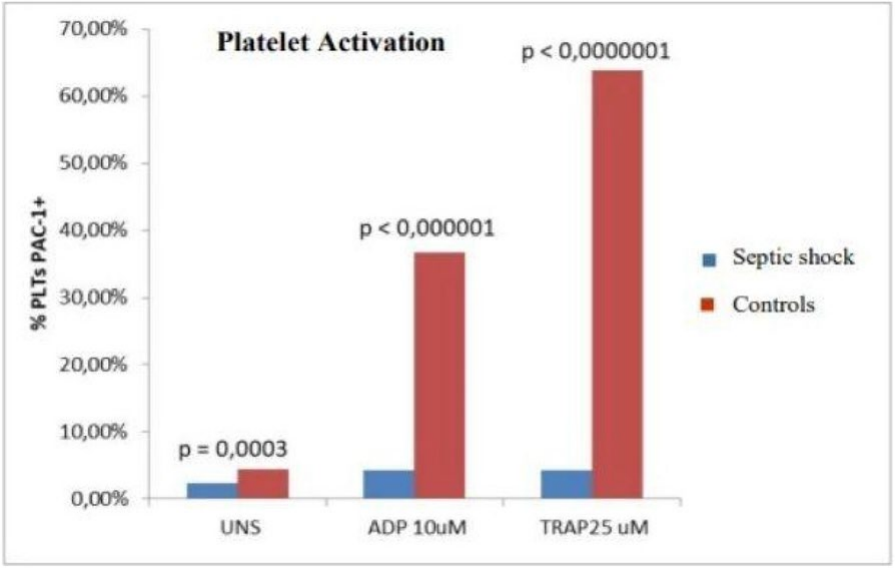
**(abstract A119).** median PAC-1 results of patients in the times compared with median results of healthy controls

**Fig. 4:**
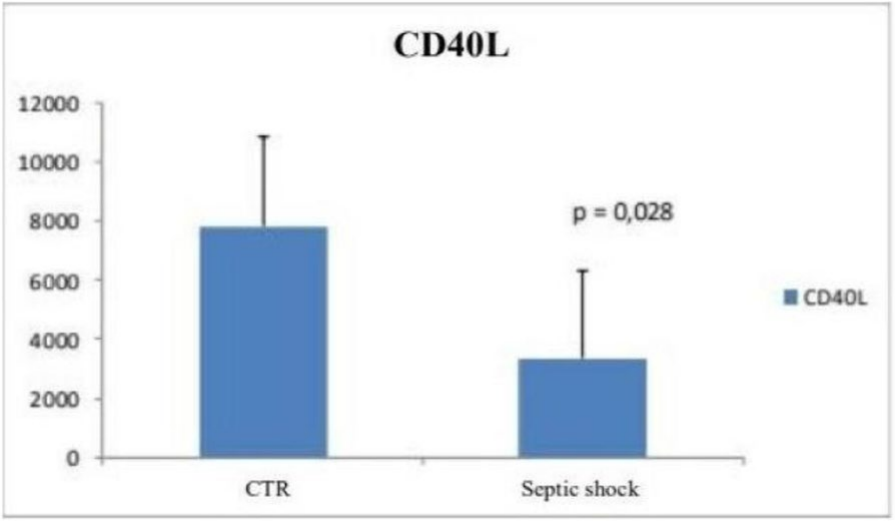
**(abstract A119).** Results of median and interquartile ranges of CD40L values in three-stroke patients compared with results of medians and interquartile ranges of T0 controls

**Fig. 5:**
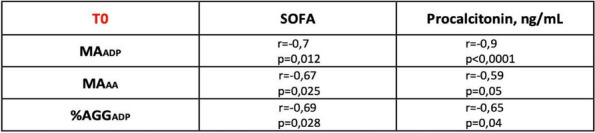
**(abstract A119).** Correlation between viscoelastic tests and aggregometry and procalcitonin and SOFA score at T0

### A120 Early extracorporeal membrane oxygenation for ards caused by legionella: a case report

#### M. Barberio, S. Ferraro, R. Giurazza, F. Falso, R.C. De Rosa

##### Department of Anesthesia and Intensive Care A.O.R.N. Ospedali dei Colli—P.O. Domenico Cotugno, Naples, Italy

###### **Correspondence:** M. Barberio

*Journal of Anesthesia, Analgesia and Critical Care 2024*, **4(1):**A120

A 36-year-old male, obese, without medical history, except recent COVID-19 infection, presented to our department for serious dyspnea. His initial vital signs were: desaturation, tachycardia, fever, hypotension. Laboratory evaluation showed white blood cell count of 23.7 10^3^/mL, serum creatinine of 2.5 m/ dL, procalcitonin of 10.4 ng/mL and a lactic acid level of 9.5 mmol/L.

A computed tomography scan of the chest showed diffuse bilateral consolidations. He was admitted with presumed COVID-19 ARDS. Due to the severity of clinical picture, mechanical ventilation, resuscitation with IV fluids, norepinephrine infusion 0.25 mcg/kg/min were started.

His arterial blood gas (ABG) post-intubation showed pH 7.12, PaCO2 45 mmHg and PaO2 59 mmHg under IPPV (tidal volume 500 ml x respiratory rate 25 breaths/minute, peak airway pressure 35 cmH2O, positive end expiratory pressure PEEP 10 cmH2O and fraction of inspired oxygen FIO2 1). Upon admission, Legionella urine antigen was positive so that Levofloxacin 750 mg and Azithromycin 500 mg, iv /die, were initiated.

After a cycle of pronation, without results, VV-ECMO cannulation (right femoral vein-right internal jugular vein configuration) was made (day zero).

The initial ECMO flow was 5.5 L/min, sweep gas fraction of delivered oxygen (FdO2) was 100% and a sweep gas flow was 6 L/min. After ECMO cannulation, oxygenation significantly improved and IPPV was set to “rest”; ABG showed pH 7.43, PaCO2 35 mmHg and PaO2 95 mmHg; shock condition rapidly improved and vasopressors were discontinued; lactate normalized. Legionellosis was confirmed by rapid molecular PCR testing for viral and bacterial pathogens (BioFire® multiplex PCR, 96.2% sensitivity, 98.3% specificity) on bronchoalveolar lavage (BAL).

The BAL PCR test was negative for COVID-19. After 5 days from beginning, the weaning from ECMO was started with decannulation on day 8. At the time of decannulation, ventilation modality was pressure support volume with support pressure 15 cmH2O above PEEP 5 cmH2O. While on ECMO, pulmonary infiltrates improved significantly. On day 10 he was estubated. On day 13 he was transferred out of the ICU and was discharged home on day 16 on room air. This case demonstrates the benefit of Legionella urine antigen and rapid molecular PCR testing for the diagnosis of Legionella as well as the utility of early VV-ECMO for ARDS caused by Legionella. ECMO survival for ARDS secondary to Legionella, in fact, is high, ranging from 73 to 85.7%. This case is unique because the positive COVID-19 test prior to admission was a diagnostic challenge.

Informed consent was obtained.

**References**

1. Naqvi A, Kapoor S, Pradhan M, Dicpinigaitis PV. Outcomes of severe Legio- nella pneumonia requiring extracorporeal membrane oxygenation (ECMO). J Crit Care. 2021;61:103–6.
2. Dorfman MV, Clark JD, Brogan TV. ECLS for Legionella: all ages welcome in the ELSO Registry. ASAIO J. 2020;66(2):226–9.
3. Andrea L, Dicpinigaitis PV, Fazzari MJ, Kapoor S. Legionella Pneumonia in the ICU: a Tertiary Care Center Experience over 10 years. Crit Care Explor. 2021;3(8):e0508

### A121 Linezolid concentration at different clinical scenarios of renal function, continuous veno-venous renal replacement therapy and continuous versus intermittent infusion: a retrospective study

#### L. Baggio^1^, S. Agliardi^2^, G. Gazzaniga^2^, M. Laratta^3^, S.M. Santambrogio^3^, G. Monti^3^, R. Fumagalli^1,3^

##### ^1^Department of Medicine and Surgery, University of Milan-Bicocca, Milano-Bicocca, Milan, Italy; ^2^Department of Medical Biotechnology and Translational Medicine, University of Milan, Milan, Italy; ^3^Department of Anesthesia and Intensive Care Medicine, Niguarda Ca' Granda, Milan, Italy

###### **Correspondence:** L. Baggio

*Journal of Anesthesia, Analgesia and Critical Care 2024*, **4(1):**A121

**Background**

Plasma levels of antimicrobials, can vary considerably in critical care patients due to several factors such as altered volume of distribution, albumin concentration, kidney function and Continuous Renal Replacement Therapy (CRRT). Linezolid, an antibiotic with time-dependent activity, has a low volume of distribution and it’s primarily metabolized by the liver and excreted only partially (33%) by kidneys. The desired range of linezolid concentration (Cmin) is narrow and was set between 2 and 7 mg/L. Overexposure (Cmin > 7 mg/L) is associated with adverse effects. This study aims to explore therapeutic drug monitoring (TDM) across varying levels of renal clearance (CrCL), presence of CRRT, and strategy of administration (extended infusion versus continuous infusion).

**Methods**

This retrospective study was conducted in two Intensive Care Units of Niguarda Hospital from January 2023 to April 2024. We included all patients with Linezolid TDM analyzed on the third day of antibiotic therapy. Linezolid was administered either as 3 h extended or continuous infusion, at a daily dose of 1200 mg. TDM was measured by the central laboratory. The total clearance of Linezolid was calculated as dose (mg/day) divided by plasmatic concentration (mg/L). The area under the curve (AUC) was expressed in mg*h/L. Data on measured creatinine clearance (CrCL) were collected. Patients were stratified into four groups based on measured CrCL, with a separate group for CRRT patients. An additional analysis was conducted based on the infusion strategy in patients with or without CRRT. Continuous variables were compared using Wilcoxon signed rank test while categorical variables were compared using the Chi-square test. Informed consent was obtained.

**Results**

A total of 39 patients receiving Linezolid were included. According to renal function (CrCL) stratification, 5 patients were assigned to group 1 (< 20 ml/min), 6 to group 2 (21–50 ml/min), 5 to group 3 (51–70 ml/min), 5 to group 4 (71–130 ml/min), 2 to group 5 (> 130 ml/min), and 16 to CRRT group. Groups were comparable according to daily dose and BMI. Linezolid trough levels correlate with renal clearance, with CRRT-group (mean dialytic dose 34 ± 6 ml/Kg/h) drug clearance was comparable to the groups of patients with CLCr > 20 ml/min. Mean Linezolid TDM trough levels were 18 mg/L for group 1 (< 20 ml/min), 9,4 mg/L for group 2 (21–50 ml/min), 6,48 mg/L for CRRT-group, 4,46 mg/L for group 3 (51–70 ml/min), 4,42 mg/L for group 4 (71–130 ml/min), and 1,7 mg/L for group 5 (> 130 ml/min). (Fig. 1) The analysis conducted based on strategy of infusion in patient without CRRT revealed significant increases in linezolid Cmin (p 0.041) in the continuous infusion group (median 10 mg/L, IQR 4–13) compared to extended infusion group (median 2 mg/L IQR 2–7). (Table 1) (Fig. 2) However, no significant differences were observed in CRRT group (Table 2).

**Conclusions**

Linezolid clearance seems to be significantly affected by renal function. Patients undergoing CRRT show Cmin within those observed in patients with renal function ranging from 20–50 ml/min to 51–70 ml/min. Continuous infusion appears to reach higher Cmin compared to extended infusion. Further studies are recommended to confirm these findings.

**Fig. 1:**
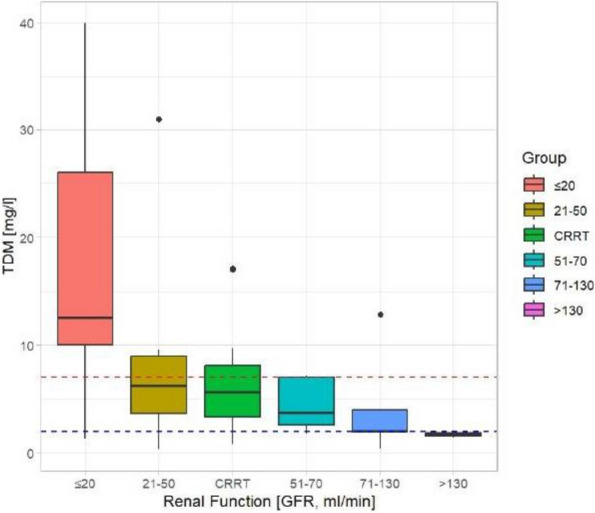
**(abstract A121).** TDM of linezolid at different scanarios of renal function and CRRT

**Fig. 2:**
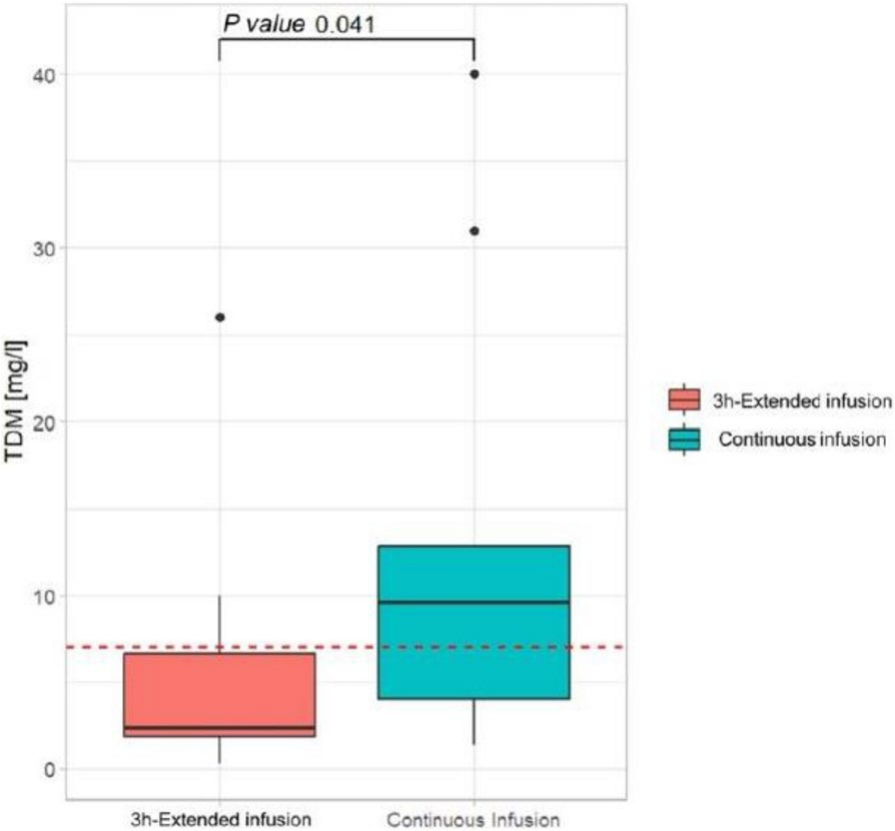
**(abstract A121).** Correlation between TDM levels of linezolid strategy of infusion (3 h-extended infusion vs. continuous infusion)

**Table 1:** **(abstract A121).** Results of the analysis of the group without CRRT

**NO CRRT group**	**Overall**,	**Extended infusion (3 h)**, N = 14	**Continuous infusion**,	**p-value**
	N = 23		N = 9	
**TDMs** (mg/l)	4 (2, 10)	2 (2, 7)	10 (4, 13)	0.041
**Cltot** (L/h)	13 (5, 26)	22 (8, 27)	5 (4, 13)	0.041
**AUC** (mg * h/L)	96 (47, 235)	55 (44, 160)	230 (96, 307)	0.041
**BMI** (kg/m^2^)	25.39 (24.26, 27.72)	27.66 (24.75, 29.14)	24.49 (22.04, 25.77)	0.038

**Table 2:** **(abstract A121).** Results of the analysis of the group with CRRT

CRRT group	Overall,	Extended infusion (3 h),	Continuous infusion,	p-value
	N = 16	N = 11	N = 5	
**TDMs** (mg/l)	5.6 (3.3, 8.1)	4.3 (3.0, 7.5)	7.6 (6.6, 7.7)	0.4
**Cltot** (L/h)	9 (6, 16)	12 (7, 18)	7 (6, 8)	0.4
**AUC** (mg * h/L)	134 (80, 194)	103 (71, 179)	182 (158, 185)	0.4
**BMI** (kg/m^2^)	26 (25, 28)	26 (25, 28)	26 (25, 41)	0.9

### A122 Effects of antibiotic prophylaxis on early-onset pulmonary infections in comatose patients admitted to ICU: an observational retrospective study

#### R. Antolini^1^, E. Casarotta^1^, A. Raponi^2^, F. Violini^1^, A. Salvucci Salice^1^, E. Vitali^1^, F. Santoni^1^, C. Pacini^1^, G. Perini^1^, A. Donati^1^, A. Carsetti^1^

##### ^1^Department of Biomedical Sciences and Public Health, Università Politecnica delle Marche, Ancona, Italy; ^2^Emergency Medicine Residency Program, Università Politecnica delle Marche, Ancona, Italy

###### **Correspondence:** R. Antolini

*Journal of Anesthesia, Analgesia and Critical Care 2024*, **4(1):**A122

**Background**

Ventilator-associated pneumonia (VAP) represents a frequent cause of death in comatose patients admitted to the intensive care unit. In this population of patients, early-onset VAP occurring in the first 4 days may have an incidence of up to 70%. There are numerous risk factors, including the immunosuppression resulting from the sympathetic activation induced by the intracranial event, and the difficulty in applying the VAP prevention bundles in this population of patients. Other measures, such as prophylactic administration of antibiotics during endotracheal intubation, have been considered to prevent early-onset VAP. However, the role of antibiotic prophylaxis remains unclear. Therefore, this study aims to evaluate whether early administration of antibiotics in comatose patients admitted to the ICU could reduce the incidence of early-onset pulmonary infections.

**Materials and methods**

We conducted an observational retrospective single-center study, including all the comatose patients (consequent to traumatic brain injury, stroke, seizures, post-cardiac arrest) admitted to our ICU (Clinica di Anestesia e Rianimazione Generale, Respiratoria e del Trauma Maggiore, AOU delle Marche, Ancona) from January 2023 to December 2023. We collected demographic and clinical data from the electronic medical records and we calculated the SAPS II and the APACHE score at ICU admission. We also collected data regarding endotracheal intubation time, days of mechanical ventilation, initiation of antibiotic treatment, microbiological tests performed, microorganisms isolated, and length of ICU stay.

**Results**

We considered 123 patients. Demographic and clinical characteristics of the whole cohort of patients are presented in Table 1.

Of the 123 patients included, 96 (78%) received antibiotic prophylaxis from the first day of admission in the ICU. In the group of patients treated with antibiotics (Treated), 52 (54%) developed an early-onset pulmonary infection within the first 4 days from ICU admission, while in the group of patients untreated (Untreated) 13 (48%), p = 0,66. Comparing the demographic and clinical characteristics, we observed a significant difference in the median age between the two groups of patients (57 [35 – 72] age in the Treated vs 69 [60 – 82] age in Untreated, p < 0,01). Significantly different were also the SAPS II and APACHE scores at ICU admission (42 [31 – 51,5] points in the Treated vs 52 [39 – 58] points in Untreated, p = 0,01, and 14,5 [10 – 19] points in the Treated vs 20 [15,2 – 22] points in Untreated, p < 0,01, respectively). We observed no significant difference in the median days of mechanical ventilation or length of ICU stay between the two groups of patients, as well as any significant association between antibiotic treatment and the type of microorganism isolated.

**Conclusions**

The preliminary analysis of this study showed no significant association between antibiotic prophylaxis and early-onset pulmonary infection within the first 4 days from ICU admission in comatose patients. However, further analysis could be useful to identify any confounding factors.

**Table 1:** **(abstract A122).** Demographic and clinical characteristics of the whole cohort of patients

	**Patients (n = 123)**
Males, n (%)	85 (69,1)
Age, years	60 [38 – 74]
SAPS II score	44,5 ± 14,9
APACHE score	16 ± 6,5

Data are presented as mean ± standard deviation, median [interquartile range], absolute and relative frequencies

## Loco-regional anaesthesia

### A123 Perioperative dexmedetomidine intravenous infusion for prolonging plexus block analgesia

#### A. Alfieri^1^, V. Donatiello^1^, P. Bonagura^1^, E. Prisco^1^, V. Maffei^1^, I. Biancogiglio^1^, F. Coppolino^2^, P. Sansone^2^, M.C. Pace^2^, M.B. Passavanti^2^

##### ^1^AORN Antonio Cardarelli, Napoli, Italy; ^2^AOU Luigi Vanvitelli, Napoli, Italy

###### **Correspondence:** A. Alfieri

*Journal of Anesthesia, Analgesia and Critical Care 2024*, **4(1):**A123

**Background**

Opioids have long been used to manage pain, but their adverse effects and the opioid epidemic have highlighted the need for alternative pain management strategies. Locoregional anesthesia has emerged as an effective and opioid alternative pain management technique. However, it may not provide sufficient analgesia in all cases. This study evaluated the efficacy of intravenous (IV) dexmedetomidine in prolonging the analgesic duration of plexus blocks for patients underwent shoulder and knee surgery. [1].

**Methods**

This retrospective observational study conducted at Cardarelli Hospital in Naples analyzed data from patients who underwent shoulder or knee surgery and received plexus block with either IV dexmedetomidine infusion (0.6 mcg/kg/h, DexIV group) or IV midazolam sedation (Control group). Ninety-nine patients, aged > 18 years with ASA score < 3, with no chronic pharmacological treatment with benzodiazepines, opioids, or alpha-2 agonists for pre-existing conditions, and undergoing orthopedic surgery with peripheral nerve blocks were included, if receiving the same dose of local anesthetic. Primary endpoints: differences between the two groups in mean analgesic time, analgesic rescue-dose frequency, time to first rescue analgesic administration, total opioid dose in the postoperative period. Secondary endpoint: tolerability profiles of adjuvant drugs. Propensity scores were calculated to ensure group homogeneity, minimize selection bias, and improve analysis reliability. A sample size of 100 patients was determined for statistical significance.

**Results**

No significant differences were found in demographic characteristics, ASA classification, surgical procedure types or block types between the two groups. The mean analgesic time of the plexus block was 9.1 h for the DexIV group and 4.0 h for the control group (p < 0.001). Comparing the opioid-sparing effects in the two groups, Fisher's exact test revealed significant differences in postoperative analgesic demand for both single doses (p = 0.001) and repeated doses (p = 0.0003). Kaplan–Meier analysis demonstrated a higher probability of analgesic request in the control group compared to the dexmedetomidine group. The log-rank test confirmed a significant difference in analgesic request survival functions, favoring dexmedetomidine (p < 0.05). Both treatments showed similar safety profiles, except for a higher incidence of bradycardia associated with dexmedetomidine. Patients in the DexIV group showed a significantly prolonged duration of analgesia compared to those in the midazolam control group for both shoulder and knee surgical procedures. Additionally, DexIV patients exhibited reduced 24-h cumulative postoperative morphine consumption. There were no significant differences in hemodynamic variations, opioid-related side effects, postoperative neurologic symptoms, or patient satisfaction between the two groups. Written informed consensus was obtained for each involved patient.

**Conclusion**

Intravenous dexmedetomidine infusion prolonged the analgesic time of the plexus blocks compared to the control group.

No significant differences emerged in the occurrence of side effects in both groups.

**Reference**

1. Faraj W. Abdallah, Tim Dwyer, Vincent W. S. Chan, Ahtsham U. Niazi, Darrell J. Ogilvie-Harris, Stephanie Oldfield, Rajesh Patel, Justin Oh, Richard Brull; IV and Perineural Dexmedetomidine Similarly Prolong the Duration of Analgesia after Interscalene Brachial Plexus Block: A Randomized, Three-arm, Triple-masked, Placebo-controlled Trial. Anesthesiology 2016; 124:683–695

### A124 Peng block in elderly patients with hip fracture: less is more?

#### V. Donatiello^1^, A. Alfieri^1^, V. Maffei^1^, E. Prisco^1^, M.C. Mazza^1^, O. Volino^1^, F. Coppolino^2^, M.C. Pace^2^, P. Sansone^2^, M.B. Passavanti^2^

##### ^1^AORN Antonio Cardarelli, Napoli, Italy; ^2^AOU Luigi Vanvitelli, Napoli, Italy

###### **Correspondence:** M.C. Mazza

*Journal of Anesthesia, Analgesia and Critical Care 2024*, **4(1):**A124

**Background**

Hip fracture in the elderly is a debilitating condition. Treatment of postoperative pain reduces the incidence of complications(1), especially in high-risk patients (ASA III-V). The PENG block (PEricapsular Nerve Group) is an effective and safe ultrasound-guided regional analgesia technique, which provides analgesia of the hip joint capsule. The aim of our analysis is to evaluate the analgesic impact and safety of different concentrations of local anesthetic in PENG block in order to reduce the use of opioid in the postoperative period.

**Materials and Methods**

It is a prospective observational study still ongoing, data are preliminary. The 110 patients enrolled, undergoing orthopedic surgery, were divided based on the type of surgery (endoprosthesis or osteosynthesis). All patients received PENG block preoperatively with different concentration of local anesthetic (20 ml of ropivacaine 0.375% vs 20 ml of ropivacaine 0.25%).

The included patients all received the same dose of subarachnoid anesthesia (2 ml of ropivacaine 0.5%) and the same dose of analgesics in the first 24 h (ketorolac 30 mg + paracetamol 1 gr every 8 h)and morphine as a rescue dose of analgesic. The study received Campania 3 Ethics Committee approval and all patients signed written informed consent.

**Result**

A non-inferiority test was performed to compare the effectiveness of the two treatments in the context of requests for additional doses of analgesic postoperatively. The analysis revealed that the success rate of treatment with PENG at concentration 0.375 was 77.39%, while for treatment with PENG at concentration 0.25 it was 75.49% (Tab. 1). The estimated difference between success proportions was approximately 1.90 percentage points, with a 95% confidence interval of 0.36 to 3.44 percentage points. Since the confidence interval does not include zero and the difference exceeds the specified delta value (0.05), the null hypothesis of non-inferiority was rejected. Furthermore, the patients who required the rescue dose of morphine were 24%, with almost the same percentage in the two groups, 23% of the PENG 0.375 group and 25% of the PENG 0.25 group.

**Conclusion**

The results suggest that treatment with PENG 0.25% is not inferior to PENG 0.375% in terms of effectiveness in preventing requests for additional doses of opioid during postoperative period. These findings may have significant implications in clinical practice, offering effective therapeutic options and potentially reducing the risks associated with the use of higher concentrations of local anesthetic.

**Reference**

1. Gabriel RA, Swisher MW, Sztain JF, Furnish TJ, Ilfeld BM, Said ET. State of the art opioid-sparing strategies for post-operative pain in adult surgical patients. Expert Opion Pharmacother. 2019 Jun;20(8):949-961. 10.1080/14656566.2019.1583743. Epub 2019 Feb 27. PMID: 30810425

**Table 1:** **(abstract A124).** Demographic and results table

Colonna1	Overall	0,375	0,25	p-value
total	110	58	52	
age (mean)	83	82	84	0,5
male	51	28	22	
female	59	30	30	
bmi	25	24	25	0,4
endo	37	19	19	
gamma	73	39	33	
time	65	65	65	0,9
n morph	26	13	13	0,7

### A125 Multimodal analgesia with fascial blocks: esp block and tap block, their role

#### A. Tozzi^1^, E. Spasari^1^, F. Imperatore^2^, P. Diglio^2^, C. Iacovazzo^1^, D. Cirillo^1^, G. Ranieri^3^, A. Coviello^1^

##### ^1^Department of Neurosciences, Reproductive and Odontostomatological Sciences, University of Naples Federico II, Napoli, Italy; ^2^Unit of Anesthesia and Intensive Care, San Giovanni di Dio Hospital, Frattamaggiore, Italy; ^3^Ospedale Isola Tiberina, Gemelli, Roma, Italy

###### **Correspondence:** E. Spasari

*Journal of Anesthesia, Analgesia and Critical Care 2024*, **4(1):**A125

**Background**

Laparoscopic cholecystectomy (LC) is a common minimally invasive surgical procedure for conditions like cholecystitis and cholelithiasis. Despite its minimally invasive nature, patients often experience postoperative pain, both somatic and visceral, which remains a significant concern. Our study proposes the use of two regional anesthesia techniques, the erector spinae plane (ESP) block[1] and the transversus abdominis plane (TAP) block[2], to manage this pain effectively.

**Case report**

The study aimed to optimize analgesic protocols for patients undergoing LC. Eight cases were described, where both ESP and TAP blocks were utilized. Patients were carefully selected based on various factors, including BMI, anxiety levels, and comorbidities. Written informed consent was obtained from all the patients. All patients were admitted in block room. ESP block was performed with the patient seated, a scout ultrasound scan identified and mark the transverse process at T5 level. The skin was sterilized and a 20-gauge block needle was inserted under the posterior fascia of erector spinae making contact with the transverse process. A total of 20 mL of 0.25% ropivacaine plus dexamethasone 4 mg was injected each side. Surgery was performed under general anaesthesia using Propofol (2 mg/kg iv) for the induction, rocuronium (0.6 mg/kg iv), remifentanil (0.15 mcg/kg/minutes) titrated to effect, and desflurane with (MAC) 1 for maintenance. Then was executed bilateral TAP block with patient still sedated in the operating room. With a scout ultrasound scan we identified a virtual space between internal oblique muscle superficially and the deep transversus abdominis muscle. A total of 20 ml of 0.25% ropivacaine was injected each side. All patients received postoperative nausea and vomiting (PONV) prophylaxis according to APFEL score. Paracetamol 1 g was administered every 8 h after surgery. In the case of acute pain, patients received an analgesic rescue dose. If NRS > 4 ketorolac 30 mg was administered. Post-operative pain is investigated with NRS, administered to the patient at 0 h, 6 h, 12 h, 24 h and 48 h. We achieved significant analgesic effects with lower opioid consumption and pain scores in the postoperative period. None of the patients required analgesic rescues. The combination of ESP and TAP blocks offering improved pain management and reducing the risk of postoperative complications like nausea and vomiting. Indeed, 0% of the patients reported showed postoperative nausea and vomit.

Conclusions: Our multimodal analgesic technique (ESP plus TAP block) showed significant analgesic effect during Laparoscopic cholecystectomy (LC) both in intraoperative and postoperative period, with lower opioid consumption, lower pain scores in postoperative and fewer analgesic rescue requests. We hypothesised that the combination of preoperative ESP block and postoperative TAP block can be a useful alternative to other analgesic techniques in Laparoscopic cholecystectomy (LC).

Informed consent to publish had been obtained.

**References**

1. Forero M, The erector spinae Plane Block: a novel analgesic technique in thoracic neuropathic Pain. reg anesth Pain Med 2016;41:621–7.
2. Takimoto K., The effects of adding upper and lower subcostal transversus abdominis plane blocks to a lateral transversus abdominis plane block after laparoscopic cholecystectomy: A randomised, double-blind clinical trial. European Journal of Anaesthesiology. 2015;32(11):819–820.

### A126 The role of loco-regional anesthesia in major surgery: esp block and tap block

#### P. Diglio^1^, F. Imperatore^1^, M.S. Barone^2^, P. Buonanno^2^, L. Zazzaro^1^, A. D'Abrunzo^2^, I. Piccione^2^, A. Coviello^2^

##### ^1^Unit of Anesthesia and Intensive Care, San Giovanni di Dio Hospital, Frattamaggiore, Italy; ^2^Department of Neurosciences, Reproductive and Odontostomatological Sciences, University of Naples Federico II, Napoli, Italy

###### **Correspondence:** P. Diglio

*Journal of Anesthesia, Analgesia and Critical Care 2024*, **4(1):**A126

**Background**

For years, the gold standard for postoperative analgesia in abdominal surgery has been, despite the complexity of the technique and its contraindications, epidural analgesia [1]. In recent years, following the indications of the ERAS protocols, Peripheral Nerve Blocks (PNB) have acquired increasing importance[2]. The aim of our study is to evaluate the impact of a double Peripheral Nerve Block for the control of postoperative pain.

**Case Report**

We report a case in which ESP-Block and TAP-Block were successfully used for analgesia in Major Abdominal Laparotomic Surgery. The anesthetic management was explained to the patient and informed consent for the procedure was obtained. Before General Anesthesia the patient practices bilateral ESP-Block. The patient was placed in a sitting position and once the transverse process of the T8 vertebra was identified, using a high-frequency linear probe, a 21G needle was inserted. 20 mL of solution per side containing Ropivacaine 0.4%, Clonidine 75mcg and Dexamethasone 2 mg was administered. General anesthesia was subsequently induced with Propofol (titrated by weight), Sufentanil 10mcg, Rocuronium (titrated by weight) and maintained with Desflurane; before surgery, bilateral TAP-Block was performed with a subcostal approach. Using an aseptic technique, once the fascial plane between the internal oblique muscle and the transversus muscle has been identified, using a high-frequency linear probe, a 21G needle was inserted and 15-20 mL of solution was administered per side containing Ropivacaine 0.25%, Clonidine 75mcg and Dexamethasone 2 mg. The patient received PONV prophylaxis consisting of dexamethasone and 5HT3 antagonist, according to APFEL score, administered after induction of general anesthesia. For post-operative pain, a dose of rescue analgesic as needed was provided, consisting of Paracetamol 1000 mg for NRS 1–2, Ketoralac 30 mg for NRS 3–5, Oxycodone 5 mg for NRS > 5. Post-operative pain was investigated with the international numerical scale NRS, administered to the patient at 0 h, 6 h, 12 h, 24 h and 48 h. During the intervention conducted we achieved notable hemodynamic stability. No further administration of intraoperative opioids necessary. Upon awakening always excellent pain control (NRS < 2). Patient not used rescue analgesic. Other important events were the total absence of post-operative nausea and vomiting and the rapid recovery of walking.

**Conclusion**

This case report demonstrates how the use of double peripheral nerve block allows a notable reduction in opioid consumption, optimal pain control, notable stability of the intraoperative anesthetic plan and a notable reduction in the risk of PONV. The encouraging result of this case report recommends expanding the study sample.

Informed consent to publish had been obtained.

**References**

1. Jeong, Young Hyun et al. “Transverse abdominis plane block compared with patient-controlled epidural analgesia following abdominal surgery: a meta-analysis and trial sequential analysis.” Scientific reports vol. 12,1 20,606. 29 Nov. 2022, 10.1038/s41598-022-25073-w
2. De Cassai, Alessandro et al. “Erector spinae plane block as a rescue therapy for uncontrolled pain after laparotomic surgery: A report of two cases.” Saudi journal of anaesthesia vol. 13,1 (2019): 66–68. 10.4103/sja.SJA_449_18

### A127 Impact Of Perioperative Opioid Use On Postoperative Outcomes: A Comprehensive Study Of 1270 Hip Fracture Patients

#### A. Del piano^1^, G. Balato^1^, L. Mori^1^, G. Grasso^1^, F. Esposito^1^, G. Servillo^1^, L. Al husinat^1^

##### ^1^AOU Federico II, Napoli, Italy; ^2^AOU Federico II, Napoli, Italy; ^3^AOU Duilio Casola, Cagliari, Italy; ^4^AOU Federico II, Napoli, Italy; ^5^AOU Federico II, Napoli, Italy; ^6^AOU Federico II, Napoli, Italy; ^7^Yarmouk University, Irbid, Jordan

###### **Correspondence:** A. Del piano

*Journal of Anesthesia, Analgesia and Critical Care 2024*, **4(1):**A127

**Abstract**

**Background and aims**

Hip fractures are common in the elderly and lead to serious health issues. Effective pain management during this period is vital for recovery and preventing complications like deep vein thrombosis and muscle atrophy. While opioids are key for pain relief during the perioperative phase, they carry risks like respiratory depression, and constipation. In this study, we aim to investigate the impact of perioperative opioid use on postoperative outcomes in hip fracture repair patients.

**Methods**

This is a retrospective observational study analyzing data from 1,270 patients who underwent hip fracture surgery between 2019 and 2021. The study used anonymized patient data from hospital databases, focusing on demographic characteristics, perioperative opioid use, hospital stay, ICU readmission, thromboembolic complications, and revision surgeries. We employed descriptive statistics, independent samples t-test, and Chi-square Test to compare postoperative outcomes, particularly examining the impact of perioperative opioid use. A multivariate analysis was also conducted to adjust for various covariates and reduce bias. The study was approved by the Ethical Committee and adhered to confidentiality guidelines.

**Results**

Of the independent samples t-test revealed significantly less hospital stay duration in patients with perioperative opioid use (P < 0.05). Additionally, perioperative opioid use was independently associated with reduced hospital stay (coefficient estimate = -0.656 (95%CI -1.217 – -0.096, P = 0.022)). Moreover, patients with perioperative opioids also had significantly less frequent ICU readmissions within a month after surgery (p = 0.024). On the other hand, the chi-square tests did not show any significant difference between perioperative opioid use and thromboembolic complications (p = 0.759), death within a year after surgery (p = 0.089), or the need for revision surgery (p = 0.336). Having a cardiovascular disease is an independent risk factor for hospital readmission within a month (OR = 1. 509 95% CI 1.026–2.220, p = 0.037).

**Conclusion**

Overall, this study contributes to the growing body of literature on perioperative opioid use and its implications on surgical recovery, offering valuable insights for healthcare practitioners to optimize patient care and enhance surgical outcomes.

**Keywords:** Thromboembolic complications, ICU, opioids, hip fracture, DVT, PE.

### A128 Fascial block in breast surgery beyond the limits: a case of total mastectomy in an obese male

#### D.P. Anceschi ^1^, C. D'Errico ^2^, A. Coviello ^1^, T. Iovino ^2^, G. Zagaria ^3^, R. Di Donna^3^

##### ^1^A.U. P. Federico II, Napoli, Italy; ^2^A.O.R.N. A. Cardarelli, Napoli, Italy; ^3^A.O.U. L. Vanvitelli, Napoli, Italy

###### **Correspondence:** D.P. Anceschi

*Journal of Anesthesia, Analgesia and Critical Care 2024*, **4(1):**A128

There are a lot of discussions and publications on loco-regional anaesthesia techniques in female breast surgery. Male cases of breast cancer account for 0.5–1 per cent of the total number of such diseases. According to the most recent AIRTUM (Italian Association of Cancer Registries) data, 1.7 breast cancers per 100,000 men and 150 cases per 100,000 women are diagnosed each year.

We present the case of a 35-year-old male patient suffering from severe obesity: height 1.78 weight 170 kg (BMI 53.6 kg/m2). The intubation indixes were prognostically unfavourable, Mallampati IV, Thyromental gap < 5 cm, inter-incisor gap < 6 cm. We placed nasal cannulae with O2 flow 5 l/min during the procedure.

First of all we premedicated patient with Midazolam 0.05 mg/kg, then we performed a Deep Serratus plane block with a 150 mm 22 G needle along the middle axillary, after hydro-localization with 0.9% saline solution 1–2 ml and advancing the needle along the plane of the serratus injecting 30 ml of Ropivacaine 0.75% 225 mg + Decadron 4 mg. Then using a 50 mm needle we performed a parasternal block after hydro-localization with 0.9% saline solution 1 -2 ml and injection of Ropivacaine 0.75% 75 mg + Decadron 2 mg.

The patient was sedated with Propofol in TCI 3–4 mcg/ ml with BIS monitoring maintaining values between 50 -60 and spontaneous breathing. (Fig. 1).

Write consent was obtained by the patient.

We monitored vital parameters, which were kept constant throughout the operation.

Post-operative pain was assessed with the NRS scale on awakening and after every half hour for the first two hours, then at 6 12 and 24 h.

No additional doses of paracetamol 1 g were administered beyond the two every twelve hours already prescribed.

In conclusion, we can state that the band block is a valid anaesthesia option even in cases of severe obesity.

**Fig. 1:**
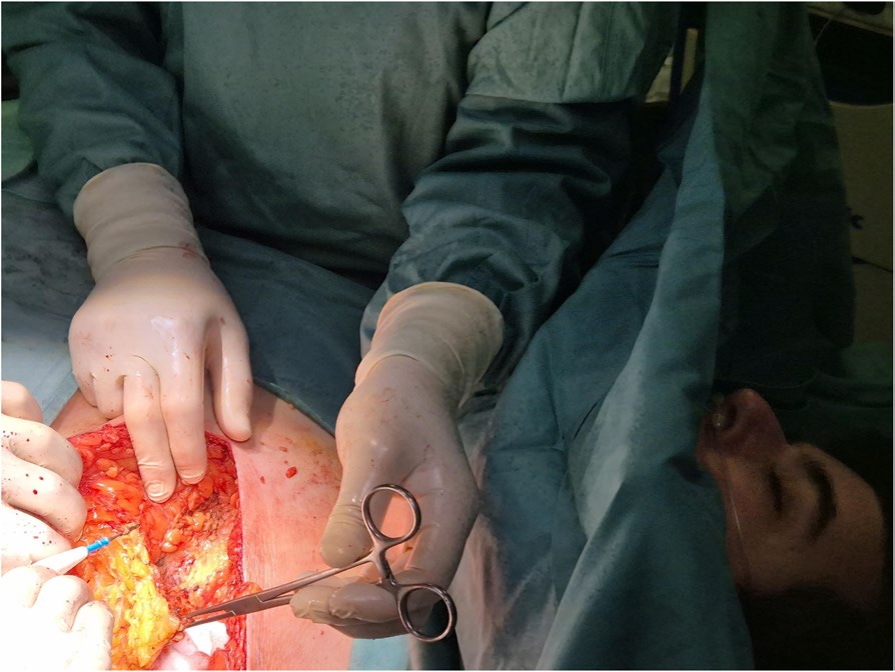
**(abstract A128).** See text for description

### A129 Multimodal or opioid based loco-regional analgesia? a pilot study in orthopedic surgery

#### D. Correnti, R. Beck, A. Discenza, M. Brattoli, L. Distaso, S. Tamburrano, A. Cotoia, L. Mirabella, G. Cinnella

##### Università degli studi di Foggia, Foggia, Italy

###### **Correspondence:** D. Correnti

*Journal of Anesthesia, Analgesia and Critical Care 2024*, **4(1):**A129

**Background**: Multimodal analgesia (MA), is increasingly used in orthopedic surgery for its advantage of combining loco-regional anesthesia (RA) with drugs from different classes to obtain a pre, intra and postoperative effect. An increasing amount of data in literature shows MA as more effective than traditional opioid-based analgesia, in terms of opioid-consumption and patient’s outcome. Aim of our study was to compare MA with standard opioid-based analgesia, in patient undergoing orthopedic surgery, in terms of reduction of postoperative pain and resumption of normal physiological functions and mental status.

**Methods**: Consecutive patients undergoing lower limbs elective orthopedic surgery were evaluated for inclusion. Inclusion criteria were: age > 65 years, inpatient stay of at least 48 h after surgery, ASA < III. Patients were randomly assigned to two groups: multimodal (GM) and Opioid-based (GO). GM received 0,3 mg/kg e.v. of Ketamine and peripheral nerve blocks as preemptive analgesia; infiltration of the surgical site with Lidocaine was performed in the perioperative period and post operative analgesia was obtained by continuous epidural analgesia with 240 mg of Levobupivacaine over 48 h. GO received 20 mcg of intrathecal Fentanyl, in the preoperative period, and 20 mg of Morphine in e.v. elastomeric pump over 48 h in the post operative period. Pre and post operative pain were recorded preoperatively (T0) and at 48 h (T48) by Visual Analogue Scale (VAS); psychophysical evaluation by Hamilton Anxiety Scale (HAS) was performed at T48.

**Results**: From September 2023 to February 2024, 46 patients were enrolled (n.23 per group). On T0 there were no differences in VAS between the two groups and all patients had a VAS > 6. On T48 pain was < 4 in every patient with a difference between GM, where median VAS was 2 and GO, where median VAS was 3 (p < 0,01). On T48 there are also differences between GM and GO in HAS, shown in Table I.

**Conclusions**: Our preliminary results, although confirming the effectiveness of opioid-based analgesia to control surgical pain, do highlight how MA is more effective in improving patient's outcome, in terms of pain reduction plus improvement of clinical and psychophysical conditions. Tough more data are needed, it is possible to speculate that the multimodal approach is able to control the multiple processes underlying the surgical pain, in a more targeted and specific way by acting at different levels and blocking its transmission at the level of the frontal cortex.

Informed consent was obtained.

**Table 1:** **(abstract A129).** See text for description

	**Multimodal (N. 23)**	**Opioid-based (N. 23)**	***P*-Value**
Preoperative VAS	≥ 6	≥ 6	
Postoperative VAS	2 ± 0,6	3 ± 0,6	(p < 0,01)
HAS	Calm N.20 (43,5%)	Calm N.3 (6,5%)	(p < 0,01)
	Anxious N.3 (6,5%)	Anxious N.6 (13,0%)	
	Agitated N.0 (0,0%)	Agitated N.14 (30,4%)	

### A130 Spinal anesthesia in the jack-knife prone position: a little used weapon

#### P. Coppola, A. Tortoriello, A. Galdieri, N.E. Caccavale, C. Nappo, A. Lanna, A. Caruso, R. Vincenti

##### UOC Anestesia e Rianimazione OO.RR. Area Nolana—Asl Napoli 3 Sud, Nola, Italy

###### **Correspondence:** P. Coppola

*Journal of Anesthesia, Analgesia and Critical Care 2024*, **4(1):**A130

**Background**

Minor anorectal diseases are rather common. Anorectal surgeries are notorious for being extremely stimolating and painful1. The preferred choice is spinal anesthesia in a sitting position with hyperbaric anesthetic, and after 10—15 min, in jack-knife prone position. It causes both motor and sensory blocks. However, it is associated with the possibility of respiratory compromise and hemodynamic instability2. The alternative technique is admistering spinal anethesia in jack-knife prone position.

**Case Report**

In “Santa Maria della Pietà” hospital, Nola, during three months six excisions of sacrococcygeal fistula were perfomed. All patients were male, 20–35 y.o., ASA I – II. In the opereting room, they were positioned directly in prone jack-knife position with a pillow under the abdomen, to achieve lumbar spine flexion and for optimal surgical exposure. Standard anethesia monitors (pulse oximetre, non-invasive blood pressure, 5-lead EKG) and nasal canula at 4 L/min were placed. If necessary, sedation with midazolam 2 mg. In asepsis, after local anethesia with lidocaine 2% 3–5 ml, subarachnoid anesthesia was perfomed with a spinal needle 27-gauge Withacre pencil-point at L4-L5 spinal level. Confirmation of the needle correct positioning occurred through aspiration of the cerebrospinal fluid. A hypobaric mix of approximately 1.5 ml containing: Ropivacaine 7,5 mg with sufentanil 3 µg, were injected in intrathecal space. Evaluation of the anesthetic plan was carried out through prick test. Surgical incision was made after approximately 10 min, the average duration of the operation was 30–40 min. In the intraoperative period, the patients, awake and calm, maintained the ability to move their lower limbs and bend their knees (Bromage scale 0–1); with a sensitive plan suitable for the ongoing intervention (Hollmen scale grade 4; NRS 0). At the end of the operation, the patient, with the support of the nursing team, positions himself in a supine position on the stretcher. Neither in the intraoperative period nor in the postoperative period were any adverse effects recorded that would prolong hospital stay.

**Conclusion**

Spinal anethesia in jack-knife prone position allows to obtain a selective hemianesthesia by blocking only dorsal sensory roots, this is due to the pharmacodynamics of the hypobaric local anesthetic in the intrathecal space. The advantages of this technique are: absence of motor blockage, greater hemodynamic stability, reduction of urinary retention, reduction of time spent in the operating block and post-operative hospital stay, resulting in greater comfort for the patient3. There are limited published studies and guidekines on this technique. It is hoped that the case series will build more interest in this tecnique and will establish futher studies and research.

Informed consent was obtained.

**References**

1. Li S, Coloma M, White PF, et al. Comparison of the Costs and Recovery Profiles of Three Anesthetic Techniques for Ambulatory Anorectal Surgery. Anesthesiol. 2000;93(5):1225–30.
2. Poon KS, Wu KC, et al. Hemodynamic Changes During Spinal Surgery in the Prone Position. Acta Anaesthesiol Taiwan. 2008;46(2):57–60.
3. Missouri S, Ray J, Geara E, Ayoub M, Grieco M. Administration of Spinal Anesthesia in the Jack-knife Prone Position for Anorectal Surgeries. J Anaesth Surg Res. 2023;3(2):178–83.

### A131 Perioperative patient blood management in lower limb orthopedic surgery

#### M. Comuzzi ^1,2^, A. Poles ^1,2^, M. Fabris ^1,2^, M. Camolese ^1,2^, F. Mazzon ^1,2^, M. Grandesso ^1,2^, G. Melchioretto ^1,2^, G. Tripi ^2^, T. Bove ^1,2^

##### ^1^Department of Medicine (DMED), University of Udine, Udine, Italy; ^2^Department of Anesthesia and Intensive Care Medicine, ASUFC University Hospital of Udine, Udine, Italy

###### **Correspondence:** M. Comuzzi

*Journal of Anesthesia, Analgesia and Critical Care 2024*, **4(1):**A131

**Introduction**

Proximal femur fracture is one of the urgent surgical indications that affects mostly the elderly population [1]. Up to 40% of the patients suffering from femur fracture are affected by anemia (hemoglobin concentration less than 13 g/dL in men and 12 g/dL in women) [2,3]. As regards surgical patients, the literature has shown that anemic patients have a greater risk of developing major complications such as cardiovascular events, infections and death compared to non-anemic population [4]. The trend of hemoglobin in the perioperative care of these patients has been widely studied and it has been seen that a low hemoglobin value upon admission to hospital is an independent risk factor for poor outcomes, while a higher postoperative hemoglobin correlates with a shorter hospitalization [5].

Our target was to evaluate whether preoperative hemoglobin concentration, even after the anemia correction, could still affect the length of post-operative hospitalization.

**Materials and methods**

Our retrospective analysis includes all patients that needed urgent orthopedic surgery for proximal femur fracture at the hospital “Santa Maria della Misericordia” (Udine, Italy) between 1st January 2023 and 31st May 2023. We enrolled patients older than 65 years with fractures referred at maximum of two body districts that underwent surgery in regional anesthesia; polytraumatic patients were excluded. We considered the hemoglobin’s values at the arrive in hospital, at the day of the surgery and at the date of the discharge. We also measured the length of hospitalization after the surgery and categorized the postoperative complications.

**Results**

We enrolled 156 patients: 42 males (26.9%) and 114 female (73.1%). The median age was 85.6 ± 6.5 years. The overall mortality at 30 days was 1.9% (3 patients) whereas the mean time to discharge was 14.9 ± 7.1 days after the surgery. The hemoglobin mean levels were 12.0 ± 1.77 at the time of admission, 11.3 ± 1.6 at the day of surgery and 10.4 ± 1.1 at the day of discharge (Fig. 1). Statistical analysis (Spearman’s rank correlation) found a relationship between the hemoglobin’s value at the day of surgery and the length of postoperative recovery (p-value 0.028).

**Conclusion**

In contrast with previous literature [4], our study found that perioperative hemoglobin levels do not appear to correlate with postoperative complications. Conversely, hemoglobin levels at hospital admission correlate with the length of postoperative hospitalization, as already seen by literature [5]. Further, we found a persisting risk of prolonged postoperative stay even when hemoglobin concentration levels were improved with proper transfusion strategies (Table 1). In conclusion, it would be recommendable to improve our strategies to increase the concentration of hemoglobin in the over65 patients to diminish the length of recovery and to enhance the poor outcomes bound to the anemia.

The consent was waived due to retrospective nature of the study.

**Fig. 1:**
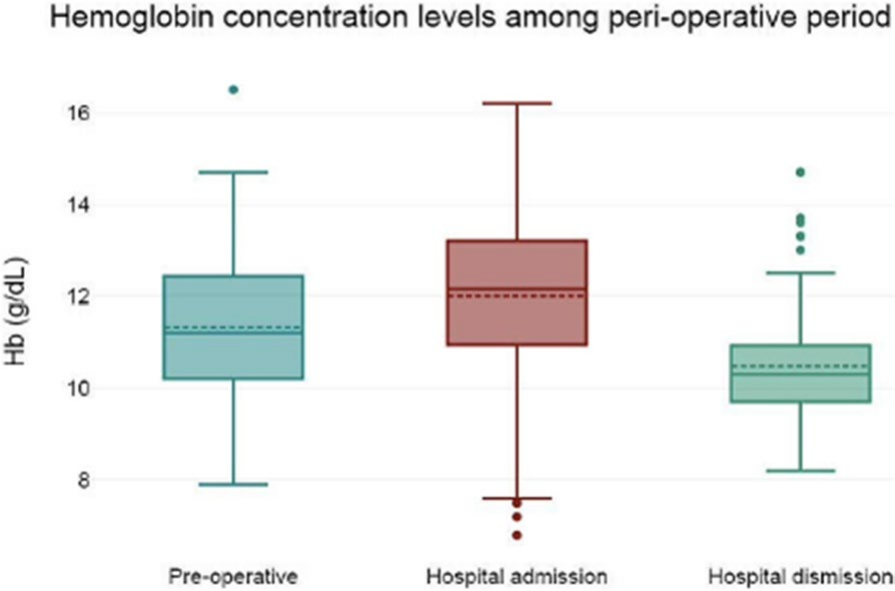
**(abstract A131).** Hemoglobin concentration levels among peri-operative period

**Table 1:** **(abstract A131).** Statistical analysis between major variables studied

Variables to correlate		*P*-value
*Preoperative Hb concentration*	*Postoperative recovery length*	**0.028**
*Hb levels at first medical contact (emergency department admission)*	*Postoperative recovery length*	**0.045**
*Minimal Hb concentration within 72 h after surgery*	*Postoperative recovery length*	0.289

### A132 Patient-friendly lumbar plexus block: combining ultrasound quick-look and traditional chinese medicine

#### L. Brugiaferri ^1^, M. Ciuffreda ^2^, E. Pisello ^2^, L. Angelelli ^2^, A. Grilli ^3^, J. Silvestri ^1^, D. Aucone ^4^, C. Piangatelli ^2^, D. Galante^5^

##### ^1^ Scuola di Specializzazione Anestesia, Rianimazione, Terapia intensiva e del dolore UNIVPM, Ancona, Italy; ^2^ UOC Anestesia Rianimazione Terapia del Dolore, Ospedale E. Profili, AST Ancona, Fabriano, Italy; ^3^ Scuola di Specializzazione Chirurgia Generale ULB, Bruxelles, Belgium; ^4^ UOC Ortopedia e Traumatologia, Fabriano, Italy; ^5^ UOC Anestesia Rianimazione Terapia del Dolore, Ospedale Tatarella, ASL Foggia, Cerignola, Italy

###### **Correspondence:** L. Brugiaferri

*Journal of Anesthesia, Analgesia and Critical Care 2024*, **4(1):**A132

**Background**

The lumbar plexus block is an advanced technique of regional anesthesia, not without risks, particularly in obese individuals or those with unfavorable anatomy. Given its usefulness in lower limb surgery, we have developed a new eco-assisted operative protocol based on measures derived from Traditional Chinese Medicine to increase its safety and reproducibility.

The aim of this preliminary prospective observational study was to evaluate the safety and reproducibility of our operative protocol.

**Materials and Methods**

Forty-seven patients aged between 16 and 86 years undergoing elective or emergency orthopedic surgery of the lower limbs with lumbar plexus block prior to general or subarachnoid anesthesia were evaluated. The needle puncture with electrical stimulation was performed at the transverse process of L5 (identified by ultrasound) with a lateral drift of 1.5 CUN (distance between the 2nd and 3rd paired fingers measured at the level of the distal interphalangeal joint of the non-dominant hand of the patient, converted to centimeters) from the interspinous line. The described landmark corresponds to point 26 of the bladder meridian, also used in acupuncture. After bone contact (depth from the skin estimated by ultrasound), the needle was oriented to pass the transverse process of L5 (cranially or laterally) advancing 2–3 cm deeper until confirmed by electrical stimulation.

**Results**

Our approach showed a success rate of 91.49% regardless of patient age and BMI. No complications such as intrathecal injection, renal damage, LAST, vascular injuries, or nerve injuries were detected.

**Conclusions**

Our approach based on simple ultrasound guidance and measures derived from Traditional Chinese Medicine allows for greater customization in the choice of landmarks, contrary to what happens with blind methods, ultimately resulting in satisfactory safety and reproducibility.

The patients have expressed their consent for the processing of their personal data for the above purposes.

**References**

1. Hogan MV, Grant RE, Lee L, Analgesia for total hip and knee arthroplasty: a review of lumbar plexus, femoral and sciatic nerve blocks. Jr. Am J Orthop (Belle Mead NJ). 2009 Aug;38(8):E129-33. PMID: 19809608 Review.
2. Saranteas T et al., A Lumbar Paravertebral Space Ultrasound Plexus Block Technique for Hip Fracture Surgery in Elderly. J Long Term Eff Med Implants. 2022;32(3): 65–71. PMID: 35993990.
3. Schultz-Stubner S et al., A new rule for femoral nerve blocks, Reg Anesth Pain Med. 2005 PMID: 16135352 Clinical Trial.
4. Roboubi B., What is cun?, Reg Anesth Pain Med. 2006. PMID: 16418038.

### A133 Sacral erector spinae plane block in a patient who underwent coccygectomy for chronic coccydynia, a case report

#### L.M. Bottazzo^1^, G. Cosenza^1^, G. Sepolvere^2^, F. Di Zazzo^2^, M. Della Valle^2^, L. Merola^2^, F. Coppolino^1^, V. Pota^1^, P. Sansone^1^, M.B. Passavanti^1^, C. Pace^1^

##### ^1^Department of Woman, Child, General and Specialistic Surgery, University of Campania L. Vanvitelli, Napoli, Italy; ^2^Department of Anesthesia and Cardiac Surgery Intensive Care Unit, San Michele Hospital, Maddaloni, Italy

###### **Correspondence:** L.M. Bottazzo

*Journal of Anesthesia, Analgesia and Critical Care 2024*, **4(1):**A133

**Background**

Coccydynia represents a painful syndrome affecting the coccyx and the surrounding area. Although coccydynia can affect individuals of all ages and both sexes, it occurs more frequently in women. The most common cause is trauma, the therapeutic approach is variable as well. The main symptom of the disorder is localized pain at the base of the spine, where the coccyx is located. Coccyx pain can be so intense as to make normal daily activities such as driving, or sitting difficult. In a small number of cases, only when all other conservative treatments fail, surgical removal of the coccyx (coccygectomy) may be recommended. Patients who suffer chronically from coccydynia use NSAIDs and weak opioids, making postoperative pain management in these patients potentially more complex. Therefore, we suggest a multimodal approach.

**Case Report**

A 30-year-old woman, ASA 2 classification, weighing 67 kg and measuring 168 cm in height (BMI 23.74), underwent coccygectomy surgery at our facility. Informed consent to publish had been obtained; our anesthetic management included intravenous general anesthesia and a Sacral (ESP) block [1] for postoperative pain control. General anesthesia was induced with propofol at a dosage of 8 mg/kg/h in continuous infusion, fentanyl 0.1 mg at induction, and remifentanil 0.1 mcg/kg/min in continuous infusion. The block was performed at the end of the surgery with the patient in the prone position. Under aspetic precautions, a high-frequency linear ultrasound transducer was placed midline just above the sacrum [2]. Using an in-plane technique from cranial to caudal direction, a 21G echogenic needle was inserted to the S4 crest. After negative aspiration, 10 mL of 0.375% levobupivacaine and 4 mg of dexamethasone were injected at the S4 median sacral crest level with aspiration every 5 mLs; the process was repeated at the S2 median sacral crest level (Fig. 1). Local anesthetic (LA) was deposited in the appropriate fascial plane between the erector spinae muscles and the S4 and S2 median sacral crests; the block was performed bilaterally with a volume of 20 ml of mixture per side. After the approximately 50-min surgery, the patient was transferred to the appropriate ward. In the first 24 h following the surgery, the patient required only one administration of Paracetamol 1 g and Ketorolac 30 mg; on the second day, the patient reported no pain but only mild discomfort.

**Conclusions**

The sacral ESP block is an alternative regional analgesic technique for this kind of procedures. The utilization of the Sacral ESP block has allowed us to significantly reduce the consumption of opioids and NSAIDs in the perioperative period (Table 1).

Informed consent was obtained.

**References**

1. Marrone F, Fusco P, Paventi S, Tomei M, Lolli S, Chironna E, Pullano C. Combined lumbar and sacral erector spinae plane (LS-ESP) block for hip fracture pain and surgery. Minerva Anestesiol. 2024 Apr 24. 10.23736/S0375-9393.24.18093-5. Epub ahead of print. PMID: 38656087.
2. Kukreja P, Deichmann P, Selph J P, et al. (April 14, 2020) Sacral Erector Spinae Plane Block for Gender Reassignment Surgery. Cureus 12(4): e7665. 10.7759/cureus.7665

**Fig. 1:**
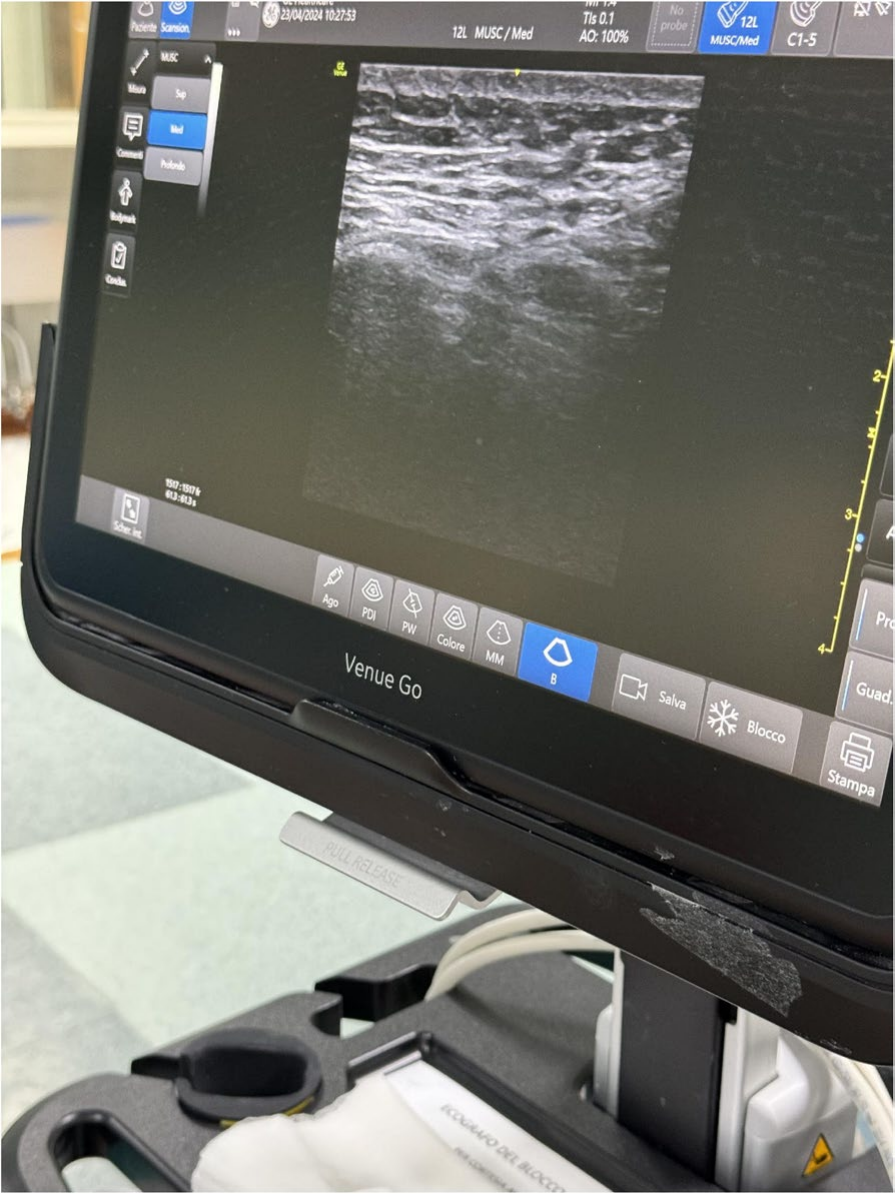
**(abstract A133).** See text for description

**Table 1:** **(abstract A133).** NRS Pain Scores

***NRS Pain Scores:***	
1st hour	3 (0–7)
12th hour	2 (0–6)
24th hour	2 (0–5)
48th hour	1 (0–3)
Total opioid consumption in 24 h (mg)	0.3 – 0.5

### A134 Burn patient and weaning, the role of local regional anesthesia: case report and literature review

#### M. Bianco ^1^, S. Badano ^2^, G. Perniciaro ^3^, N. Mereto^4^

##### ^1^ASL3 Genovese, Genova, Italy; ^2^ ASL3 Genovese, Genova, Italy; ^3^ ASL3 Genovese, Genova, Italy; ^4^ ASL3 Genovese, Genova, Italy

###### **Correspondence:** M. Bianco

*Journal of Anesthesia, Analgesia and Critical Care 2024*, **4(1):**A134

**Introduction**

Perioperative pain management in major burn patients remains a multidisciplinary challenge. Multimodal analgesia and locoregional anesthesia using fascial, nerve and central blocks have been shown to improve post-operative pain control, reduce the use of opioids and allow physiotherapy and patient weaning.

**Clinical case**

Informed consent and personal data processing were collected for the publication of the following case report.

31-year-old woman was admitted to the ED following a domestic accident while attempting to light a stove. The patient suffered extensive 2nd and 3rd degree burns of 30% TBSA involving the lower limbs, abdomen and face.

During hospitalization the patient underwent balneotherapy and surgical interventions with skin grafts. Pain was initially controlled by intravenous infusion of high-dose opioids. Weaning from pulmonary ventilation was managed by positioning an epidural catheter with continuous infusion of local anesthetic which allowed better pain control and favored an early start of active physiokinesitherapy. The patient was discharged after 101 days of hospitalization.

**Conclusions**

Locoregional techniques can be used in major burn victims to improve pain management, reduce the adverse effects of opioids and allow early active physiokinesitherapy.

### A135 Pericapsular nerve group block as opioid sparing strategy for total hip arthroplasty: a case report

#### A. Allieta ^1^, C. Montagnini ^1^, R. Vaschetto^2^

##### ^1^Ospedale degli Infermi, Ponderano, BI, Italy; ^2^Ospedale Maggiore della Carità, Università del Piemonte Orientale, Novara, Italy

###### **Correspondence:** A. Allieta

*Journal of Anesthesia, Analgesia and Critical Care 2024*, **4(1):**A135

**Background**

In recent years, regional anesthesia has evolved as a standard technique to achieve pain relief in patients undergoing total hip arthroplasty [1–3]. In this regard, the pericapsular nerve group (PENG) block represents a successful option for the management of acute hip pain [4]. Despite recent papers demonstrating the efficacy of PENG block in reducing opioid consumption [5–6], there is paucity of studies on the use of this technique as opioid sparing strategy in total hip arthroplasty pain management [7]. The aim of this study was to assess the role of PENG block in avoiding opioid consumption in patients undergoing total hip arthroplasty.

**Case report**

A 75-year-old female was admitted to our Emergency Department complaining about stabbing pain in her left hip and referring a recent fall. The hip X-rays showed a left transcervical femoral neck fracture (Fig. 1). On the day after hospital admission, the patient was scheduled for total hip replacement surgery. Since the patient had a full dabigatran dose the day before and considering coagulation disorders (enhanced partial thromboplastin time of 75 s), we decided to provide balanced general anesthesia. Guided by Bispectral index and by Train of four monitoring we induced the patient by administrating 1 mg of midazolam, 100 mcg of fentanyl and 120 of propofol followed by 40 mg of rocuronium bromide. Maintenance of anesthesia was assured by desflurane at a minimum alveolar concentration value of 1. As shown in Table 1, before performing surgery, we administered 1 g of acetaminophen and 30 mg of ketorolac. Then, we performed ultrasound-guided PENG block using a 22G × 50 mm SonoPlex needle injecting 20 ml of levobupivacaine 0.375% combined with 4 mg of dexamethasone (Fig. 2, 3, 4, 5). Before extubating the patient, 20 mg of nefopam, 4 mg of ondansetron and 200 mg of sugammadex were given intravenously, and eventually the surgeon infiltrated the wound with 20 ml of levobupivacaine 0.375%. We decided to ensure postoperative pain control with 1 g of acetaminophen every 8 h with a first rescue therapy of 30 mg of ketorolac and a second rescue therapy of 100 mg of tramadol in case of NRS > 3. Pain was assessed with the numeric rating scale (NRS). The patient NRS values at 1, 24, 48 and 72 h after emergence from anesthesia were 3, 5, 3 and 3, respectively. The patient needed 30 mg of ketorolac once daily, but she never asked for the administration of tramadol.

**Conclusion**

The uniqueness of this case is related to the use of a single-shot PENG block associated with standard dose of acetaminophen and ketorolac for providing pain relief in a total hip arthroplasty (Fig. 5). Additionally, in the present case, several factors suggest that, in such patients, pain control could be reached without any postoperative opioids and without the need for continuous peripheral nerve block. However, further studies are clearly needed to determine whether PENG block may represent the standard strategy for opioid sparing analgesia in hip-related procedures.

**Consent to publish**

Informed consent for publication was obtained.

**Fig. 1:**
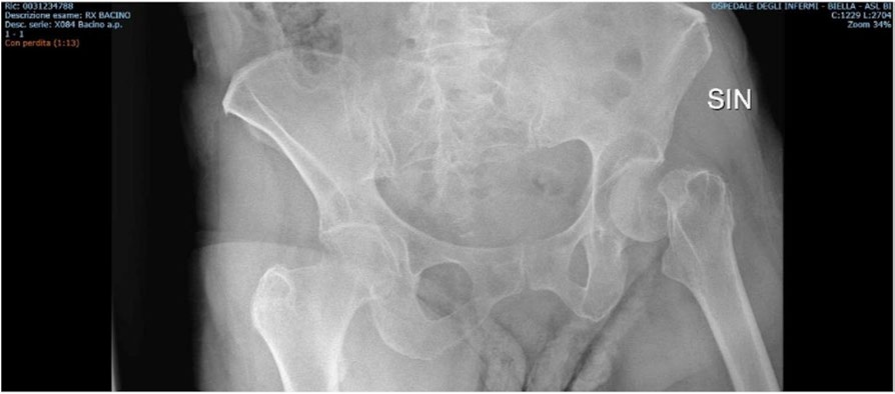
**(abstract A135).** Preoperative anterior–posterior hip X-ray showing left transcervical femoral neck fracture

**Fig. 2:**
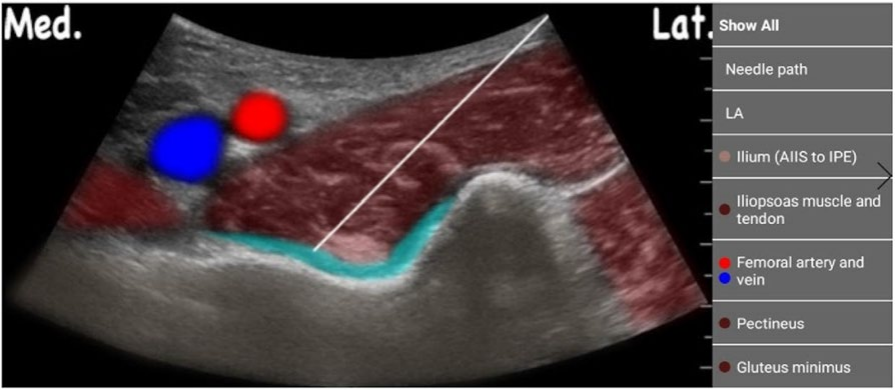
**(abstract A135).** PENG block landmark pattern. © Sumo Enterprise, AnSo, Anaesthesia Sonoanatomy. LA: local anesthetic; AIIS: anterior inferior iliac spine; IPE: iliopubic eminence

**Fig. 3:**
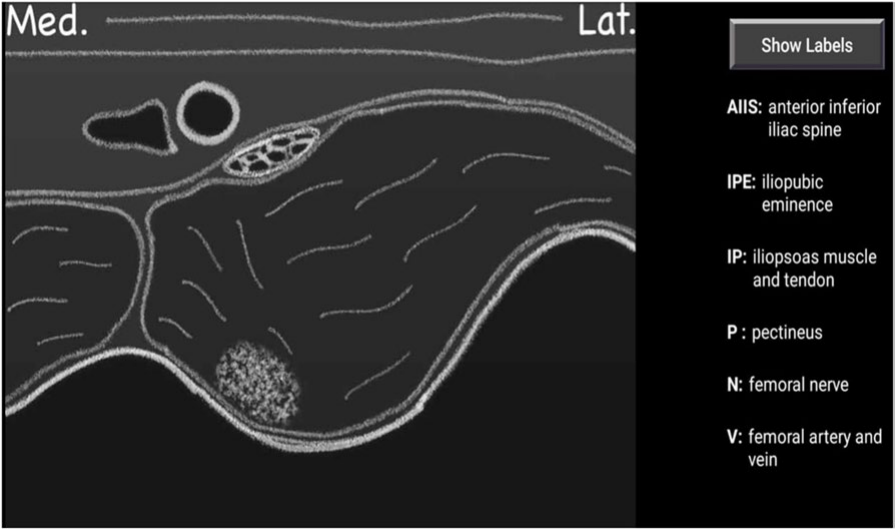
**(abstract A135).** PENG block sonoanatomy. © Sumo Enterprises, AnSo, Anaesthesia Sonoanatomy

**Fig. 4:**
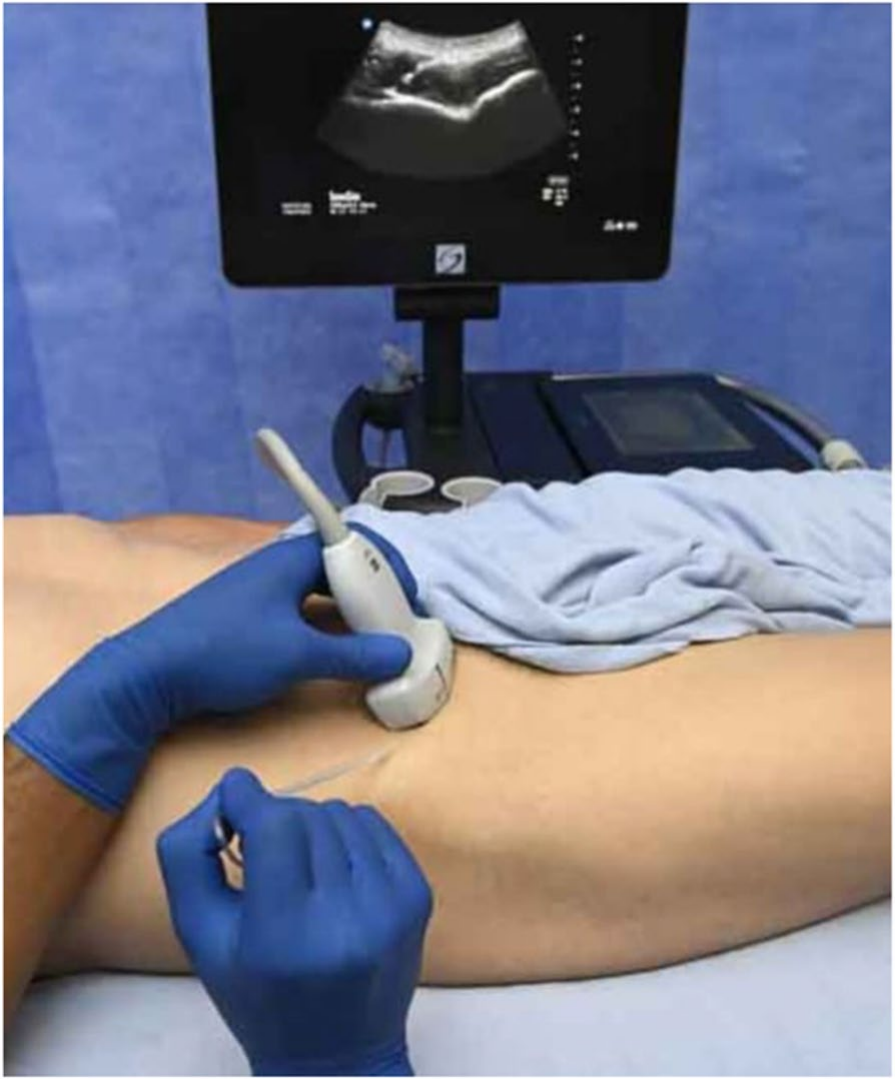
**(abstract A135).** PENG block ergonomics. © Sumo Enterprises, AnSo, Anaesthesia Sonoanatomy

**Fig. 5:**
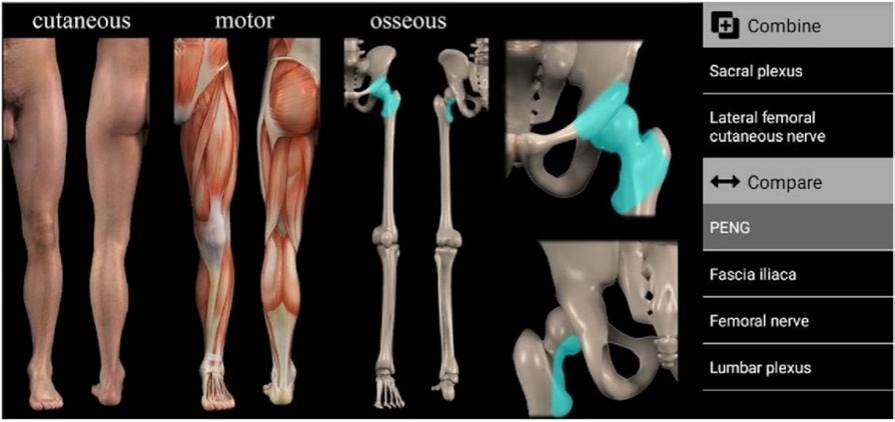
**(abstract A135).** PENG block innervation. © Sumo Enterprises, AnSo, Anaesthesia Sonoanatomy

**Table 1:** **(abstract A135).** See text for description

Opioid Sparing Analgesia Strategy	
	∙ Fentanyl 1,5 mcg/kg for induction of anesthesia
	∙ Pre-emptive analgesia:
**Intraoperative analgesia**	o 1 g acetaminophen
	o 30 mg ketorolac
	∙ Single-shot PENG block with 20 ml of levobupivacaine 0.375% combined with 4 mg of dexamethasone
	∙ Wound infiltration by the surgeon with 20 ml of levobupivacaine 0.375%
	∙ Nefopam 20 mg before emerging from anesthesia
	∙ 1 g acetaminophen every 8 h
**Postoperative**	∙ 30 mg ketorolac as first rescue therapy in case of NRS > 3: the patients needed a single dose every 24 h after surgery
**analgesia**	∙ **No need for second rescue therapy with tramadol 100 mg**

## Chronic pain

### A136 A new frontier in the application of minimally invasive techniques in pain therapy: first clinical experiences with the application of percutaneous microendoscopy for neuromodulation treatments

#### A. Violini, M. Pozzi, S. Pergher, G. Bellani

##### APSS, S. Chiara Hospital, Trento, Italy

###### **Correspondence:** A. Violini

*Journal of Anesthesia, Analgesia and Critical Care 2024*, **4(1):**A136

**Introduction**

Techniques for neuromodulation of peripheral nerves in chronic pain are long established.

Although ultrasound guidance allows the procedure to be performed with relative precision, it means that it is impossible to visualise the nerve except indirectly.

The direct visualisation of the nerve structures to be treated by iapplication of radiofrequencies for neuromodulation (1) or thermolesion (2) in the case of persistent or chronic pain has been described in the literature using small trocar-like cannulas into which an optical fibre is inserted (3).

Today, it is possible to use a microscopic optical fibre inserted inside a cannula with a diameter ranging from 20 to 22G, allowing minimally invasive procedures to be performed on nerve or endocavitary structures under direct vision.

**Methods and results**

The following is a description of three cases of patients suffering from chronic nociceptive or neuropathic pain treated with pulsed radiofrequency neuromodulation (T = 42°, V = 60, 480 doses) in minimally invasive percutaneous microendoscopy, after signing informed consent.

Case 1: Male patient, 57 years old, suffering from lumbar vertebral facet syndrome L3-L4 sx and L4-L5 dx. The treatment was performed under fluoroscopic guidance and microendoscopy (Fig. 1).

Efficacy at 3 months was immediate and long-lasting with complete pain relief for the patient (reduction of NRSm from 7 to 0).

Case 2: Male patient, 76 years old, suffering from chronic intractable neuropathic pain at the level of the left geniculate nerves, subjected to neuromodulation with radiofrequency with ultrasound technique combined with the microendoscopic technique. The patient reports complete pain relief after 3 months and a marked functional improvement (70% reduction in ODI score).

Case 3: Female patient, 68 years old, suffering from lumbar spinal facet syndrome in the setting of PSPS, underwent radiofrequency treatment of the L3-L4 right and left lumbar facets under fluoroscopic guidance and microendoscopy. The patient reported a benefit on low back pain with a 60% reduction in NRSm at 3 months.

**Discussion**

Visualisation of structures makes it possible to avoid injury or bleeding and to reduce the patient's clinical risk, increasing the safety and precision.

**Conclusions**

Further studies and clinical experience will certainly be needed to support this, but we believe that this technology can now open up the possibility of hitherto impossible applications in various fields.

**Consent to publish**

Informed consent for publication was obtained.

**References**

1. M. J. Teixeira, F. Freitas de Almeida, Sifuentes Almeira de Oliveira, E.T. Fonoff: Microendoscopic stereotactic-guided percutaneous radiofrequency trigeminal nucleotractotomy. J Neurosurg. 2012 Feb;116(2):331–5
2. E. T. Fonoff, W. C. Contreras Lopez, Y. Sifuentes Almeira de Oliveira, M. J. Texeira: Microendoscopy-guided percutaneous cordotomy for intractable pain: case series of 24 patients. J Neurosurg.2016 Feb;124(2):389–96.
3. J. Moon, H.S.Park, J.Y. Kim, K. S. Lee, S. Jeon, D. Lee, S. J. Bai, N. J. Kim: Analgesic Efficacies of Intraoperative Pectoralis Nerve II Block under Direct Vision in Patients Undergoing Robotic Nipple-Sparing Mastectomy with Immediate Breast Reconstruction: A Prospective, Randomized Controlled Study. J Pers Med. 2022 Aug; 12(8): 1309.

**Fig. 1:**
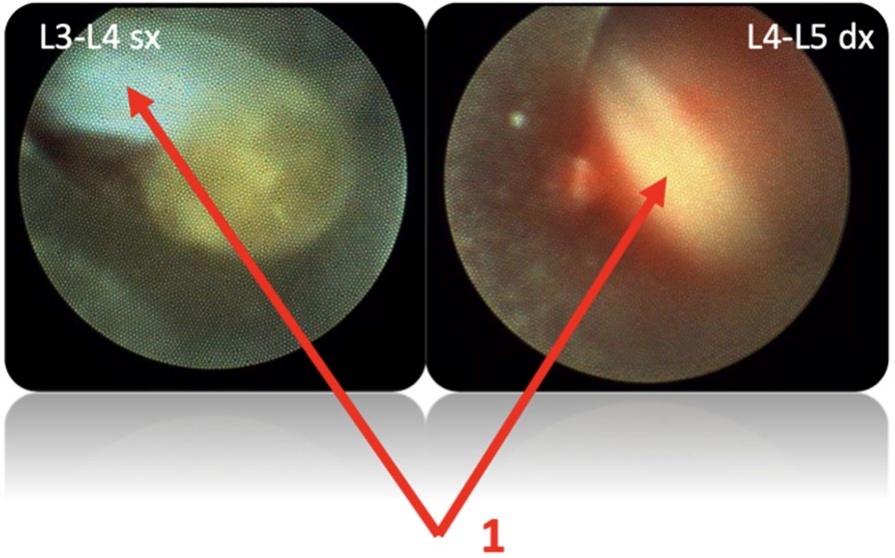
**(abstract A136).** Direct visualisation of the medial branch of the spinal nerve (1) at the level of the lumbar vertebral facets using microendoscopy

### A137 Implantation of Mesenchymal Stem Cells (MSCS) for the treatment of knee chondropathies. retrospective results of a multi-centric study

#### F. Saturno^1^, G. Monaco^1^, A. Salerno^3^, T. Alfieri^2^, M.C. Lombardi^1^, P. Guadagno ^3^, E. Cianciola^1^

##### ^1^ASL Salerno, Sapri, Italy; ^2^Scuola di Medicina e Chirurgia Università degli studi Magna Graecia, 88,100 Catanzaro., Catanzaro, Italy; ^3^ Biologa specializzanda in Patologia clinica e Biochimica clinica., Napoli, Italy

###### **Correspondence:** F. Saturno

*Journal of Anesthesia, Analgesia and Critical Care 2024*, **4(1):**A137

**Background**

Osteoarthritis (OA) is the most common joint pathology and is responsible, in the elderly, for a higher number of cases of disability than those caused by any other clinical condition. It is estimated that over 50% of the population over the age of 60 is affected by OA. Currently, OA is no longer framed as a pure degenerative process linked only to biomechanical alterations and/or aging, but rather as a pathological expression of wear, inflammation and immunological imbalance of the joint. Early diagnosis and treatment are crucial for the management of the disease. With the growing diffusion of MRI, in the coming years we will certainly see an increasing number of diagnoses, but also early treatment of the pathology itself. The therapy is based on oral NSAIDs (burdened by numerous side effects), intra-articular infiltrations of high molecular weight cortisone, infiltrations of high molecular weight hyaluronic acid and the implantation of mesenchymal stem cells with sampling from bone marrow or fat.

**Methods**

We retrospectively analyzed our series from 10.1.2023 to 3.1.2024 including 46 patients with an average age (53 years), males 20 and females 16. Patients are previously treated with three intra-articular infiltrations with local anesthetic and triamcinolone dep. 40 mg, weekly, to reduce inflammation of the joint. We have collected specific informed consent.

**Results**

No patient presented post-operative side effects such as intense pain, edema, surgical site infections, bruising at the sampling site, however patients report a feeling of heaviness in the joint for approximately 15 days.

The I.K.D.C. questionnaire was used. and the V.A.S. scale for the evaluation of pain while for the clinical examination an MRI was also used as an instrumental examination. Overall, 11 patients (24%) had a pain reduction < 50% while the remaining 35 patients (76%) had a pain reduction > 50%.

**Conclusions**

The outcomes of RESULTS OF A MULTI-CENTRIC STUDY would suggest encouraging results. However, the data in the literature are still lacking both in terms of efficacy and safety.

The main concern is the lack of long-term results.

### A138 Psychopathology and personality in chronic pain: the P3 study

#### S. Pupo ^1^, C. Barbi ^2^, S. Sivelli ^1^, E. Manferdini ^1^, G. Musetti ^1^, L. Pelizza^3^

##### ^1^Azienda Ospedaliero-Universitaria di Parma, Parma, Italy; ^2^ Università degli studi di Modena, Modena, Italy; ^3^ Università degli Studi di Bologna, Bologna, Italy

###### **Correspondence:** S. Pupo

*Journal of Anesthesia, Analgesia and Critical Care 2024*, **4(1):**A138

**Background—**In 50's, Engel suggested that, although the painful sensation originated from physiopathological phenomena, the individual interpretation and description of pain were predominantly psychological phenomena. Indeed, he believed that physical pain provided a more acceptable way of expressing unacceptable emotions directed towards oneself or others, so defining a defense mechanism against the most intimate emotional suffering. Alternatively, the painful physical sensation could also help the patient continue to play the functional role of victim. Within this cultural framework, Engel reported high prevalence rates psychiatric diagnoses in patients with chronic pain (e.g., conversion disorder, depression, hypochondriasis, anxiety disorders and paranoia), supporting the hypothesis that pain is a complex phenomenon involving both physical and psychological dimensions. With the introduction of the biopsycho-social model in medicine, the interest in understanding the complex interactions of psychological factors in painful sensations grew further, also including personality traits. Most of empirical studies examining personological aspects of patients with chronic pain used an eminently psychological-dimensional research approach, separated from the current categorical nosography of mental disorder. In order to improve treatment outcome and to plan multidisciplinary intervention for chronic pain based on personalized and precision medicine, the research methodology on psychopathological and personality featrures in patients with chronic pain more recently used clinical instruments developed according to the categorical model of mental disorders (e.g., ICD-11, DSM-5). However, the evidence reported to date, particularly lacking in italy, is still scarce and contradictory, although reporting high prevalence of both Cluster B personological traits (borderline, histrionic and narcissistic) and avoidant traits. The aim of this abstract was to carefully describe the P3 study, a research program aimed at investigating psychopathology and personality traits of patients with chronic pain attending an Italian center for pain therapy.

**Materials and Methods—**The P3 study is an observational (cohort), prospective and non-pharmacological research aimed to examine the main psychopathological and personality syndromes in patients with chronic pain who attended the outpatient clinics of the center for pain therapy at the Parma Hospital. This was aimed to collect epidemiological data on central aspects of the psychological components of chronic painful sensations, in the context of a multi-disciplinary intervention for both physical and psychic characteristics of chronic pain. All participants completed an ad-hoc schedule for collecting sociodemographic and clinical data, the Brief Pain Inventory (BPI), the Millon Multiphasic Clinical Inventory-III Edition (MMCI-III), the Personality Inventory for DSM-5 personality disorders (PID-5), and the Toronto Alexithymia Scale-20 items (TAS-20).

**Results—**110 participants were recruited in the first six months. 17% of them showed a psychiatric comorbidity (especially anxious-depressive disorders) and 20% personality disorder (especially cluster B dysfunctional traits). Only 7 were retained in care within specialist mental health services.

**Conclusions—**It's crucial that clinicians pay attention to psychopathology of patients with chonic pain, in a treatment context of multi-disciplinary interventions focused on both physical and psychological characteristics of protracted painful sensations.

### A139 Psychiatric comorbidity in patients with chronic pain attending an italian center for pain therapy: the first 100 cases of the P3 study

#### S. Pupo ^1^, C. Barbi ^2^, G. Musetti ^1^, L. Pelizza^3^

##### ^1^Azienda Ospedaliero-Universitaria di Parma, Parma, Italy, ^2^ Università di Modena e Reggio Emilia, Modena, Italy, ^3^ Università di Bologna, Bologna, Italy

###### **Correspondence:** S. Pupo

*Journal of Anesthesia, Analgesia and Critical Care 2024*, **4(1):** A139

**Background**—In 1959, Engel reported high prevalence rates of psychiatric symptoms in patients with chronic pain, supporting the hypothesis that pain was a complex phenomenon involving both physical and psychological dimensions. Within the biopsychosocial model in medicine, the interest in understanding psychological factors of chronic pain also included personality traits. In order to plan multidisciplinary intervention programs for chronic pain based on personalized and precision medicine, the research methodology on psychopathological components recently used clinical tools developed according to the categorical model of mental disorders (i.e., DSM-5). However, the evidence reported to date, decidedly lacking in Italy, is still poor and conflicting, although potentially encouraging personalized (patient-tailored) multi-professional intervention programs for chronic pain also supporting its psychological components in order to overall improve treatment outcomes. The aim of this investigation was to examine the prevalence of psychiatric comorbidity in the first 100 patients with attending the center of pain therapy Center of the Parma Hospital, as part of the P3 research study.

**Materials and Methods—**All 100 participants (aged 18–60 years) completed an ad-hoc schedule for collected sociodemographic and clinical data, the Brief Pain Inventory (BPI) and the Millon Multiphasic Clinical Inventory-III Edition (MMCI-III). Data were first analyzed using descriptive statistics methods. Any statistically significant difference in terms of clinical and psychopathological parameters between participants with and without psychiatric comorbidity were then examined, using the Chi-square test or the Mann–Whitney test (where appropriate).

**Results—**17 participants (17%) showed psychiatric comorbidity (especially anxiety-depressive disorders). Of them, only 7 were retained in care within specialist mental health services. This prevalence reached the 25% when considering the use of psychotropic drugs (especially benzodiazepines). Compared to patients without psychiatric comorbidity, those with psychiatric comorbidity had higher prevalence of chronic widespread pain (especially fibromyalgia) and fixed-dose analgesic pharmacotherapy (especially NSAIDs). Furthermore, they showed higher scores in BPI subscales relating to the interference of pain on emotional components (especially mood, social relationships and hedonic tone). In a not insignificant percentage of patients without psychiatric comorbidity (20%), significant scores were observed in MCMI-III subscales relating to anxiety, somatoform disorder, dysthymic disorder, and major depression.

**Conclusions—**A significant proportion (1 in 5) of patients with chronic pain had an established psychiatric comorbidity (of them, more than half without specialist retention in care). This prevalence reached the 25% when considering the prescription of psychotropic drugs (without analgesic indication) and the 34% (about 1 in 3) when considering the percentage of patients without previously ascertained psychiatric comorbidity who exceeded the MCMI-III cut-off scores for the presence of clinically prominent psychopathological syndromes. Attention to early detection and treatment of this subgroup of patients with chronic pain may overall improve clinical and functional outcome of pain treatment.

Informed consent was obtained.

### A140 Efficacy of therapeutic cannabis supplementation in fibromyalgic patients

#### N. Morlino, A.A. Carrideo, G. Zingarelli, N. Di Francesco, t. Di Foggia, A. Cotoia, M. Rauseo, L. Mirabella

##### Università degli studi di Foggia, Foggia, Italy

###### **Correspondence:** N. Morlino

*Journal of Anesthesia, Analgesia and Critical Care 2024*, **4(1):**A140

**Background**

Fibromyalgia syndrome (FMS) is a chronic disorder characterized by widespread body pain, commonly accompanied by stiffness, fatigue, sleep disturbances, cognitive impairments, and psychiatric signs. Optimum FMS management should be established in a multimodal structure: drug and non-drug treatments should be chosen in combination. Presently there is not clear-cut conclusion regarding the use of cannabinoids for pain management in FMS. The aim of this study is to examine the real efficacy of use of cannabis supplementation in reducing pain and improving quality of life.

**Material and methods**

All consecutive adults with diagnosis of FMS, refractory to any treatment, that received a cannabis prescription as herbal tea 300 mg/die up to 1000 mg/die based on patients needs, were retrospectively analysed from January 2018 to March 2024, at the Pain Department of the Policlinic of Foggia. Exclusion criteria were: cancer, pregnant and patients with a history of mental illness or drug addictions.

Patients were evaluated with a pain scale NRS and SF-12 score before prescription of cannabis (T0) and after six months (T1). On T1 patients were divided in two groups: Group 1, compliant patients with cannabis prescription; Group 2 patients who disagreed with cannabis therapy and continued only with standard therapy (opiates, paracetamol/gabapentinoids) and behavioral therapy. Other data were collected: age, gender.

Results

Overall a total of 70 patients were included: 30 patients in Group 1, 40 patients in Group 2 (Table 1).

On T0, PCS was < 31, MCS < 43 and NRS was > 7 in both groups.

On T1, SF12 e NRS improved in Group 1 (respectively p < 0,002 e p < 0,001) while in Group 2 data basically did not change (respectively p > 0,2 e p > 0,05). (Fig. 1).

**Conclusions**

In conclusion our study show that cannabis is effective as a supplement of therapy to reduce pain in patients with FMS. It would be interesting to evaluate the effects after longer.

**Consent to publish**

Informed consent for publication was obtained.

**Fig. 1:**
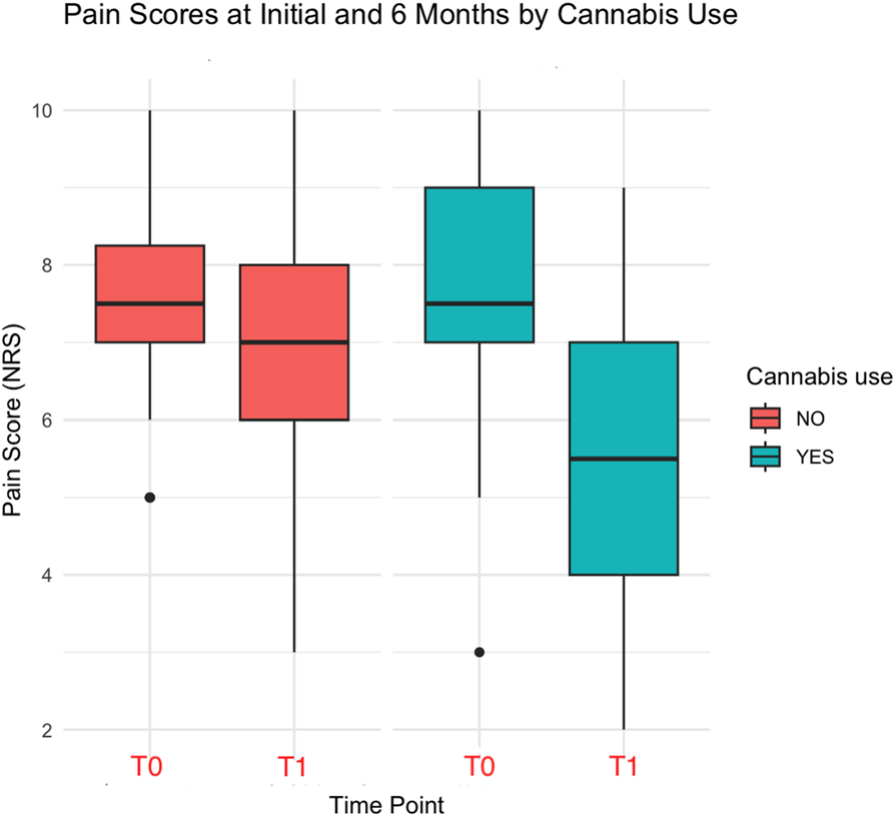
**(abstract A140).** See text for description

**Table 1:** **(abstract A140).** See text for description

	**Group 1**	**Group 2**	**p-value**
	**30**	**40**	
SEX M/F	3/27	3/27	0,71
Age (y)	55 ± 13	63 ± 10	0,45
SF12 T0	PCS* 29,8 ± 1,6	PCS 30,9 ± 1,9	0,53
	MCS*42,2 ± 1,3	MCS 32,36 ± 1,7	
SF12 T1	PCS 43,7 ± 1,2	PCS 39,0 ± 1,5	0,13
	MCS 42,8 ± 1,3	MCS 32,8 ± 1,8	

*PCS-12 physical component score, *MCS-12* mental component score

### A141 Peripheral nerve stimulation positioning the electrodes in the erector spinae fascial plane: case series and description of the technique

#### S. Angiolini ^1^, V. Gagliardi ^2^, G. Ferraro ^3^, G. Gagliardi^4^

##### ^1^ Università degli studi di Padova, Padova, Italy; ^2^ Università degli studi di Padova, Padova, Italy; ^3^ Aulss5 Polesana, ospedale Santa Maria della Misericordia di Rovigo, Rovigo, Italy; ^4^ Aulss 5 Polesana, ospedale Santa Maria della Misericordia di Rovigo, Rovigo, Italy

###### **Correspondence:** S. Angiolini

*Journal of Anesthesia, Analgesia and Critical Care 2024*, **4(1):**A141

**Background**

Electrical neuromodulation with either central and peripheral stimulation is an effective treatment for chronic pain, well tolerated and easily applicable.

Peripheral nerve stimulation consists of applying an electrical stimulus either on a peripheral nerve or on a group of nervous structures involved in the pathogenesis of pain.

In this framework, the fascia is a membranous anatomical structure richly innervated by sensory and autonomic nerve endings, nociceptors, and proprioceptors. Therefore, it plays a pivotal role in the genesis of persistent pain.

Patients affected by chronic pain have structural and functional modification in the fascial tissue: it could change its morphology, with a macroscopical rearrangement of its conformation associated to microscopical changes of the collagen fibers and their disposition.

Hence, the fascia could be a pain generator and could be involved in the mechanisms of central and peripheral sensitization to pain.

In particular, toraco-lumbar fascia is involved in the genesis of non-specific low back pain, whereas deep cervical fascia has been identified as a pain generator in cervicobrachial pain.

Focusing on the fascial structure, it has been the target of our therapeutic approach: in this study we have assessed the effect of nerve stimulation positioning the electrodes in the erector spinae fascial plane to treat chronic mixed pain.

**Materials and Methods**

Eight patients have undergone the treatment, three are males, five females. The age varie from 18 to 72 years. Our patients are affected by different types of pain, mainly identifiable as mixed pain: with neuropathic and musculoskeletal components.

The procedure has been performed in the operating room in local anesthesia, the patients were either awake or under conscious sedation. We have administered antibiotic prophylaxis before the treatment.

Technique of implantation: The patients have undergone percutaneous positioning of one or two electrodes in the erector spinae plane, identified and located with the ultrasound guidance. The electrodes were either quadripolar or octopolar. The vertebral level of the positioning of the electrode have been identified with fluoroscopy. At the end, a tonic stimulation evoking paresthesia has been administered, to verify the exact correspondence with the area of pain, so the correct positioning of the electrode (Fig. 1: neuropathic pain after surgical spine stabilization at C4-C5 level in a polytraumatized patient).

**Results**

The intensity of the pain has been evaluated using the NRS (Numerical Rating Scale) before the implantation, at 1 month, 3 months, 6 months, and 1 year after. Moreover, we have assessed the impact of the treatment on the quality of life using the Questionnaire QoL (EQ-5D-5L) before the implantation and after one year. (Table 1: NRS and EQ 5D 5L before the implantation and one year after).

**Conclusion**

From our case series, we can infer that the described technique allows a significant reduction in the intensity of pain, and a relevant improvement of the quality of life.

Positioning the electrodes in the erector spinae fascial plane has demonstrated to be effective in reducing symptoms in patients affected by chronic mixed pain, considering the fascia as an essential factor in its pathogenesis.

Informed consent was obtained.

**Fig. 1:**
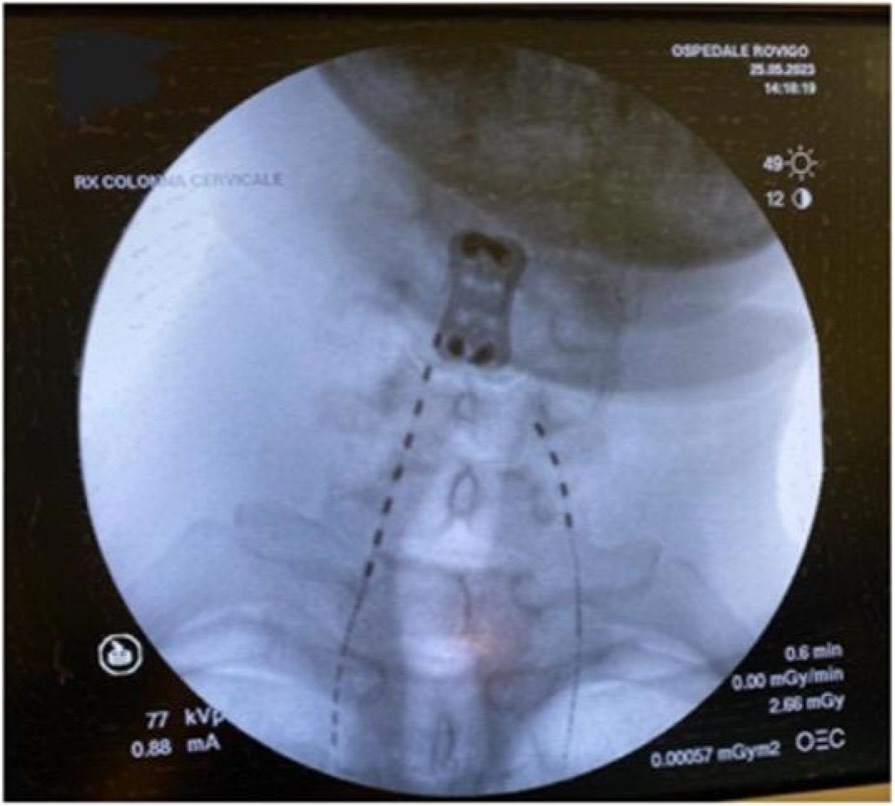
**(abstract A141).** Neuropathic pain after surgical spine stabilization at C4-C5 level in a polytraumatized patient

**Table 1:** **(abstract A141).** NRS and EQ 5D 5L before the implantation and one year after

Pathology	Sex	Age	NRS before implantation	NRS after 1 year	EQ 5D 5L before implantation	EQ 5D 5L after 1 year
Right brachial plexus avulsion in polytrauma	M	18	8	1	43,455	31,111
Persistent pain after spinal surgery	F	72	9	3	34,343	22,244
Cervical myelopathy after cervical discal herniation surgery	M	53	9	4	43,345	32,211
Neuropathic pain in surgical stabilisation of C4-C5 in polytrauma	M	52	8	1	42,233	21,121
Persistent pain after spinal surgery	F	70	9	3	44,353	22,122
Persistent pain after spinal surgery	F	69	10	4	54,433	32,214
Persistent pain after spinal surgery	F	71	10	4	43,333	33,323
Cervicobrachialgia in cervical arthrodesis	F	52	10	5	43,354	21,122

### A142 Use of platelet-rich plasma prp in the treatment of chronic pain from arthrosis. Our experience

#### A. Fruncillo ^1^, D. Giordano ^1^, L. Baccari ^1^, P. Russo ^1^, F. Barra ^1^, C. Chiumiento ^2^, F. Chiumiento^1^

##### ^1^ASL Salerno DEA Battipaglia-Eboli, Eboli, Italy; ^2^ Università degli Studi di Salerno- Scuola Spec.ne Anestesia e Rianimazione, Salerno, Italy

###### **Correspondence:** A. Fruncillo

*Journal of Anesthesia, Analgesia and Critical Care 2024*, **4(1):**A142

**Introduction**

Osteoarthritis is a degenerative joint disease that affects millions of people worldwide. Its prevalence increases with age and represents a major cause of disability and reduction in quality of life. Conventional treatments for osteoarthritis include pain medications, physical therapy and, in severe cases, replacement surgeries. However, these approaches are not always able to provide complete relief from symptoms and may have unwanted side effects. PRP represents an emerging therapy that aims to exploit the regenerative potential of the patient's platelets to promote cartilage tissue repair and relieve joint pain and stiffness. Platelets contain a rich array of growth factors that can stimulate cartilage cell proliferation, extracellular matrix synthesis, and reduction of inflammation.

**Objective**

To evaluate the effectiveness of PRP in the treatment of osteoarthritis of the knee, shoulder and hip.

**Materials and methods**

The study, carried out between January 2022 and April 2024, involved 380 patients with full-blown osteoarthritis of the knee (n = 205), shoulder (n = 97) or hip (n = 78). All patients received three intra-articular PRP injections every 21 days. The patients underwent a venous blood sample from which platelets were isolated using a cell separator. The plates were processed by the automatic, standardized and closed-loop Impact system. The PRP obtained was injected intra-articularly under ultrasound guidance. Patients were monitored for any adverse side effects. Symptoms were assessed using the VAS (Visual Analogue Scale) and the Western Ontario and McMaster Universities Arthritis Index (WOMAC) at 1, 6 and 12 months after treatment. Patients who did not show greater than 50% improvement at 12 months (n = 43) received a fourth PRP and radiofrequency injection.

**Results**

The results showed a significant improvement in symptoms in all patients except the 57 who required the fourth treatment associated with radiofrequency. Of these, 14 patients were found to be unresponsive to therapy and underwent a further PRP + radiofrequency session 6 months later. Of the 14 patients, 9 were non-responders and candidates for re-evaluation for alternative treatments including the surgical approach.

**Conclusions**

Treatment with PRP proved effective in improving the symptoms of knee, shoulder and hip osteoarthritis in over 80% of patients. The majority of patients experienced a significant improvement in symptoms, with a reduction in pain and stiffness and an increase in joint mobility. These results are consistent with those of previous clinical studies evaluating the use of PRP for osteoarthritis.

Informed consent was obtained.

**References**

1. Pereira D, Ramos E, Branco J. Osteoarthritis. Acta Med Port. (2015) 28:5477. 10.20344/amp.5477—DOI—PubMed
2. Sen R, Hurley JA. Osteoarthritis. StatPearls. Treasure Island, FL: StatPearls Publishing; (2023).
3. Wang T, He C. Pro-inflammatory cytokines: the link between obesity and osteoarthritis. Cytokine Growth Factor Rev. (2018) 44:38–50. 10.1016/j.cytogfr.2018.10.002—DOI—PubMed
4. Efficacy and safety of platelet-rich plasma injections for the treatment of osteoarthritis: a systematic review and meta-analysis of randomized controlled trials PMID: 37,441,691 PMCID: PMC10333515 10.3389/fmed.2023.1204144

### A143 Dorsal root ganglion stimulation for painful peripheral neuropathy in diabetic patients

#### M. De Luca, G. Liguori, S. De Santis, A. Bernardo, M.J. Sucre

##### Ospedale San Leonardo Castellammare Di Stabia Asl Napoli3sud, Castellammare Di Stabia, Italy

###### **Correspondence:** M. De Luca

*Journal of Anesthesia, Analgesia and Critical Care 2024*, **4(1):**A143

**Background**

Diabetic peripheral neuropathy (DPN) is one of the most frequent chronic complications of diabetes.

DPN affects more than 50% of diabetic patients, approximately 15–25% experience neuropathic pain.

Painful diabetic neuropathy is defined as a symmetric sensorimotor polyneuropathy, attributable to metabolic and microvascular alterations due to chronic exposure to hyperglycemia and other cardiovascular risk factors.

Only a third of patients achieve relief with conventional therapies.

Appropriate treatment of painful DPN is important because this pain determines a poor quality of life causing sleep disturbances, anxiety and depression.

The basic principle for managing DPN is to control hyperglycemia and other modifiable risk factors, but these treatments are often insufficient to prevent or improve symptoms of neuropathy.

Because there is no disease-modifying drug, it is important to treat pain.

Drugs used for DPN are gabapentinoids, serotonin-noreepinefin reuptake inhibitors, tricyclic antidepressants, alpha lipoic acid, topical capseiscin.

Spinal cord stimulation has been approved by the FDA for the treatment of painful diabetic neuropathy.

We thought that DRG stimulation could be an alternative or prior treatment to SCS (neuromedullary stimulation) to reduce pain and use of medications.

The dorsal root ganglion contains a collection of cell bodies of primary sensory neurons. DRG neurons are involved in the translation of pain to the CNS by acting as a filter for the propagation of afferent signals to the dorsal horn. DRG stimulation is a selective neuromodulation that can be used in numerous chronic neuropathic pain conditions and has been approved by the FDA (food and drug administration) for the treatment of CRPS complex regional syndrome.

The DRG-S allows precise targeting of nerve fibers innervating targeted painful regions without nonspecifically recruiting uninvolved dermatomes.

**Materials and methods**

We treated 5 diabetic patients with DPN with DRG-S who also simultaneously presented low back pain in the lumbosacral region.

Both low back and neuropathic lower extremity pain were not controlled by conventional medications: gabapentinoids and high-dose tapentalol. The NRS ranges between 7/9.

We performed neuromodulation with an electrocatheter inserted at sacral level and treated the L4 L5 S1 ganglion bilaterally.

The procedure was performed under fluoroscopic guidance and after suitable sensory and motor stimulation.

**Results**

All patients had a reduction in NRS of 7 to 1 for lumbar pain and from 9 to 2 for foot pain, as well as an improvement in the quality of life due to an improvement in walking, lumbar motility, paraesthesia of the lower limbs.

**Conclusion**

Given the results obtained, stimulation of the GRD represents a useful alternative and initial strategy for patients suffering from painful peripheral neuropathy associated with diabetes. This neuropathy determines alteration of proprioception that leads to alteration of posture and walking, it is often associated with lumbar pain which can be treated at the same time when associated with neuropathic pain.

Informed consent was obtained.

### A144 Esp block and ozone therapy: a new frontier in the treatment of chronic discogenic back pain

#### L. Brugiaferri ^1^, M. Ciuffreda ^2^, E. Pisello ^2^, A. Grilli ^3^, J. Silvestri ^1^, D. Aucone ^4^, C. Piangatelli ^2^, D. Galante^5^

##### ^1^ Scuola di Specializzazione Anestesia, Rianimazione, Terapia intensiva e del dolore UNIVPM, Ancona, Italy; ^2^ UOC Anestesia Rianimazione Terapia del Dolore, Ospedale E. Profili, AST Ancona, Fabriano, Italy; ^3^ Scuola di Specializzazione Chirurgia Generale ULB, Bruxelles, Belgium; ^4^ UOC Ortopedia e Traumatologia, Fabriano, Italy; ^5^ UOC Anestesia Rianimazione Terapia del Dolore, Ospedale Tatarella, ASL Foggia, Cerignola, Italy

###### **Correspondence:** L. Brugiaferri

*Journal of Anesthesia, Analgesia and Critical Care 2024*, **4(1):**A144

**Background**

Erector spinae plane (ESP) block is a fairly novel method of locoregional anesthesia, used mostly postoperatively in spine surgery. Although its mechanism of action has not been completely understood, its effectiveness has made its use increasingly frequent, even in pain therapy field.

Otherwise, Ozone Therapy has long played an important role in the treatment of chronic discogenic low back pain, albeit its use in locoregional procedures is still little explored.

In this case report we describe a patient with a chronic discogenic low back pain who underwent Ozone based ESP block.

**Case report**

Patient initially presented symptoms compatible with acute left lumbosciatica in L5-S1 area (NRS 8–9). MRI revealed a median-paramedian L4-L5 hernia and an extruded L5-S1 hernia in the left preforaminal area with a tendency to caudal migration.

Initial therapy involved the use of oral Methylprednisolone with slow improvement. Following yet another flare-up, steroid was replaced with slow-release Diclofenac with excellent response (NRS 0). Upon suspension, pain returns (NRS 4–5) with sciatica-like features, from the left glutes to the foot, exacerbated by movement and passive leg raise at maximal degrees (positive Lasegue Sign).

Since patient refused epidural injections and could not continue chronic treatment with NSAIDs, we decided to perform an Ozone based ESP block, thus exploiting both the anesthetic efficacy of the block (some studies suggest that the drug diffuses anteriorly into the paravertebral space, although an interfascial spread toward the posterior rami of spinal nerves is probably the main mechanism of action) and the anti-inflammatory, antioxidant, anti-edema and ultimately painkiller properties of Ozone.

An ultrasound-guided left L4-L5 ESP block was therefore performed using a convex probe and a 50 mm needle by injecting 20 ml of Ozone at 40 mcg. After the procedure, in the following 2 days pain initially decreased (NRS 2–3), then slowly increased (NRS 4–5) and then reduced further 2 week later (NRS 2–3).

ESP block was repeated 1 month after the first procedure and pain resolved completely (NRS 0).

**Conclusions**

Ultrasound guided ESP Block is a relatively new procedure and its use with Ozone in DLBP management had been recently described in literature.

It already appears clear, however, that combining an effective and versatile block such as ESP with Ozone therapy, a practice already in use for years and which also boasts a fair amount of literature, can be a winning choice in the management of chronic discogenic low back pain.

The patient has expressed his consent for the processing of his personal data for the above purposes.

**References**

1. Inklebarger J et al., Ultrasound Guided Erector Spinae Plane Block with Ozone and Corticosteroid for the Management of Discogenic Back Pain: A Case Report, International Journal of Medical Science and Clinical Invention, Vol 9 No 10 (2022), 6286–6295.
2. Sachdev D et al., Narrative Review: erector spinae block in spine surgery, J Spine Surg 2023.
3. Costa T et al., Ozone therapy for low back pain. A systematic review, Acta Reumatol Port 2018.

## General anaesthesia and perioperative medicine

### A145 High flow nasal cannula and diaphragmatic function after video-assisted thoracic surgery. A randomized, open-labeled, controlled trial

#### A. Fogagnolo ^1^, G. De Paoli ^1^, F. Dalla Corte ^2^, A. Andalò ^1^, G. Benetto ^1^, M. Riccardo ^1^, C.A. Volta ^1^, S. Spadaro^1^

##### ^1^Azienda Ospedaliero Universitaria di Ferrara, Ferrara, Italy; ^2^ IRCCS Humanitas, Rozzano, Italy

###### **Correspondence:** A. Fogagnolo

*Journal of Anesthesia, Analgesia and Critical Care 2024*, **4(1):**A145

**Background**

Data on high-flow nasal cannula (HFNC) after thoracic surgery are limited and the heterogeneity of the studies increases the uncertainty. We hypothesized that among patients undergoing video-assisted lobectomy, early postoperative support with HFNC may reduce the incidence of diaphragmatic dysfunction. We also compared the incidence of PPCs between HFNC and conventional oxygen therapy (COT).

**Methods**

This is a single center randomized trial including patients undergoing VATS lobectomy. Informed consent was taken from each patient before the surgery. After two hours from extubation, patients were randomized 1:1 to HFNC or COT; the intervention was maintained for at least 24 h. FiO2 was titrated to maintained SpO2 > 93%. In HFNC, oxygen flow was initially set at 60 L/min. In case of patient discomfort, oxygen flow was lowered to 50 L/min. In patients with SpO2 > 93% when breathing on room air, HFNC was still applied 24 h with FIO2 set at 24%. In COT group, low-oxygen flow was delivered through nasal cannula until a maximum of 4 L/m. In case of SpO2 < 94% with 4 L/m of oxygen, higher FIO2 were delivered by a Venturi face mask. In patients with SpO2 > 93% when breathing on room air, no supplemental respiratory support was given. After 24 h, ultrasound evaluation of diaphragmatic displacement (DD) and thickening fraction (TF%) was performed. The primary outcome was the incidence of diaphragmatic dysfunction defined as DD < 11 mm. The secondary outcome was the rate of PPCs, which were categorized as mild (hypoxemia, actelectasis, pleural effusion) or severe (pneumonia, new onset of respiratory failure).

**Results**

One-hundred-sixteen patients were randomized (58 in HFNC and 58 in COT group). Diaphragmatic dysfunction occurred in 38/116 (32%) of patients. Clinical characteristics are shown in Table 1. Density distribution of DD and TF% in the two groups are shown in Fig. 1.

Incidence of diaphragmatic dysfunction did not differ between groups (29% [17/58] in HFNC vs 36% [21/58]; = 0.55). PPCs occurred in 59% of the patients (54% in HFNC vs 64% in COT;p = 0.45). Incidence severe PPCs was 5% in HFNC and 19% in COT group; p = 0.04. HFNC did not reduced the occurrence of PPCs (HR 0.86,95% CI [0.54–1.38] p = 0.54). In a mixed model, the application of HFNC in patients with diaphragmatic dysfunction seems able to prevent the occurrence of PPCs (OR 0.16, [95% CI 0.02–0.83];p = 0.035). Incidence of severe PPCs was 14/116 (12%), being lower in HFNC patients (3/58 (5%) vs 11/58 (19%); p = 0.043; OR = 0.03 95% CI [0.61–0.89]). Accordingly, length of hospital stay was significantly lower in HFNC vs COT (4 [4–5] vs 5 [4–7]; p = 0.004).

**Discussion**

The main findings of our study are that early application of HFNC in after thoracoscopy lobectomy did not reduce the incidence of diaphragmatic dysfunction or postoperative pulmonary complication. Nonetheless, HFNC seems to reduce the incidence of PPCs in patients with postoperative diaphragmatic dysfunction; finally, patients in HFNC group had less rate of severe PPCs and less length of hospital stay.

**Fig. 1:**
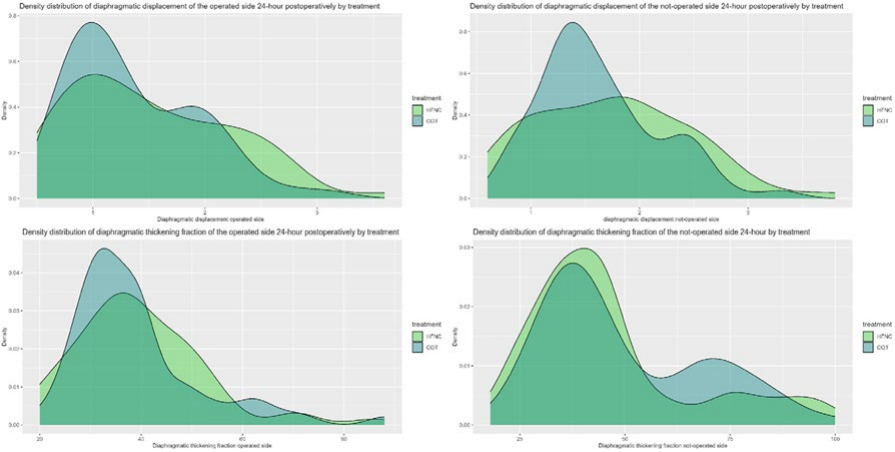
**(abstract A145).** See text for description

**Table 1:** **(abstract A145).** Preoperative characteristics of the patients.

Variables	All patients	HFNC	COT	p. value
	**N = 116**	**N = 58**	**N = 58**	
Sex, female %	58 (50%)	29 (50%)	29 (50%)	1.000
Height, cm	167 [160;171]	166 [160;173]	167 [160;171]	0.875
Weight, Kg	74.0 [64.0;82.0]	74.0 [64.2;81.8]	73.0 [63.0;82.0]	0.660
ASA score				0.309
2	21 (18.1%)	12 (20.7%)	9 (15.5%)	
3	93 (80.2%)	44 (75.9%)	49 (84.5%)	
4	2 (1.72%)	2 (3.45%)	0 (0.00%)	
ARISCAT score	49 [42;49]	49 [47;57.0]	49 [42;49]	0.001
***Comorbidities***				
Hypertension, n%	78 (67.2%)	46 (79.3%)	32 (55.2%)	0.010
Heart failure, n%	2 (1.72%)	1 (1.72%)	1 (1.72%)	1.000
Coronay disease, n %	12 (10.3%)	6 (10.3%)	6 (10.3%)	1.000
Atrial Fibrillation, n %	8 (6.90%)	2 (3.45%)	6 (10.3%)	0.272
COPD, n %	29 (25.0%)	18 (31.0%)	11 (19.0%)	0.198
Asthma, n %	6 (5.17%)	2 (3.45%)	4 (6.90%)	0.679
Steroids therapy, n %	15 (12.9%)	6 (10.3%)	9 (15.5%)	0.580
OSAS, n %	5 (4.31%)	4 (6.90%)	1 (1.72%)	0.364
History of smocking, n %	55 (47.4%)	31 (53.4%)	24 (41.4%)	0.28
Actual smocker, n %	41 (35.3%)	20 (34.5%)	21 (36.2%)	1.000
CPAP, n %	4 (3.45%)	2 (3.45%)	2 (3.45%)	1.000
CKD, n %	6 (5.17%)	3 (5.17%)	3 (5.17%)	1.000
Diabetes, n %	17 (14.7%)	11 (19.0%)	6 (10.3%)	0.294
***Preoperative spirometry***				
FVC, L	3.0 [2.2;3.5]	2.8 [2.1;3.7]	3.0 [2.5;3.4]	0.411
FEV1, L	2.1 [1.6;2.6]	2.0 [1.4;2.6]	2.2 [1.8;2.6]	0.190
FEV1, %	93 [80;109]	100 [84;114]	88 [80;103]	0.218
FEV1/FVC, %	80 [73;87]	80 [73;87]	78 [72;86]	0.623
DLCO, %	75 [62;84]	74 [64; 83]	77.5 [61;84]	0.834
***Preoperative Blood gas analysis***				
pH	7.40	7.40 [7.34;7.42]	7.40 [7.38;7.42]	0.352
	[7.37;7.42]			
PaO2, mmHg	88 [77;92]	88 [75;92]	88 [81;91]	0.799
PaCO2, mmHg	42 [39;45]	42 [38;44]	42 [40;44]	0.629
***Preoperative clinical parameters***				
Systolic pressure, mmHg	140 [130;150]	140 [130;150]	140 [130;150]	0.715
Dyastolic pressure, mmHg	80 [75;85]	80 [75;84]	80 [75;85]	0.272
Heart rate, bpm	75 [67;8]	74 [68;80]	75 [66;78]	0.439
Respiratory rate	15 [14;16]	15 [14;16]	15 [14;16]	0.979
SpO2 in room air	97 [96;98]	97 [96;98]	97 [96;98]	0.294

### A146 Effect of different peep strategies on reduction of atelectasis determined by lung ultrasound in patients undergoing robot-assisted radical prostatectomy: a prospective study at a single center

#### A. Lanotte, P. Raimondo

##### ASL Lecce, Lecce, Italy

###### **Correspondence:** A. Lanotte

*Journal of Anesthesia, Analgesia and Critical Care 2024*, **4(1):**A146

The pneumoperitoneum and the steep Trendelenburg position, in patients undergoing robot-assisted prostatectomy, are both elements that could worsen intraoperative respiratory mechanics and induce postoperative atelectasis. We investigated the effects of two different PEEP (positive end-expiratory pressure) in particular low PEEP (5 cmH2O) and high PEEP (10 cmH2O) on reduction of postoperative atelectasis, evaluated with the use of lung ultrasonography. After obtaining informed consent, 22 male patients undergoing robot-assisted prostatectomy were recruited and were randomly allocated into two groups (low PEEP or high PEEP). All patients underwent monitoring vital sign and depth of anesthesia using bispectral index (BIS), subarachnoid analgesia with 150 mcg of morphine and subsequent balanced general anesthesia. Restrictive fluid therapy with 4 ml/kg/h of crystalloids is applied. All patients were ventilated protectively with tidal volume of 6–8 ml/kg PBW (predicted body weight), FiO2 35–40%, I:E ratio 1:2 and respiratory rate adjusted in order to maintain EtCO2 between 33–40 mmHg. Ultrasound examination was performed on 12 sections of thorax and at three times: T0 ten minutes before oro-tracheal intubation, T1 at the end of surgery in mechanically ventilated patients, T2 fifteen minutes after extubation. For each section was measured a lung ultrasound score from 0 to 3 according to the number of B lines or the presence of subpleural consolidation. The aim of the study was the impact of two different PEEP levels on lung aeration determined by lung ultrasound score. An increase of the difference of LUS score at T2 between group L and group H means the development of atelectasis. According to the demographic analysis of the two groups it emerged that there are no differences regarding age, eight, BMI, prostate volume, duration of surgery and main comorbidities; only the smoking habit is more expressed in L group. The analysis of the trend of LUS scores shows that in both groups there is an increase in LUS going from T0 to T2 which is more marked in L group. Comparing the average LUS scores of the two groups shows how the average LUS score at T2 is significantly higher in group L. Only for the L group was observed an inverse correlation between P/F ratio and LUS score. High PEEP (10 CmH2O) during the pneumoperitoneum and steep Trendelenburg position significantly reduced postoperative atelectasis, evaluated using lung ultrasonography. However, the clinical significance should be evaluated by a larger clinical trial.

### A147 Postoperative continuous monitoring in surgical ward with wearable devices

#### M. Panizzi, V. Bellini, F. Bezzi, M. Mion, M. Bagnoli, E.G. Bignami

##### Anesthesiology, Intensive Care and Pain Medicine Division, Department of Medicine and Surgery, University of Parma, Parma, Italy

###### **Correspondence:** M. Bagnoli

*Journal of Anesthesia, Analgesia and Critical Care 2024*, **4(1):**A147

**Background**

Postoperative deterioration is often preceded by abnormalities in vital parameters [1, 2]. Nevertheless, limited resources generally preclude continuous monitoring in ICU/SICU for all patients. The development of new technologies recently allowed the introduction of wearable devices (WDs) and wireless data-transmission protocols such as LoRa: these tools are potentially capable of extending access possibilities to more intense monitoring regimes for a larger patient population, especially in general hospital wards [3]. The aim of this review was to investigate the state of the art of the use of WDs as remote early warning systems in the postoperative period.

**Materials and Methods**

This review was conducted according to PRISMA-ScR guidelines. PICO framework was used before the search to define the review protocol. A systematic literature research has been performed on PubMed, MeSH, MEDLINE, and Embase, considering a period between 2018 and February 2024. Both retrospective and prospective studies were eligible for inclusion and no automated tools were used.

**Results**

10 articles were included in the review, with a total of 11 different CE/FDA wearable devices used in the analyzed studies. Devices information and monitored parameters are summarized in (Fig. 1). In all the studies considered, devices were applied on surgery day upon patient arrival to recovery room or surgical ward. Mean monitoring duration was 6.7 days, ranging from 72 h to 14 days. Globally, both beyond-threshold alarms and trend analysis protocols were used to set up reactive or proactive intervention on patients, although in some studies the output was blinded to medical staff.

**Conclusions**

The use of WDs in clinical practice as an integral part of post-operative monitoring systems is feasible and safe, and soon its potential could be enhanced by the implementation of LoRa data transmission protocol and artificial intelligence. Assessing the trend in vital signs with such systems could help to identify post-operative complications earlier, shorten the length of hospital stay, reduce the likelihood of unplanned ICU/clinical admissions and, despite a high initial investment, reduce healthcare costs. However, further studies are needed to clarify the potential role of these devices in perioperative medicine.

**References**

1. Breteler MJM, KleinJan E, Numan L et al. Are current wireless monitoring systems capable of detecting adverse events in high-risk surgical patients? A descriptive study. Injury. 2020;51 Suppl 2:S97-S105.
2. Hillman KM, Bristow PJ, Chey T et al. Duration of life-threatening antecedents prior to intensive care admission. Intensive Care Med. 2002;28(11):1629–1634.
3. Churpek MM, Yuen TC, Park SY et al. Using electronic health record data to develop and validate a prediction model for adverse outcomes in the wards*. Crit Care Med. 2014;42(4):841–848.

**Fig. 1:**
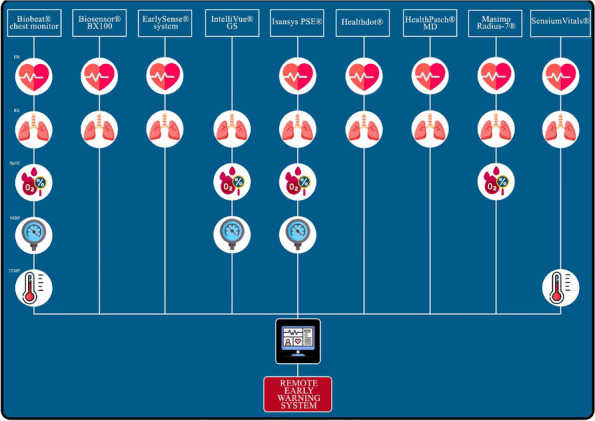
**(abstract A147).** Devices overwiev. IntelliVue GS = Guardian Solution; Isansys PSE = Patient Status Engine (3 devices system); HR = Heart Rate; RR = Respiratory Rate; NIBP = Non-Invasive Blood Pressure; Temp = Temperature; Icons credits: Freepik from Flaticon.com; Karyative from Flaticon.com; Flaticon.com.

### A148 Lung ultrasound score profile in obese patients undergoing laparoscopic-robotic surgery: a secondary analysis of peep lap study

#### F. Verdina ^1,2^, G. Furlan ^2^, D. Rosalba ^1,2^, L. Carrera ^2^, F. Santangelo ^2^, A. Rivolta ^2^, A. Guzzo ^2^, f. Vietti ^2^, a. Migliavacca ^2^, S. Gentilli ^2,3^, R. Romito ^4^, L. Portigliotti ^4^, D. Surico ^2,5^, M. Giana ^5^, A. Vigone ^5^, A. Volpe ^2,6^, M. Billia ^6^, R. Vaschetto ^1,2^, G. Cammarota ^2,7^

##### ^1^ Anestesia e Terapia Intensiva, Azienda Ospedaliero Universitaria Maggiore della Carità, Novara, Italy; ^2^ Dipartimento di Medicina Traslazionale, Università del Piemonte Orientale, Novara, Italy; ^3^ Clinica Chirurgica, Azienda Ospedaliero Universitaria Maggiore della Carità, Novara, Italy; ^4^ Chirurgia Generale 2 Azienda Ospedaliero Universitaria Maggiore della Carità, Novara, Italy; ^5^ Ginecologia e Ostetricia, Azienda Ospedaliero Universitaria Maggiore della Carità, Novara, Italy; ^6^ Urologia, Azienda Ospedaliero Universitaria Maggiore della Carità, Novara, Italy; ^7^ Azienda Ospedaliero Universitaria di Alessandria SS. Antonio e Biagio e Cesare Arrigo, Alessandria, Italy

###### **Correspondence:** D. Rosalba

*Journal of Anesthesia, Analgesia and Critical Care 2024*, **4(1):**A148

**Background**

Laparoscopic surgery and General Anesthesia are known to cause atelectasis. Additionally, elevated FiO2 levels, can exacerbate alveolar collapse. Positive end-expiratory pressure (PEEP) can be used to mitigate this risk. In obese patients the risk of atelectasis is even higher. While higher PEEP values may seem necessary in these patients to keep airways open and improve lung mechanics [1], the impact of elevated PEEP on postoperative respiratory complications remains unclear [2]. Furthermore, the optimization of PEEP levels is still under evaluation.

**Materials and Methods**

After informed consent, we recruited adult obese patients (BMI > 30 kg/m2) who underwent laparoscopic or robotic abdominal/pelvic surgery. Prior to anesthesia induction, a twelve-point Lung Ultrasound Score (LUSS) examination was conducted (T0). Volume-controlled ventilation was administered during surgery, with a tidal volume of 6 ml/kg. Respiratory rate and FiO2 were adjusted to maintain normocapnia and normal blood oxygenation levels. PEEP was set based on lung reclutability assessed with Recruitment-to-Inflation Ratio (RIR). At the conclusion of the procedure, LUSS was reassessed (T1). Data are reported as median and 25-75th interquartile range.

**Results**

Between January and April 2024, we included 10 patients (5 males) with a median age of 59 years (48–66) and a median body mass index of 36 kg/m2 (34–39). Higher PEEP levels (median 15, 13–17) were set in case of high lung recruitability (RIR >  = 0.5), whereas lower PEEP levels (median 7, 6–9) were administered in instances of low lung recruitability (RIR < 0.5). During surgery FiO2 remained unchanged in both groups 0.49 (0.46–0.5) vs. 0.5 (0.4–0.63). Oxygenation was maintained with SpO2/FiO2 at 457 (442–467) postoperatively compared to 471 (463–475) preoperatively, despite a slight increase of LUSS from 0 (0–2) at baseline to 3 (2–4) after surgery. If RIR was >  = 0.5, LUSS increased from 0 (0–1) at T0 to 2(2–3) at T1. Conversely, in the RIR < 0.5 subgroup, LUSS increased from 0(0–1) at T0 to 4(2–6) at T1. Analyzing regional differences, significant effects were observed in the dorsal parts of the lung compared to the anterior regions: 2 (1–3) vs 0 (0–0) in patients with high potential for lung recruitment, and 4 (3–4) vs 0 (0–0) in patients with low potential for lung recruitment. In one patient, a LUSS > 10 was recorded, with an increase of 11 points from baseline (13 vs 2), yet this did not result in major effects on oxygenation (SpO2/FiO2: 461).

**Conclusions**

In obese patients undergoing laparoscopic or robotic surgery, despite adequate PEEP setting according to RIR, a slight worsening of LUSS was observed postoperatively. This effect is more pronounced in patients with low potential for lung recruitment and especially in the dorsal parts of the lungs.

**References**

1. Nestler C, et al. Individualized positive end-expiratory pressure in obese patients during general anaesthesia: a randomized controlled clinical trial using electrical impedance tomography. Br J Anaesth. 2017
2. PROBESE Collaborative Group, Effect of Intraoperative High Positive End-Expiratory Pressure (PEEP) With Recruitment Maneuvers vs Low PEEP on Postoperative Pulmonary Complications in Obese Patients: A Randomized Clinical Trial. JAMA. 2019

### A149 Recruitment-to-inflation ratio in obese patients undergoing laparoscopic/robotic surgery

#### F. Verdina ^1,2^, G. Furlan ^2^, D. Rosalba ^1,2^, S. Spadaro ^3,4^, G. Scaramuzzo ^3,4^, D.L. Grieco ^5^, S.M. Maggiore ^6^, L. Ball ^7^, C. Gregoretti ^8^, A. Cortegiani ^8^, M. Carron ^9^, P. Navalesi ^9^, S. Gentilli ^2,10^, R. Romito ^11^, D. Surico ^2,12^, A. Volpe ^2,13^, E. DE Robertis ^14^, R. Vaschetto ^1,2^, R. Simonte ^14^, G. Cammarota^2,15^

##### ^1^ Anestesia e Terapia Intensiva, Azienda Ospedaliero Universitaria Maggiore della Carità, Novara, Italy; ^2^ Dipartimento di Medicina Traslazionale, Università del Piemonte Orientale, Novara, Italy; ^3^ Dipartimento di Medicina Traslazionale, Università degli Studi di Ferrara, Ferrara, Italy; ^4^ Anestesia e Terapia Intensiva, Azienda Ospedaliero Universitaria di Ferrara, Ferrara, Italy; ^5^ Dipartimento di Emergenze, Terapia Intensiva e Anestesia, Fondazione Policlinico Universitario A. Gemelli IRCCS, Roma, Italy; ^6^ Dipartimento di Anestesia e Terapia Intensiva, Ospedale SS Annunziata e Dipartimento di Tecnologie innovative in Medici, Chieti, Italy; ^7^ Dipartimento di Scienze chirurgiche e Diagnostiche integrate, Università di Genova, Genova, Italy; ^8^ Dipertimenti di Scienze Chirurgiche, Oncologiche e Stomatologiche, Università di Palermo, Palermo, Italy, ^9^ Dipartimento di Medicina (DIMED), Policlinico Universitario di Padova e Istituto di Anestesia e Terapia, Padova, Italy; ^10^ Clinica Chirurgica, Azienda Ospedaliero Universitaria Maggiore della Carità, Novara, Italy; ^11^ Chirurgia Generale 2 Azienda Ospedaliero Universitaria Maggiore della Carità, Novara, Italia, Novara, Italy; ^12^ Ginecologia e Ostetricia, Azienda Ospedaliero Universitaria Maggiore della Carità, Novara, Italia, Novara, Italy; ^13^ Urologia, Azienda Ospedaliero Universitaria Maggiore della Carità,, Novara, Italy; ^14^ Dipartimento di Medicina e Chirurgia, Università degli Studi di Perugia, Perugia, Novara, Italy; ^15^ Anestesia e Rianimazione, Azienda Ospedaliero Universitaria di Alessandria SS.Antonio e Biagio e Cesare Arrigo, Alessandria, Italy

###### **Correspondence:** F. Verdina

*Journal of Anesthesia, Analgesia and Critical Care 2024*, **4(1):**A149

**Background**

Setting positive end-expiratory pressure (PEEP) in obese patients undergoing surgery could be challenging. Recruitment-to-inflation ratio (RIR) allows to assess lung recruitability [1], but its application in surgery has not been explored yet. The present study sought at investigating the feasibility of RIR maneuver to assess lung recruitability in obese patients undergoing laparoscopic/robotic surgery with application of pneumoperitoneum (PNP).

**Materials and Methods**

Adult obese patients (body mass index > 30 kg/m2) undergoing laparoscopic or robotic abdominal/pelvic surgery were prospectively enrolled after informed consent collection. Exclusion criteria were severe chronic respiratory or impaired cardiac conditions. With intubated patients undergoing general anesthesia and protective mechanical ventilation, PNP was applied and definitive body position was achieved. Afterwards, a low-flow insufflation maneuver to assess airway opening pressure (AOP) was performed and two different RIR assessment were performed: after a 5-min high-PEEP test and, subsequently, after a 30-min high-PEEP test. RIR was assessed through a simplified one-breath derecruitment maneuver with prolonged exhalation from PEEP = 15 cmH2O to PEEP = 5 cmH2O. In case AOP > 5 cmH2O, high PEEP was AOP + 10 cmH2O (1). RIR >  = 0.5 indicated high potential for lung recruitment; RIR < 0.5 low potential for lung recruitment. SpO2/FiO2 was computed and data regarding ventilatory setting and vital parameters were collected after intubation (baseline), following PNP application, and after RIR maneuvers. Data are reported as median and 25-75th interquartile range.

**Results**

From January to April 2024, 10 patients (5 male) with a median age of 59 (48–66) years and a median body mass index of 36 (34–39) kg/m2 were included. Two patients were excluded for severe comorbidities and laparotomic conversion during surgery.

AOP was observed in 50% of the cases with a median value of 8 (8–10) cmH2O. When assessed after the 5-min high-PEEP test, median RIR was 0.16 (0.06–0.43); when assessed after the 30-min high-PEEP test, RIR was 0.51 (0.14–0.59). In one patient, the RIR after 30 min at high PEEP was not computed due to the early ending of the surgery. Two patients vs. five patients were considered having high potential for lung recruitment with RIR assessed after 5 or 30 min, respectively. Overall, at 30 vs. 5 min, the potential for lung recruitment changed in three patients (30%).

**Conclusions**

In obese patients undergoing laparoscopic/robotic surgery with application of PNP, RIR maneuver was feasible to assess potential for lung recruitment. The present study indicates that exposure lasting at least 30 min is needed to properly exploit PEEP effects on lung recruitment [2], also in the intraoperative setting.

**References**

1. Chen L et al. Potential for Lung Recruitment Estimated by the Recruitment-to-Inflation Ratio in Acute Respiratory Distress Syndrome. A Clinical Trial AJRCCM 2020
2. Cammarota G et al. PEEP-induced alveolar recruitment in patients with COVID-19 pneumonia: take the right time! Crit Care 2021
3. Collaborative group for PEEP LAP study: Laura Carrera, Francesca Santangelo, Alessio Rivolta, Alessia Guzzo, Filippo Vietti, Alberto Migliavacca, Luca Portigliotti, Michele Giana, Alessandro Vigone, Michele Billia, Giovanni Misseri;

### A150 Neuromuscular monitoring and incidence of postoperative residual curarization: a prospective observational study (Porc Trial)

#### A. Piersanti, R. Garra, F. Sbaraglia, R. Lamacchia, C. Cuomo, M. Gozza, G. Bernardi, F. Maiellare, D. Posa, M. Rossi

##### Fondazione Policlinico Agostino Gemelli IRCCS. Università Cattolica del Sacro Cuore, Rome, Italy

###### **Correspondence:** D. Posa

*Journal of Anesthesia, Analgesia and Critical Care 2024*, **4(1):**A150

**Background**

Non-depolarizing neuromuscular blocking (NMB) agents are commonly used in anesthesia practice to facilitate tracheal intubation and allow muscle relaxation during surgery.

An incomplete postoperative recovery of neuromuscular function potentially expose the patient to adverse respiratory events.

Anesthesiologist's subjective qualitative assessment of the patient's recovery of muscle strength before extubation based solely on clinical signs is not predictive of adequate neuromuscular recovery. An expert consensus statement in 2018 suggested that a quantitative and objective assessment of neuromuscular function using the train-of-four ratio (TOFR) acceleromyographic method at the level of the adductor muscle of the thumb represents the best way to minimize this risk.

Aim of this study was the evaluation of the incidence of residual curarization upon arrival in the PACU in a cohort of surgical patients receiving NMB agents.

**Methods**

This single-center observational cohort study was approved by the Internal Ethic Committee (ID Number: 5991, Protocol Number 0000494/23) on 23/11/2023. The study was registered at ClinicalTrials.gov (NCT06193213, on 05/01/2024) and the protocol conforms to the Declaration of Hensinki.

The study was performed at the IRCCS Fondazione Policlinico Universitario Agostino Gemelli of Rome, Italy between February and April 2024 according to STROBE guidelines for observational studies. Informed consent was obtained from all participants included in the study.

Primary outcome was the incidence of postoperative residual neuromuscular blockade upon arrival at the PACU, defined as a TOFR < 0.9 by acceleromyographic method in 90 patients who received NMB agents for tracheal intubation and/or for maintaining paralysis during surgery. We performed 2 TOFR measurements 30 s apart. If their difference was < 10%, we considered the average value while in case of a difference > 10%, a third measurement was taken and we considered the average of the two closest results.

Secondary outcomes were the number of any respiratory adverse events (defined as episodes of SpO2 < 92% requiring O2 supplementation or the finding of atelectasis, pneumonia or non-cardiac pleural effusion on imaging tests) occurred in the PACU and before hospital discharge, as well as residual neuromuscular blockade estimated risk factors by logistic regression model.

Preliminary results. In our cohort of 90 patients (median age 57 (41, 72) years), the incidence of residual neuromuscular blockade was 4% at arrival at the PACU (Table 1). Rocuronium was the only agent used. Five (5%) patients received > 1 dose of rocuronium during surgery and in 11 (12%) patients neuromuscular blockade was reversed with sugammadex before extubation. Of note, only 41 (45%) anesthesiologists reported any form of neuromuscular transmission monitoring on anesthesia sheets. One (1%) patient with residual curarization received O2 supplementation during PACU stay. Among patients with no residual neuromuscular blockade 9 (10%) of them received O2 for episodes of SpO2 < 92% during PACU stay and 3 (3%) of them also during hospitalization (Table 2).

**Conclusions**

Residual neuromuscular blockade frequently occurs even when a single NMB dose is administered and represents a relevant cause of preventable desaturation during postoperative care. Efforts to improve awareness of the problem among anesthesiologists increasing routine monitoring of neuromuscular function is warranted.

**Table 1:** **(abstract A150).** See text for description

Characteristic	Total patients (N = 90)
Age, years	57 (41, 72)
Height, cm	168 ± 9
Weight, Kg	73 ± 13
Body mass index, Kg/m^2^	26 ± 4
Female	47 (52)
Male	43 (48)
ASA status:	
1	27 (30)
2	54 (60)
3	9 (10)
Comorbidities:	
Cardiovascular diseases	33 (37)
Pulmonary diseases	11 (12)
Diabetes	6 (7)
Renal	1 (1)
History of cancer	15 (17)
Type of surgery:	
Head and neck	45 (50)
Spinal	28 (31)
Breast	7 (8)
Reconstructive	7 (8)
Orthopedic	3 (3)

Demographic and surgical characteristics of the study population. Data are presented as N (%), mean ± standard deviation or median (interquartile range)

*ASA* American Society of Anaesthesiologists

**Table 2:** **(abstract A150).** See text for description

Characteristic	**Total patients (N = 90)**
Duration of surgery, min	87 (58, 170)
Duration of anesthesia, min	121 (79, 215)
Time from last NMBD dose administration to the end of anesthesia in patients who received > 1 dose of NMBD, min	167 (115, 185)
Time from end of anesthesia to PACU arrival, min	6 (5, 8)
Inhalational anesthesia	51 (57)
Target controlled infusion anesthesia	39 (43)
Total dose of sufentanil, mcg	15 (10, 20)
Total dose of fentanyl, mcg	200 (100, 200)
Use of remifentanil	52 (58)
Neuromuscular blocking agent:	
Rocuronium	90 (100)
Total dose of rocuronium, mg	30 (20, 50)
Received ≥ 1 dose of rocuronium	5 (5)
Reversal agent use:	
Sugammadex	11 (12)
Total dose of sugammadex, mg	200 (200, 300)
Number of anesthesiologists who reported intraoperative neuromuscular monitoring in anesthesia charts	41 (45)
Reported basal TOFR at induction of anesthesia	3 (3)
Reported TOFR on anesthesia sheet at extubation	24 (27)
Incidence of TOFR ≤ 90% at PACU arrival	4 (4)
Patients with episodes of SpO_2_ ≤ 92% during PACU stay	25 (28)
Patients with TOFR ≤ 90% requiring O_2_ supplementation during PACU stay	1 (1)
Patients with no residual NMB receiving O_2_ supplementation during PACU stay	9 (9)
Patients who received O_2_ supplementation during hospitalization	3 (3)

Data are presented as N (%), mean ± standard deviation or median (interquartile range)

*NMBD* neuromuscular blocking drug, *PACU* Post-Anesthesia Care Unit, *TOFR* Train-of-four ratio

### A151 Anesthetic management of a giant inferior vena cava leiomyosarcoma: a case report

#### G. Torregiani ^1^, P. Papa ^2^, G. Gazzè ^2^, V. Ceccarelli ^2^, S. Orlando ^2^, F. Sardellitti ^1^, E. Venti ^1^, C. Coccia ^1^, E.M.A. Forastiere^1^

##### ^1^Department of Anesthesia, Intensive Care and Pain Therapy, IRCCS—Regina Elena National Cancer Institute, Roma, Italy; ^2^Department of Anesthesia, Intensive Care and Pain Therapy, Policlinico Umberto I, Sapienza University of Rome, Roma, Italy

###### **Correspondence:** P. Papa

*Journal of Anesthesia, Analgesia and Critical Care 2024*, **4(1):**A151

**Background**

Leiomyosarcoma (LMS) of the inferior vena cava (IVC) is a rare malignant retroperitoneal tumor emerging from the smooth muscle cells of the venous vassels. It presents as intra- or extra-luminal growth and often involves vital retroperitoneal organs and vascular structures [1].

**Case Report**

Our case involves a 62-year-old man (83 kg, BMI 25.6 kg/m^2^), ASA 3, diagnosed with a rare LMS of IVC. He presented with hypertension, anemia (Hb 10.6 g/dL), pyelonephritis, and AKI due to tumor infiltration of the right kidney and ureter, managed with antibiotics and percutaneous nephrostomy.

CT-scan revealed an occlusive lesion of the infrarenal IVC measuring 24 × 20x16 cm by-passed by retroperitoneal collateral circles (Fig. 1). The lesion infiltrated the kidney, right ureter and established contiguous relationships with the abdominal aorta, which appeared modestly compressed, the left renal vein, and the right renal artery, necessitating en bloc excision.

Surgery was performed under general endotracheal anesthesia. Three PVC and one CVC were placed.

Hemodynamic management was guided by continuous monitoring of SVV, CI, IBP, ECG, HR, PetCO2, BIS, temperature by esophageal and bladder probe and SpO2 at left upper limb and lower limb bilaterally.

Intraoperatively, careful monitoring of blood count and coagulation status was conducted through serial arterial blood gas analyses and laboratory tests. The system for intraoperative hemorecovery was arranged.

The most critical moment occurred during the tangential clamping of the subrenal aorta, coupled with complete clamping of the IVC to facilitate excision of the subrenal IVC segment, tumor mass, kidney, adrenal gland, and right ureter. In that circumstance, monitoring revealed values of: PAM 48 mmHg, SVV 24%, CI 1.6L/min/m2 and HR 112 bpm. In addition, the minimum Hb level reached 5.3 g/dL.

Therefore, we positioned the patient in Trendelemburg at 30-degrees angle, administered norepinephrine and adrenaline at maximum dose of 0.08mcg/kg/min and 0.18mcg/kg/min, respectively, and promptly infused 1000 mL of Gelofusine and 100 mL of Albumin20%, achieving improvement in hemodynamic parameters (PAM 68 mmHg, SVV 16%, CI 2.9L/min/m2 and HR 105 bpm).

Throughout the procedure, a total of eight units of packed red blood cells, two units of fresh frozen plasma, and one unit of platelets were administered.

Renal function was maintained by correcting metabolic acidosis with bicarbonates (340 mEq) and administering Furosemide in refractory boluses (40 mg).

The doses of vasopressors were gradually reduced until adrenaline was discontinued entirely and norepinephrine was maintained at 0.03mcg/kg/min.

Subsequently, the patient, intubated, was admitted to the ICU with an hemoglobin level of 8.9 g/dL, INR 1.27 and aPTTratio 1.34. The patient was discharged from the hospital on the sixth day after the surgery.

**Conclusions**

The management of patients with retroperitoneal LMS of the IVC presents unique challenges. Effective intraoperative management requires meticulous planning, vigilant monitoring, and prompt intervention to optimize outcomes and minimize perioperative complications. Collaboration among surgical, anesthesia, and critical care teams is essential for achieving favorable outcomes in these complex cases.

Informed consent to publish had been obtained.

**Reference**

1. Kapoor R, Bansal A, Sharma SC. Leiomyosarcoma of inferior vena cava: Case series of four patients. J Cancer Res Ther. 2015;11(3):650-650

**Fig. 1:**
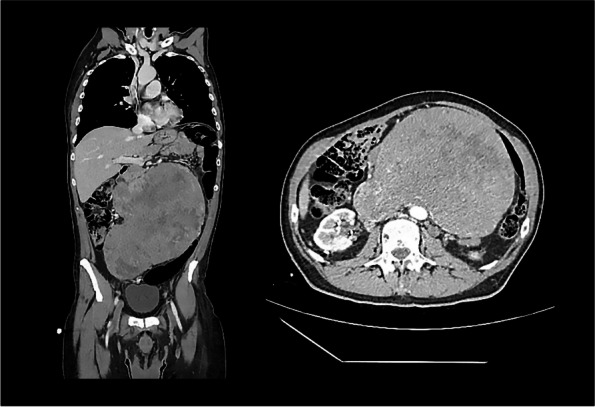
**(abstract A151).** CT-scan revealed an occlusive lesion of the infrarenal IVC measuring 24 × 20x16 cm by-passed by retroperitoneal collateral circles

### A152 The dual use of smote and ghost algorithms: handling class imbalance in ai-driven post-operative acute kidney failure prediction in the isaki (Intelligent Score for AKI) project

#### V. Bellini ^1^, D. Azzolina ^2,3^, M. Panizzi ^1^, F. Bezzi ^1^, M. Mion ^1^, E. Bignami^1^

##### ^1^ Anesthesiology, Critical Care and Pain Medicine Division, Department of Medicine and Surgery, University of Parma, Parma, Italy; ^2^ Department of Environmental and Preventive Science, University of Ferrara, Ferrara, Italy, ^3^ Clinical Trial and Biostatistics, Research and Development Unit, University Hospital of Ferrara, Ferrara, Italy

###### **Correspondence:** M. Panizzi

*Journal of Anesthesia, Analgesia and Critical Care 2024*, **4(1):**A152

**Background and Objectives**

Post-operative acute kidney injury (PO-AKI) remains a common complication, affecting approximately one-fifth of patients after major surgery [1]. Although several PO-AKI prediction models already exist, they contain many biases [2]. The ISAKI study aims to create a dynamic and patient-tailored risk model for the development of PO-AKI using Artificial Intelligence (AI) and Machine Learning (ML). The model will be able to predict the onset of PO-AKI at three distinct times: preoperatively, postoperatively and at ward discharge.

**Methods**

ISAKI is a retrospective-prospective, observational, single-center study (ethics-committee-registration-number:803/2022 /OSS/AOUPR).

After obtaining informed consent, patients undergoing major non-cardiac surgery were enrolled. Once the dataset has been created, several ML algorithms (Random Forest, Gradient Boosting Machine, Neural Network, and Generalized Linear Model) were trained by considering 100 bootstrap internal validation runs.

This study applies a dual strategy to tackle the classification imbalance in PO-AKI prediction: the employment of the synthetic minority oversampling technique (SMOTE) and the innovative generalized tHreshOld ShifTing (GHOST) procedure. SMOTE addresses this imbalance by augmenting the minority class through synthetic data generation, directly influencing the composition of the training dataset. Complementarily, GHOST optimizes the decision threshold based on the distribution of prediction scores, effectively adjusting the bias towards the majority class typically seen in imbalanced datasets [3].

**Results**

Preliminary data from retrospective patients and the first prospectively enrolled patients are presented. Patient’s charateristics are summarized in Table 1. The model performed reasonably well considering our limited sample size and the outcome imbalance (25 events of PO-AKI among 313 healthy patients). The internal validation balanced accuracy on internal testing was 0.7, achieved by the Random Forest trained with the SMOTE and GHOST procedures (Fig. 1). After the training phase, which exploited the automated GHOST procedure, the training balanced accuracy was 0.82.

Regarding the preoperative variables impact on the onset of PO-AKI it emerges that preoperative creatinine level and the presence of cardiovascular disease are the most impactful.

**Discussion and Conclusion**

Even though the results obtained are still partial, this preliminary model we created showed a satisfying performance. This encourages us to continue the study to further enhance the model's performance and reliability. Furthermore, our work provides an example of how it is possible to use synthetic data in preliminary analyses when only an unbalanced dataset it’s available.

**References**

1. Vaara ST, Bellomo R. Postoperative renal dysfunction after noncardiac surgery. Curr Opin Crit Care. 2017 Oct;23(5):440–446.
2. Bell S, Prowle J. Is postoperative AKI-prevention Better than Cure? J Am Soc Nephrol. 2019 Jan;30(1):4–6. 10.1681/ASN.2018111127.
3. Esposito C, Landrum GA, Schneider N, et al. GHOST: Adjusting the Decision Threshold to Handle Imbalanced Data in Machine Learning. J Chem Inf Model. 2021 Jun 28;61(6):2623–2640. 10.1021/acs.jcim.1c00160. Epub 2021 Jun 8. PMID: 34100609.

**Fig. 1:**
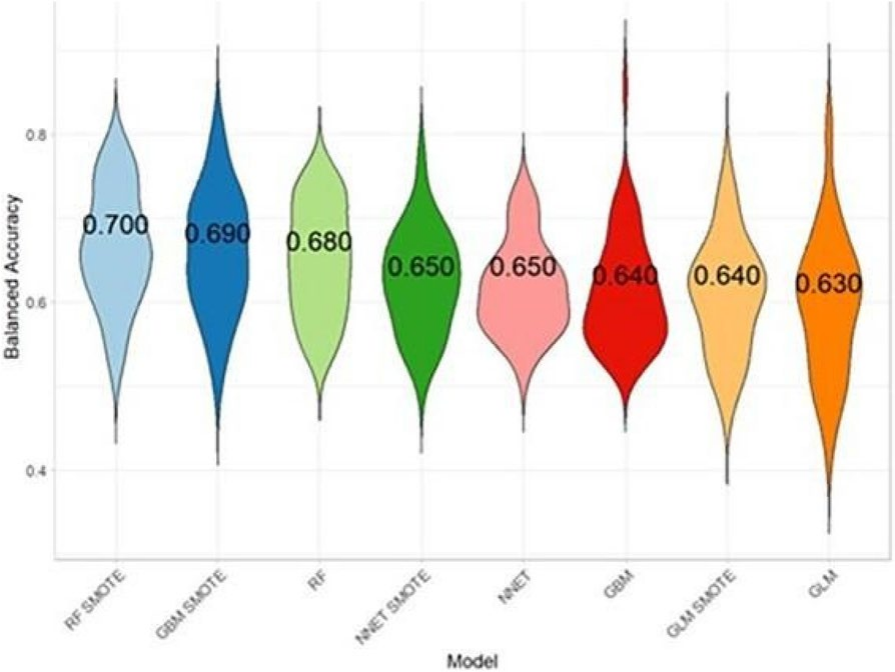
**(abstract A152).** Balanced accuracy GHOST Plot and models with and without SMOTE. (RF = Random Forest, GBM = Gradient Boosting Machine, NNET = Neural Network, GLM = Generalized Liner Model)

**Table 1:** **(abstract A152).** The first 313 enrolled patients charateristics are presented using descriptive statistics

Data analysis using descriptive statistic	
*Sample of 313 patients (Expected sample: 689)*	
Gender	Men 72% (226 patients)
	Women 28% (87 patients)
Surgery type	Urological surgery 53.7% (168 patients)
	General surgery 45% (141 patients)
	Vascular surgery 0.3% (1 patient)
	Thoracic surgery 1% (3 patient)
Development PO-AKI	Yes 8% (25 patients)
	No 92% (288 patients)
Resolution PO-AKI	Yes 48% (12 patients)
	No 52% (13 patients)
Onset PO-AKI	Early 88% (22 patients)
	Late 12% (3 patients)

### A153 Efficacy of post-operative oxygen therapy using High-Flow Nasal Cannulas (HFNC) in the prevention of immediate post-operative respiratory complications

#### G. Paladini, A. Di Filippo, L. Foti, G. Villa, T. Del Santo, S. Romagnoli

##### Department of Anesthesia and Critical Care Azienda Ospedaliera-Universitaria Careggi, University of Florence, Firenze, Italy

###### **Correspondence:** G. Paladini

*Journal of Anesthesia, Analgesia and Critical Care 2024*, **4(1):**A153

**Background**

The widespread diffusion of the HFNC oxygenation method during the period of the Sars-Cov-2 pandemic for the treatment of respiratory insufficiency has aroused growing interest in its use as an alternative to standard oxygen therapy[1,2,3]. Although many papers have dealt with the effectiveness of its post-operative use in reducing the incidence of post-operative complications [4,5,6,7,8], it is still not clear what its role is in reducing immediate post-operative respiratory complications. The aim of this pilot study is therefore to evaluate the efficacy of oxygen therapy using HFNC, administered in the first 2 post-operative hours, in the prevention of immediate post-operative respiratory complications in major surgery. Informed consent was collected for each patient who participated in this pilot study.

**Materials and methods**

Two groups of 22 patients were selected: the study group (H) and the control group (C). The groups were not different in ASA class, BMI, gender, age, preexisting respiratory pathologies and types of surgery. Once patients arrived in the Recovery Room, those in group H were administered HFNC for a duration of 2 h at 60 L/min, patients in group C received a standard protocol of O2 administration with facial mask or nasal cannulae; the FiO2 in the two group, was modulated according to the patient's SpO2 (for SpO2 greater than or equal to 94%, the FiO2 was set at 21%). SpO2, HR, RR and ROX index [9,10,11,12,13] were detected at the following times: T0 (5 min from the start of the administration of HFNC), T1 (1 h after), T2 (10 min after removal of high flow, discontinued 1 h after T1) and on day 1 (G1). In group H also the PaO2 was collected.

**Results**

The two groups were not statistically different for SpO2, HR, RR detected at T0, T1, T2 and G1. ROX index was statistically higher in group H (Group H vs Group C = 31.5 ± 6.9 vs 25,3 ± 7,8 p < 0.05) at T2; SpO2 in the two groups was not different, but FiO2 was higher at T2 in group C vs group H (Group H vs Group C = 21 ± 0 vs 29,95 ± 12,81 p < 0.05). PaO2/FiO2 calculated at T1 in group H exceeds the value of 400 indicating a very effective state of oxygenation.

**Conclusions**

These preliminary data indicate the achievement, at the end of recovery in recovery room, of a better oxygenation status in the HFNC treated patients with no need of supplemental Oxygen administration. This finding is assumed to be due to the HFNC mechanism of action resulting in an improvement in the ventilation/perfusion ratio, that is so effective to indicate an increasing diffusion of HFNC application in the immediate post-operative period to preventing post-operative immediate respiratory complications.

**References**

1. Effect of High-Flow Oxygen Therapy vs Conventional Oxygen Therapy on Invasive Mechanical Ventilation and Clinical Recovery in Patients With Severe COVID-19: A Randomized Clinical Trial. Ospina-Tascón G.A., Calderón-Tapia L.E., García A.F. JAMA, 2021.
2. Wang Y, Ni Y, Sun J, Liang Z. Use of High-Flow Nasal Cannula for Immunocompromise and Acute Respiratory Failure: A Systematic Review and Meta-Analysis. J Emerg Med. 2020 Mar.
3. Guo K, Liu G, Wang W, Guo G, Liu Q. Effects of high-flow nasal oxygen cannula versus other noninvasive ventilation in extubated patients: a systematic review and meta-analysis of randomized controlled trials. Expert Rev Respir Med. 2021 Aug 17.
4. Gaspari R, Spinazzola G, Ferrone G, Soave P-M, Pintaudi G, Cutuli S-L, Avolio A-W, Conti G, and Antonelli M. High-Flow Nasal Cannula Versus Standard Oxygen Therapy After Extubation in Liver Transplantation: A Matched Controlled Study. Respiratory Care. 2020, January.
5. Huang H-W, Sun X-M, Shi Z-H, Chen G-Q, Chen L, Friedrich J-O, and Zhou J-X. Effect of High-Flow Nasal Cannula Oxygen Therapy Versus Conventional Oxygen Therapy and Noninvasive Ventilation on Reintubation Rate in Adult Patients After Extubation. A Systematic Review and Meta-Analysis of Randomized Controlled Trials. J Intensive Care Med. 2018 Nov.
6. Xiang GL, Wu QH, Xie L, Song JQ, Wu X, Hao SY, Zhong M, Li SQ. High flow nasal cannula versus conventional oxygen therapy in postoperative patients at high risk for pulmonary complications: A systematic review and meta-analysis. Int J Clin Pract. 2021 Mar.
7. Lu Z, Chang W, Meng S–S, et al. Effect of high-flow nasal cannula oxygen therapy compared with conventional oxygen therapy in postoperative patiens: a systematic review and meta-analysis. BMJ Open 2019.
8. Wang Y, Zhu J, Wang X, Liu N, Yang Q, Luan G, Ma X and Liu J. Comparison of High-Flow Nasal Cannula (HFNC) and Conventional Oxygen Therapy in Obese Patients Undergoing Cardiac Surgery: A Systematic Review and Meta-analysis. In Vivo 2021 July.
9. Reyes LF, Bastidas Goyes A, Tuta Quintero EA, Pedreros KD, Mantilla YF, Herrera M, Carmona GA, Saza LD, Bello LE, Muñoz CA, Chaves JC, Arias JC, Alcaraz PM, Hernández MD, Nonzoque AP, Trujillo N, Pineda AF, Montaño GS. Validity of the ROX index in predicting invasive mechanical ventilation requirement in pneumonia. BMJ Open Respir Res. 2022 Sep.
10. Zhou X, Liu J, Pan J, Xu Z, Xu J. The ROX index as a predictor of high-flow nasal cannula outcome in pneumonia patients with acute hypoxemic respiratory failure: a systematic review and meta-analysis. BMC Pulm Med. 2022 Apr 1.
11. Roca O, Caralt B, Messika J, Samper M, Sztrymf B, Hernández G, García-de-Acilu M, Frat JP, Masclans JR, Ricard JD. An Index Combining Respiratory Rate and Oxygenation to Predict Outcome of Nasal High-Flow Therapy. Am J Respir Crit Care Med. 2019 Jun 1.
12. Chandel A, Patolia S, Brown AW, Collins AC, Sahjwani D, Khangoora V, Cameron PC, Desai M, Kasarabada A, Kilcullen JK, Nathan SD, King CS. High-Flow Nasal Cannula Therapy in COVID-19: Using the ROX Index to Predict Success. Respir Care. 2021 Jun.
13. Yu PT, Chen CH, Wang CJ, Kuo KC, Wu JC, Chung HP, Chen YT, Tang YH, Chang WK, Lin CY, Wu CL. Predicting the successful application of high-flow nasal oxygen cannula in patients with COVID-19 respiratory failure: a retrospective analysis. Expert Rev Respir Med. 2023 Apr.

### A154 Preoperative infusion of levosimendan in heart failure patient undergoing robotic cystectomy: a case report

#### G. Torregiani ^2^, S. Orlando ^1^, G. Gazzè ^1^, P. Papa ^1^, V. Ceccarelli ^1^, H. Matteucci ^1^, M. Covotta ^2^, M.E. Marcelli ^2^, E. Forastiere^2^

##### ^1^Università di Roma La Sapienza, Roma, Italy; ^2^ IRCCS Regina Elena National Cancer Institute, Roma, Italy

###### **Correspondence:** S. Orlando

*Journal of Anesthesia, Analgesia and Critical Care 2024*, **4(1):**A154

**Background**

Heart failure is an important risk factor for mortality and cardiovascular complications after noncardiac surgery [1,2]. Levosimendan is a calcium sensitizer. Levosimendan administration is associated with a reduction in preload and afterload and an increase in coronary blood flow, plus an energetically favorable type of increase in myocardial contractility. Improved myocardial tissue perfusion might contribute to cardioprotective effect of levosimendan [3]. Levosimendan may be safe and effective for the perioperative optimization of patients with heart failure undergoing elective non-cardiac surgery [4]. There are few clinical studies on levosimendan in non-cardiac surgery [5].

**Case Report**

71 year-old man, BMI 21.60 kg/m2, post-chemotherapy dilated cardiomyopathy with ejection fraction 38%, previous Hodgkin's lymphoma, diabetes mellitus, 25 pack-year smoker, coronary angiography without stenosis. Home therapy: antiaggregant, SGLT2 inhibitors, ACE inhibitor, beta blocker. Denies angor, dyspnea, heart palpitations or lipothymia, reported good functional capacity. Increased cardiac surgical risk. He is admitted to the intensive care unit the day before the scheduled cystectomy + orthotopic neobladder surgery. Basal vital parameters were heart rate (HR) 61 bpm, mean arterial pressure (MAP) 98 mmHg, SpO2 99%, P/F ratio 419. Central venous catheter and arterial cannula were positioned, advanced hemodynamic monitoring is started with cardiac index (CI) 4.1 L/min/m2, global ejection fraction (GEF) 21%. Levosimendan started at 0.1 mcg/kg/min and continued for 24 h. After we proceeded with surgery. Intraoperative monitoring: electrocardiography, HR, SpO2, advanced hemodynamic monitoring, BIS, mioresolution, EtCO2. During the entire duration of the operation, the patient was hemodynamically stable without drugs. CI was between 3.5 and 3.9 L/min/m2, good respiratory exchanges. Fluids administered 2500 ml. Operation duration approximately 275 min, anesthesia duration approximately 300 min. At the end of the surgery, the patient is extubated and transferred to intensive care unit. The patient was alert and cooperative, in valid spontaneous breathing, stable hemodynamics with good hemodynamic indices (CI 3.9 L/min/m2, GEF 20%, HR 61 bpm, MAP 89 mmHg), diuresis valid (100 ml/h). During the first postoperative day the patient remains stable, good hemodynamic indices (CI 3.9 L/min/m2, GEF 23%, HR 58 bpm, MAP 75 mmHg) (Table 1) without infusion of vasoconstrictors or cardiokinetics. Respiratory exchanges were good (SpO2 98%, P/F ratio 279). Diuresis present with bolus diuretic stimulus. Resumes home therapy. On the second postoperative day, the patient was transferred to the urology department. The patient remained stable for the entire duration of the hospital stay and was discharged on the ninth postoperative day.

**Conclusion**

During robotic cystectomy, carbon dioxide insufflation, high abdominal pressure, steep Trendelenburg position and prolongation of the surgical time result in hemodynamic and homeostatic alterations involving increases in arterial pressure and systemic vascular resistance [6]. In a patient with reduced ejection fraction and increased cardiac surgical risk, it is desirable to optimize cardiac performance preoperatively. Levosimendan could be safely administered in patients with chronic heart failure and reduced ejection fraction undergoing major oncological abdominal surgery, but preoperative prophylactic treatment with levosimendan in these patients deserves further study.

Informed consent to publish had been obtained.

**Table 1:** **(abstract A154).** Trend of hemodynamic indices after Levosimendan infusion

	**1 h**	**3 h**	**Intraop**	**3 h postop**	**6 h postop**	**24 h postop**
C.I. (L/min/m2)	3.6	3.5	3.9	3.9	3.1	3.9
SVI (ml/m2)	60	55	57	57	42	62
SVV (%)	5	12	15	11	7	5
SVRI (dyne-s-m2/cm2)	2142	1977	1478	1905	2115	1104
GEF (%)	22	22	21	20	24	23
MAP (mmHg)	96	96	93	92	80	62

### A155 Impact of midazolam premedication on propofol concentrations: comparing schnider and eleveld models in TCI general anesthesia—a prospective observational study

#### F. Linassi ^1^, P. Zanatta ^2^, L. Spanò ^3^, C. Rizzetto ^2^, M. Carron^3^

##### ^1^Dipartimento di Scienze del Farmaco UNIPD, Padova, Italy; ^2^ ULSS2 Marca Trevigiana, Treviso, Italy; ^3^ Istituto di Anestesia e Rianimazione, Padova, Italy

###### **Correspondence:** F. Linassi

*Journal of Anesthesia, Analgesia and Critical Care 2024*, **4(1):**A155

**Background**

Midazolam is frequently used as premedication for general anesthesia due to its sedative, anxiolytic, and amnestic effects. 1,2 It significantly influences Bispectral Index (BIS) scores, reduces the required propofol dose for anesthesia induction, and impacts the estimated concentration at the effector site of Propofol (CeP) with the Diprifusor Pump. 3 However, no studies have assessed midazolam’s effects on CeP using the Schnider or Eleveld PK/PD models in Total Intravenous Anesthesia with Target Controlled Infusion (TIVA-TCI), nor their outcomes. 4

**Methods**

Following approval from the Institutional Ethical Committee (registered as NCT05800288), a prospective observational study was conducted at Treviso Regional Hospital, Italy. This study evaluated the impact of midazolam premedication on CeP values at Loss of Responsiveness (LoR), during Maintenance of Anesthesia (MA), and at Return of Responsiveness (RoR) in adult female patients undergoing breast cancer surgery with TIVA-TCI using the Schnider and Eleveld models. It also explored the incidence of unwanted spontaneous responsiveness events (USRE), burst suppression events (BSuppE), and post-operative delirium (POD).

**Results**

Eighty patients were enrolled. Significant differences in CeP values were noted across three time points (LoR, MA, RoR) for different age groups and both model groups (p < 0.001). Unwanted anesthesia events occurred in 36.3% of patients, with USRE in 10% and BSuppE in 26.3%; POD was experienced by 1.2% of patients. CeP values at RoR significantly differed between adults and elders.

General Population: No significant demographic or anesthesia duration differences were.

observed between the group receiving compared with the group not receiving midazolam premedication. The total dose of propofol was significantly higher in patients not receiving midazolam, with increased BIS, CeP at LoR, CeP during MA, CeR during MA, and.

Schnider Model: Similar to the general population, propofol dosage was significantly higher without midazolam, with increased values of CeP at LoR, CeP during MA, CeR during MA, and δCeP at RoR (Img 1).

Eleveld Model: No demographic differences or differences in anesthesia duration were noted. While propofol doses did not differ significantly, BIS and CeP at LoR, CeP during MA, CeR during MA, and δCeP at RoR were significantly higher in the non-premedicated group. BSuppE rates were notably lower in the midazolam premedicated group (Table 1).

**Conclusion**

Midazolam premedication significantly affects CeP during TIVA-TCI with both the Eleveld and Schnider models, requiring lower doses of propofol and particularly reducing BSuppE with the Eleveld model.

Informed consent was obtained.

**References**

1. Nordt SP, Clark RF. Midazolam: a review of therapeutic uses and toxicity. J Emerg Med. 1997 May-Jun;15(3):357–65.
2. Linassi F, Obert DP, Maran E, et al. Implicit Memory and Anesthesia: A Systematic Review and Meta-Analysis. Life (Basel). 2021 Aug 19;11(8):850.
3. Struys M, Versichelen L, Rolly G. Influence of pre-anaesthetic medication on target propofol concentration using a 'Diprifusor' TCI system during ambulatory surgery. Anaesthesia. 1998 Apr;53 Suppl 1:68–71.
4. Linassi F, Kreuzer M, Kratzer S, et al. Unwanted spontaneous responsiveness and burst suppression in patients undergoing entropy-guided total intravenous anesthesia with target-controlled infusion: An observational prospective trial. J Clin Anesth 2023;86:111045.

**Fig. 1:**
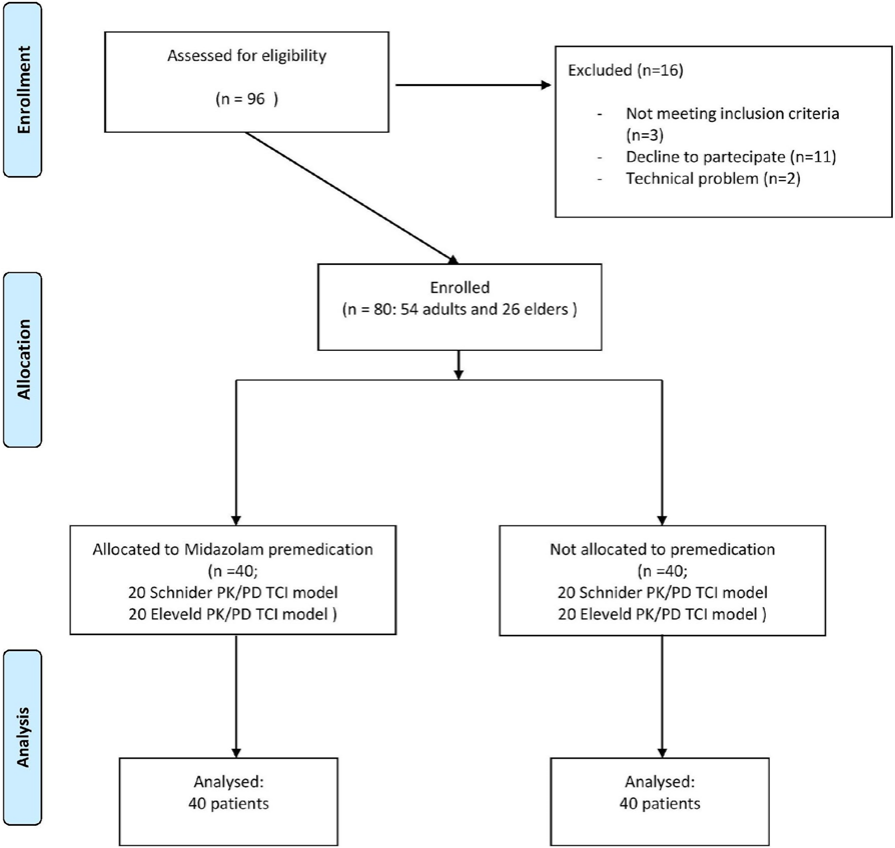
**(abstract A155).** See text for description

**Table 1:** **(abstract A155).** See text for description

	Total population			Schnider PK/PD			Eleveld PK/PD		
Variable	No Midazolam	Midazolam	p value	No Midazolam	Midazolam	p value	No Midazolam	Midazolam	p value
Age, yrs	60 [57, 65]	57 [52.2, 65]	2.44	62.4 [59, 67.1]	56.5 [51.7, 65.2]	4.16	59 [57, 65]	57.5 [52.5, 62.7]	5.36
Weight, kg	67 [62.5, 76.5]	75 [63, 83.2]	2.31	68 [61.7, 79.5]	76.5 [63.5, 83.2]	7.15	67.8 [62.5, 77.5]	75.5 [67.7, 90.5]	1.28
Height, cm	167.1 [160.5, 171]	170 [164.7, 173]	0,219	166.5 [159, 170.8]	169 [165.5, 174]	3.15	167.1 [164, 170.7]	168.9 [162.9, 175.2]	8.13
BMI, kg/m2	24.1 [22.6, 27.6]	26.3 [23.9, 28.4]	4.04	25.3 [22.6, 27.6]	26.5 [21.9, 28.4]	15.46	24.1 [22.2, 27.5]	26.3 [24.6, 29.1]	1.34
Propofol total dose, mg	531.8 [416.4, 671.3]	443.7 [347.7, 585.9]	0.12	518.5 [461.6, 78]	378.1 [302, 507.6]	0.06	545.6 [376, 648.4]	432.2 [342.9, 568.8]	9.25
Anesthesia time, min	57.5 [47.9, 71]	60 [37.5, 77.1]	6.09	55.7 [46, 69.8]	41 [32.4, 69.5]	1.08	58.1 [48.7, 65]	60.8 [52.7, 75.5]	0.52
CeP at LoR, μg/ml	2.7 [1.8, 4.1]	1.6 [0.8, 2.5]	0.01	3.9 [3.4, 4.3]	2.4 [2.1, 3.3]	0.01	1.7 [1.4, 2.3]	0.9 [0.5, 1.3]	< 0.001
CeR at LoR, ng/ml	0.8 [0.8, 0.8]	0.8 [0.8, 0.8]	5.17	0.8 [0.8, 0.8]	0.8 [0.8, 0.8]	5.17	0.8 [0.8, 0.8]	0.8 [0.8, 0.8]	5.17
CePMA, μg/ml	2.6 [2.3, 3.3]	2.2 [1.9, 2.7]	0.06	2.3 [2.1, 2.7]	2 [1.4, 2.3]	< 0.05	2.8 [2.6, 3.3]	2.6 [2.1, 4]	0.04
CeRMA, ng/ml	3 [3, 3]	2.8 [2.4, 3]	< 0.001	3 [3, 3]	2.5 [2.4, 3]	< 0.005	3 [3, 3]	2.8 [2.5, 3]	0.02
Time to CePMA, min	23.6 [19.8, 33.1]	29 [21.6, 36]	4.12	23.6 [20, 30]	27.5 [21.5, 34]	3.32	26 [18.9, 36]	28.2 [21.7, 36]	11.49
CeP at RoR, μg/ml	1 [0.7, 1.6]	1.1 [0.7, 1.6]	15.36	0.7 [0.6, 0.8]	0.6 [0.5, 0.8]	5.11	1.5 [1.2, 1.8]	1.5 [1.2, 1.9]	12.38
CeR at RoR, ng/ml	0.6 [0.5, 0.9]	0.8 [0.5, 0.9]	4.24	0.7 [0.6, 0.9]	0.7 [0.5, 1]	10.55	0.6 [0.5, 0.8]	0.8 [0.7, 0.9]	0.55
Time to RoR, min	9.1 [7, 13]	8 [6, 12]	1.02	8.5 [7, 11.6]	8 [6, 11]	9.11	9.5 [7, 12]	8 [5.6, 10.2]	5.08
Δ CeP, μg/ml	1.8 [0.3, 3]	0.5 [-0.7, 1.7]	0.04	2.9 [2.6, 3.5]	1.8 [1.4, 2.7]	0.01	0.3 [-0.2, 0.9]	-0.7 [-0.9, -0.1]	0.01
USRE, n (%)	3 (7.5)	5 (12.5)	11.52	1 (5)	0 (0)	1.00	2 (10)	5 (25)	6.47
BSupp, n (%)	14 (35)	7 (17.5)	2.06	1 (5)	3 (15)	10.05	13 (65)	4 (20)	0.01

### A156 Iron deficiency anaemia and preoperative optimization program

#### A. Gilardenghi ^1^, K. Licciardi ^1^, C. Deli ^2^, A. Florio ^1^, B. Mentore ^1^, M. Bonfiglio^1^

##### ^1^ASL 4 Liguria- Ospedale Del Tigullio, Lavagna (GE), Italy; ^2^ Università Bicocca, Milano (MI), Italy

###### **Correspondence:** A. Gilardenghi

*Journal of Anesthesia, Analgesia and Critical Care 2024*, **4(1):**A156

Iron-deficiency anaemia in patients having elective surgery is associated with spending longer time in hospital, an increase in the rate of blood transfusions, and higher mortality. In addition, receiving a red blood cell concentrate transfusion is linked with increased rates of illness, death, and length of stay in hospital.

Although the suggested benefits of PBM for both patients and the healthcare system seem the result of common sense, implementation is not necessarily straightforward and there are several hurdles to overcome. In our study, we highlight how a PBM programme requires planning, foresight, and a strong leadership. Therefore, it is imperative to rely on consensus between disciplines concerning the set of diagnostics, therapeutic and logistical interventions needed.

**Methods**

Our retrospective study refers to surgical patients admitted to our pre-reception centre, with informed consensus, in the years 2019, 2022, 2023; specifically, we examined the data associated with a particular case mix: patients undergoing elective hip and knee replacement surgeries and right haemicolectomy by videolaparoscopy.

We took in consideration the year 2023 in which it was observed the implementation of a simplified and standardised protocol with organisational solutions,and we compared it to two other reference years (2019 and 2022).

To achieve the necessary organizational adjustments, we put in place a procedure through trial periods and training sessions.

In the pre-admission assessment, the nurse case manager reviews the count data of taken blood samples (Fig. 1 operational directive 2023) and sends patients with Hb < 13 g/dL and ferritin < 100 mcg/L to the blood transfusion centre, that handles a greater number of outpatient appointments and cares for individuals in need of intravenous iron treatment.

Patients with a long waiting time (4–6 weeks) for surgery or affected by mild anaemia are referred to theirhealth care provider with an accompanying letter to undertake oral iron therapy.

**Results**

We analysed the total population of pre-hospitalisation patients and the percentage of anaemic patients (Tab. 1).

Focusing on the identified subpopulation we found a higher percentage of anaemics (Tab. 2).

Within the same population, we studied patients who had been treated with iron therapy and the percentage of transfused patients: the implementation of a uniform organisational model resulted in a rise in patient recruitment for iron treatment and a concurrent decline in the administration of allogeneic transfusions (Tab.3).

The average length of stay was calculated in days, showing a decrease of more than 48 h in hospital stay (Fig. 2).

**Conclusions**

The organisational protocolemployed in our centre has allowed a more extensive application of the PBM, which has been associated with an improving trend in the preoperative management of anaemic patients; therefore, we observed important results in decreasing the use of transfusions and the hospital length of stay.

Informed consent to publish had been obtained.

Conclusions

The organisational protocolemployed in our centre has allowed a more extensive application of the PBM, which has been associated with an improving trend in the preoperative management of anaemic patients; therefore, we observed important results in decreasing the use of transfusions and the hospital length of stay.

Informed consent to publish had been obtained.

**Fig. 1:**
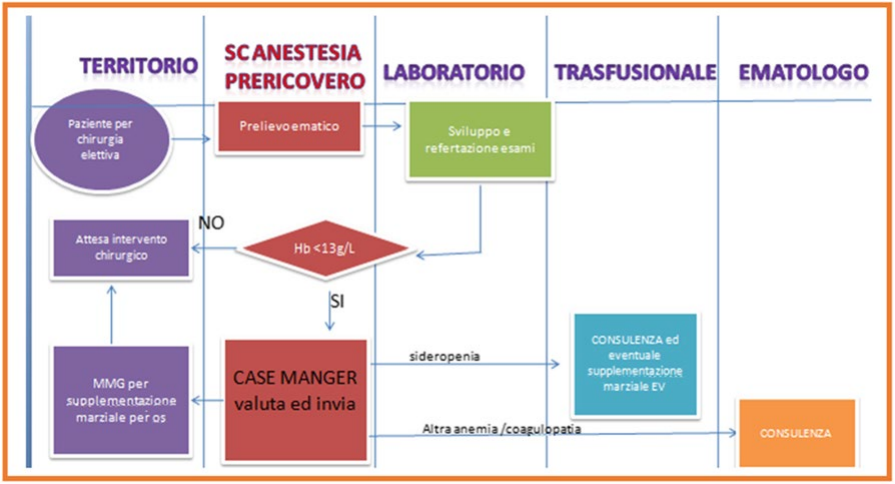
**(abstract A156).** Nursing case manager operational instruction 2023

**Fig. 2:**
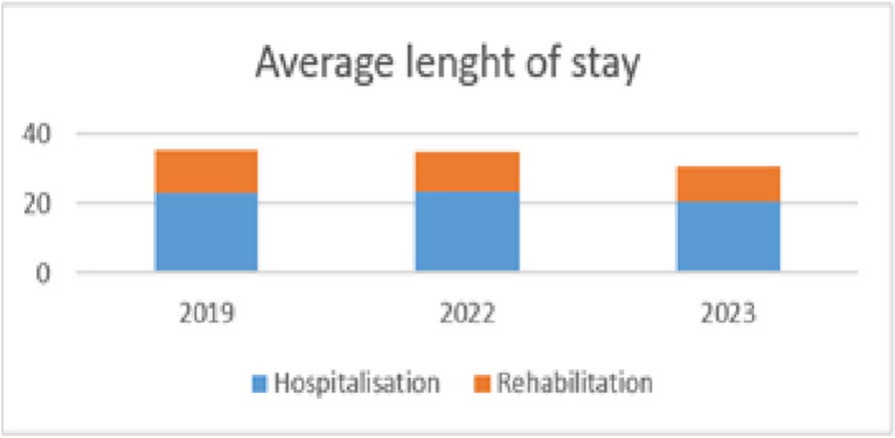
**(abstract A156).** Average length of stay in 2019, 2022, 2023

**Table 1:** (abstract A156). Pre-hospitalisation—anaemic patients

	**2019**	**2022**	**2023**
Pre-hospitalisation patients	3240	4191	5012

**Table 2:** **(abstract A156).** Analysed case mix of 3 reference years

	**2019**	**2022**	**2023**
Analysed case mix in pre-hospitalisation patients	n.257	n.285	n.238
Anaemics in case mix	35% (n.90)	35% (n.100)	31.1% (n.74)

**Table 3:** **(abstract A156).** Application of iron therapy in pre-hospitalisation

	**2019**	**2022**	**2023**
AnaemicsIV iron therapy	10,00%	38,00%	43,24%
Anaemics OS iron therapy	/	/	12,16%
**Transfused anaemic patients**	**22.6% (n.58)**	**21.4% (n.61)**	**6.3% (n.15)**

### A157 Anesthetic considerations for robot-assisted lobectomy in a patient with previous transient quadriparesis following cervical injury: a case repor

#### G. Torregiani ^1^, G. Gazzè ^2^, P. Papa ^2^, V. Ceccarelli ^2^, S. Orlando ^2^, F. Sardellitti ^1^, L. Fabbrocile ^1^, M. Covotta ^1^, F. Pierconti ^1^, C. Coccia ^1^, E.M.A. Forastiere^1^

##### ^1^ Department of Anesthesia, Intensive Care and Pain Therapy, IRCCS—Regina Elena National Cancer Institute, Rome, Italy; ^2^ Department of Anesthesia, Intensive Care and Pain Therapy, Policlinico Umberto I, Sapienza University of Rome, Rome, Italy

###### **Correspondence:** G. Gazzè

*Journal of Anesthesia, Analgesia and Critical Care 2024*, **4(1):**A157

**Background**

Transient quadriparesis (TQ), also known as cervical cord neuropraxia or spinal cord concussion, is a temporary loss of motor and/or sensory function in all four limbs, typically following excessive compression, flexion, or extension injury of the cervical spine. The more severe and long-lasting the symptoms, the greater the risk of neurological sequelae with repeated stress/injuries [1].

In this situation, anesthesiologists must make crucial decisions to ensure patient safety, especially during intubation in the sniffing position or other maneuvers requiring hyperextension of the cervical spine, as well as during positioning in lateral decubitus for lobectomy.

**Case Report**

Our clinical case involves a 54-year-old man (body weight 81 kg, BMI 25.56 kg/m2) with a history of TQ following a traumatic cervical injury during water rescue maneuvers that occurred 16 years earlier. This event resulted in sensory and motor deficits with absence of deep tendon reflexes in all four limbs, which completely resolved within six weeks with methylprednisolone administration and physical rehabilitation. No other comorbidities were reported in the patient's anamnesis. The calculated El-Ganzouri risk index for difficult airway was 3 (low risk).

Recently, following an episode of acute sciatic pain radiating to the left lower extremity, the patient underwent a spinal contrast-enhanced MRI, which detected multiple median disc protrusions and signs of a previous spinal cord injury at the C3-C4 level, consistent with the documented traumatic injury in patient's anamnesis (Fig. 1). Additionally, a collateral finding of parenchymal thickening with spiculated margins at the right upper lobe was observed, which was subsequently confirmed by contrast-enhanced CT.

Therefore, surgery for a robotic-assisted right-upper-lobectomy was indicated.

In the operating room, before the induction of general anesthesia, a disposable Philadelphia-type cervical collar was applied to stabilize the cervical spine.

Prolonged preoxygenation (5 min) under spontaneous breathing and 5 cmH2O PEEP was performed. Anesthesia was induced with Fentanyl, Propofol, and Rocuronium bromide and followed by endobronchial intubation with a left double-lumen Robertshaw tube (39 Fr) under videolaryngoscopy, with careful attention to avoid excessive force and hyperextension of the cervical spine.

Subsequently, the patient was positioned in left lateral decubitus, taking great care to keep the head and spine in axis, although protected by the cervical collar. In addition, special supports were placed between the operating bed and the patient's head.

The intraoperative course as well as the awakening were regular.

The Philadelphia-type cervical collar was removed only after extubating the patient and ruling out motor and sensory deficits through neurological objective examination.

Afterwards, the patient was transferred to the thoracic surgery department and discharged on the fourth postoperative day.

**Conclusions**

This case report highlights the challenges and strategies needed to manage patients with a history of TQ during general anesthesia and surgery in the lateral decubitus position. Spinal stabilization, careful airway management, and accurate patient positioning contribute to the successful management of this particular situation.

Informed consent to publish had been obtained.

**Reference**

1. Mayer JE, Cho SK, Qureshi SA. Cervical spine injury in athletes. Curr Orthop Pract. 2012;23(3):181–7

**Fig. 1:**
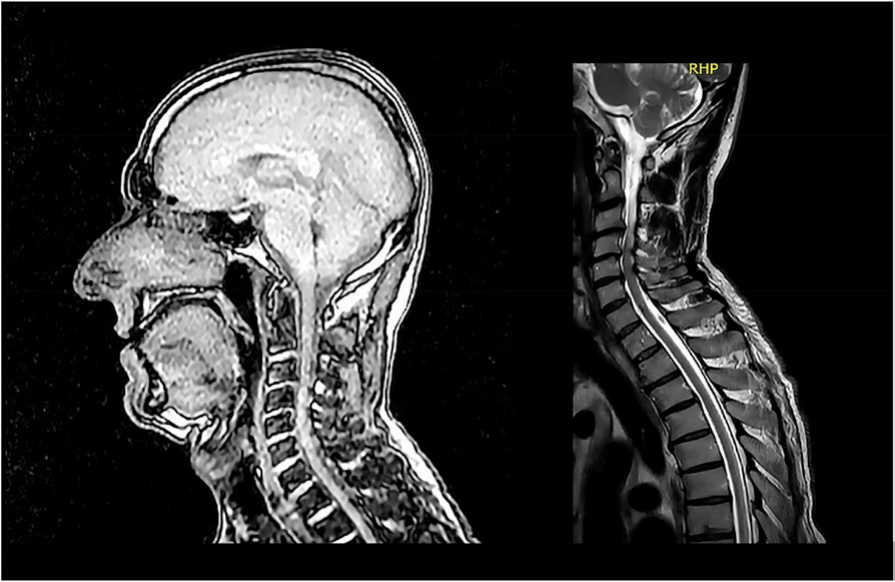
**(abstract A157).** Spinal contrast-enhanced MRI

### A158 Preoperative sarcopenia in patients scheduled for elective major surgery: risk factor modifiable with Multimodal Prehabilitation (MPREHAB)?

#### M. Feriti ^1^, F. Livi ^1^, S. Casati ^1^, L. Foti ^1^, F. Barbani ^1^, A. Durval ^1^, S. Amatucci ^1,2^, C. Tognozzi ^1,2^, A. Travelli ^1,2^, S.S. Peyrov Sajad ^1,2^, S. Chierici ^1,2^, G. Alpiciano ^1,2^, C. Fiorindi ^1,2^, G.D. Testa ^1,3^, S. Romanazzo ^1,4^, F. Cianchi ^5,6^, S. Scaringi ^5,7^, M. Cricchio ^9^, S. Romagnoli ^2,4,11^, G. Baldini^2,4,11^

##### ^1^ Centro di Preabilitazione Multimodale, Azienda Ospedaliero Universitaria Careggi, Firenze, Italy; ^2^ Dipartimento delle Professioni Tecnico Sanitarie e della Riabilitazione, Azienda Ospedaliero Universitaria Careggi, Firenze, Italy; ^3^ Divisione di Medicina Geriatrica e Terapia Intensiva, Azienda Ospedaliero Universitaria Careggi, Firenze, Italy; ^4^ Dipartimento di Scienze della Salute, Università di Firenze, Firenze, Italy; ^5^ Dipartimento di Medicina Sperimentale e Clinica, Università degli Studi di Firenze, Firenze, Italy; ^6^ Divisione di Chirurgia Digestiva, Azienda Ospedaliera Universitaria Careggi, Firenze, Italy; ^7^ IBD Unit, Chirurgia dell'Apparato Digerente, Azienda Ospedaliera Universitaria Careggi, Firenze, Italy; ^8^ Divisione di Oncologia Chirurgica e Robotica, Dipartimento di Oncologia e Robotica Ospedale Universitario Careggi, Firenze, Italy; ^9^ Dipartimento di Medicina Sperimentale e Clinica, Chirurgia Pancreatica Epato-Biliare, Università degli Studi di Firenze, Firenze, Italy; ^10^ Dipartimento di Psichiatria e Neuropsicologia, Università di Maastricht, Maastricht, The Netherlands; ^11^ Dipartimento di Anestesiologia, Università McGill, Montreal, Quebec, Canada

###### **Correspondence:** M. Feriti

*Journal of Anesthesia, Analgesia and Critical Care 2024*, **4(1):**A158

**Introduction**

MPreHab is a personalized intervention that aims at preparing high.

risk patients to better withstand the stress induced by surgery by optimizing their preoperative risk factors and their functional capacity. Sarcopenia, intended as a generalized disorder of.

skeletal muscle mass, associated with a reduction in muscle mass, strength, and physical.

performance, is linked to an increased likelihood of adverse postoperative events such as falls, fractures, disabilities, mortality, frailty, surgical complications, and hospitalization times.

**Materials and Methods**

High-risk patients who were candidates for major surgery were referred to the Multimodal Prehabilitation Center of the Careggi Hospital. The patients were evaluated before (baseline assessment) and after MPreHab (preoperatively assessment), within one week of the scheduled surgery. MPreHab included physical exercise (aerobic and resistance training), nutritional and psychological support, and medical optimization. The diagnosis of Sarcopenia was made following the diagnostic criteria of the European Working Group on Sarcopenia in OlderPeople.

**Results**

Of the 86 patients evaluated at baseline, 20 were sarcopenic (23.2%). Of these, 9 patients were no longer sarcopenic at the preoperative evaluation after MPreHab (-45%): their muscle mass strength, and their functional capacity improved (Table 1). Conversely, 5 patients who were not sarcopenic at the baseline evaluation, became sarcopenic (+ 7.5%).

**Conclusions**

MPreHab is an effective strategy to treat preoperative sarcopenia in high-risk patients scheduled for elective major surgery. In particular, sarcopenia was reduced in 45% of the patients, as demonstrated by an improvement of muscle mass, strength and functional capacity.

Informed consent was obtained.

**Table 1:** **(abstract A158).** Preoperative change in sarcopenia criteria according to the European Working Group On Sarcopenia In Older People 2 (EWGSOP2) between baseline and preoperative assessment after prehabilitation treatment. HGS (Hand Grip Strength); SPPB (Short Physical Performance Battery); TUG (Time Up and Go); FFMI (Free Fat Mass Index); ASMI (Appendicular Skeletal Mass Index). M = Male; F = Females. Data are reported as median (interquartile range)

	**Patiens no longer sarcopenic (n = 9)**		**Patiens still sarcopenic (n = 11)**	
	**Baseline assesment**	**Preoperative assesment**	**Baseline assesment**	**Preoperative assesment**
**HSG, Kg**				
*M*	24,1 (23,2–25,5)	27,2 (22,7–29,6)	21,3 ((20,2–23,3)	20,6 (19,7–22,3)
*F*	11,3 (8,5–14,2)	14,6 (12,6–16,6)	14,9 (13,9–15,9)	12,2 (11,4–13,1)
**SPPB**	10 (9–11)	11 (10–11)	8 (7–11)	9 (7–11)
**TUG, s**	9 (7,8–12,8)	8,2 (7,7–8,6)	10 (8,5–14,7)	9 (7,5–11)
**Gait Speed, m/s**	1,1 (1–1,2)	1,2 (1–1,3)	1,1 (1–1,33)	1,2 (1,2–1,4)
**FFMI, Kg/m2**				
*M*	18,6 (17,4–18,8)	18,6 (18,2–20,3)	17,7 (16,2–18,5)	17,3 (15,8–18,5)
*F*	14,6 (13,1–16,2)	16,2 (16–16,4)	14,2 (12,1–16,4)	14,7 (13,5–16)
**ASMI, Kg/m2**				
*M*	6,7 (6,3–6,7)	7,1 (6,7–7,2)	6,5 (6,3–6,6)	6,2 (6,1–6,6)
*F*	4,8 (4,4–5,2)	5,4 (5,3–5,6)	4,7 (4,2–5,3)	4,7 (4,2–5,2)

### A159 Experience with eras protocol in colorectal surgery: a preliminary report from a third-level hospital

#### G. Opramolla ^1^, A. Corrente ^1^, G. Dinatale ^2^, G. Del Vecchio ^2^, L.M.A. Cerverizzo ^1^, L.F. Mileti^1^

##### ^1^UOC Anestesia E Rianimazione—AOR San Carlo, Potenza, Italy, ^2^ UOC Chirurgia Generale—AOR San Carlo, Potenza, Italy

###### **Correspondence:** G. Opramolla

*Journal of Anesthesia, Analgesia and Critical Care 2024*, **4(1):**A159

**Background**

The Enhanced Recovery After Surgery (ERAS) protocols, originating from colorectal surgery since 2005, with the latest update in 2018, aim to minimize perioperative stress and improve recovery outcomes1. These protocols include comprehensive measures ranging from preoperative nutritional assessments to multimodal postoperative pain management, designed to reduce hospital stay lengths and morbidity rates. To assess the real-world impact of these protocols, we conducted a monocentric, prospective observational study evaluating the outcomes of ERAS protocol implementation in patients undergoing colorectal surgery.

**Methods**

In June 2023, our hospital implemented a dedicated ERAS protocol managed by a multidisciplinary team. Since July 2023, patient enrollment began. Informed consent was obtained from each patient for the publication of the data. During pre-admission, nutritional status was assessed using the Patient Generated Subjective Global Assessment (PG-SGA)2. Malnourished patients received immunonutrient supplements (three servings daily) for one week pre-surgery. All patients were given preoperative carbohydrate-rich drinks (800 ml the evening before and 400 ml two hours before anesthesia). Anesthesia involved total intravenous anesthesia (TIVA) with target-controlled infusion (TCI) of propofol and remifentanil, protective low-volume ventilation, restrictive fluid management, and normothermia maintenance. An opioid-sparing strategy was integral, using multimodal analgesia to minimize opioid use and enhance recovery. Pain control was achieved through either bilateral ultrasound-guided transversus abdominis plane (TAP) blocks, a combination of right TAP and left quadratus lumborum (QL) blocks (20 ml of 0.5% ropivacaine with 4 mg dexamethasone per side), or intrathecal morphine (150 mcg). Before the surgical incision, all patients received 1 g of intravenous paracetamol and 30 mg of ketorolac, followed by two further doses within 24 h. Pain levels and side effects were assessed postoperatively at 0 (T0), 6 (T6), 12 (T12), and 24 (T24) hours using the Visual Analog Scale (VAS). The protocol also promoted early mobilization by removing drains and catheters promptly and initiating oral intake early. Adherence to the protocol by the healthcare team was closely monitored.

**Results**

Between July 2023 and April 2024, 85 patients underwent colorectal surgeries, of which 65 were elective surgery and 20 were emergency surgery. 14 of whom followed the ERAS protocol. These patients experienced a median postoperative hospital stay of three days, significantly shorter than those receiving traditional care. Protocol adherence was high (90%), with no reoperations required within 30 days post-surgery, compared to a 6.9% reoperation rate in non-ERAS cases. Table 1 shows baseline characteristics and outcome measures.

**Conclusions**

The ERAS protocol markedly improves postoperative recovery, reduces hospital stays, and enhances pain management in colorectal surgery. Despite these benefits, the main challenge remains overcoming healthcare providers' reluctance to change established practices. Addressing these barriers is essential for wider adoption and implementation of ERAS protocols.

**References**

1. Gustafsson UO, Scott MJ, Hubner M, et al. Guidelines for perioperative care in elective colorectal surgery: Enhanced Recovery After Surgery (ERAS®) Society recommendations: 2018. World J Surg. 2019;43(3):659–95.
2. Harrie¨t Jager-Wittenaara and Faith D. Otterya. Assessing nutritional status in cancer: role of the Patient-Generated Subjective Global Assessment. Curr Opin Clin Nutr Metab Care 2017, 20:322–329.

**Table 1:** **(abstract A159).** Baseline characteristics and outcome measures.

Patient ID	Sex	Age	BMI	ASA	PG-SGA	Surgical Procedure	Analgesic Strategy	VAS T0	VAS T6	VAS T12	VAS T24	Complications
1	M	72	26	2	11-B	Robotic anterior resection	Bilateral TAP	4	5	4	4	Shivering
2	M	79	16,9	3	13-B	Robotic anterior resection	TAP + QL	4	3	2	2	Delayed awakening
3	M	57	30	1	3-A	Right emicolectomy	IM	6	6	5	5	Shivering
4	M	69	26,9	3	6-B	Robotic anterior resection + Ileostomy	TAP + QL	4	3	1	1	Hypotension
5	M	77	22,7	2	7-A	Robotic anterior resection	TAP + QL	2	1	0	0	Surgical site infection
6	M	69	34	2	7-B	Robotic anterior resection	TAP + QL	0	0	0	0	n/a
7	M	70	24,2	2	7-A	Right hemicolectomy	Bilateral TAP	5	4	2	2	Nausea
8	M	73	21,2	2	7-B	Laparoscopic sigmoidectomy	IM	2	2	2	2	Itching
9	M	60	21,8	2	8-A	Laparoscopic anterior resection	Bilateral TAP	4	3	2	2	n/a
10	M	79	25	2	6-A	Laparoscopic Left hemycolectomy	TAP + QL	5	3	3	3	n/a
11	M	77	26	2	6-A	Laparoscopic anterior resection	TAP + QL	3	2	1	1	Hypotension
12	M	54	26	2	5-A	Robotic anterior resection	TAP + QL	2	2	0	0	Nausea and vomiting
13	F	66	24,8	2	13-B	Laparoscopic right hemicolectomy	Bilateral TAP	2	2	0	0	n/a
14	F	66	23,4	2	15-A	Laparoscopic Left hemycolectomy	TAP + QL	3	3	2	2	Nausea

### A160 Determinants and mechanical effects of pneumoperitoneum-associated expiratory flow limitation during major abdominal surgery

#### F. Cinquegrana ^1^, F. Montanaro ^2^, M. Orsingher ^1^, M. Riccardo ^1^, V. Piccolo ^1^, G. Benetto ^1^, R. Ragazzi ^1,2^, M. Riccardo ^1^, M. Verri ^1^, M. Bertoni ^3,4^, F. Bongiovanni ^3^, S. Piva ^3,4^, C.A. Volta ^1,2^, S. Spadaro ^1,2^, G. Scaramuzzo ^1,2^

##### ^1^ Department of Translational Medicine, University of Ferrara, Ferrara, Italy; ^2^ Azienda Ospedaliero Universitaria Di Ferrara, Ferrara, Italy; ^3^ Department of Anesthesia, Critical Care and Emergency, Spedali Civili University Hospital, Brescia, Italy; ^4^ Departement of Medical and Surgical Specialties, Radiological Sciences and Public Health, University of Brescia, Brescia, Italy

###### **Correspondence:** F. Cinquegrana

*Journal of Anesthesia, Analgesia and Critical Care 2024*, **4(1):**A160

**Background**

Expiratory flow limitation (EFL) is associated with respiratory postoperative complications [1] and can prolong hospital recovery. Laparoscopic surgery can expose patients to develop intraoperative expiratory flow limitation (EFL), especially during the pneumoperitoneum phase. Indeed, previous evidence showed that some patients with no EFL after intubation may become flow limited during pneumoperitoneum, but the demographic and mechanical characteristics of these patients hasn’t been explored yet.

**Materials and Methods**

We enrolled patients undergoing elective laparoscopic abdominal surgery from two academic hospitals (Sant’Anna, Ferrara & Spedali Civili, Brescia). Demographic data and comorbidities were collected before surgery. To evaluate EFL, a PEEP test [2] consisting in a sudden removal of PEEP was done respectively ten minutes after induction (T1) and 10 min after pneumoperitoneum (T2). At the same time points, we collected arterial blood gas data, ventilatory and hemodynamic data. Patients were classified in two group according to the occurrence of EFL at T2: patients not EFL at both T1 and T2 (EFL -/-) and patients not EFL at T1 becoming EFL at T2 (EFL -/ +).

**Results**

107 patients, aged 69 [28–78] years, were analyzed. 21/107 (19,2%) became EFL after the induction of pneumoperitoneum. Despite we did not find difference in sex (p = 0.26) and age (p = 1.0), patients EFL -/ + showed significantly higher weight (85 [70.5–93] vs 71.5 [61.7 – 83.5] kg, p = 0.025) and had higher body mass index (27.6 [24.8–32.9] vs 25 [22.9 – 28] kg/m2, p = 0.038). At T1, patients EFL -/ + showed lower end-tidal CO2 (31 [30–34] vs 33.5 [31–36] mmHg, p = 0.035) and worse P/F ratio (249[203–369] vs 382.50[273–476]mmHg, p = 0.004), while at T2, higher driving pressure (18 [17.2–22.7] vs 17[14.75–20] cmH2O, p = 0.013), plateau pressure (22 [22 -27] vs 21.5[18.7–24] cmH2O, p = 0.005) and worse P/F ratio 244 [192–336] vs 325[269–402] mmHg, p = 0.003, Table 1).

**Conclusion**

Pneumoperitoneum may determine EFL in patients otherwise not flow limited. Higher BMI may be a risk factor for developing EFL during pneumoperitoneum. During pneumoperitoneum, patients becoming EFL had higher driving pressure, plateau pressure, and worse gas exchange. The impact of this phenomenon of pulmonary postoperative complications must be evaluated furtherly.

Informed consent was obtained.

**Refences**

1. Spadaro S, Caramori G, Rizzuto C, Mojoli F, Zani G, Ragazzi R, et al. Expiratory Flow Limitation as a Risk Factor for Pulmonary Complications After Major Abdominal Surgery. Anesth Analg 2017; 124:524–30
2. Marangoni et al. Respiratory mechanics at different PEEP level during general anesthesia in the elderly: a pilot study. Minerva Anestesiol. 2012 Nov;78(11):1205–14. Epub 2012 Jul 6. PMID: 22772859.

**Table 1:** **(abstract A160).** Principal characteristics of patients that became EFL. EFL-/- = patients do not flow limited both after anesthesia induction and during pneumoperitoneum. EFL -/ +  = patients don’t flow limited after anesthesia induction and flow limited during pneumoperitoneum. Data expressed as median [IQR] or n (%)

	**EFL -/- (86)**	**EFL -/ + (21)**	**p value**
Age (years)	66 [55–79]	68.5 [56–76]	1.0
Sex (n females, %)	39/86 (42.8%)	9/21 (43.8%)	0.259
Weight (kg)	71.5 [61.75 – 83.5]	85 [70.5–93]	0.025
BMI (kg/m^2^)	25 [22.9 – 28.05]	27.6 [24.8–32.9]	0.038
EtCO2 (mmHg)—T1	33.5 [31—36]	31 [30–34]	0.035
Driving Pressure (cmH_2_O) – T2	17 [14.75–20]	18 [17.2–22.7]	0.013
Plateau Pressure (cmH_2_O) – T2	21.5 [18.7–24]	22 [22—27]	0.005
P/F ratio (mmHg) – T1	382.5 [273–476]	249 [203–369]	0.004
P/F ratio (mmHg) – T2	325 [269–402]	244 [192–336]	0.003

### A161 Pulmonary ultrasound for the assessment of atelectasis in anesthetized children using an LMA: a randomized double-blinded CT that compares the spontaneous ventilation and pressure support ventilation

#### M. Carvalho Graça

##### Dr. Andrea CARINI, Brussels, Bélgica

*Journal of Anesthesia, Analgesia and Critical Care 2024*, **4(1):**A161

**Background**

General anesthesia (GA) is known to diminish functional residual capacity (FRC) in pediatric patients, leading to atelectasis and compromised gas exchange. The application of positive end expiratory pressure (PEEP) during GA has been observed to enhance FRC optimization. Pressure support ventilation (PSV) is also known for its potential to improve gas exchange during GA. However, there is a scarcity of data regarding the ideal PEEP and pressure support levels, for children undergoing surgical procedures with a laryngeal mask airway (LMA). To investigate the occurrence of atelectasis under PSV and spontaneous ventilation (SV), lung ultrasound (LUS) was utilized.

**Objectives**

Access the impact of inspiratory pressure support levels on the prevention of atelectasis following induction of anesthesia and residual atelectasis in the early postoperative phase using LUS, as well as, to evaluate the influence of this pressure support on ventilation parameters in pediatric patients undergoing GA with a LMA for minor outpatient surgery.

**Setting**

Single institution study,University Hospital.

**Patients**

43, American Society of Anesthesiologists physical status Class I-II, pediatric patients (12 months to 8 years), scheduled for minor outpatient surgery under standardized GA with LMA and locoregional anesthesia in supine position, with a surgery duration of less than 1 h.

INTERVENTIONS: Following approval from the ethics committee and signed parental consent,a randomized double-blinded controlled clinical trial was conducted in an ambulatory surgical unit. The patients were divided into two groups: Control group (C-group, n = 21) underwent anesthesia in SV with a constant PEEP of 5cmH2O, while the PSV-group (n = 22) received a constant PEEP of 5cmH2O and inspiratory pressure support between 5-10cmH2O, limited to a maximum of 15cmH2O (Fig. 1).The pressure support was removed upon emergence from anesthesia. Different ventilation parameters were evaluated and compared at three phases during the procedure. To assess lung aeration,Lung Aeration Score (LAS) based on LUS patterns was used in three phases of the study.

**Main outcome measures**

Evaluate and compare pulmonary aeration between SV with PEEP of 5cmH2O and PSV with a PEEP of 5cmH2O and a maximum inspiratory pressure (Pmax) of 15cmH2O by utilizing a LUS and by calculating the LAS in each step of the study protocol.

**Results**

Statistically significant differences were observed in the LAS between the two groups in all phases of the study, with higher score in the C-group vs PSV-group (P < 0.001). Significant variations were noted in ventilation parameters, including end-tidal carbon dioxide concentration (ETCO2), which was significantly higher in the C-group vs PSV-group (P < 0.001). Tidal volume was significantly lower in the C-group vs PSV-group in the first two phases of measurement (P < 0.001), and respiratory rate was significantly higher in the C-group vs PSV-group (P < 0.001). No statistically significant difference was observed in saturation levels between the two groups at T0 (P = 0.2058),T10 (P = 0.1826),T20 (P = 0.04539) or at the recovery room (P = 0.03894). Refer to Fig. 2 and Tables 1–3.

**Conclusion**

PSV has been shown to enhance ventilation parameters and mitigate the occurrence of post-induction and residual atelectasis in the immediate postoperative phase among children undergoing GA with mechanical ventilation utilizing a LMA for minor elective outpatient surgeries.

**Fig. 1:**
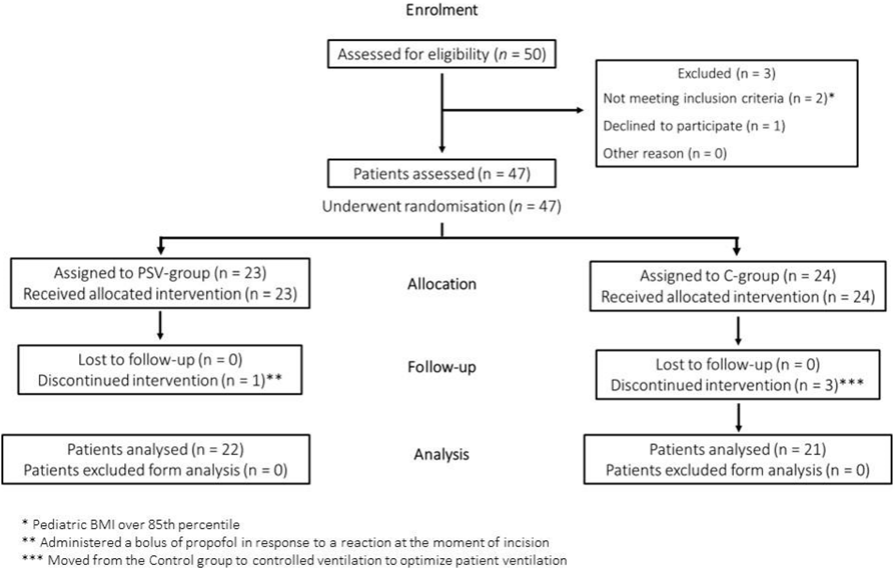
**(abstract A161).** The COSORT flow diagram

**Fig. 2:**
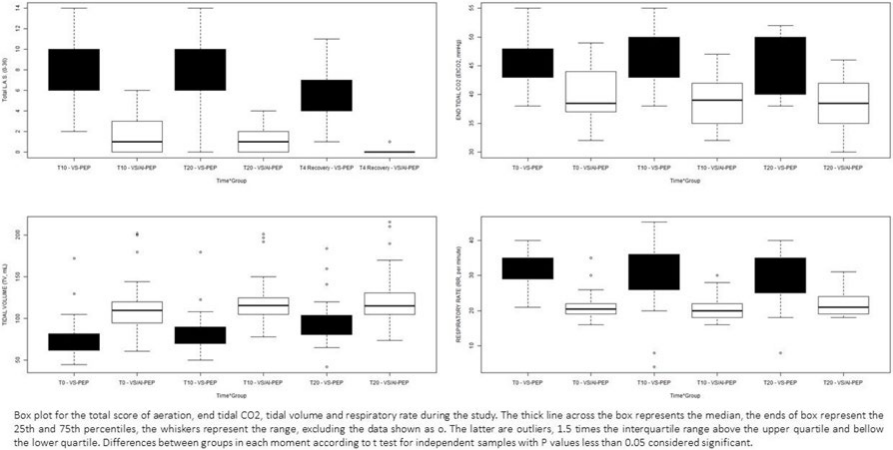
**(abstract A161).** Box plot

**Table 1:** **(abstract A161).** Primary outcomes

Variable	VS-PEP	VS/AI-PEP	p-value
Normal aeration 0 points T10	4 [2 – 6]	11 [9.25 – 12]	< 0.001
Moderate loss of aeration 1 point T10	8 [6 – 10]	1 [0 – 2.75]	< 0.001
Severe loss of aeration 2 points T10	0 [0—0]	0 [0—0]	0.3286
Complete loss of aeration 3 points T10	0 [0—0]	0 [0—0]	/
Total L.A.S. (0–36) T10	8 [6 – 10]	1 [0 – 2.75]	< 0.001
Normal aeration 0 points T20	3 [2 – 6]	11 [11 – 12]	< 0.001
Moderate loss of aeration 1 point T20	9 [6 – 10]	1 [0 – 1.75]	< 0.001
Severe loss of aeration 2 points T20	0 [0—0]	0 [0—0]	0.1522
Complete loss of aeration 3 points T20	0 [0—0]	0 [0—0]	/
Total L.A.S. (0–36) T20	9 [6 – 10]	1 [0 – 1.75]	< 0.001
Normal aeration 0 points recovery	6 [5 – 8]	12 [12 – 12]	< 0.001
Moderate loss of aeration 1 point recovery	6 [4 – 7]	0 [0 – 0]	< 0.001
Severe loss of aeration 2 points recovery	0 [0—0]	0 [0—0]	/
Complete loss of aeration 3 points recovery	0 [0—0]	0 [0—0]	/
Total L.A.S. (0–36) recovery	6 [4 – 7]	0 [0 – 0]	< 0.001

**Table 2:** **(abstract A161).** Secondary outcomes

Variable	VS-PEP	VS/AI-PEP	p-value
Age	1.92 [1.42 – 2.33]	1.29 [1.10 – 2.00]	0.03728
Weight (kg)	13 [11.60 – 16.35]	11.86 [10.45 – 13.60]	0.1141
BMI	17.13 [16.39 – 18.27]	17.88 [15.73 – 18.27]	0.3817
FiO2 T0	0.8 [0.8 – 0.8]	0.8 [0.8 – 0.8]	/
SevoEXP T0	0.03 [0.03 – 0.03]	0.03 [0.03 – 0.03]	0.9307
SpO2 T0	0.99 [0.99 – 1]	1 [0.99 – 1]	0.2058
PEP T0	5 [5 – 5]	5 [5 – 5]	/
Ai T0		7.95 ± 0.90	
Trigger T0		0.8 ± 0	
PEAK PRESSURE (Ppeak, mbar) T0	4 [3 – 5]	13 [12 – 14]	< 0.001
TIDAL VOLUME (TV, mL) T0	72 [62 – 82]	110 [95 – 120]	< 0.001
RESPIRATORY RATE (RR, per minute) T0	31.14 ± 5.29	21.59 ± 4.23	< 0.001
END TIDAL CO2 (EtCO2, mmHg) T0	46.14 ± 4.53	39.82 ± 4.69	< 0.001
FiO2 T10	0.3 [0.3 – 0.3]	0.3 [0.3 – 0.3]	/
SevoEXP T10	0.3 [0.3 – 0.3]	0.3 [0.3 – 0.3]	0.2617
SpO2 T10	0.99 [0.99 – 1]	1 [0.99 – 1]	0.1826
PEP T10	5 [5 – 5]	5 [5 – 5]	/
Ai T10		8.77 ± 0.81	
Trigger T10		0.8 ± 0	
PEAK PRESSURE (Ppeak, mbar) T10	4 [3 – 5]	14 [13.25 – 14]	< 0.001
TIDAL VOLUME (TV, mL) T10	82 [70 – 90$	116 [105 – 125]	< 0.001
RESPIRATORY RATE (RR, per minute) T10	32[26 – 36]	20 [18 – 22]	< 0.001
END TIDAL CO2 (EtCO2, mmHg) T10	46 [43 – 50]	39 [35 – 41.75]	< 0.001
FiO2 T20	0.3 [0.3 – 0.3]	0.3 [0.3 – 0.3]	/
SevoEXP T20	0.3 [0.3 – 0.3]	0.3 [0.3 – 0.3]	0.3471
SpO2 T20	0.99 [0.98 – 1]	1 [0.99 – 1$	0.04539
PEP T20	5 [5 – 5]	5 [5 – 5]	/
Ai T20		9 ± 1.02	
Trigger T20		0.8 ± 0	
PEAK PRESSURE (Ppeak, mbar) T20	4 [3 – 5]	14 [13 – 15]	< 0.001
TIDAL VOLUME (TV, mL) T20	90 [81 – 104]	115 [105.5 – 130.75]	0.002
RESPIRATORY RATE (RR, per minute) T20	28 [25 – 35]	21 [19 – 23.75]	< 0.001
END TIDAL CO2 (EtCO2, mmHg) T20	45 [40 – 50]	38.5 [35 – 41.5]	< 0.001
BLOOD OXYGEN SATURATION SpO2 (%)	1 [0.99 – 1]	1 [1 – 1]	0.03894

**Table 3:** (abstract A161). Correlations

Variable	Total L.A.S. (0–36) T10	Total L.A.S. (0–36) T20
SevoEXP T0	0.172604	0.03660593
SpO2 T0	0.1441426	0.1506006
PEAK PRESSURE (Ppeak, mbar) T0	-0.7970639***	-0.830611***
RESPIRATORY RATE (RR, per minute) T0	0.5141147***	0.5793376***
TIDAL VOLUME (TV, mL) T0	-0.4384476**	-0.469102**
RESPIRATORY COMPLIANCE (VT/(Pplateau—PEEP)) T0	-0.457328**	-0.5283469***
END TIDAL CO2 (EtCO2, mmHg) T0	0.4392392**	0.4262176**
SevoEXP T10	-0.01312824	-0.1345061
SpO2 T10	-0.009199673	-0.163623
PEAK PRESSURE (Ppeak, mbar) T10	-0.79394***	-0.8282962***
RESPIRATORY RATE (RR, per minute) T10	0.4320559**	0.4191194**
TIDAL VOLUME (TV, mL) T10	-0.4734926**	-0.5196295***
RESPIRATORY COMPLIANCE (VT/(Pplateau—PEEP)) T10	-0.3516533*	-0.454436**
END TIDAL CO2 (EtCO2, mmHg) T10	0.4551641**	0.5453022***
SevoEXP T20	0.07544607	0.06800289
SpO2 T20	-0.075735	-0.2317459
PEAK PRESSURE (Ppeak, mbar) T20	-0.8052076***	-0.8085495***
RESPIRATORY RATE (RR, per minute) T20	0.3314909*	0.4432867**
TIDAL VOLUME (TV, mL) T20	-0.3414235*	-0.4033561**
RESPIRATORY COMPLIANCE (VT/(Pplateau—PEEP)) T20	-0.3337839*	-0.38315*
END TIDAL CO2 (EtCO2, mmHg) T20	0.3577365*	0.5860963***

*p-value < 0.05

**p-value < 0.01

***p-value < 0.001

### A162 Anesthesia and analgesia for abdominal wall reconstruction

#### R. Caramia ^1^, S. Francavilla ^2^, M. Robero ^3^, A. Gallicchio ^4^, C. Lopalco ^5^, G. Bellanova ^6^, A. De Matteis ^7^, D. Lamacchia ^8^, S. Pizzoleo ^9^, L. Napolitano ^10^, S. Erario ^11^, G. Caputi ^12^, P. Fedele^13^

##### ^1^ Anesthesia, Resuscitation and Pain Therapy Unit, D. Camberlingo, Francavilla Fontana, Italy; ^2^ Anesthesia, Resuscitation and Pain Therapy Unit, D. Camberlingo, Francavilla Fontana, Italy; ^3^ Anesthesia, Resuscitation and Pain Therapy Unit, D. Camberlingo, Francavilla Fontana, Italy; ^4^ Anesthesia, Resuscitation and Pain Therapy Unit, D. Camberlingo, Francavilla Fontana, Italy; ^5^ Anesthesia, Resuscitation and Pain Therapy Unit, D. Camberlingo, Francavilla Fontana, Italy; ^6^ General Surgery Unit, D. Camberlingo Hospital, Francavilla Fontana, Italy; ^7^ General Surgery Unit, D. Camberlingo Hospital, Francavilla Fontana, Italy; ^8^ General Surgery Unit, D. Camberlingo Hospital, Francavilla Fontana, Italy; ^9^ General Surgery Unit, D. Camberlingo Hospital, Francavilla Fontana, Italy; ^10^ General Surgery Unit, D. Camberlingo Hospital, Francavilla Fontana, Italy; ^11^ General Surgery Unit, D. Camberlingo Hospital, Francavilla Fontana, Italy; ^12^ Anesthesia, Resuscitation and Pain Therapy Unit, D. Camberlingo, Francavilla Fontana, Italy; ^13^ Anesthesia, Resuscitation and Pain Therapy Unit, D. Camberlingo, Francavilla Fontana, Italy

###### **Correspondence:** R. Caramia

*Journal of Anesthesia, Analgesia and Critical Care 2024*, **4(1):**A162

**Background**

Abdominal wall defects are pathological conditions that predominantly affect subjects such as women who have had pregnancies, or those who have undergone several abdominal operations. Abdominal wall reconstruction are surgical techniques performed for the repair of abdominal hernias, which seek to restore the function and structure of the wall itself.

The repair interventions for these wall defects are of various types. We describe some effective anesthetic approaches for minimally invasive surgical interventions.

**Materials and methods**

At our hospital there is a specialized center for the repair of abdominal wall defects with a minimally invasive technique. A surgical technique used is the -minimally invasive stapled abdominal reconstruction- (MISAR) which aims to restore the anatomy and function of the abdominal wall. It allows for quicker recovery for the patient and less pain. Surgical interventions are performed under general anesthesia and various anesthetic techniques are used for postoperative analgesia.

There are no publications in international literature dealing with this new surgical technique or associated anesthesia, the choice of postoperative analgesia depends on patient-related factors and the anesthesiologist's preferences.Useful for this purpose are: epidural anesthesia with continuous anesthetic infusion for 24 h after surgery via epidural catheter; spinal anesthesia with intrathecal morphine administration only; wall anesthesia such as the TAP block, also performed by the surgeon with direct vision during laparoscopy. These procedures are superior to the administration of intravenous analgesics alone in the perioperative period.

**Results**

Each anesthetic strategy has strengths and weaknesses; therefore, it must be personalized to the individual patient following indications similar to the ERAS (Enhanced Recovery After Surgery) protocol of colorectal surgery which involves a multimodal approach to the patient. Epidural anesthesia, even if it can be modulated, increases the risk of perioperative hypotension; the use of intravenous or intrathecal morphine instead predisposes to nausea, especially in some subjects. The bilateral wall block, although effective, requires high doses of local anesthetic compared to other techniques. In our experience we recommend combining multimodal analgesia techniques with general anesthesia, because the position of the patient and the duration of the interventions, which can last up to 3–4 h or even more, would create discomfort in an awake patient even if sedated with the real risk of having to proceed with intubation and general anesthesia during surgery.

**Conclusions**

Abdominal wall surgery is continually evolving. We have moved from the open technique to minimally invasive techniques which allow the patient to recover more quickly and have less impact on pain. The MISAR technique allows the repair of major incisional hernias and diastasis of the rectus muscles with laparoscopic technique giving excellent results. The association of multimodal anesthesia/analgesia techniques gives optimal results for patient satisfaction.

Patients gave consent for publication.

### A163 Suppression-bursts-epochs during surgery-related anesthesia: a descriptive Analysis Of The Three Eeg State Space Descriptors Sigma, PHI and Omega

#### N. Bruno ^1^, W. Simone ^1^, K. Thomas ^2^, S. Claudia^1^

##### ^1^Charité-Universitätsmedizin Berlin, Berlin, Germany; ^2^ University Hospital of Psychiatry,, Bern, Switzerland

###### **Correspondence:** N. Bruno

*Journal of Anesthesia, Analgesia and Critical Care 2024*, **4(1):**A163

**Background:** State Space Descriptors, derived from multichannel EEG analyses, are global EEG parameter [Wackermann, Acta Neurobiol Exp (Wars), 1996]. These parameters change during hours of surgery-related anesthesia, depending on the duration of anesthesia, the EEG status (suppression versus non-suppression EEG) and the postoperative delirium status (POD versus no POD) [Neuner et al., Brain Communications, 2023]. The three State space descriptors Sigma (global field strength), Phi (global frequency of field changes) and Omega (the number of uncorrelated brain processes) are strongly correlated, but they are usually not analyzed in one model together. Therefore, the aim of this study was to investigate the dynamics of state space descriptors by using Mahalanobis distances—a statistical measure that accounts for correlations between variables.

**Methods:** Secondary analysis of multichannel (19 electrode positions, International 10/20 system) EEG recordings obtained from the Surgery Depth of Anaesthesia and Cognitive Outcome study, SRCTN 36437985. More than 6900 pairs of suppression / bursts EEG epochs were analyzed. Using the mean values of Sigma, Phi, and Omega, averaged across each EEG-epoch, log-transformed ratios of the three state space descriptors were calculated between consecutive epochs of suppression and bursts epochs: log(TranscriptorBursts / TranscriptorSuppression). The three ratios were regarded as one point in a 3D-space and represented overall six values of the state space descriptors (three during suppression epochs and three during the consecutive bursts epochs). Mahalanobis distances (which are not affected by the different scales of the three state space descriptors) were analyzed between consecutive points in this 3D-space. Analyses were by descriptive statistics and by linear mixed-effects models (lmer function from the lmerTest package in R [Kuznetsova A, J Stat Software, 2017]).

**Results:** Data from 48 surgical patients (mean age 70.5 years) were available. Twenty-one (43.8%) were females, and 27 (56.3%) with ASA-PS III or IV. The median anesthesia time was 208 (range 75–430) minutes. Seventeen (35.4%) patients developed POD. All three state space descriptors showed significant trajectories with ongoing anesthesia duration, both in suppression and in bursts epochs (all p < 0.001, see Fig. 1). Likewise, the log-transformed ratios of the three state space descriptors between consecutive epochs of suppression and bursts epochs significantly changed with increasing anesthesia duration (all p < 0.001, figures not shown). The Mahalanobis distances between consecutive points in this 3D-space showed a decline with ongoing anesthesia duration (predicted values per hour anesthesia duration: -0.07 (95% confidence interval: -0.09 to – 0.05), p < 0.001) and this decline varied by tendency between patients with and without subsequent POD, (contrast of patients with POD vs. patients with no POD: p = 0.099).

Conclusions: During surgery related anesthesia, global electrical brain activity exhibits distinct trajectories in both suppression and burst epochs. Moreover, these trajectories vary, in part, between patients with and without subsequent POD. Their role in predicting POD warrants further research.

**References**

1. Wackermann J. Acta Neurobiol Exp (Wars). 1996; 56(1): 197–208.
2. Neuner B, Wolter S, McCarthy MJ et al. Brain Commun. 2023; 5(6):fcad270.
3. Kuznetsova A, Brockhoff PB, Christensen RHB. J Stat Software. 2017; 82(13), 1–26.

**Fig. 1:**
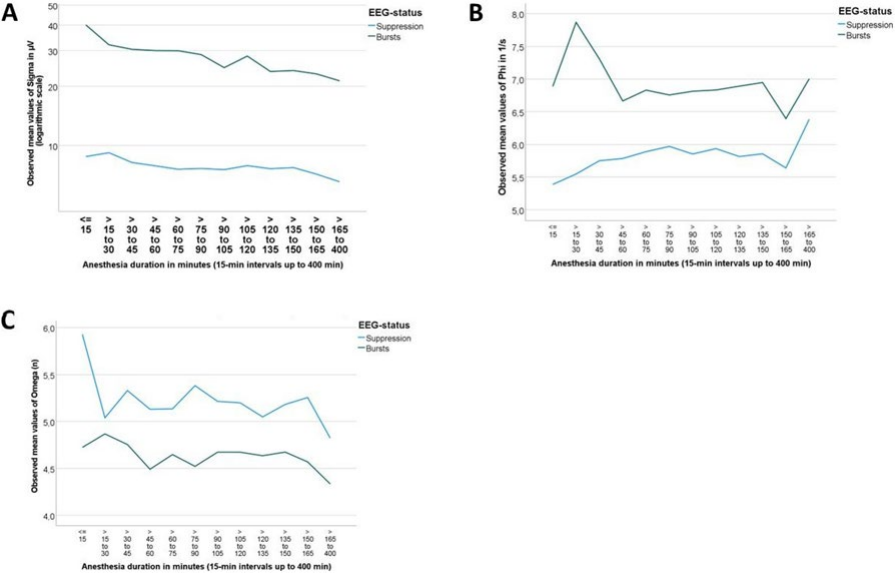
**(abstract A163).** State Space descriptors Sigma (panel A), Phi (panel B), and Omega (panel C) over ongoing surgery-related anesthesia duration (15-min intervals), n = 48 patients

### A164 Optimization of preoperative cardiological evaluation in non-cardiac surgery protocol

#### S. Biasoli, S.M. Cozzi, D.F. D'Onofrio, L. Guzzetti, B. Castiglioni, A. Bacuzzi

##### Ospedale di Circolo e Fondazione Macchi, Varese, Italy

###### **Correspondence:** S. Biasoli

*Journal of Anesthesia, Analgesia and Critical Care 2024*, **4(1):**A164

**Introduction**

Cardiological assessment in the perioperative context aims to reduce risks associated with the procedure, perioperative morbidity, and mortality, and to optimize postoperative recovery. The effectiveness of such consultation lies in its ability to influence clinical, anesthesiological, and surgical decisions through an accurate evaluation of the patient's cardiac status. This observational study aims as a preliminary objective to evaluate the actual adherence to the proposed protocol and the short-term impact on organizational structure.

**Description**

By adopting the ESC 2022 guidelines, the Hospital has implemented a standardized and multidisciplinary approach to cardiovascular risk management. This protocol integrates clinical risk factors and results of cardiological tests with the type of planned surgical procedure, allowing for personalized risk assessment. An innovative point is the introduction of a preliminary multidisciplinary assessment (cardiologist and anesthetist) of clinical documentation for selected categories of.

patients—defined as an on-desk evaluation—where the opportunity for cardiological assessments (outpatient cardiological evaluation + echocardiogram) is assessed.

**Materials and Methods**

To assess the actual effectiveness of the protocol in perioperative risk stratification and in recognizing the adequacy of cardiological evaluation requests, data collection (still ongoing) of patients requiring preoperative cardiological consultation according to the decision-making protocol at pre-hospitalization has been carried out. The decision-making process for elective NCS develops in various steps, establishing the need for cardiological consultation based on surgical timing, the risk class of the intervention (MACE classification), and a thorough clinical evaluation of the patient, with attention to the presence of cardiovascular risk factors and integrated with laboratory tests and preoperative ECG. Lastly, the introduction of risk scores (RCRI and DASI) allows for further stratification and can help support additional clinical decisions regarding the request for preoperative cardiological consultation.

**Discussion**

The analysis of data from 90 patients collected from 19/01/24, the date of introduction of the decision-making protocol, to 4/4/24, who required preoperative cardiological investigations, highlighted on one hand a good adherence to the decision-making protocol with absence of non-relevant requests especially for low and intermediate-risk MACE. It also showed how the introduction of on-desk cardiological evaluation has reduced the need for outpatient visits, thus optimizing the use of healthcare resources—only 45% of patients for whom on-desk cardiological consultation was requested needed outpatient evaluation in person.

**Conclusion**

The standardized procedure for requesting preparatory cardiological consultations aims to optimize the clinical management of patients undergoing non-cardiac surgery, reducing the risk of cardiovascular complications, and improving perioperative outcomes. The implementation of on-desk cardiological visits represents a significant advancement in the multidisciplinary treatment of surgical patients, ensuring personalized and evidence-based care, and improving the detection of previously unrecognized cardiac pathologies through an effective screening process.

**Limitations**

Data collection is ongoing, and to assess adequacy and effectiveness, it is necessary to integrate with data regarding the occurrence of perioperative cardiac complications with medium and long-term follow-up.

**Fig. 1:**
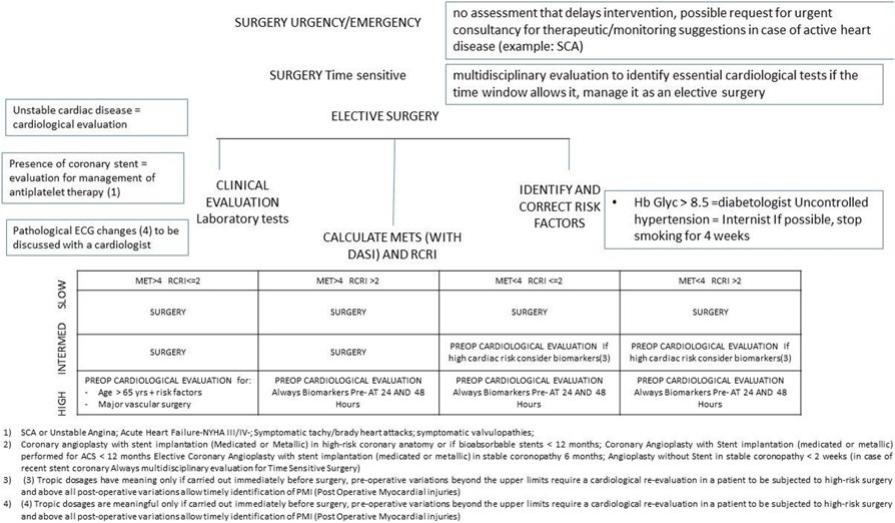
**(abstract A164).** See text for description

## Acute pain

### A165 Sphenopalatine ganglion block for the treatment of post-traumatic headache secondary to subdural hematoma and epidural cerebrospinal fluid collection. a case report

#### B. Marcellini ^1^, F. Giulietti ^2^, R. Filingeri ^2^, G. Napoli ^2^, C. Scala^2^

##### ^1^Università Politecnica delle Marche, Ancona, Italy; ^2^ Ospedale civile, Senigallia, Italy

###### **Correspondence:** B. Marcellini

*Journal of Anesthesia, Analgesia and Critical Care 2024*, **4(1):**A165

**Background**

The sphenopalatine ganglion block (SPGB) is normally used in post dural puncture headache treatment to avoid the invasive performance of the epidural blood patch (EBP).

The SPGB is a non-invasive, safe, inexpensive and well-tolerated procedure.

The main indications are severe migraine, trigeminal neuralgia and post dural puncture headache (PDPH).

In this case presented, we described a woman with post-traumatic bilateral, frontal and a nuchal headache which was present for three months in upright position and not in reclining position.

After having carried out pharmacological therapies without any benefit, the woman performed a brain and spine nuclear magnetic resonance imaging (MRI) which showed the presence of subdural hematoma in the occipito-fronto-parietal area bilaterally and a cervical-dorso-lumbar fluid collection at the level of anterior epidural space from C2 to L3, which was probably cerebrospinal fluid (CSF).

The patient received four treatments of SPGB. One month after entering the hospital, the MRI showed notable reduction of subdural hematoma with stationary CSF collection and the patient was pain-free.

**Case report**

We describe the case of L.F., a 33-year-old Moroccan woman, with post-traumatic bilateral, frontal and nuchal headache secondary to subdural hematoma and an epidural cerebrospinal fluid collection from C2 to L3.

Informed consent was obtained.

The patient received four SPGBs as described in the Table 1.

For the SPGB to be performed, the patient should be in supine position with a slight cervical extension.

For the procedure we used the SphenoCath device. The SphenoCath was first inserted into a single nostril and then advanced to the antero-superior nasal cavity, parallel to the nasal pyramid. Two milliliters of 2% lidocaine were injected via the catheter, which was then removed. This process was repeated for the contralateral nostril. The treatment was the same for all the four times.

At the end of each treatment, after 5 min, the patient was asked to sit up and assess the presence of a headache using a numeric pain score (NRS) (0-no pain to 10 – worst pain imaginable) (Table 2).

**Conclusion**

The sphenopalatine ganglion (SPG) has been identified as a site that communicates with parasympathetic autonomic nervous system and pain receptors. A blockade of SPG prevents the activation of the trigeminal-autonomic reflex, blocking vasodilating peptides and the resulting neurogenic inflammation.

The SPGB does not restore the normal cerebrospinal fluid or change the circulation dynamics, but it reduces symptoms associated with hypotension.

No major complications have been described. Nevertheless, minor bleeding due to the traumatic introduction of the applicator, paresthesia and initial discomfort of the nasopharynx, related to the spread of anesthetic, have been reported.

This case highlights the effectiveness and safety of SPGB on immediate and sustained pain relief in patients with subdural hematoma and epidural cerebrospinal fluid collection.

**Table 1:** **(abstract A165).** Temporal distance ot treatments.

I SPGB	II SPGB	III SPGB	IV SPGB
1 day	8 days	23 days	38 days

**Table 2:** **(abstract A165).** NRS before and after each SPGB.

	NRS before SPGB	NRS after SPGB
I SPGB	8	5
II SPGB	5	3
III SPGB	3	0
IV SPGB	0	0

### A166 Assessment of postoperative acute pain in thoracic surgery: how studying pain trajectories can improve acute pain service

#### M. Panizzi, V. Bellini, L.J. Darhour, M. Berdini, F. Bezzi, E. Bignami

##### Anesthesiology, Critical Care and Pain Medicine Division, Department of Medicine and Surgery, University of Parma, Parma, Italy

###### **Correspondence:** L.J. Darhour

*Journal of Anesthesia, Analgesia and Critical Care 2024*, **4(1):**A166

**Background:** Acute postoperative pain is often related to patients’ outcome. A specialist service, the Acute Pain Service (APS), is therefore often responsible for managing it. This study examines the clinical characteristics and effectiveness of acute postoperative pain management in thoracic surgery at Azienda Ospedaliero-Universitaria di Parma in 2023. It aims to correlate patients’ features with pain experience for optimal postoperative pain management. It also aims to conduct an analysis of pain trajectories, to improve the effectiveness of APS service.

**Materials and methods:** A signed and written informed consent was obtained from each patient. Retro-prospective survey, conducted at the Azienda Ospedaliero-Universitaria di Parma, with preliminary data from 96 patients undergoing thoracic surgery in 2023. Data analysis consists in comparing averages between independent samples, correlating patients’ characteristics to different levels of pain: ABSENT-MILD (NRS 0–3), MODERATE (NRS 4–6), SEVERE (NRS 7–10).

**Results:** The sub analysis includes 96 male-predominant patients (57%), with the main diagnosis being neoplasia (84%). 97% percent of study participants received blended intraoperative anesthesia (general + epidural) combined with postoperative epidural analgesia. With gold standard treatment, pain control was effective in 67% of cases, while 27% reported moderate pain and 6% reported severe pain. The data show that pain is greater in the immediate postoperative period and during movement. The initial trend of pain is similar between the two sexes. Men showed a gradual reduction over time with a mild flare-up on the third postoperative day. Younger women initially manifested more intense pain but with a more pronounced reduction than men. The pain trajectories showed more linear curves in patients with ABSENT-MILD pain than in patients with MODERATE-SEVERE pain (Fig. 1). These results are consistent with evidence in the literature1.

**Conclusions:** Blended anesthesia and postoperative peridural analgesia provided adequate pain control. The study was designed to improve our APS and ensure tailored analgesia for our patients. Pain trajectories may provide useful data to identify patients who will develop chronic pain2. For this purpose, we will implement the data with new follow-ups at 1 and 3 months, using the benefits of televisit to ensure optimal surveillance over time. We recommend a systematic data collection for anyone having an APS, to improve the service and integrate it with new technologies and new surgical and anesthesiological techniques.

**References**

1. Bendixen M, Jørgensen OD, Kronborg C, Andersen C, Licht PB. Postoperative pain and quality of life after lobectomy via video-assisted thoracoscopic surgery or anterolateral thoracotomy for early stage lung cancer: a randomised controlled trial. Lancet Oncol. 2016 Jun;17(6):836-844.
2. Liu CW, Page MG, Weinrib A, Wong D, Huang A, McRae K, Fiorellino J, Tamir D, Kahn M, Katznelson R, Ladha K, Abdallah F, Cypel M, Yasufuku K, Chan V, Parry M, Khan J, Katz J, Clarke H. Predictors of one year chronic post-surgical pain trajectories following thoracic surgery. J Anesth. 2021 Aug;35(4):505-514.

**Fig. 1:**
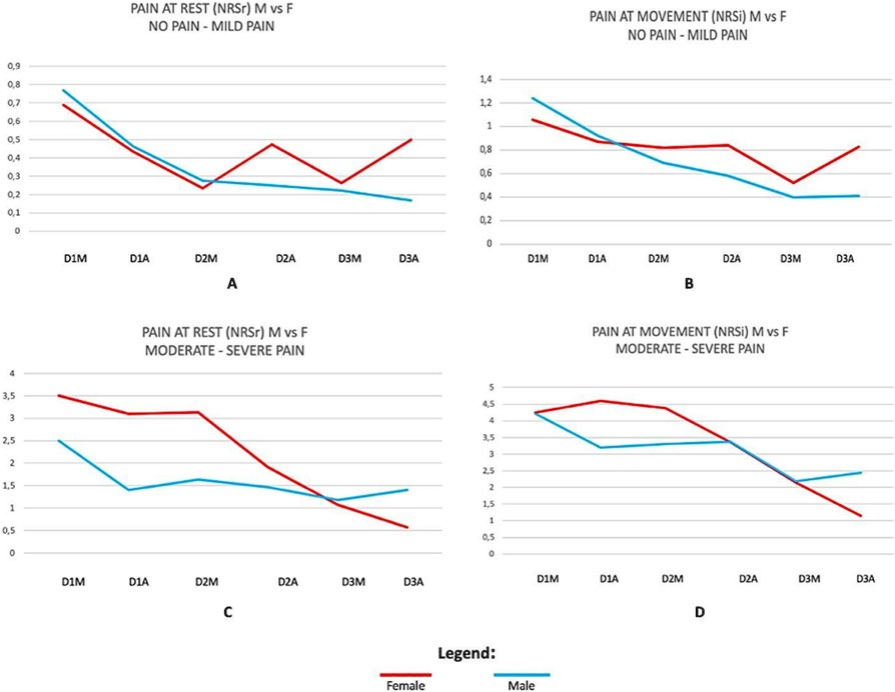
**(abstract A166).** A (NRSr M vs F no pain-mild pain) B (NRSi M vs F no pain-mild pain) C (NRSr M vs F moderate-severe pain) D (NRSi M vs F moderate-severe pain)

### A167 Postoperative pain management, automated application development for APS-POQ-R

#### V. D'Amicis ^1^, R. Ranni ^1^, M. Giglio ^2^, C. Bruzzone ^3^, S. Solari ^3^, M. Bonfiglio^3^

##### ^1^ Università degli Studi di Genova, Genova, Italy; ^2^ App Producer, ASL 4 Chiavarese, ADI, Chiavari, Italy; ^3^ Anestesia e terapia intensiva ASL 4 Chiavarese, Lavagna, Italy

###### **Correspondence:** V. D'Amicis

*Journal of Anesthesia, Analgesia and Critical Care 2024*, **4(1):**A167

Introduction

Pain is an inevitable consequence of surgery. They way it is handled is a completely subjective and multidimensional experience. Despite the development of recommendations and guidelines by scientific societies, postoperative pain is poorly managed in approximately 60%-86.7% of surgical patients and is associated with increased morbidity, impaired recovery following surgery, and decreased quality of life.

The American Pain Society's revised Patient Outcome Questionnaire (APS-POQ-R) assesses pain management strategies based on clinical outcomes, as well as patient response to pain and analgesia, patient ability to receive pain treatment information and participation in treatment, nonpharmacological pain treatment methods, and satisfaction with pain treatment. For this purpose, quality improvement (QI) validated by standardized measurements is essential. Therefore, in addition to monitoring clinical outcomes, continuous quality assessment in pain management is essential to guide effective health care and achieve a high level of patient satisfaction.

The purpose of this study is to describe the efficacy of the APS-POQ-R application as a mapping tool for specific use in the characterization and management of postoperative pain. Secondary goal is to develop a better understanding of pain and its management that may direct the drafting of a specific procedure to be applied locally.

Materials and methods

To conduct the study, our grup developed a software which was installed on a dedicated tablet. This application collects questionnaires by integrating information regarding age, sex, ASA class, type of surgery (minor, medium, major), subtype of surgery (urologic, gynecologic, and general), contraindications to NSAID/ opioid/other use, type of anesthesia (general, locoregional), type of analgesia prescribed (molecule and route of administration), nausea and vomiting (PONV), and postoperative dizziness or drowsiness.

We want to raise awareness on the importance of this study to all patients attending our prehospital through brochures (Fig. 1) and information posters (Fig. 2) that were hung in the surgical wards of our hospital. Our software records all data entered and automatically creates an excel database which continuously updates. Resident interns from the University of Genoa were trained to menage the questionnaires.

Results

Point of strenght of the software 1.Integration of an existing and validated questionnaire with information useful for the purpose of the post operative pain study. 2.The software can automatically record the medical record number by framing the bar-coded identification bracelet (there is also the possibility of manual entry) for faster registration of patients. 3. no difficulties from young doctors on using a computer application. Point of weakness: as the current gadget does not support digital signatures, consent for data management and study is currently obtained on paper, which is scanned and filed. (a gadget update could address this vulnerability).

The benefits of using computer software for data collecting that we value the most include reduced ecological impact, less data dispersion of sensitive information, and simpler data processing.

Conclusions

Our computer application can play an important role in postoperative pain control, enabling quality improvement (QI) by ensuring standardized measurement, helping healthcare in its constant challenge of achieving high level of efficiency and patient’s satisfaction.

**Fig. 1:**
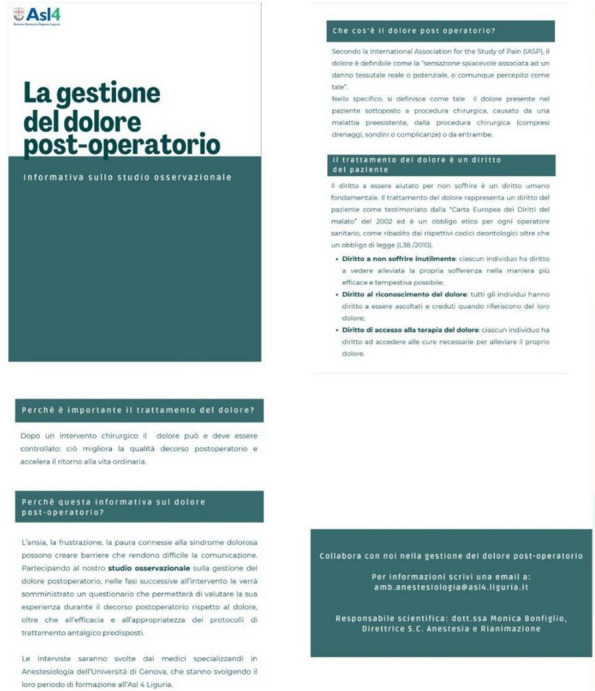
**(abstract A167).** See text for description

**Fig. 2:**
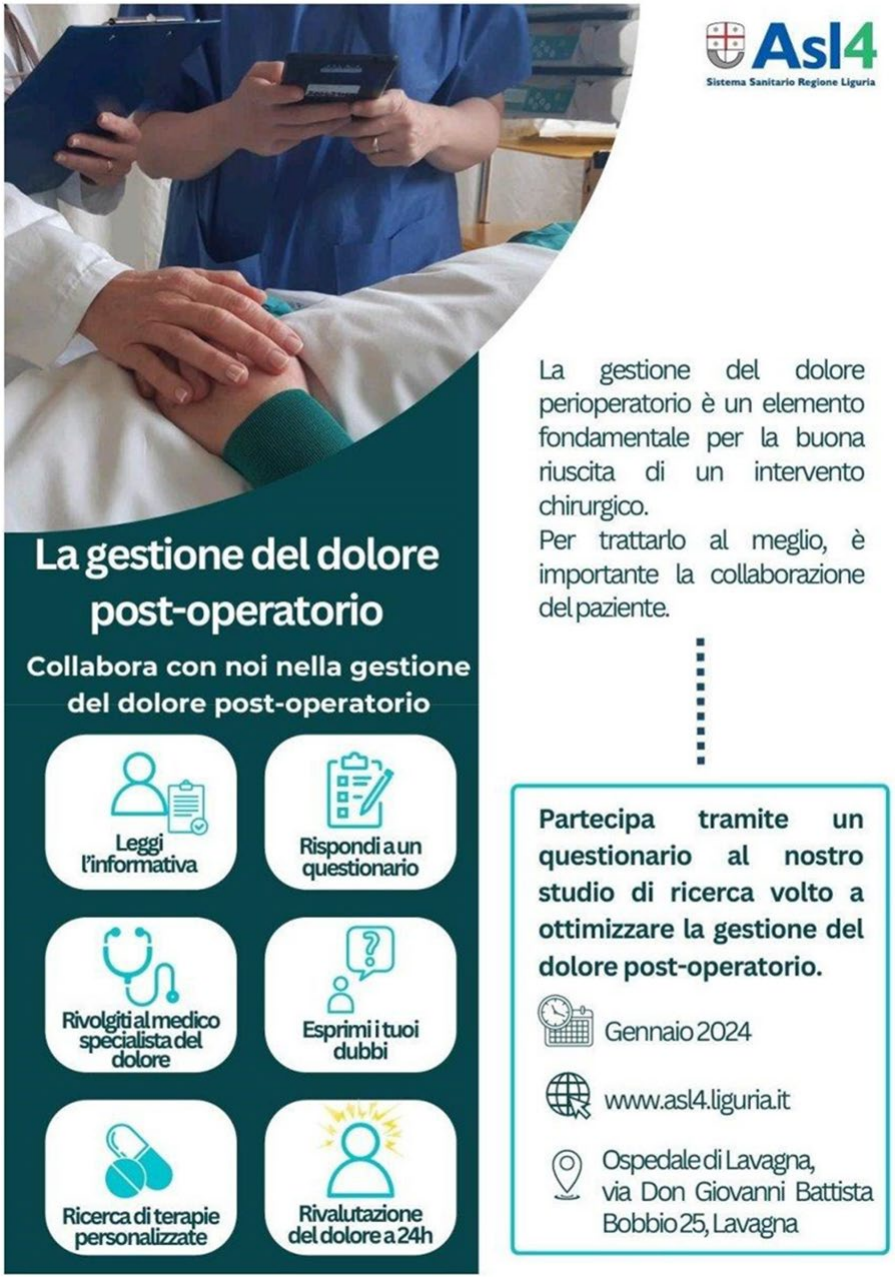
**(abstract A167).** See text for description

### A168 Regional anesthesia for postoperative pain control after open hysterectomy: the role of continuous wound infiltration. A randomized comparison with tap block

#### F. Costa ^1^, M. Cuccarelli ^1^, A. Ruggiero ^1^, G. Pascarella ^1^, A. Strumia ^1^, D. Sammartini ^1^, P. Fusco ^2^, F. Fattorini ^3^, E. Bruno ^1^, F. Plotti ^4^, M. Carassiti^1^

##### ^1^ UOC Anestesia-Rianimazione-Terapia del dolore—Fondazione Policlinico Universitario Campus Bio-Medico, Roma, Italy; ^2^ Unit of Anesthesia and Intensive Care, SS Filippo e Nicola Hospital, Avezzano, Italy; ^3^ AOU Policlinico Umberto I-Università La Sapienza, Roma, Italy; ^4^ Uoc Ginecologia—Fondazione Policlinico Universitario Campus Bio-Medico, Roma, Italy

###### **Correspondence:** M. Cuccarelli

*Journal of Anesthesia, Analgesia and Critical Care 2024*, **4(1):**A168

**Background**

Hysterectomy is commonly performed with a laparoscopic procedure, but sometimes open surgery is required and a severe postoperative pain is expected. Fascial plane blocks, such as TAP block are effective in improving pain management. We compared continuous wound infiltration (CWI) with the TAP block as regional anesthesia techniques for postoperative pain control after total abdominal hysterectomy (TAH), hypothesizing the CWI to be non-inferior to TAP block in reduce pain scores in the first 48 h.

Materials and methods: After local ethical committee approval (71.22) and registration (NCT05686382) on “clinicaltrials.gov”, we enrolled 32 patients scheduled for open TAH from January to July 2023. Patients who signed informed consent were randomly allocated into two groups. All procedures were carried out under general anesthesia. At the end of surgery, control group patients (TAP) received a postoperative bilateral ultrasound guided TAP block with ropivacaine; interventional group patients (CWI) received postoperative infusion of ropivacaine through a multi-holed catheter, placed by the surgeon in the plane between the rectus muscles and the peritoneum (preperitoneal plane) (Fig. 1).

At different time points, the difference between the groups in pain scores at rest was the primary outcome. Other outcomes were: dynamic pain; PONV; quality of recovery. Complications or undesired side effects were also recorded.

**Results**

Based on available data regarding TAP block for THA we calculated the sample size on our primary hypothesis with a 90% power to detect group differences in pain as small as approximately 1. Differences between groups were assessed by Student’s t-test for continuous parametric variables, while the Wilcoxon test was used when appropriate. The level of statistical significance was set for a p value < 0.05. Median (IQR) pain scores (Numeric Rating Scale—NRS) in the CWI group were 5 (3.75-7.75) compared to 7 (6-8) for TAP patients [p = 0.09]. At 6 h post-surgery, the scores were 3 (1.25-4) vs 7 (5.5-8) [p < 0.05] for CWI and TAP, respectively; at 12 h, the scores were 1.5 (0-3) vs 5 (5-7) [p < 0.05]; at 24 h, 1 (0-2.75) vs 4 (3.25-6) [p < 0.05]; and at 48 h, 0 (0-1) vs 3 (2-4) [p < 0.05]. [tab 1]. For dynamic pain scores we recorded similar differences as showed in [tab 2], however, no significant differences between the groups were observed for secondary outcomes, including PONV, and quality of recovery. Complications or undesired side effects were not observed.

**Conclusions**

In our trial, continuous wound infiltration with ropivacaine through a multiholed catheter placed preperitoneally, provided better analgesia compared to TAP block for postoperative pain control after TAH. We verified our hypothesis and concluded that CWI is a valid alternative to ultrasound guided fascial plane blocks for postoperative analgesia after TAH. Future trials with larger sample sizes are needed to confirm the effectiveness of this approach, also focusing on other outcomes such as length of hospital stay and costs or on developing Enhanced Recovery After Surgery (ERAS) protocols.

**Fig. 1:**
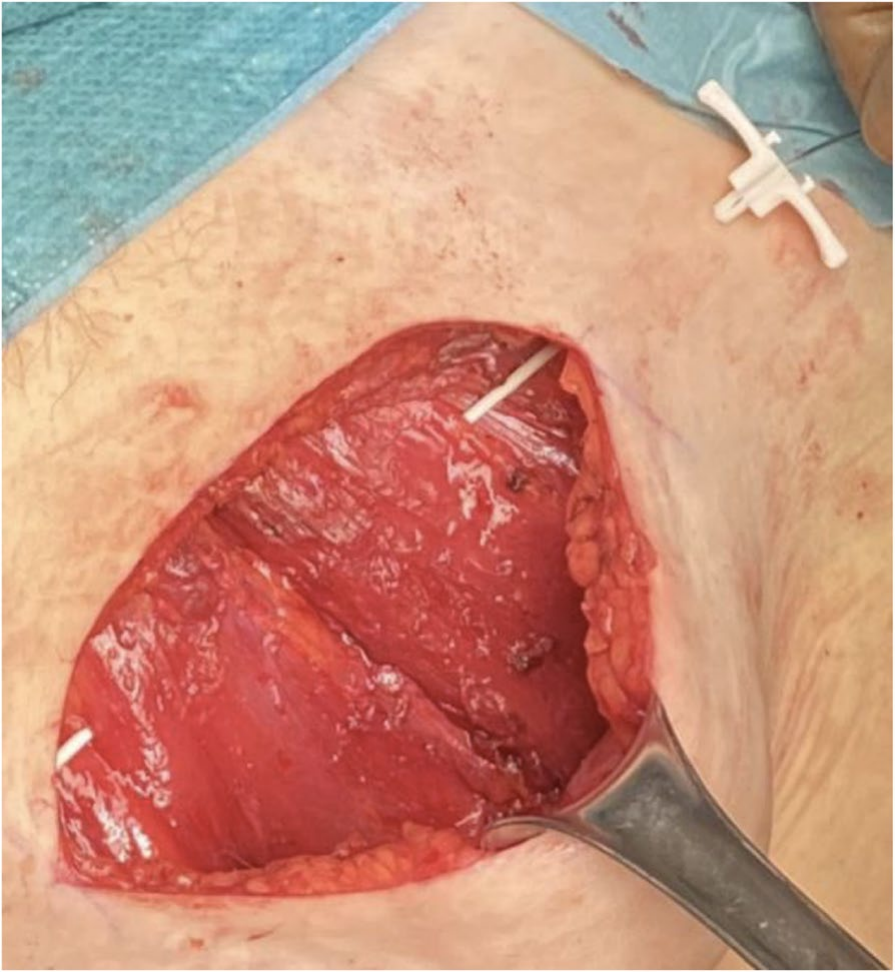
**(abstract A168).** See text for description

**Table 1:** **(abstract A168).** See text for description

	**P value**	**Mean rank of CWI**	**Mean rank of TAP**	**Mean rank diff**	**Mann–Whitney U**	**q value**
STATIC PAIN RR	0,095058	13,75	19,25	-5,5	84	0,019202
STATIC PAIN 6H	0,000025	10,06	22,94	-12,88	25	0,000006
STATIC PAIN 12H	0,000001	9,406	23,59	-14,19	14,5	< 0,000001
STATIC PAIN 24 H	0,000001	9,469	23,53	-14,06	15,5	< 0,000001
STATIC PAIN 48H	0,000021	10,03	22,97	-12,94	24,5	0,000006

**Table 2:** **(abstract A168).** See text for description

	**P value**	**Mean rank of CWI**	**Mean rank of TAP**	**Mean rank diff**	**Mann–Whitney U**	**q value**
DYNAMIC PAIN RR	0,097582	13,78	19,22	-5,438	84,5	0,019712
DYNAMIC PAIN 6H	0,000383	11	22	-11	40	0,000097
DYNAMIC PAIN 12H	0,000002	9,469	23,53	-14,06	15,5	0,000001
DYNAMIC PAIN 24 H	0,000002	9,438	23,56	-14,13	15	0,000001
DYNAMIC PAIN 48H	0,000009	9,781	23,22	-13,44	20,5	0,000003

## Invasive and interventional techniques

### A169 Cervical dorsal root ganglion pulsed radiofrequency and peridurolysis is much simpler to perform than lumbar one

#### P.P. Murdaca^1^, G. Di Gregorio^1^, L. Frigo^1^, C. Pretto^2^, C. Schiavolin^2^

##### ^1^ulss6 euganea, cittadella (PD), Italy; ^2^ Università Medicina e Chirurgia, Padova, Italy

###### **Correspondence:** P.P. Murdaca

*Journal of Anesthesia, Analgesia and Critical Care 2024*, **4(1):**A169

Cervical radicular pain is a challenging medical problem in terms of therapeutic management. Pulsed radiofrequency (PRF) stimulation on the dorsal root ganglion (DRG) has been used to control several types of chronic pain. Its effect on cervical radicular pain is still not well studied but is effective for alleviating cervical radicular pain, which was unresponsive to oral medications, physical therapy, or epidural steroid injection. This technique has gained popularity in years for both cervical and lumbosacral radicular pain

The approach can generally be:

- transforaminal/paraforaminal/extraforaminal with a needle electrode
- or intracanalicular with special multifunctional catheters, which generally allow the execution of multiple pulsed radiofrequencies and mechanical/ pharmacological epidurolysis and the injection of drugs (for example cortisone, ialunorinadisis) as well as needle,

In our clinical practice,we prefer to use the catheter, because with a single access it is possible to carry out multiple ganglion treatments and lysis of the synechiae

Although access to the sacral level may appear simpler and certainly offers greater safety due to the distance from the spinal cord compared to the thoracic and cervical level, it usually requires greater operator experience and greater exposure to ionizing radiation in the advancement and correct positioning of the tip of catheter on the dorsal ganglion target, probably for various reasons due to pathological anatomy:

- frequent, more or less severe, multifactorial stenosis (hypertrophy of the yellow ligaments and zygoapophyseal joints, disc disease with hernial degeneration);
- usual alteration of the posterior epidural plane due to rotoscoliosis e/o spondylolisthesis degeneration;
- frequent fibrotic alterations as a result of inflammatory reparative events secondary to advanced pathologies hernia or to iatrogenic invasive surgical treatments on the spine (stabilization, laminectomy, hernia removal…) but probably the physiological anatomy characteristics are more relevant:
- the double opposite curvature of the sacrum ( kyphosis) and loins ( lordosis), Fig. 1-2, which imposes on the catheter, during advancement, an adjustment along even more than three vectors;
- considerable distance of the ganglia from the catheter entry point (hiatus sacralis), with loss of the original solidity (and therefore maneuverability) of the catheter tip towards the external handpiece.

At the high thoracic level, the oblique paramedian interlaminar approach, under fluoroscopic/ultrasound guidance, although it requires greater experience with regards to finding the epidural space (considering the proximity of the medulla) allows an immediate and natural alignment of the angle of incidence of the the tuohy needle ( one vector) to the curvature of the posterior epidural plane so (Fig. 3, 4) the governability of the catheter appears more immediate and intuitive. Also associated with a lower incidence, at the cervical level, of degenerative alterations of the vertebral structures, compared to the lumbar level, it allows greater and easier navigability even in the presence of fibrosis (for example in failed back surgery syndrome), it allows a quicker execution of the procedure, less exposure to ionizing radiation (due to a reduced number of attempts to reposition the device)

Informed consent was obtained

**Fig. 1:**
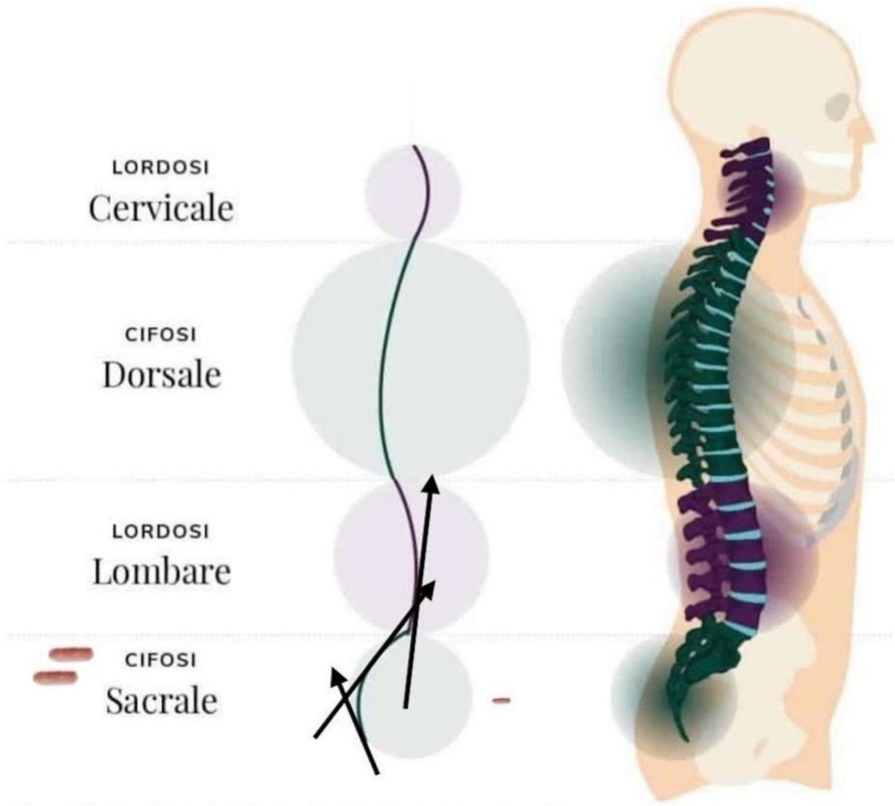
**(abstract A169).** Opposite curvature

**Fig. 2:**
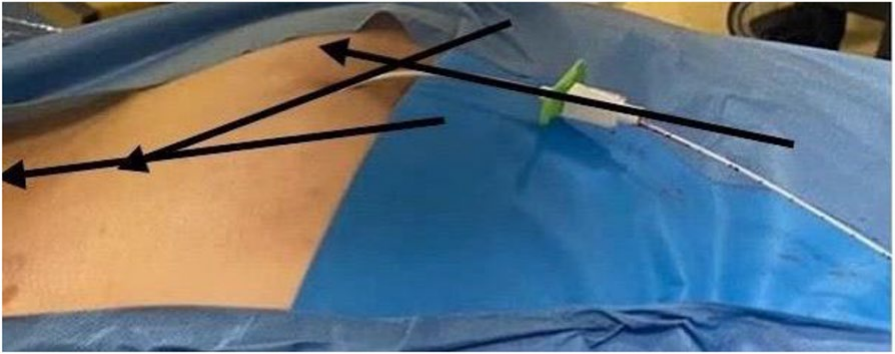
**(abstract A169).** Direction of catheter in the lumbosacral region

**Fig. 3:**
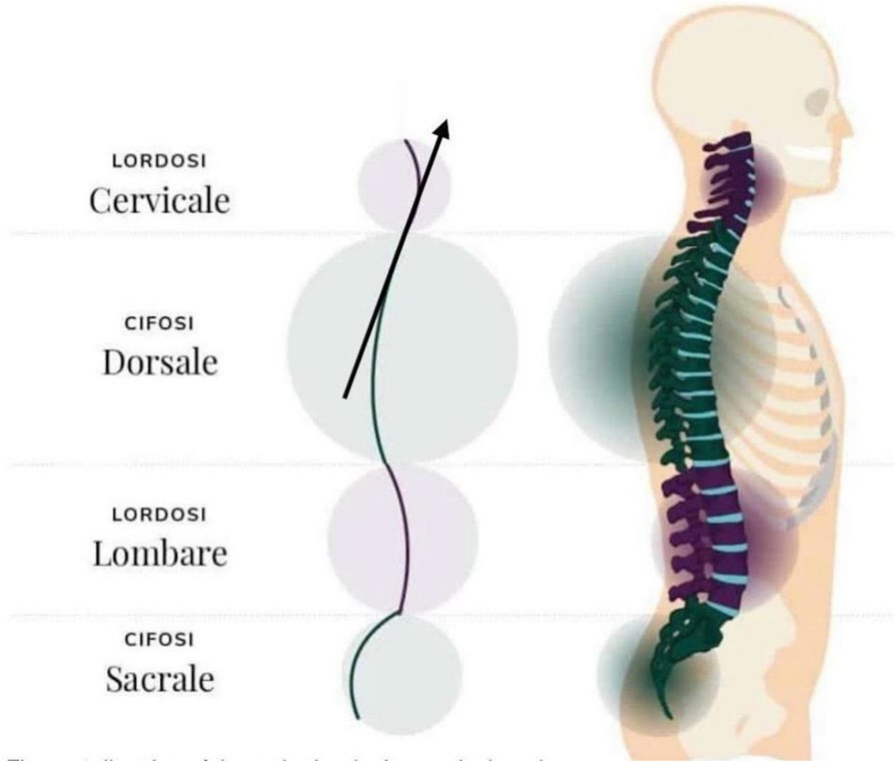
**(abstract A169).** One curvature

**Fig. 4:**
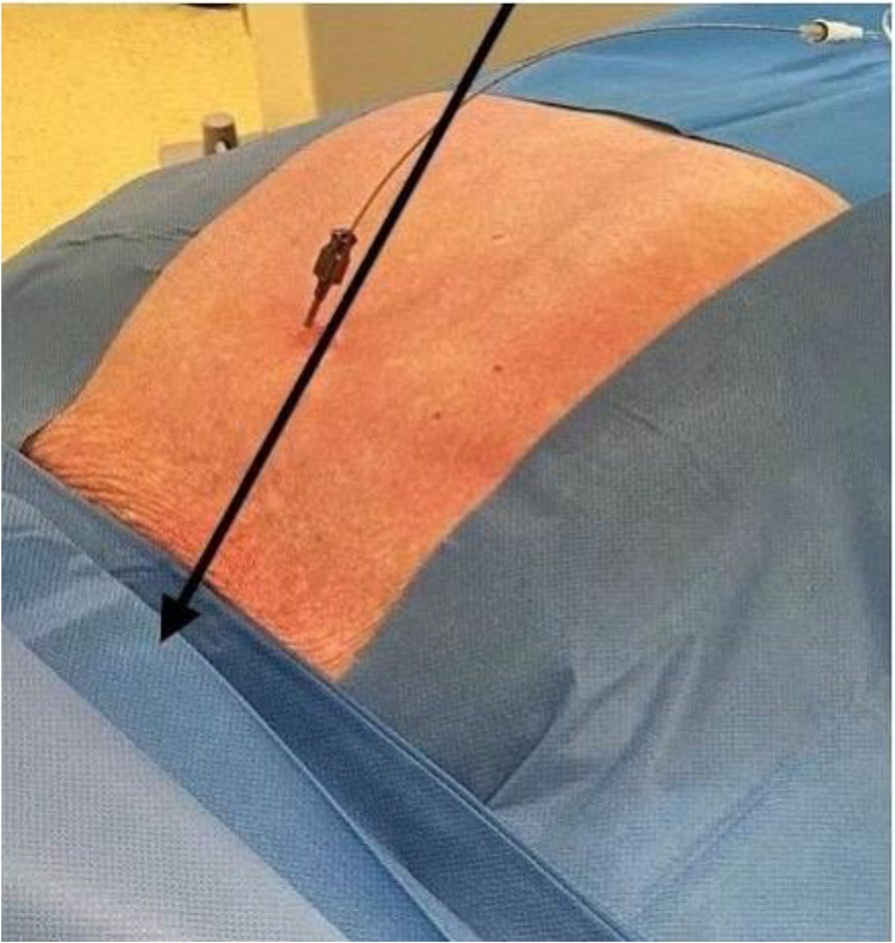
**(abstract A169).** Direction of the cathether in the cervical region

